# Identification of Key Determinants for Perceived Sweetness and Sourness in Fresh Grapes

**DOI:** 10.1002/fsn3.71824

**Published:** 2026-05-05

**Authors:** Yingjian Cai, Lingling Hu, Liufei Huang, Qianqian Zheng, Yi Qin, Yueyan Wu, Zhongyi Yang

**Affiliations:** ^1^ College of Biology and Environment Zhejiang Wanli University Ningbo China

**Keywords:** fructose, grape, perceived sourness, perceived sweetness, sensory quality, tartaric acid

## Abstract

The perception of sweetness and sourness is a critical sensory indicator of the sensory quality of table grapes. This study comprehensively analyzed the relationships between traditional physicochemical evaluation metrics (soluble solids content, SSC; titratable acidity, TAC; and their ratio, RTT) and the perceived sweetness and perceived sourness assessed by a trained sensory panel across 42 grape varieties. Our findings reveal significant discrepancies between these traditional metrics and the perceived sweetness and sourness, indicating limitations in their accuracy for precisely characterizing the sensory experience. To achieve a more accurate description of grape perceived sweetness and sourness, we employed high‐performance liquid chromatography and liquid chromatography–mass spectrometry to quantify the individual sugar and acid components within the grape juice from these 42 cultivars. Subsequently, we integrated correlation analysis and principal component analysis (PCA) for dimensionality reduction to conduct a comprehensive assessment of the sensory attributes. Ultimately, we identified tartaric acid and fructose contents as reliable key indicators for representing perceived sweetness and sourness, and the overall sensory quality of table grapes.

## Graphical abstract

This graphical abstract illustrates the identification of key indicators for perceived sweetness and sourness in fresh table grapes. Fructose and tartaric acid serve as reliable markers for sensory sweetness and sourness, performing better than conventional indices (SSC, TAC, RTT). Multivariate analysis enables effective grading of grape varieties, offering a precise method for table grape sensory quality evaluation.

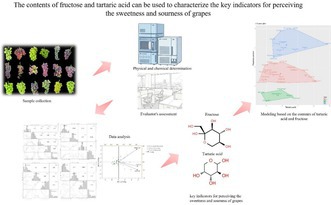

## 1 Introduction

As a globally significant fruit crop with considerable economic value and nutritional benefits, table grapes are cultivated worldwide. The perceived sweetness and sourness of table grapes is determined by the composition and concentration of sugars, acids, and volatile compounds in the fruit (Soyer et al. 2003). Sweetness (McCaughey 2008) and sourness (Frank et al. 2022) direct sensory perceptions in humans, arising from the specific composition and concentration of sugars and acids present. These taste attributes are primarily determined by the types and quantities of soluble sugars and organic acids. During consumption, the initial gustatory perception of grape berries stems from the juice released from ruptured cells, comprising cellular contents and other dissolved constituents. Consequently, research elucidating the perceived sweetness and sourness of grape berries should prioritize the measurement of juice‐soluble compounds. Current investigations, however, predominantly focus on comprehensive compositional analysis of fruit sugars and acids (Asgarian et al. 2022; Leng et al. 2022; Wang et al. 2022; Zhong et al. 2023). Direct quantitative analysis of sugar and acid profiles in freshly pressed grape juice remains limited in the current literature.

In agricultural production, soluble solids content (SSC), titratable acidity content (TAC), and their ratio (RTT) are conventional metrics for evaluating fruit maturity and taste profile (Baiano et al. 2012; Spinelli et al. 2024). These indices are widely used because they are easy to measure and are believed to reflect the balance of sugars and acids. However, preliminary physicochemical measurements in our laboratory revealed that the use of these three indicators, especially RTT, to assess the perceived sweetness and perceived sourness of the fruit is not entirely accurate. While RTT may serve as a rough guide to flavor balance, its predictive power for actual sensory perception across diverse cultivars has not been rigorously tested.

The expanding diversity of table grape cultivars in the marketplace offers increased options for consumers and producers. However, incomplete understanding of varietal perceived sweetness and sourness profiles has created two significant challenges: (1) producers frequently cultivate organoleptically similar varieties that compete directly in market channels, and (2) consumers face selection difficulties due to varying nutritional requirements, taste preferences, and health considerations. Consequently, comprehensive evaluation of perceived sweetness and sourness characteristics in commercial varieties, coupled with analysis of sugars and acids in freshly pressed juices, is essential for optimizing production practices and market efficiency.

This study selected 41 commercially representative table grape varieties based on market prevalence and regional significance. For each variety at commercial maturity, we determined SSC and TAC, while quantifying sugar and acid composition via high‐performance liquid chromatography (HPLC) and liquid chromatography–mass spectrometry (LC–MS). The research objectives were to:

- Characterize sugar and acid profiles across varieties at commercial maturity;
- Evaluate correlations between traditional indices (SSC, TAC, RTT) and perceived sweetness and sourness;
- Employ principal component analysis (PCA) to identify key taste determinants through dimensionality reduction of compositional data;
- Classify varieties by key analytical parameters to establish a selection framework for consumer preference stratification and juice industry applications.

## 2 Materials and Methods

### 2.1 Plant Material

From June to September 2024, 41 commercially significant table grape varieties were selected from a grape germplasm resource nursery under unified management in Ningbo, China. Sampling occurred at commercial maturity within a multispan steel‐frame greenhouse (single span: 7 m; gutter height: 3 m; ridge height: 5 m; length: 60 m; loam soil pH 8.58; Y‐trellis system; planting density 2 × 3 m). Fruits exhibited complete uniform coloration, slight softening without deformation, disease‐free condition, and lignified pedicels. Representative clusters were harvested from non‐adjacent vines at 1.2–1.4 m height proximal to trunks. Nine clusters per variety were randomly divided into three biological replicates (42 berries each) for sensory evaluation and physicochemical analysis. Meteorological conditions and sampling dates are detailed in Table 1.

**TABLE 1:** The 41 grape varieties tested and their harvest times at commercial maturity.

Harvest time (commercial maturity)	Variety
Late June 19 rainy days, 5 overcast days, 5 cloudy days, 1 sunny day; Average high temperature: 28°C, Average low temperature: 21°C.	‘Beda’
Early July 11 rainy days, 9 overcast days, 6 sunny days, 5 cloudy days; Average high temperature: 36°C, Average low temperature: 26°C.	‘Zuijinxiang’, ‘Zaoxiahei’, ‘Zaokangbao’, ‘Yongzaolv’, ‘Yongzaohong’, ‘Yongxiangyu’, ‘Yonglvfei’, ‘Balado Red’, ‘Balado Black’, ‘White Olin’
Mid‐July	‘Ziyuwuhe’, ‘Zitianwuhe’, ‘Zaocuixiangti’, ‘Yongfeihong’, ‘Xile’, ‘Puzhimeng’, ‘Lan Bao Shi’, ‘Gold Finger’, ‘Hutai No. 8’
Late July	‘Yuxuan No. 1’, ‘Yuxuan No. 5’, ‘Qinlongdasui’, ‘Jintianmeizhi’, ‘Rosario Rosso’, ‘Chunfeng’
Mid‐August 10 sunny days, 10 rainy days, 9 cloudy days, 2 overcast days; Average high temperature: 36°C, Average low temperature: 26°C.	‘Yinhong’, ‘Shine Muscat’, ‘Wankangbao’, ‘Moldova’, ‘Jumeigui’, ‘Kyoho grape’, ‘Golden Queen’, ‘Beni Fuji’, ‘Ruby Seedless’, ‘Takatsuma’, ‘Conglinmeigui’, ‘Amor Rose’
Early September 14 rainy days, 8 sunny days, 6 cloudy days, 2 overcast days; Average high temperature: 31°C, Average low temperature: 24°C.	‘Jade Finger’, ‘Langmanhongyan’, ‘Vitis davidii’

### 2.2 Evaluation of the Sweet and Sour Taste of Grape Fruits

A trained sensory panel (*n* = 10; 5 males, 5 females; aged 20–32 years) was established following GB/T 16291.1‐2012 guidelines. Assessors represented diverse Chinese regions with varied dietary habits. Prior to evaluation, panelists completed standardized training (GB/T 29605‐2013) to recognize reference solutions: 10 g/L sucrose (sweetness) and 0.3 g/L citric acid (sourness).

The panel testing was conducted under controlled environmental conditions (Mao et al. 2019) and followed established evaluation protocols (Colonna et al. 2004; Ferrero‐del‐Teso et al. 2022). The key parameters were as follows:

#### 2.2.1 Environment

Assessments took place in a dedicated sensory room. To minimize interpersonal influence, panelists were seated in a communal space but physically arranged to prevent visual contact or communication throughout the session. Diffuse fluorescent illumination was used, ambient temperature was maintained at 26°C, and neutral white walls and odor‐free conditions were ensured.

#### 2.2.2 Sample Presentation and Tasting Procedure

All panelists evaluated the same grape variety simultaneously. Samples were distributed by a dedicated facilitator, who presented them openly alongside the variety name; thus, evaluations were not blinded with respect to cultivar identity. For each variety, panelists sequentially tasted three peeled berries. Oral rinsing with deionized water (three times) was enforced with 30‐s intervals between samples. A mandatory 5‐min rest period was implemented between the evaluation of different varieties.

Perceived sweetness and sourness were evaluated using a structured 7‐point category scale, illustrated as follows:

Perceived sweetness: Very sweet—Moderately sweet—Slightly sweet.

Perceived sourness: Very sour—Moderately sour—Slightly sour—Not sour.

Perceived sweetness and perceived sourness were evaluated using a structured 7‐point category scale. For sweetness, the scale anchors were: 1 = Not sweet (reference: pure water), 2–3 = Slightly sweet (reference: 40 g/L sucrose), 4–5 = Moderately sweet (reference: 80 g/L sucrose), 6–7 = Very sweet (reference: 180 g/L sucrose). For sourness, the scale anchors were: 1 = Not sour (reference: pure water), 2–3 = Slightly sour (reference: 0.5 g/L citric acid), 4–5 = Moderately sour (reference: 1 g/L citric acid), 6–7 = Very sour (reference: 5 g/L citric acid). Panelists were instructed to assign integer scores based on their perception. The final perceived sweetness and perceived sourness for each variety were calculated as the arithmetic mean of the scores from all panelists.

Consensus criteria required ≥ 7/10 concordant ratings for maturity confirmation. Non‐concordant varieties underwent re‐evaluation after additional ripening. Written informed consent was obtained from all participants following institutional ethical guidelines.

### 2.3 Determination of SSC and TAC

The SSC in the grape juice was measured using a hand‐held Brix meter (Pocket Brix‐Sourness Meter Master Kit, ATAGO, Tokyo, Japan), and the TAC was determined by titrating with 0.1 M/L NaOH solution, using 1% phenolphthalein as an indicator.

Following sensory evaluation, the remaining 12 berries per variety were divided equally into three aliquots. For each aliquot, four berries were individually juiced using a stainless‐steel manual crusher simulating oral mastication mechanics. The resulting fresh juice was immediately used for SSC determination.

Residual juice from each aliquot was pooled, transferred to 1.5‐mL centrifuge tubes, and centrifuged at 158,000 *g* for 2 min. The supernatant was partitioned into three sterile microcentrifuge tubes. From each tube, 1 mL of clarified juice was diluted to 50 mL with deionized water for TAC quantification.

### 2.4 Determination of the Composition and Content of the Sugars in the Fruit Juice Using HPLC

For each variety, 100 μL of supernatant from each triplicate centrifuge tube was combined, diluted to 100 mL in a volumetric flask, and filtered through 0.22‐μm aqueous‐phase membranes into uniquely coded 1.5‐mL vials for HPLC analysis. Sugar composition (xylose, fructose, glucose, mannose, sucrose) was quantified using external calibration curves.

Chromatographic separation employed a Waters ACQUITY UPLC‐QTOF G2 system with BEH Amide column (1.7 μm, 2.1 × 100 mm). Gradient elution utilized:

Mobile phase A: Acetonitrile with 0.1% ammonium hydroxide

Mobile phase B: 0.1% ammonium hydroxide aqueous solution

At 0.15 mL/min flow rate and 40°C. The gradient program was:

0–1 min: 2.5% B

1–7 min: linear increase to 40% B

7–12 min: Return to 2.5% B

With 5‐min column re‐equilibration.

Detection used evaporative light scattering (ELSD) with drift tube temperature maintained at 70°C and N_2_ gas flow at 1.5 L/min.

### 2.5 Determination of the Composition and Content of Organic Acids in the Fruit Juice Using LC–MS

For each variety, 1 mL aliquots of supernatant from triplicate centrifuge tubes were combined, diluted to 10 mL in a volumetric flask, and filtered through 0.22‐μm aqueous‐phase membranes into uniquely labeled 1.5‐mL vials for LC–MS analysis. Organic acid composition (citric, tartaric, malic, oxalic, succinic) was quantified using external calibration standards.

Chromatographic separation employed a Waters ACQUITY UPLC‐QTOF G2 system with HSS T3 column (1.7 μm, 2.1 × 100 mm). Isocratic elution at 98% mobile phase B was maintained using:

Mobile phase A: 0.1% formic acid in acetonitrile

Mobile phase B: 0.1% formic acid in water

At 0.25 mL/min flow rate and 30°C column temperature.

Mass spectrometric detection utilized an ESI source with dual‐polarity scanning. Key parameters:

Capillary voltage: 3.0 kV (ESI ^+^), 2.2 kV (ESI ^−^)

Source temperature: 120°C

Nitrogen drying gas: 15 L/min (900 L/h).

### 2.6 Calculation and Validation of the Theoretical Sweetness and Sourness

Theoretical sweetness = fructose content × 1.7 + glucose content × 0.75 (Carocho et al. 2017).

Theoretical sourness = citric acid content × 1 + malic acid content × 0.74 + tartaric acid content × 1.21 (Mao et al. 2021).

The theoretical sweetness/sourness indices were statistically correlated with sensory perception data to evaluate their predictive accuracy across cultivars.

### 2.7 Statistical Data Analysis

Data organization was performed in Microsoft Excel. Statistical analyses were conducted using SPSS (v27.0.1) and R (v4.3.1). For sensory scores (perceived sweetness and perceived sourness), one‐way analysis of variance (ANOVA) was performed with cultivar as the fixed factor, followed by Waller‐Duncan post hoc tests (*α* = 0.05) to identify significant differences among cultivars. Pearson correlation coefficients were calculated to examine relationships among SSC, TAC, RTT, individual sugars, organic acids, theoretical indices, and sensory scores. Principal component analysis (PCA) was applied to the organic acid data (tartaric, malic, citric acids) to reduce dimensionality and identify key contributors. *K*‐means clustering (*k* = 3) was performed on the standardized fructose and tartaric acid data, and cluster visualization was created with the factoextra package (v1.0.7) in R. Correlation matrices were generated with the Hmisc package, and correlation plots were visualized using the PerformanceAnalytics package.

## 3 Results and Discussion

### 3.1 Relationships Between the SSC, TAC, and RTT of Grape Fruit and Its Taste

SSC serves as a critical maturity and taste indicator in agricultural production (Cao et al. 2010). According to industry standards (GH/T 1022‐2000), commercially mature table grapes under optimal conditions should attain SSC ≥ 15 °Brix. However, cultivation constraints including soil limitations, climatic factors, and agronomic practices result in certain varieties failing to achieve this threshold at commercial maturity.

Given that such phenotypic variation may inform varietal selection for producers under comparable growing conditions and facilitate mechanistic research, we combined the taste evaluation results from the assessors and the external characteristics of the fruit under broader standards. Varieties meeting these broader maturity standards despite substandard SSC (< 15 °Brix) were classified as commercially mature* (annotated with asterisks).

As summarized in (Table S2), the SSC of harvested grape berries ranged between 12.43 ± 0.38 °Brix (‘Zaokangbao’) and 21.93 ± 0.81 °Brix (‘Wankangbao’), with a mean value of 16.79 °Brix. TAC varied from 2.18 ± 0.15 g/L (‘Jade Finger’) to 11.88 ± 0.57 g/L (‘Beida’), yielding a mean TAC of 5.45 g/L. The raw data used to generate Table 2 are available in Table S2.

**TABLE 2:** SSC, TAC and RTT of 41 grape varieties.

	SSC (°Brix)	TAC (g/L)	RTT
‘Zuijinxiang’	15.87 ± 0.31 efgh	9.1 ± 0.11 b	1.74 opqr
‘Ziyuwuhe*’	12.93 ± 0.85 j	6.78 ± 0.71 cd	1.91 nopqr
‘Zitianwuhe’	15.23 ± 0.6 ghi	6.05 ± 0.71 cdefg	2.52 mnopqr
‘Zaoxiahei’	16.83 ± 0.15 bcdefg	6.8 ± 0.11 cd	2.48 mnopqr
‘Zaokangbao*’	12.43 ± 0.38 j	8.38 ± 0.54 b	1.48 r
‘Zaocuixiangti’	16.77 ± 0.35 bcdefg	5 ± 0.31 ghij	3.35 jklm
‘Jade Finger’	16.5 ± 0.61 bcdefg	2.18 ± 0.15 p	7.57 ab
‘Yuxuan No. 1’	21.4 ± 0.2 a	3.43 ± 0.38 klmno	6.24 c
‘Yuxuan No. 5’	18.0 ± 0.26 bcd	4.05 ± 0.46 ijklmn	4.44 fhgi
‘Yongzaolv’	18.17 ± 0.64 bc	8.1 ± 0.67 b	2.24 nopqr
‘Yongzaohong*’	13.63 ± 0.45 ij	6.33 ± 0.26 cdef	2.15 nopqr
‘Yongxiangyu’	15.47 ± 0.59 ghi	5.78 ± 0.42 defg	2.68 klmnop
‘Yonglvfei’	15.73 ± 0.35 fgh	8.18 ± 0.2 b	1.92 nopqr
‘Yongfeihong*’	13.6 ± 0.46 ij	8.15 ± 0.37 b	1.67 pqr
‘Yinhong’	17.6 ± 0.2 bcdef	3.83 ± 0.15 jklmn	4.60 fgh
‘Shine Muscat’	17.13 ± 1.11 bcdefg	2.35 ± 0.17 op	7.29 b
‘Himrod’	17.0 ± 0.3 bcdefg	3.85 ± 0.11 jklmn	4.42 fghi
‘Wankangbao’	21.93 ± 0.81 a	4.08 ± 0.37 ijklmn	5.38 cdef
‘Qinlongdasui*’	12.6 ± 0.44 j	4.5 ± 0.08 hijkl	2.80 klmno
‘Puzhimeng’	15.77 ± 0.76 fgh	5.33 ± 0.13 efgh	2.96 klmn
‘Moldova’	17.93 ± 0.06 bcde	6.43 ± 0.3 cde	2.79 kLmno
‘Langmanhongyan’	18.33 ± 0.15 b	2.2 ± 0.16 p	8.33 a
‘Lan Bao Shi’	16.17 ± 1.25 cdefg	6.95 ± 0.8 cd	2.33 mnopqr
‘Jumeigui’	21.33 ± 0.06 a	4.6 ± 0.11 hijk	4.64 fgh
‘Kyoho grape’	21.7 ± 0.1 a	5.18 ± 0.07 fghi	4.19 ghij
‘Jintianmeizhi’	15.47 ± 0.65 ghi	5.33 ± 0.6 efgh	2.90 klmn
‘Gold Finger’	16.53 ± 1.03 bcdefg	7.03 ± 1.09 c	2.35 mnopqr
‘Golden Queen’	16.73 ± 0.15 bcdefg	6.73 ± 0.04 cd	2.49 mnopqr
‘Hutai No. 8’	18.13 ± 0.06 bcd	4.58 ± 0.07 hijk	3.96 ghij
‘Rosario Rosso’	15.5 ± 0.56 ghi	4.35 ± 0.33 hijklm	3.56 ijkl
‘Beni Fuji’	21.33 ± 0.21 a	4.1 ± 0.41 ijklmn	5.20 def
‘Ruby Seedless’	16.07 ± 0.45 defg	3.3 ± 0.46 lmnop	4.87 efg
‘Balado Red’	16.8 ± 0.7 bcdefg	6.4 ± 0.34 cde	2.63 lmnopq
‘Balado Black*’	13.37 ± 2.66 j	8.4 ± 0.2 b	1.59 qr
‘Takatsuma’	16.9 ± 0.1 bcdefg	3.63 ± 0.22 klmn	4.66 fgh
‘Conglinmeigui’	16.37 ± 0.35 bcdefg	4.45 ± 0.28 hijkl	3.68 hijk
‘Vitis davidii*’	14.0 ± 0.26 hij	3.45 ± 0.3 klmno	4.06 ghij
‘Chunfeng’	18.27 ± 0.5 b	3.15 ± 0.33 mnop	5.80 cde
‘Beda’	18.4 ± 0.62 b	11.88 ± 0.57 a	1.55 r
‘White Olin’	16.43 ± 0.12 bcdefg	6.23 ± 0.27 cdef	2.64 lmnopq
‘Amor Rose’	17.93 ± 0.21 bcde	2.98 ± 0.11 nop	6.02 cd

*Note:* In one‐way ANOVA, letter designations (such as a, b, c, d) are used to indicate the significance of differences in means between groups. The same letter indicates no significant difference between groups, while different letters indicate significant differences. When the number of letters increases for the same group (such as from ab to abcd), it indicates that more differences between groups have been identified due to data variation or sample changes.

The sensory evaluation revealed a wide range of perceived sweetness and sourness ratings among the cultivars, with significant differences confirmed by ANOVA (Table 3). The raw data used to generate Table 3 are available in Table S2. For example, ‘Yuxuan No. 1’ and ‘Wankangbao’ had the highest perceived sweetness scores (both 7 ± 0), significantly higher than those of ‘Ziyuwuhe’ (2.3 ± 0.67) and ‘Yongzaohong’ (2.5 ± 0.71). For perceived sourness, ‘Beda’ (5.1 ± 0.88) and ‘Zuijinxiang’ (5.1 ± 0.88) scored highest, while many varieties, including ‘Jade Finger’ and ‘Shine Muscat’, received scores near 1.0, indicating negligible perceived sourness.

**TABLE 3:** The average results of sweetness and sourness on a 7‐point scale for different grape varieties.

	Perceived sweetness and sourness	7‐point scale sweetness	7‐point scale sourness
‘Zuijinxiang’	Moderately sweet and moderately sour	4.2 ± 0.92 ij	5.1 ± 0.88 ab
‘Ziyuwuhe*’	Slightly sweet and moderately sour	2.3 ± 0.67 mn	4.4 ± 0.97 bc
‘Zitianwuhe’	Moderately sweet and moderately sour	4.5 ± 0.85 ghij	5.2 ± 0.79 a
‘Zaoxiahei’	Very sweet and slightly sour	5.8 ± 0.92 cde	2.1 ± 0.74 ef
‘Zaokangbao*’	Moderately sweet and moderately sour	4.1 ± 1.1 ij	4.1 ± 0.74 c
‘Zaocuixiangti’	Moderately sweet and slightly sour	4.6 ± 1.07 ghij	1.9 ± 0.57efg
‘Jade Finger’	Slightly sweet and not sour	3.2 ± 0.79 kl	1 ± 0 h
‘Yuxuan No. 1’	Very sweet and not sour	7 ± 0 a	1 ± 0 h
‘Yuxuan No. 5’	Very sweet and not sour	6.8 ± 0.42 a	1.2 ± 0.42 gh
‘Yongzaolv’	Very sweet and slightly sour	6.7 ± 0.67 ab	2.4 ± 0.7 de
‘Yongzaohong*’	Slightly sweet and moderately sour	2.5 ± 0.71 lmn	5.1 ± 0.88 ab
‘Yongxiangyu’	Very sweet and slightly sour	6.9 ± 0.32 a	2.2 ± 0.63 e
‘Yonglvfei’	Very sweet and slightly sour	5.9 ± 0.74 bcde	3.1 ± 0.74 d
‘Yongfeihong*’	Slightly sweet and moderately sour	2.4 ± 0.52 lmn	4.9 ± 0.88 ab
‘Yinhong’	Moderately sweet and not sour	5.1 ± 0.57 efgh	1.4 ± 0.7 fgh
‘Shine Muscat’	Moderately sweet and not sour	5.1 ± 0.74 efgh	1.1 ± 0.32 h
‘Himrod’	Moderately sweet and slightly sour	5.1 ± 0.74 efgh	2.2 ± 0.42 e
‘Wankangbao’	Very sweet and not sour	7 ± 0 a	1 ± 0 h
‘Qinlongdasui*’	Slightly sweet and slightly sour	2.3 ± 1.06 mn	2.4 ± 0.7 de
‘Puzhimeng’	Moderately sweet and not sour	4.8 ± 0.79 fghij	1.3 ± 0.48 gh
‘Moldova’	Very sweet and slightly sour	6.3 ± 0.67 abcd	2.2 ± 1.03 e
‘Langmanhongyan’	Moderately sweet and not sour	5.3 ± 0.82 efg	1.1 ± 0.32 h
‘Lan Bao Shi’	Moderately sweet and slightly sour	4 ± 0.82 jk	3.1 ± 0.57 d
‘Jumeigui’	Very sweet and not sour	6.9 ± 0.32 a	1 ± 0 h
‘Kyoho grape’	Very sweet and not sour	6.8 ± 0.42 a	1 ± 0 h
‘Jintianmeizhi’	Slightly sweet and not sour	3.1 ± 0.74 lm	1.2 ± 0.42 gh
‘Gold Finger’	Moderately sweet and slightly sour	4.2 ± 0.92 ij	1.9 ± 0.74 efg
‘Golden Queen’	Moderately sweet and moderately sour	4.3 ± 0.95 hij	4.6 ± 1.17 abc
‘Hutai No. 8’	Very sweet and not sour	6.6 ± 0.52 abc	1.2 ± 0.42 gh
‘Rosario Rosso’	Moderately sweet and slightly sour	4.1 ± 0.88 ij	1.9 ± 0.74 efg
‘Beni Fuji’	Very sweet and not sour	6.8 ± 0.42 a	1 ± 0 h
‘Ruby Seedless’	Moderately sweet and not sour	4.9 ± 0.99 fghi	1.4 ± 0.7 fgh
‘Balado Red’	Moderately sweet and moderately sour	5.2 ± 0.63 efg	4.1 ± 0.88 c
‘Balado Black*’	Slightly sweet and moderately sour	2.3 ± 0.48 mn	5.3 ± 0.82 a
‘Takatsuma’	Moderately sweet and not sour	4.5 ± 0.85 ghij	1.2 ± 0.42 gh
‘Conglinmeigui’	Moderately sweet and slightly sour	4.2 ± 0.79 ij	2.1 ± 0.57 ef
‘Vitis davidii*’	Slightly sweet and not sour	1.8 ± 0.63 n	1.4 ± 0.7 fgh
‘Chunfeng’	Moderately sweet and not sour	5.6 ± 0.84 def	1.3 ± 0.48 gh
‘Beda’	Slightly sweet and moderately sour	1.9 ± 0.57 n	5.1 ± 0.88 ab
‘White Olin’	Very sweet and slightly sour	6.2 ± 0.79 abcd	1.9 ± 0.74 efg
‘Amor Rose’	Moderately sweet and not sour	5.3 ± 0.67 efg	1.3 ± 0.48 gh

*Note:* In one‐way ANOVA, letter designations (such as a, b, c, d) are used to indicate the significance of differences in means between groups. The same letter indicates no significant difference between groups, while different letters indicate significant differences. When the number of letters increases for the same group (such as from ab to abcd), it indicates that more differences between groups have been identified due to data variation or sample changes..

Although ‘Zaokangbao*’, with the lowest SSC (12.43 ± 0.38 °Brix), was marked as a special “commercial maturity fruit” and did not meet the industry standards for table grape maturity in China, its perceived sweetness score (4.1 ± 1.1) placed it in the moderately sweet category based on panel ratings. Notably, ‘Jade Finger’ (SSC 16.5 ± 0.61 °Brix) and ‘Beda’ (SSC 18.4 ± 0.62 °Brix) were rated as having relatively low perceived sweetness (3.2 ± 0.79 and 1.9 ± 0.57, respectively). These results indicate that SSC has limited predictive power for the actual perceived sweetness of grape berries.

**FIGURE 1:**
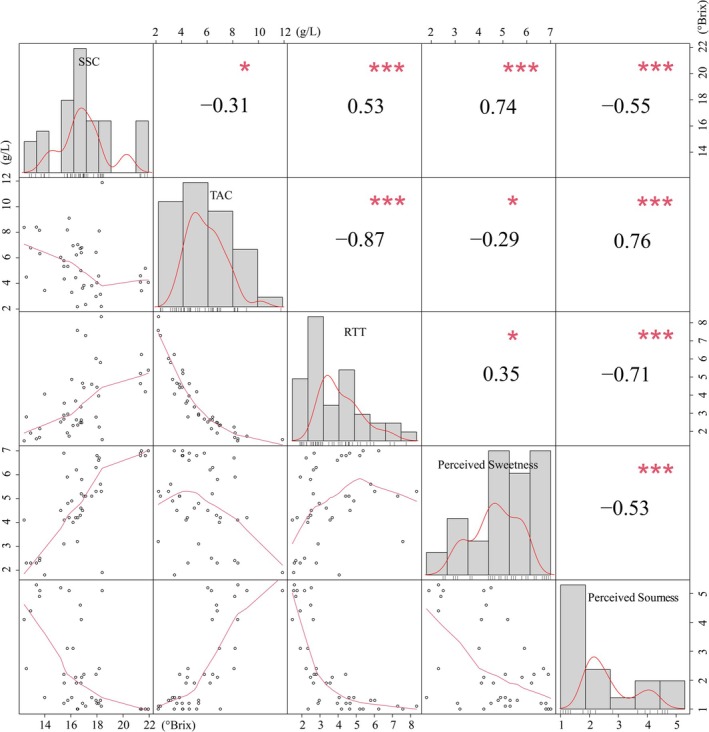
Relationships between SSC, TAC, RTT and perceived sweetness and sourness; * represents a *p* < 0.05, ** represents a *p* < 0.05, and *** represents a *p* < 0.001. The diagonal and the broken lines represent the numerical frequency distributions.

To visualize the relationships between physicochemical parameters (SSC, TAC, RTT) and sensory perception, correlation analysis was performed using the mean panel scores on the 7‐point scale (Figure 1). Perceived sweetness was highly significantly and positively correlated with SSC (*r* = 0.74, *p* < 0.001). Perceived sourness demonstrated a highly significant negative correlation with both SSC (*r* = −0.55, *p* < 0.001) and RTT (*r* = −0.71, *p* < 0.001), but a highly significant positive correlation with TAC (*r* = 0.76, *p* < 0.001). These findings indicate significant mutual suppression between sweetness and sourness in commercially mature grapes, with sweetness exerting stronger inhibitory effects on sourness perception. This phenomenon aligns with established taste interaction mechanisms reported in sensory literature (King et al. 2006; Marsh et al. 2004; Pelletier et al. 2004; Yan et al. 2024).

Notably, the RTT demonstrated limited predictive value for sensory evaluation, exhibiting a highly significant inverse correlation with perceived sourness that was not as strong with perceived sweetness. This finding indicates that RTT may be unsuitable for predicting the perceived sweetness and sourness of grape berries across different cultivars. This is likely because grapes are direct hexose accumulators (Alem et al. 2021; Coetzee et al. 2019; Xu et al. 2015); sugar accumulation occurs primarily through sucrose cleavage rather than acid‐to‐sugar interconversion. Although our correlation analysis revealed an inverse relationship between SSC and TAC (Figure S1), this represents a correlational relationship without direct metabolic causation.

Collectively, these experimental and statistical findings demonstrate that while SSC and TAC are valid parameters for assessing maturity, they have limited utility for predicting the actual sensory perception of sweetness and sourness across diverse cultivars. RTT, in particular, proves unreliable for sensory quality assessment.

### 3.2 Analysis of the Composition and Content of Sugars and Acids in the Different Grape Varieties

These findings demonstrate that exclusive reliance on SSC and TAC provides insufficient accuracy for evaluating sensory quality across grape cultivars. Perceived sweetness and sourness involve complex chemosensory transduction mechanisms, where perceived intensity depends fundamentally on ligand concentration at taste receptors (Chaudhari and Roper 2010; Keast and Breslin 2003). The experimental results revealed that table grapes have a very high water content, making it easy to extract juice even using a manually pressed metal juicer instead of an electric homogenizer. In previous grape drying experiments, the average initial water content of the selected grape samples reached as high as 79.8% ± 0.1% (Essalhi et al. 2018). In addition, previous studies have shown that vacuoles are the primary site for the accumulation of sugars, water, and organic acids in grape fruits (Bai et al. 2023; De Angeli et al. 2013; Keller and Shrestha 2014). During mastication, taste bud activation occurs primarily through juice released from ruptured vacuoles, justifying the quantification of perceived sweetness and sourness via juice solute analysis.

To elucidate varietal differences in sugar/acid profiles and their sensory implications, we conducted targeted quantification using HPLC and LC–MS.

Quantitative profiles are detailed in Table 4. The raw data used to generate Table 4 are available in Table S1 and Figure S1.

**TABLE 4:** Sugar and acid contents of the different grape varieties.

	Tartaric acid content (g/L)	Citric acid content (g/L)	Malic acid content (g/L)	Glucose content (g/L)	Fructose content (g/L)
‘Zuijinxiang’	7.21 ± 0.18 c	0.16 ± 0.01 mno	2.26 ± 0.12 c	77.11 ± 0.74 pq	80.08 ± 1.77 mnop
‘Ziyuwuhe*’	5.1 ± 0.2 fg	0.13 ± 0.01 qrs	1.18 ± 0.02 hijkl	53.04 ± 6.34 v	58.97 ± 3.82 rs
‘Zitianwuhe’	4.31 ± 1.23 ij	0.4 ± 0.02 c	1.75 ± 0.49 de	67.4 ± 2.29 rs	77.41 ± 1.34 nop
‘Zaoxiahei’	4.51 ± 0.05 hi	0.18 ± 0.01 jkl	1.17 ± 0.03 hijkl	110.55 ± 0.27 e	112.64 ± 1.5 cd
‘Zaokangbao*’	5.58 ± 0.22 e	0.34 ± 0.01d	4.58 ± 0.22 a	79.12 ± 1.55 op	89.88 ± 1.23 ijk
‘Zaocuixiangti’	3.44 ± 0.17 lm	0.15 ± 0 opq	1.69 ± 0.1 def	84.25 ± 2.34 mn	85.25 ± 2.98 klm
‘Jade Finger’	2.31 ± 0.03 pq	0.05 ± 0 v	0.59 ± 0.02 pq	70.16 ± 0.87 r	62.65 ± 0.21 qr
‘Yuxuan No. 1’	4.21 ± 0.05 ij	0.15 ± 0 no	0.87 ± 0.02 lmnop	123.96 ± 1.15 c	131.93 ± 0.44 a
‘Yuxuan No. 5’	4.09 ± 0.26 ijk	0.18 ± 0 j	0.8 ± 0.02 mnopq	109.63 ± 2.01 e	117.27 ± 2.9 bc
‘Yongzaolv’	8.87 ± 0.23 a	0.31 ± 0.01 e	1.74 ± 0.06 de	112.1 ± 0.39 e	117.45 ± 2.07 bc
‘Yongzaohong*’	4.31 ± 0.31 ij	0.28 ± 0.01 f	1.65 ± 0.16 def	62.93 ± 2.29 t	66.42 ± 1.75 q
‘Yongxiangyu’	3.6 ± 0.06 lm	0.23 ± 0.01 g	1.31 ± 0.08 ghi	93.45 ± 2.02 gh	103.82 ± 2.06 f
‘Yonglvfei’	3.72 ± 0.31 kL	0.33 ± 0.03 d	2.96 ± 0.78 b	92.7 ± 0.47 ghi	105.12 ± 0.92 ef
‘Yongfeihong*’	5.49 ± 0.22 ef	0.28 ± 0.01 f	1.9 ± 0.14 d	56.52 ± 2.1 u	58.4 ± 1.04 rs
‘Yinhong’	2.47 ± 0.04 opq	0.16 ± 0 mno	0.72 ± 0.04 nopq	81 ± 1.17 no	77.06 ± 0.17 nop
‘Shine Muscat’	2.7 ± 0.1 nop	0.13 ± 0 qrs	0.78 ± 0.04 mnopq	82.56 ± 1.04 mno	80.02 ± 3.39 mnop
‘Himrod’	4.29 ± 0.08 ij	0.04 ± 0 v	0.29 ± 0.01 rs	89.42 ± 1.4 ij	80.08 ± 1.45 mnop
‘Wankangbao’	4.46 ± 0.12 hi	0.16 ± 0 lmn	0.98 ± 0.03 klmn	116.87 ± 2.08 d	119.18 ± 1.46 b
‘Qinlongdasui*’	3.84 ± 0.1 jkl	0.13 ± 0 rs	1.04 ± 0.03 ijklm	49.13 ± 0.57 w	60.71 ± 2.52 r
‘Puzhimeng’	3.59 ± 0.05 lm	0.15 ± 0 opq	1.44 ± 0.06 fgh	90.52 ± 1.67 hij	94.72 ± 1.53 ghi
‘Moldova’	6.64 ± 0.12 d	0.24 ± 0.01 g	0.66 ± 0.02 opq	106.18 ± 2.08 f	112.74 ± 2.08 cd
‘Langmanhongyan’	2.1 ± 0.03 q	0.14 ± 0 pqr	0.57 ± 0.03 pq	84.45 ± 0.71 mn	76.68 ± 1.31 op
‘Lan Bao Shi’	4.49 ± 0.33 hi	0.16 ± 0 no	1.51 ± 0.04 efg	85.4 ± 0.27 klm	97.2 ± 1.28 g
‘Jumeigui’	5.57 ± 0.4 e	0.23 ± 0.01 g	1.22 ± 0.03 ghijk	129.52 ± 2.3 ab	134.81 ± 15.59 a
‘Kyoho grape’	4.9 ± 0.39 gh	0.2 ± 0 hi	0.82 ± 0.03 mnop	126.76 ± 2.77 bc	134.64 ± 2.69 a
‘Jintianmeizhi’	3.51 ± 0.03 lm	0.18 ± 0 jkl	0.99 ± 0.02 jklmn	74.7 ± 1.06 q	82.53 ± 2.45 lmno
‘Gold Finger’	3.17 ± 0.01 mn	0.18 ± 0.01 jk	1.67 ± 0.1 def	82.68 ± 1.9 mno	83.25 ± 0.37 lmn
‘Golden Queen’	5.29 ± 0.16 efg	0.46 ± 0.01 b	1.32 ± 0.04 ghi	84.88 ± 1.56 lm	90.71 ± 0.54 hijk
‘Hutai No. 8’	4.14 ± 0.14 ijk	0.14 ± 0 qrs	0.92 ± 0.02 klmno	104.56 ± 1.71 f	110 ± 0.96 de
‘Rosario Rosso’	4.31 ± 0.09 ij	0.21 ± 0 h	1.08 ± 0.03 ijklm	64.91 ± 2.73 st	74.19 ± 0.21 p
‘Beni Fuji’	3.4 ± 0.19 lm	0.14 ± 0.01 qrs	0.5 ± 0.02 qr	130.88 ± 2.43 a	122.32 ± 7.56 b
‘Ruby Seedless’	2.66 ± 0.09 op	0.13 ± 0 rs	0.66 ± 0.02 opq	81.82 ± 2.32 mno	85.51 ± 1.4 klm
‘Balado Red’	6.59 ± 0.35 d	0.15 ± 0 nop	1.29 ± 0.07 ghij	82.58 ± 1.61 mno	87.46 ± 1.29 jkl
‘Balado Black*’	7.8 ± 0.05 b	0.19 ± 0 ij	2.17 ± 0.2 c	54.93 ± 1.74 uv	75.41 ± 2.41 p
‘Takatsuma’	2.65 ± 0.09 op	0.16 ± 0.01 lmn	0.81 ± 0.01 mnopq	94.24 ± 0.74 g	93.33 ± 3.15 ghij
‘Conglinmeigui’	2.9 ± 0.07 no	0.08 ± 0 u	1.03 ± 0.05 ijklm	88.06 ± 1.88 jkl	87.54 ± 1.24 jkl
‘Vitis davidii*’	2.79 ± 0.11 no	0.12 ± 0 s	0.22 ± 0 s	58.28 ± 1.1 u	53.45 ± 0.29 s
‘Chunfeng’	3.14 ± 0.07 mn	0.17 ± 0 jklm	0.94 ± 0.03 klmno	88.53 ± 1.55 jk	96.51 ± 3.58 gh
‘Beda’	6.71 ± 0.01 d	0.64 ± 0.01 a	1.51 ± 0.03 efg	93.13 ± 2.73 gh	81.88 ± 2.29 lmno
‘White Olin’	5.67 ± 0.16 e	0.1 ± 0 t	0.96 ± 0.04 klmno	126.05 ± 1.15 c	110.8 ± 2.56 d
‘Amor Rose’	3.52 ± 0.11 lm	0.17 ± 0 klmn	0.95 ± 0.04 klmno	96.18 ± 2.33 g	102.6 ± 1.83 f

*Note:* Different letters within the same column indicate significant differences among cultivars (*p* < 0.05, Waller‐Duncan test).

Within the analytical parameters of this study, only glucose, fructose, tartaric acid, malic acid, and citric acid were quantifiable across the 41 grape juice samples. Other potential constituents fell below detection limits, aligning with prior juice‐based investigations (Chen et al. 2015; Coelho et al. 2018; Dos Santos Lima et al. 2014; Liu et al. 2007). Notably, sucrose remained undetectable in all freshly pressed juices. This finding contrasts with studies employing whole‐fruit sugar extraction (Asgarian et al. 2022; Leng et al. 2022; Wang et al. 2022; Zhong et al. 2023) but corroborates juice‐specific analyses (Da Silva et al. 2019; Scettri et al. 2024).

Two mechanistic hypotheses may explain this phenomenon: On one hand, sucrose predominantly localizes within phloem and vascular tissues (Wang et al. 2022), whose structural integrity resists breakdown during manual pressing; on the other hand, significant invertase activity rapidly hydrolyzes released sucrose into glucose and fructose.

This enzymatic explanation aligns with recent findings (Scettri et al. 2024), providing the most probable basis for sucrose's consistent absence in pressed grape juice.

**FIGURE 2:**
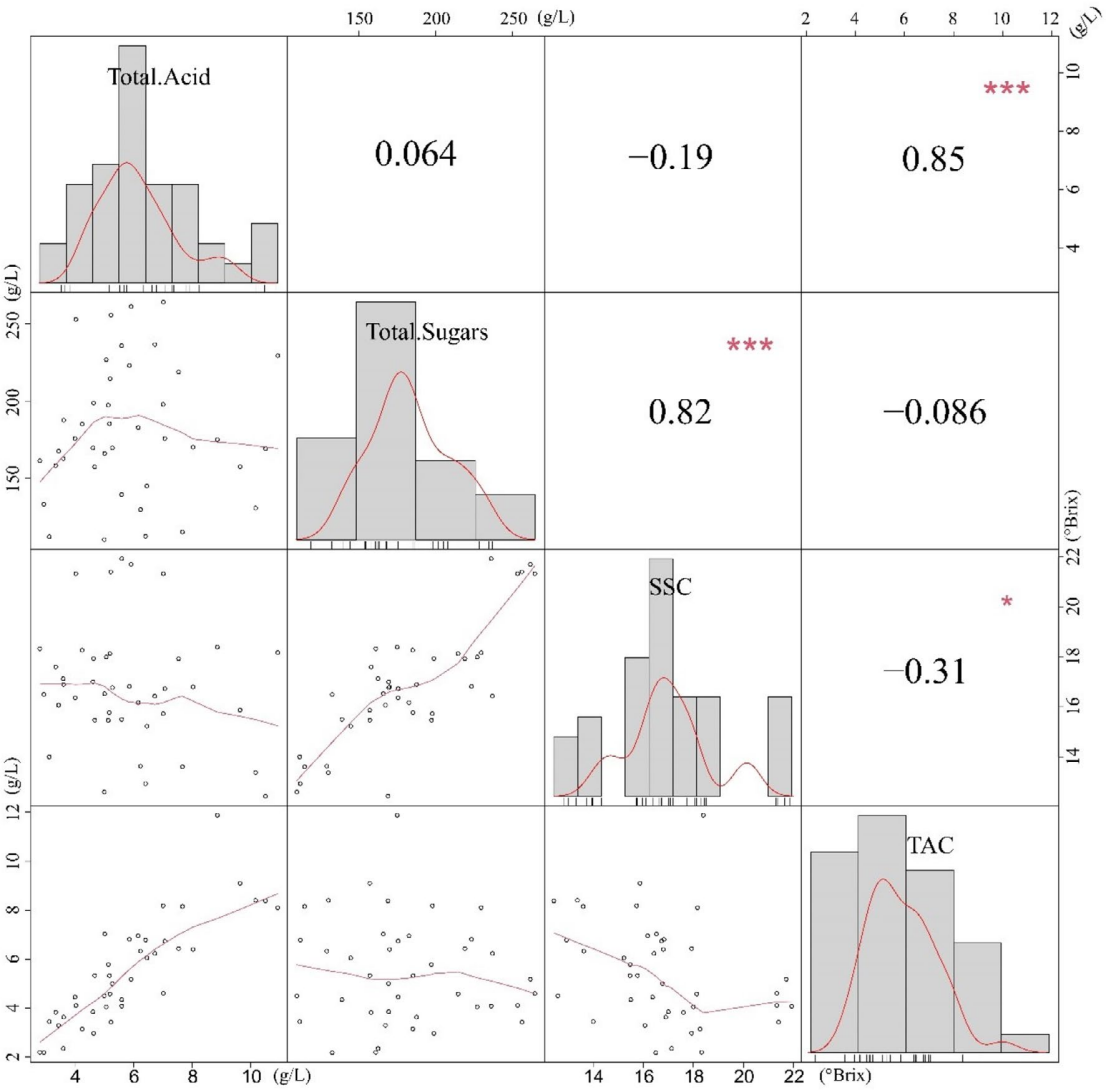
Correlations between key chemical metrics: total sugars (glucose and fructose) vs. total acids (tartaric, malic, and citric); and SSC vs. TAC.

Table 4 presents the sugar and acid contents of the 41 grape varieties. Significant differences among cultivars were observed for all quantified compounds (*p* < 0.05, Waller‐Duncan test). Correlation analysis (Figure 2) revealed highly significant positive associations between SSC and total sugar content (glucose + fructose) (*r* = 0.81, *p* < 0.001), as well as between TAC and total acid content (tartaric + malic + citric acids) (*r* = 0.79, *p* < 0.001). This mechanistic linkage provides the chemical basis for SSC and TAC serving as reliable maturity indicators, as well as for their association with perceived sweetness and sourness.

### 3.3 Relationship Between the Theoretically Calculated Sweetness/Sourness Values and the Perceived Sweetness and Sourness Across Different Varieties and Its Validation

**FIGURE 3:**
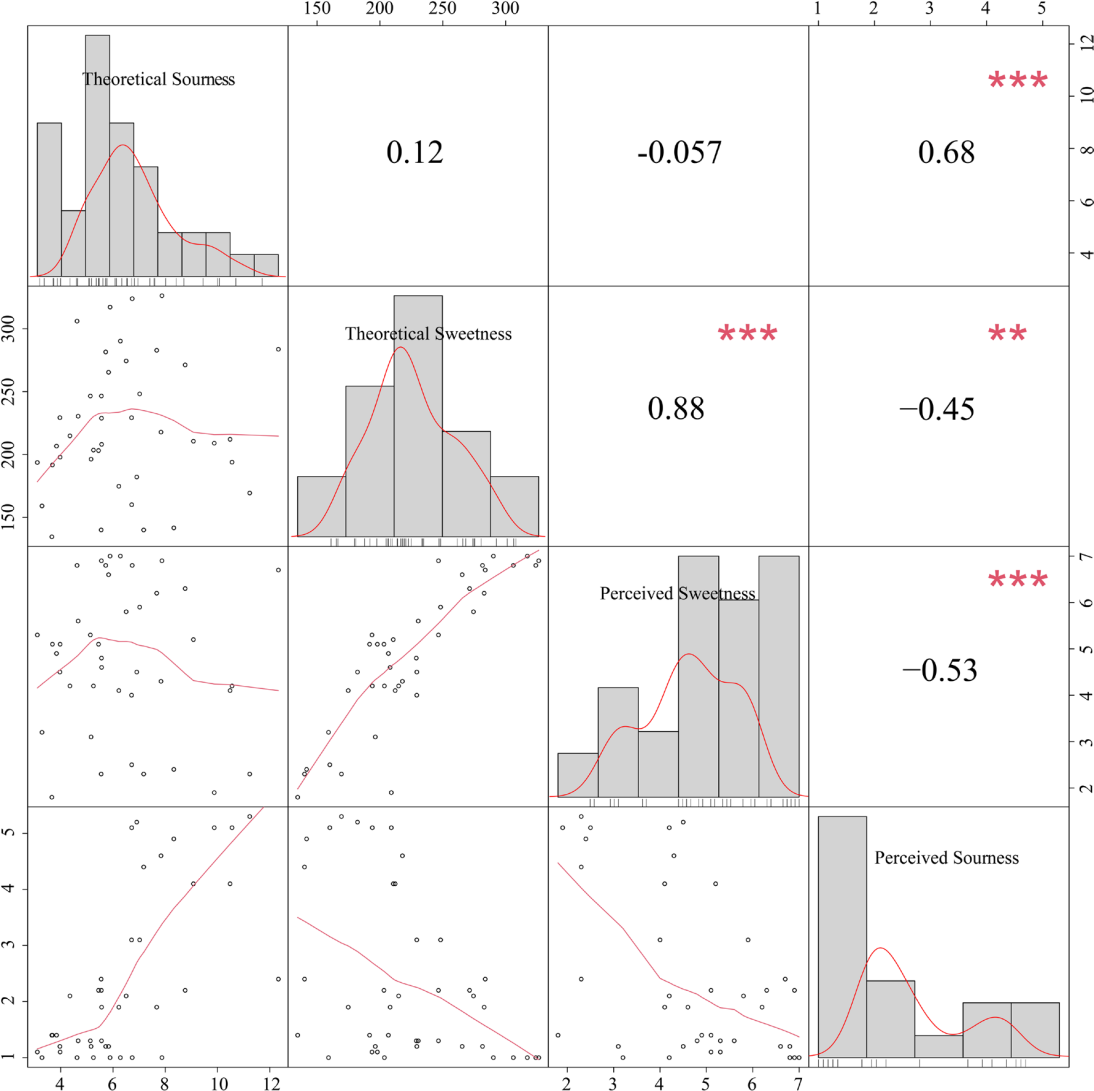
Correlations between calculated theoretical sweetness/sourness and perceived sweetness and sourness across varieties.

While our analyses established relationships between sugar/acid composition and physicochemical indices (SSC/TAC), their connections with sensory perception required further validation. We therefore computed theoretical sweetness/sourness values based on established monosaccharide sweetness equivalence coefficients and organic acid taste thresholds. These theoretical values were statistically correlated with panelists' sensory ratings (Figure 3).

Notably, theoretical sweetness correlated significantly with perceived sweetness (*r* = 0.88, *p* < 0.001), and this correlation was stronger than that between SSC and perceived sweetness (*r* = 0.74). In contrast, the correlation of perceived sourness with theoretical sourness (*r* = 0.68, *p* < 0.001) was weaker than that with TAC (*r* = 0.76). These findings confirm that computational taste models effectively capture varietal sensory profiles, particularly for sweetness. However, the model for sourness perception has certain limitations in predicting perceived sourness, possibly due to the complex interactions among acids and the suppressive effect of high sugar content.

### 3.4 PCA Dimensionality Reduction Analysis to Identify Key Indicators Affecting Fruit Quality

While computational taste models demonstrate alignment with sensory perception, their complexity necessitates simplification. We therefore applied principal component analysis (PCA) to theoretical sourness indicators from organic acids. Given the near 1:1 glucose:fructose ratio and fructose's higher sweetness potency, fructose was retained without dimensionality reduction.

**FIGURE 4:**
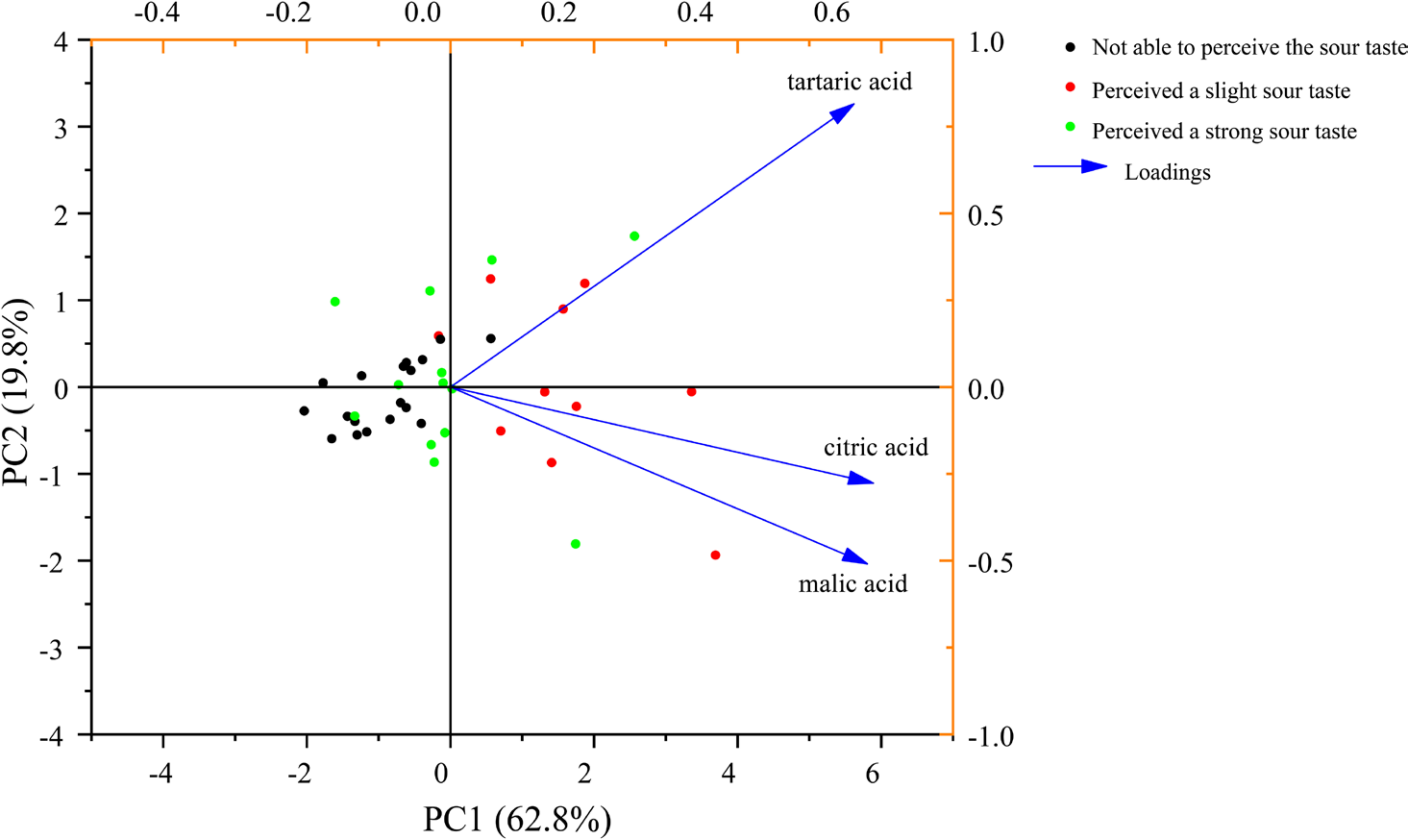
PCA of organic acids.

PCA revealed tartaric acid's theoretical sourness loaded positively on both PC1 and PC2 (cumulative variance explained: 82.6%; Figure 4). Tartaric acid loaded positively on both PC1 and PC2, indicating that it captures a substantial portion of the variation in organic acid composition. However, it is important to note that citric, tartaric, and malic acids do not covary completely; thus, tartaric acid alone may not fully represent the complexity of acid perception.

**FIGURE 5:**
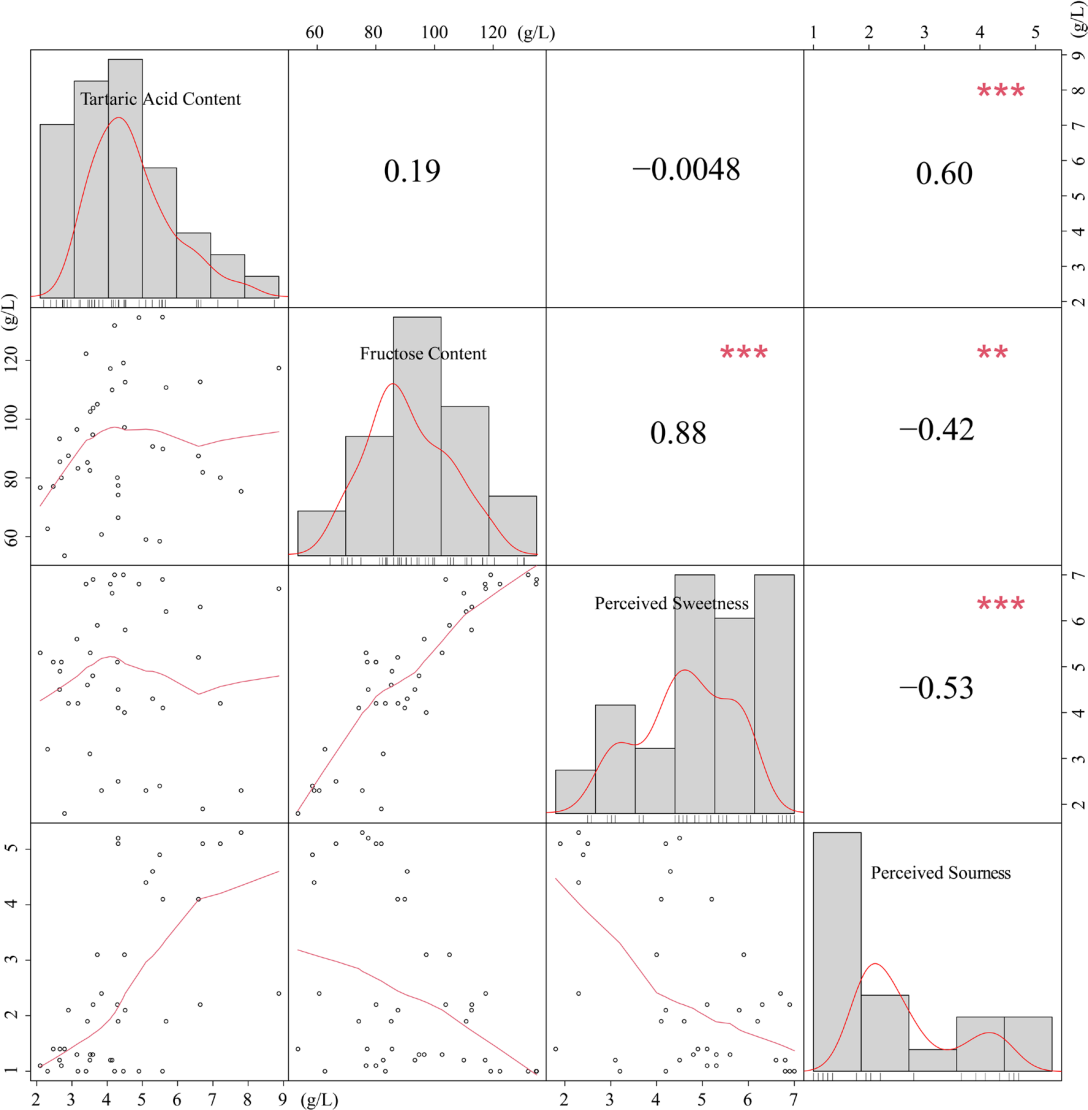
Correlations between tartaric acid/fructose content and perceived sourness/sweetness.

Subsequent correlation analysis (Figure 5) confirmed that fructose content was significantly positively correlated with perceived sweetness (*r* = 0.88, *p* < 0.001), while tartaric acid content showed a significant positive correlation with perceived sourness (*r* = 0.60, *p* < 0.001). These findings establish fructose and tartaric acid as key chemical indicators for perceived sweetness and sourness in grape cultivars. Notably, fructose enables direct inference of perceived sweetness. Contrary to expectations, however, the predictive capacity of tartaric acid for perceived sourness did not exceed that of TAC. This outcome was unexpected given the distinct chemical properties of tartaric acid, particularly its stronger acidic character (lower pKa) compared to other grape acids, which we hypothesized would correlate more strongly with sensory sourness. We attribute this result to gustatory suppression effects (Junge et al. 2020; Green et al. 2010; Qin et al. 2022) wherein extreme sugar‐acid imbalance (sugar:acid ratio > 8:1) compromises perceived sourness, while reliance solely on tartaric acid amplifies measurement error under such conditions. Additionally, the incomplete covariance among organic acids means that tartaric acid alone may not capture all variation relevant to sourness perception.

### 3.5 Quality Grading of the Different Grape Varieties

PCA identified tartaric acid and fructose as key determinants of perceived sweetness and sourness. To explore the underlying chemical drivers that structure varietal differences, we conducted k‐means clustering on 41 cultivars based on their tartaric acid and fructose concentrations. This focus on fundamental chemical metrics offers a complementary perspective to sensory profiles for understanding cultivar relationships.

**FIGURE 6:**
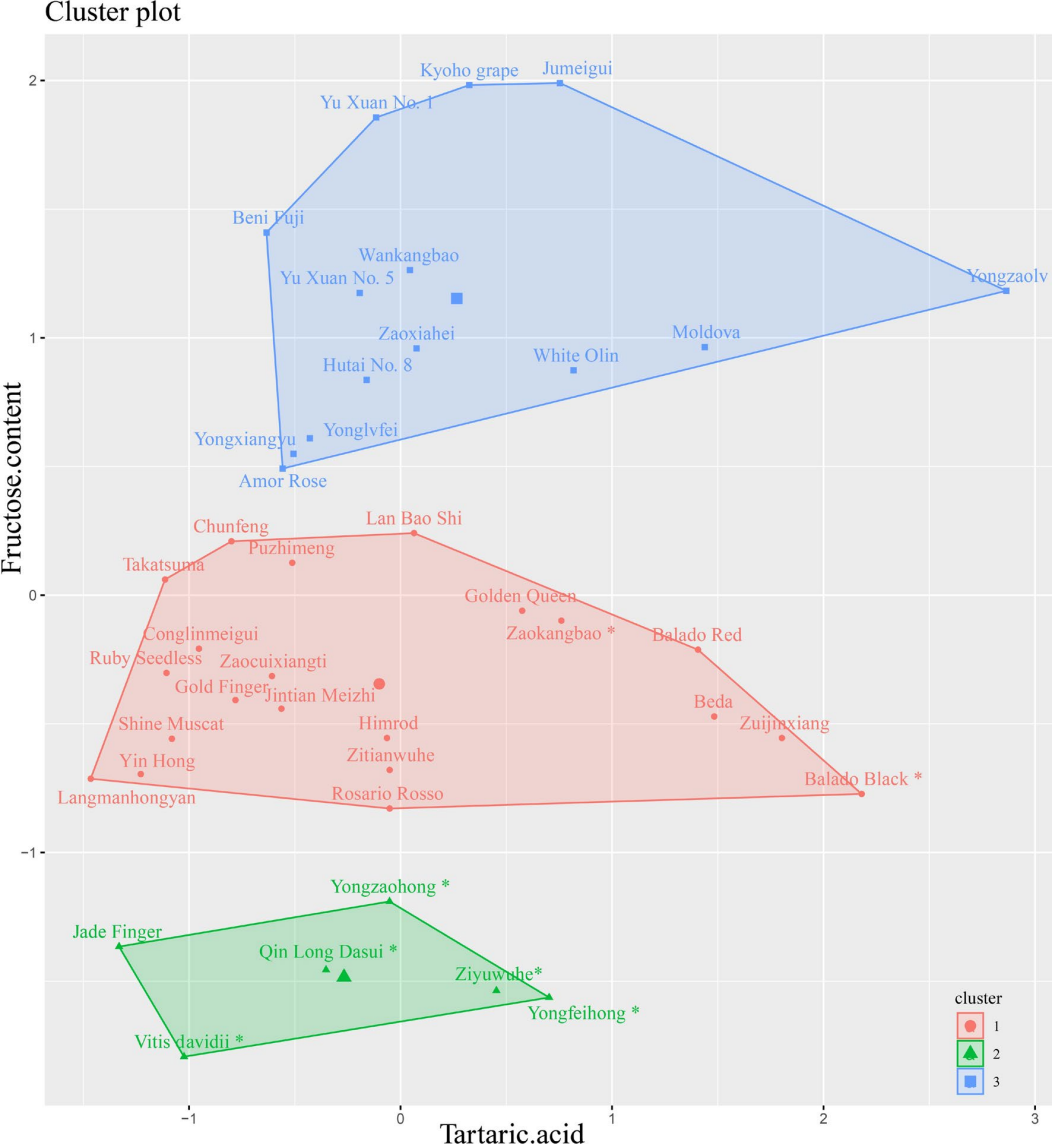
Grape quality grading of the different varieties. The abscissa represents the tartaric acid content of the different varieties, the ordinate represents the fructose content, and different colors represent different groups.

Cluster analysis delineated three distinct groups (Figure 6), there are 14 high‐sugar, 21 medium‐sugar, and 6 low‐sugar varieties. This classification result is essentially consistent with the perceived sweetness results (e.g., high‐sugar cluster included varieties with high perceived sweetness scores such as ‘Yuxuan No. 1’ and ‘Kyoho grape’). However, we were unable to classify varieties based on acid content, possibly because the proportion of organic acid content was too low relative to sugar content, resulting in clustering driven primarily by sugar levels.

The integrated clustering approach demonstrates that tartaric acid and fructose alone are sufficient to drive a robust stratification of cultivars based on sugar levels. While effectively predicting perceived sweetness, current methodology cannot resolve acidity grading in sugar‐dominant systems. Future research should develop acid‐targeted analytical protocols for unbalanced matrices.

## 4 Conclusion

This study quantified SSC, TAC, RTT, and sugar/acid profiles across 41 grape cultivars, correlating these parameters with sensory perception. Juice analysis detected only glucose, fructose, tartaric acid, malic acid, and citric acid. Sucrose absence may reflect either: (i) high invertase activity cleaving sucrose to monosaccharides, or (ii) phloem‐localized sucrose inaccessible to manual extraction.

SSC and TAC demonstrated highly significant positive correlations with total soluble sugars and total acids, respectively, validating their utility as maturity indicators. Conversely, RTT proved unreliable for multi‐cultivar sensory quality assessment, showing only weak correlation with perceived sweetness.

Theoretical sweetness and sourness values, calculated via monosaccharide and organic acid taste intensity models, showed strong concordance with sensory ratings. Notably, theoretical sweetness outperformed SSC in predicting perceived sweetness. In contrast, theoretical sourness slightly underperformed TAC in predicting perceived sourness.

Principal component analysis identified fructose and tartaric acid as dominant taste determinants. Their contents showed significant positive correlations with sensory perception. Cluster analysis based on these analytes classified juices into three groups: high‐sugar (14 cultivars), medium‐sugar (21 cultivars), and low‐sugar (6 cultivars). This classification aligned with perceived sweetness but failed to stratify perceived sourness. We attribute this to sugar‐acid imbalance (sugar ≫ acid) causing gustatory suppression of sourness perception.

## Author Contributions

**Qianqian Zheng:** investigation. **Zhongyi Yang:** writing – review and editing, methodology. **Liufei Huang:** data curation, visualization, formal analysis. **Lingling Hu:** project administration, resources, validation. **Yingjian Cai:** writing – original draft, software, data curation. **Yueyan Wu:** writing – review and editing, methodology, funding acquisition, conceptualization. **Yi Qin:** supervision.

## Funding

This work was supported by Public Welfare Research Program Key Project of Ningbo (2024S016), Zhejiang Provincial Top Discipline of Biological Engineering (Level A) Open Fund (KF2024001) and Zhejiang Provincial Top Discipline of Biological Engineering (Level A) Self‐Set Subject (ZS2023016).

## Ethics Statement

Ethics approval for this study was obtained from Zhejiang Wanli University Experimental Animal Ethics Committee (approval number: 20221129001). All participants were provided with information regarding the study and gave their written informed consent prior to participation. The data obtained from the evaluation were only used for scientific research, not for other purposes. This study was conducted in compliance with the Declaration of Helsinki and all applicable ethical guidelines.

## Conflicts of Interest

The authors declare no conflicts of interest.

## Supporting information


**Figure S1:** The raw data for sugar and acid contents in different grape varieties, as determined by liquid chromatography.

**Table S1:** The results of sugar acid content for each variety obtained through LC–MS analysis.

**Table S2:** The soluble solids content (SSC) and titratable acidity (TAC) values of different grape varieties, the theoretical sweetness and acid from the sugar and acid contents of the fruits, and the raw scores provided by evaluators using a 7‐point scale.

## Data Availability

The authors confirm that the data supporting the findings of this study are available within the article or its Supporting Information.
