# Supplementary material for: Identification of Key Determinants for Perceived Sweetness and Sourness in Fresh Grapes
Source: Food Sci Nutr. 2026 May 5;14(5):e71824. doi: 10.1002/fsn3.71824 (PMC13144547; doi:10.1002/fsn3.71824)
Supplement: Supplementary file 1 — Figure S1: The raw data for sugar and acid contents in different grape varieties, as determined by liquid chromatography. [file FSN3-14-e71824-s001.pdf]

project_wangzhonghua_BeiMu
Dataset: Untitled
Last Altered: Friday, October 11, 2024 15:42:35 China Standard Time
Printed: Friday, October 11, 2024 15:43:34 China Standard Time

Method: F:\data\Wu_yueyan.PRO\MethDB\20241011_ organic acid .mdb 11 Oct 2024 13:22:01
Calibration: F:\data\zhanghuien.PRO\CurveDB\20241011_organic acid003.cdb 11 Oct 2024 15:39:56

Name: 202401001_youjisuan_sample001, Date: 01-Oct-2024, Time: 18:16:45, ID: , Description:

jiushisuan

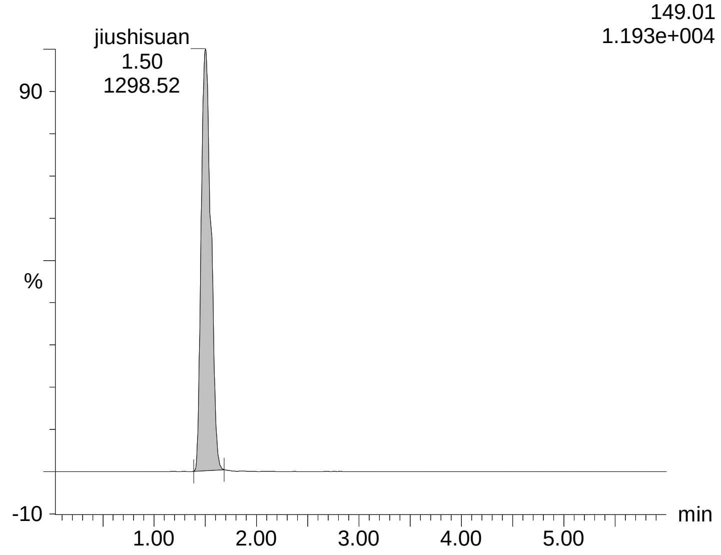

ningmensuan

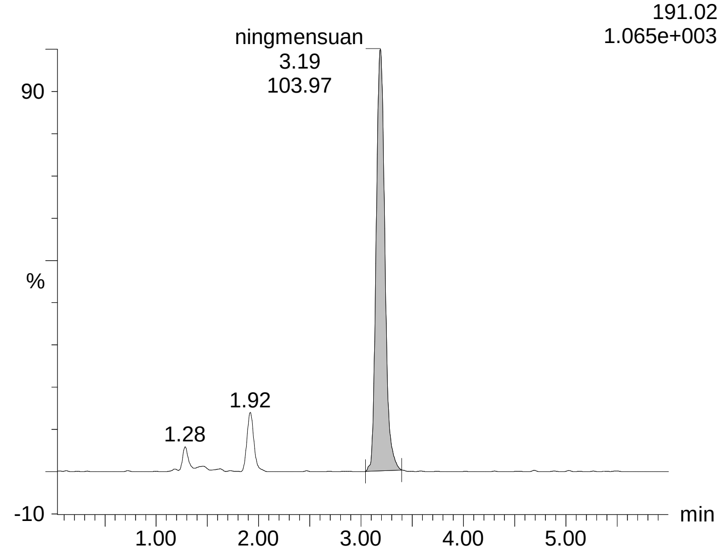

pinguosuan

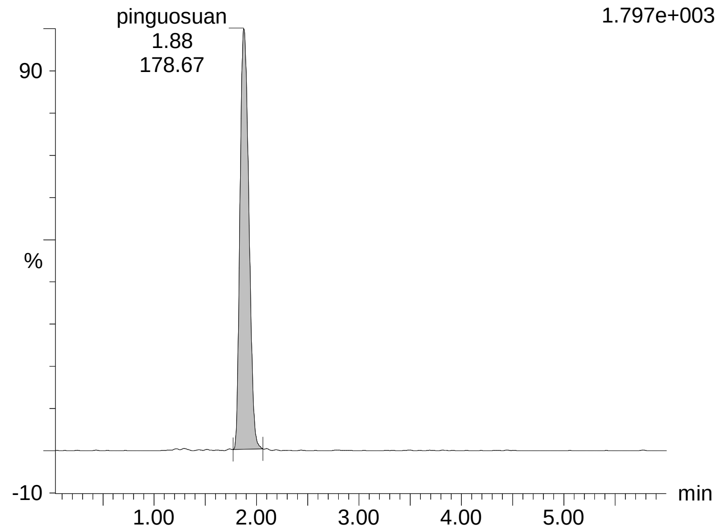

	#	Name	Sample Text	RT	Area	Std. Conc	Conc.
1	1	jiushisuan		1.50	1298.524		210.560354
2	2	ningmensuan		3.19	103.968		14.039831
3	3	pinguosuan		1.88	178.674		57.440286

Name: 202401001_youjisuan_sample002, Date: 01-Oct-2024, Time: 18:23:43, ID: , Description:

jiushisuan

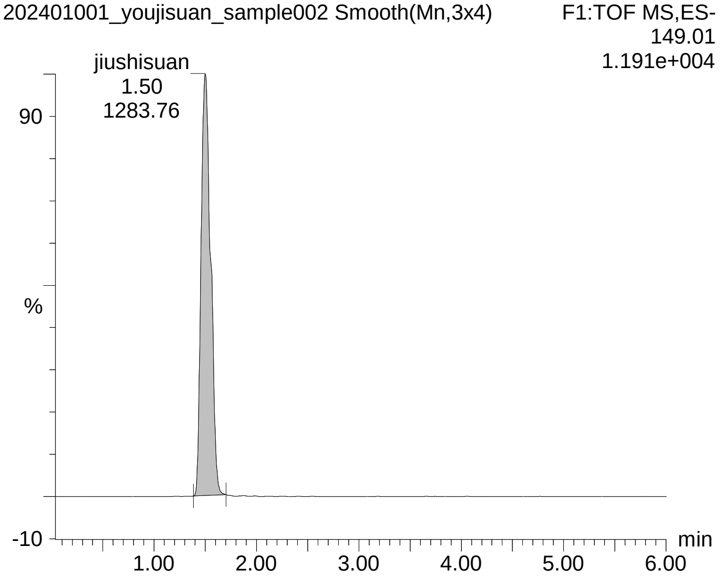

ningmensuan

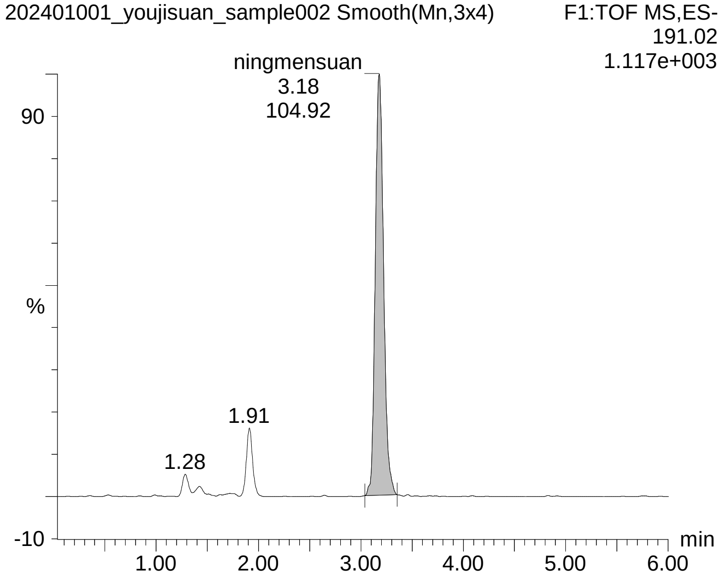

pinguosuan

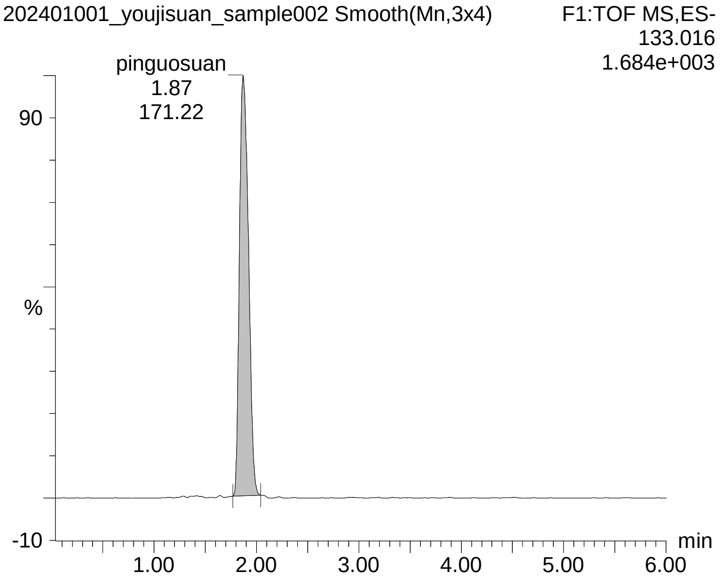

	#	Name	Sample Text	RT	Area	Std. Conc	Conc.
1	1	jiushisuan		1.50	1283.755		207.015826
2	2	ningmensuan		3.18	104.918		14.178285
3	3	pinguosuan		1.87	171.224		54.173721

Name: 202401001_youjisuan_sample003, Date: 01-Oct-2024, Time: 18:30:43, ID: , Description:

jiushisuan

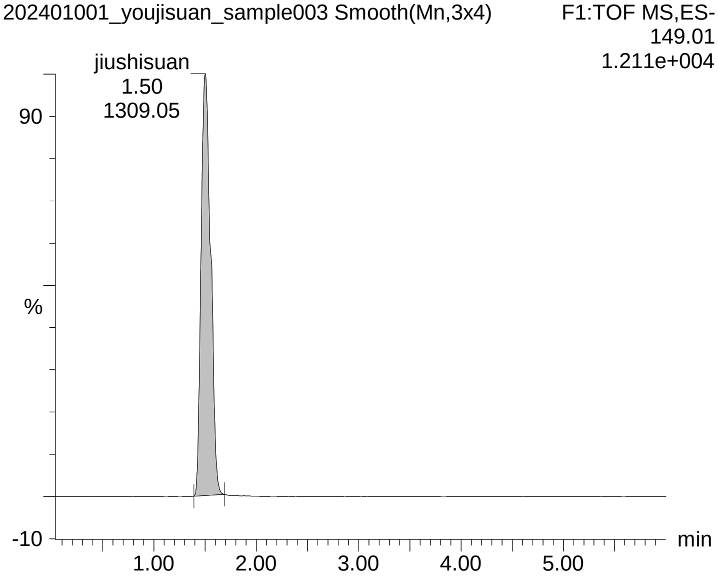

ningmensuan

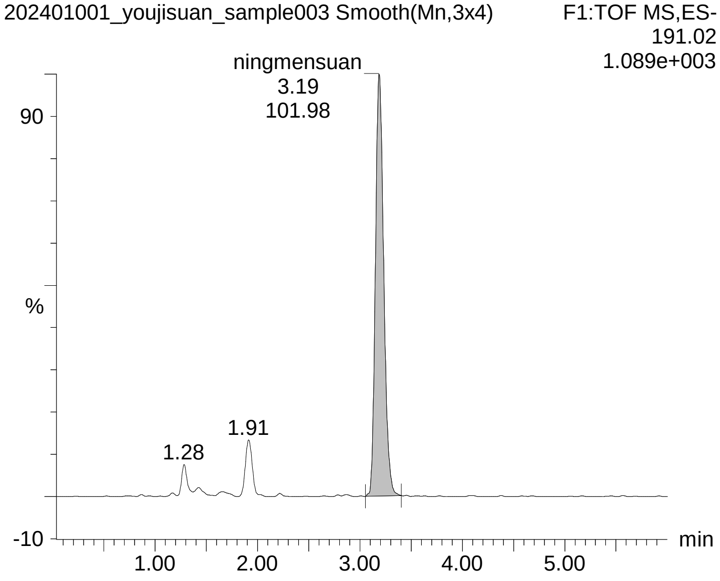

pinguosuan

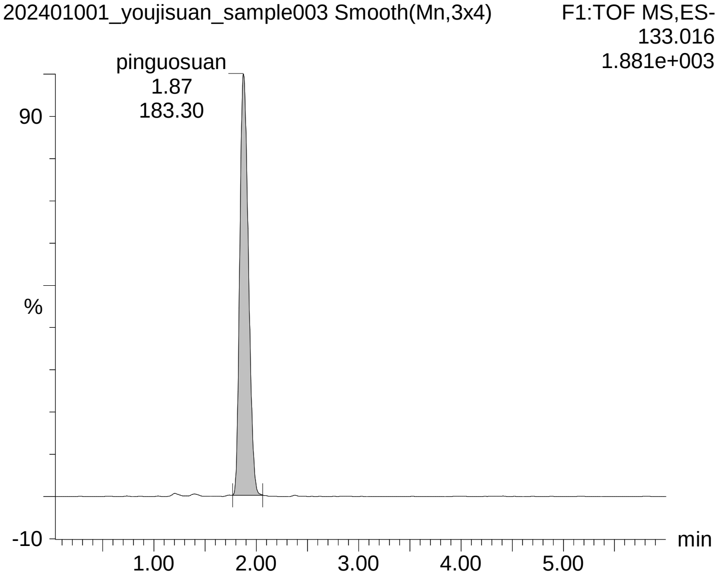

	#	Name	Sample Text	RT	Area	Std. Conc	Conc.
1	1	jiushisuan		1.50	1309.045		213.111528
2	2	ningmensuan		3.19	101.984		13.750682
3	3	pinguosuan		1.87	183.301		59.507890

Name: 202401001_youjisuan_sample004, Date: 01-Oct-2024, Time: 18:37:43, ID: , Description:

jiushisuan

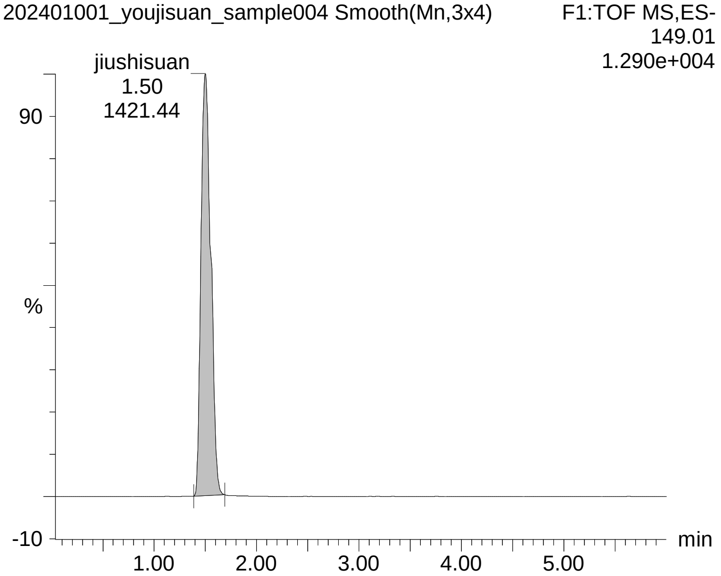

ningmensuan

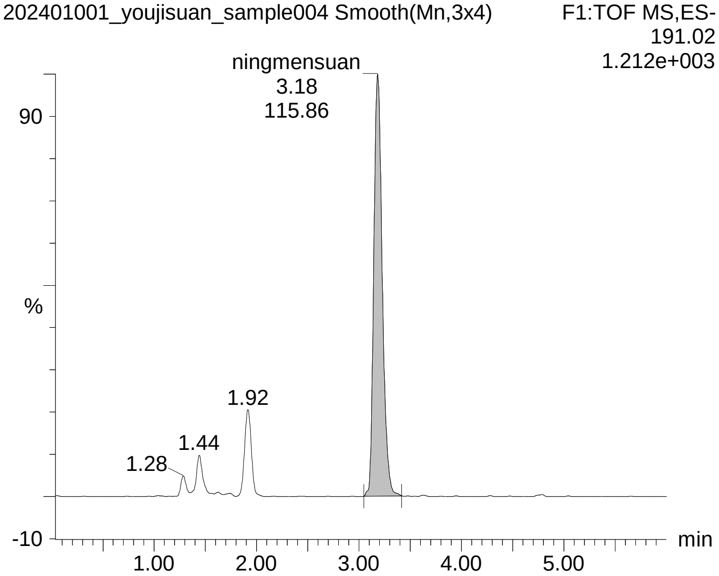

pinguosuan

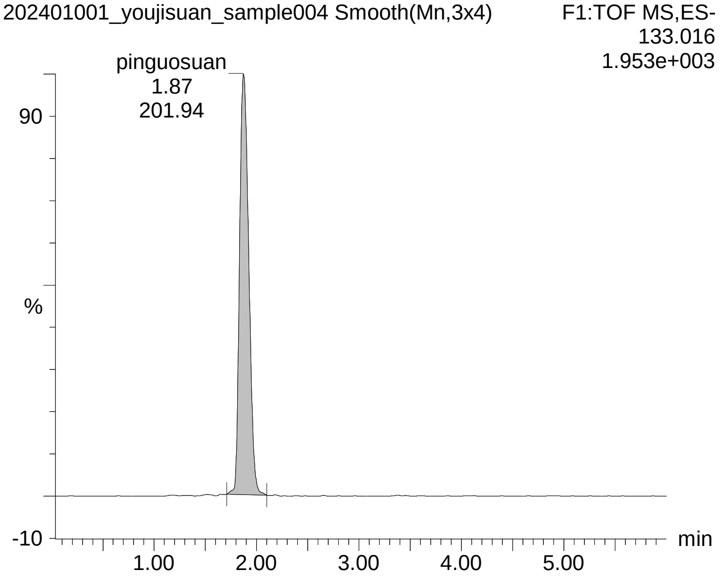

	#	Name	Sample Text	RT	Area	Std. Conc	Conc.
1	1	jiushisuan		1.50	1421.443		241.857473
2	2	ningmensuan		3.18	115.865		15.773707
3	3	pinguosuan		1.87	201.942		68.165124

Name: 202401001_youjisuan_sample005, Date: 01-Oct-2024, Time: 18:44:43, ID: , Description:

jiushisuan

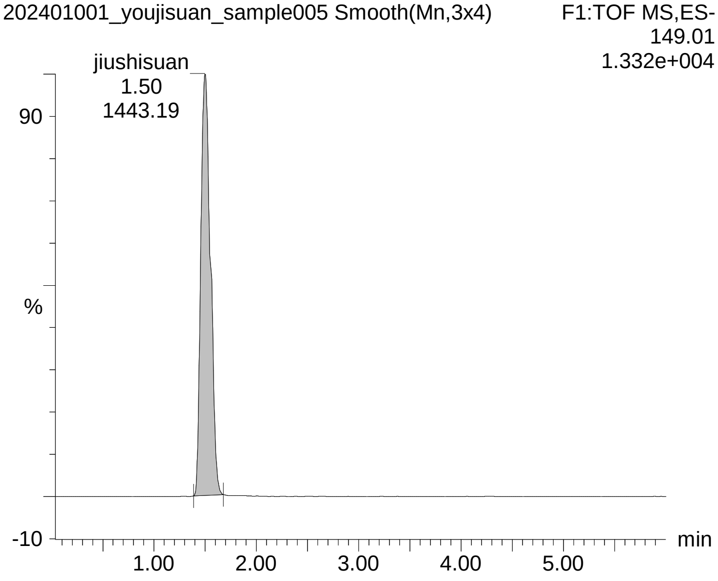

ningmensuan

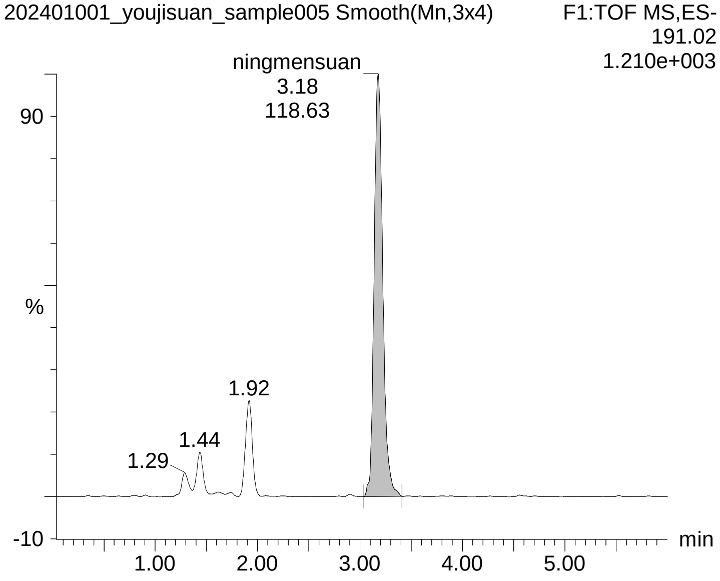

pinguosuan

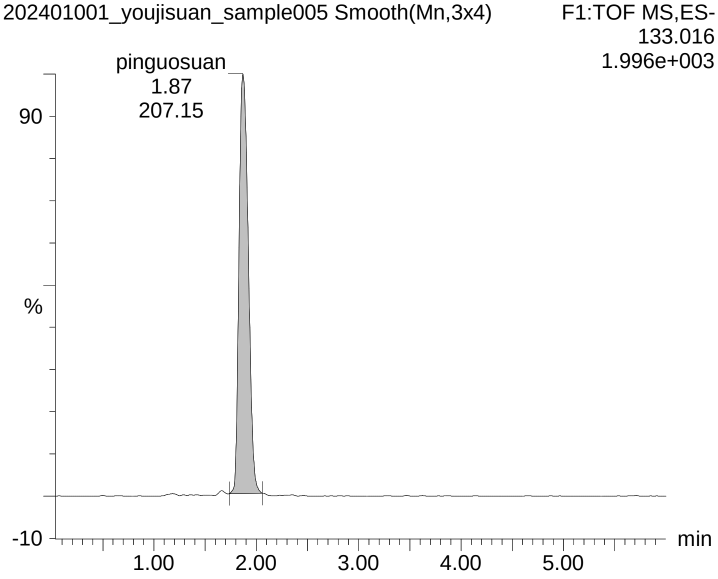

	#	Name	Sample Text	RT	Area	Std. Conc	Conc.
1	1	jiushisuan		1.50	1443.186		247.766651
2	2	ningmensuan		3.18	118.625		16.175952
3	3	pinguosuan		1.87	207.146		70.683388

project_wangzhonghua_BeiMu
Dataset: Untitled
Last Altered: Friday, October 11, 2024 15:42:35 China Standard Time
Printed: Friday, October 11, 2024 15:43:34 China Standard Time

Name: 202401001_youjisuan_sample006, Date: 01-Oct-2024, Time: 18:51:43, ID: , Description:

jiushisuan

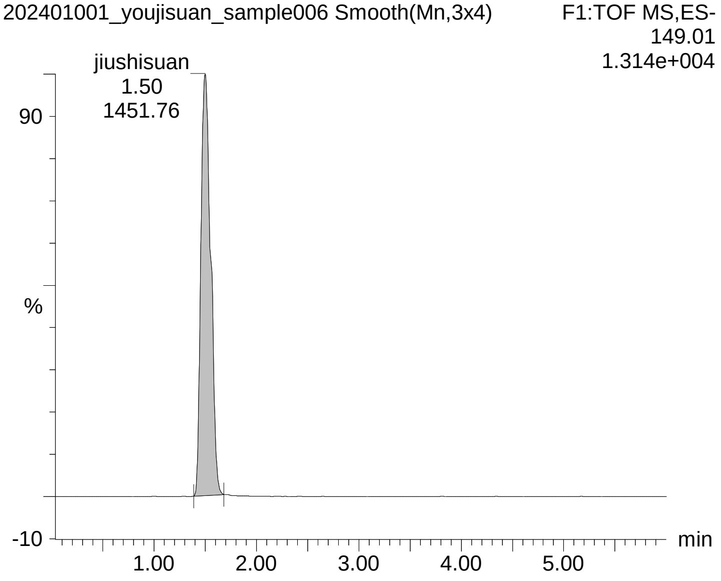

ningmensuan

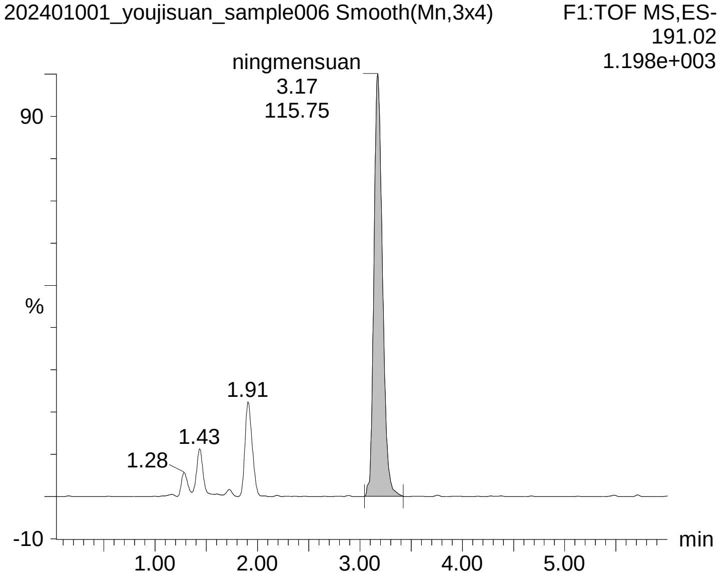

pinguosuan

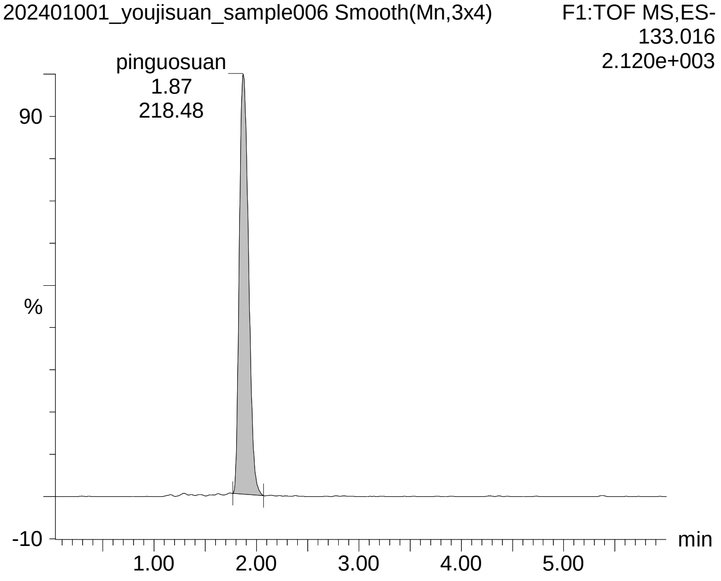

	#	Name	Sample Text	RT	Area	Std. Conc	Conc.
1	1	jiushisuan		1.50	1451.759		250.130956
2	2	ningmensuan		3.17	115.754		15.757530
3	3	pinguosuan		1.87	218.476		76.335806

Name: 202401001_youjisuan_sample007, Date: 01-Oct-2024, Time: 18:58:44, ID: , Description:

jiushisuan

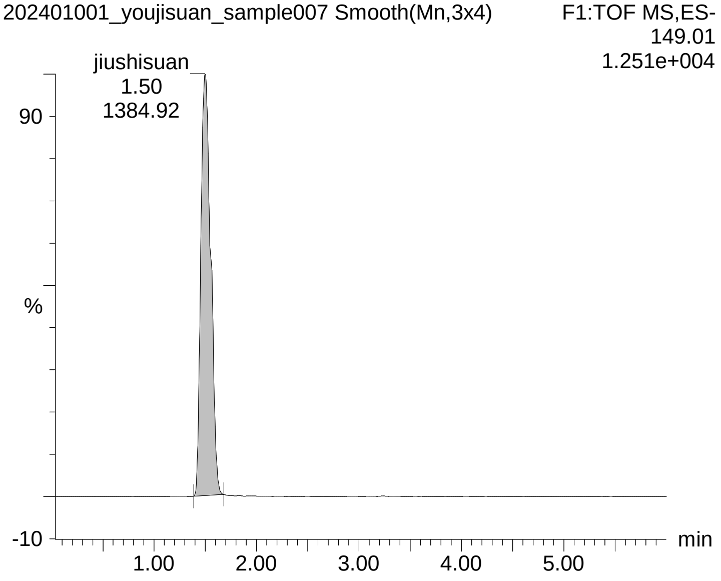

ningmensuan

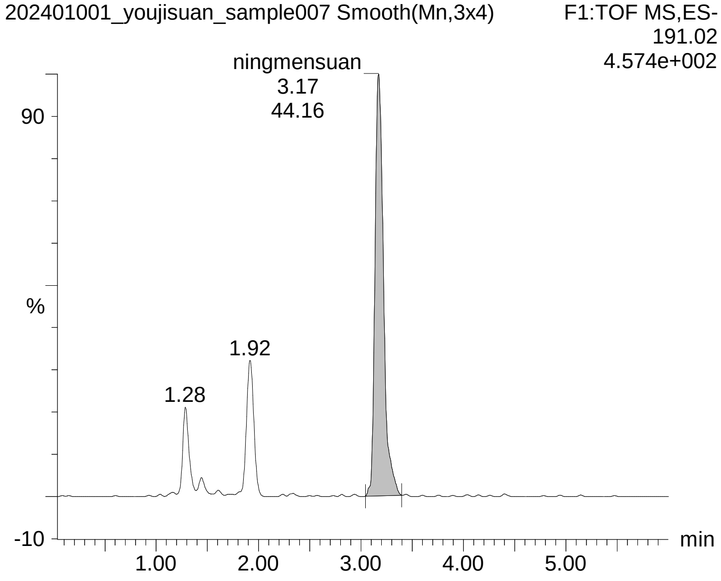

pinguosuan

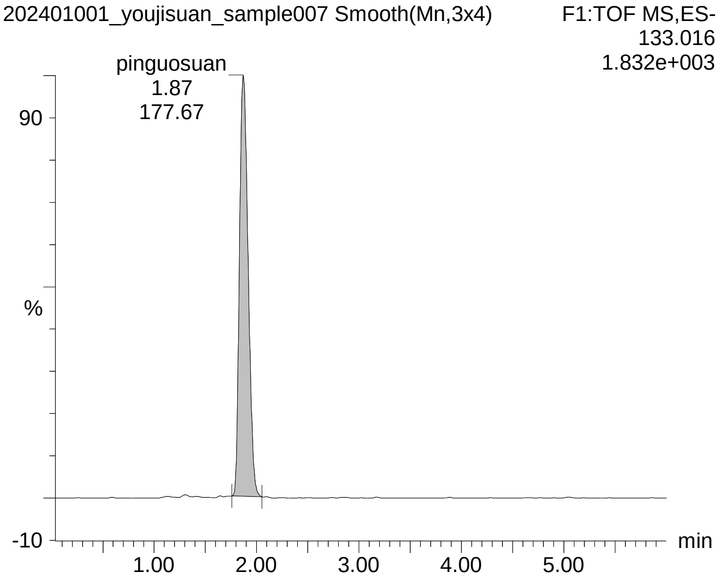

	#	Name	Sample Text	RT	Area	Std. Conc	Conc.
1	1	jiushisuan		1.50	1384.917		232.198638
2	2	ningmensuan		3.17	44.161		5.323523
3	3	pinguosuan		1.87	177.671		56.996078

project_wangzhonghua_BeiMu
Dataset: Untitled
Last Altered: Friday, October 11, 2024 15:42:35 China Standard Time
Printed: Friday, October 11, 2024 15:43:34 China Standard Time

Name: 202401001_youjisuan_sample008, Date: 01-Oct-2024, Time: 19:05:44, ID: , Description:

jiushisuan

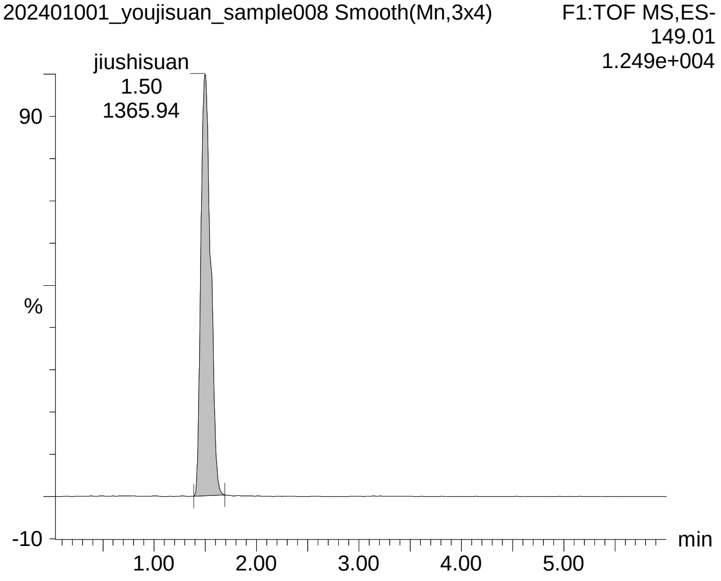

ningmensuan

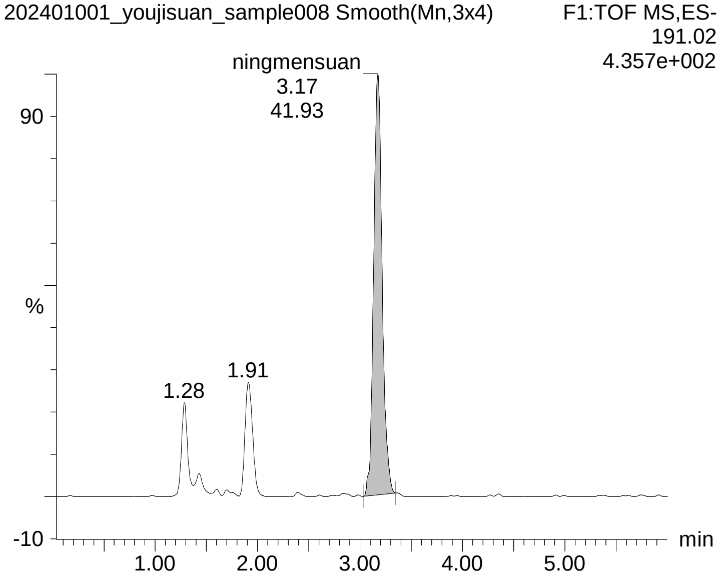

pinguosuan

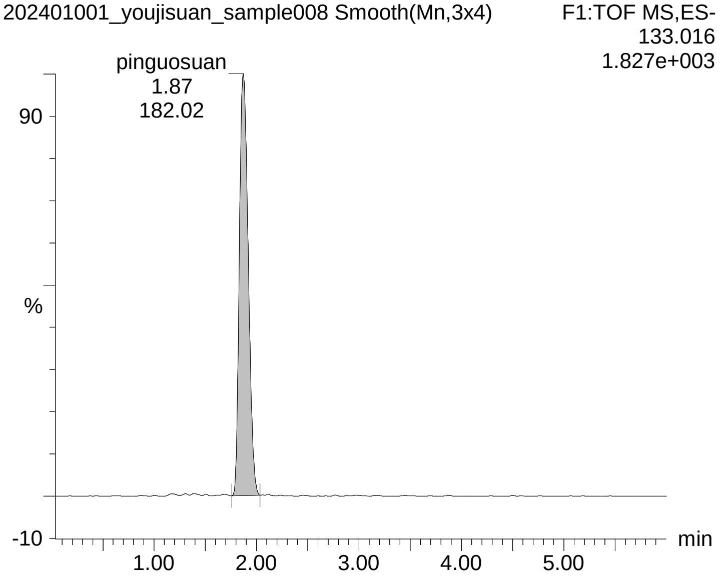

	#	Name	Sample Text	RT	Area	Std. Conc	Conc.
1	1	jiushisuan		1.50	1365.936		227.304755
2	2	ningmensuan		3.17	41.927		4.997938
3	3	pinguosuan		1.87	182.022		58.933301

project_wangzhonghua_BeiMu
Dataset: Untitled
Last Altered: Friday, October 11, 2024 15:42:35 China Standard Time
Printed: Friday, October 11, 2024 15:43:34 China Standard Time

Name: 202401001_youjisuan_sample009, Date: 01-Oct-2024, Time: 19:12:44, ID: , Description:

jiushisuan

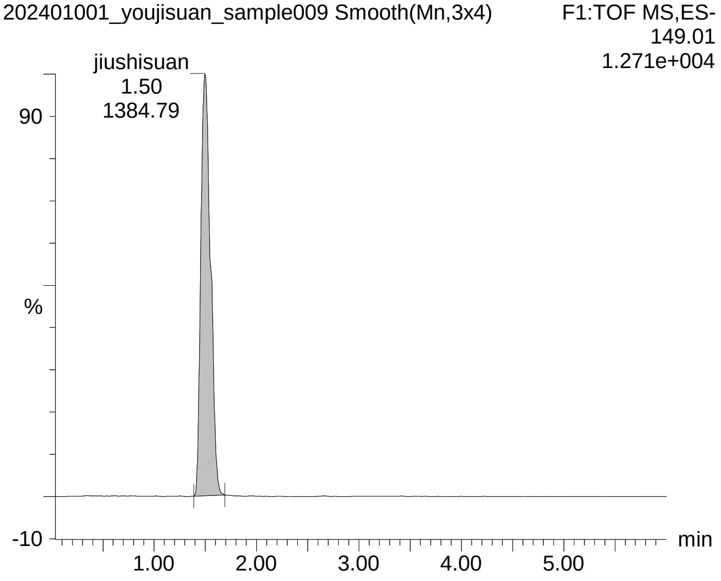

ningmensuan

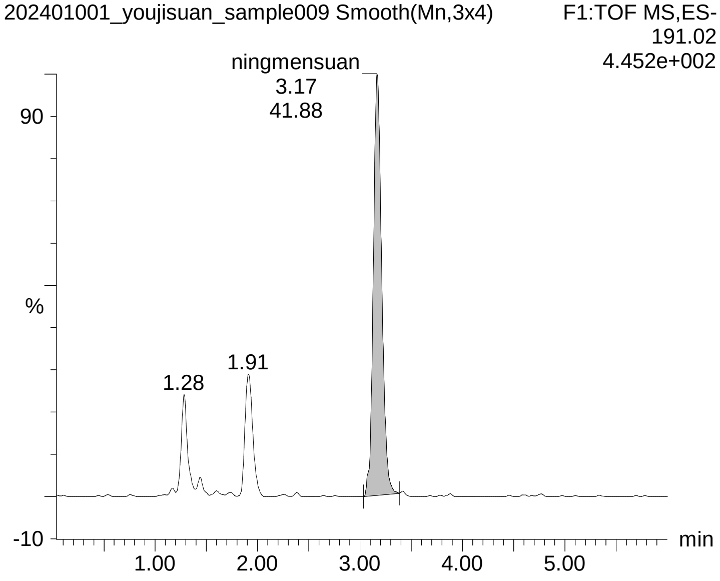

pinguosuan

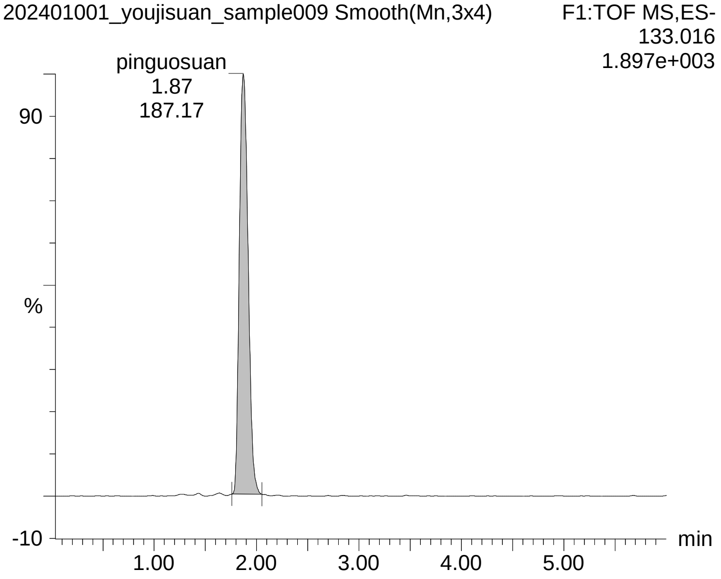

	#	Name	Sample Text	RT	Area	Std. Conc	Conc.
1	1	jiushisuan		1.50	1384.789		232.165356
2	2	ningmensuan		3.17	41.884		4.991671
3	3	pinguosuan		1.87	187.166		61.258767

Name: 202401001_youjisuan_sample010, Date: 01-Oct-2024, Time: 19:19:44, ID: , Description:

jiushisuan

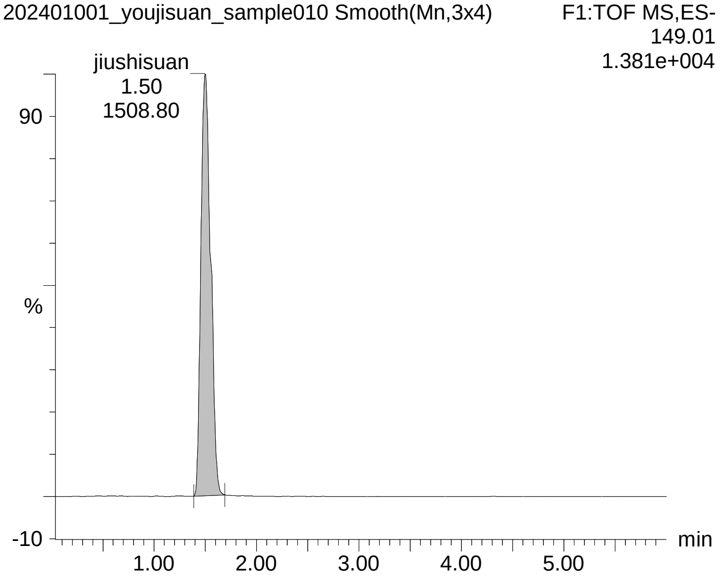

ningmensuan

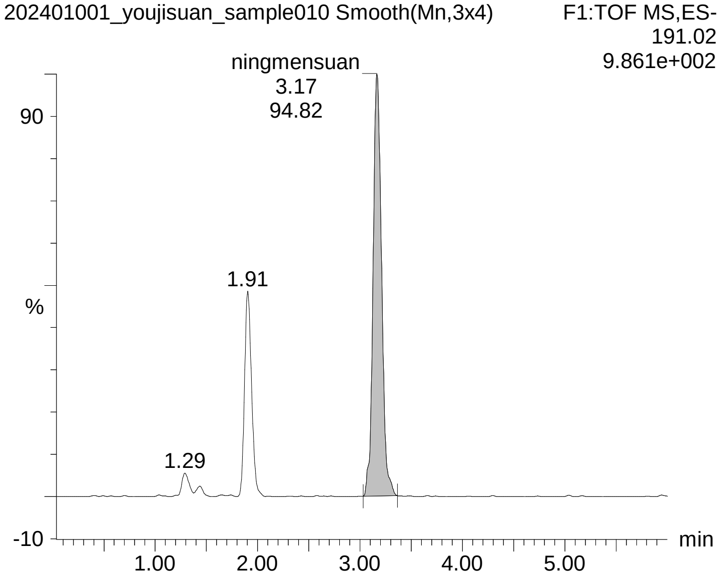

pinguosuan

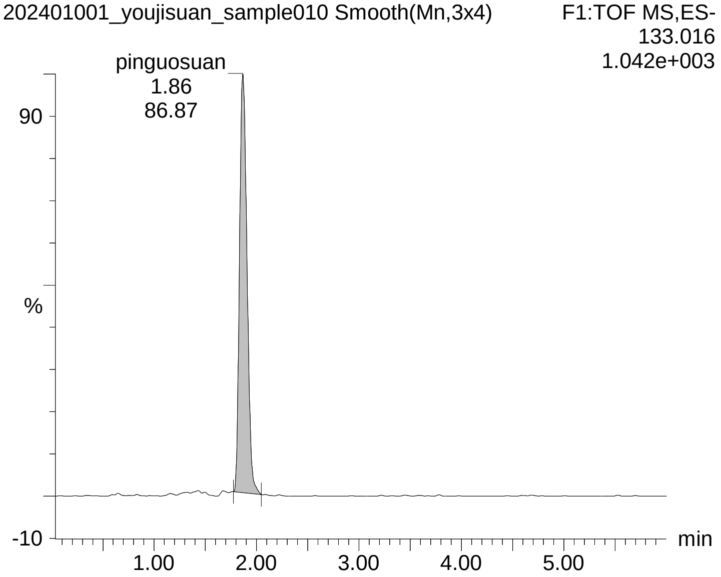

	#	Name	Sample Text	RT	Area	Std. Conc	Conc.
1	1	jiushisuan		1.50	1508.798		266.391604
2	2	ningmensuan		3.17	94.818		12.706305
3	3	pinguosuan		1.86	86.868		21.510697

project_wangzhonghua_BeiMu
Dataset: Untitled
Last Altered: Friday, October 11, 2024 15:42:35 China Standard Time
Printed: Friday, October 11, 2024 15:43:34 China Standard Time

Name: 202401001_youjisuan_sample011, Date: 01-Oct-2024, Time: 19:26:45, ID: , Description:

jiushisuan

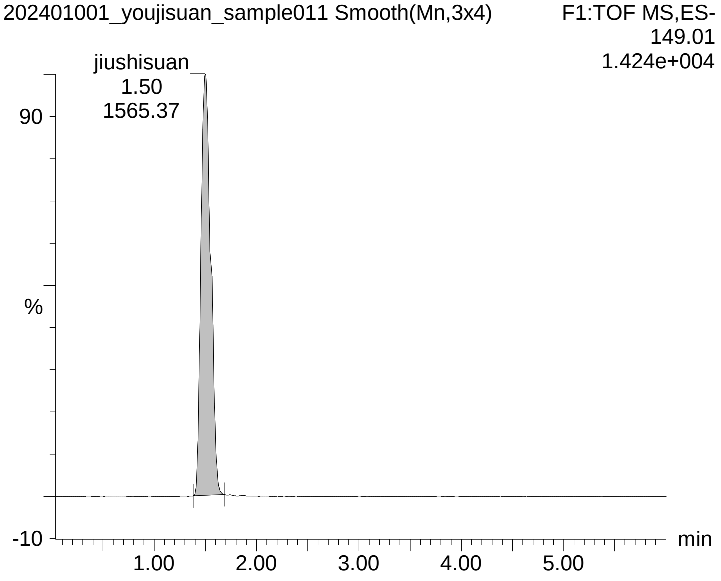

ningmensuan

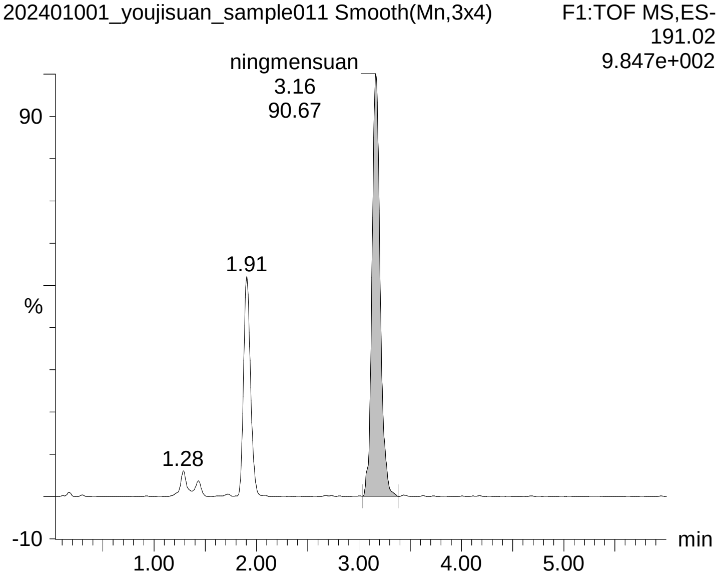

pinguosuan

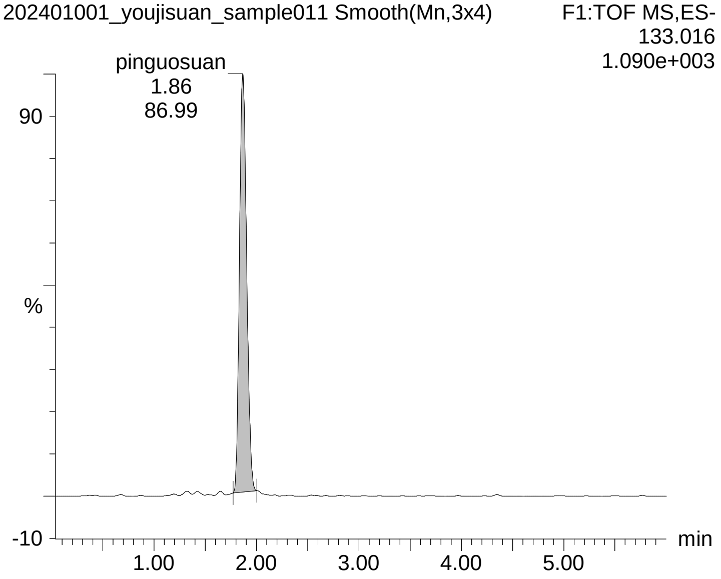

	#	Name	Sample Text	RT	Area	Std. Conc	Conc.
1	1	jiushisuan		1.50	1565.369		283.525329
2	2	ningmensuan		3.16	90.674		12.102356
3	3	pinguosuan		1.86	86.990		21.553231

project_wangzhonghua_BeiMu
Dataset: Untitled
Last Altered: Friday, October 11, 2024 15:42:35 China Standard Time
Printed: Friday, October 11, 2024 15:43:34 China Standard Time

Name: 202401001_youjisuan_sample012, Date: 01-Oct-2024, Time: 19:33:45, ID: , Description:

jiushisuan

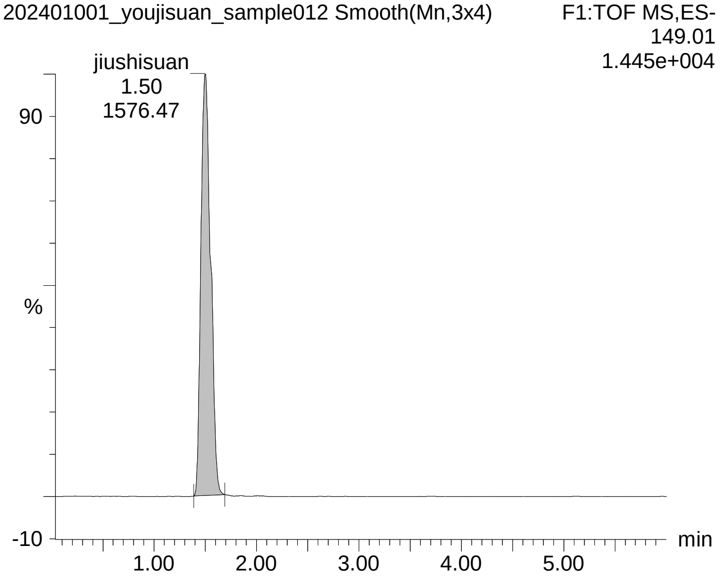

ningmensuan

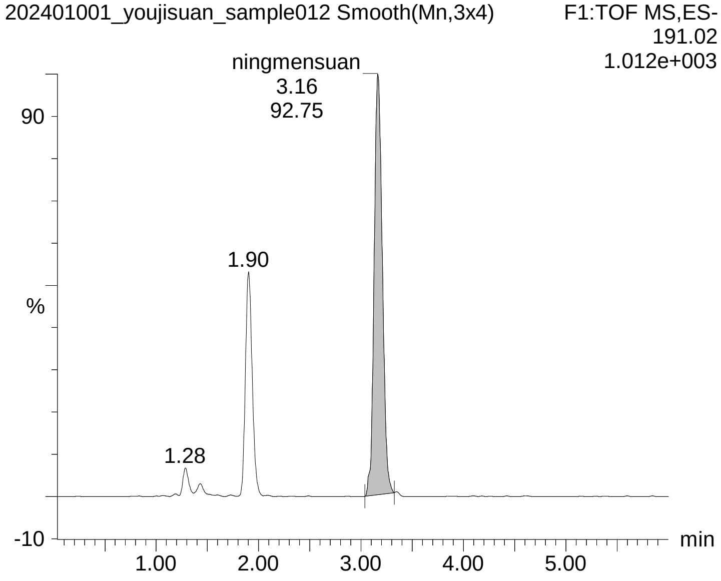

pinguosuan

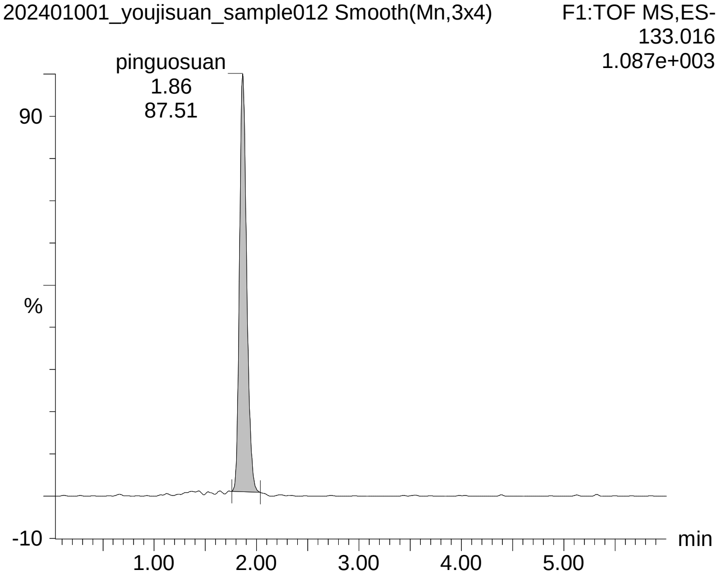

	#	Name	Sample Text	RT	Area	Std. Conc	Conc.
1	1	jiushisuan		1.50	1576.472		287.018797
2	2	ningmensuan		3.16	92.752		12.405205
3	3	pinguosuan		1.86	87.509		21.734304

project_wangzhonghua_BeiMu
Dataset: Untitled
Last Altered: Friday, October 11, 2024 15:42:35 China Standard Time
Printed: Friday, October 11, 2024 15:43:34 China Standard Time

Name: 202401001_youjisuan_sample013, Date: 01-Oct-2024, Time: 19:40:46, ID: , Description:

jiushisuan

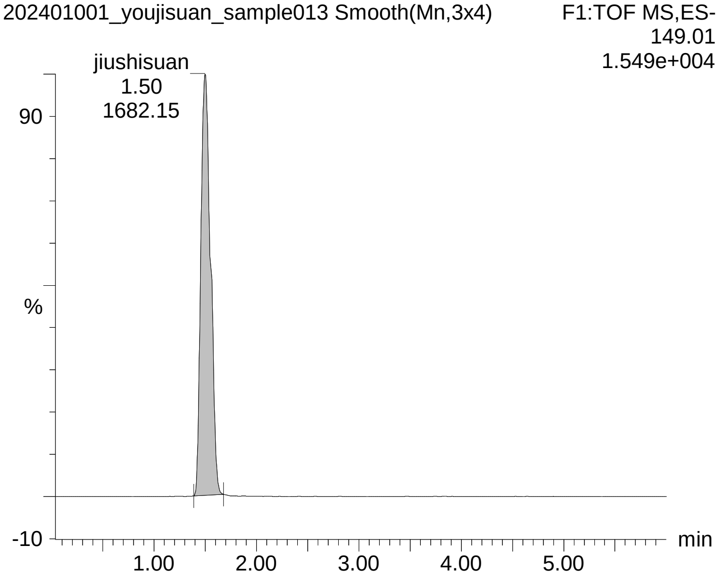

ningmensuan

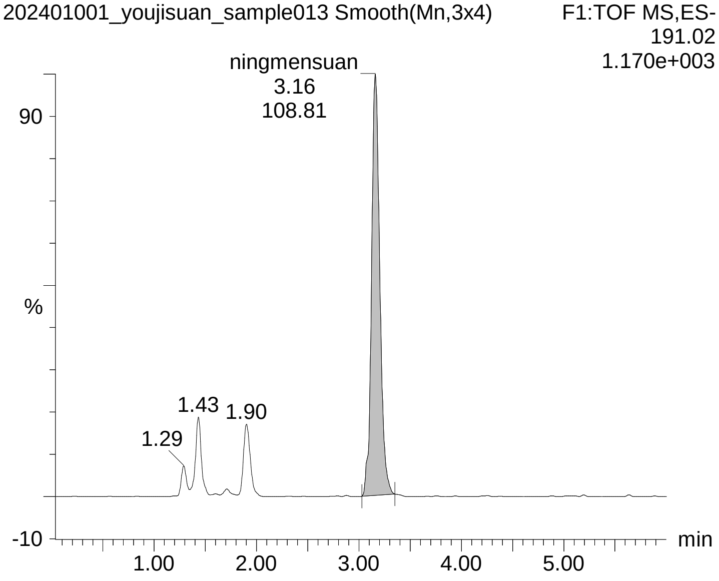

pinguosuan

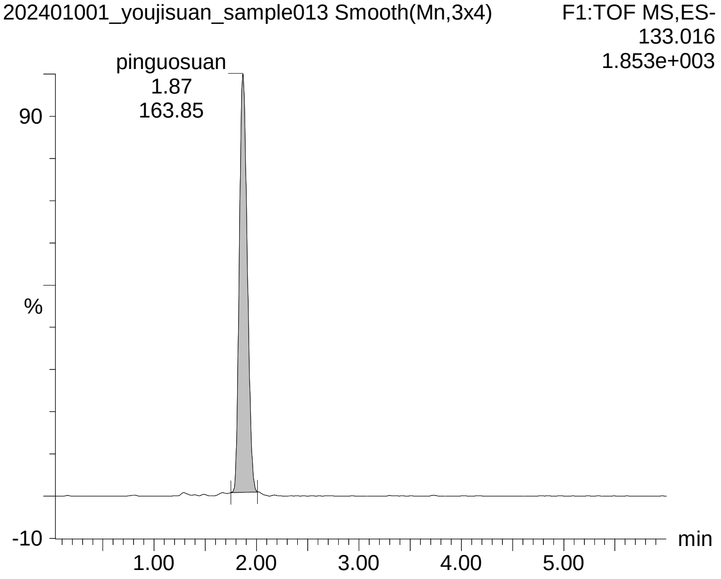

	#	Name	Sample Text	RT	Area	Std. Conc	Conc.
1	1	jiushisuan		1.50	1682.150		322.792966
2	2	ningmensuan		3.16	108.814		14.746090
3	3	pinguosuan		1.87	163.853		51.014021

project_wangzhonghua_BeiMu
Dataset: Untitled
Last Altered: Friday, October 11, 2024 15:42:35 China Standard Time
Printed: Friday, October 11, 2024 15:43:34 China Standard Time

Name: 202401001_youjisuan_sample014, Date: 01-Oct-2024, Time: 19:48:42, ID: , Description:

jiushisuan

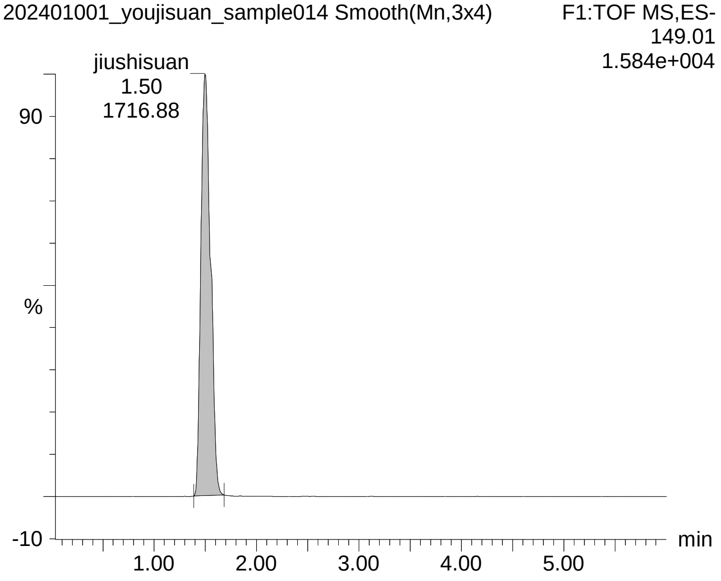

ningmensuan

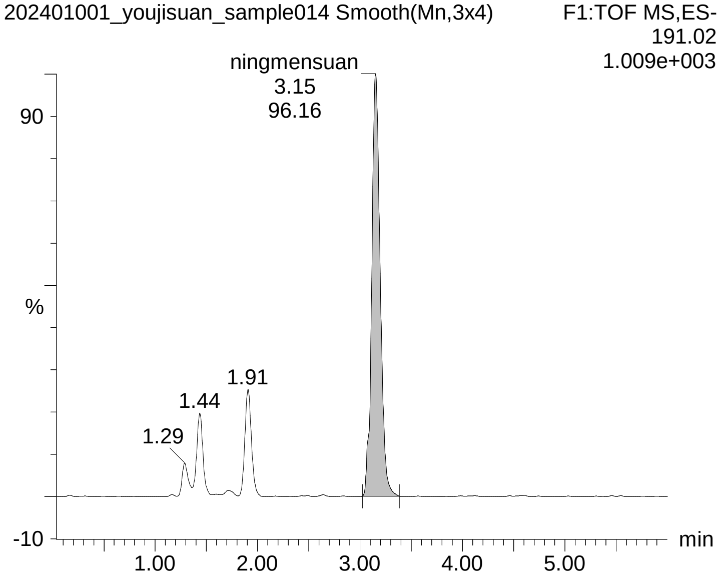

pinguosuan

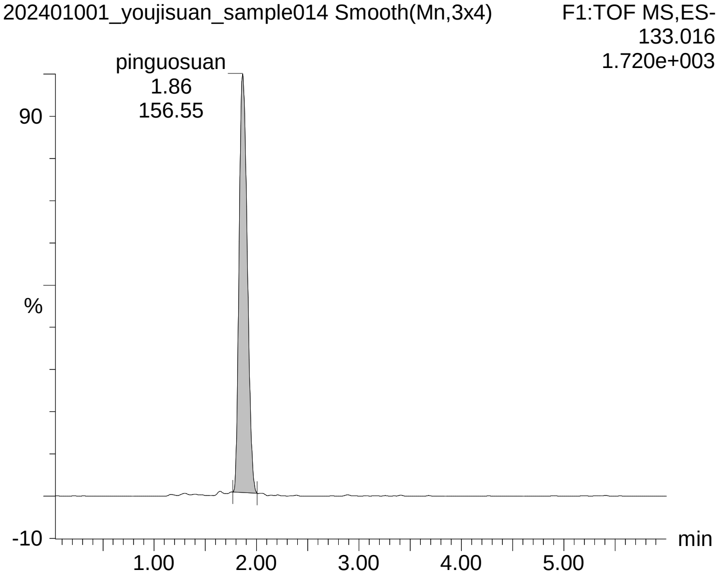

	#	Name	Sample Text	RT	Area	Std. Conc	Conc.
1	1	jiushisuan		1.50	1716.875		335.708526
2	2	ningmensuan		3.15	96.158		12.901597
3	3	pinguosuan		1.86	156.547		47.949288

Name: 202401001_youjisuan_sample015, Date: 01-Oct-2024, Time: 19:55:43, ID: , Description:

jiushisuan

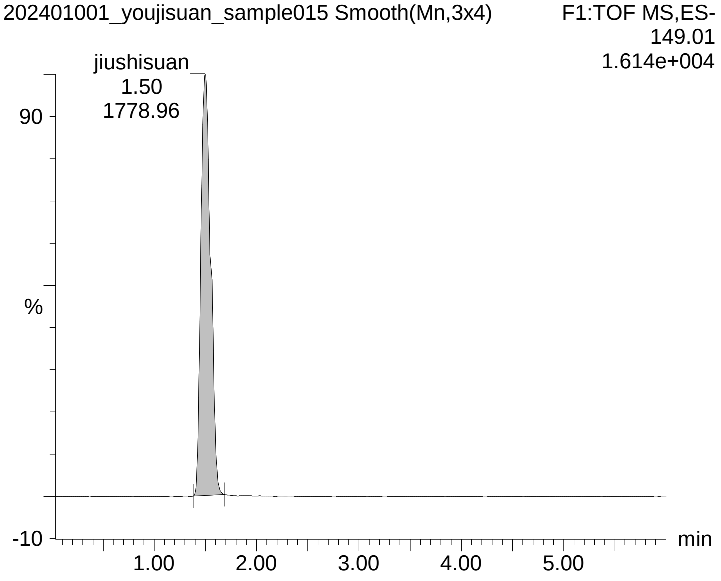

ningmensuan

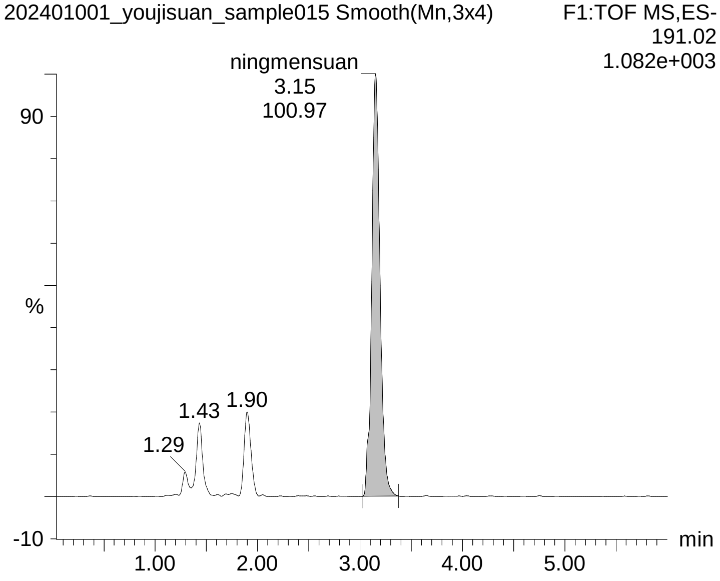

pinguosuan

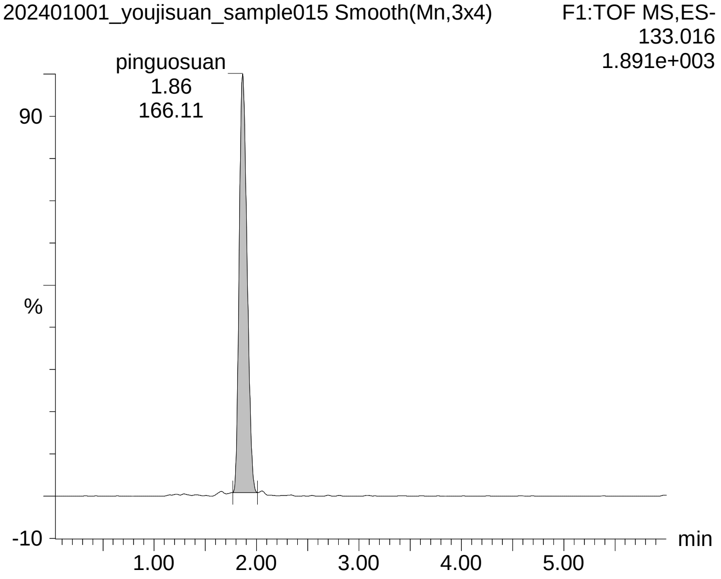

	#	Name	Sample Text	RT	Area	Std. Conc	Conc.
1	1	jiushisuan		1.50	1778.964		360.580127
2	2	ningmensuan		3.15	100.967		13.602464
3	3	pinguosuan		1.86	166.107		51.972847

Name: 202401001_youjisuan_sample016, Date: 01-Oct-2024, Time: 20:02:43, ID: , Description:

jiushisuan

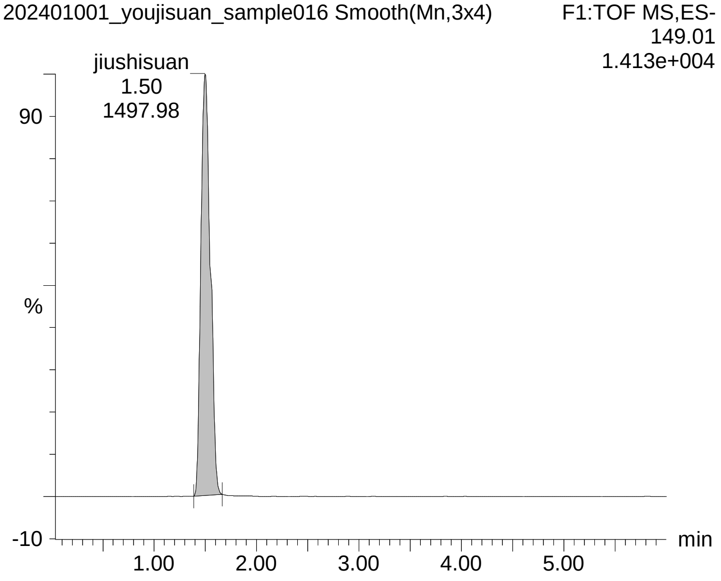

ningmensuan

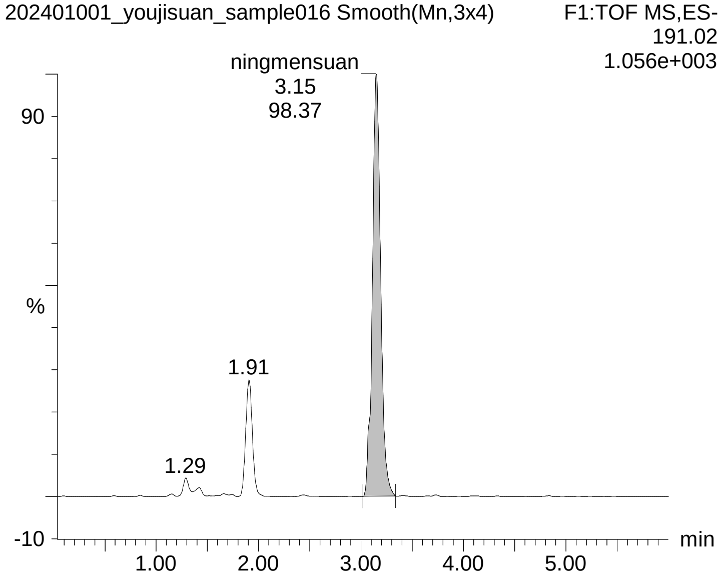

pinguosuan

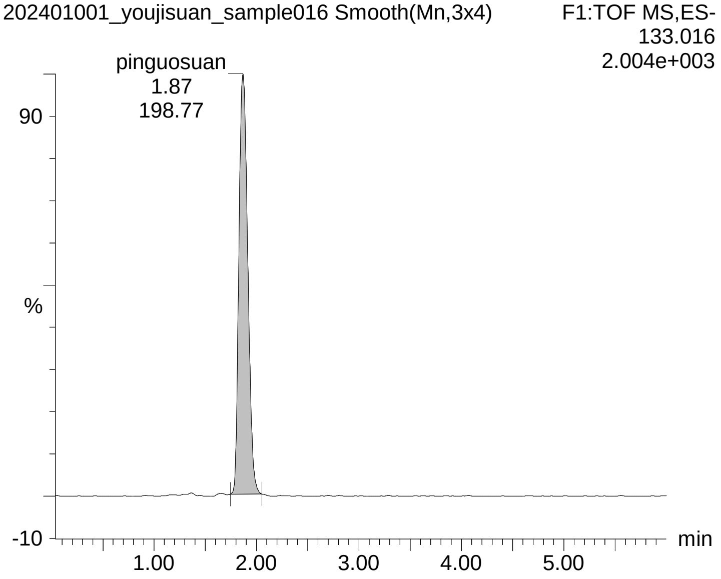

	#	Name	Sample Text	RT	Area	Std. Conc	Conc.
1	1	jiushisuan		1.50	1497.982		263.234224
2	2	ningmensuan		3.15	98.370		13.223976
3	3	pinguosuan		1.87	198.774		66.654742

Name: 202401001_youjisuan_sample017, Date: 01-Oct-2024, Time: 20:09:43, ID: , Description:

jiushisuan

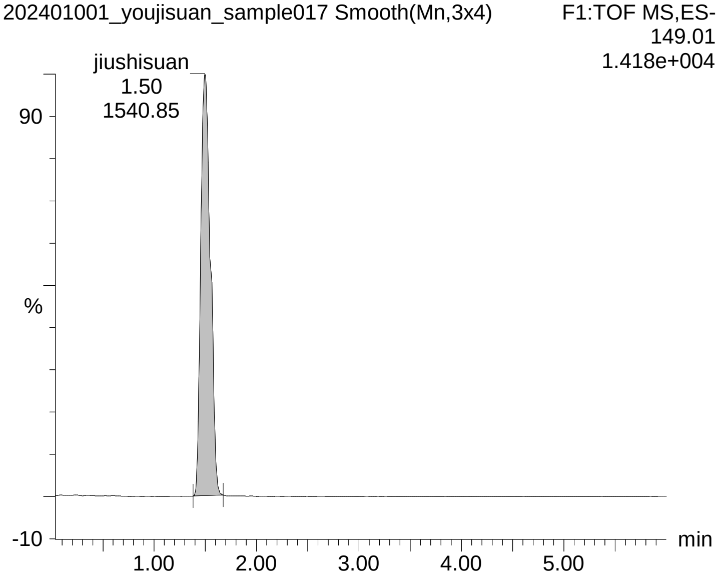

ningmensuan

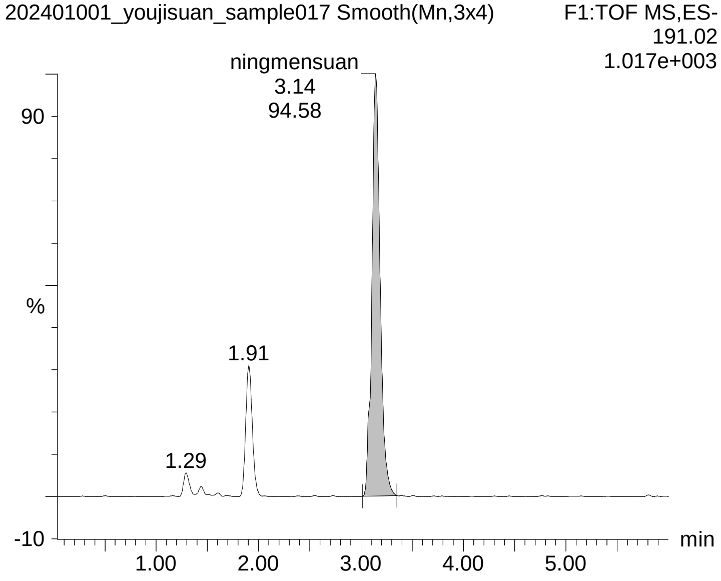

pinguosuan

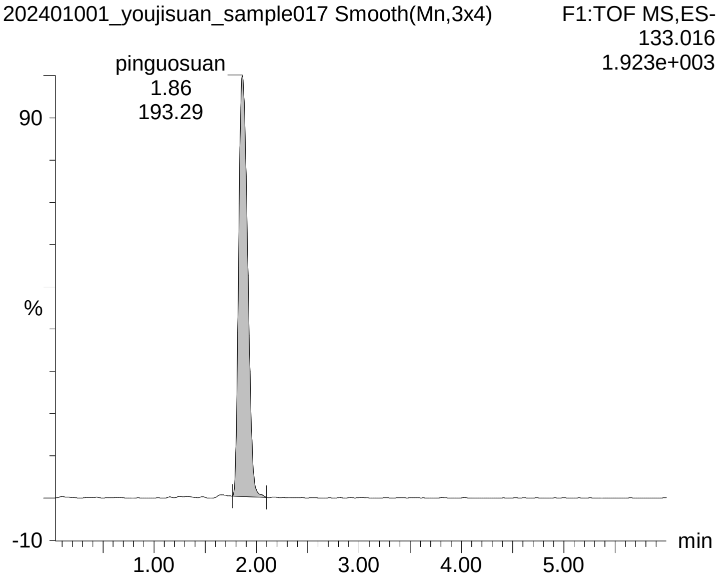

	#	Name	Sample Text	RT	Area	Std. Conc	Conc.
1	1	jiushisuan		1.50	1540.848		275.966165
2	2	ningmensuan		3.14	94.585		12.672347
3	3	pinguosuan		1.86	193.290		64.079090

Name: 202401001_youjisuan_sample018, Date: 01-Oct-2024, Time: 20:16:44, ID: , Description:

jiushisuan

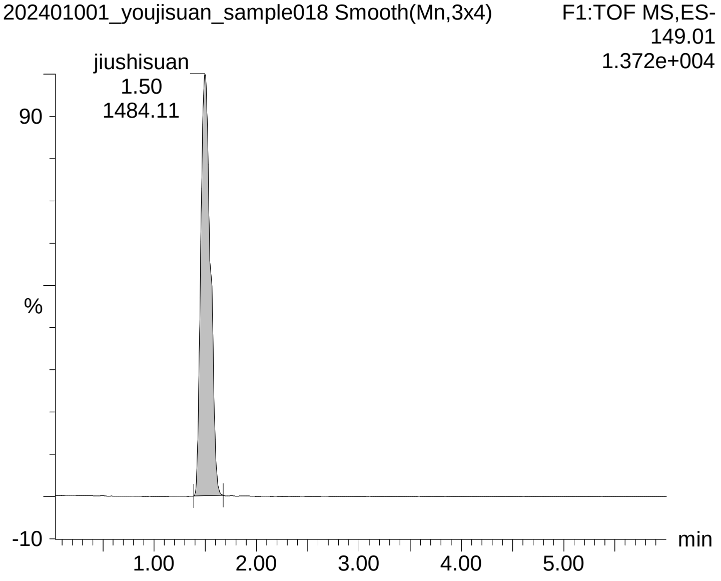

ningmensuan

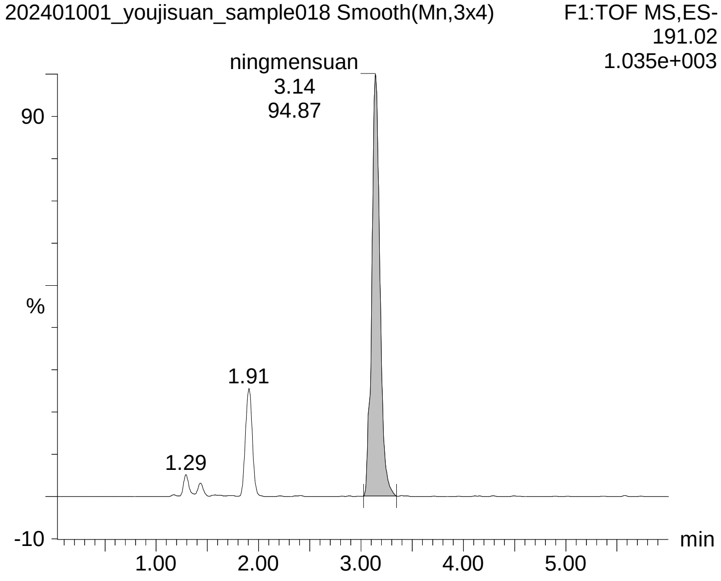

pinguosuan

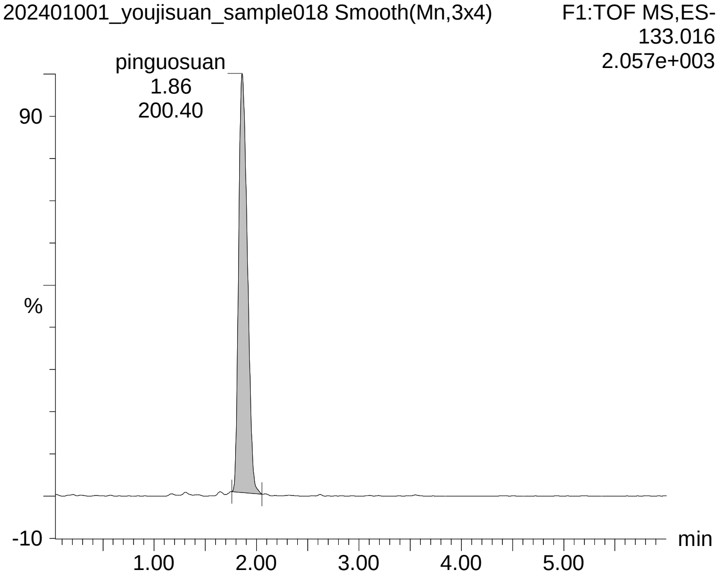

	#	Name	Sample Text	RT	Area	Std. Conc	Conc.
1	1	jiushisuan		1.50	1484.114		259.237870
2	2	ningmensuan		3.14	94.868		12.713592
3	3	pinguosuan		1.86	200.400		67.427859

Name: 202401001_youjisuan_sample019, Date: 01-Oct-2024, Time: 20:23:44, ID: , Description:

jiushisuan

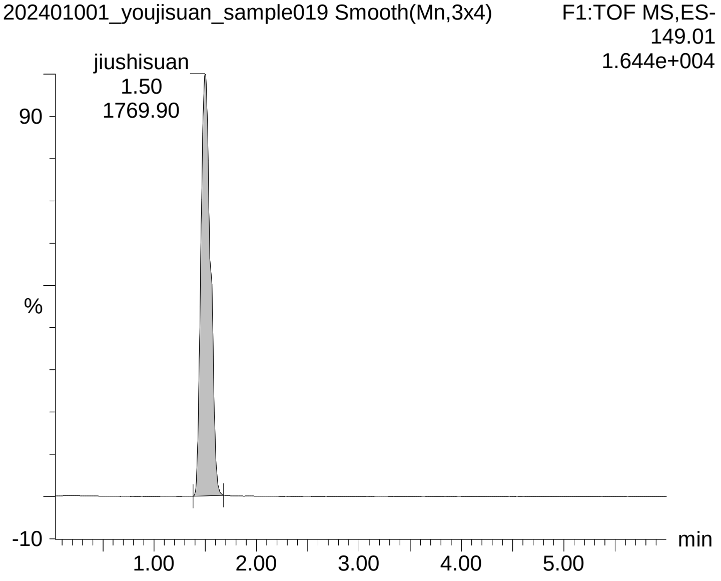

ningmensuan

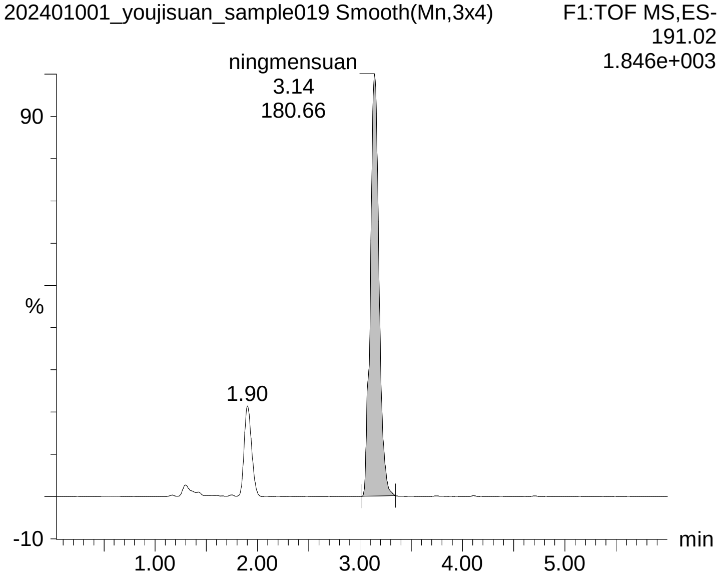

pinguosuan

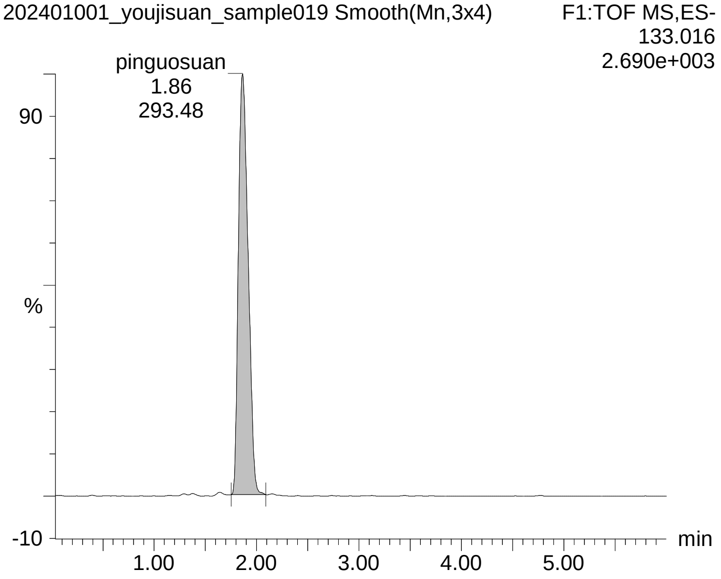

	#	Name	Sample Text	RT	Area	Std. Conc	Conc.
1	1	jiushisuan		1.50	1769.901		356.792302
2	2	ningmensuan		3.14	180.655		25.216242
3	3	pinguosuan		1.86	293.477		122.016148

Name: 202401001_youjisuan_sample020, Date: 01-Oct-2024, Time: 20:30:44, ID: , Description:

jiushisuan

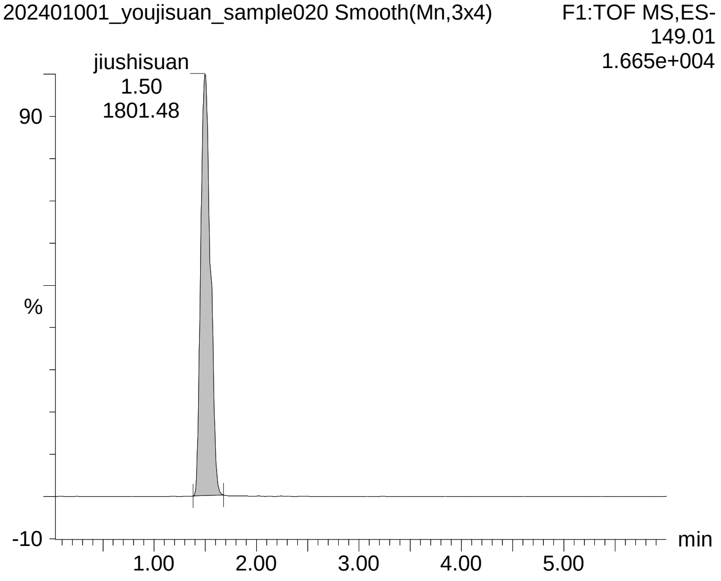

ningmensuan

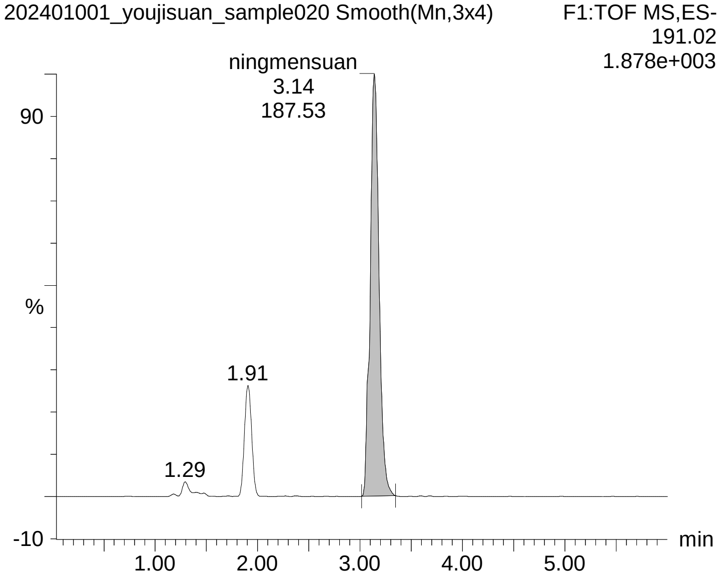

pinguosuan

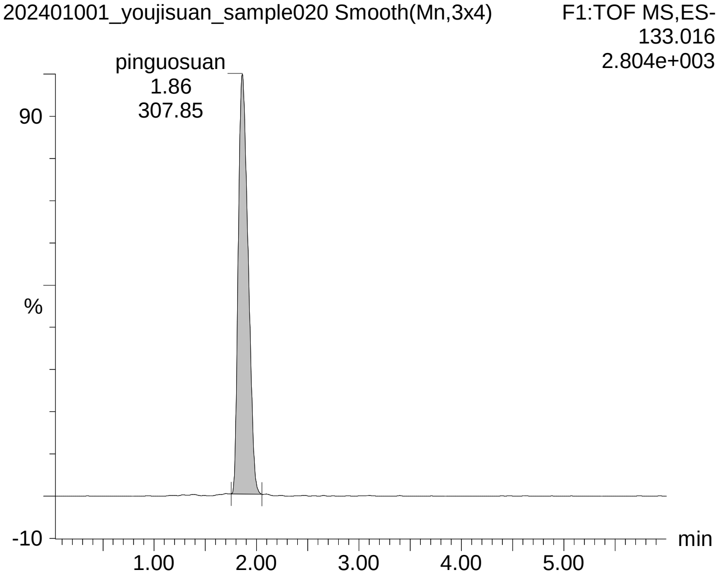

	#	Name	Sample Text	RT	Area	Std. Conc	Conc.
1	1	jiushisuan		1.50	1801.484		370.250507
2	2	ningmensuan		3.14	187.528		26.217917
3	3	pinguosuan		1.86	307.849		133.290742

project_wangzhonghua_BeiMu
Dataset: Untitled
Last Altered: Friday, October 11, 2024 15:42:35 China Standard Time
Printed: Friday, October 11, 2024 15:43:34 China Standard Time

Name: 202401001_youjisuan_sample021, Date: 01-Oct-2024, Time: 20:37:44, ID: , Description:

jiushisuan

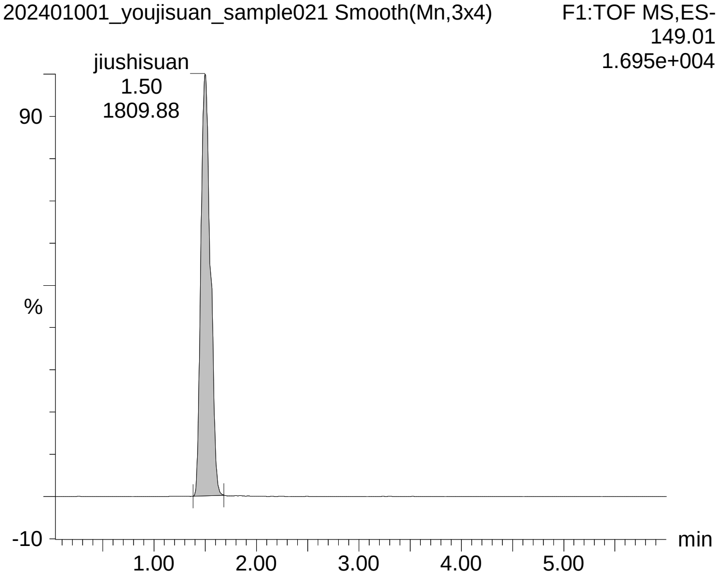

ningmensuan

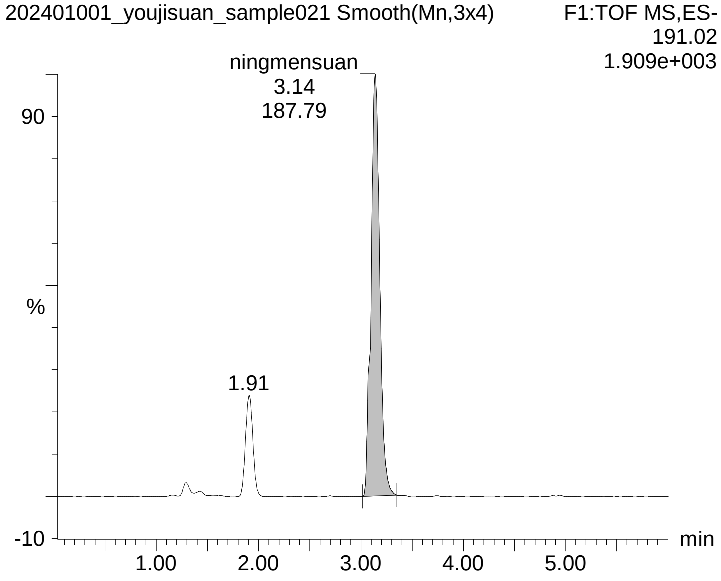

pinguosuan

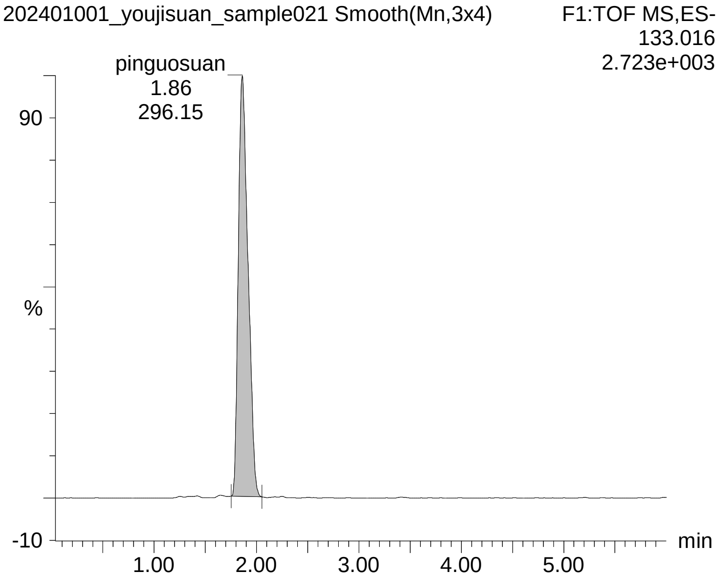

	#	Name	Sample Text	RT	Area	Std. Conc	Conc.
1	1	jiushisuan		1.50	1809.884		373.956861
2	2	ningmensuan		3.14	187.791		26.256247
3	3	pinguosuan		1.86	296.150		124.025784

Name: 202401001_youjisuan_sample022, Date: 01-Oct-2024, Time: 20:44:45, ID: , Description:

jiushisuan

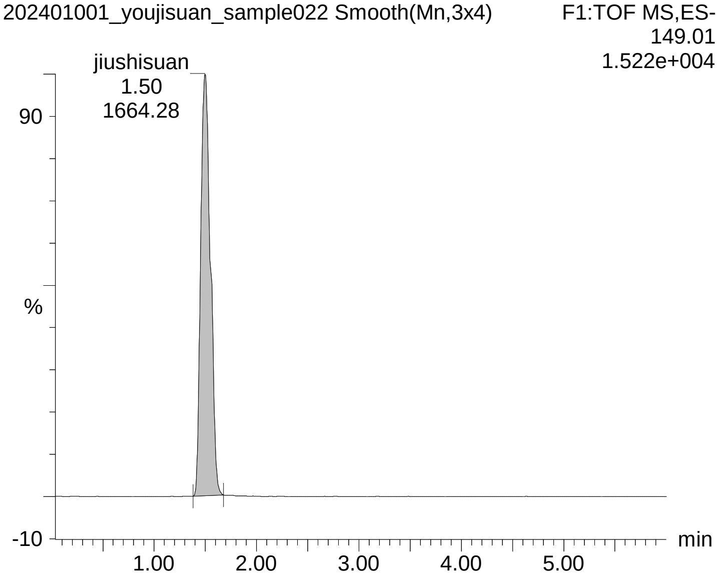

ningmensuan

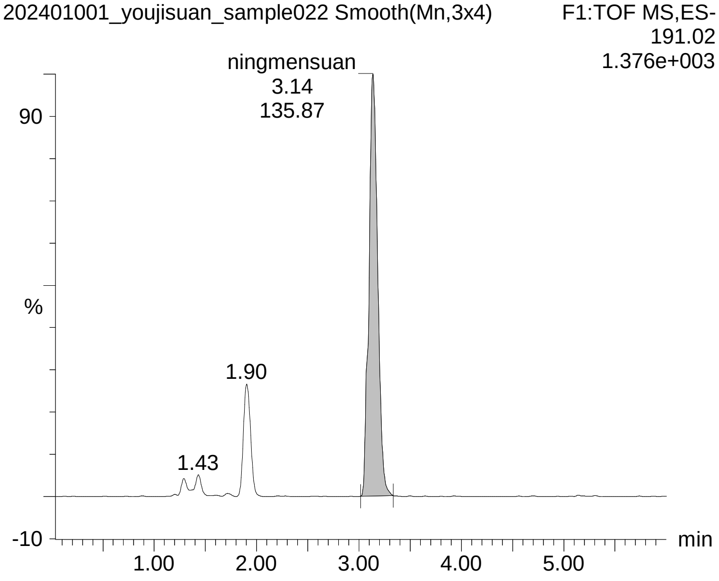

pinguosuan

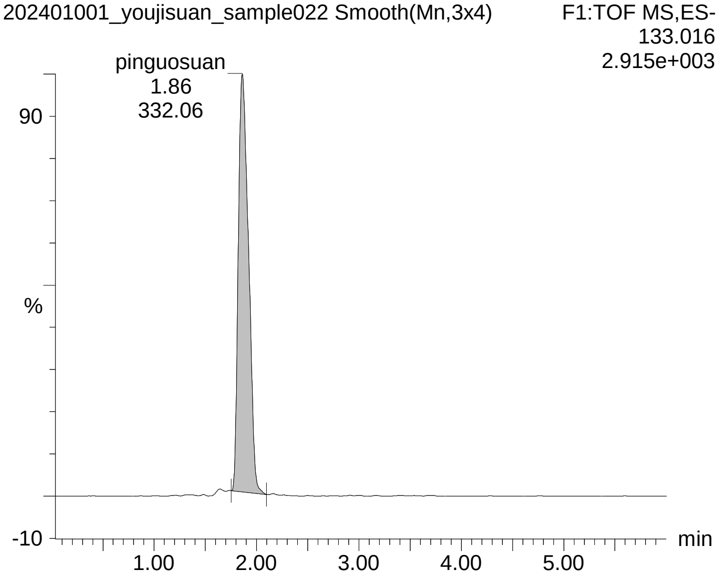

	#	Name	Sample Text	RT	Area	Std. Conc	Conc.
1	1	jiushisuan		1.50	1664.276		316.389094
2	2	ningmensuan		3.14	135.869		18.689103
3	3	pinguosuan		1.86	332.063		155.620718

project_wangzhonghua_BeiMu
Dataset: Untitled
Last Altered: Friday, October 11, 2024 15:42:35 China Standard Time
Printed: Friday, October 11, 2024 15:43:34 China Standard Time

Name: 202401001_youjisuan_sample023, Date: 01-Oct-2024, Time: 20:51:45, ID: , Description:

jiushisuan

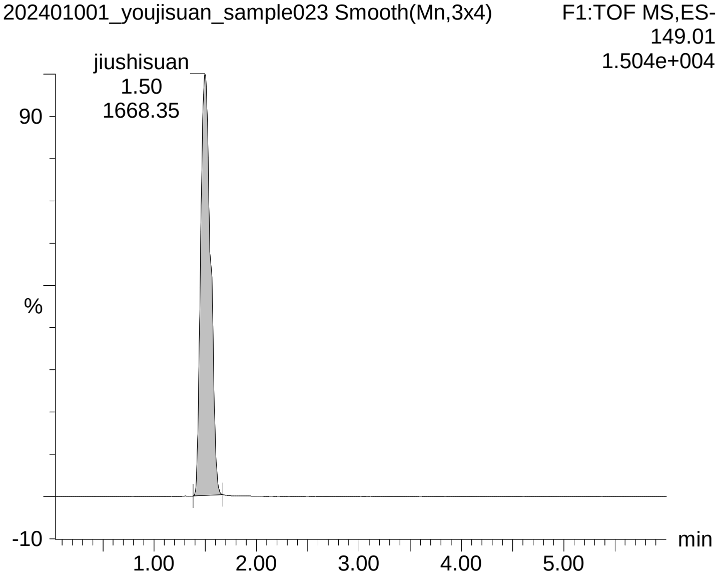

ningmensuan

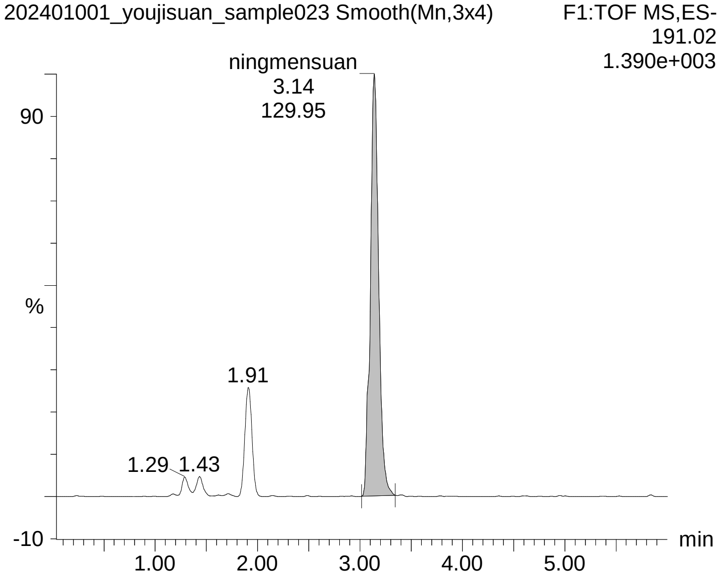

pinguosuan

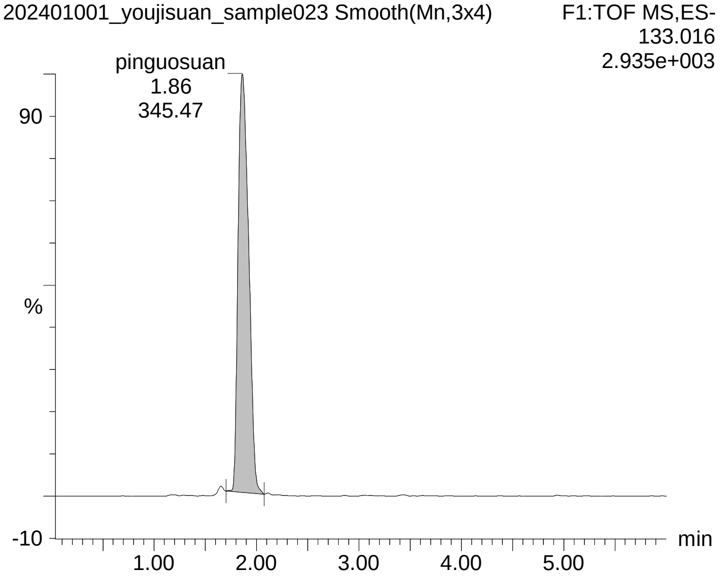

	#	Name	Sample Text	RT	Area	Std. Conc	Conc.
1	1	jiushisuan		1.50	1668.346		317.833444
2	2	ningmensuan		3.14	129.946		17.825881
3	3	pinguosuan		1.86	345.469		170.674384

Name: 202401001_youjisuan_sample024, Date: 01-Oct-2024, Time: 20:59:43, ID: , Description:

jiushisuan

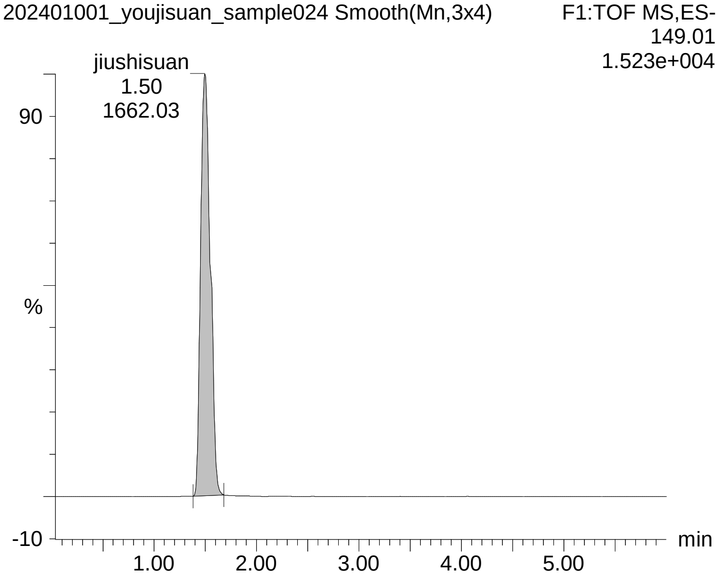

ningmensuan

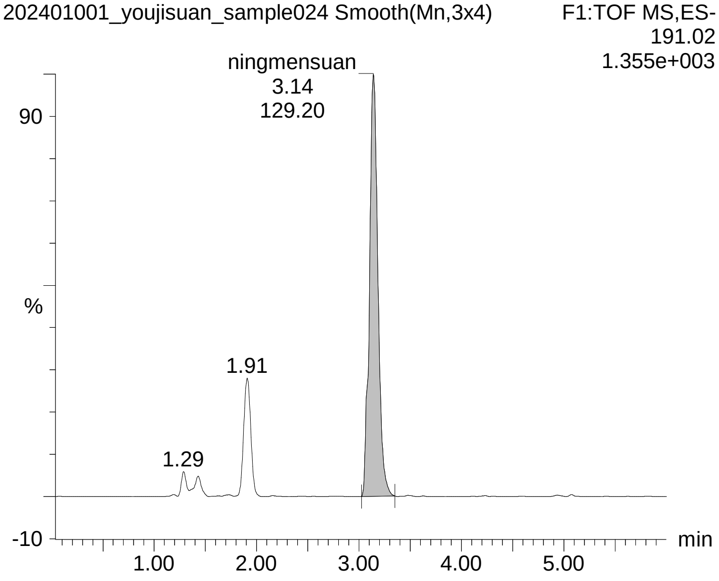

pinguosuan

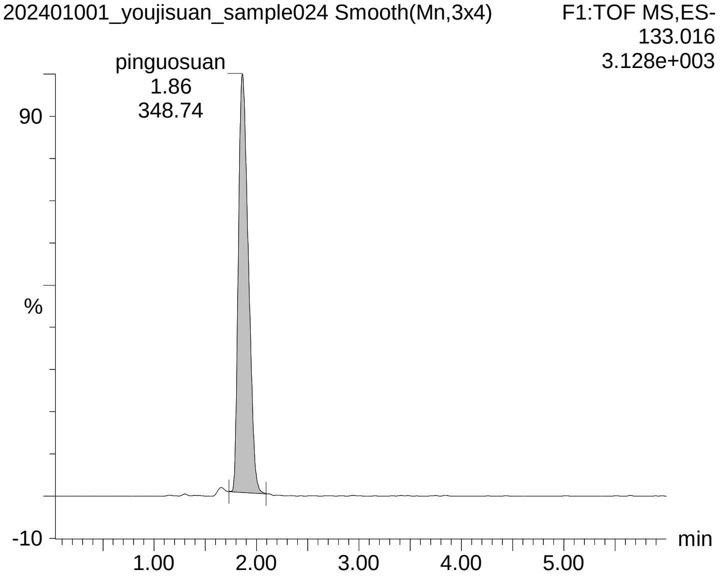

	#	Name	Sample Text	RT	Area	Std. Conc	Conc.
1	1	jiushisuan		1.50	1662.028		315.594757
2	2	ningmensuan		3.14	129.200		17.717159
3	3	pinguosuan		1.86	348.739		174.766433

project_wangzhonghua_BeiMu
Dataset: Untitled
Last Altered: Friday, October 11, 2024 15:42:35 China Standard Time
Printed: Friday, October 11, 2024 15:43:34 China Standard Time

Name: 202401001_youjisuan_sample025, Date: 01-Oct-2024, Time: 21:06:42, ID: , Description:

jiushisuan

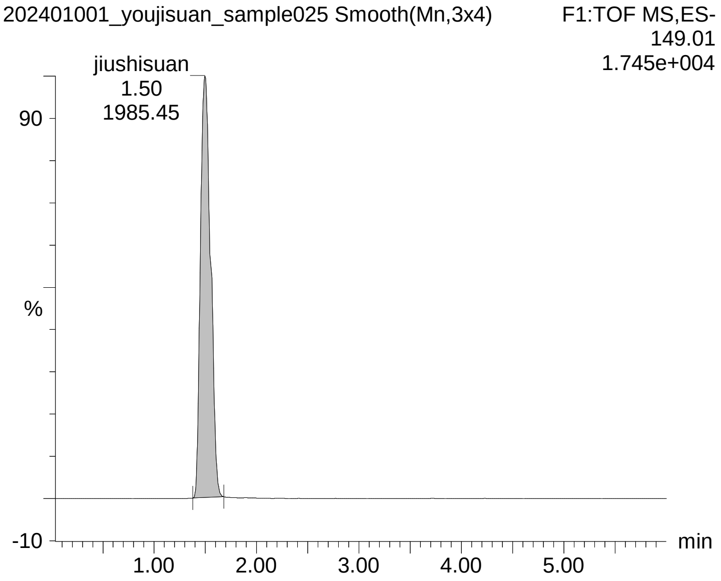

ningmensuan

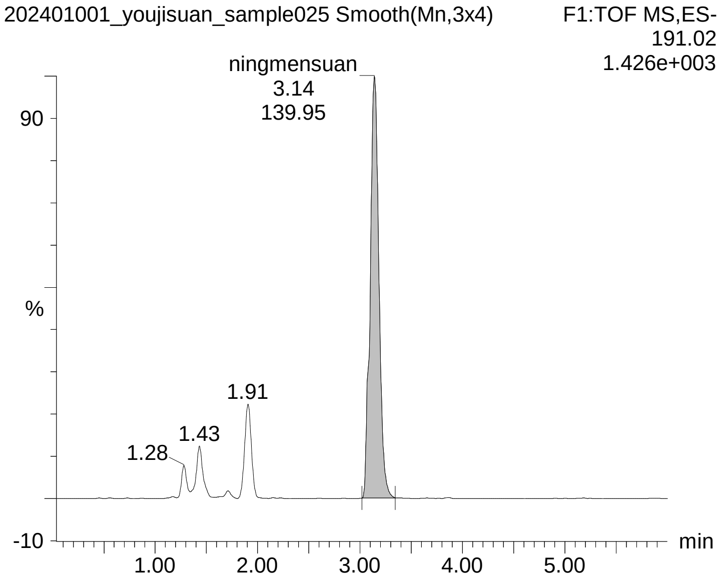

pinguosuan

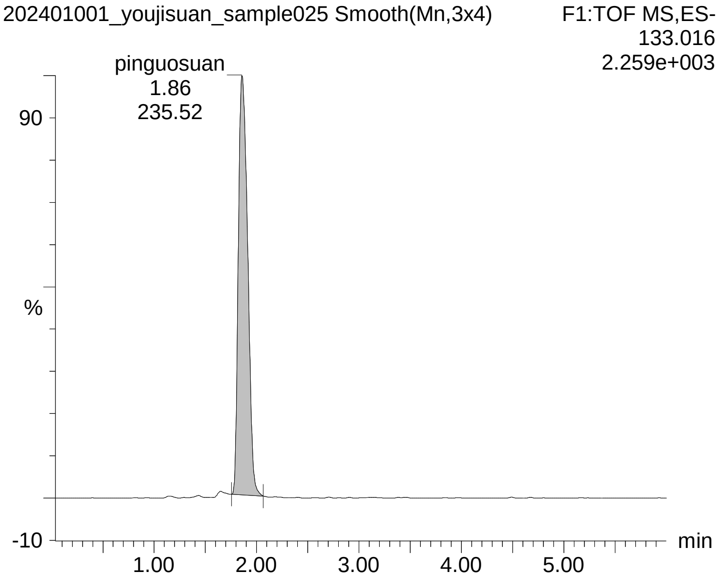

	#	Name	Sample Text	RT	Area	Std. Conc	Conc.
1	1	jiushisuan		1.50	1985.446		468.271107
2	2	ningmensuan		3.14	139.947		19.283433
3	3	pinguosuan		1.86	235.516		85.324383

Name: 202401001_youjisuan_sample026, Date: 01-Oct-2024, Time: 21:13:36, ID: , Description:

jiushisuan

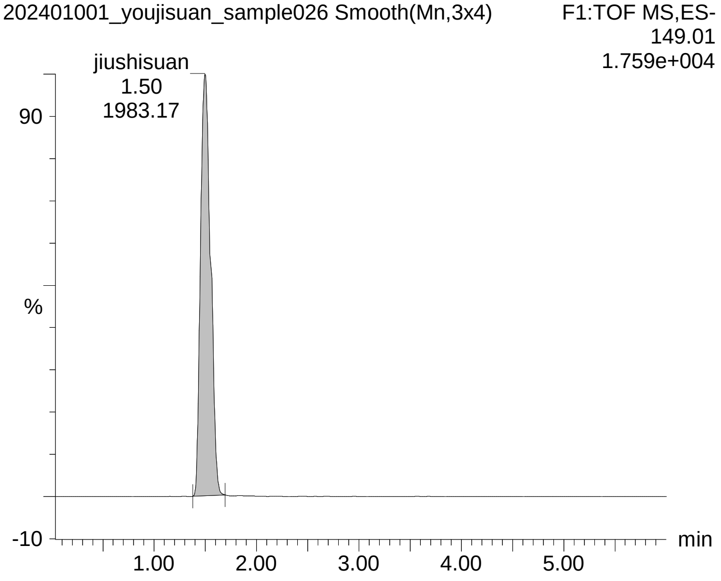

ningmensuan

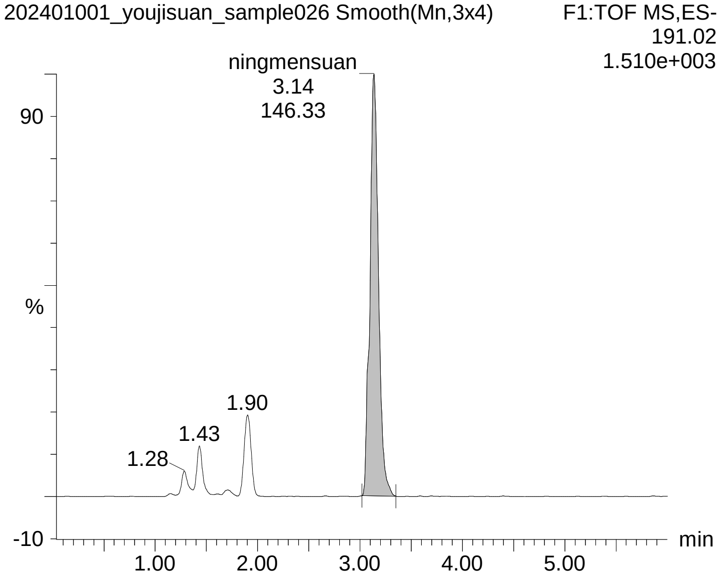

pinguosuan

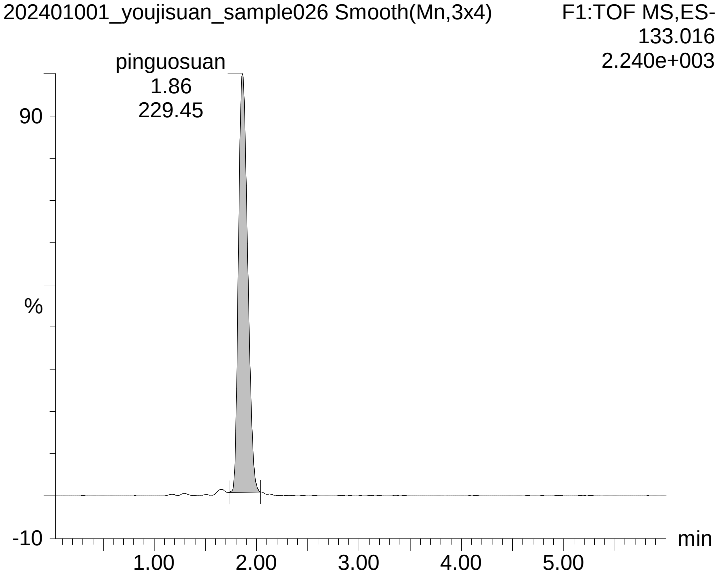

	#	Name	Sample Text	RT	Area	Std. Conc	Conc.
1	1	jiushisuan		1.50	1983.166		466.773412
2	2	ningmensuan		3.14	146.334		20.214278
3	3	pinguosuan		1.86	229.447		82.050823

project_wangzhonghua_BeiMu
Dataset: Untitled
Last Altered: Friday, October 11, 2024 15:42:35 China Standard Time
Printed: Friday, October 11, 2024 15:43:34 China Standard Time

Name: 202401001_youjisuan_sample027, Date: 01-Oct-2024, Time: 21:20:37, ID: , Description:

jiushisuan

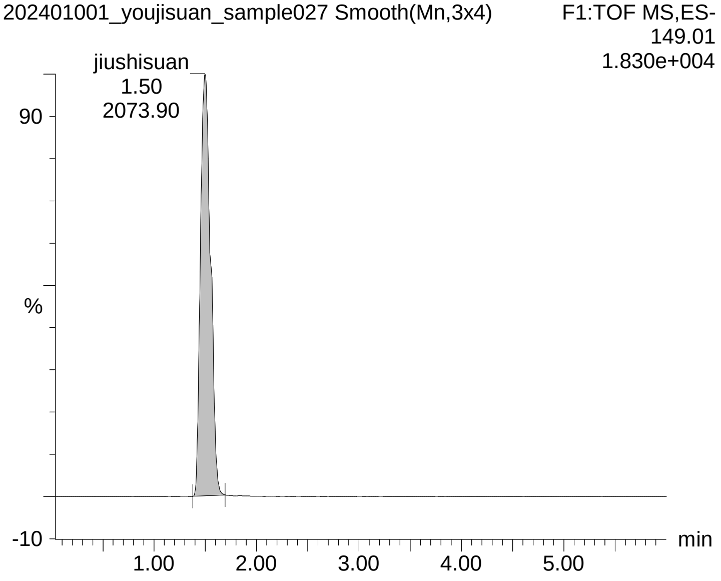

ningmensuan

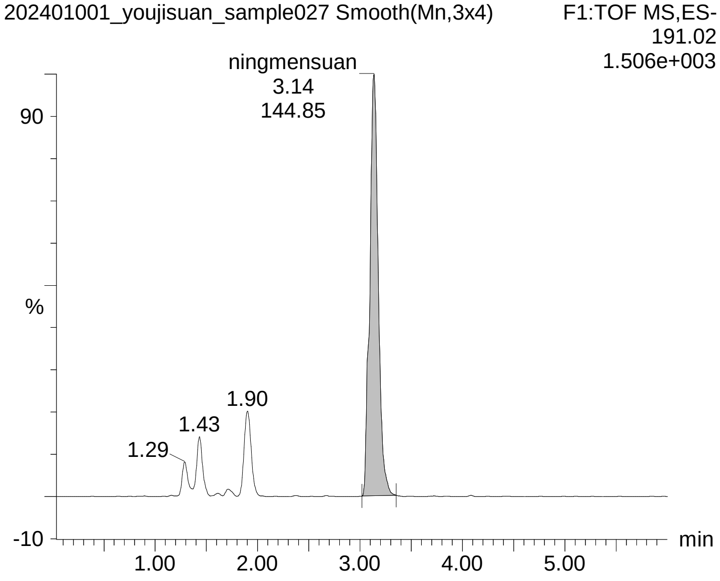

pinguosuan

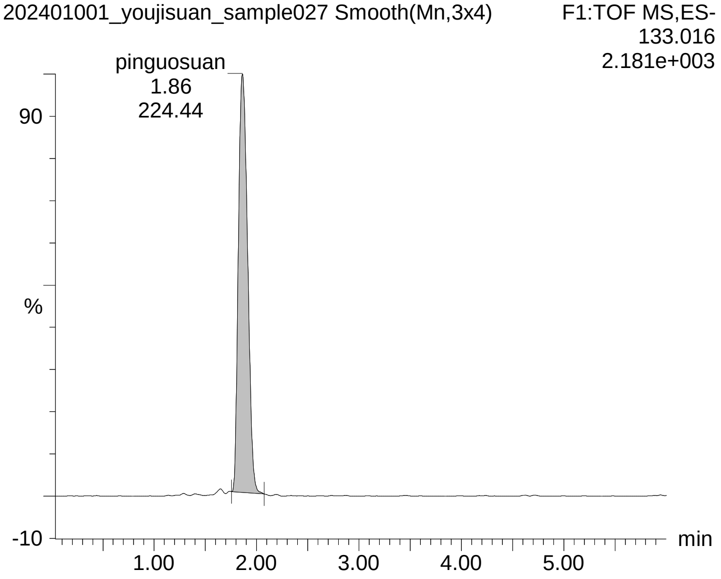

	#	Name	Sample Text	RT	Area	Std. Conc	Conc.
1	1	jiushisuan		1.50	2073.899		534.894150
2	2	ningmensuan		3.14	144.855		19.998728
3	3	pinguosuan		1.86	224.445		79.414012

project_wangzhonghua_BeiMu
Dataset: Untitled
Last Altered: Friday, October 11, 2024 15:42:35 China Standard Time
Printed: Friday, October 11, 2024 15:43:34 China Standard Time

Name: 202401001_youjisuan_sample028, Date: 01-Oct-2024, Time: 21:27:37, ID: , Description:

jiushisuan

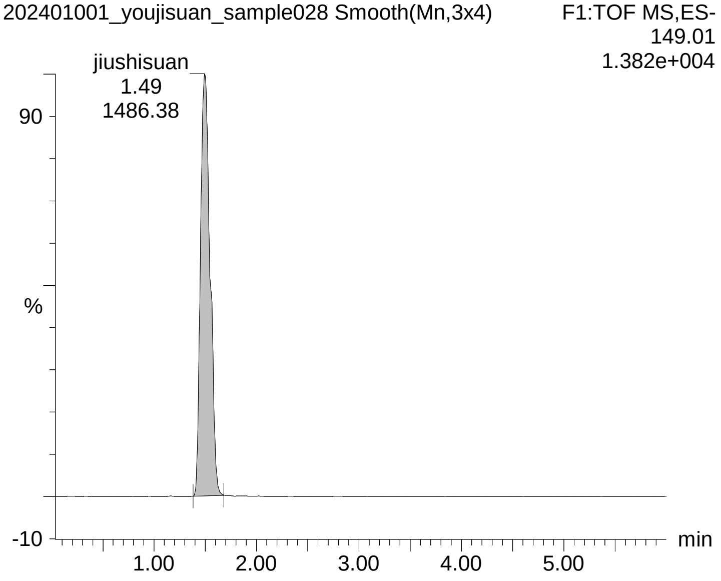

ningmensuan

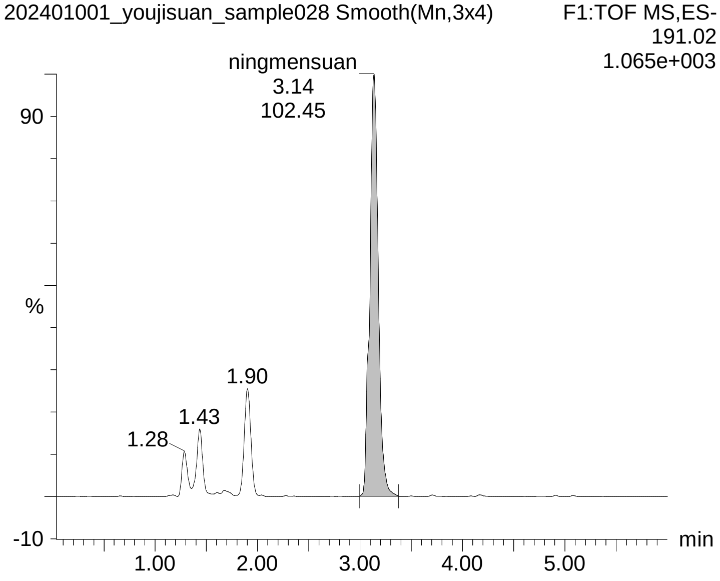

pinguosuan

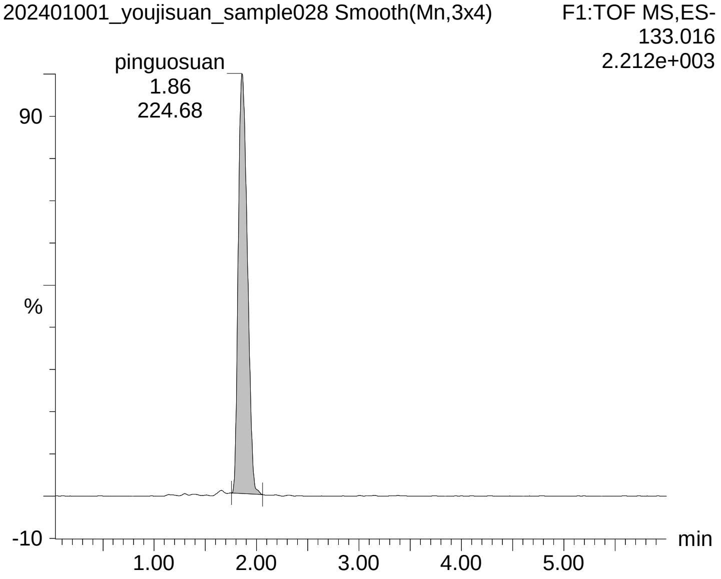

	#	Name	Sample Text	RT	Area	Std. Conc	Conc.
1	1	jiushisuan		1.49	1486.375		259.885513
2	2	ningmensuan		3.14	102.448		13.818306
3	3	pinguosuan		1.86	224.680		79.536699

project_wangzhonghua_BeiMu
Dataset: Untitled
Last Altered: Friday, October 11, 2024 15:42:35 China Standard Time
Printed: Friday, October 11, 2024 15:43:34 China Standard Time

Name: 202401001_youjisuan_sample029, Date: 01-Oct-2024, Time: 21:34:37, ID: , Description:

jiushisuan

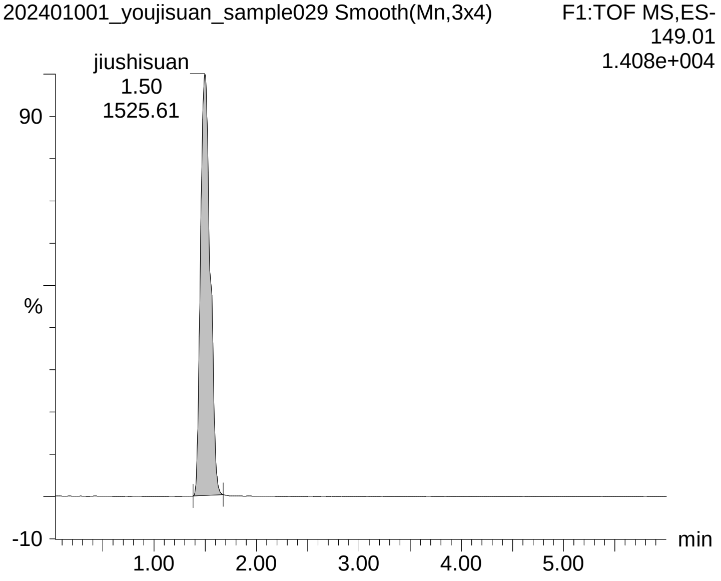

ningmensuan

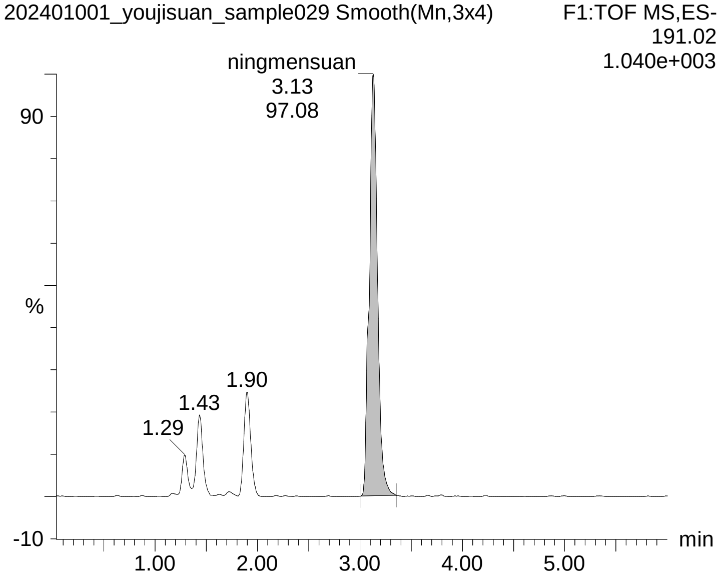

pinguosuan

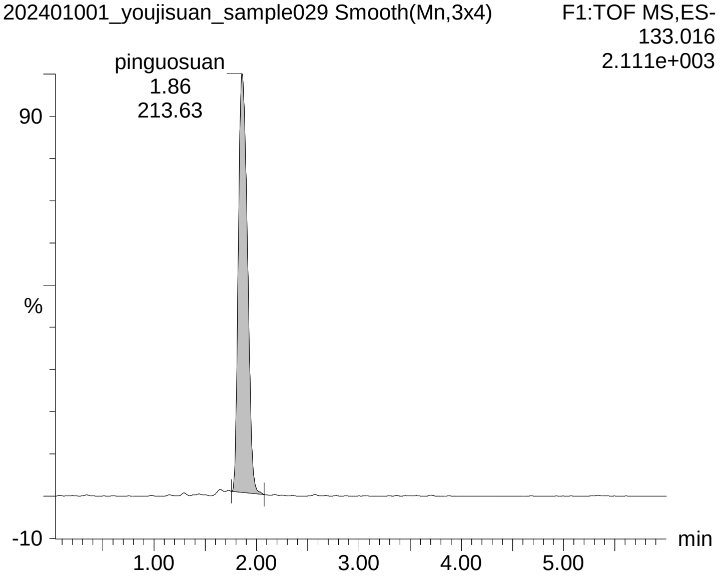

	#	Name	Sample Text	RT	Area	Std. Conc	Conc.
1	1	jiushisuan		1.50	1525.606		271.371081
2	2	ningmensuan		3.13	97.080		13.035970
3	3	pinguosuan		1.86	213.627		73.887190

Name: 202401001_youjisuan_sample030, Date: 01-Oct-2024, Time: 21:41:37, ID: , Description:

jiushisuan

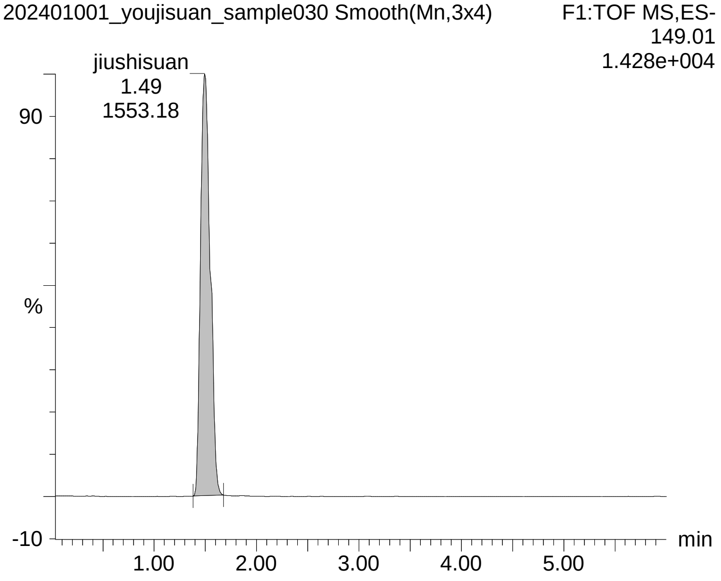

ningmensuan

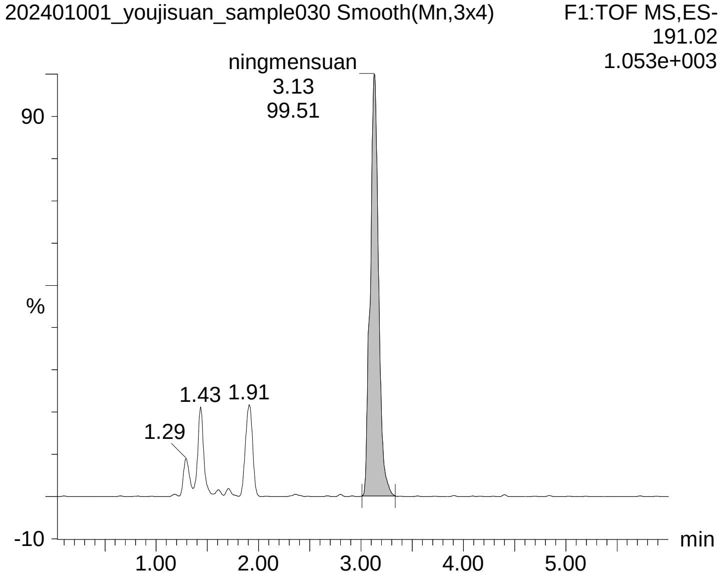

pinguosuan

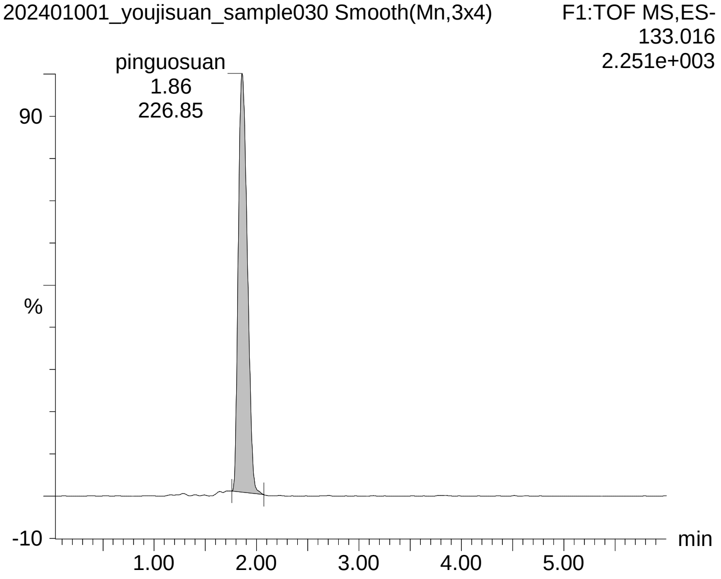

	#	Name	Sample Text	RT	Area	Std. Conc	Conc.
1	1	jiushisuan		1.49	1553.184		279.742701
2	2	ningmensuan		3.13	99.512		13.390411
3	3	pinguosuan		1.86	226.850		80.675109

Name: 202401001_youjisuan_sample031, Date: 01-Oct-2024, Time: 22:23:44, ID: , Description:

jiushisuan

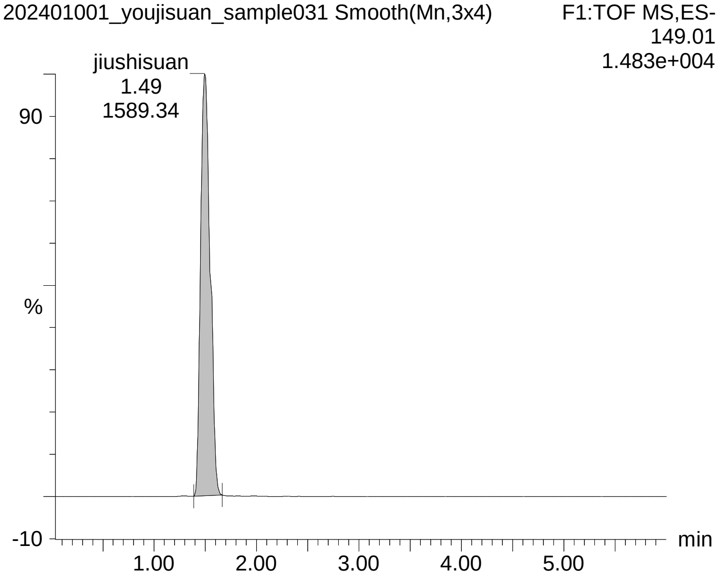

ningmensuan

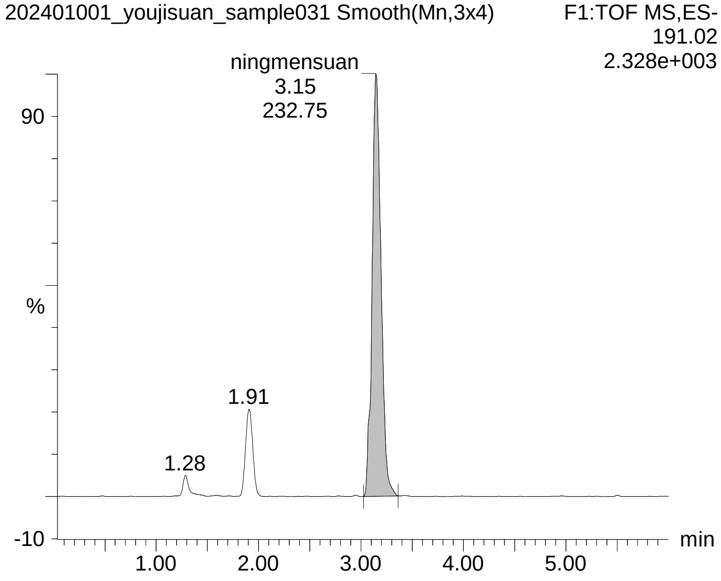

pinguosuan

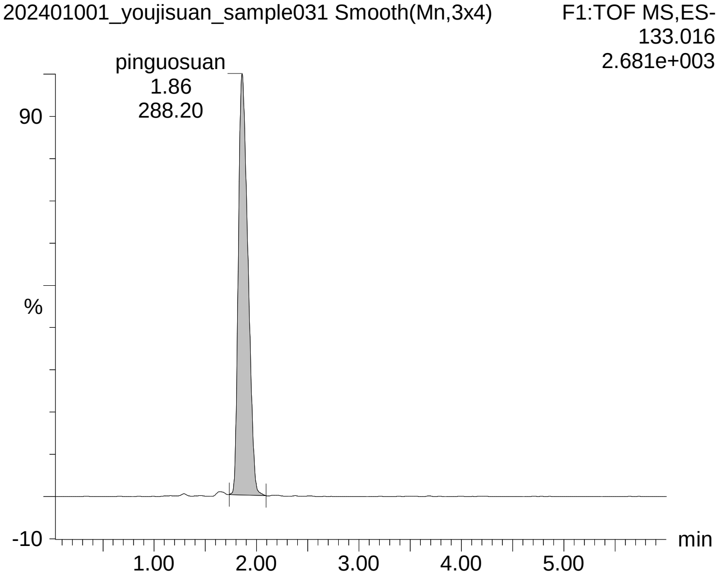

	#	Name	Sample Text	RT	Area	Std. Conc	Conc.
1	1	jiushisuan		1.49	1589.338		291.124564
2	2	ningmensuan		3.15	232.745		32.807870
3	3	pinguosuan		1.86	288.201		118.153941

Name: 202401001_youjisuan_sample032, Date: 01-Oct-2024, Time: 22:30:42, ID: , Description:

jiushisuan

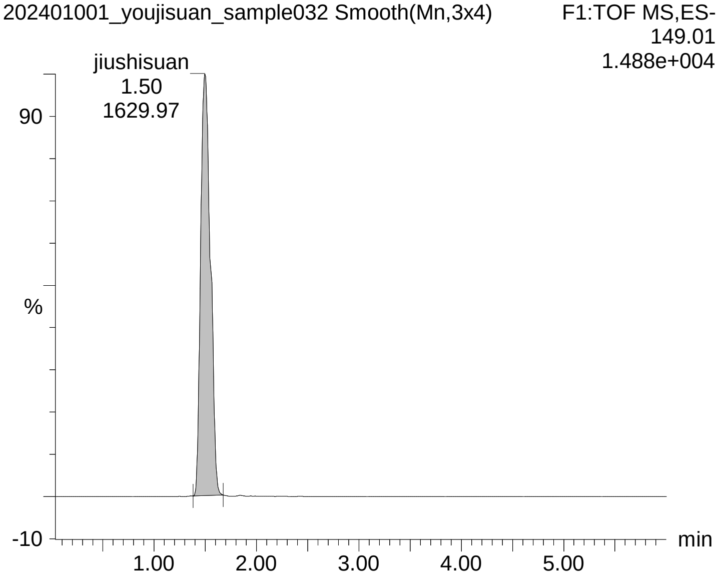

ningmensuan

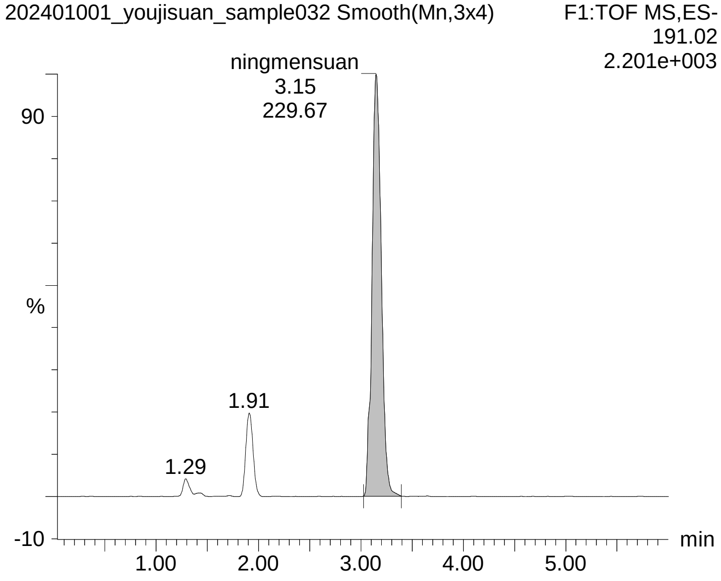

pinguosuan

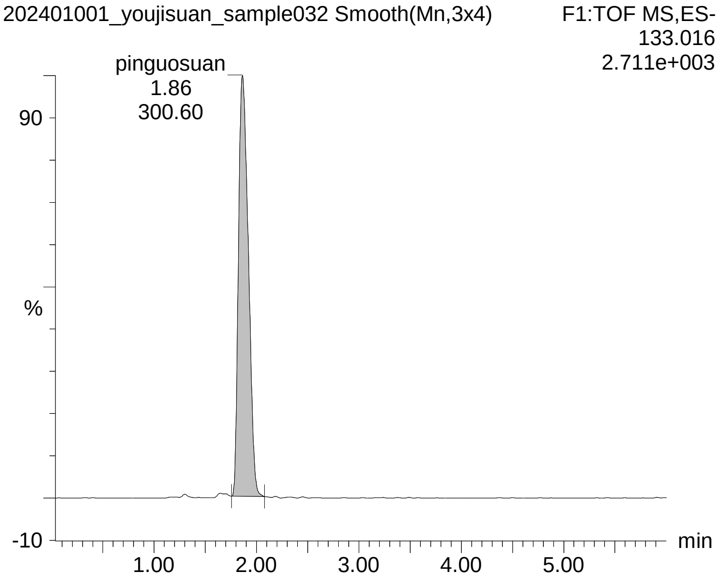

	#	Name	Sample Text	RT	Area	Std. Conc	Conc.
1	1	jiushisuan		1.50	1629.971		304.522477
2	2	ningmensuan		3.15	229.667		32.359280
3	3	pinguosuan		1.86	300.596		127.452932

Name: 202401001_youjisuan_sample033, Date: 01-Oct-2024, Time: 22:37:42, ID: , Description:

jiushisuan

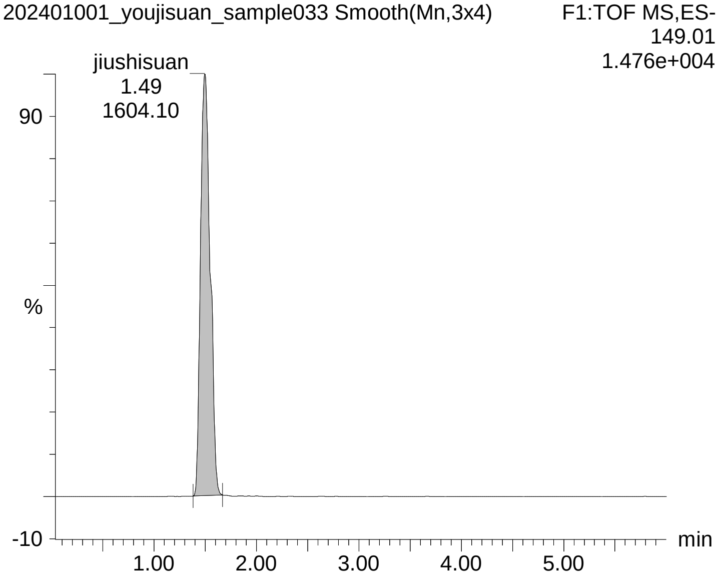

ningmensuan

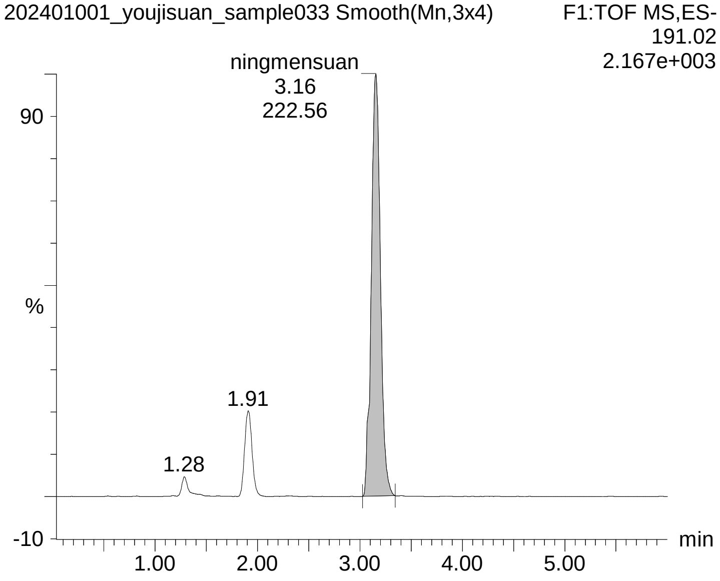

pinguosuan

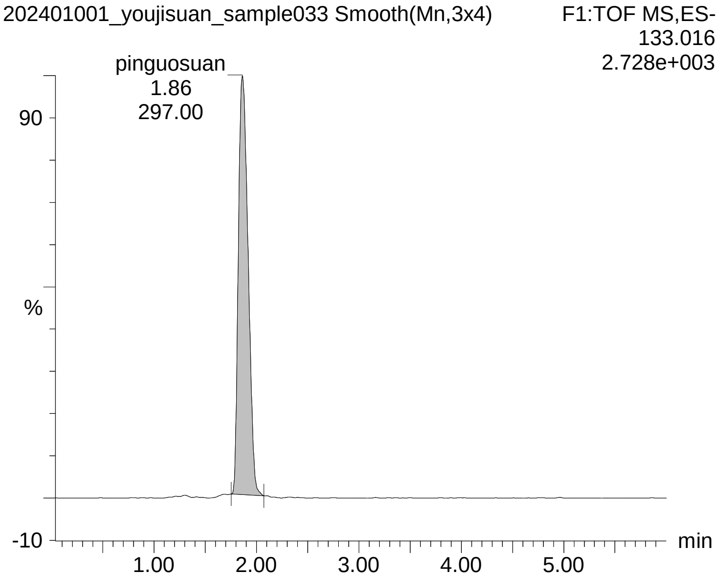

	#	Name	Sample Text	RT	Area	Std. Conc	Conc.
1	1	jiushisuan		1.49	1604.100		295.914300
2	2	ningmensuan		3.16	222.558		31.323210
3	3	pinguosuan		1.86	297.004		124.675722

Name: 202401001_youjisuan_sample034, Date: 01-Oct-2024, Time: 22:44:42, ID: , Description:

jiushisuan

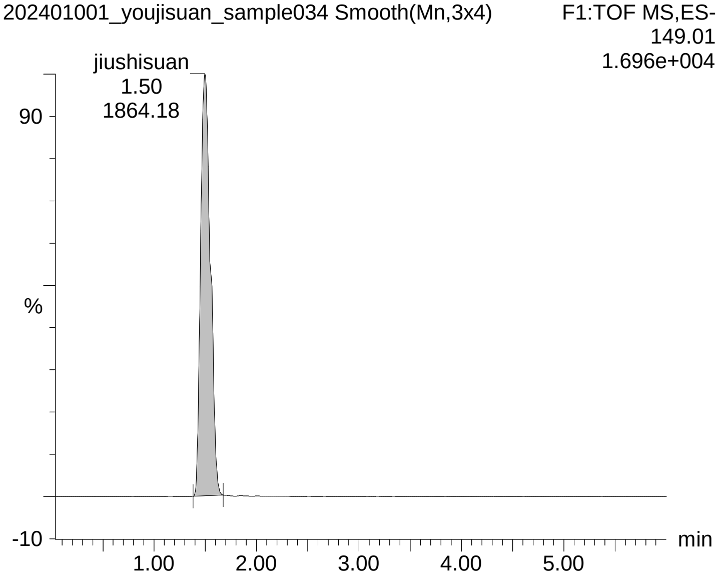

ningmensuan

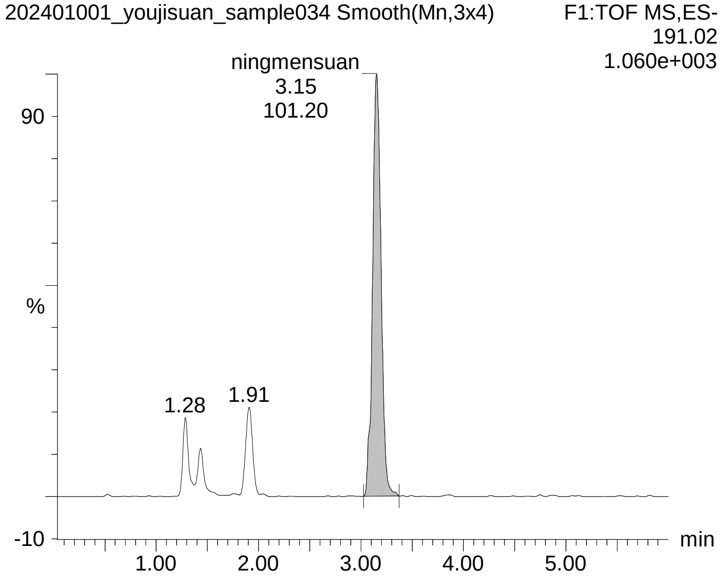

pinguosuan

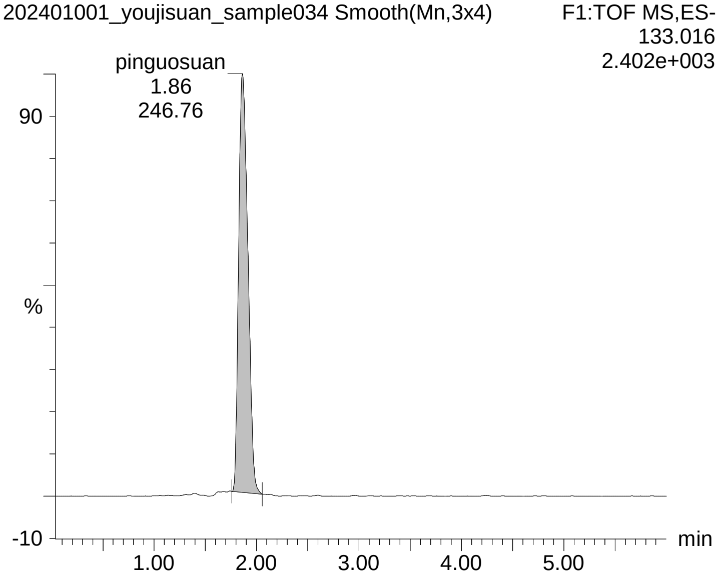

	#	Name	Sample Text	RT	Area	Std. Conc	Conc.
1	1	jiushisuan		1.50	1864.181		399.375110
2	2	ningmensuan		3.15	101.196		13.635839
3	3	pinguosuan		1.86	246.762		91.623311

Name: 202401001_youjisuan_sample035, Date: 01-Oct-2024, Time: 22:51:42, ID: , Description:

jiushisuan

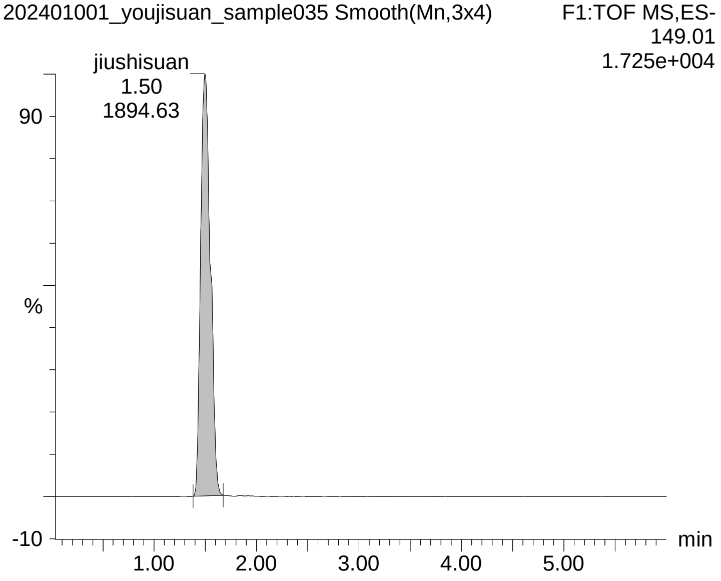

ningmensuan

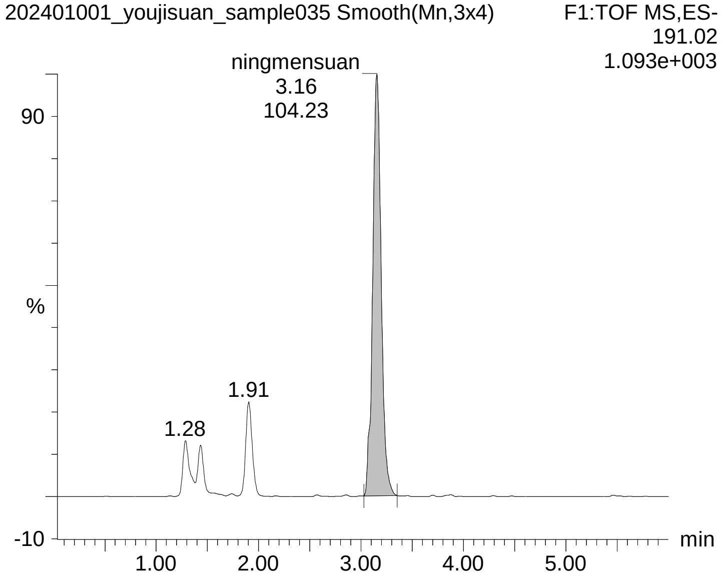

pinguosuan

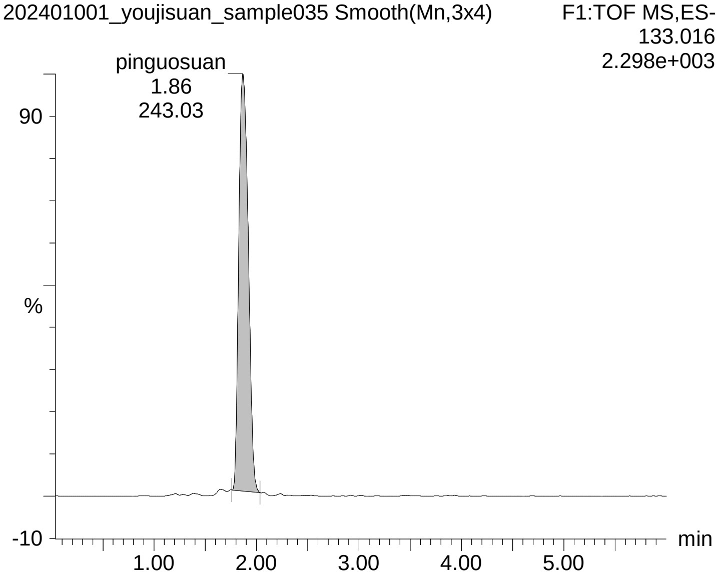

	#	Name	Sample Text	RT	Area	Std. Conc	Conc.
1	1	jiushisuan		1.50	1894.628		414.892438
2	2	ningmensuan		3.16	104.232		14.078307
3	3	pinguosuan		1.86	243.034		89.500041

Name: 202401001_youjisuan_sample036, Date: 01-Oct-2024, Time: 22:58:42, ID: , Description:

jiushisuan

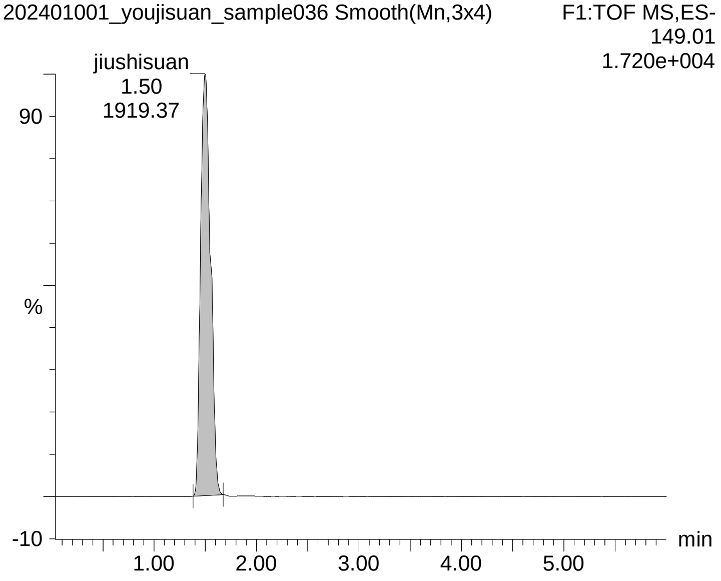

ningmensuan

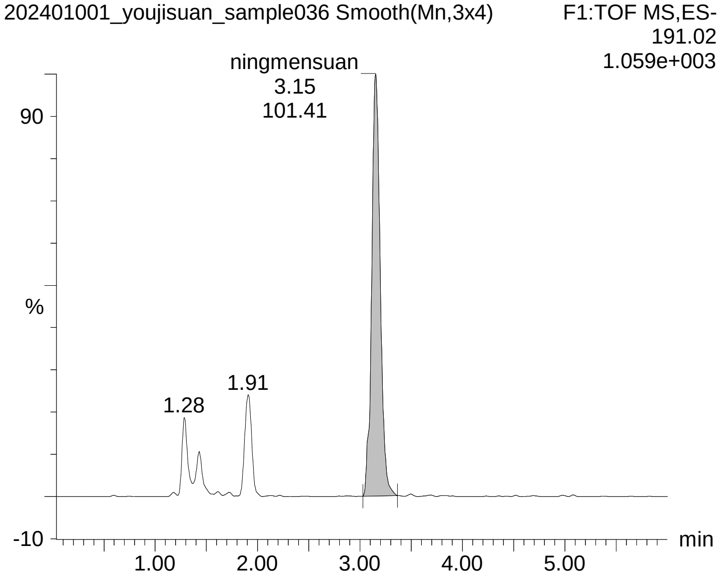

pinguosuan

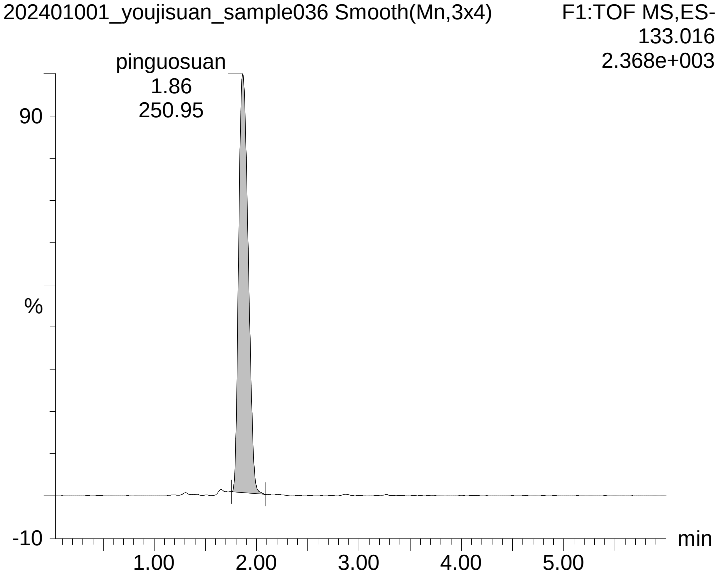

	#	Name	Sample Text	RT	Area	Std. Conc	Conc.
1	1	jiushisuan		1.50	1919.369		428.287731
2	2	ningmensuan		3.15	101.410		13.667027
3	3	pinguosuan		1.86	250.950		94.052686

project_wangzhonghua_BeiMu
Dataset: Untitled
Last Altered: Friday, October 11, 2024 15:42:35 China Standard Time
Printed: Friday, October 11, 2024 15:43:34 China Standard Time

Name: 202401001_youjisuan_sample037, Date: 01-Oct-2024, Time: 23:05:43, ID: , Description:

jiushisuan

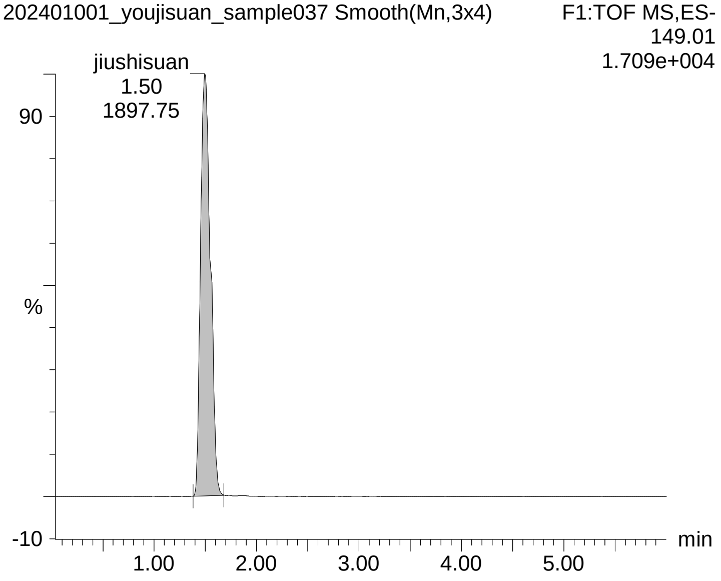

ningmensuan

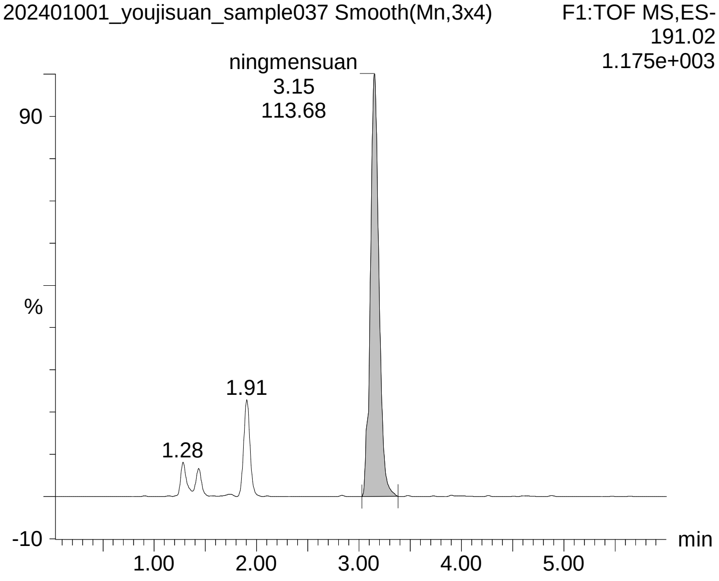

pinguosuan

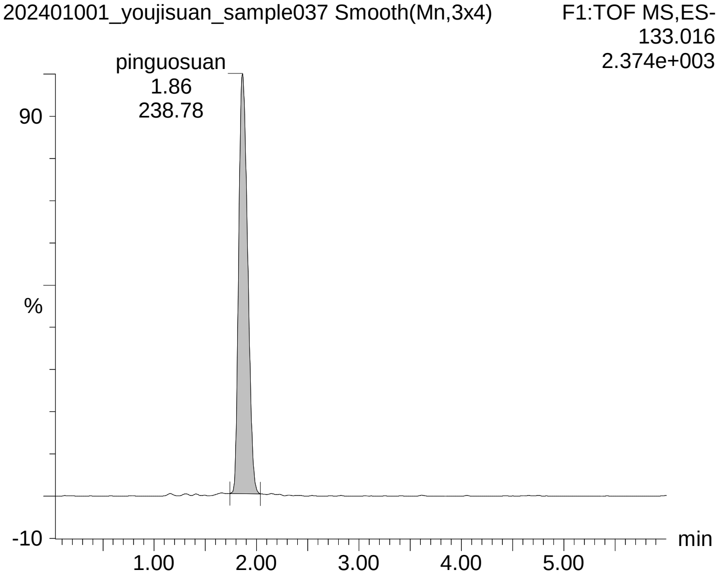

	#	Name	Sample Text	RT	Area	Std. Conc	Conc.
1	1	jiushisuan		1.50	1897.751		416.542403
2	2	ningmensuan		3.15	113.678		15.454973
3	3	pinguosuan		1.86	238.782		87.121453

Name: 202401001_youjisuan_sample038, Date: 01-Oct-2024, Time: 23:12:43, ID: , Description:

jiushisuan

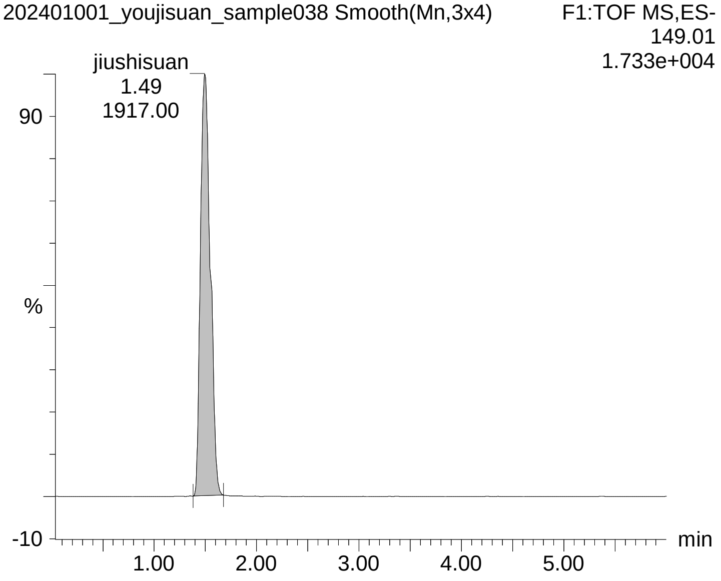

ningmensuan

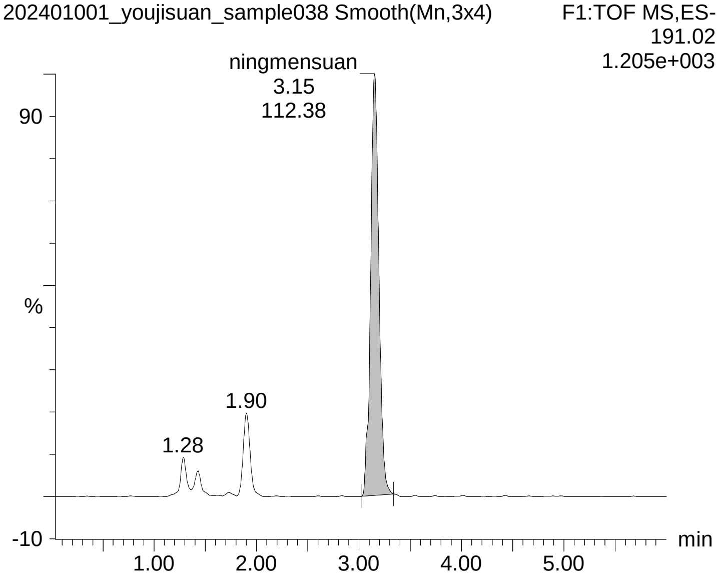

pinguosuan

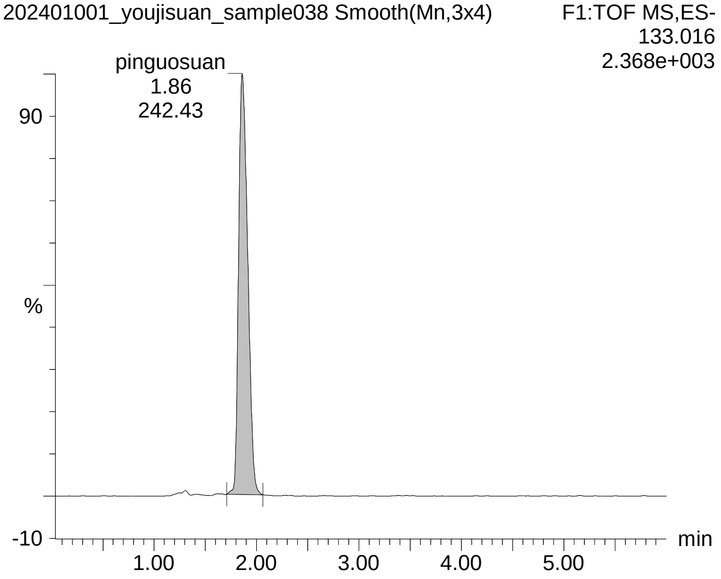

	#	Name	Sample Text	RT	Area	Std. Conc	Conc.
1	1	jiushisuan		1.49	1916.996		426.969797
2	2	ningmensuan		3.15	112.384		15.266384
3	3	pinguosuan		1.86	242.434		89.161658

Name: 202401001_youjisuan_sample039, Date: 01-Oct-2024, Time: 23:19:43, ID: , Description:

jiushisuan

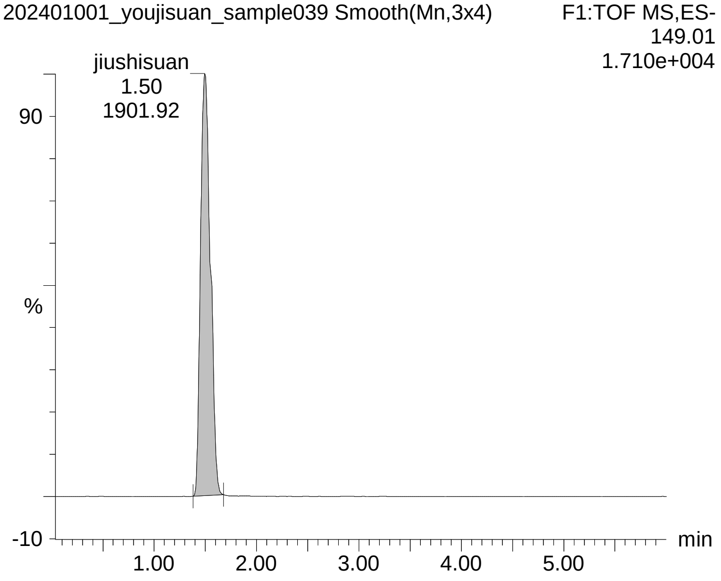

ningmensuan

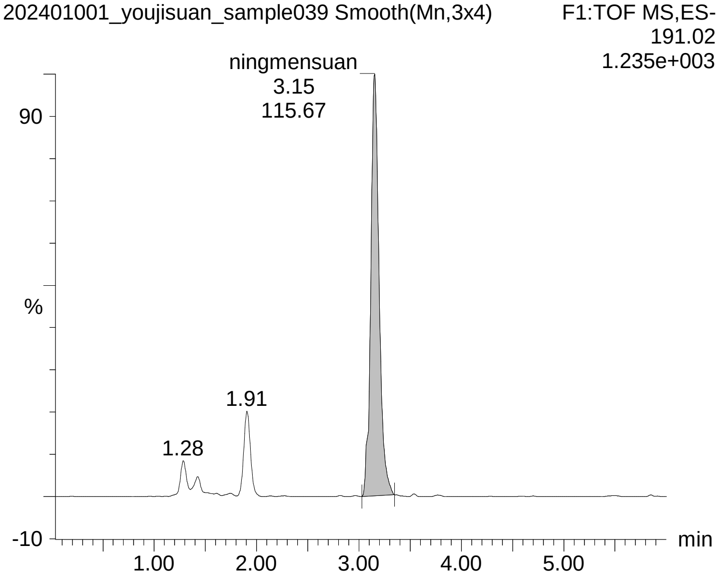

pinguosuan

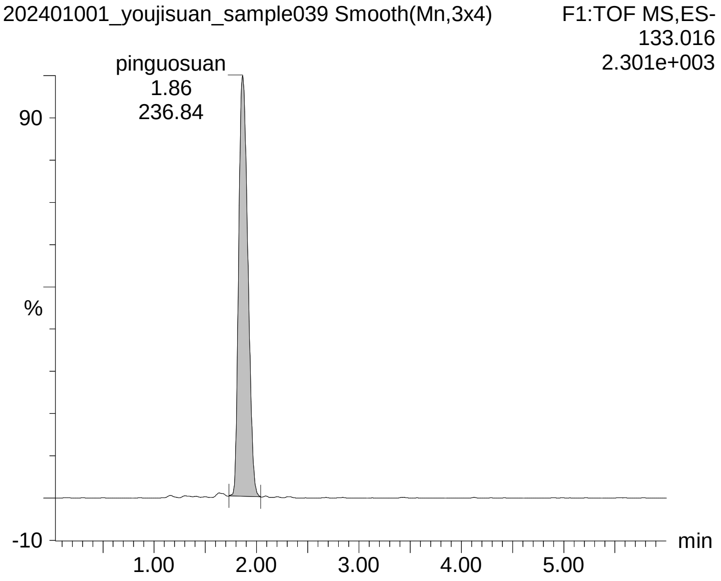

	#	Name	Sample Text	RT	Area	Std. Conc	Conc.
1	1	jiushisuan		1.50	1901.921		418.763443
2	2	ningmensuan		3.15	115.666		15.744705
3	3	pinguosuan		1.86	236.843		86.051478

project_wangzhonghua_BeiMu
Dataset: Untitled
Last Altered: Friday, October 11, 2024 15:42:35 China Standard Time
Printed: Friday, October 11, 2024 15:43:34 China Standard Time

Name: 202401001_youjisuan_sample040, Date: 01-Oct-2024, Time: 23:26:43, ID: , Description:

jiushisuan

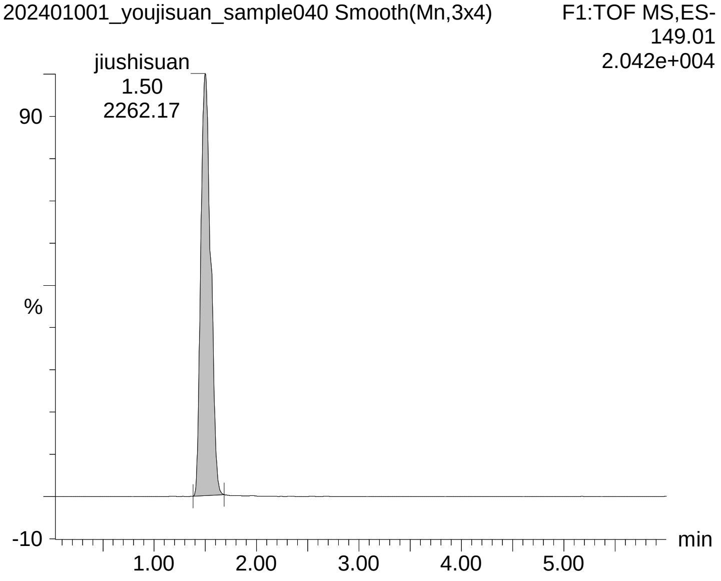

ningmensuan

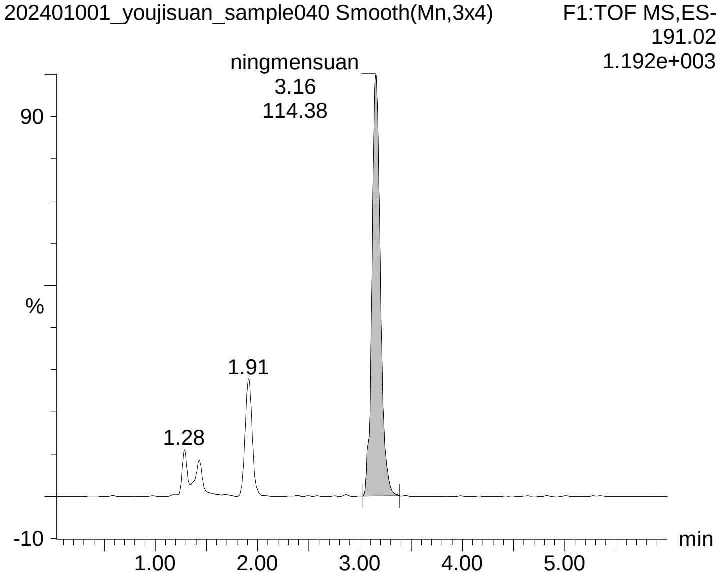

pinguosuan

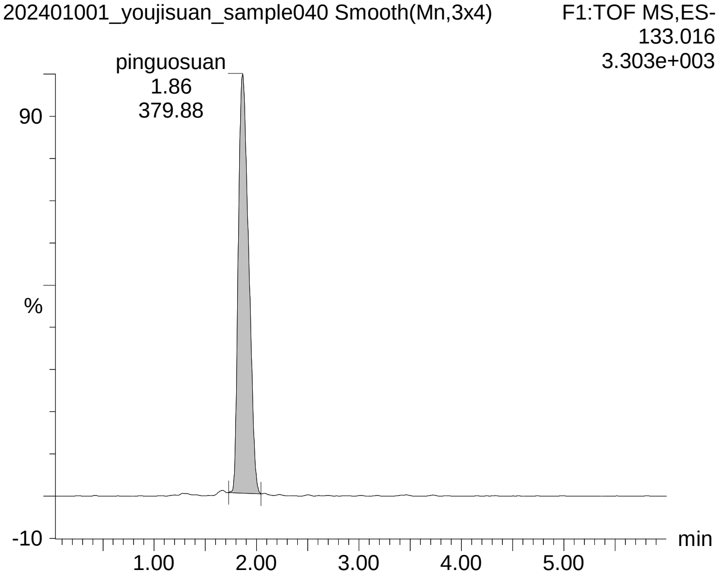

	#	Name	Sample Text	RT	Area	Std. Conc	Conc.
1	1	jiushisuan		1.50	2262.172		739.922776
2	2	ningmensuan		3.16	114.377		15.556845
3	3	pinguosuan		1.86	379.875		230.667634

project_wangzhonghua_BeiMu
Dataset: Untitled
Last Altered: Friday, October 11, 2024 15:42:35 China Standard Time
Printed: Friday, October 11, 2024 15:43:34 China Standard Time

Name: 202401001_youjisuan_sample041, Date: 01-Oct-2024, Time: 23:34:40, ID: , Description:

jiushisuan

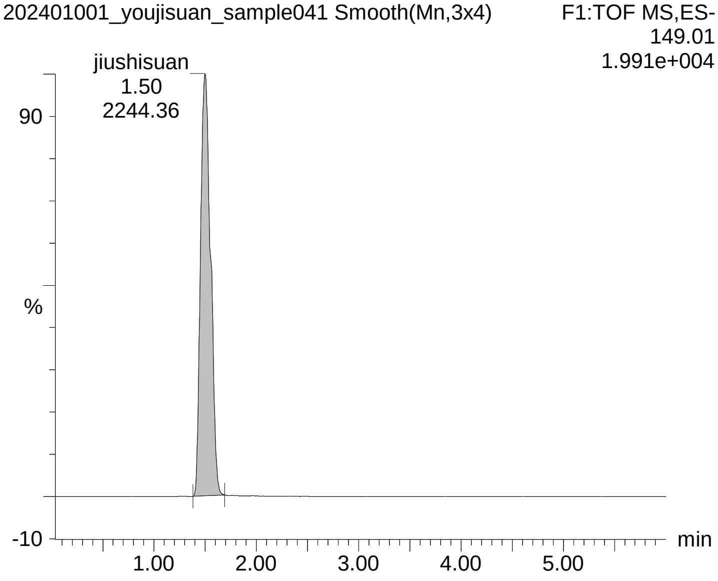

ningmensuan

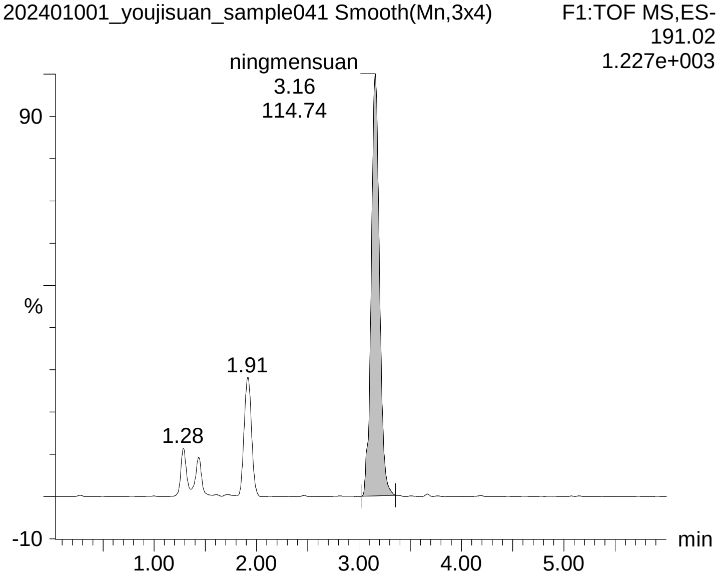

pinguosuan

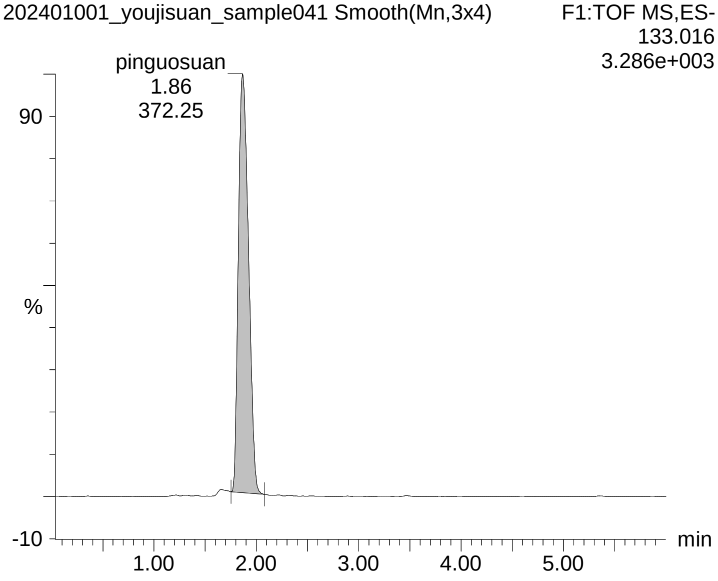

	#	Name	Sample Text	RT	Area	Std. Conc	Conc.
1	1	jiushisuan		1.50	2244.360		719.434498
2	2	ningmensuan		3.16	114.741		15.609895
3	3	pinguosuan		1.86	372.254		212.529230

project_wangzhonghua_BeiMu
Dataset: Untitled
Last Altered: Friday, October 11, 2024 15:42:35 China Standard Time
Printed: Friday, October 11, 2024 15:43:34 China Standard Time

Name: 202401001_youjisuan_sample042, Date: 01-Oct-2024, Time: 23:41:40, ID: , Description:

jiushisuan

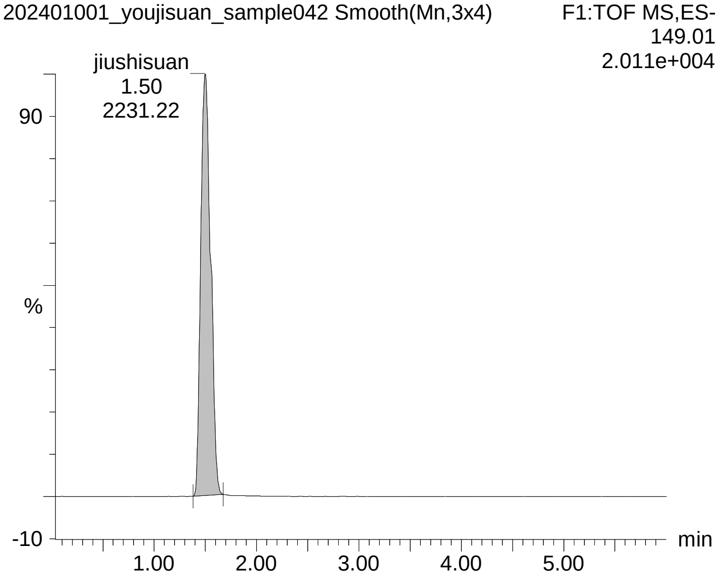

ningmensuan

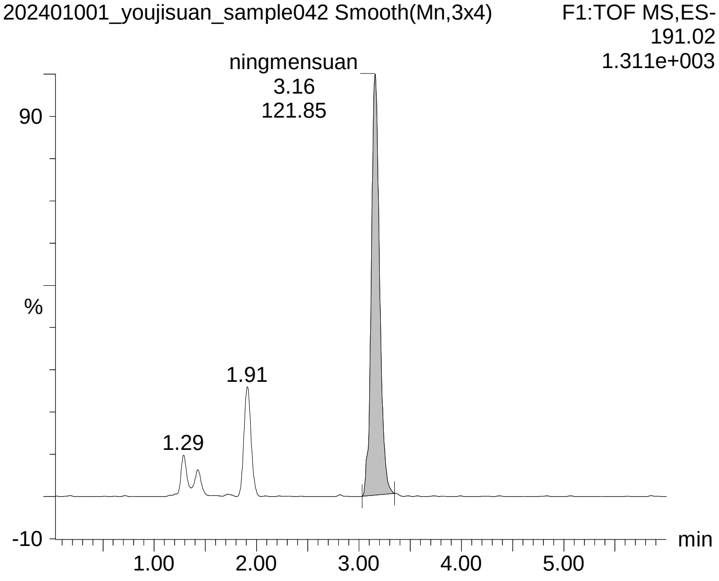

pinguosuan

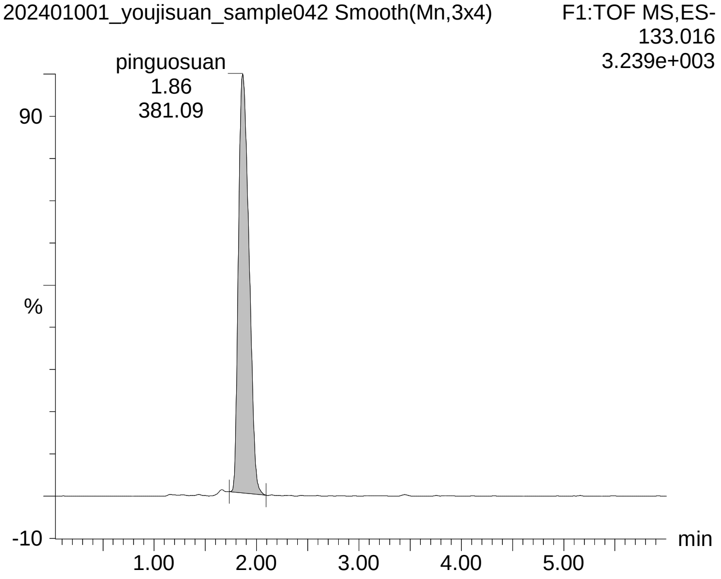

	#	Name	Sample Text	RT	Area	Std. Conc	Conc.
1	1	jiushisuan		1.50	2231.223		703.958035
2	2	ningmensuan		3.16	121.846		16.645382
3	3	pinguosuan		1.86	381.092		234.099458

project_wangzhonghua_BeiMu
Dataset: Untitled
Last Altered: Friday, October 11, 2024 15:42:35 China Standard Time
Printed: Friday, October 11, 2024 15:43:34 China Standard Time

Name: 202401001_youjisuan_sample043, Date: 01-Oct-2024, Time: 23:48:40, ID: , Description:

jiushisuan

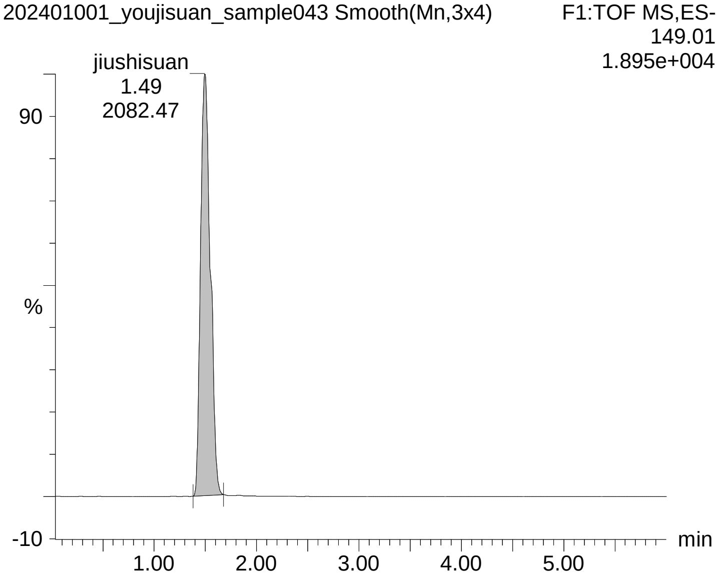

ningmensuan

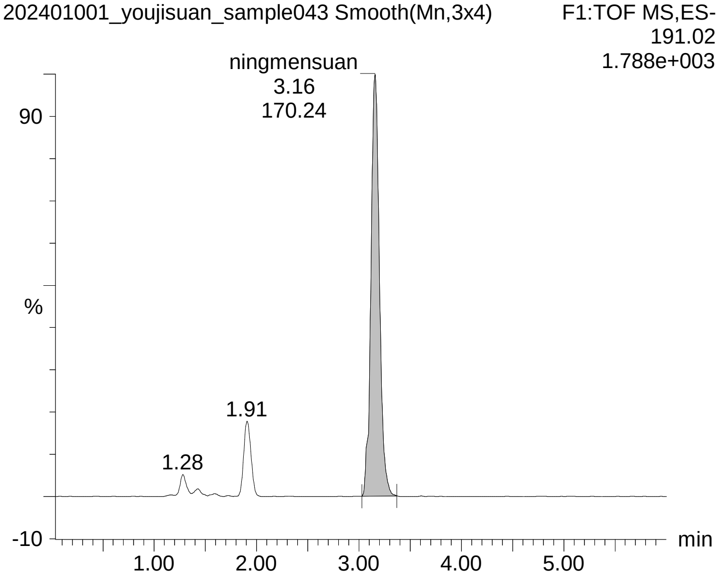

pinguosuan

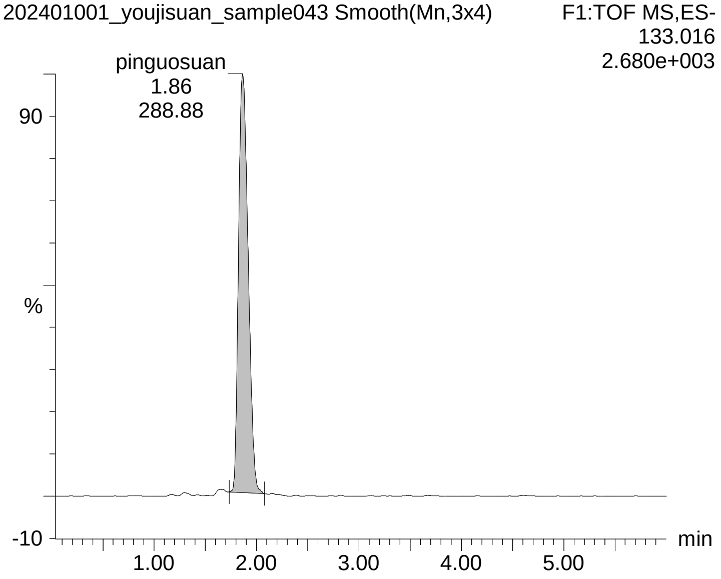

	#	Name	Sample Text	RT	Area	Std. Conc	Conc.
1	1	jiushisuan		1.49	2082.475		542.393567
2	2	ningmensuan		3.16	170.235		23.697624
3	3	pinguosuan		1.86	288.878		118.642078

Name: 202401001_youjisuan_sample044, Date: 01-Oct-2024, Time: 23:55:41, ID: , Description:

jiushisuan

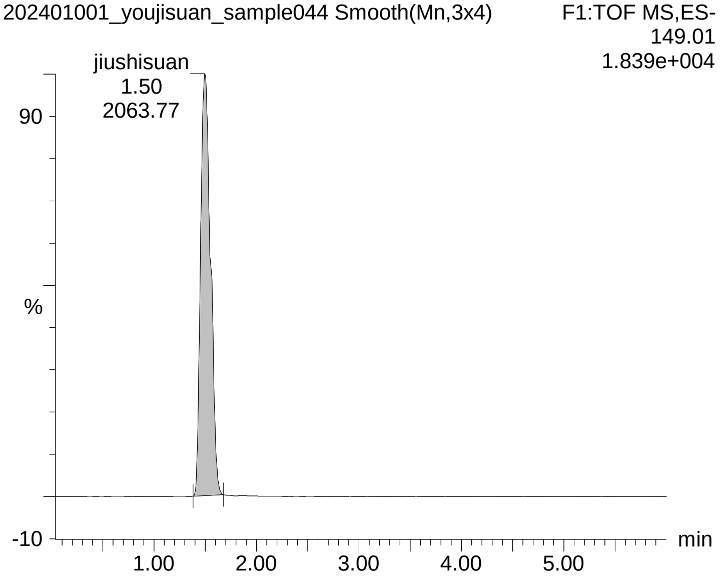

ningmensuan

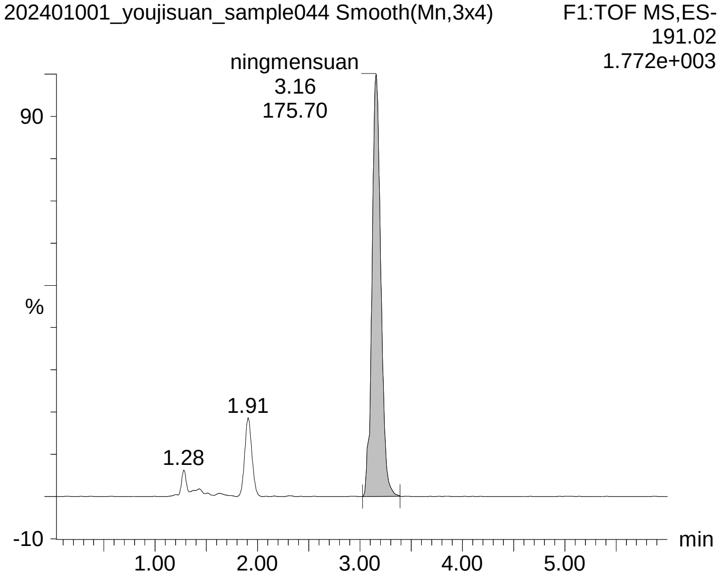

pinguosuan

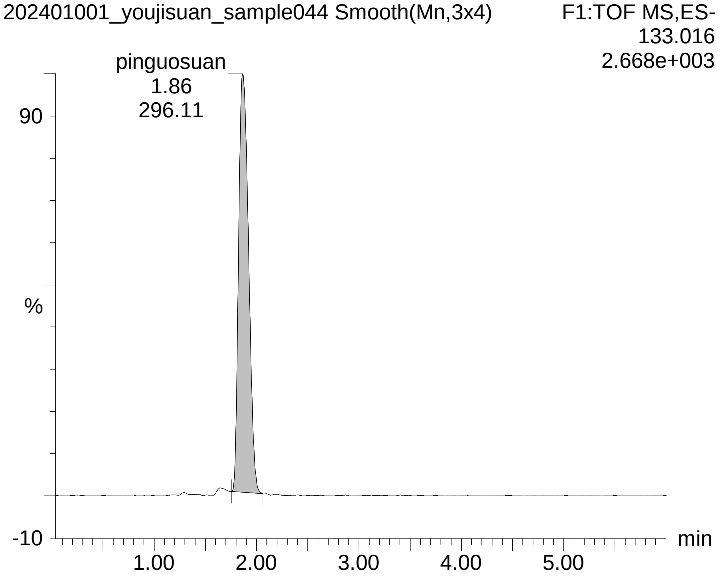

	#	Name	Sample Text	RT	Area	Std. Conc	Conc.
1	1	jiushisuan		1.50	2063.773		526.304788
2	2	ningmensuan		3.16	175.699		24.493951
3	3	pinguosuan		1.86	296.113		123.997713

project_wangzhonghua_BeiMu
Dataset: Untitled
Last Altered: Friday, October 11, 2024 15:42:35 China Standard Time
Printed: Friday, October 11, 2024 15:43:34 China Standard Time

Name: 202401001_youjisuan_sample045, Date: 02-Oct-2024, Time: 00:02:41, ID: , Description:

jiushisuan

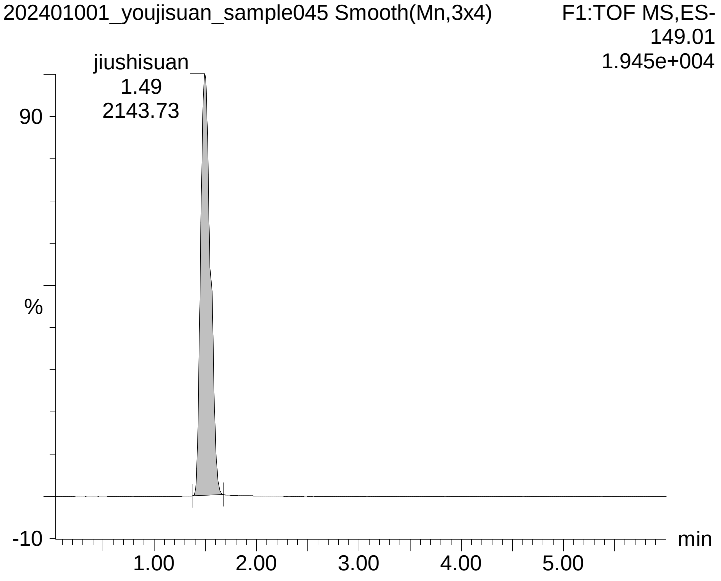

ningmensuan

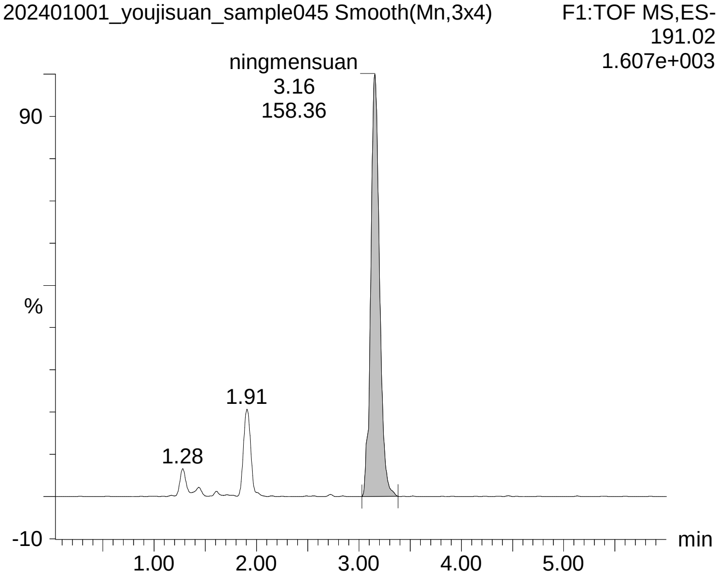

pinguosuan

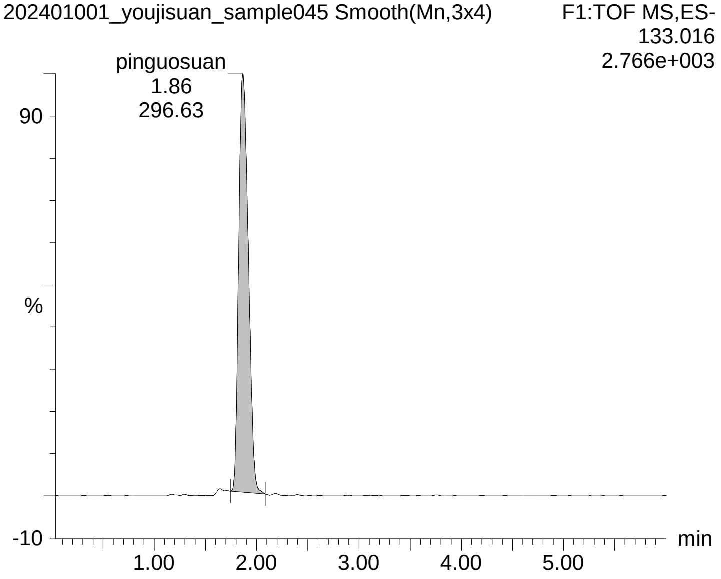

	#	Name	Sample Text	RT	Area	Std. Conc	Conc.
1	1	jiushisuan		1.49	2143.729		602.408575
2	2	ningmensuan		3.16	158.358		21.966663
3	3	pinguosuan		1.86	296.635		124.394417

Name: 202401001_youjisuan_sample046, Date: 02-Oct-2024, Time: 00:09:41, ID: , Description:

jiushisuan

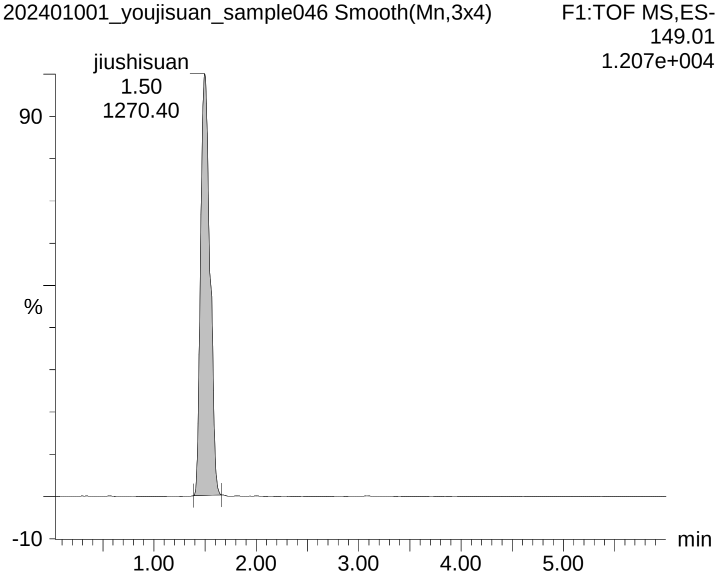

ningmensuan

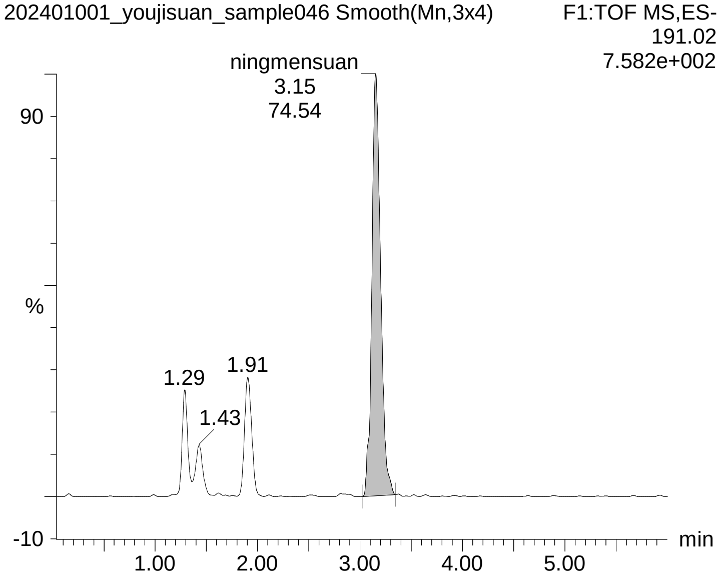

pinguosuan

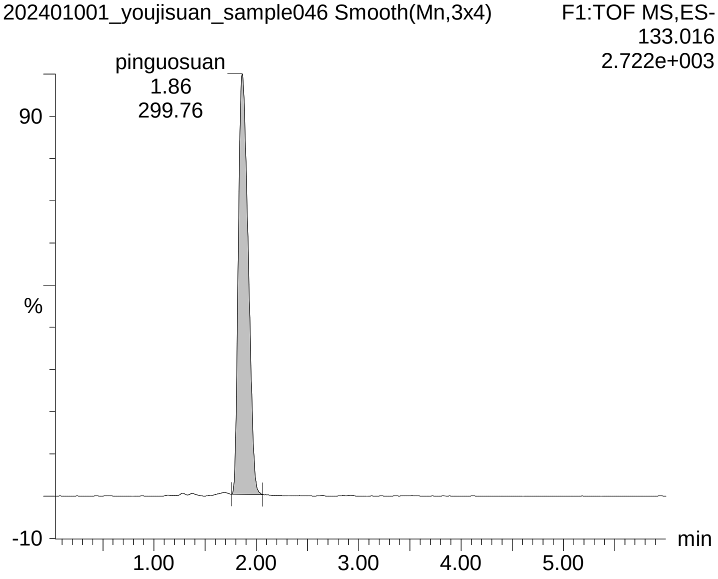

	#	Name	Sample Text	RT	Area	Std. Conc	Conc.
1	1	jiushisuan		1.50	1270.399		203.846438
2	2	ningmensuan		3.15	74.541		9.751123
3	3	pinguosuan		1.86	299.757		126.797818

Name: 202401001_youjisuan_sample047, Date: 02-Oct-2024, Time: 00:16:41, ID: , Description:

jiushisuan

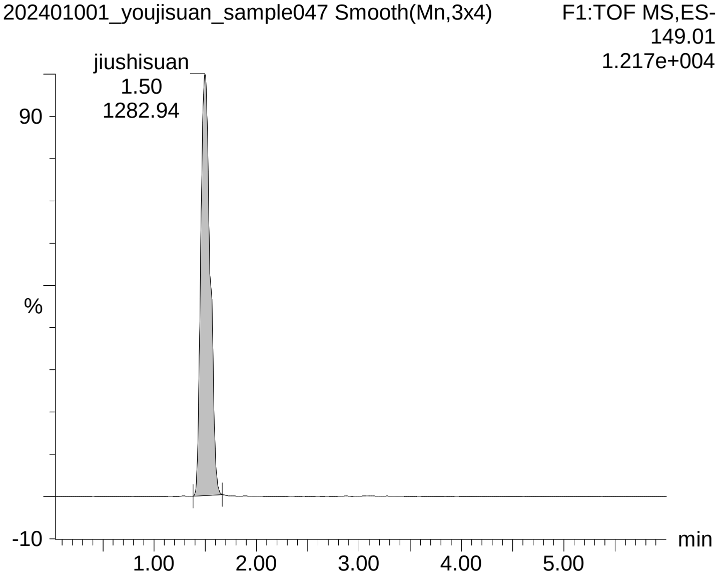

ningmensuan

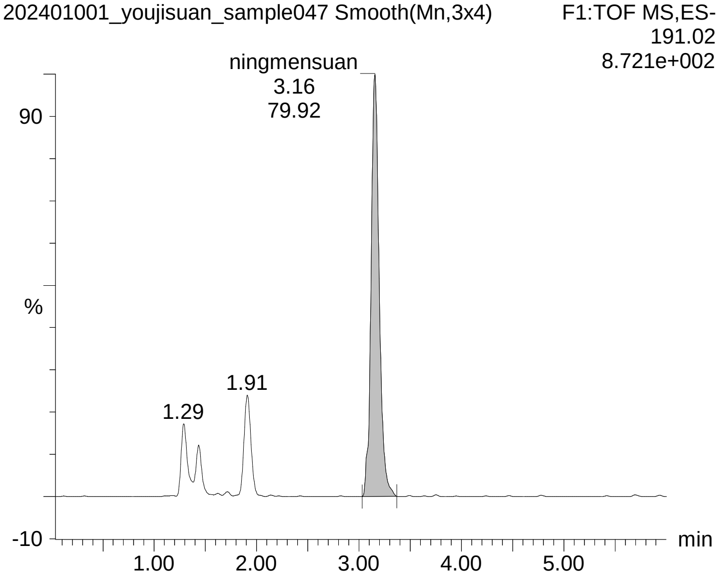

pinguosuan

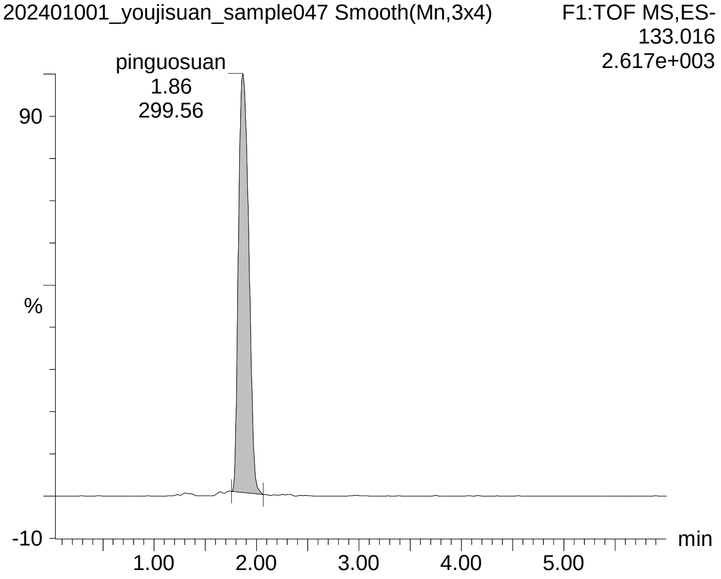

	#	Name	Sample Text	RT	Area	Std. Conc	Conc.
1	1	jiushisuan		1.50	1282.939		206.821217
2	2	ningmensuan		3.16	79.915		10.534333
3	3	pinguosuan		1.86	299.561		126.645350

project_wangzhonghua_BeiMu
Dataset: Untitled
Last Altered: Friday, October 11, 2024 15:42:35 China Standard Time
Printed: Friday, October 11, 2024 15:43:34 China Standard Time

Name: 202401001_youjisuan_sample048, Date: 02-Oct-2024, Time: 00:23:41, ID: , Description:

jiushisuan

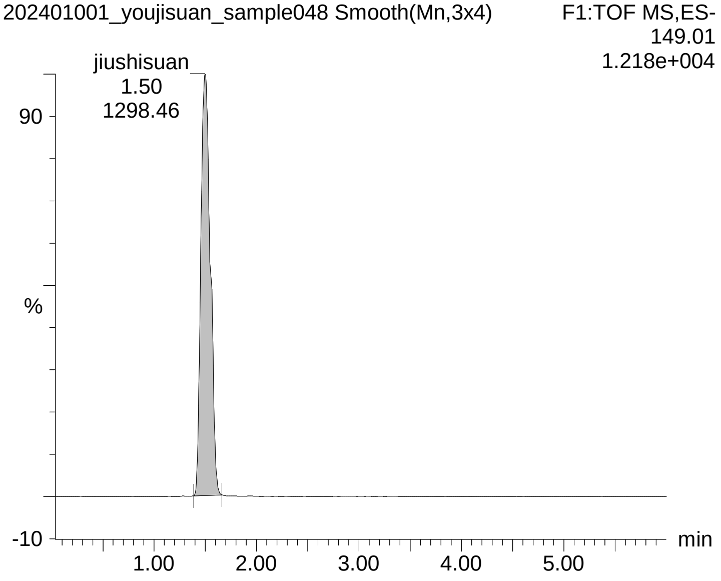

ningmensuan

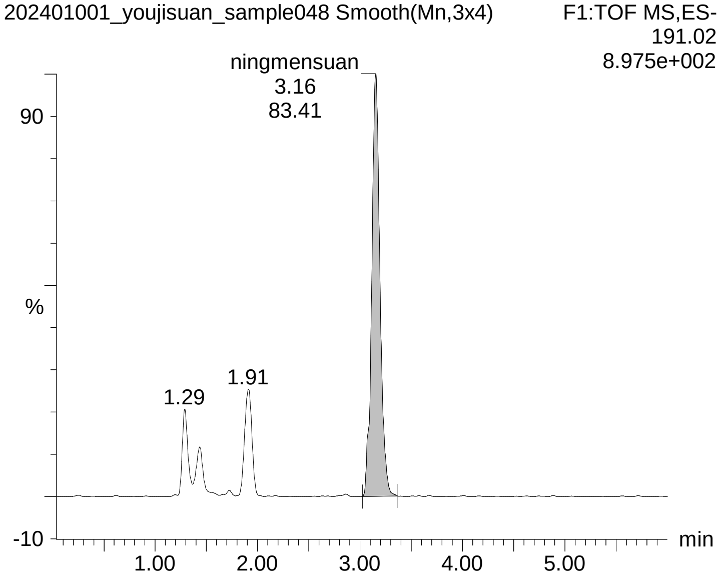

pinguosuan

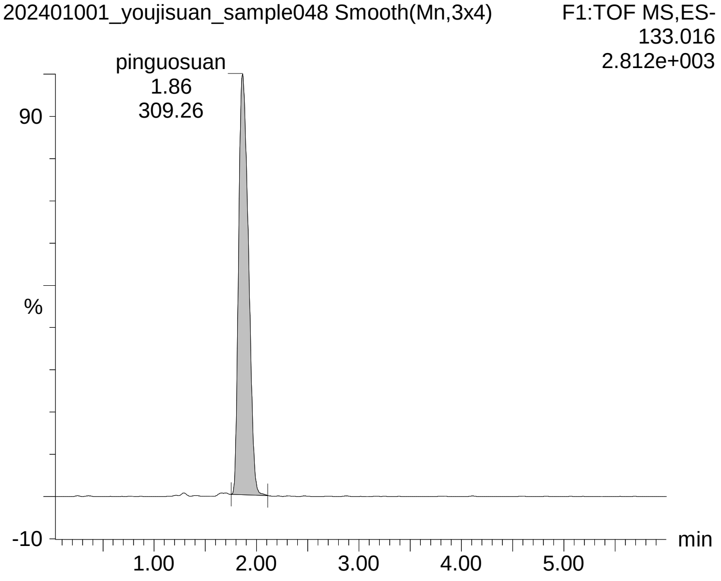

	#	Name	Sample Text	RT	Area	Std. Conc	Conc.
1	1	jiushisuan		1.50	1298.465		210.546110
2	2	ningmensuan		3.16	83.406		11.043113
3	3	pinguosuan		1.86	309.261		134.465835

project_wangzhonghua_BeiMu
Dataset: Untitled
Last Altered: Friday, October 11, 2024 15:42:35 China Standard Time
Printed: Friday, October 11, 2024 15:43:34 China Standard Time

Name: 202401001_youjisuan_sample049, Date: 02-Oct-2024, Time: 00:30:44, ID: , Description:

jiushisuan

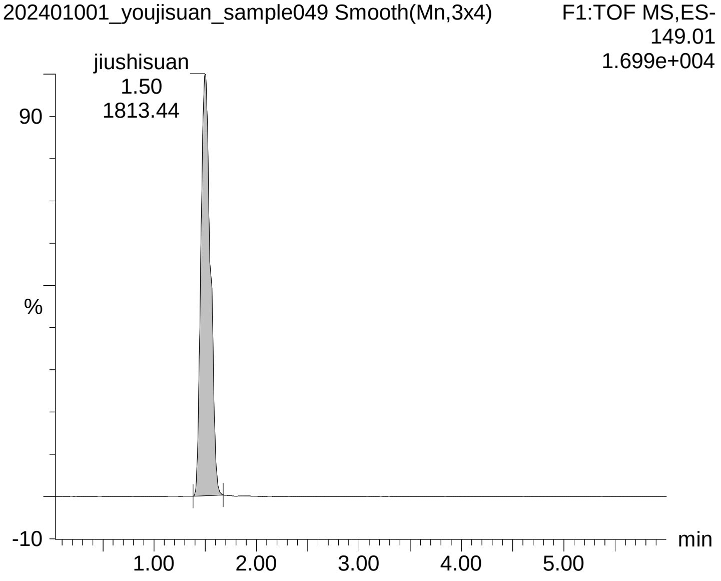

ningmensuan

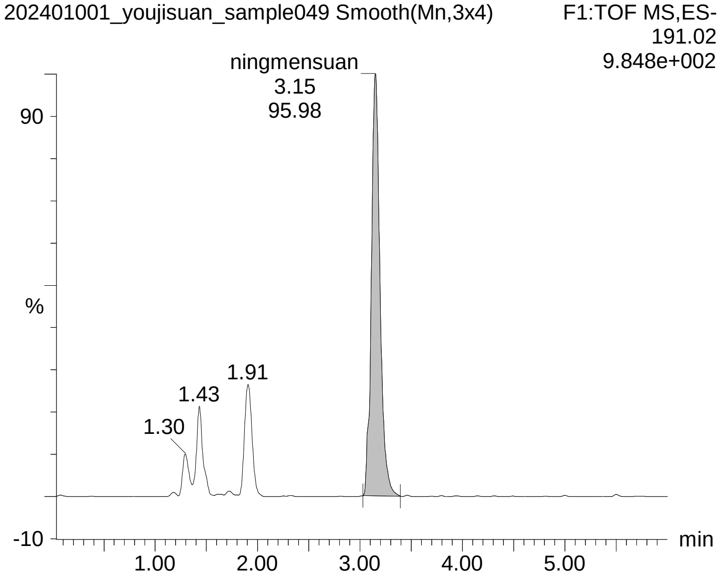

pinguosuan

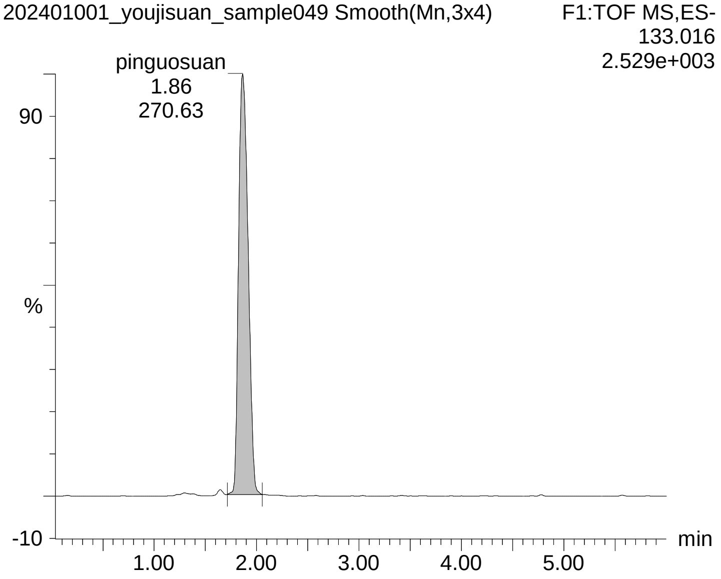

	#	Name	Sample Text	RT	Area	Std. Conc	Conc.
1	1	jiushisuan		1.50	1813.441		375.543330
2	2	ningmensuan		3.15	95.983		12.876093
3	3	pinguosuan		1.86	270.631		106.168293

Name: 202401001_youjisuan_sample050, Date: 02-Oct-2024, Time: 00:37:45, ID: , Description:

jiushisuan

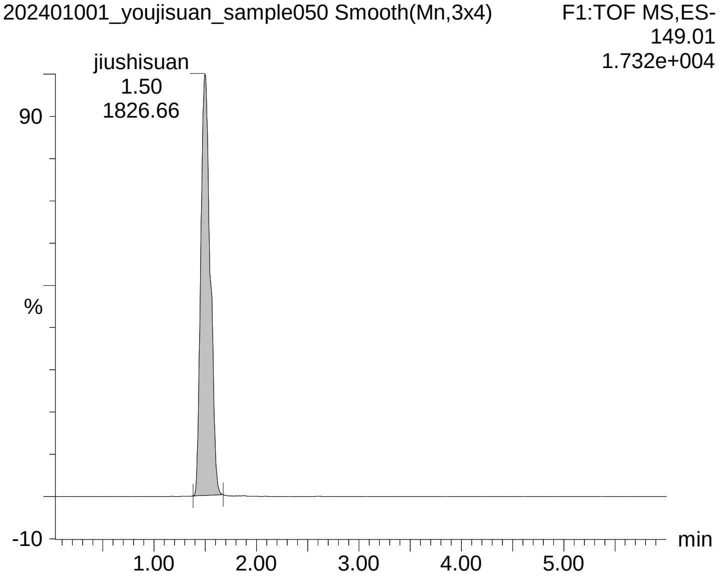

ningmensuan

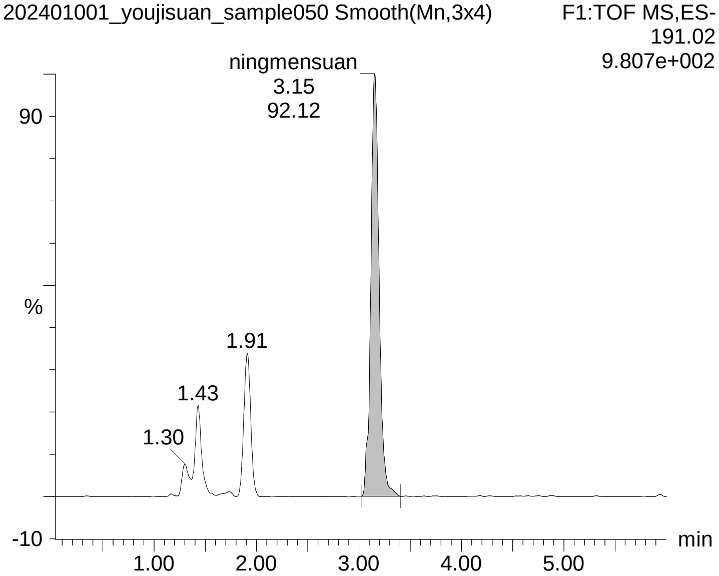

pinguosuan

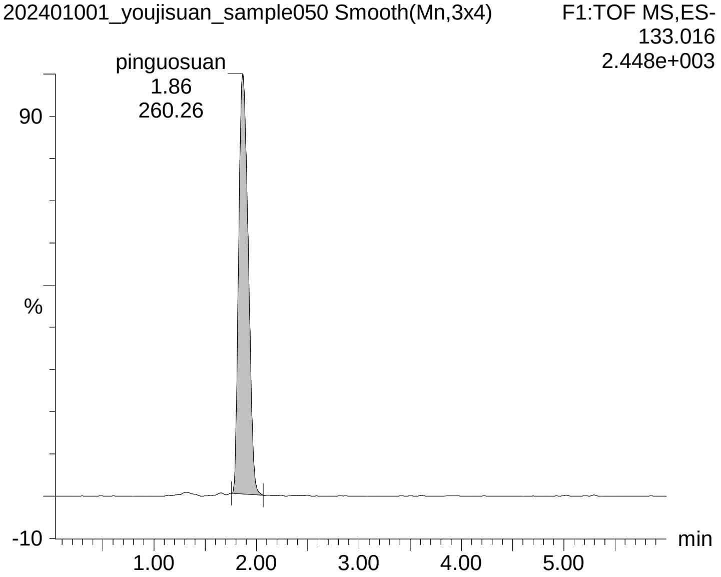

	#	Name	Sample Text	RT	Area	Std. Conc	Conc.
1	1	jiushisuan		1.50	1826.656		381.529045
2	2	ningmensuan		3.15	92.117		12.312660
3	3	pinguosuan		1.86	260.264		99.634616

project_wangzhonghua_BeiMu
Dataset: Untitled
Last Altered: Friday, October 11, 2024 15:42:35 China Standard Time
Printed: Friday, October 11, 2024 15:43:34 China Standard Time

Name: 202401001_youjisuan_sample051, Date: 02-Oct-2024, Time: 00:44:45, ID: , Description:

jiushisuan

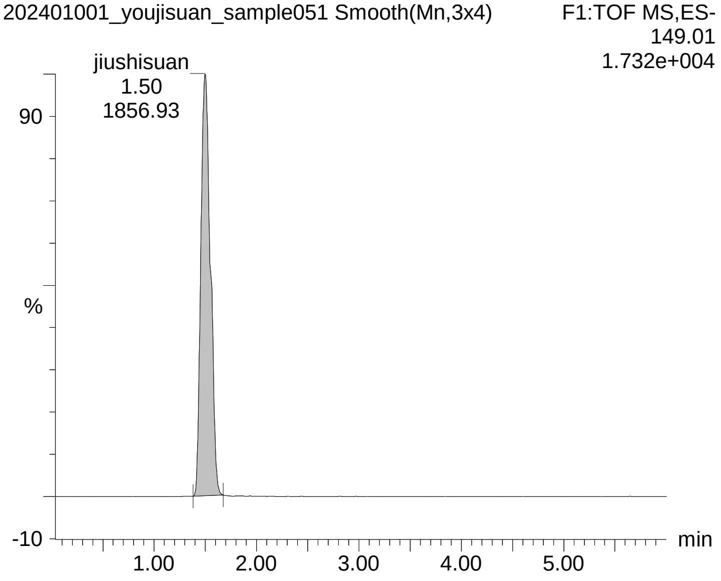

ningmensuan

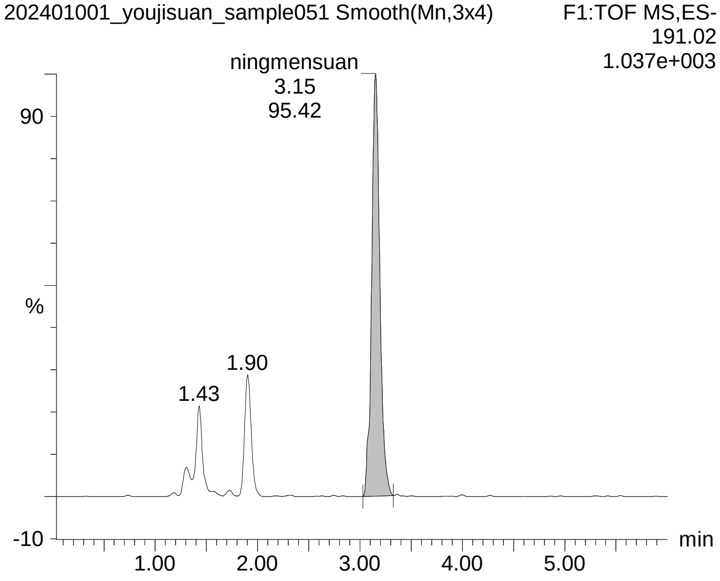

pinguosuan

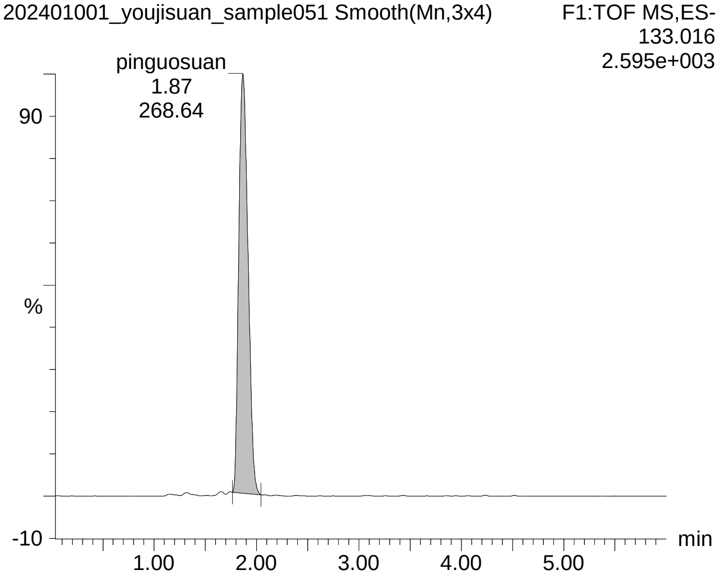

	#	Name	Sample Text	RT	Area	Std. Conc	Conc.
1	1	jiushisuan		1.50	1856.928		395.821695
2	2	ningmensuan		3.15	95.425		12.794769
3	3	pinguosuan		1.87	268.636		104.882679

project_wangzhonghua_BeiMu
Dataset: Untitled
Last Altered: Friday, October 11, 2024 15:45:23 China Standard Time
Printed: Friday, October 11, 2024 15:46:51 China Standard Time

Method: F:\data\Wu_yueyan.PRO\MethDB\20241011_ organic acid .mdb 11 Oct 2024 13:22:01
Calibration: F:\data\zhanghuien.PRO\CurveDB\20241011_organic acid003.cdb 11 Oct 2024 15:39:56

Compound name: jiushisuan
Coefficient of Determination: R^2 = 0.994685
Calibration curve: 4.94514e-006 * x^3 + -0.0101268 * x^2 + 7.74871 * x + 69.7696
Response type: External Std, Area
Curve type: 3rd Order, Origin: Include, Weighting: Null, Axis trans: None

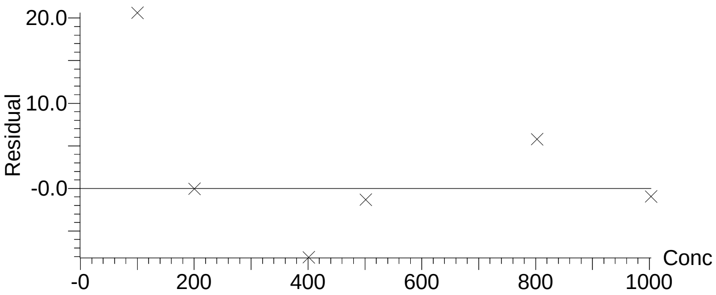

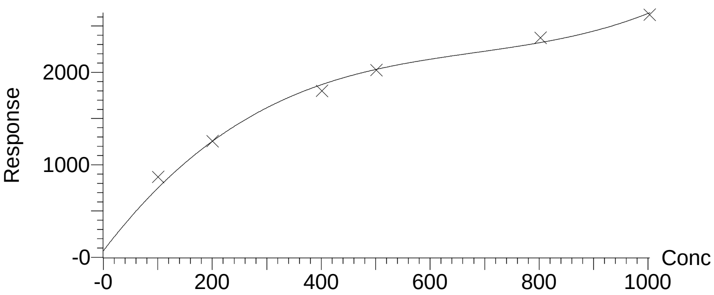

Compound name: ningmensuan
Correlation coefficient: r = 0.998196, r^2 = 0.996396
Calibration curve: 6.86151 * x + 7.63362
Response type: External Std, Area
Curve type: Linear, Origin: Include, Weighting: Null, Axis trans: None

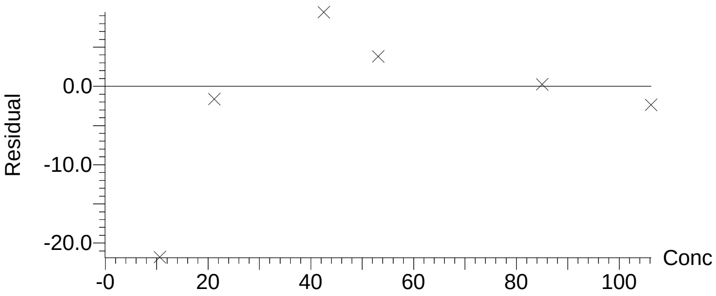

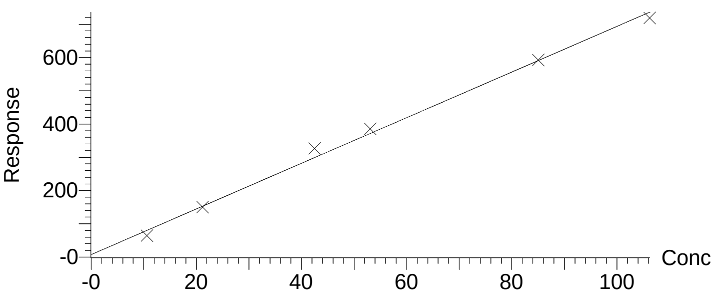

Compound name: pinguosuan
Coefficient of Determination: R^2 = 0.984709
Calibration curve: 9.85928e-006 * x^3 + -0.00971593 * x^2 + 3.27297 * x + 20.8616
Response type: External Std, Area
Curve type: 3rd Order, Origin: Include, Weighting: Null, Axis trans: None

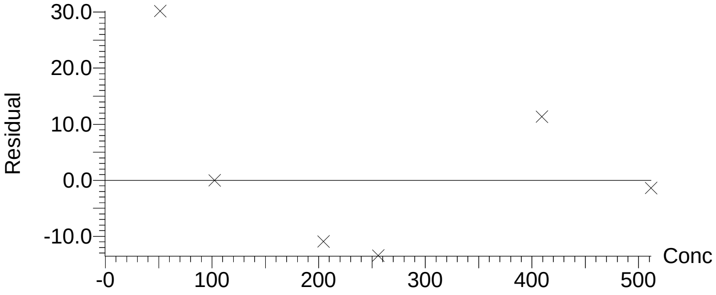

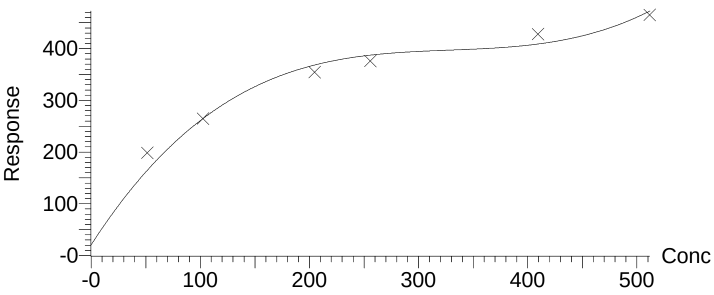

Compound name: huposaun
Coefficient of Determination: R^2 = 0.999608
Calibration curve: 3.18016e-005 * x^3 + -0.0146291 * x^2 + 2.726 * x + 0.728245
Response type: External Std, Area
Curve type: 3rd Order, Origin: Include, Weighting: Null, Axis trans: None

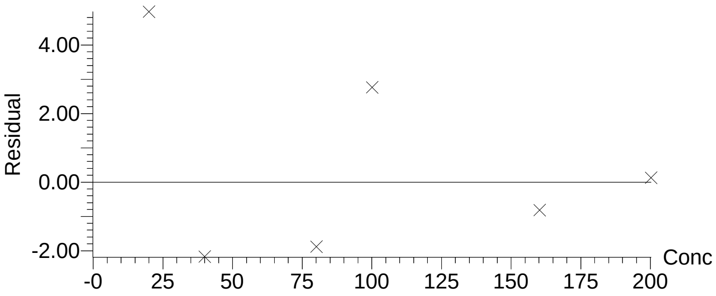

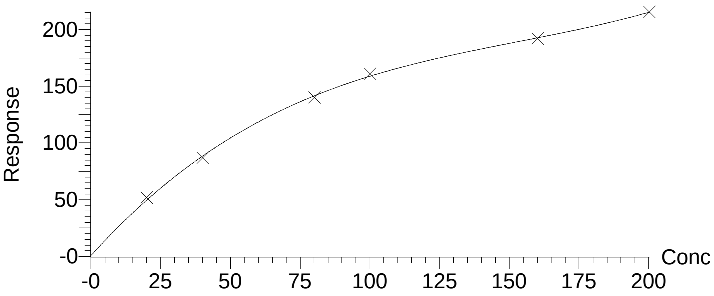

project_wangzhonghua_BeiMu
Dataset: Untitled
Last Altered: Friday, October 11, 2024 15:45:23 China Standard Time
Printed: Friday, October 11, 2024 15:46:51 China Standard Time

Method: F:\data\Wu_yueyan.PRO\MethDB\20241011_ organic acid .mdb 11 Oct 2024 13:22:01
Calibration: F:\data\zhanghuien.PRO\CurveDB\20241011_organic acid003.cdb 11 Oct 2024 15:39:56

Name: 202401001_youjisuan_sample052, Date: 02-Oct-2024, Time: 00:52:45, ID: , Description:

jiushisuan

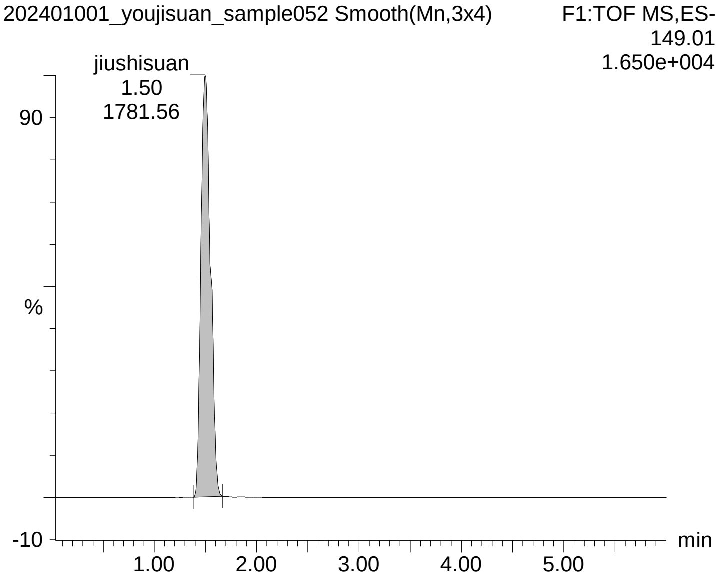

ningmensuan

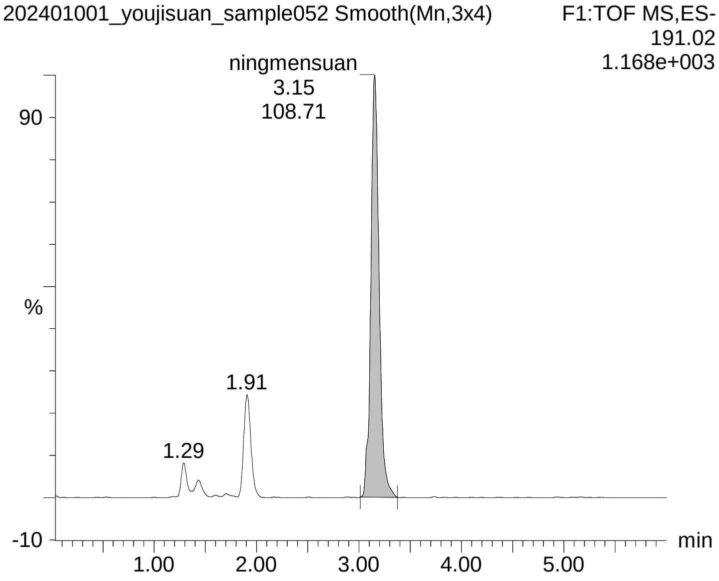

pinguosuan

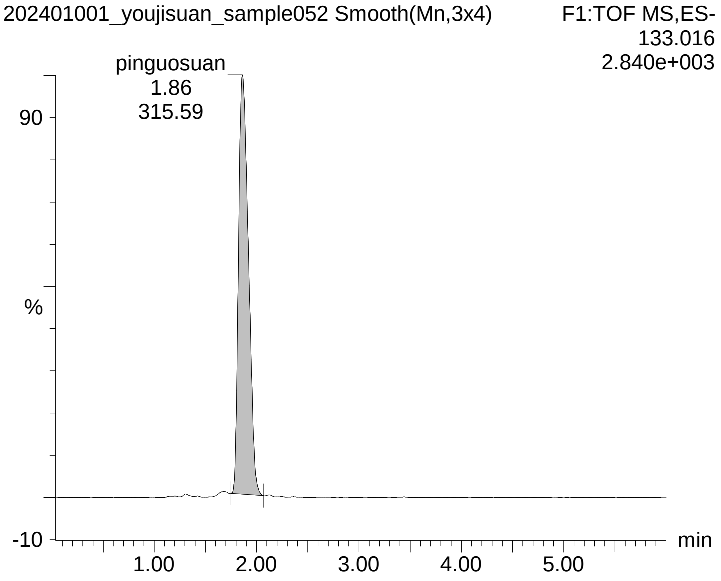

	#	Name	Sample Text	RT	Area	Std. Conc	Conc.
1	1	jiushisuan		1.50	1781.564		361.677516
2	2	ningmensuan		3.15	108.713		14.731371
3	3	pinguosuan		1.86	315.588		139.901653

Name: 202401001_youjisuan_sample053, Date: 02-Oct-2024, Time: 00:59:42, ID: , Description:

jiushisuan

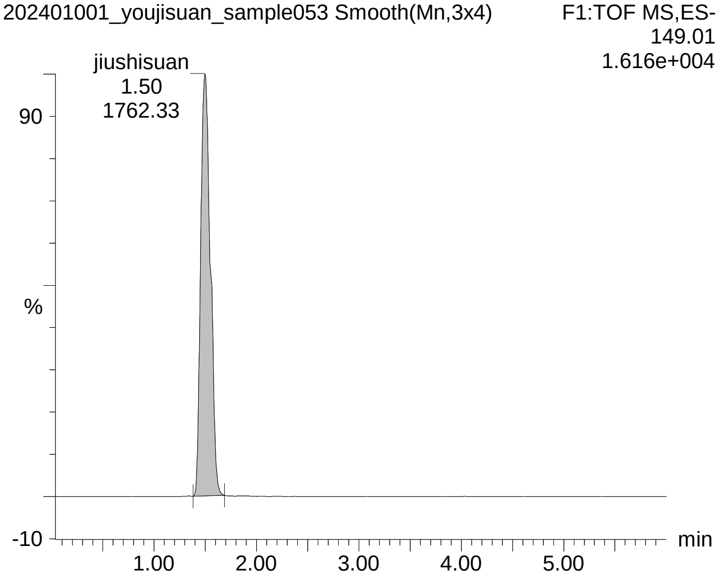

ningmensuan

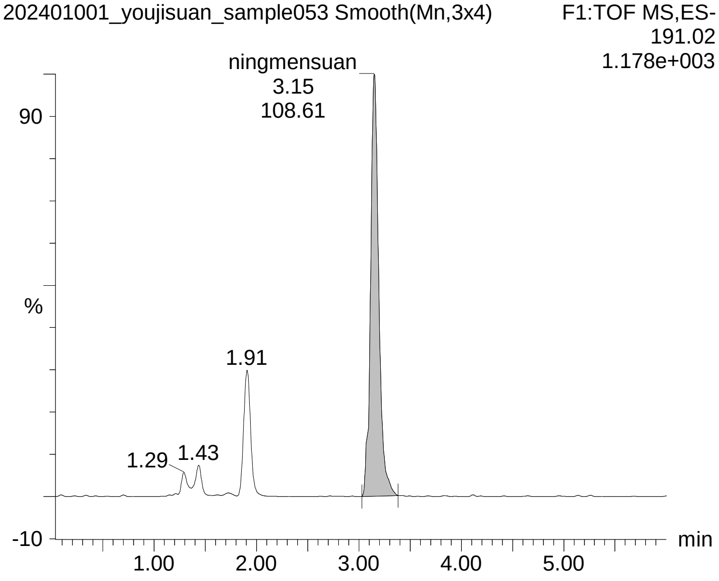

pinguosuan

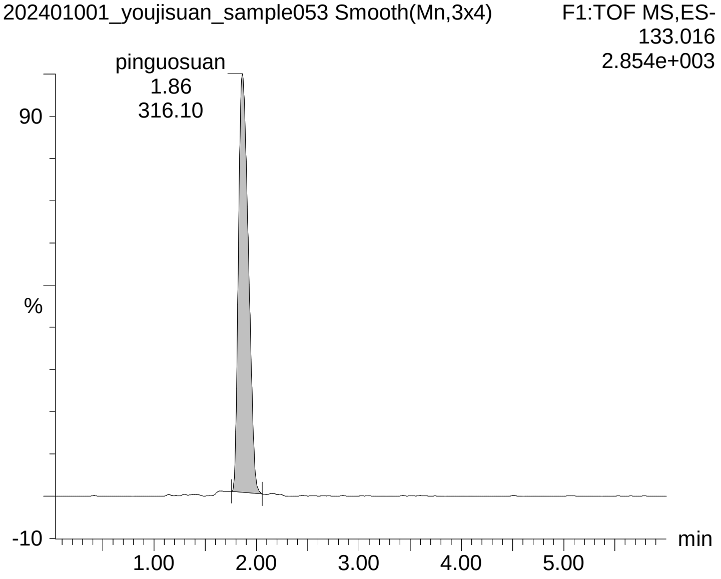

	#	Name	Sample Text	RT	Area	Std. Conc	Conc.
1	1	jiushisuan		1.50	1762.331		353.671984
2	2	ningmensuan		3.15	108.605		14.715631
3	3	pinguosuan		1.86	316.101		140.355311

project_wangzhonghua_BeiMu
Dataset: Untitled
Last Altered: Friday, October 11, 2024 15:45:23 China Standard Time
Printed: Friday, October 11, 2024 15:46:51 China Standard Time

Name: 202401001_youjisuan_sample054, Date: 02-Oct-2024, Time: 01:06:42, ID: , Description:

jiushisuan

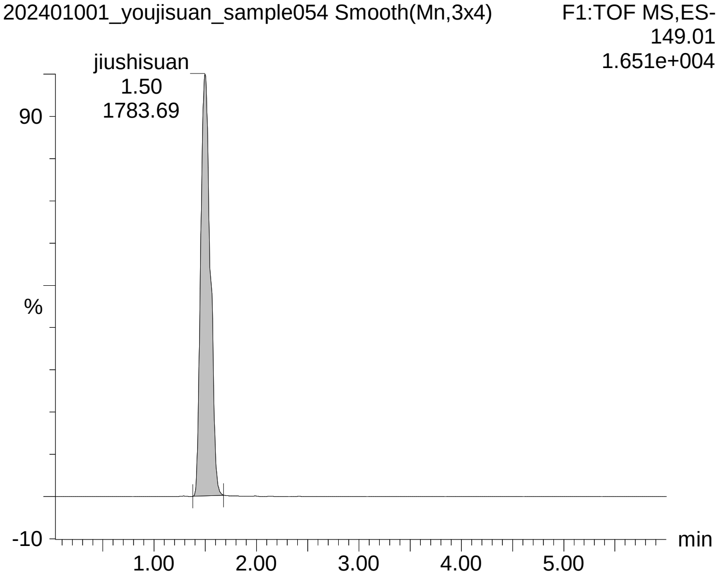

ningmensuan

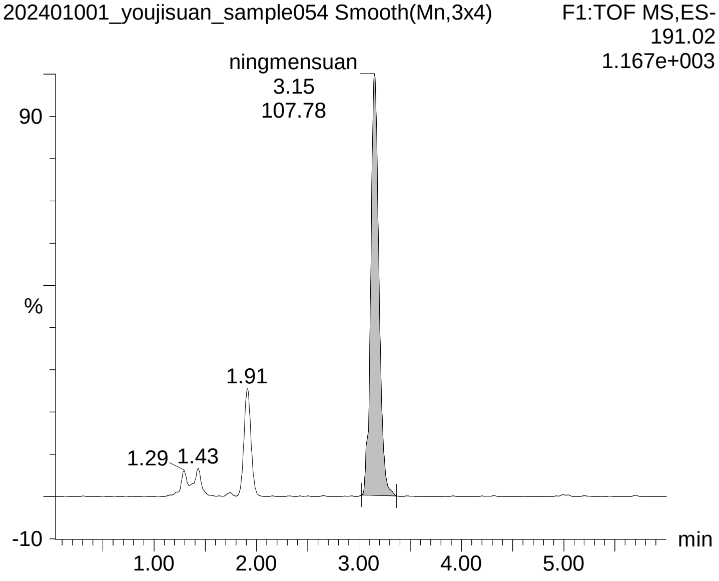

pinguosuan

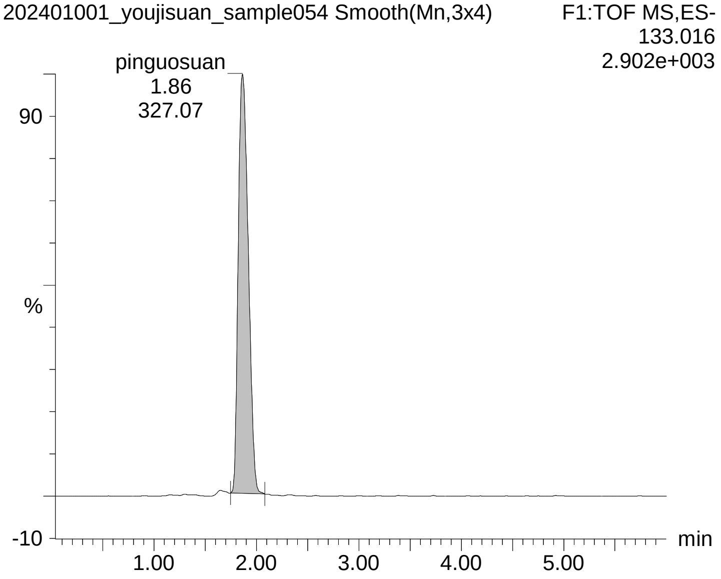

	#	Name	Sample Text	RT	Area	Std. Conc	Conc.
1	1	jiushisuan		1.50	1783.691		362.578885
2	2	ningmensuan		3.15	107.777		14.594957
3	3	pinguosuan		1.86	327.071		150.584811

Name: 202401001_youjisuan_sample055, Date: 02-Oct-2024, Time: 01:13:43, ID: , Description:

jiushisuan

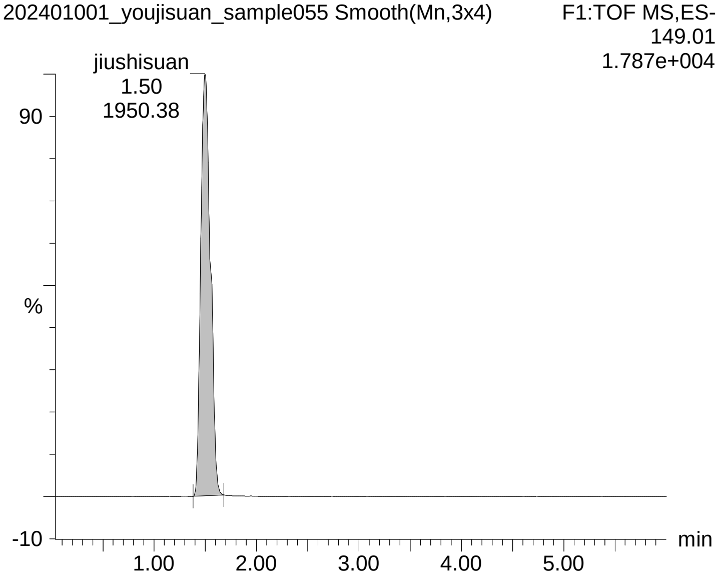

ningmensuan

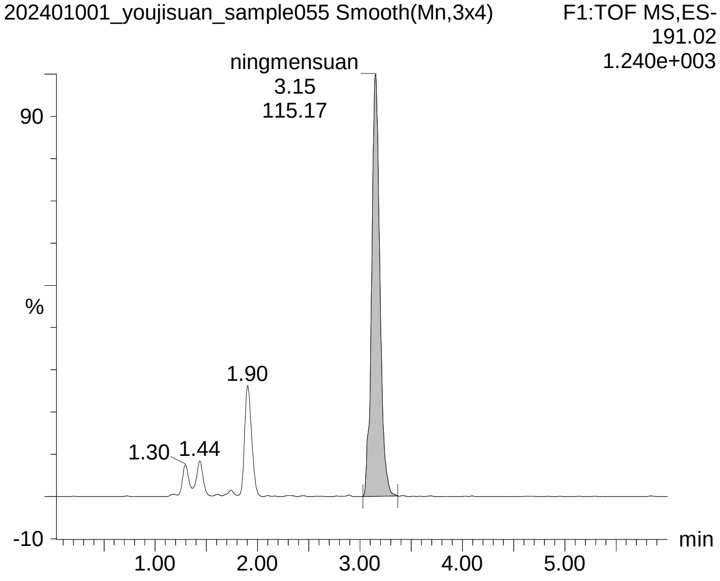

pinguosuan

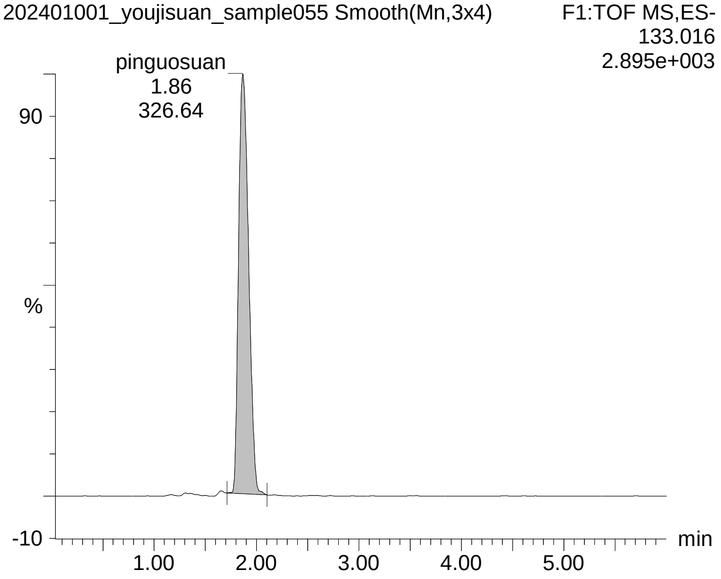

	#	Name	Sample Text	RT	Area	Std. Conc	Conc.
1	1	jiushisuan		1.50	1950.377		446.216529
2	2	ningmensuan		3.15	115.170		15.672418
3	3	pinguosuan		1.86	326.642		150.164117

Name: 202401001_youjisuan_sample056, Date: 02-Oct-2024, Time: 01:20:44, ID: , Description:

jiushisuan

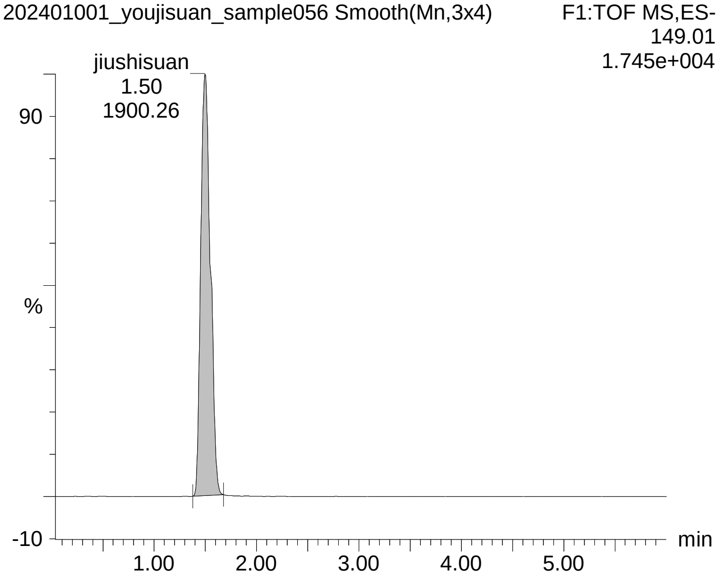

ningmensuan

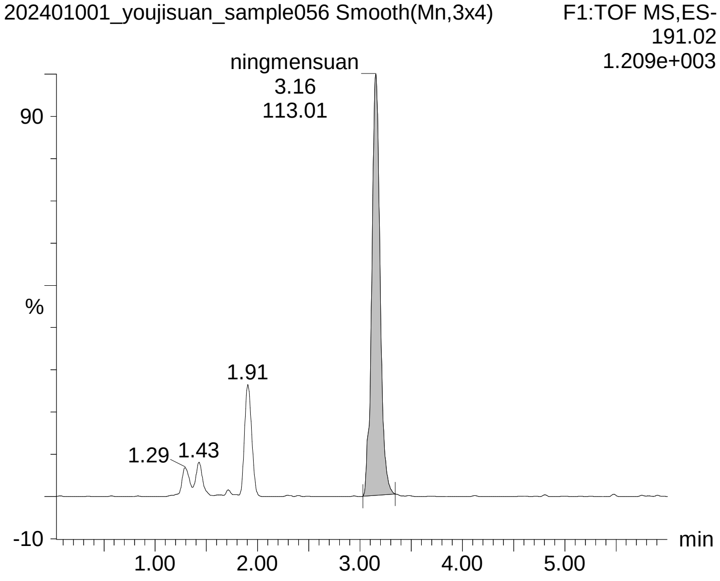

pinguosuan

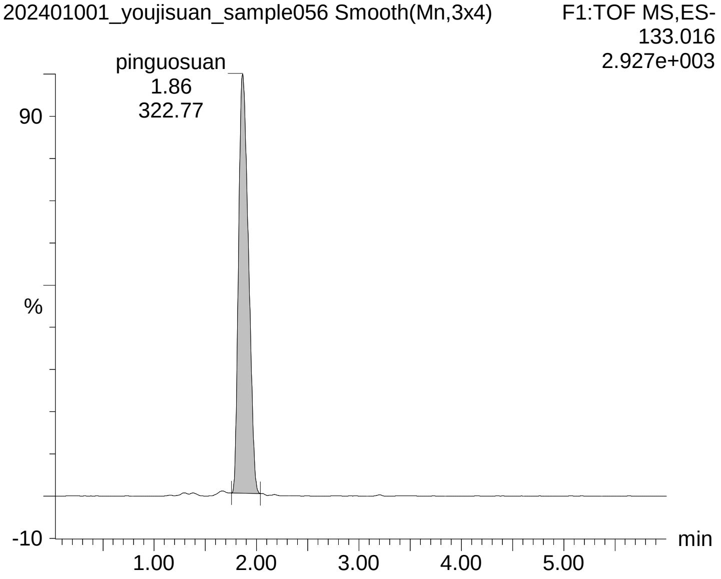

	#	Name	Sample Text	RT	Area	Std. Conc	Conc.
1	1	jiushisuan		1.50	1900.263		417.877878
2	2	ningmensuan		3.16	113.010		15.357618
3	3	pinguosuan		1.86	322.769		146.446187

Name: 202401001_youjisuan_sample057, Date: 02-Oct-2024, Time: 01:27:45, ID: , Description:

jiushisuan

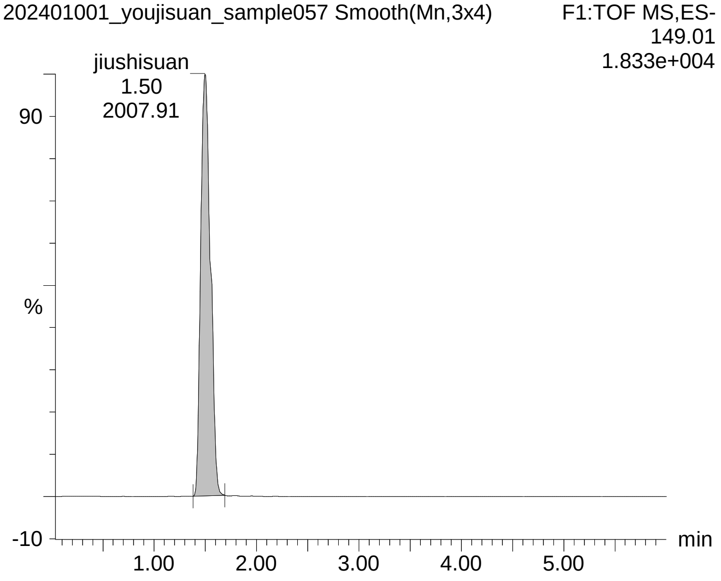

ningmensuan

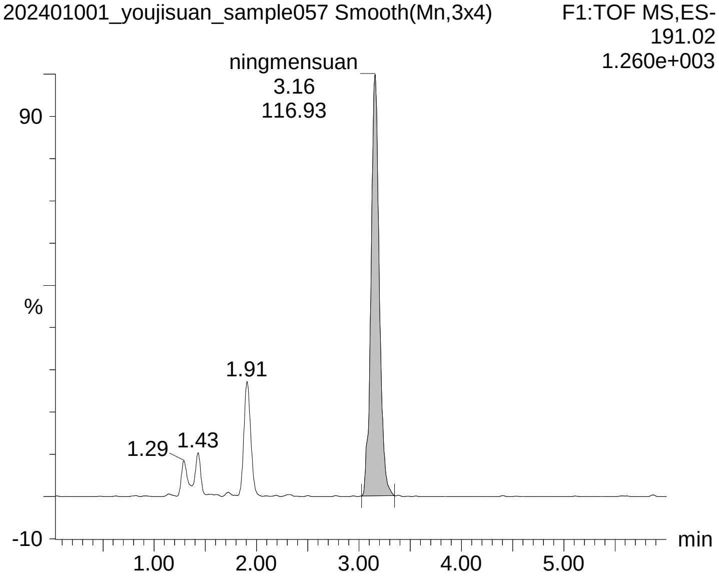

pinguosuan

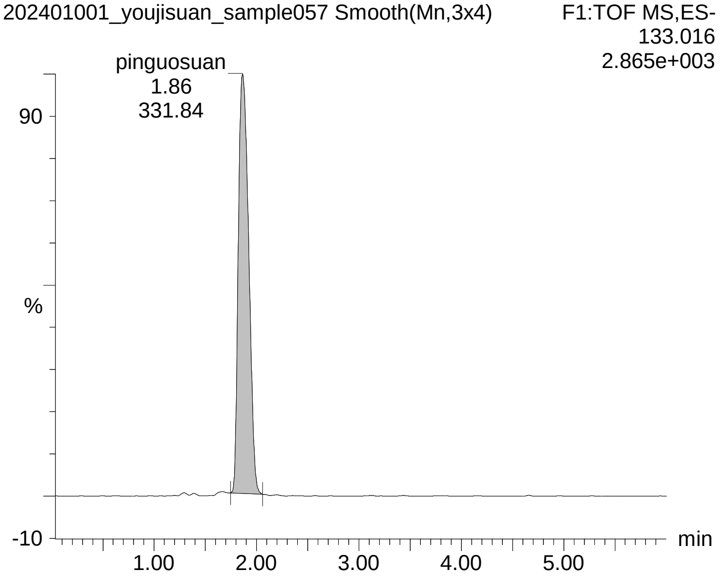

	#	Name	Sample Text	RT	Area	Std. Conc	Conc.
1	1	jiushisuan		1.50	2007.914		483.554189
2	2	ningmensuan		3.16	116.934		15.929504
3	3	pinguosuan		1.86	331.843		155.393080

project_wangzhonghua_BeiMu
Dataset: Untitled
Last Altered: Friday, October 11, 2024 15:45:23 China Standard Time
Printed: Friday, October 11, 2024 15:46:51 China Standard Time

Name: 202401001_youjisuan_sample058, Date: 02-Oct-2024, Time: 01:34:45, ID: , Description:

jiushisuan

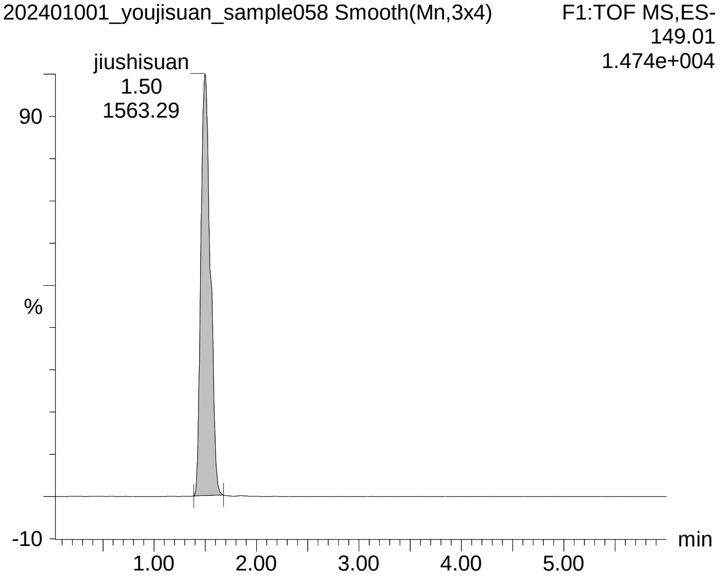

ningmensuan

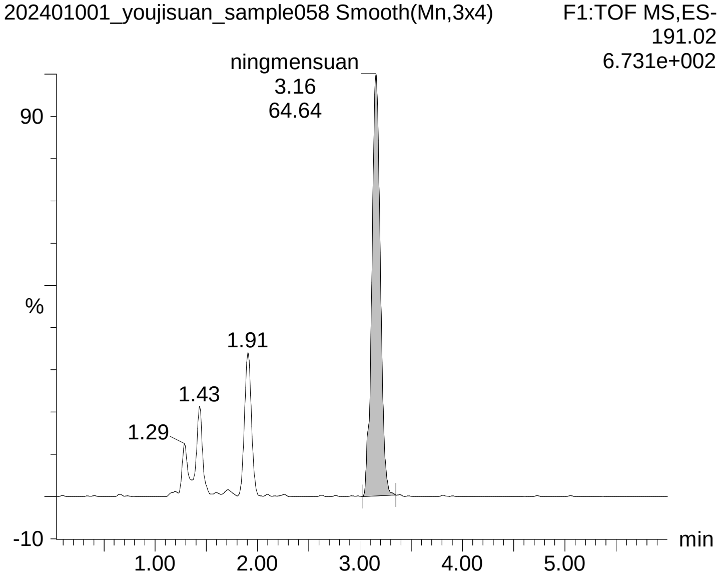

pinguosuan

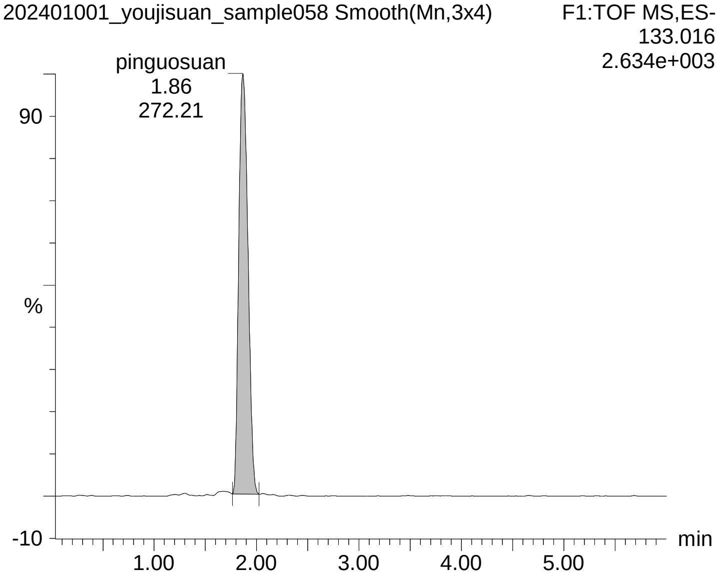

	#	Name	Sample Text	RT	Area	Std. Conc	Conc.
1	1	jiushisuan		1.50	1563.292		282.876810
2	2	ningmensuan		3.16	64.642		8.308436
3	3	pinguosuan		1.86	272.208		107.194599

Name: 202401001_youjisuan_sample059, Date: 02-Oct-2024, Time: 01:41:46, ID: , Description:

jiushisuan

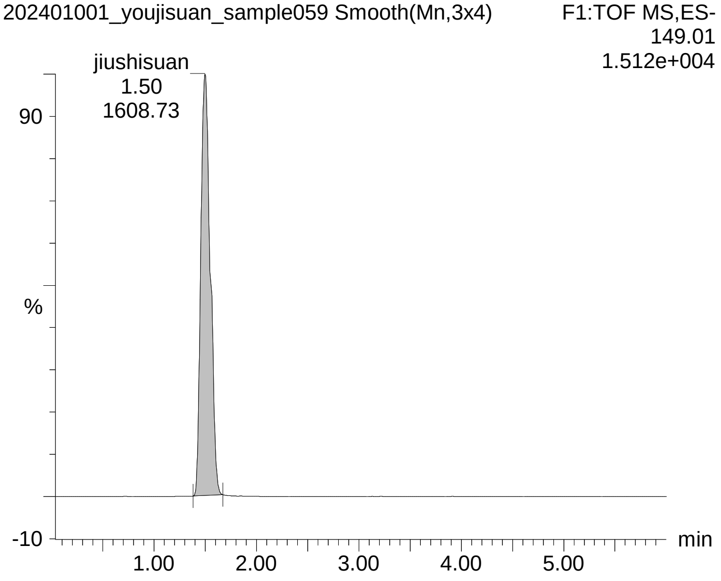

ningmensuan

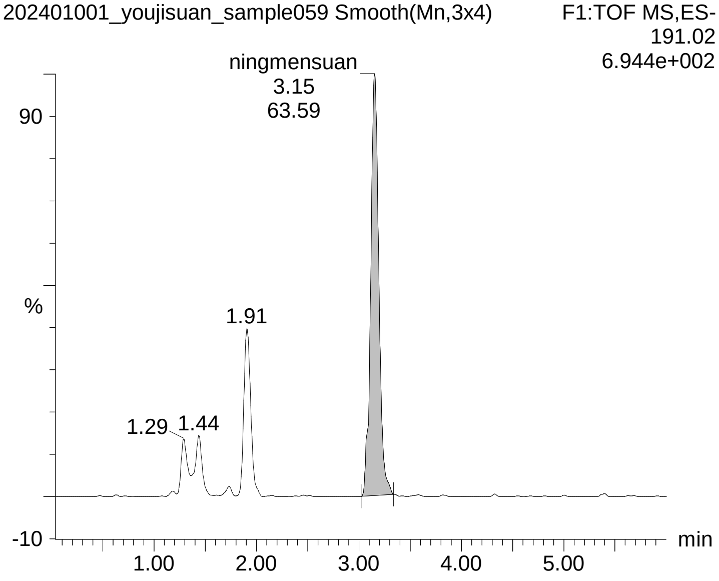

pinguosuan

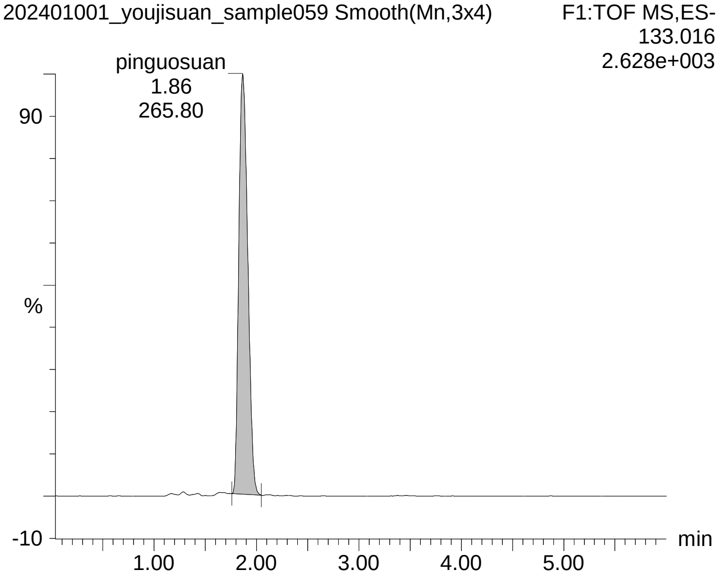

	#	Name	Sample Text	RT	Area	Std. Conc	Conc.
1	1	jiushisuan		1.50	1608.733		297.435461
2	2	ningmensuan		3.15	63.589		8.154971
3	3	pinguosuan		1.86	265.801		103.079510

Name: 202401001_youjisuan_sample060, Date: 02-Oct-2024, Time: 01:48:46, ID: , Description:

jiushisuan

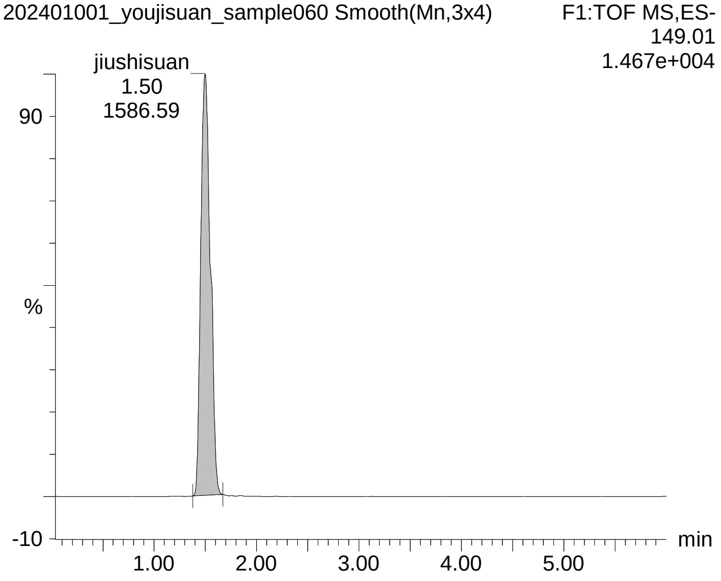

ningmensuan

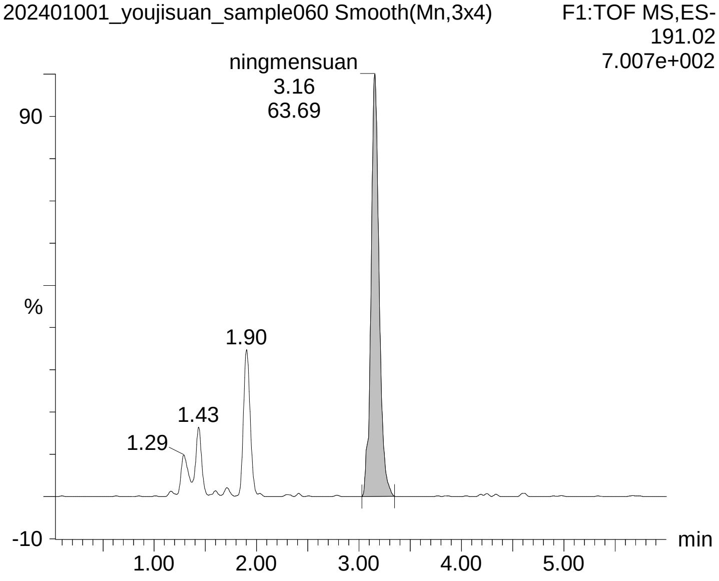

pinguosuan

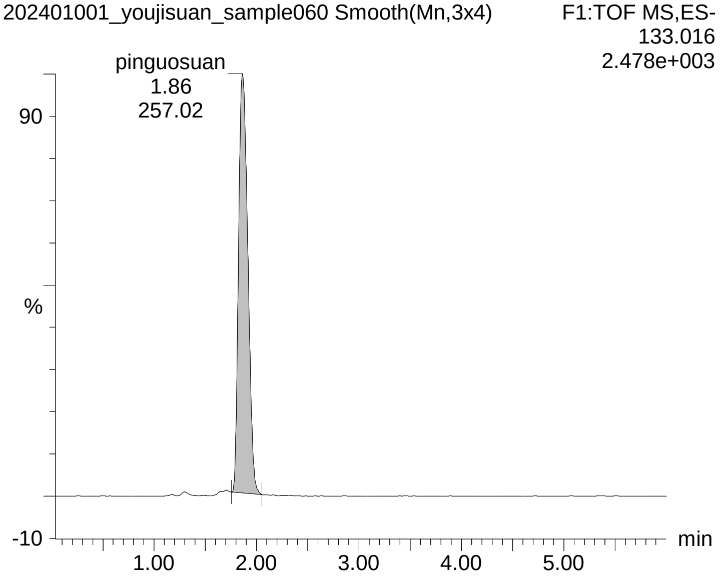

	#	Name	Sample Text	RT	Area	Std. Conc	Conc.
1	1	jiushisuan		1.50	1586.586		290.241054
2	2	ningmensuan		3.16	63.690		8.169691
3	3	pinguosuan		1.86	257.022		97.662471

project_wangzhonghua_BeiMu
Dataset: Untitled
Last Altered: Friday, October 11, 2024 15:45:23 China Standard Time
Printed: Friday, October 11, 2024 15:46:51 China Standard Time

Name: 202401001_youjisuan_sample061, Date: 02-Oct-2024, Time: 01:55:46, ID: , Description:

jiushisuan

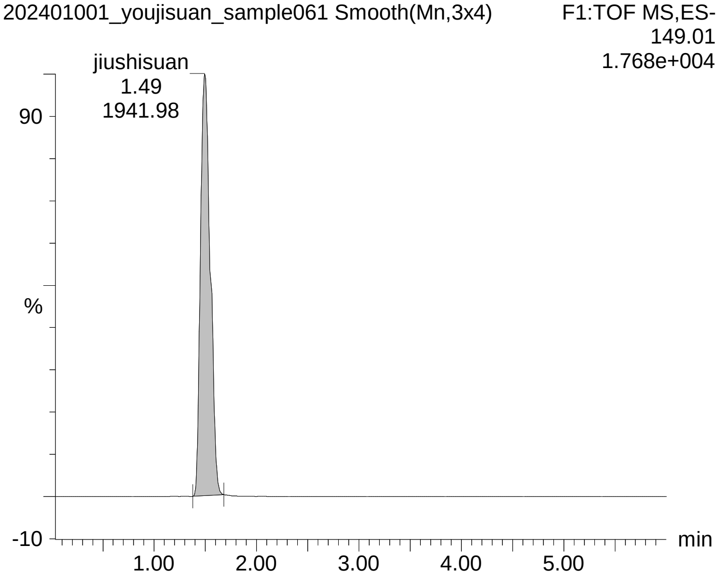

ningmensuan

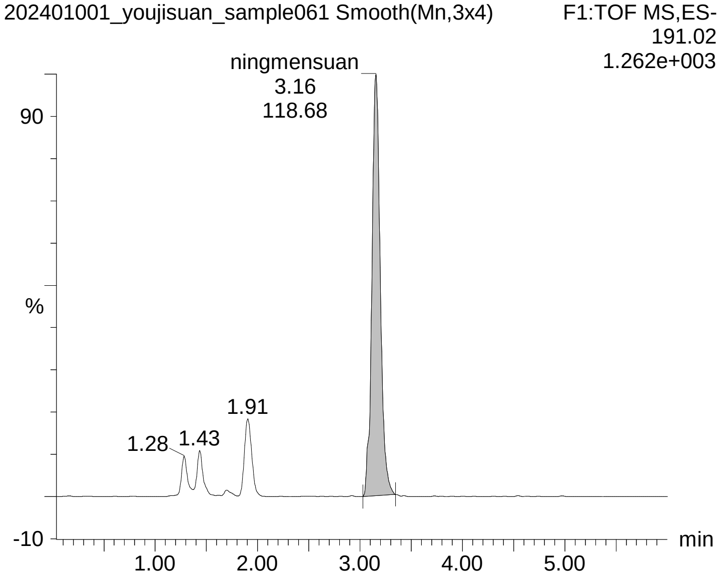

pinguosuan

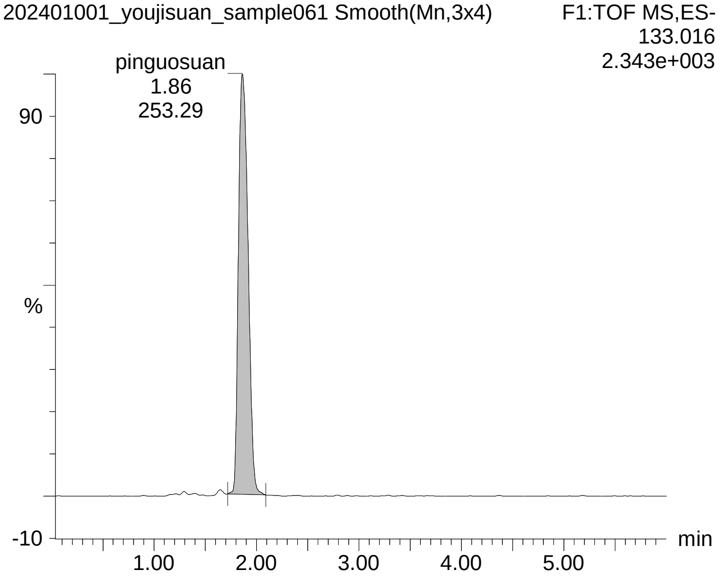

	#	Name	Sample Text	RT	Area	Std. Conc	Conc.
1	1	jiushisuan		1.49	1941.978		441.225057
2	2	ningmensuan		3.16	118.680		16.183967
3	3	pinguosuan		1.86	253.291		95.431811

Name: 202401001_youjisuan_sample062, Date: 02-Oct-2024, Time: 02:02:46, ID: , Description:

jiushisuan

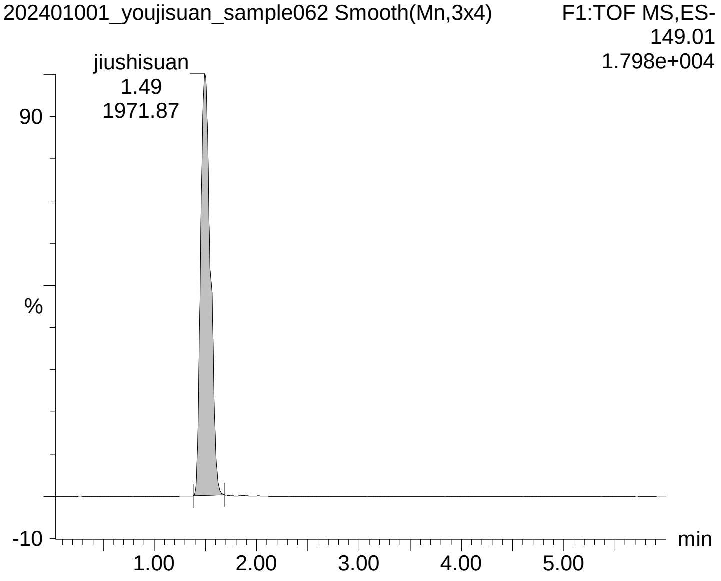

ningmensuan

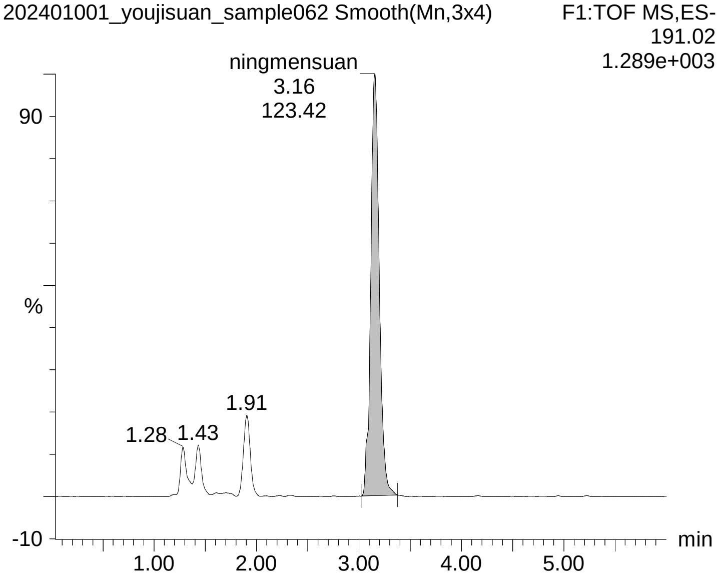

pinguosuan

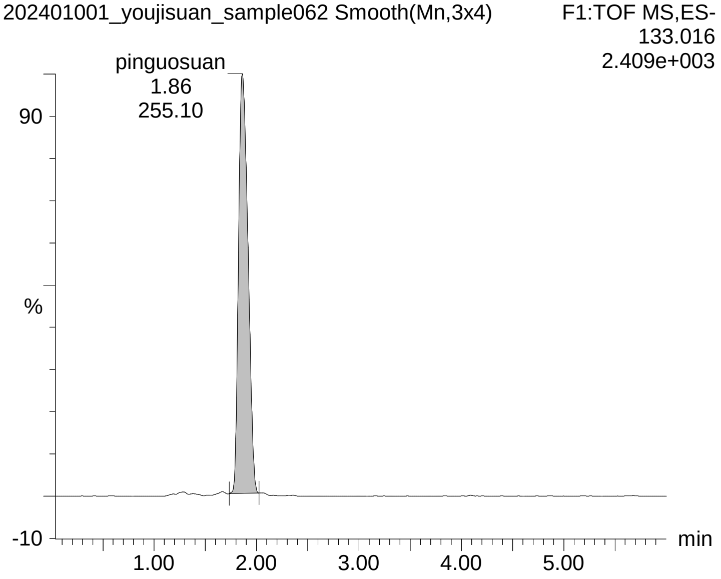

	#	Name	Sample Text	RT	Area	Std. Conc	Conc.
1	1	jiushisuan		1.49	1971.870		459.489010
2	2	ningmensuan		3.16	123.417		16.874341
3	3	pinguosuan		1.86	255.103		96.510058

Name: 202401001_youjisuan_sample063, Date: 02-Oct-2024, Time: 02:10:43, ID: , Description:

jiushisuan

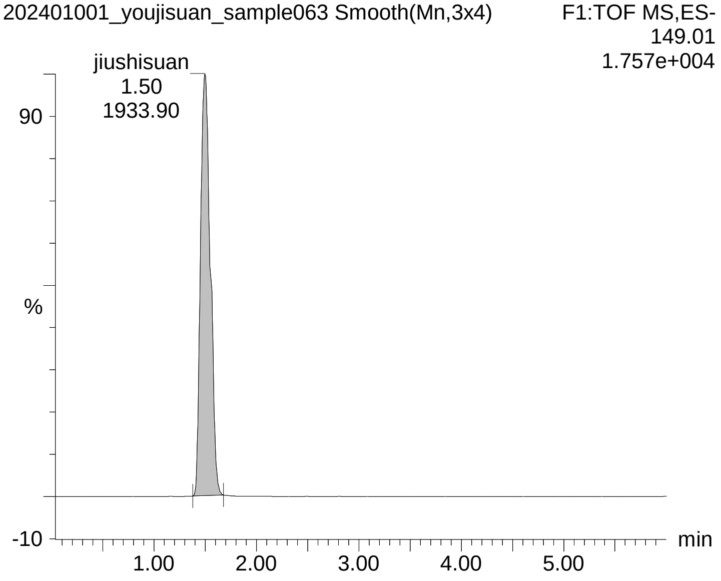

ningmensuan

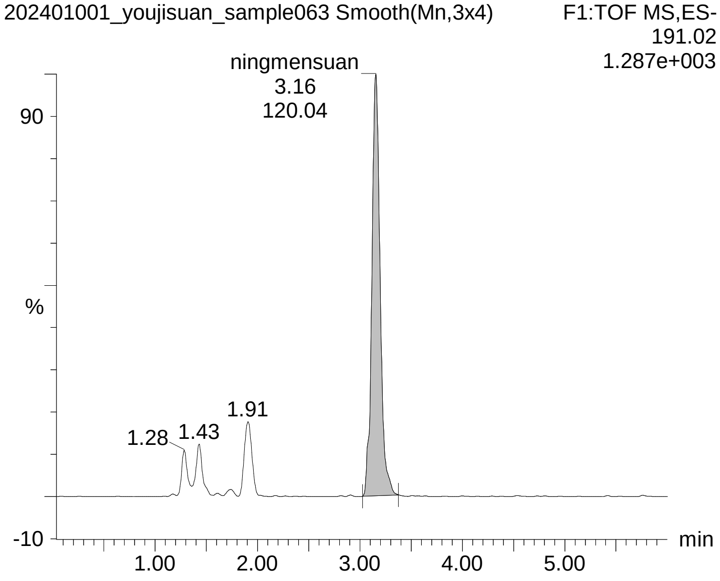

pinguosuan

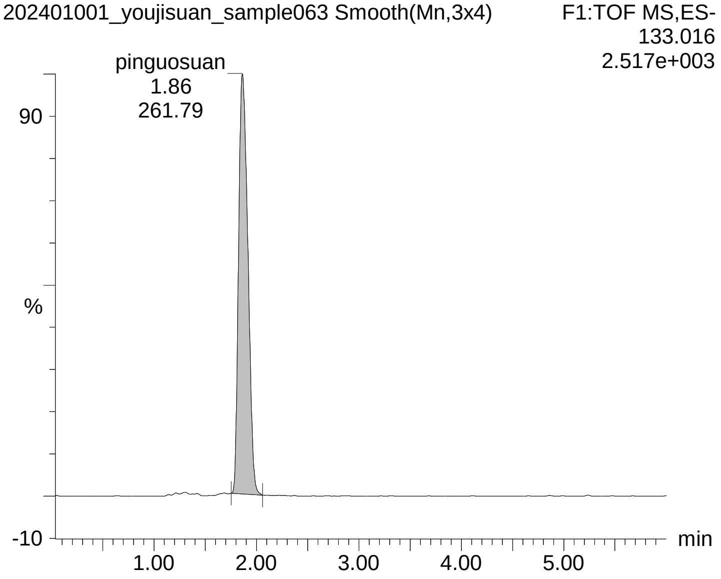

	#	Name	Sample Text	RT	Area	Std. Conc	Conc.
1	1	jiushisuan		1.50	1933.895		436.518485
2	2	ningmensuan		3.16	120.035		16.381446
3	3	pinguosuan		1.86	261.790		100.574181

Name: 202401001_youjisuan_sample064, Date: 02-Oct-2024, Time: 02:17:43, ID: , Description:

jiushisuan

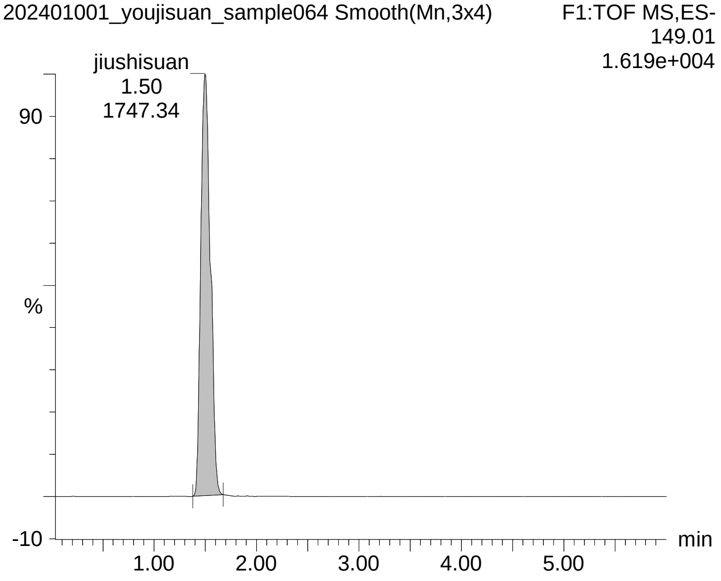

ningmensuan

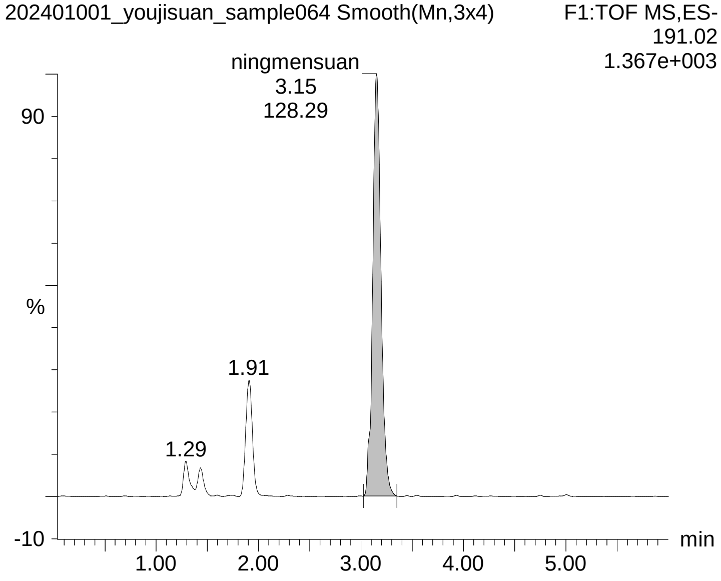

pinguosuan

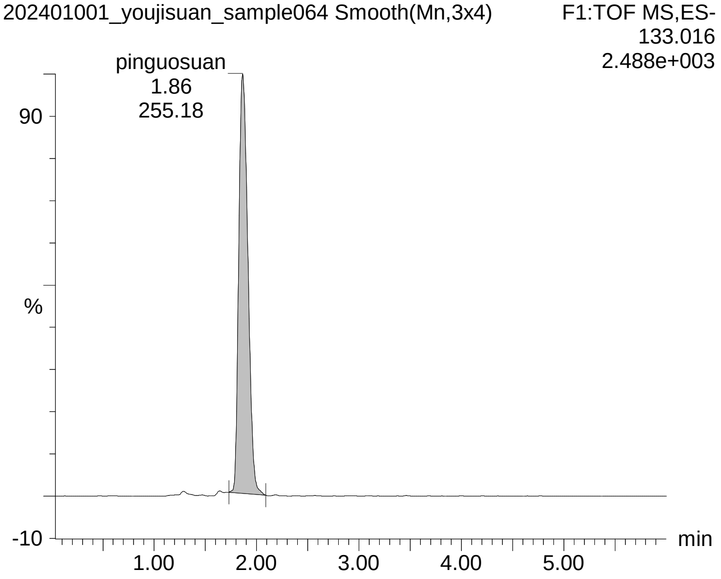

	#	Name	Sample Text	RT	Area	Std. Conc	Conc.
1	1	jiushisuan		1.50	1747.340		347.605212
2	2	ningmensuan		3.15	128.295		17.585263
3	3	pinguosuan		1.86	255.182		96.557285

project_wangzhonghua_BeiMu
Dataset: Untitled
Last Altered: Friday, October 11, 2024 15:45:23 China Standard Time
Printed: Friday, October 11, 2024 15:46:51 China Standard Time

Name: 202401001_youjisuan_sample065, Date: 02-Oct-2024, Time: 02:24:43, ID: , Description:

jiushisuan

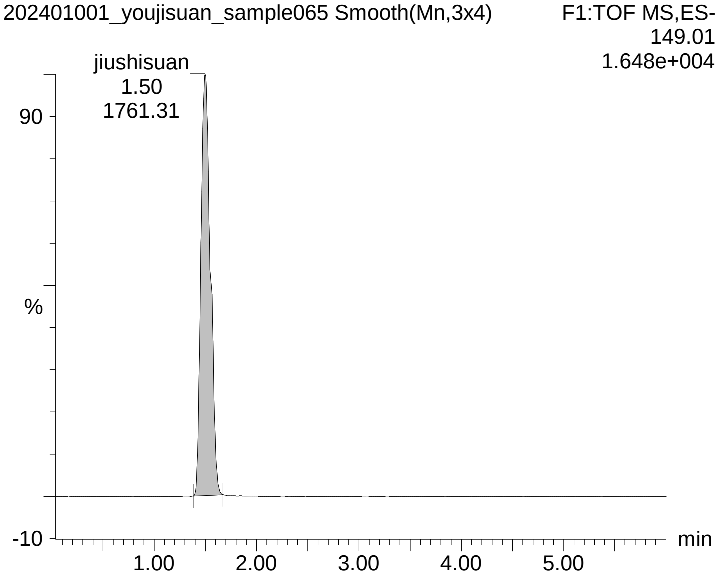

ningmensuan

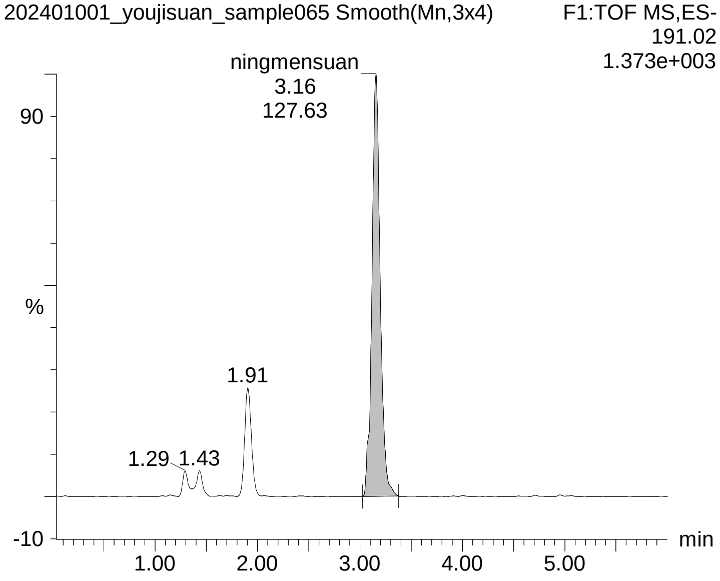

pinguosuan

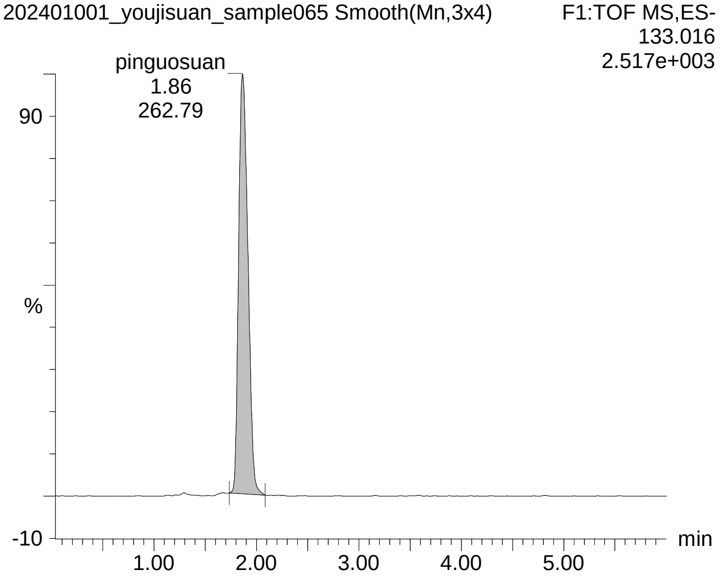

	#	Name	Sample Text	RT	Area	Std. Conc	Conc.
1	1	jiushisuan		1.50	1761.310		353.254098
2	2	ningmensuan		3.16	127.625		17.487617
3	3	pinguosuan		1.86	262.793		101.195761

Name: 202401001_youjisuan_sample066, Date: 02-Oct-2024, Time: 02:31:44, ID: , Description:

jiushisuan

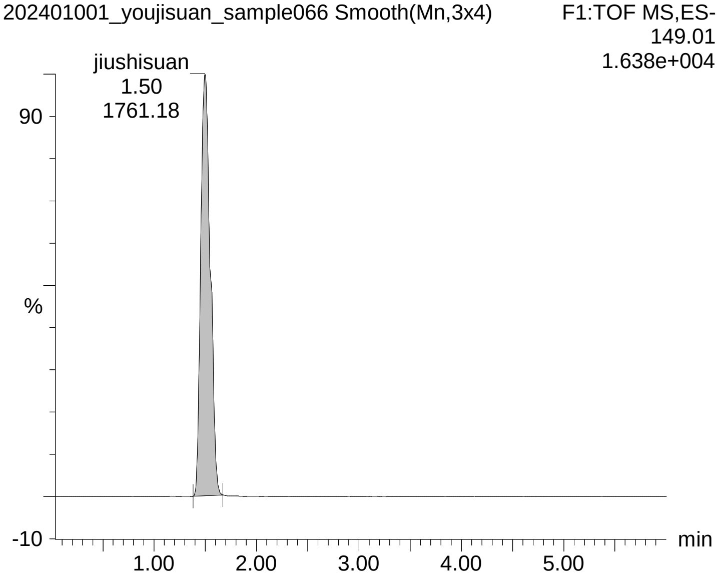

ningmensuan

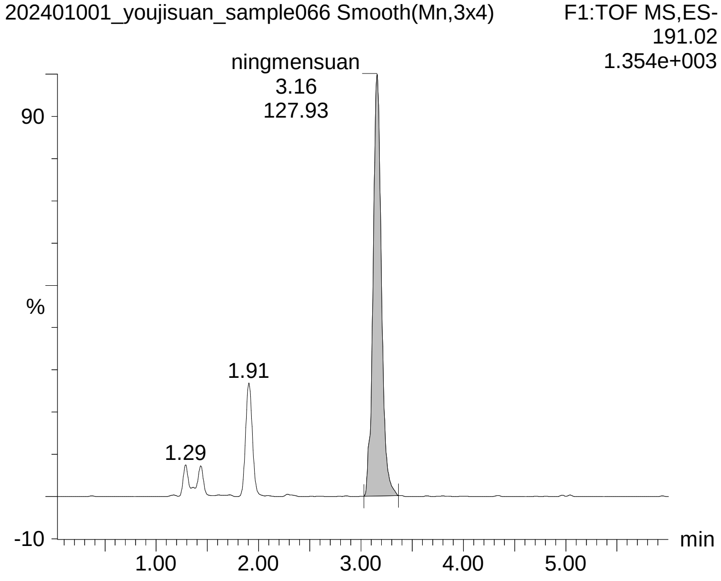

pinguosuan

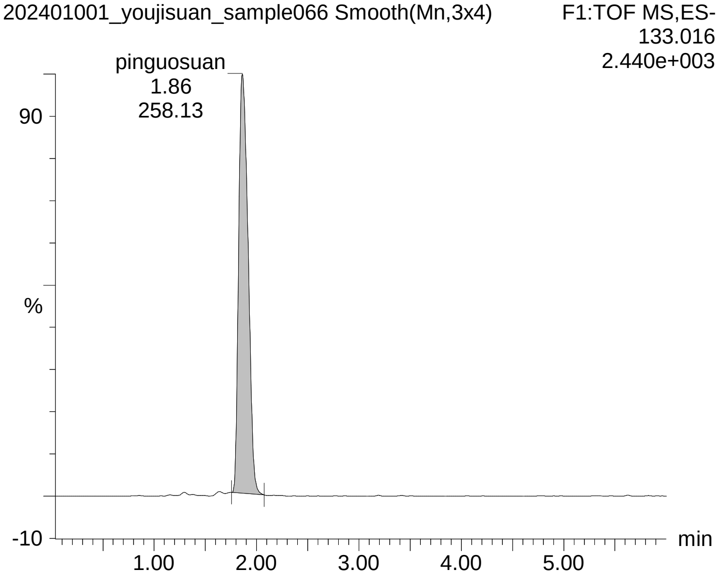

	#	Name	Sample Text	RT	Area	Std. Conc	Conc.
1	1	jiushisuan		1.50	1761.181		353.201349
2	2	ningmensuan		3.16	127.930		17.532068
3	3	pinguosuan		1.86	258.130		98.332869

project_wangzhonghua_BeiMu
Dataset: Untitled
Last Altered: Friday, October 11, 2024 15:45:23 China Standard Time
Printed: Friday, October 11, 2024 15:46:51 China Standard Time

Name: 202401001_youjisuan_sample067, Date: 02-Oct-2024, Time: 02:38:38, ID: , Description:

jiushisuan

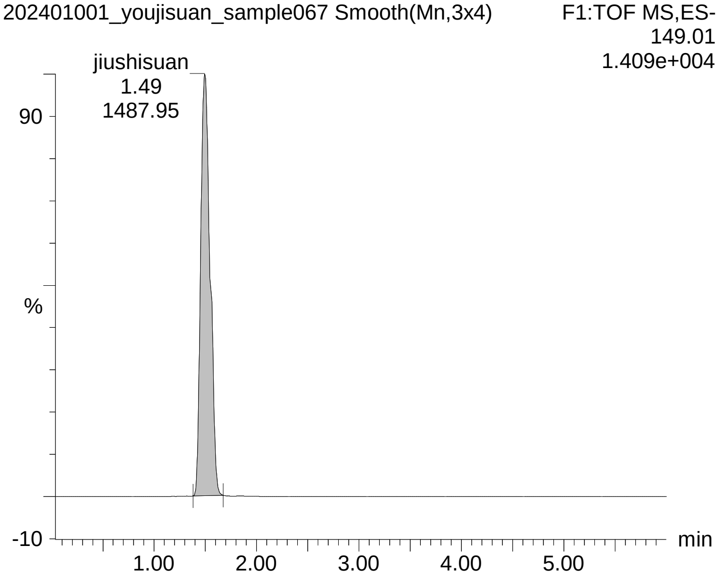

ningmensuan

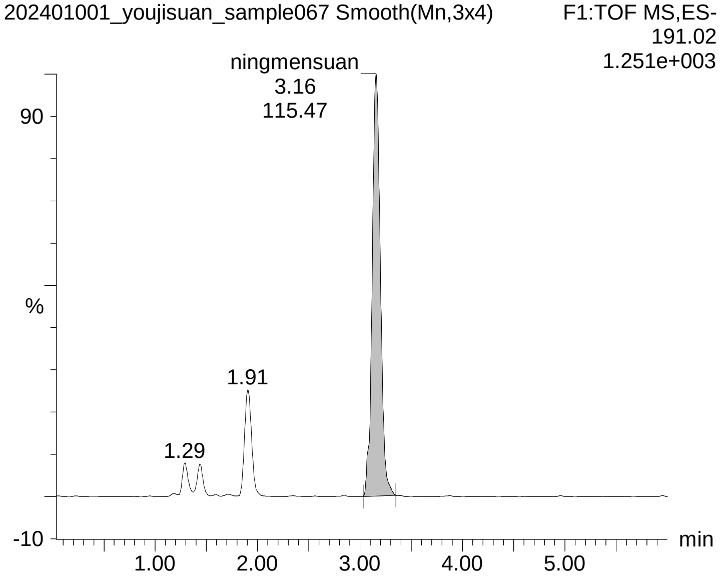

pinguosuan

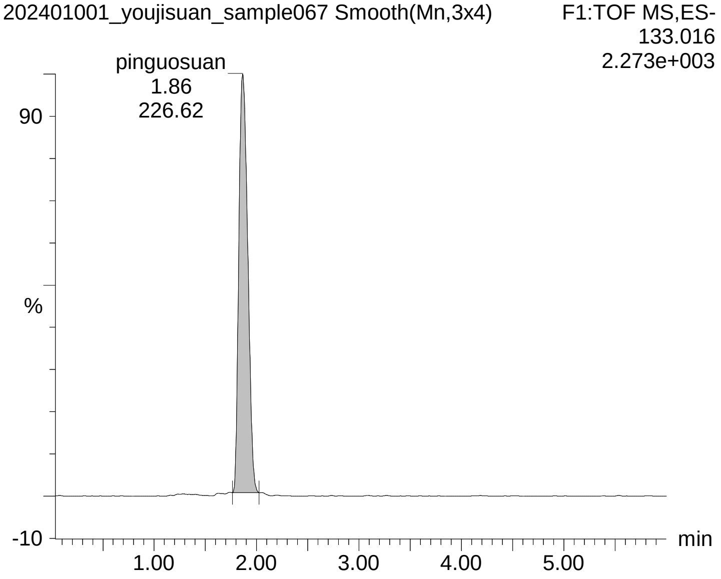

	#	Name	Sample Text	RT	Area	Std. Conc	Conc.
1	1	jiushisuan		1.49	1487.948		260.336977
2	2	ningmensuan		3.16	115.475		15.716869
3	3	pinguosuan		1.86	226.624		80.556079

project_wangzhonghua_BeiMu
Dataset: Untitled
Last Altered: Friday, October 11, 2024 15:45:23 China Standard Time
Printed: Friday, October 11, 2024 15:46:51 China Standard Time

Name: 202401001_youjisuan_sample068, Date: 02-Oct-2024, Time: 02:45:36, ID: , Description:

jiushisuan

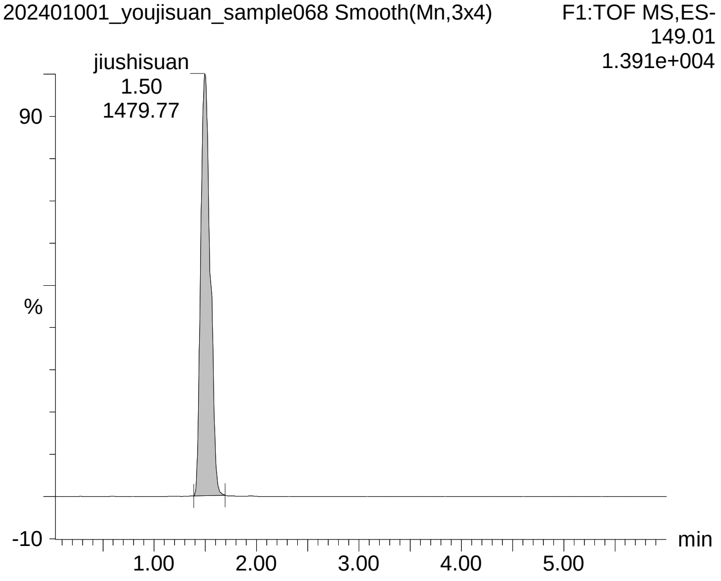

ningmensuan

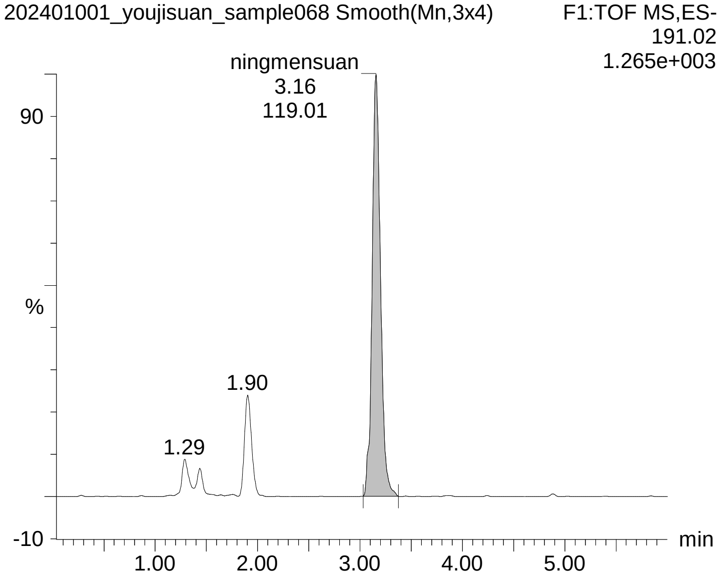

pinguosuan

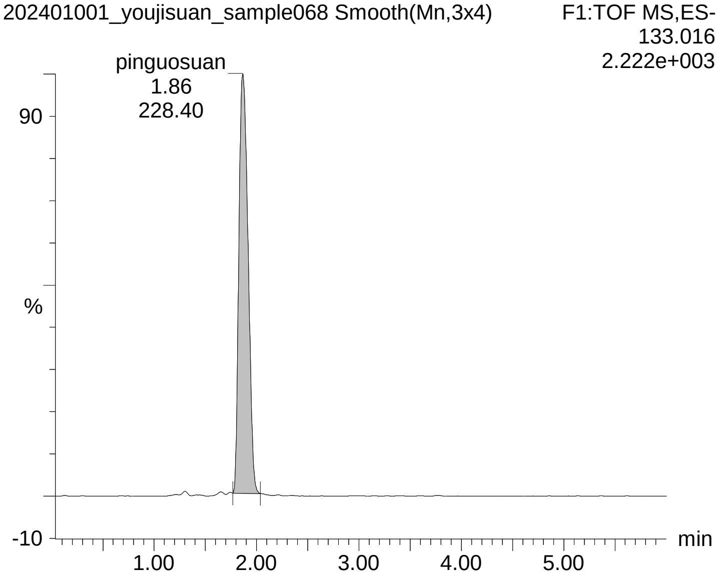

	#	Name	Sample Text	RT	Area	Std. Conc	Conc.
1	1	jiushisuan		1.50	1479.772		257.998353
2	2	ningmensuan		3.16	119.005		16.231333
3	3	pinguosuan		1.86	228.397		81.492838

Name: 202401001_youjisuan_sample069, Date: 02-Oct-2024, Time: 02:52:37, ID: , Description:

jiushisuan

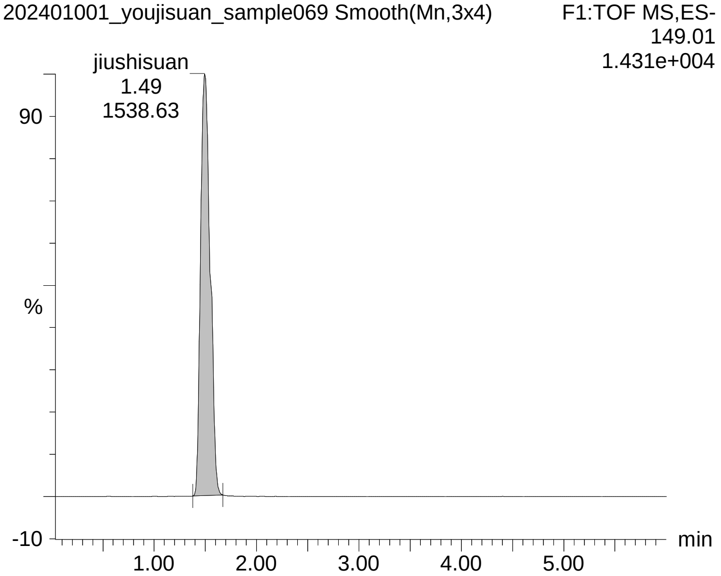

ningmensuan

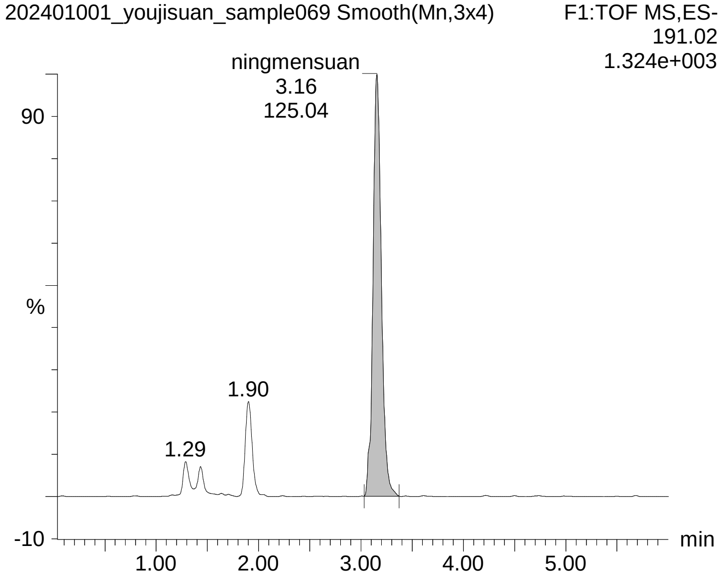

pinguosuan

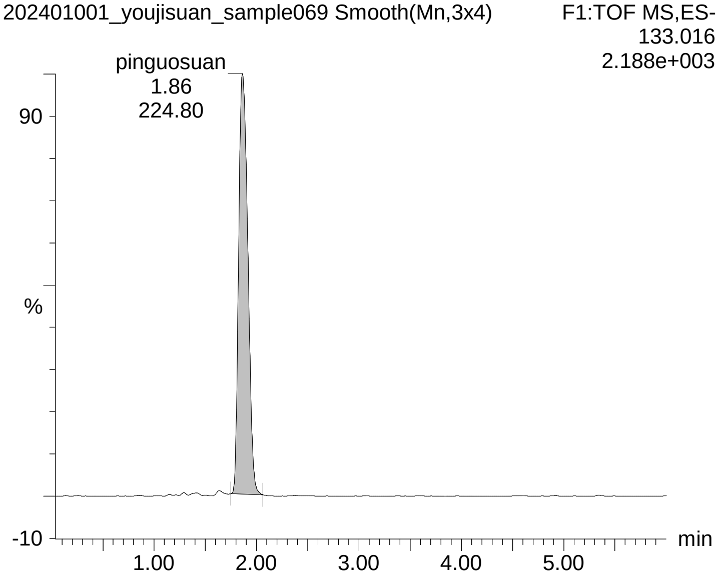

	#	Name	Sample Text	RT	Area	Std. Conc	Conc.
1	1	jiushisuan		1.49	1538.629		275.292365
2	2	ningmensuan		3.16	125.038		17.110586
3	3	pinguosuan		1.86	224.802		79.600438

Name: 202401001_youjisuan_sample070, Date: 02-Oct-2024, Time: 02:59:37, ID: , Description:

jiushisuan

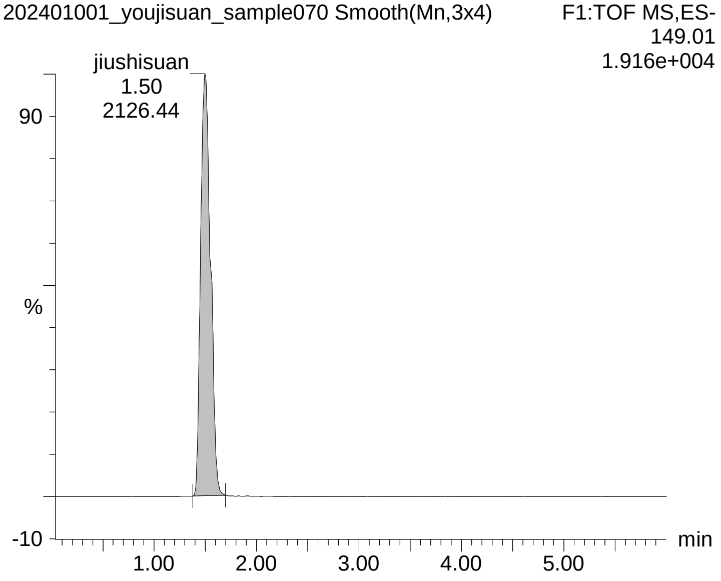

ningmensuan

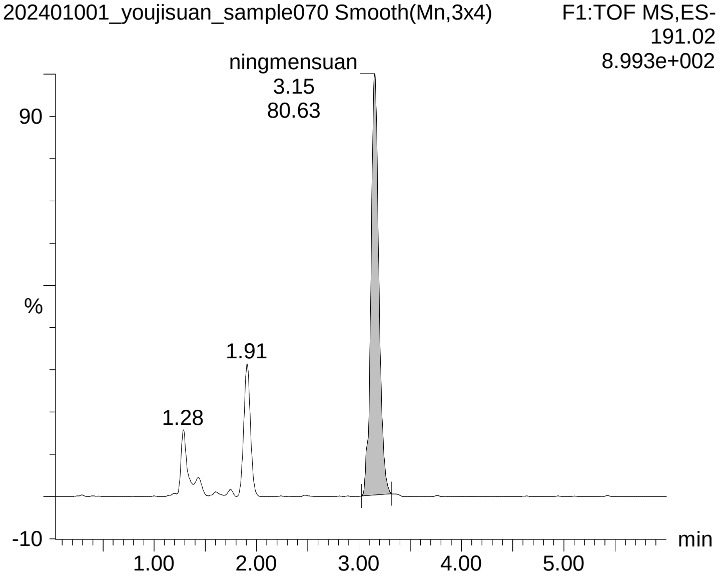

pinguosuan

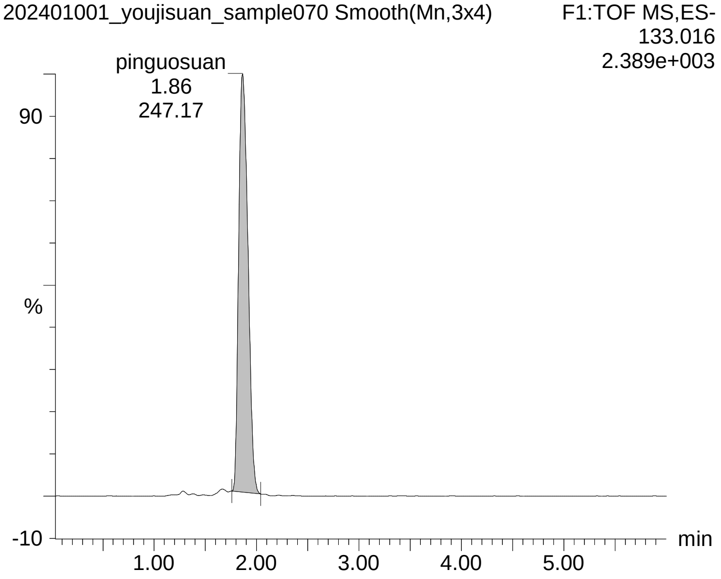

	#	Name	Sample Text	RT	Area	Std. Conc	Conc.
1	1	jiushisuan		1.50	2126.444		584.301331
2	2	ningmensuan		3.15	80.627		10.638100
3	3	pinguosuan		1.86	247.171		91.858474

Name: 202401001_youjisuan_sample071, Date: 02-Oct-2024, Time: 03:06:37, ID: , Description:

jiushisuan

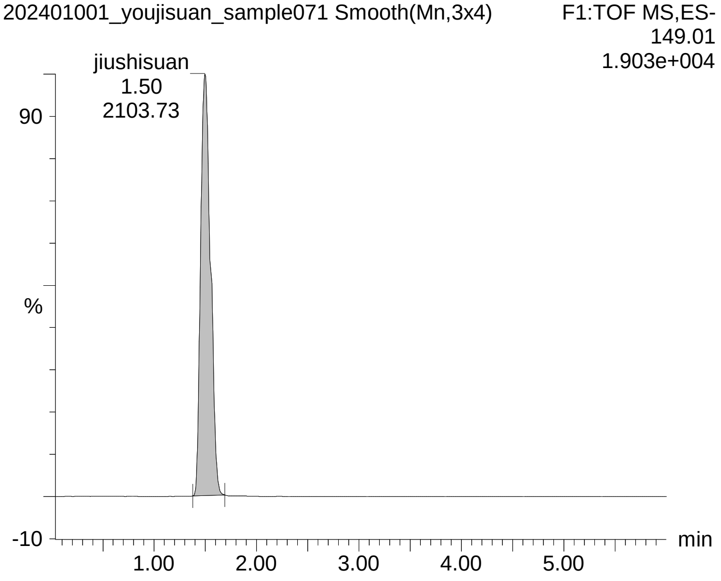

ningmensuan

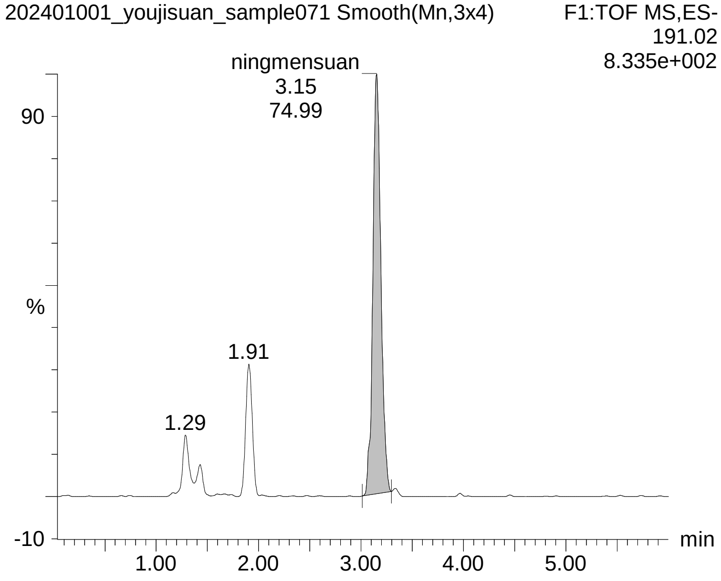

pinguosuan

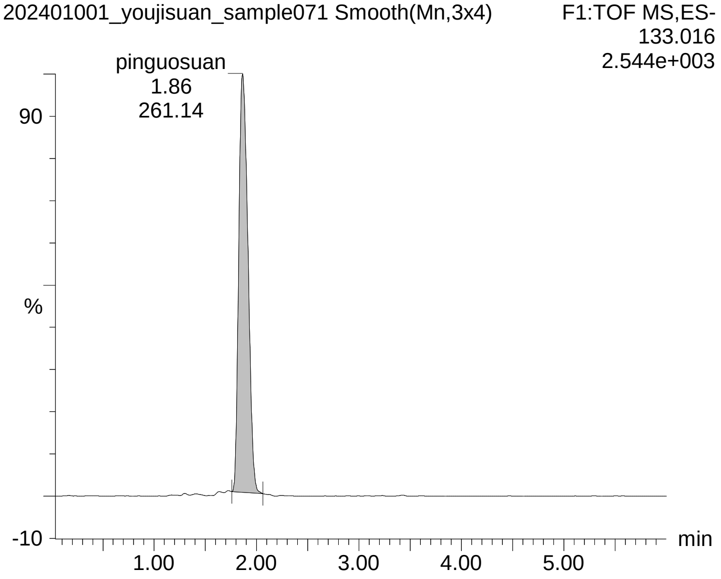

	#	Name	Sample Text	RT	Area	Std. Conc	Conc.
1	1	jiushisuan		1.50	2103.726		561.908423
2	2	ningmensuan		3.15	74.988		9.816269
3	3	pinguosuan		1.86	261.137		100.171226

Name: 202401001_youjisuan_sample072, Date: 02-Oct-2024, Time: 03:13:38, ID: , Description:

jiushisuan

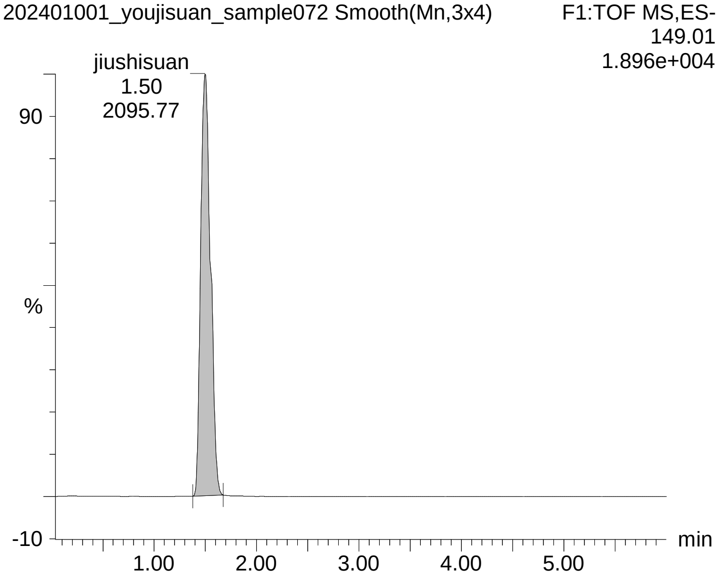

ningmensuan

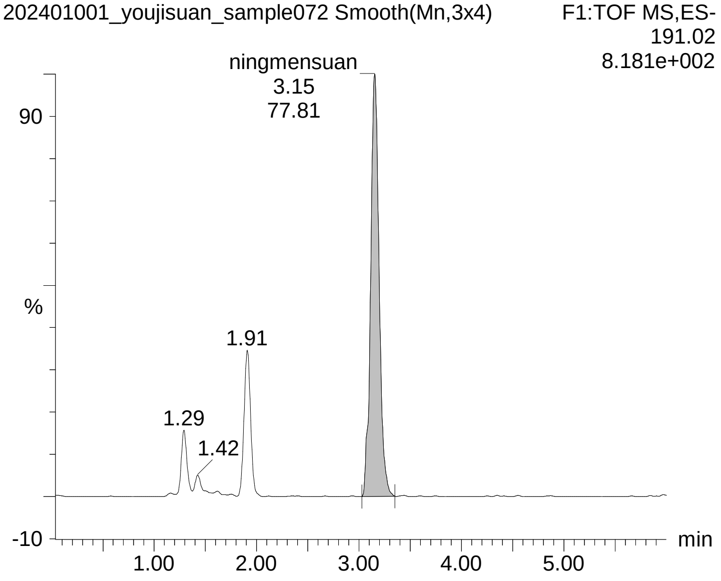

pinguosuan

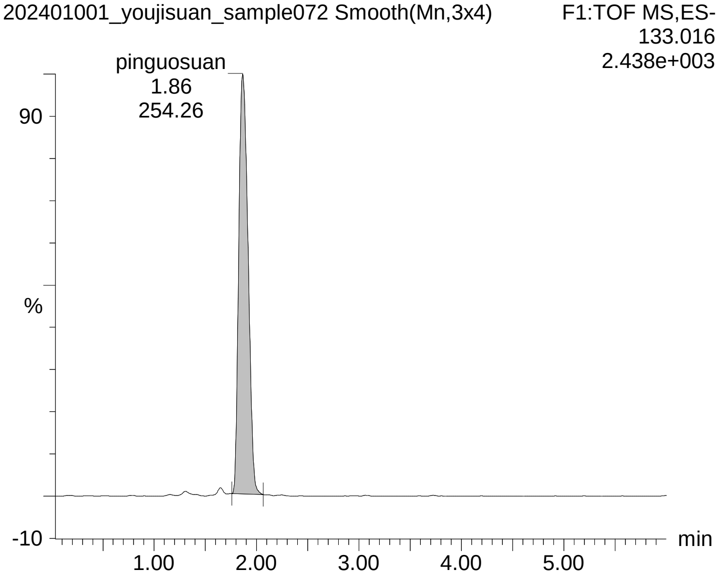

	#	Name	Sample Text	RT	Area	Std. Conc	Conc.
1	1	jiushisuan		1.50	2095.766		554.439762
2	2	ningmensuan		3.15	77.810		10.227549
3	3	pinguosuan		1.86	254.263		96.009025

Name: 202401001_youjisuan_sample073, Date: 02-Oct-2024, Time: 03:20:41, ID: , Description:

jiushisuan

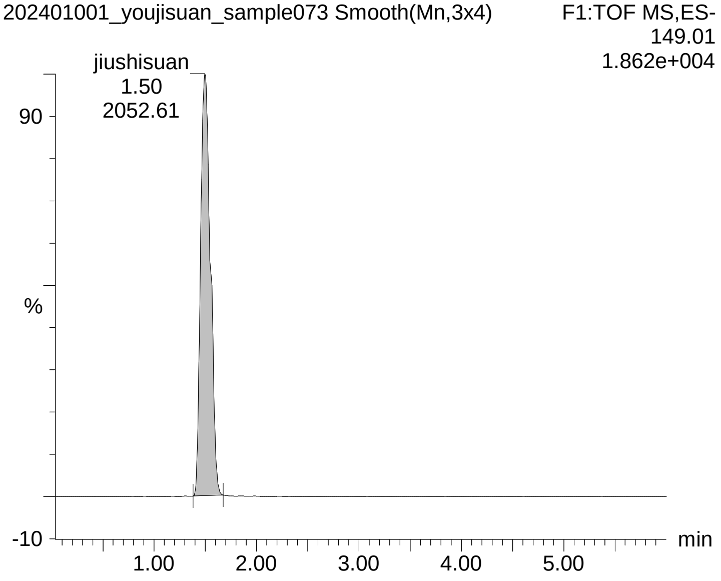

ningmensuan

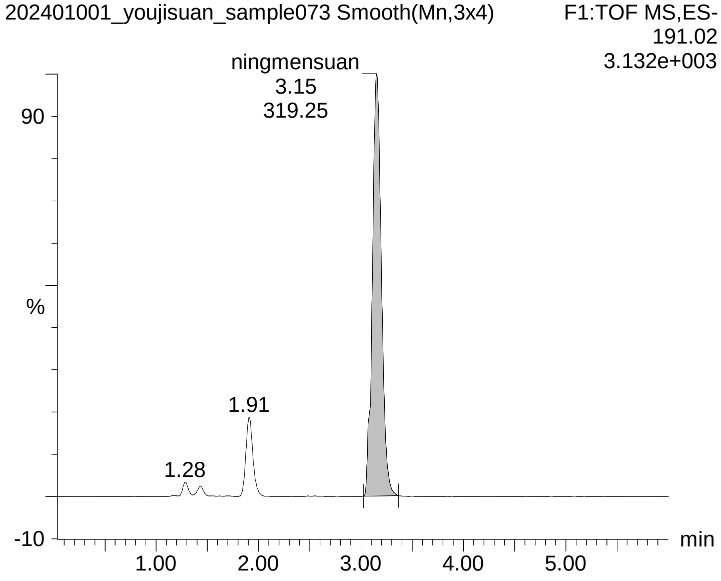

pinguosuan

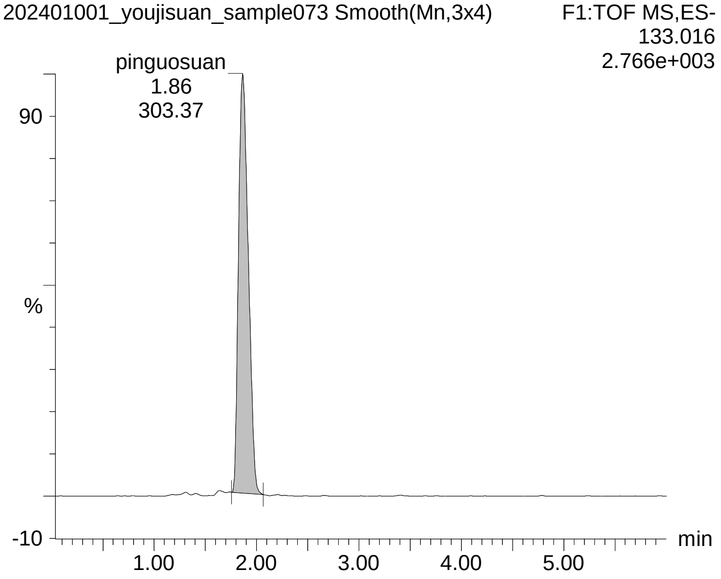

	#	Name	Sample Text	RT	Area	Std. Conc	Conc.
1	1	jiushisuan		1.50	2052.607		517.152999
2	2	ningmensuan		3.15	319.254		45.415744
3	3	pinguosuan		1.86	303.369		129.647117

Name: 202401001_youjisuan_sample074, Date: 02-Oct-2024, Time: 03:28:37, ID: , Description:

jiushisuan

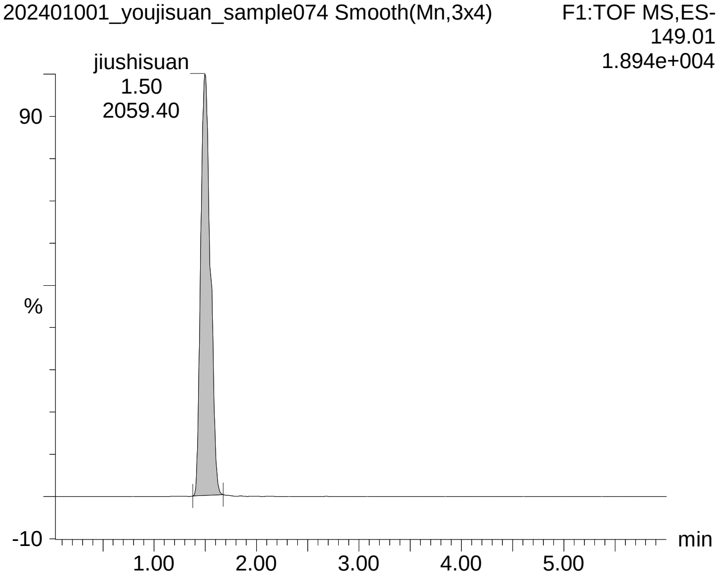

ningmensuan

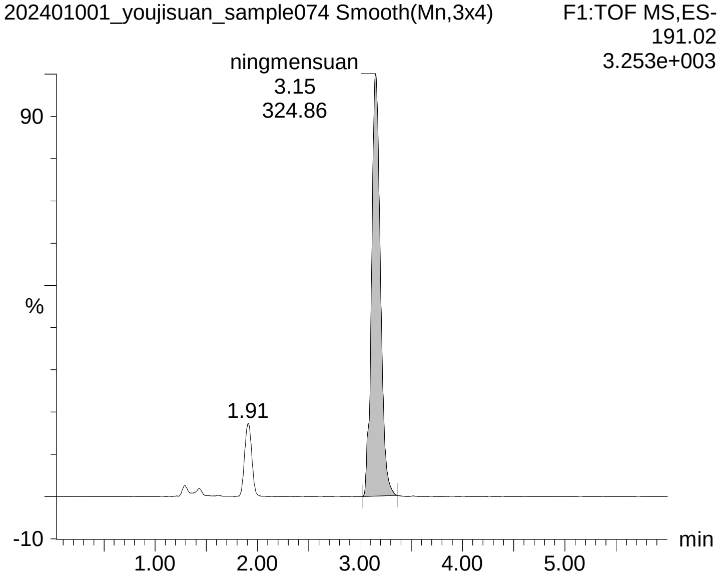

pinguosuan

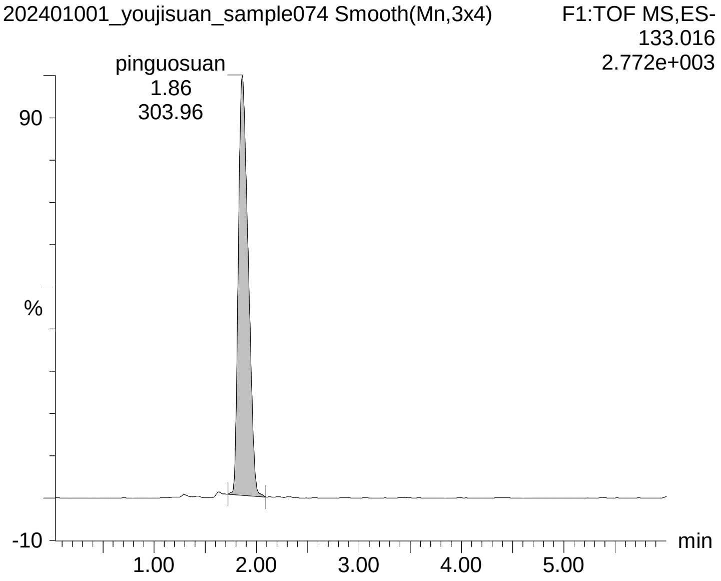

	#	Name	Sample Text	RT	Area	Std. Conc	Conc.
1	1	jiushisuan		1.50	2059.395		522.677402
2	2	ningmensuan		3.15	324.857		46.232329
3	3	pinguosuan		1.86	303.961		130.121455

project_wangzhonghua_BeiMu
Dataset: Untitled
Last Altered: Friday, October 11, 2024 15:45:23 China Standard Time
Printed: Friday, October 11, 2024 15:46:51 China Standard Time

Name: 202401001_youjisuan_sample075, Date: 02-Oct-2024, Time: 03:35:36, ID: , Description:

jiushisuan

202401001_youjisuan_sample075 Smooth(Mn,3x4) F1:TOF MS,ES-
149.01
1.915e+004

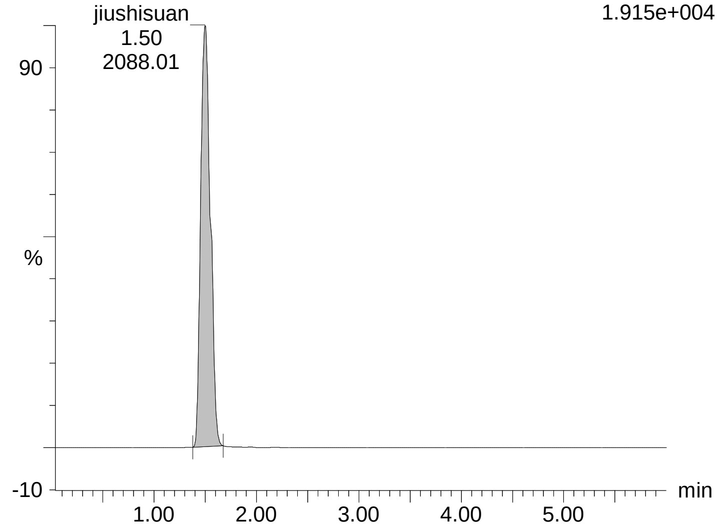

ningmensuan

202401001_youjisuan_sample075 Smooth(Mn,3x4) F1:TOF MS,ES-
191.02
3.214e+003

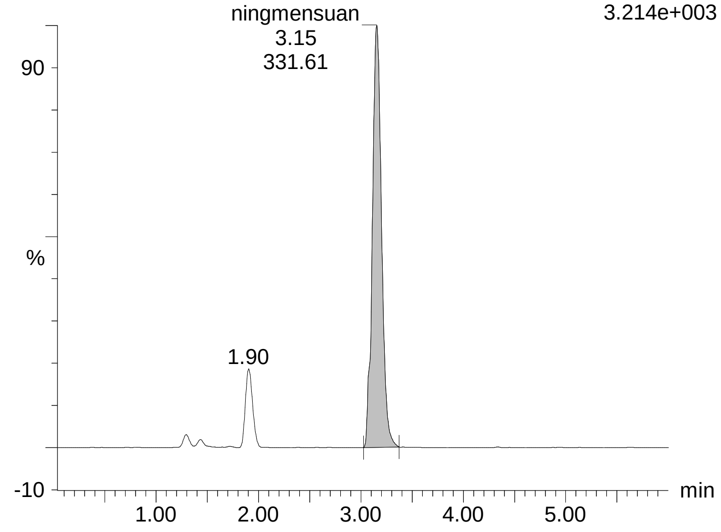

pinguosuan

202401001_youjisuan_sample075 Smooth(Mn,3x4) F1:TOF MS,ES-
133.016
2.879e+003

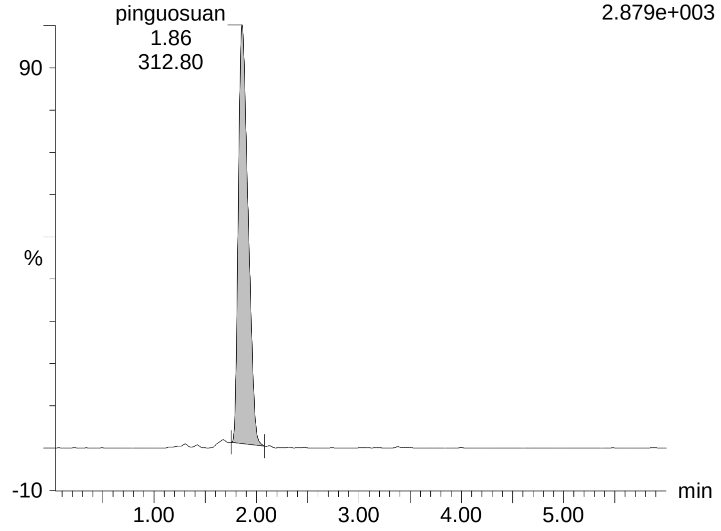

	#	Name	Sample Text	RT	Area	Std. Conc	Conc.
1	1	jiushisuan		1.50	2088.006		547.342998
2	2	ningmensuan		3.15	331.613		47.216952
3	3	pinguosuan		1.86	312.803		137.473446

Name: 202401001_youjisuan_sample076, Date: 02-Oct-2024, Time: 03:42:36, ID: , Description:

jiushisuan

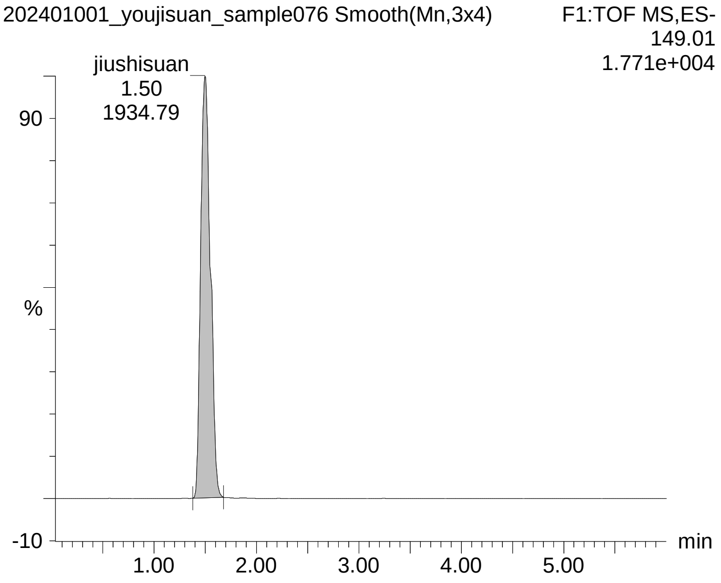

ningmensuan

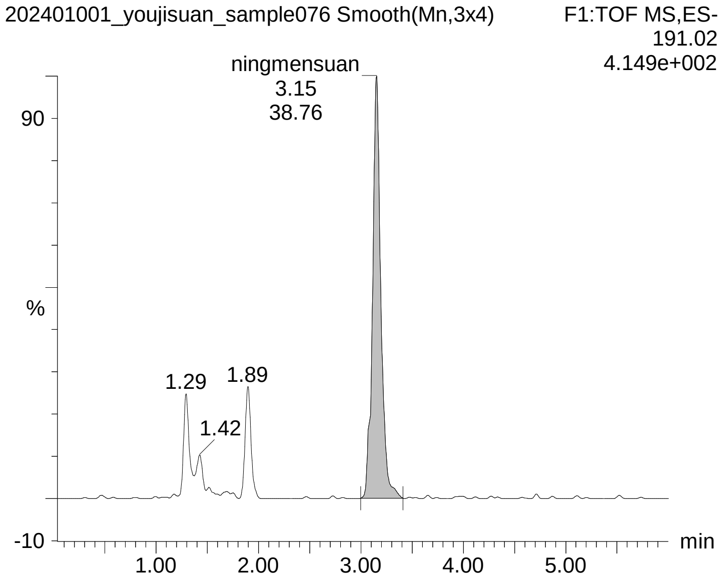

pinguosuan

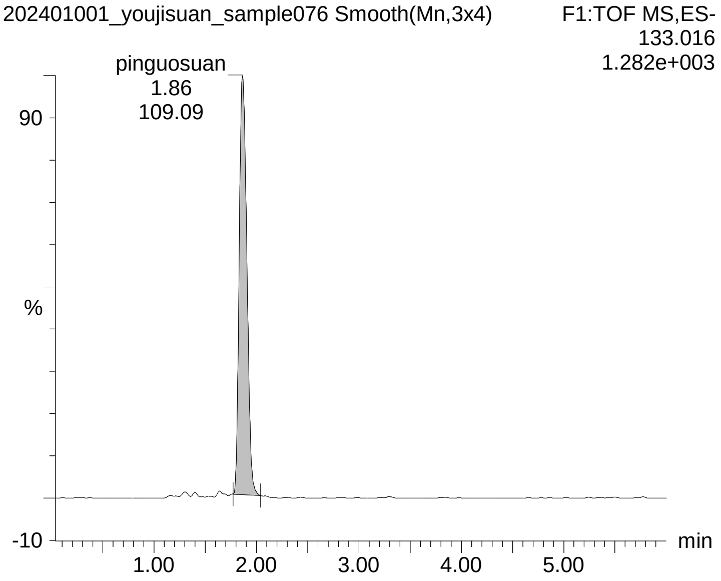

	#	Name	Sample Text	RT	Area	Std. Conc	Conc.
1	1	jiushisuan		1.50	1934.785		437.032168
2	2	ningmensuan		3.15	38.755		4.535649
3	3	pinguosuan		1.86	109.094		29.456665

Name: 202401001_youjisuan_sample077, Date: 02-Oct-2024, Time: 03:49:36, ID: , Description:

jiushisuan

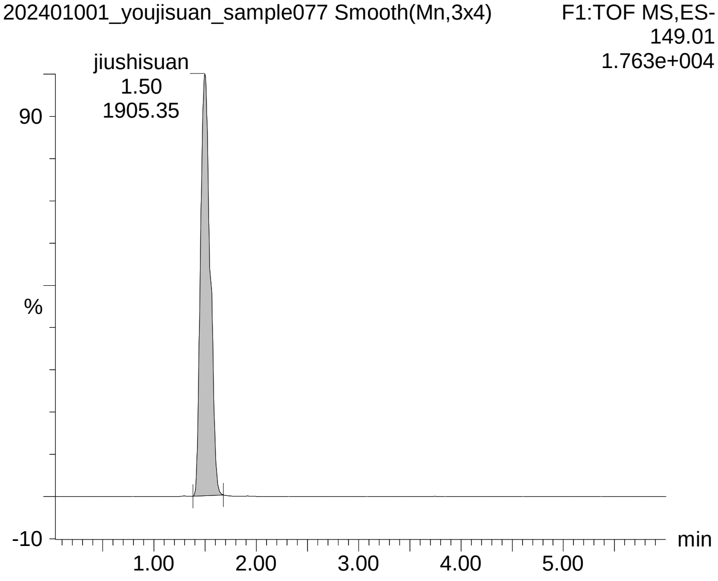

ningmensuan

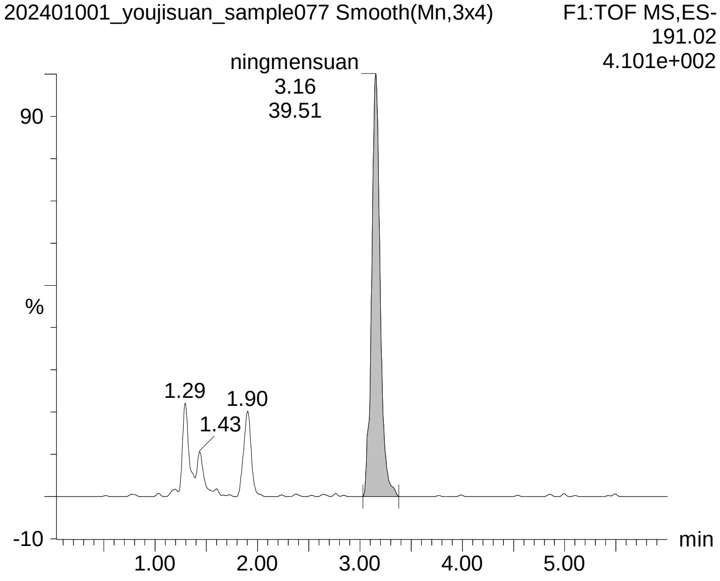

pinguosuan

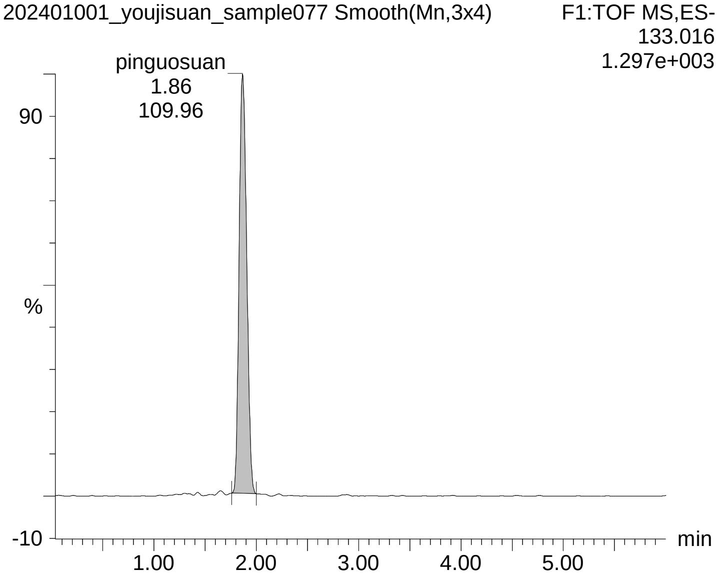

	#	Name	Sample Text	RT	Area	Std. Conc	Conc.
1	1	jiushisuan		1.50	1905.352		420.606498
2	2	ningmensuan		3.16	39.509		4.645538
3	3	pinguosuan		1.86	109.961		29.775014

Name: 202401001_youjisuan_sample078, Date: 02-Oct-2024, Time: 03:56:37, ID: , Description:

jiushisuan

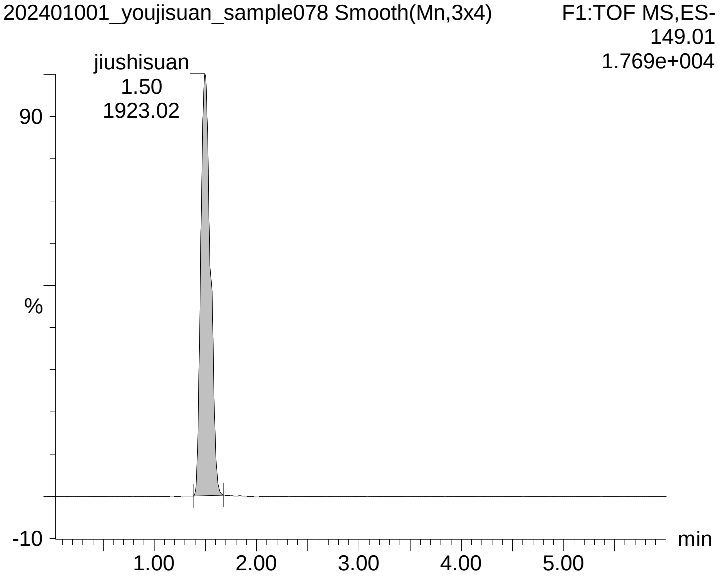

ningmensuan

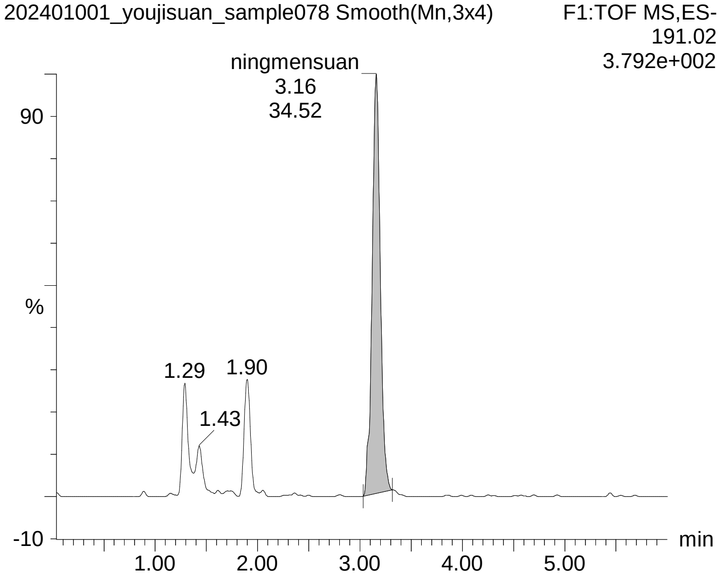

pinguosuan

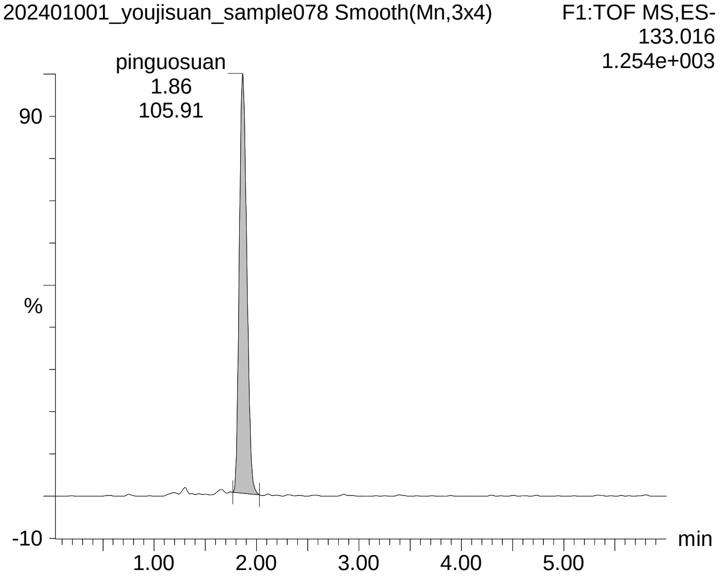

	#	Name	Sample Text	RT	Area	Std. Conc	Conc.
1	1	jiushisuan		1.50	1923.018		430.328691
2	2	ningmensuan		3.16	34.525		3.919166
3	3	pinguosuan		1.86	105.907		28.292062

Name: 202401001_youjisuan_sample079, Date: 02-Oct-2024, Time: 04:03:37, ID: , Description:

jiushisuan

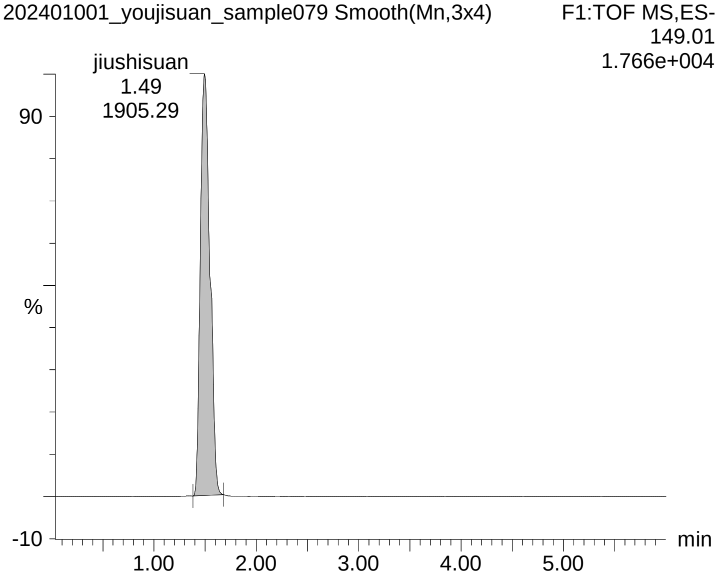

ningmensuan

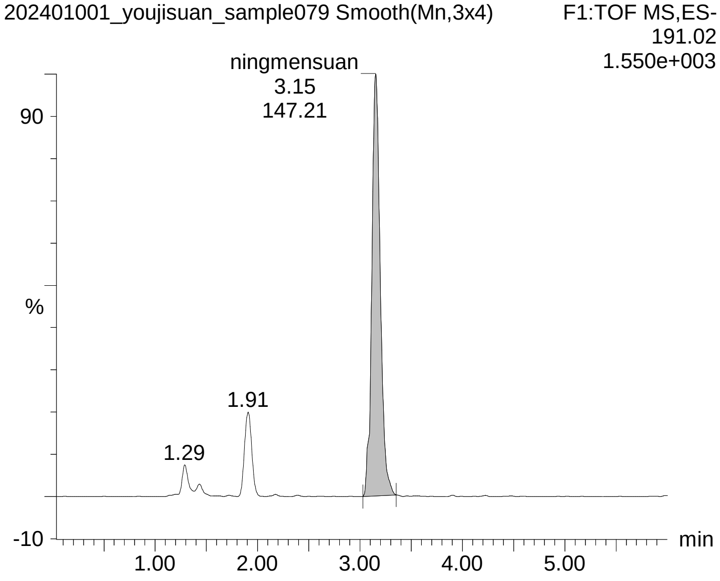

pinguosuan

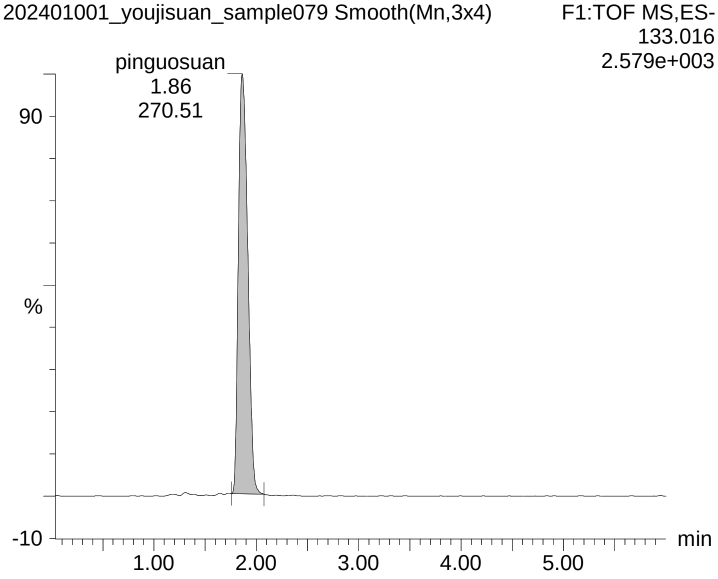

	#	Name	Sample Text	RT	Area	Std. Conc	Conc.
1	1	jiushisuan		1.49	1905.294		420.575224
2	2	ningmensuan		3.15	147.207		20.341510
3	3	pinguosuan		1.86	270.513		106.091860

Name: 202401001_youjisuan_sample080, Date: 02-Oct-2024, Time: 04:10:37, ID: , Description:

jiushisuan

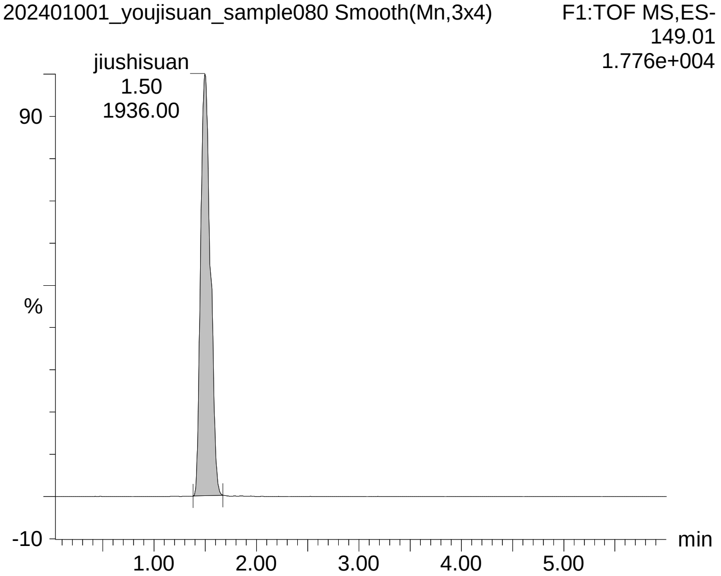

ningmensuan

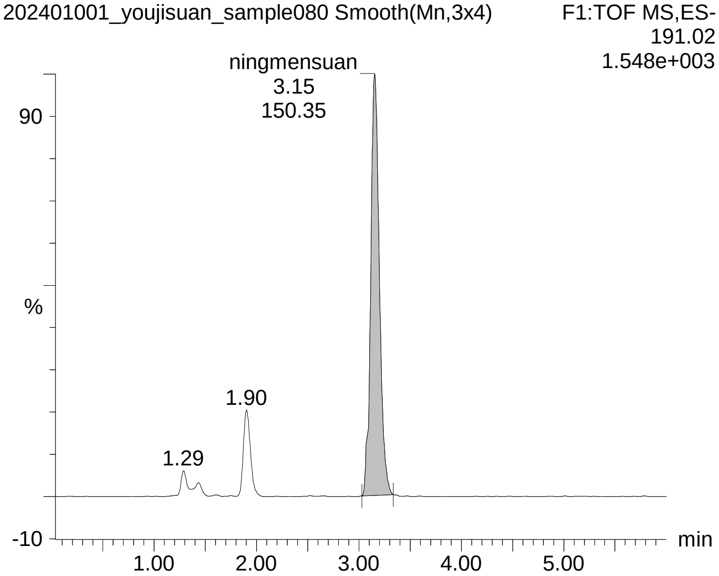

pinguosuan

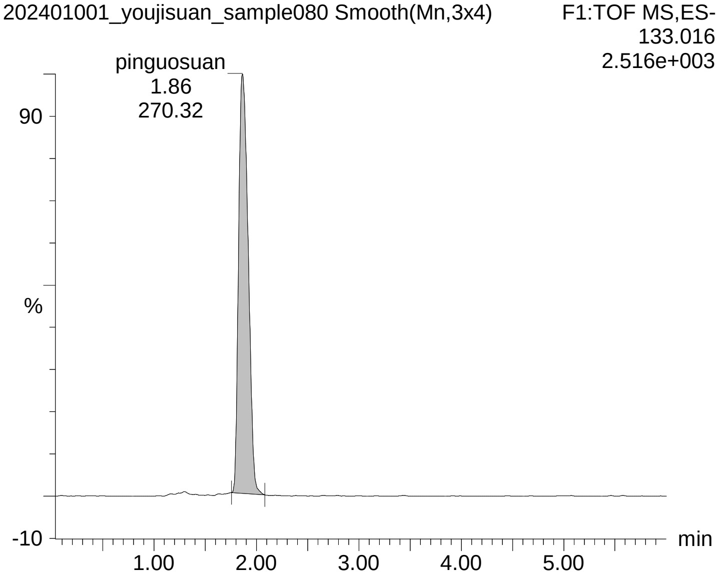

	#	Name	Sample Text	RT	Area	Std. Conc	Conc.
1	1	jiushisuan		1.50	1935.996		437.732913
2	2	ningmensuan		3.15	150.346		20.798989
3	3	pinguosuan		1.86	270.316		105.964365

Name: 202401001_youjisuan_sample081, Date: 02-Oct-2024, Time: 04:17:39, ID: , Description:

jiushisuan

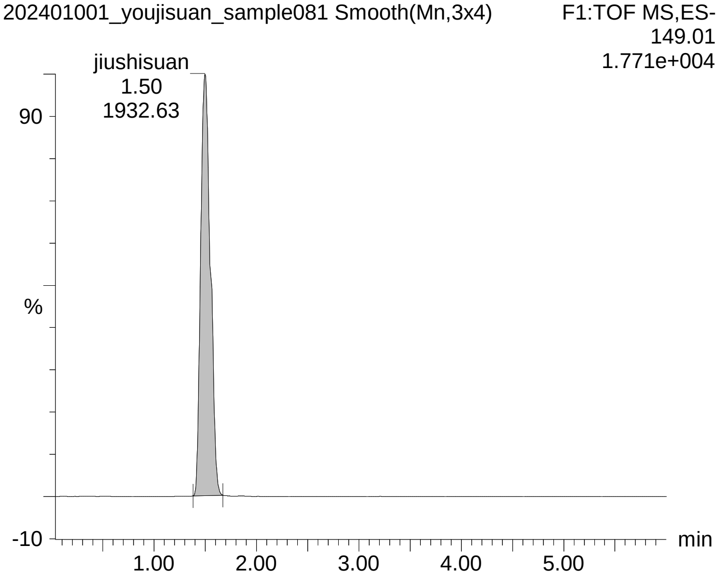

ningmensuan

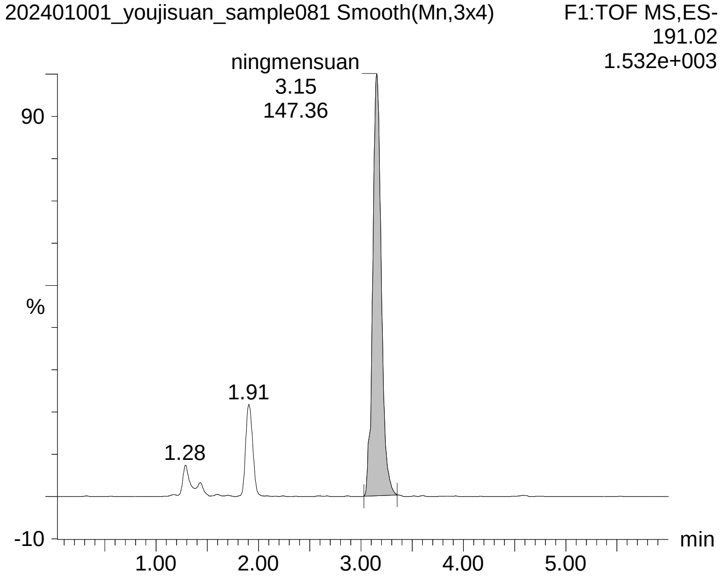

pinguosuan

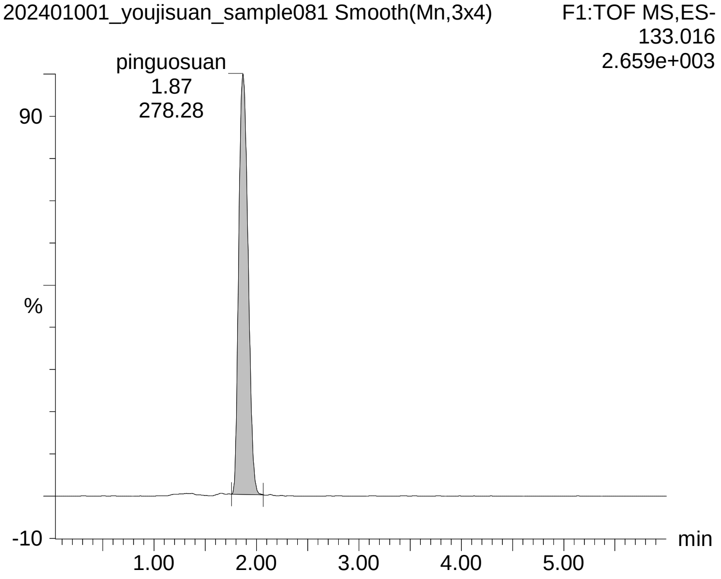

	#	Name	Sample Text	RT	Area	Std. Conc	Conc.
1	1	jiushisuan		1.50	1932.633		435.791993
2	2	ningmensuan		3.15	147.363		20.364245
3	3	pinguosuan		1.87	278.278		111.231608

Name: 202401001_youjisuan_sample082, Date: 02-Oct-2024, Time: 04:24:39, ID: , Description:

jiushisuan

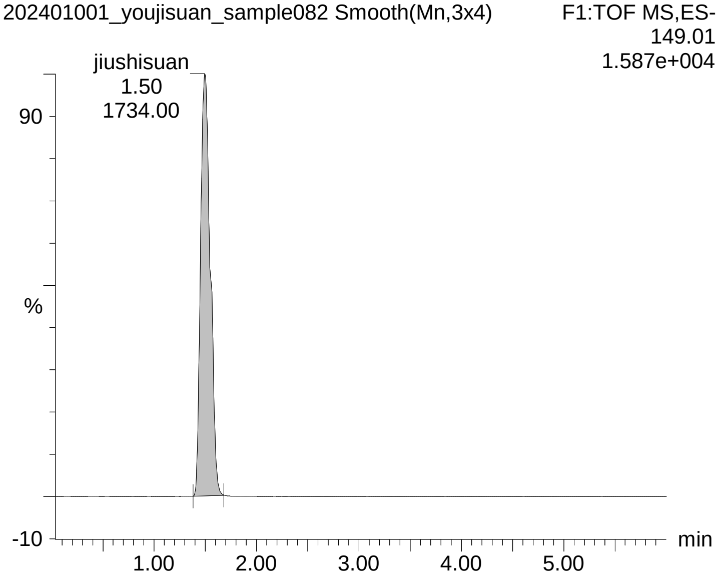

ningmensuan

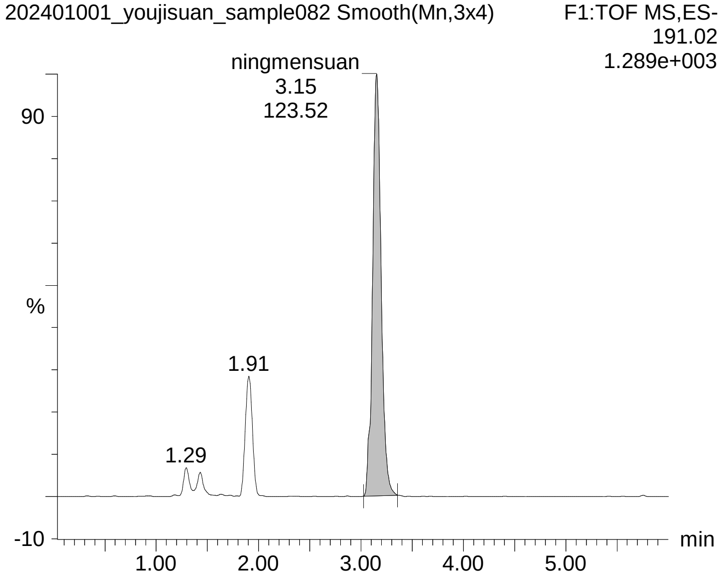

pinguosuan

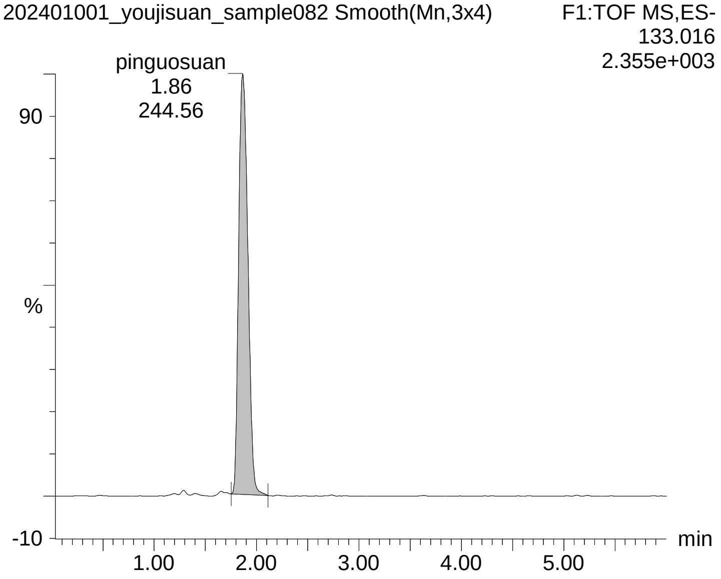

	#	Name	Sample Text	RT	Area	Std. Conc	Conc.
1	1	jiushisuan		1.50	1734.000		342.326939
2	2	ningmensuan		3.15	123.515		16.888623
3	3	pinguosuan		1.86	244.557		90.363100

Name: 202401001_youjisuan_sample083, Date: 02-Oct-2024, Time: 04:31:33, ID: , Description:

jiushisuan

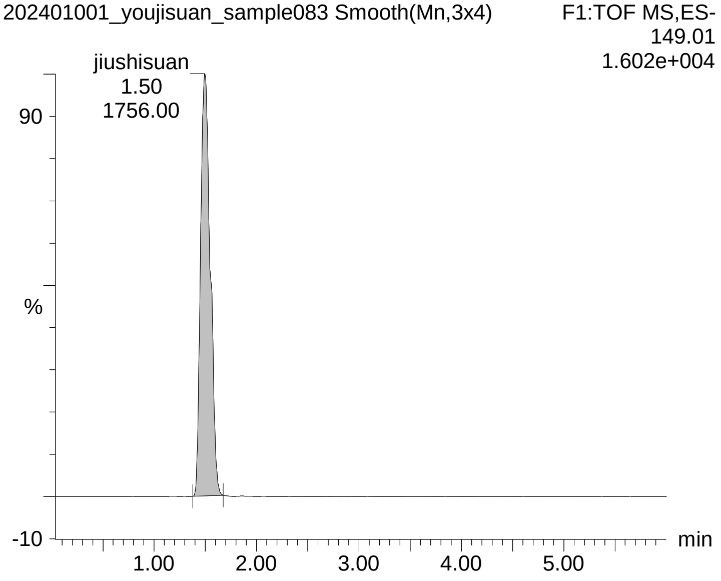

ningmensuan

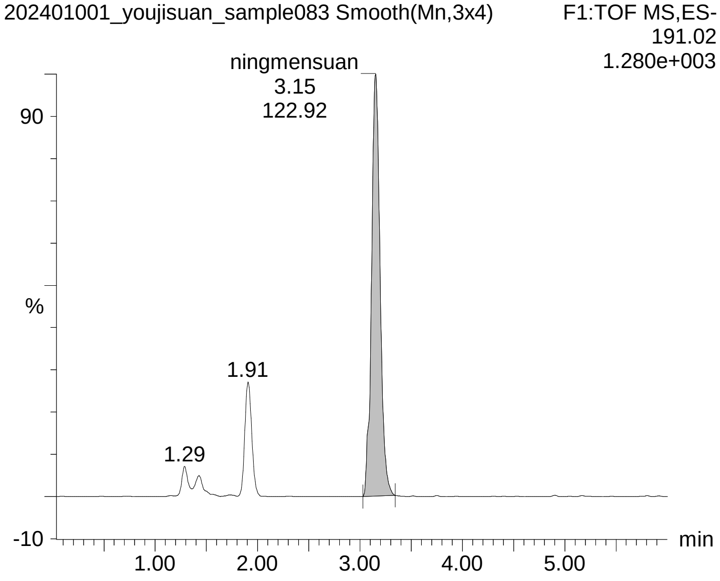

pinguosuan

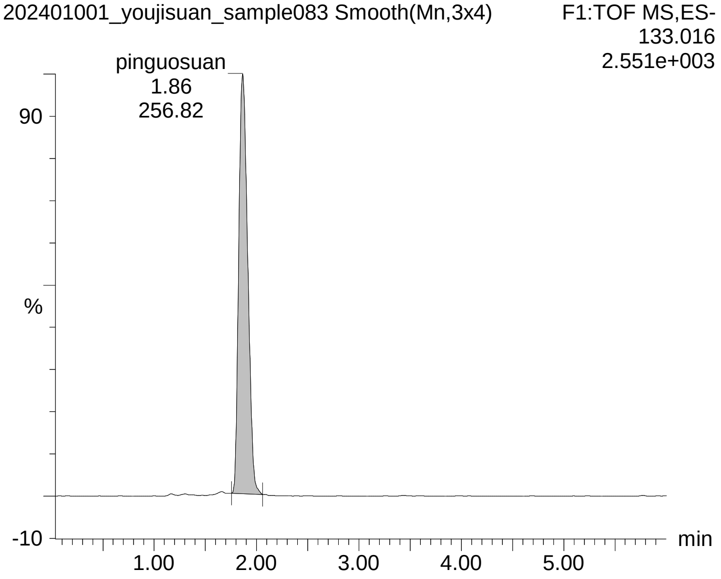

	#	Name	Sample Text	RT	Area	Std. Conc	Conc.
1	1	jiushisuan		1.50	1755.998		351.091132
2	2	ningmensuan		3.15	122.915		16.801179
3	3	pinguosuan		1.86	256.817		97.538840

Name: 202401001_youjisuan_sample084, Date: 02-Oct-2024, Time: 04:38:31, ID: , Description:

jiushisuan

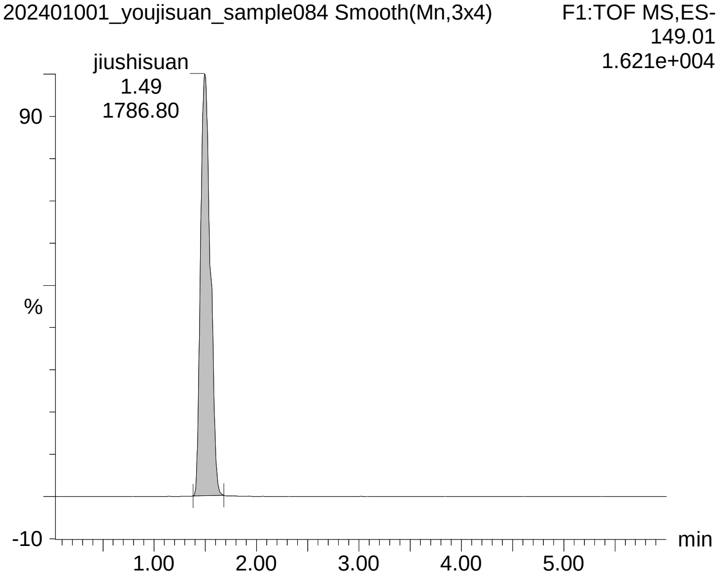

ningmensuan

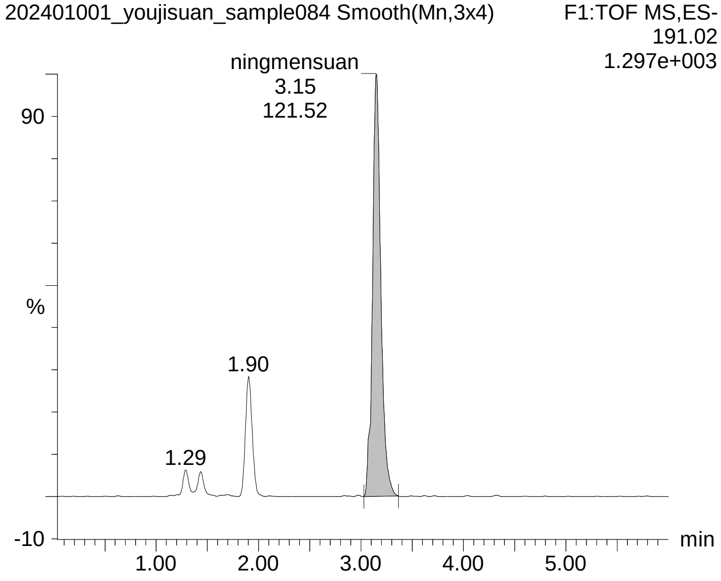

pinguosuan

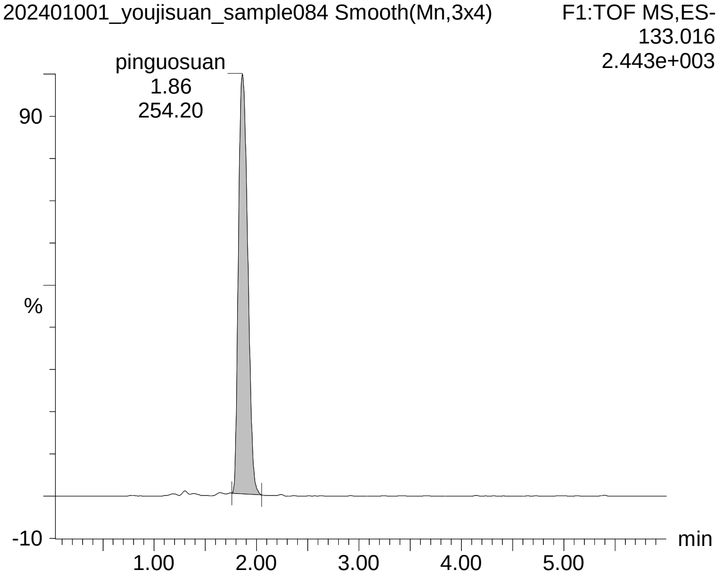

	#	Name	Sample Text	RT	Area	Std. Conc	Conc.
1	1	jiushisuan		1.49	1786.798		363.901469
2	2	ningmensuan		3.15	121.523		16.598308
3	3	pinguosuan		1.86	254.201		95.972125

project_wangzhonghua_BeiMu
Dataset: Untitled
Last Altered: Friday, October 11, 2024 15:45:23 China Standard Time
Printed: Friday, October 11, 2024 15:46:51 China Standard Time

Name: 202401001_youjisuan_sample085, Date: 02-Oct-2024, Time: 04:46:36, ID: , Description:

jiushisuan

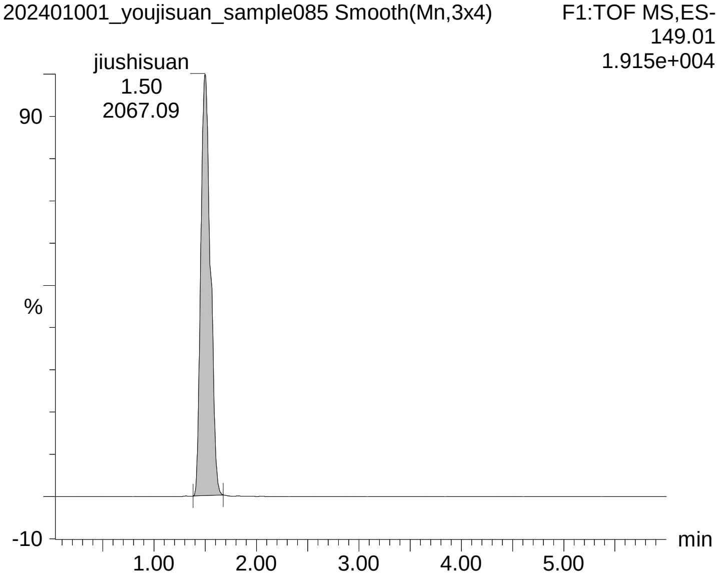

ningmensuan

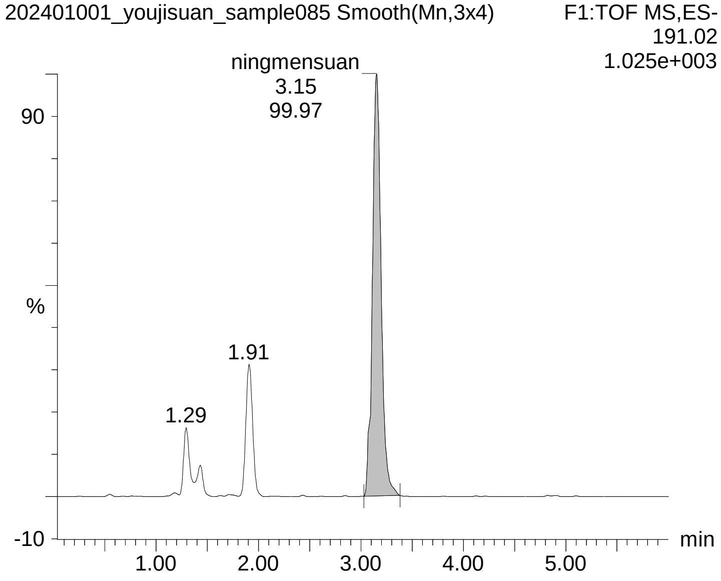

pinguosuan

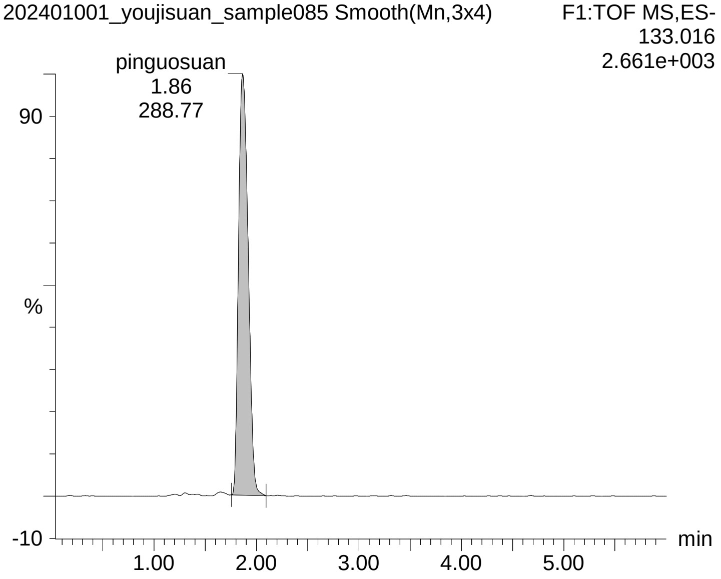

	#	Name	Sample Text	RT	Area	Std. Conc	Conc.
1	1	jiushisuan		1.50	2067.093		529.089946
2	2	ningmensuan		3.15	99.971		13.457306
3	3	pinguosuan		1.86	288.770		118.564064

Name: 202401001_youjisuan_sample086, Date: 02-Oct-2024, Time: 04:53:36, ID: , Description:

jiushisuan

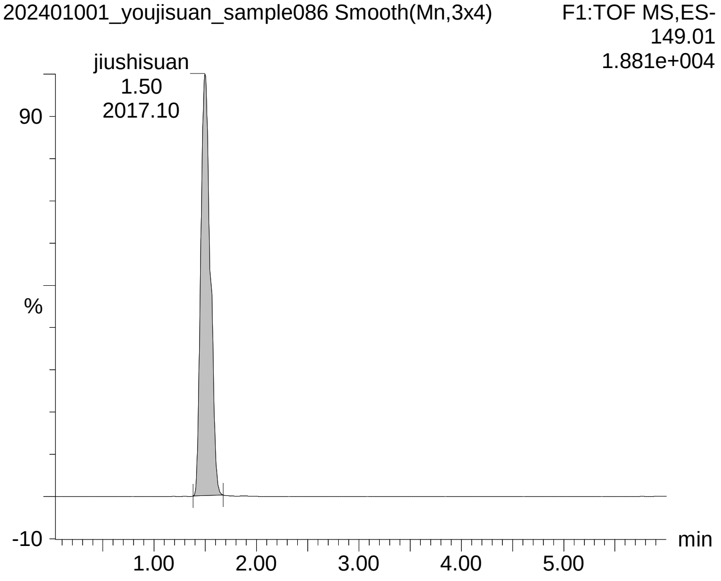

ningmensuan

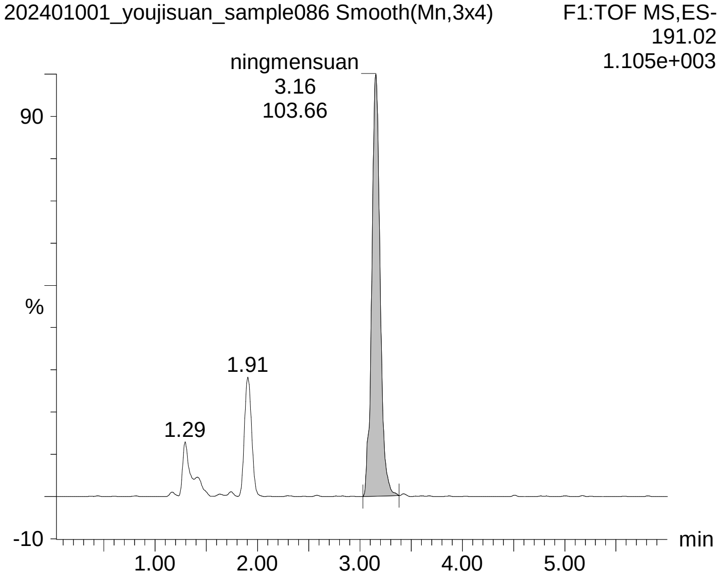

pinguosuan

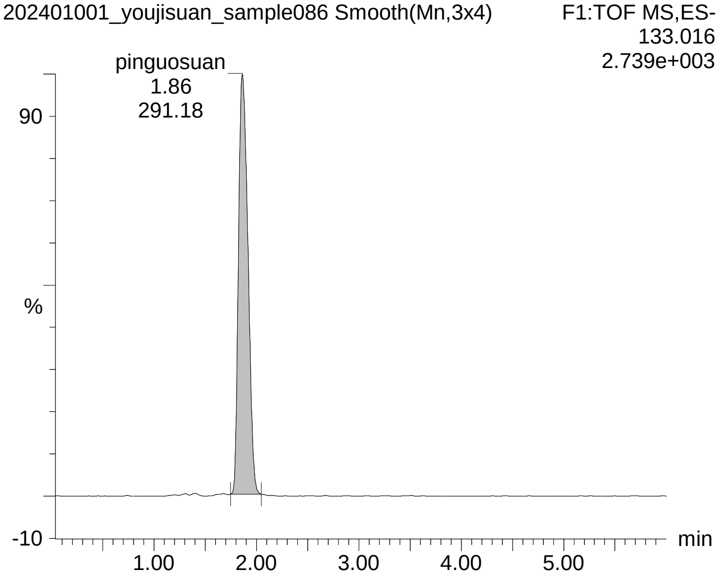

	#	Name	Sample Text	RT	Area	Std. Conc	Conc.
1	1	jiushisuan		1.50	2017.103		490.095666
2	2	ningmensuan		3.16	103.655		13.994215
3	3	pinguosuan		1.86	291.181		120.318826

Name: 202401001_youjisuan_sample087, Date: 02-Oct-2024, Time: 05:00:36, ID: , Description:

jiushisuan

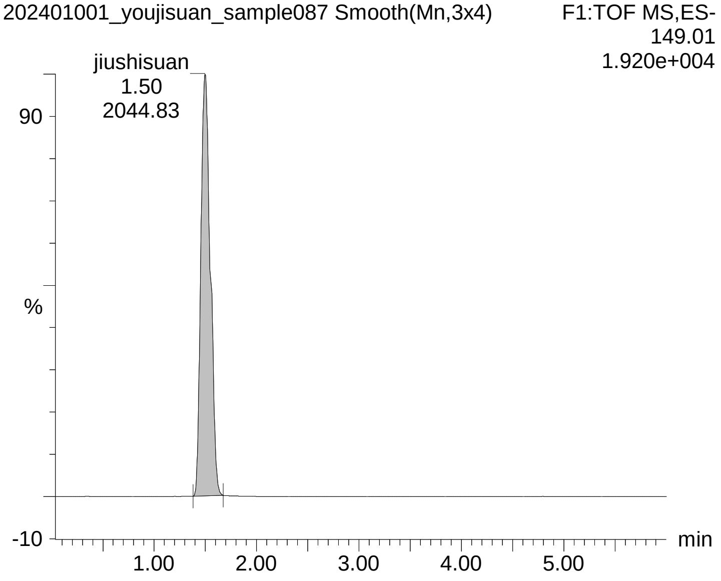

ningmensuan

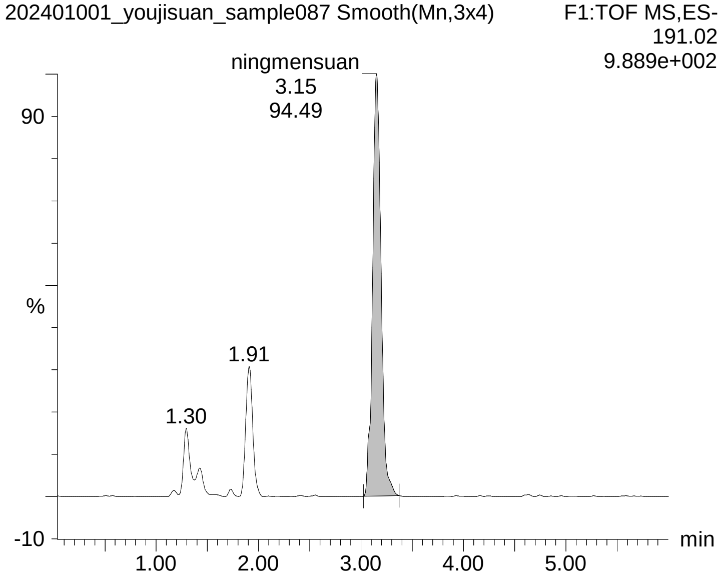

pinguosuan

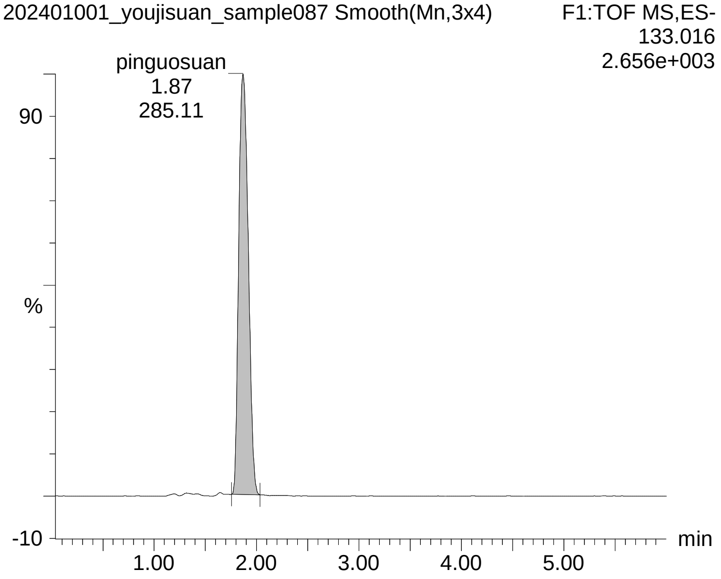

	#	Name	Sample Text	RT	Area	Std. Conc	Conc.
1	1	jiushisuan		1.50	2044.829		510.967767
2	2	ningmensuan		3.15	94.488		12.658210
3	3	pinguosuan		1.87	285.112		115.953292

Name: 202401001_youjisuan_sample088, Date: 02-Oct-2024, Time: 05:07:37, ID: , Description:

jiushisuan

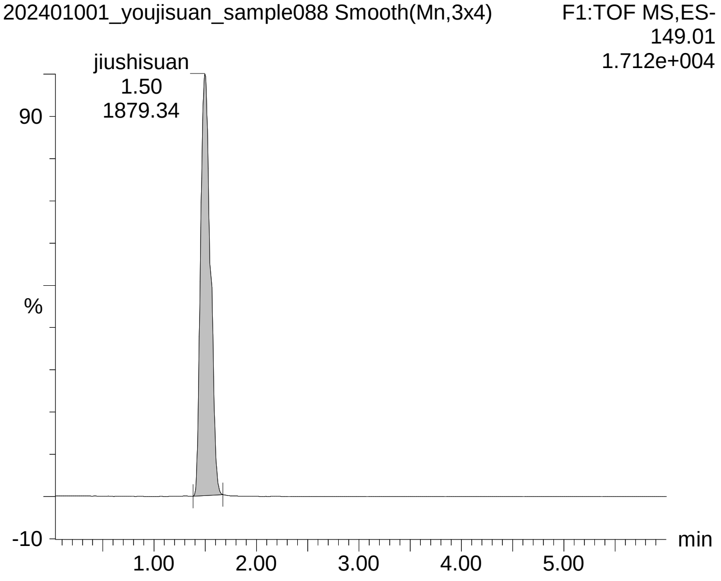

ningmensuan

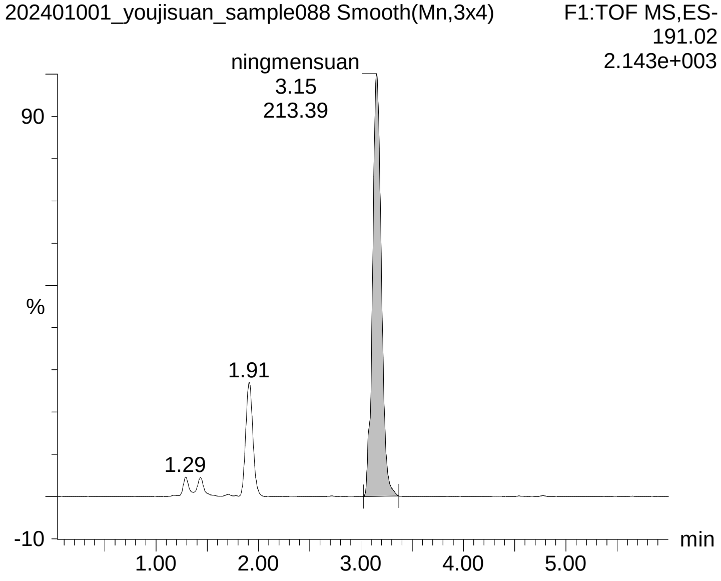

pinguosuan

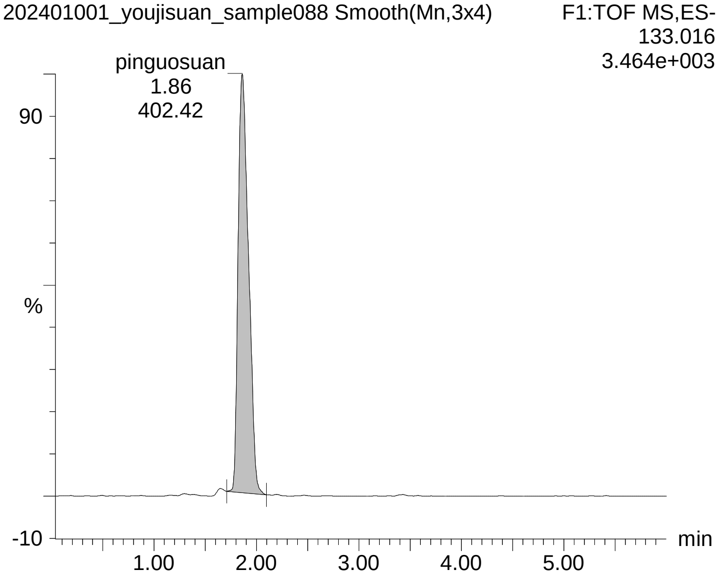

	#	Name	Sample Text	RT	Area	Std. Conc	Conc.
1	1	jiushisuan		1.50	1879.340		406.975548
2	2	ningmensuan		3.15	213.387		29.986623
3	3	pinguosuan		1.86	402.418		378.791573

project_wangzhonghua_BeiMu
Dataset: Untitled
Last Altered: Friday, October 11, 2024 16:58:58 China Standard Time
Printed: Friday, October 11, 2024 16:59:13 China Standard Time

Method: F:\data\Wu_yueyan.PRO\MethDB\20241011_ organic acid .mdb 11 Oct 2024 15:52:39
Calibration: F:\data\zhanghuien.PRO\CurveDB\20241011_organic acid003.cdb 11 Oct 2024 15:39:56

Compound name: jiushisuan
Coefficient of Determination: R^2 = 0.994685
Calibration curve: 4.94514e-006 * x^3 + -0.0101268 * x^2 + 7.74871 * x + 69.7696
Response type: External Std, Area
Curve type: 3rd Order, Origin: Include, Weighting: Null, Axis trans: None

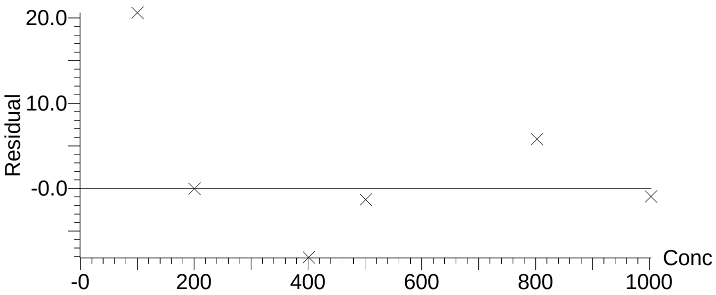

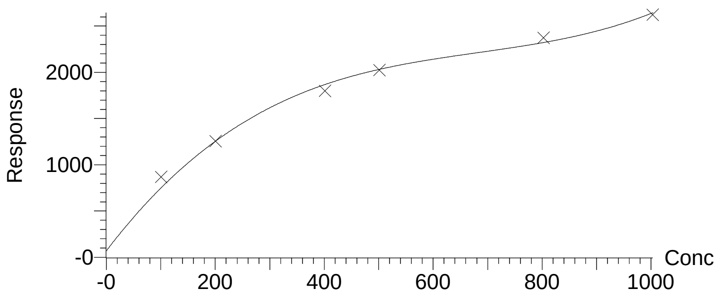

Compound name: ningmensuan
Correlation coefficient: r = 0.998196, r^2 = 0.996396
Calibration curve: 6.86151 * x + 7.63362
Response type: External Std, Area
Curve type: Linear, Origin: Include, Weighting: Null, Axis trans: None

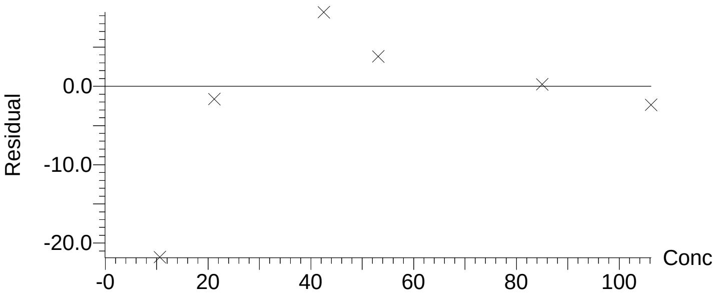

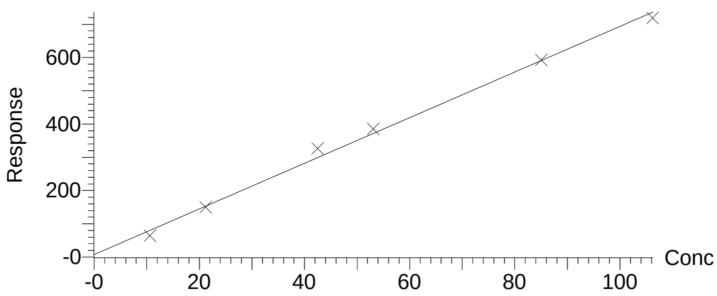

Compound name: pinguosuan
Coefficient of Determination: R^2 = 0.984709
Calibration curve: 9.85928e-006 * x^3 + -0.00971593 * x^2 + 3.27297 * x + 20.8616
Response type: External Std, Area
Curve type: 3rd Order, Origin: Include, Weighting: Null, Axis trans: None

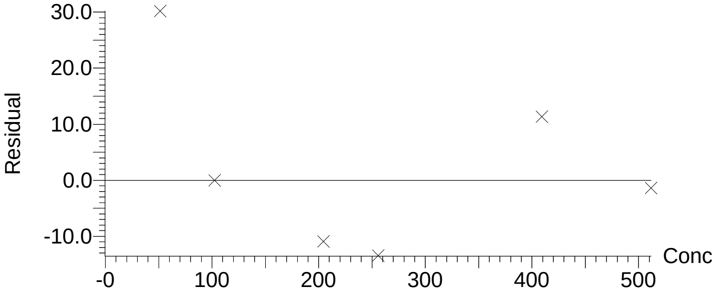

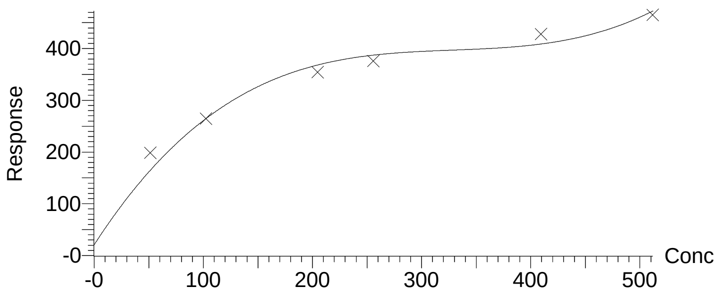

Compound name: huposaun
Coefficient of Determination: R^2 = 0.999608
Calibration curve: 3.18016e-005 * x^3 + -0.0146291 * x^2 + 2.726 * x + 0.728245
Response type: External Std, Area
Curve type: 3rd Order, Origin: Include, Weighting: Null, Axis trans: None

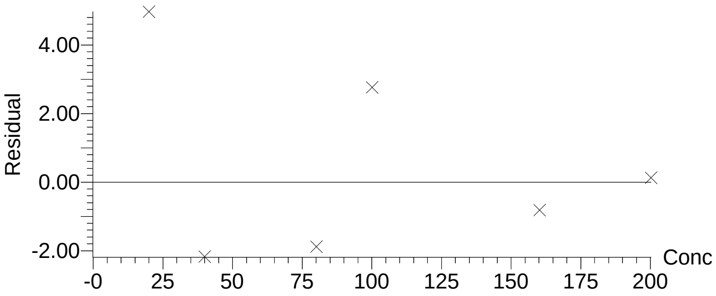

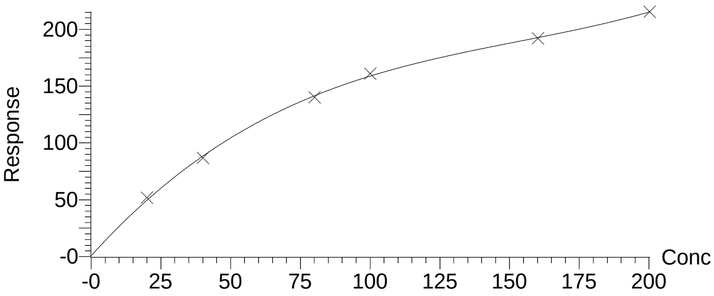

Method: F:\data\Wu_yueyan.PRO\MethDB\20241011_ organic acid .mdb 11 Oct 2024 15:52:39
Calibration: F:\data\zhanghuien.PRO\CurveDB\20241011_organic acid003.cdb 11 Oct 2024 15:39:56

Name: 202401011_youjisuan_sample089, Date: 11-Oct-2024, Time: 09:37:49, ID: , Description:

jiushisuan

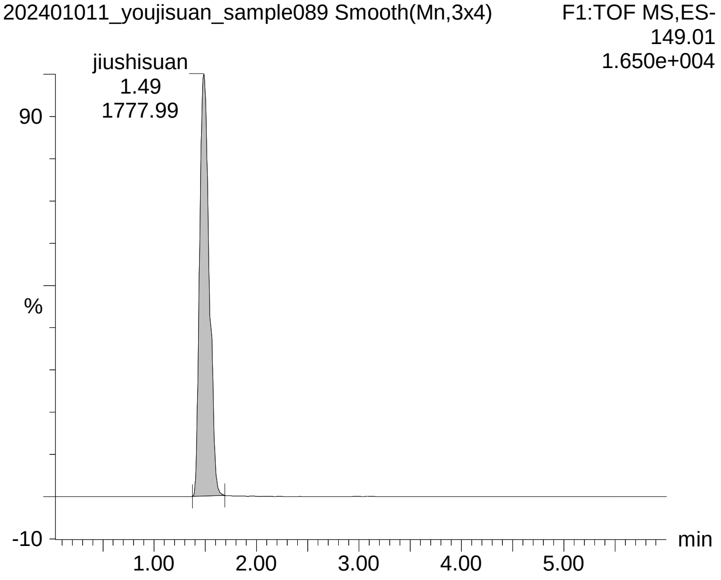

ningmensuan

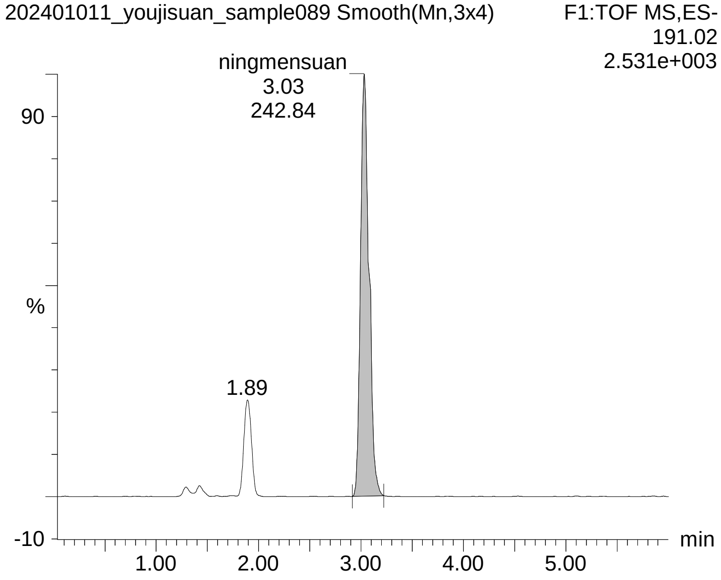

pinguosuan

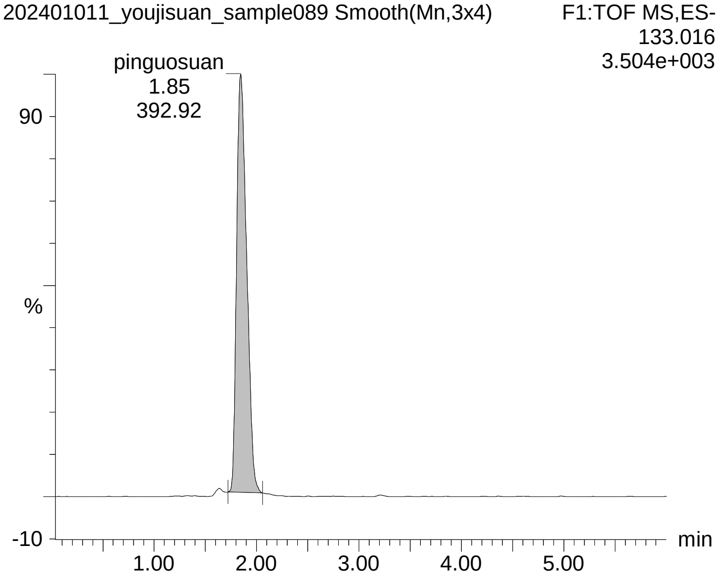

	#	Name	Sample Text	RT	Area	Std. Conc	Conc.
1	1	jiushisuan		1.49	1777.993		360.171535
2	2	ningmensuan		3.03	242.836		34.278538
3	3	pinguosuan		1.85	392.920		286.506601

project_wangzhonghua_BeiMu
Dataset: Untitled
Last Altered: Friday, October 11, 2024 16:58:58 China Standard Time
Printed: Friday, October 11, 2024 16:59:13 China Standard Time

Name: 202401011_youjisuan_sample090, Date: 11-Oct-2024, Time: 09:44:46, ID: , Description:

jiushisuan

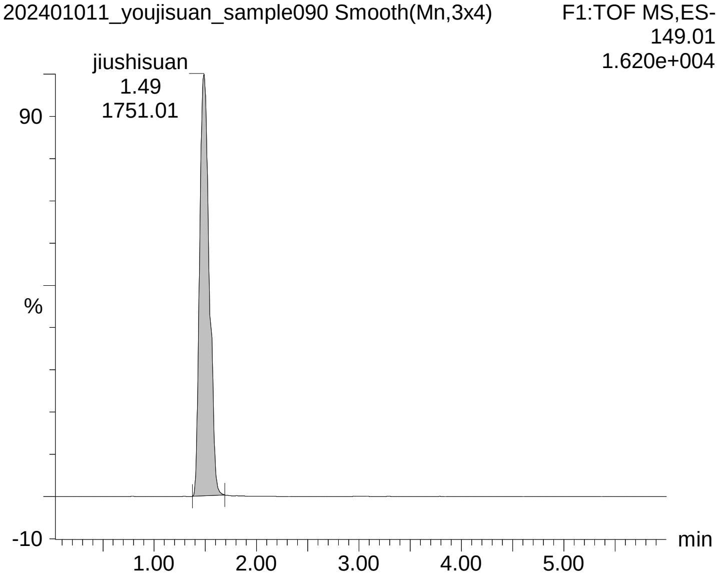

ningmensuan

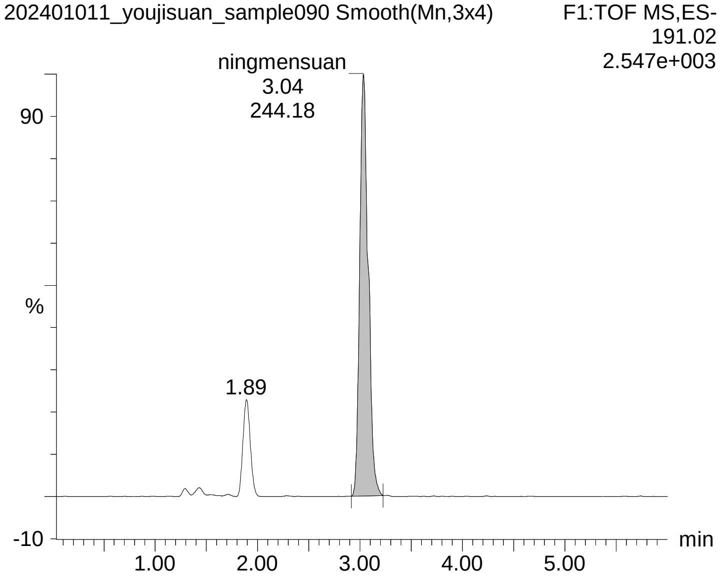

pinguosuan

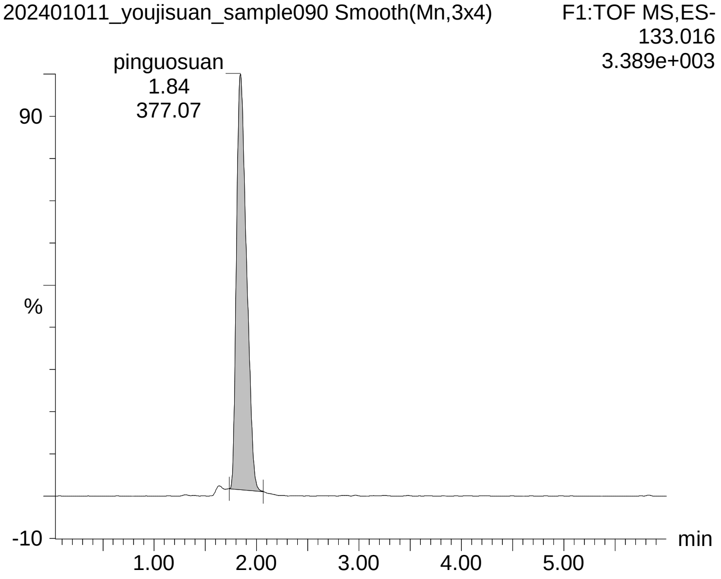

	#	Name	Sample Text	RT	Area	Std. Conc	Conc.
1	1	jiushisuan		1.49	1751.007		349.075734
2	2	ningmensuan		3.04	244.175		34.473685
3	3	pinguosuan		1.84	377.073		223.407802

project_wangzhonghua_BeiMu
Dataset: Untitled
Last Altered: Friday, October 11, 2024 16:58:58 China Standard Time
Printed: Friday, October 11, 2024 16:59:13 China Standard Time

Name: 202401011_youjisuan_sample091, Date: 11-Oct-2024, Time: 09:51:46, ID: , Description:

jiushisuan

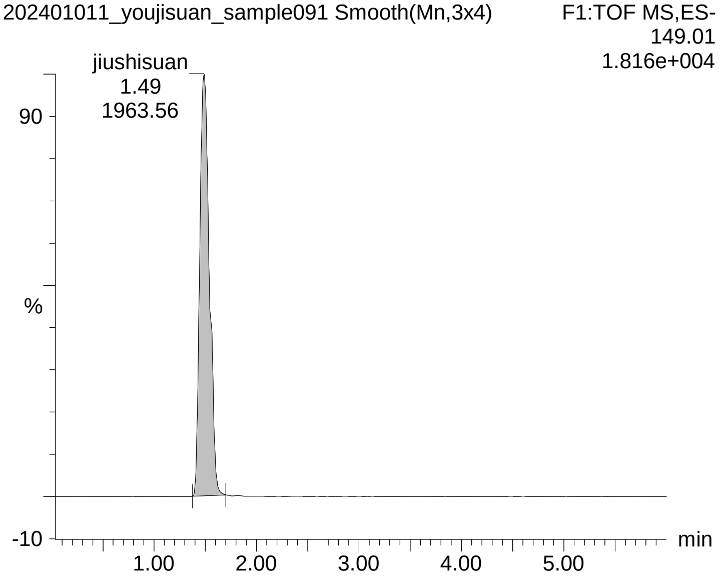

ningmensuan

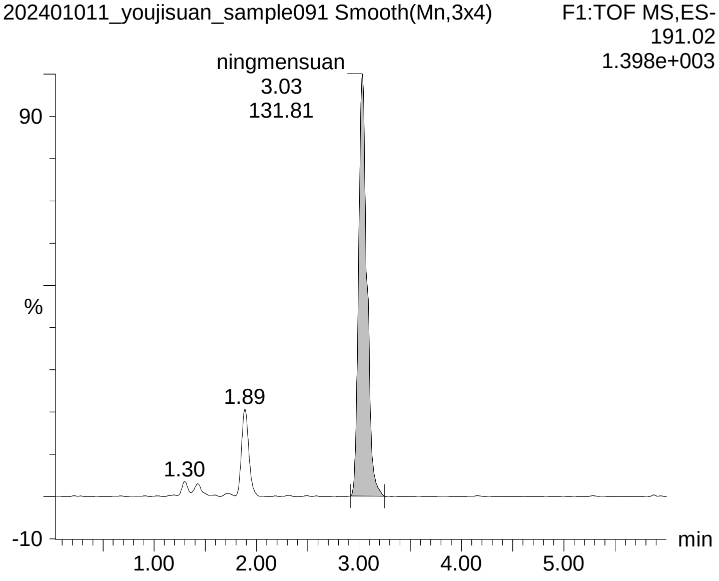

pinguosuan

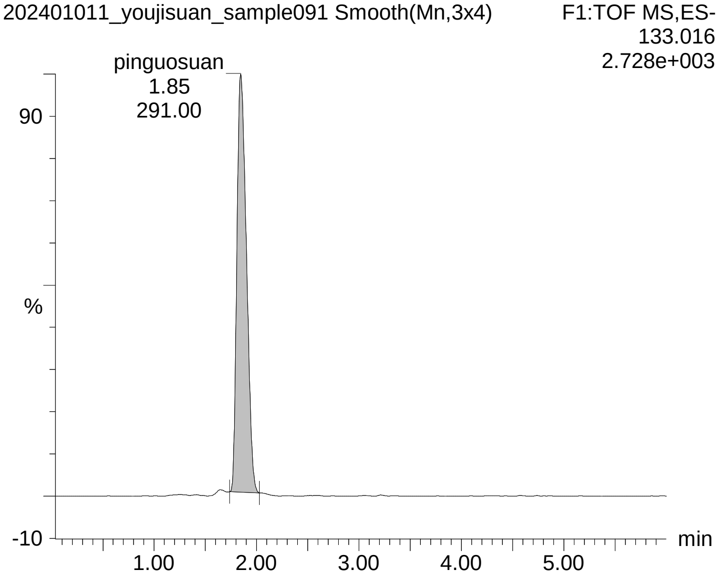

	#	Name	Sample Text	RT	Area	Std. Conc	Conc.
1	1	jiushisuan		1.49	1963.559		454.268455
2	2	ningmensuan		3.03	131.808		18.097250
3	3	pinguosuan		1.85	290.995		120.182460

project_wangzhonghua_BeiMu
Dataset: Untitled
Last Altered: Friday, October 11, 2024 16:58:58 China Standard Time
Printed: Friday, October 11, 2024 16:59:13 China Standard Time

Name: 202401011_youjisuan_sample092, Date: 11-Oct-2024, Time: 09:58:46, ID: , Description:

jiushisuan

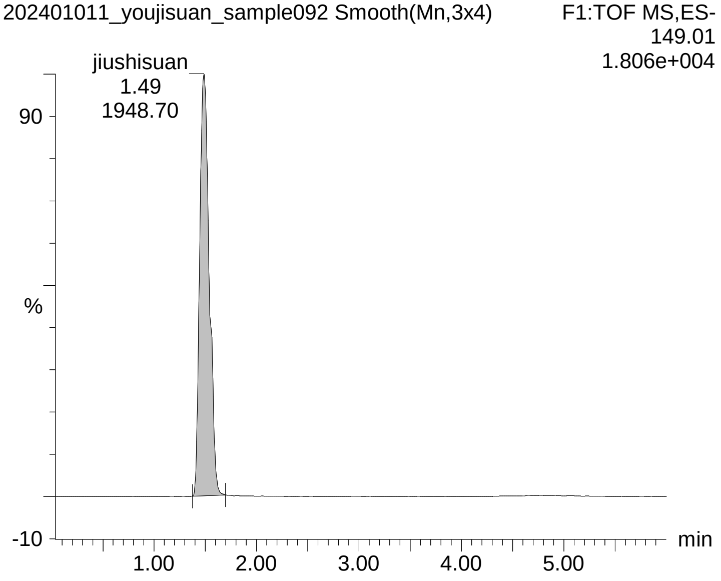

ningmensuan

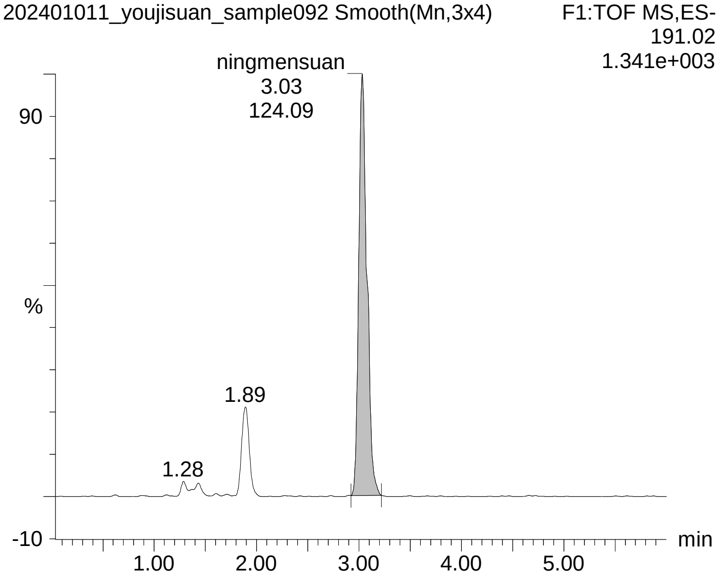

pinguosuan

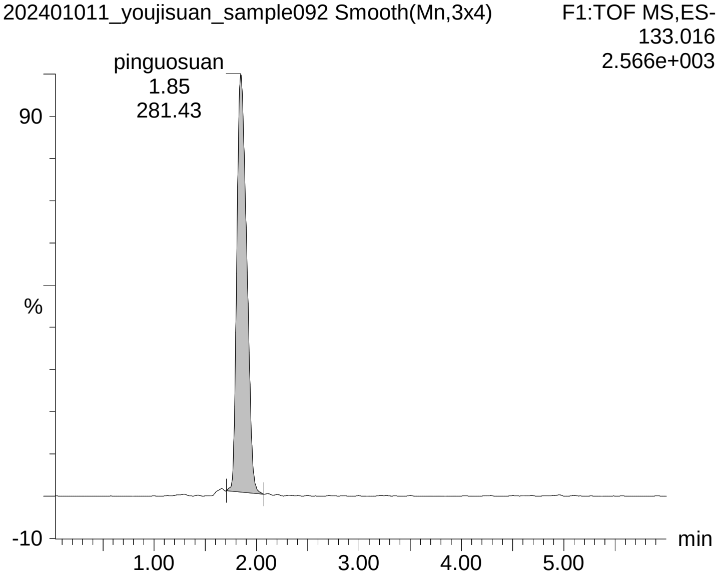

	#	Name	Sample Text	RT	Area	Std. Conc	Conc.
1	1	jiushisuan		1.49	1948.700		445.211477
2	2	ningmensuan		3.03	124.087		16.971987
3	3	pinguosuan		1.85	281.425		113.381615

project_wangzhonghua_BeiMu
Dataset: Untitled
Last Altered: Friday, October 11, 2024 16:58:58 China Standard Time
Printed: Friday, October 11, 2024 16:59:13 China Standard Time

Name: 202401011_youjisuan_sample093, Date: 11-Oct-2024, Time: 10:05:46, ID: , Description:

jiushisuan

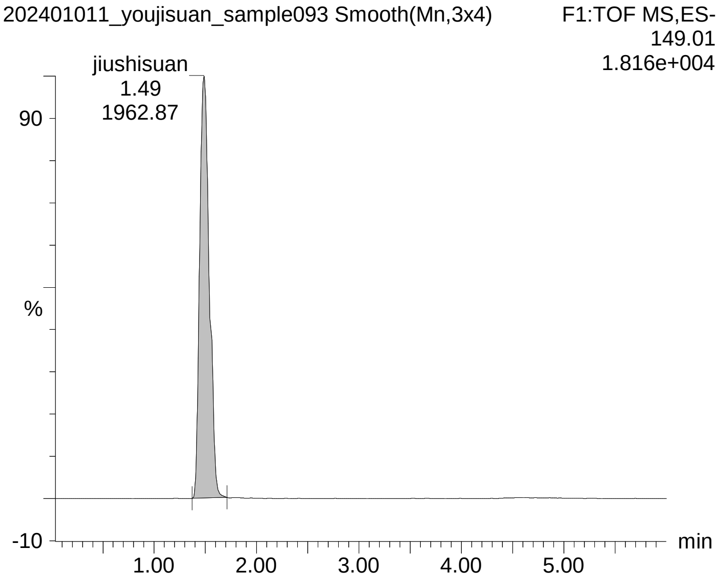

ningmensuan

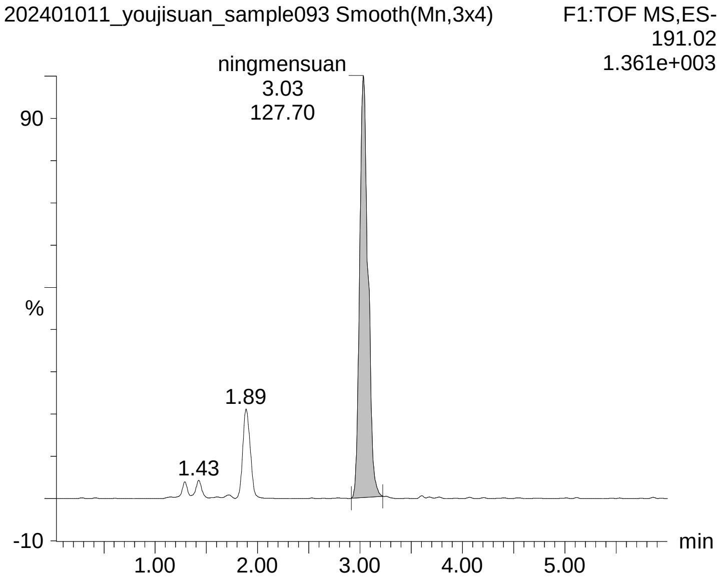

pinguosuan

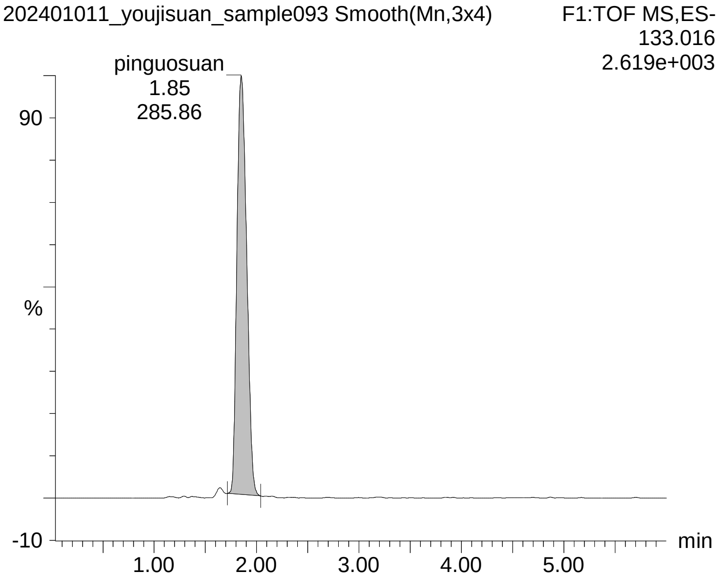

	#	Name	Sample Text	RT	Area	Std. Conc	Conc.
1	1	jiushisuan		1.49	1962.868		453.839524
2	2	ningmensuan		3.03	127.702		17.498839
3	3	pinguosuan		1.85	285.855		116.478691

Name: 202401011_youjisuan_sample094, Date: 11-Oct-2024, Time: 10:12:46, ID: , Description:

jiushisuan

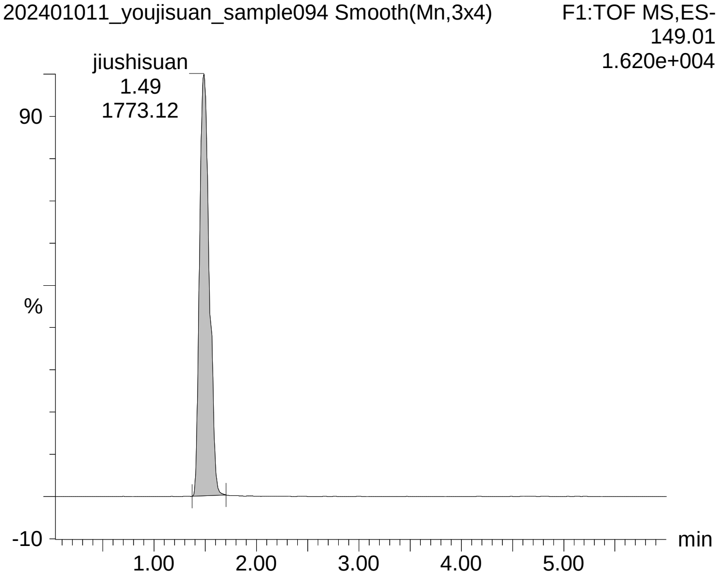

ningmensuan

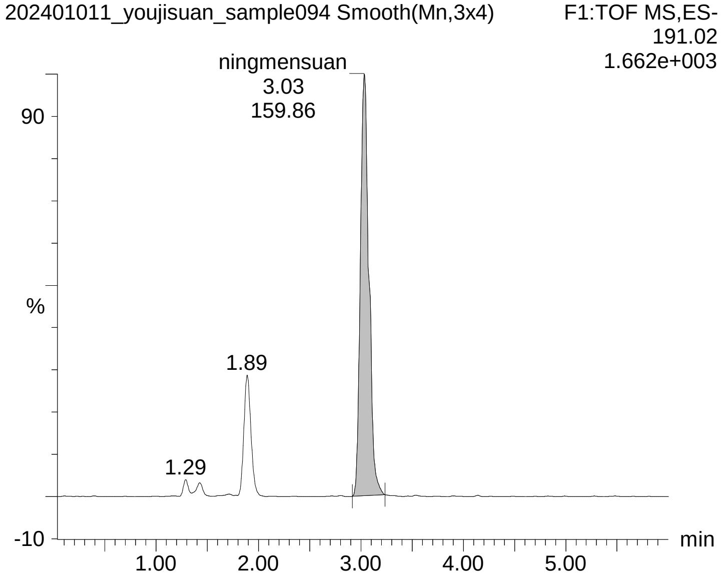

pinguosuan

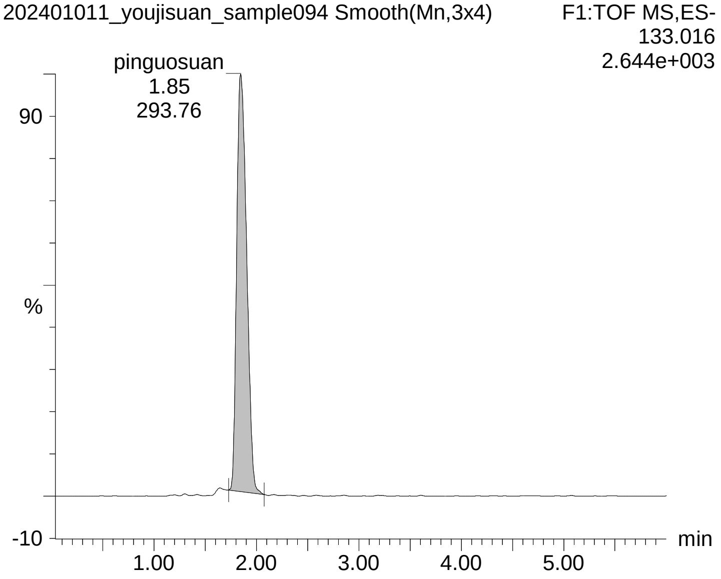

	#	Name	Sample Text	RT	Area	Std. Conc	Conc.
1	1	jiushisuan		1.49	1773.123		358.132325
2	2	ningmensuan		3.03	159.863		22.186003
3	3	pinguosuan		1.85	293.761		122.227926

project_wangzhonghua_BeiMu
Dataset: Untitled
Last Altered: Friday, October 11, 2024 16:58:58 China Standard Time
Printed: Friday, October 11, 2024 16:59:13 China Standard Time

Name: 202401011_youjisuan_sample095, Date: 11-Oct-2024, Time: 10:19:46, ID: , Description:

jiushisuan

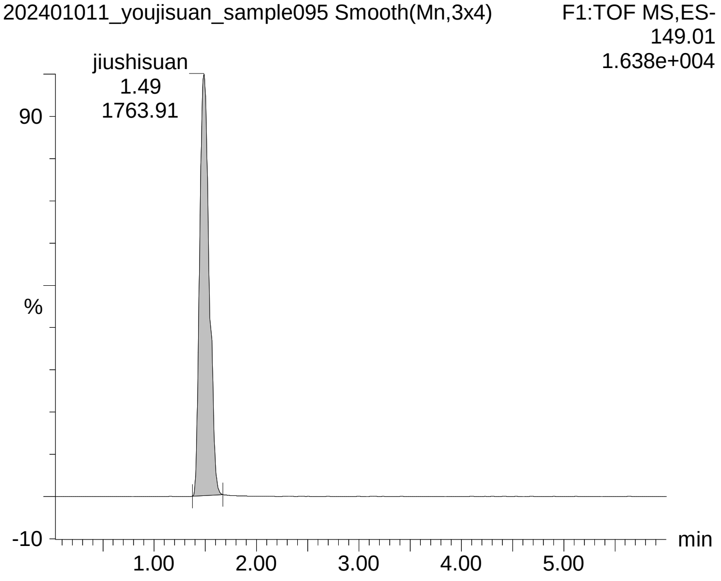

ningmensuan

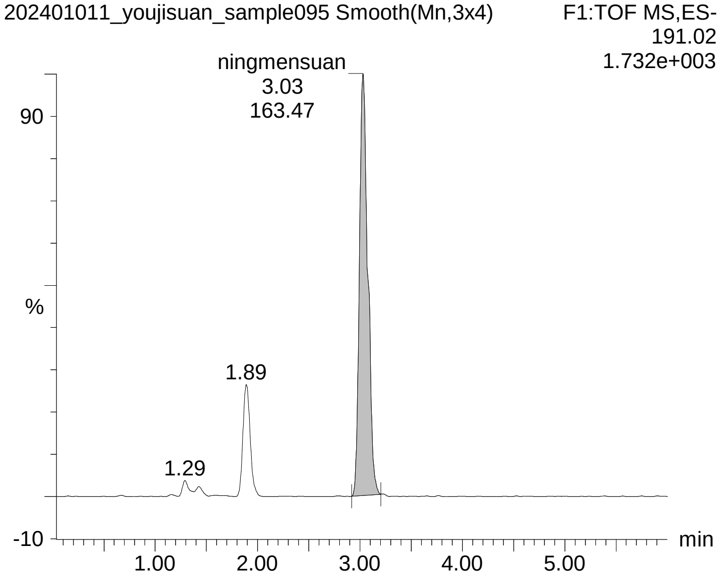

pinguosuan

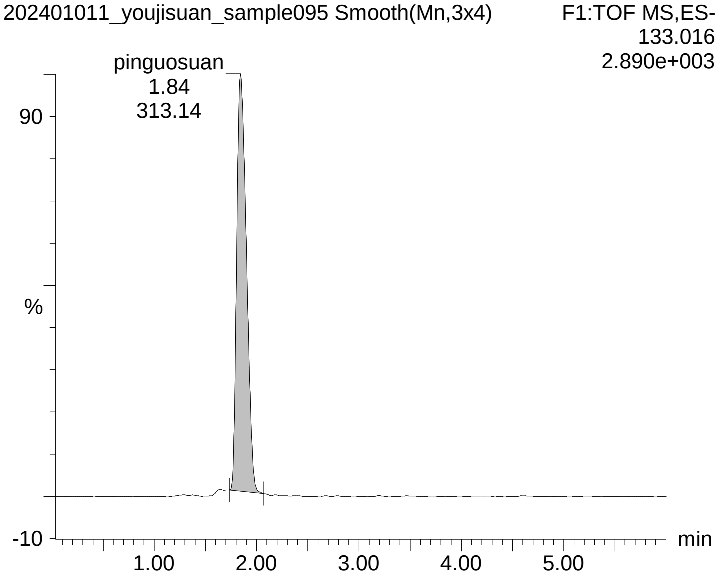

	#	Name	Sample Text	RT	Area	Std. Conc	Conc.
1	1	jiushisuan		1.49	1763.914		354.321274
2	2	ningmensuan		3.03	163.468		22.711398
3	3	pinguosuan		1.84	313.137		137.761633

project_wangzhonghua_BeiMu
Dataset: Untitled
Last Altered: Friday, October 11, 2024 17:02:46 China Standard Time
Printed: Friday, October 11, 2024 17:03:06 China Standard Time

Method: F:\data\Wu_yueyan.PRO\MethDB\20241011_ organic acid .mdb 11 Oct 2024 15:52:39
Calibration: F:\data\zhanghuien.PRO\CurveDB\20241011_organic acid003.cdb 11 Oct 2024 15:39:56

Compound name: jiushisuan
Coefficient of Determination: R^2 = 0.994685
Calibration curve: 4.94514e-006 * x^3 + -0.0101268 * x^2 + 7.74871 * x + 69.7696
Response type: External Std, Area
Curve type: 3rd Order, Origin: Include, Weighting: Null, Axis trans: None

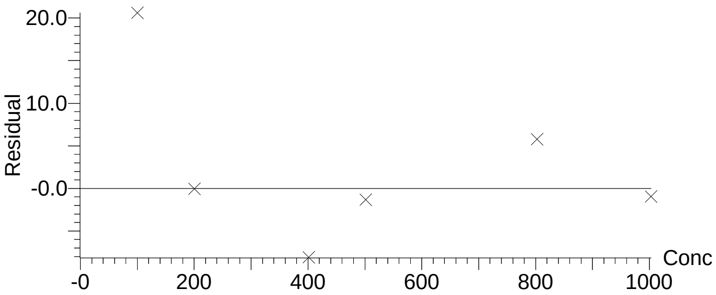

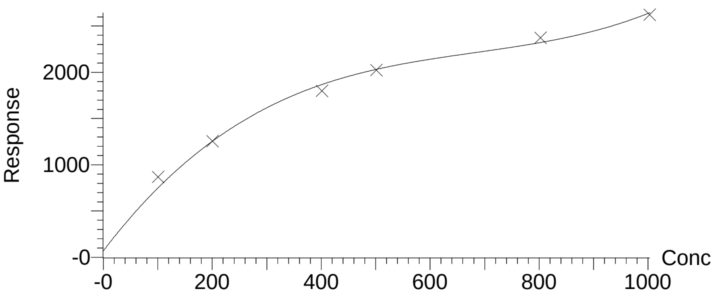

Compound name: ningmensuan
Correlation coefficient: r = 0.998196, r^2 = 0.996396
Calibration curve: 6.86151 * x + 7.63362
Response type: External Std, Area
Curve type: Linear, Origin: Include, Weighting: Null, Axis trans: None

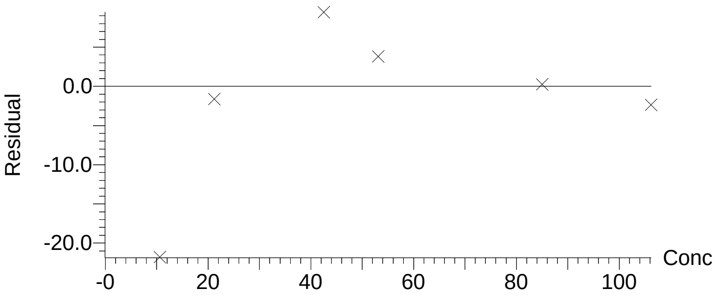

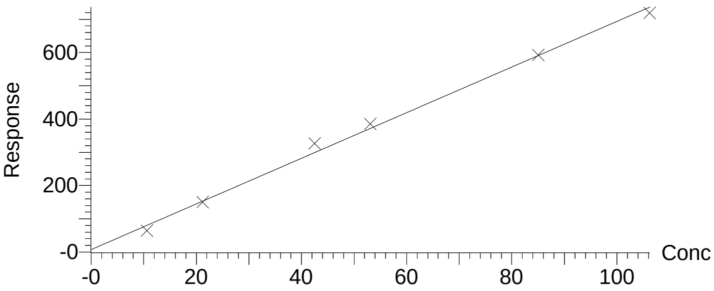

Compound name: pinguosuan
Coefficient of Determination: R^2 = 0.984709
Calibration curve: 9.85928e-006 * x^3 + -0.00971593 * x^2 + 3.27297 * x + 20.8616
Response type: External Std, Area
Curve type: 3rd Order, Origin: Include, Weighting: Null, Axis trans: None

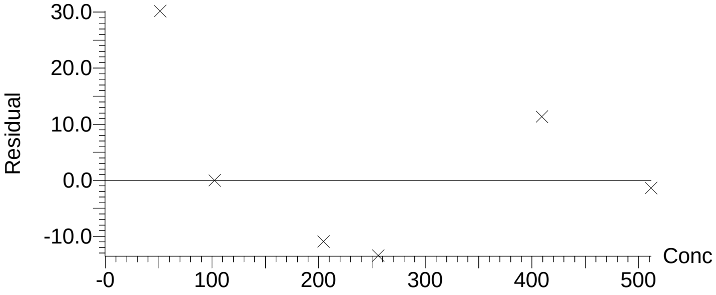

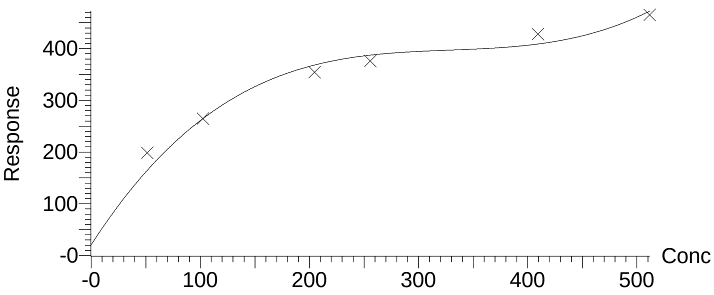

Compound name: huposaun
Coefficient of Determination: R^2 = 0.999608
Calibration curve: 3.18016e-005 * x^3 + -0.0146291 * x^2 + 2.726 * x + 0.728245
Response type: External Std, Area
Curve type: 3rd Order, Origin: Include, Weighting: Null, Axis trans: None

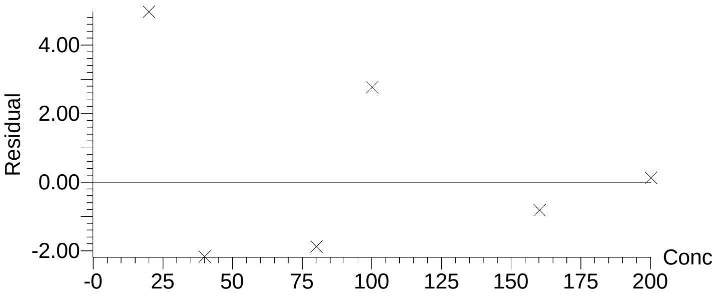

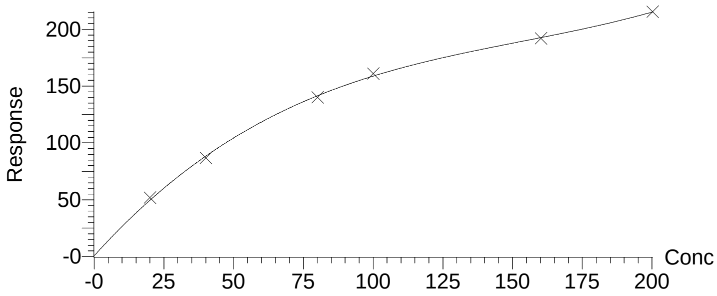

Method: F:\data\Wu_yueyan.PRO\MethDB\20241011_ organic acid .mdb 11 Oct 2024 15:52:39
Calibration: F:\data\zhanghuien.PRO\CurveDB\20241011_organic acid003.cdb 11 Oct 2024 15:39:56

Name: 202401011_youjisuan_sample096, Date: 11-Oct-2024, Time: 10:26:47, ID: , Description:

jiushisuan

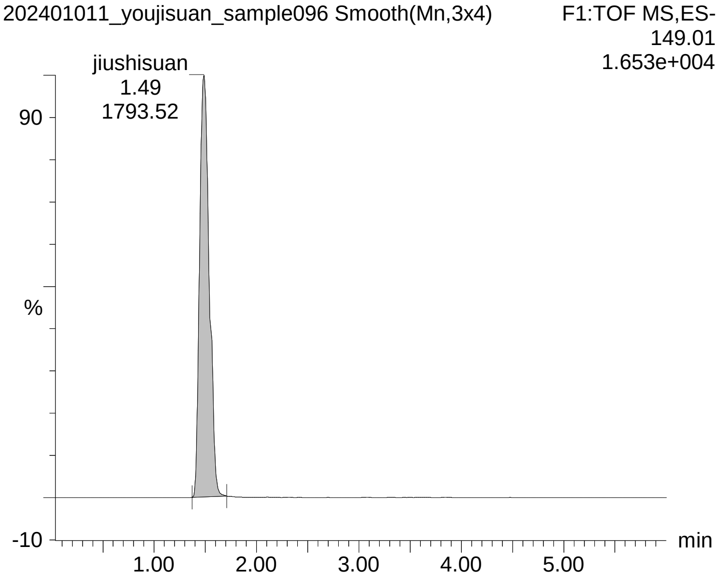

ningmensuan

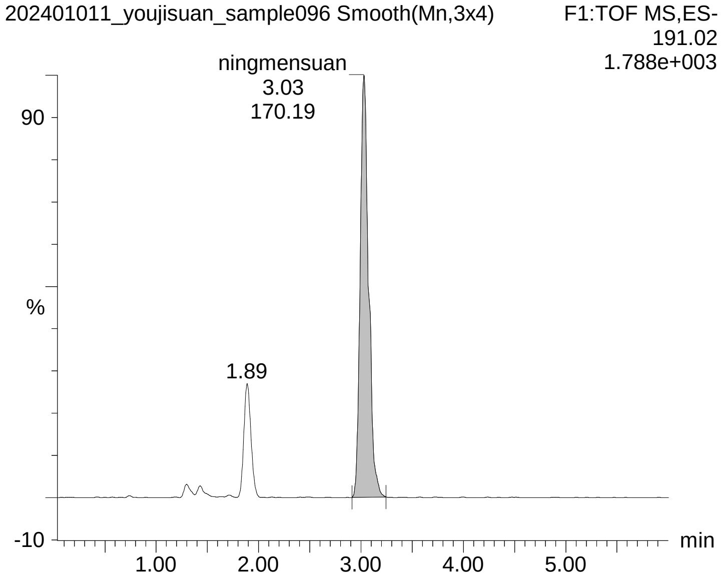

pinguosuan

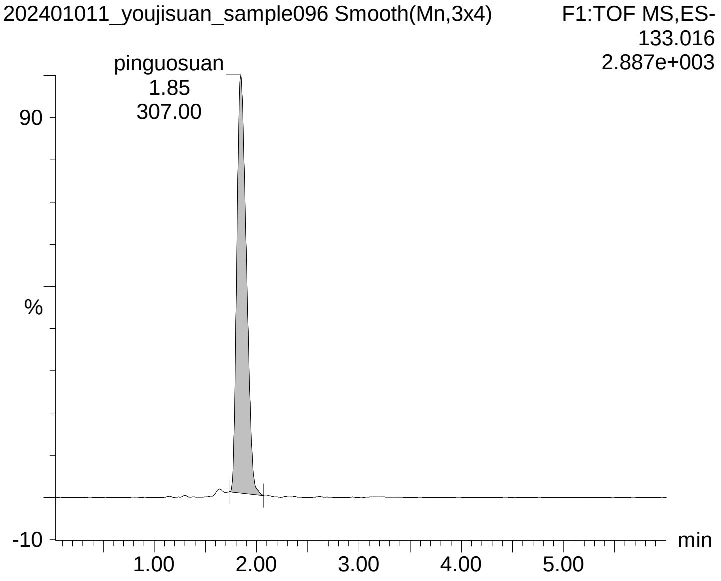

	#	Name	Sample Text	RT	Area	Std. Conc	Conc.
1	1	jiushisuan		1.49	1793.520		366.787286
2	2	ningmensuan		3.03	170.189		23.690920
3	3	pinguosuan		1.85	306.998		132.588848

Name: 202401011_youjisuan_sample097, Date: 11-Oct-2024, Time: 10:34:45, ID: , Description:

jiushisuan

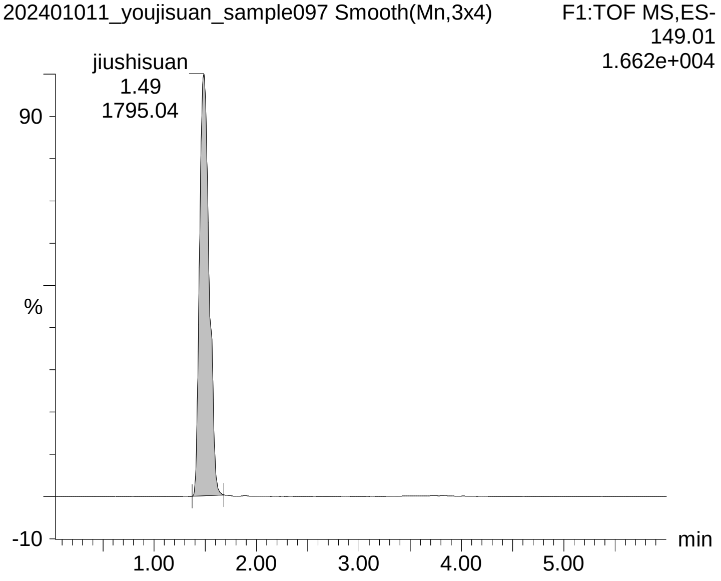

ningmensuan

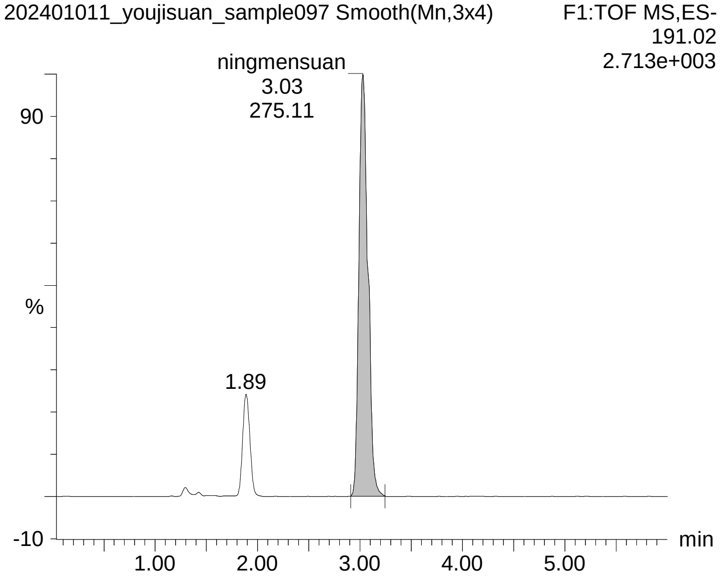

pinguosuan

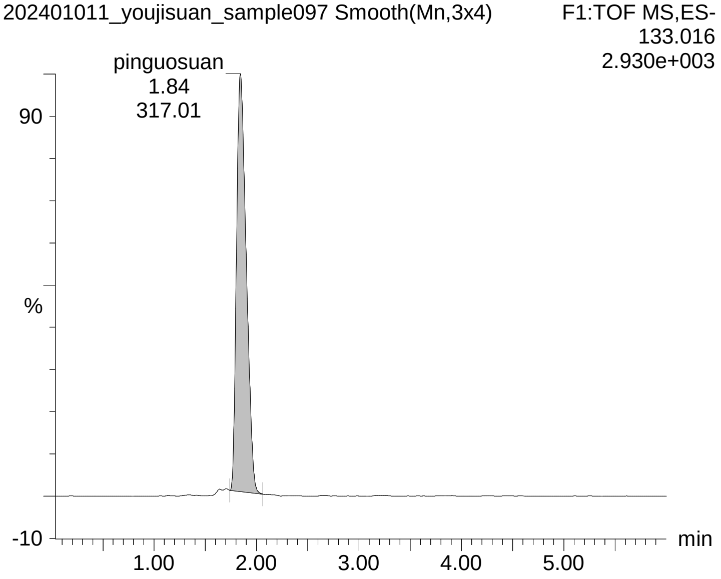

	#	Name	Sample Text	RT	Area	Std. Conc	Conc.
1	1	jiushisuan		1.49	1795.035		367.442367
2	2	ningmensuan		3.03	275.114		38.982754
3	3	pinguosuan		1.84	317.009		141.163292

project_wangzhonghua_BeiMu
Dataset: Untitled
Last Altered: Friday, October 11, 2024 17:02:46 China Standard Time
Printed: Friday, October 11, 2024 17:03:06 China Standard Time

Name: 202401011_youjisuan_sample098, Date: 11-Oct-2024, Time: 10:41:44, ID: , Description:

jiushisuan

202401011_youjisuan_sample098 Smooth(Mn,3x4) F1:TOF MS,ES- 149.01 1.643e+004

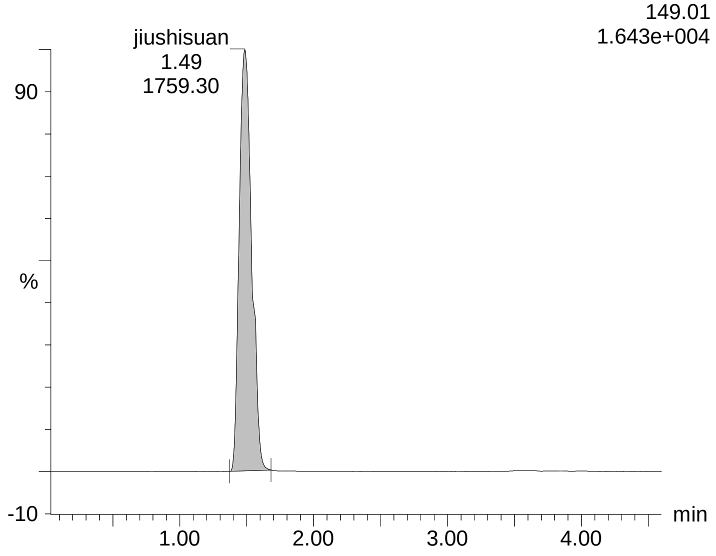

ningmensuan

202401011_youjisuan_sample098 Smooth(Mn,3x4) F1:TOF MS,ES- 191.02 2.807e+003

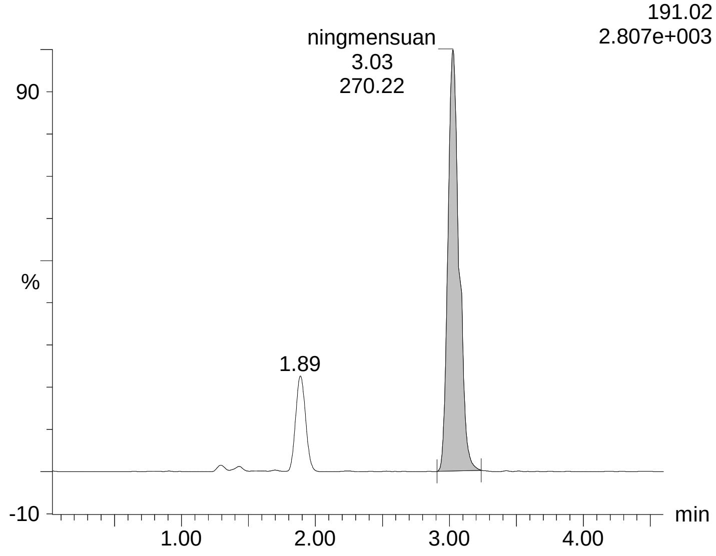

pinguosuan

202401011_youjisuan_sample098 Smooth(Mn,3x4) F1:TOF MS,ES- 133.016 2.989e+003

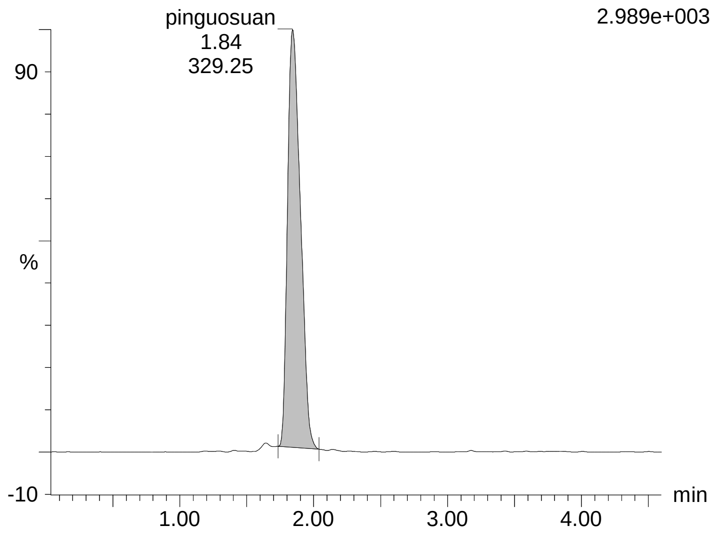

	#	Name	Sample Text	RT	Area	Std. Conc	Conc.
1	1	jiushisuan		1.49	1759.297		352.432235
2	2	ningmensuan		3.03	270.216		38.268916
3	3	pinguosuan		1.84	329.250		152.750425

project_wangzhonghua_BeiMu
Dataset: Untitled
Last Altered: Saturday, October 12, 2024 07:48:57 China Standard Time
Printed: Saturday, October 12, 2024 07:49:23 China Standard Time

Method: F:\data\Wu_yueyan.PRO\MethDB\20241011_ organic acid .mdb 11 Oct 2024 15:52:39
Calibration: F:\data\zhanghuien.PRO\CurveDB\20241011_organic acid003.cdb 11 Oct 2024 15:39:56

Compound name: jiushisuan
Coefficient of Determination: R^2 = 0.994685
Calibration curve: 4.94514e-006 * x^3 + -0.0101268 * x^2 + 7.74871 * x + 69.7696
Response type: External Std, Area
Curve type: 3rd Order, Origin: Include, Weighting: Null, Axis trans: None

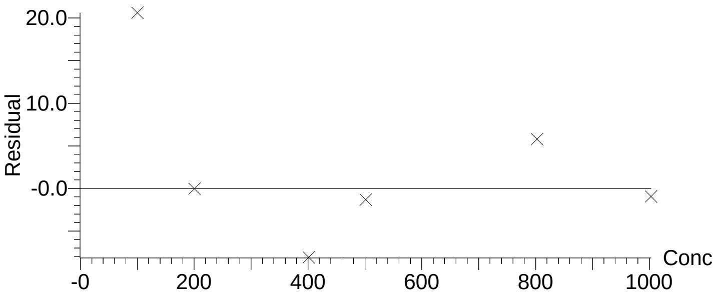

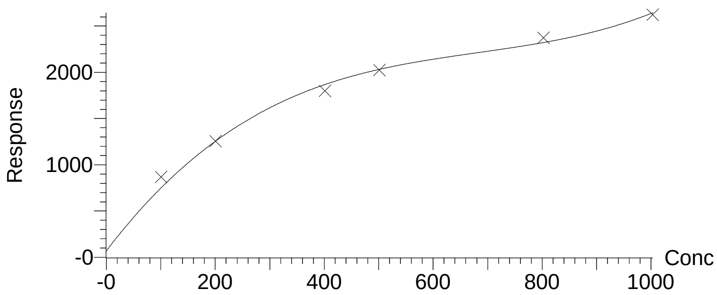

Compound name: ningmensuan
Correlation coefficient: r = 0.998196, r^2 = 0.996396
Calibration curve: 6.86151 * x + 7.63362
Response type: External Std, Area
Curve type: Linear, Origin: Include, Weighting: Null, Axis trans: None

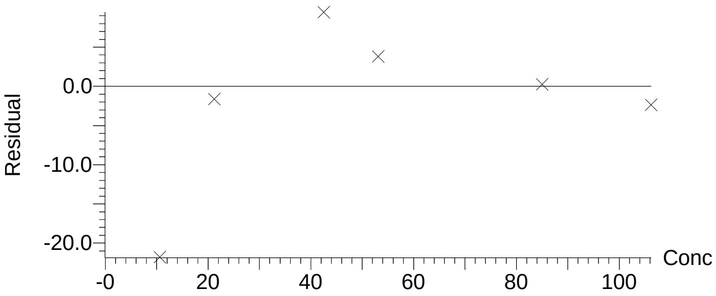

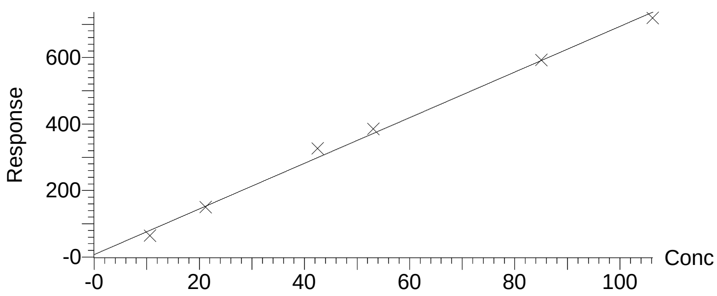

Compound name: pinguosuan
Coefficient of Determination: R^2 = 0.984709
Calibration curve: 9.85928e-006 * x^3 + -0.00971593 * x^2 + 3.27297 * x + 20.8616
Response type: External Std, Area
Curve type: 3rd Order, Origin: Include, Weighting: Null, Axis trans: None

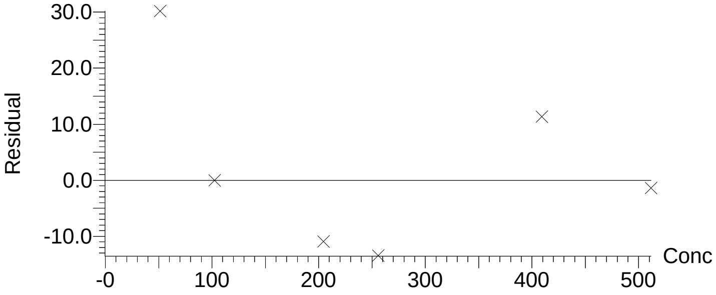

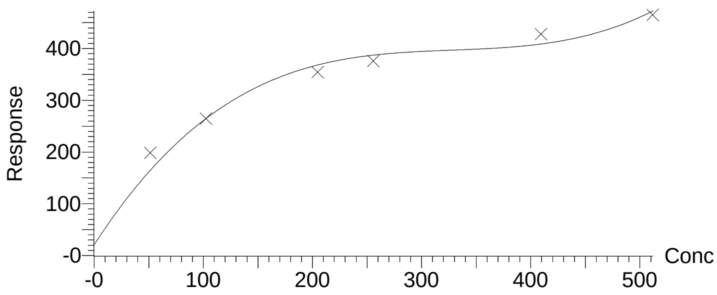

Compound name: huposaun
Coefficient of Determination: R^2 = 0.999608
Calibration curve: 3.18016e-005 * x^3 + -0.0146291 * x^2 + 2.726 * x + 0.728245
Response type: External Std, Area
Curve type: 3rd Order, Origin: Include, Weighting: Null, Axis trans: None

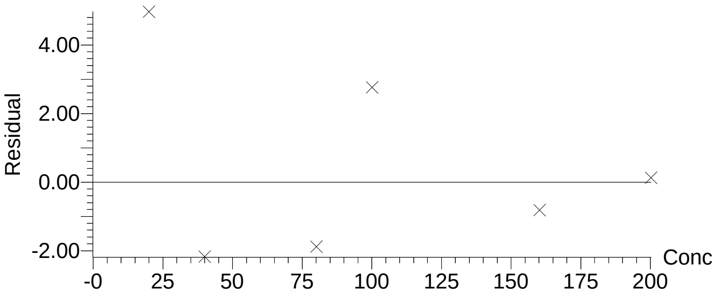

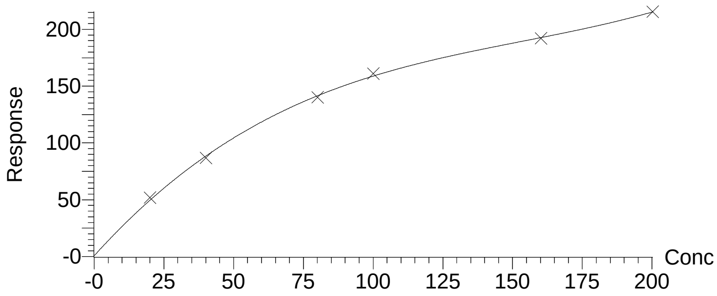

project_wangzhonghua_BeiMu
Dataset: Untitled
Last Altered: Saturday, October 12, 2024 07:48:57 China Standard Time
Printed: Saturday, October 12, 2024 07:49:23 China Standard Time

Method: F:\data\Wu_yueyan.PRO\MethDB\20241011_ organic acid .mdb 11 Oct 2024 15:52:39
Calibration: F:\data\zhanghuien.PRO\CurveDB\20241011_organic acid003.cdb 11 Oct 2024 15:39:56

Name: 202401011_youjisuan_sample099, Date: 11-Oct-2024, Time: 20:03:21, ID: , Description:

jiushisuan

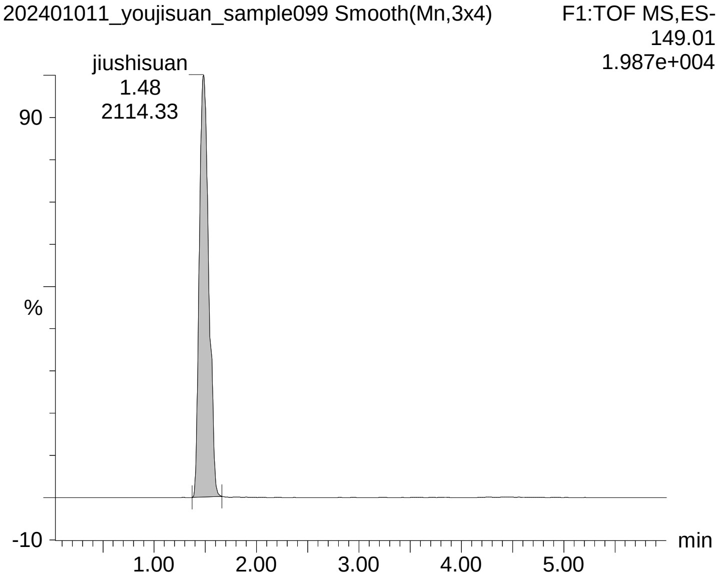

ningmensuan

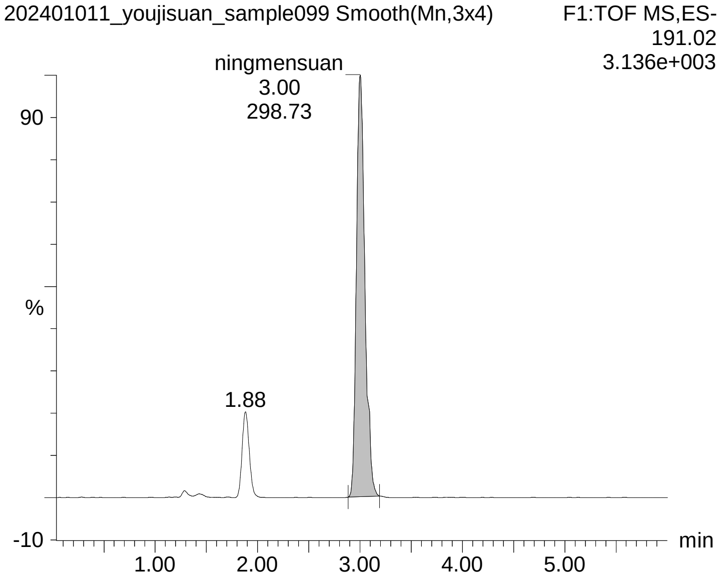

pinguosuan

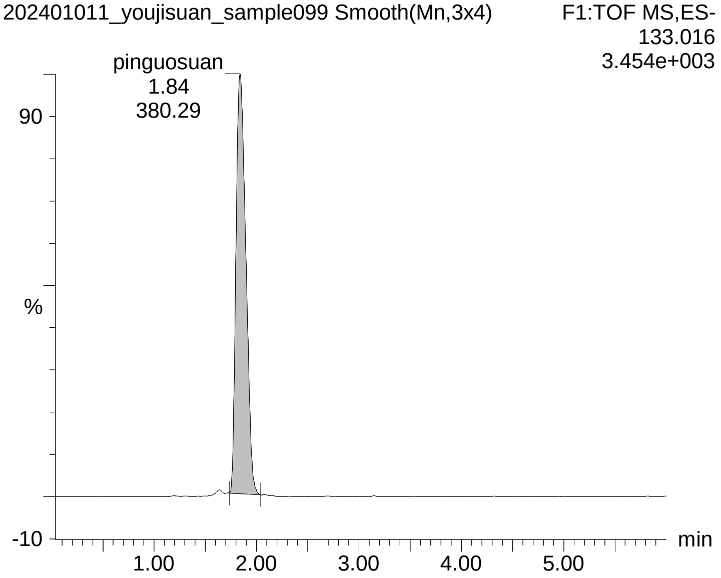

	#	Name	Sample Text	RT	Area	Std. Conc	Conc.
1	1	jiushisuan		1.48	2114.328		572.158823
2	2	ningmensuan		3.00	298.731		42.424710
3	3	pinguosuan		1.84	380.294		231.828006

project_wangzhonghua_BeiMu
Dataset: Untitled
Last Altered: Friday, October 11, 2024 17:06:05 China Standard Time
Printed: Friday, October 11, 2024 17:06:40 China Standard Time

Method: F:\data\Wu_yueyan.PRO\MethDB\20241011_ organic acid .mdb 11 Oct 2024 15:52:39
Calibration: F:\data\zhanghuien.PRO\CurveDB\20241011_organic acid003.cdb 11 Oct 2024 15:39:56

Compound name: jiushisuan
Coefficient of Determination: R^2 = 0.994685
Calibration curve: 4.94514e-006 * x^3 + -0.0101268 * x^2 + 7.74871 * x + 69.7696
Response type: External Std, Area
Curve type: 3rd Order, Origin: Include, Weighting: Null, Axis trans: None

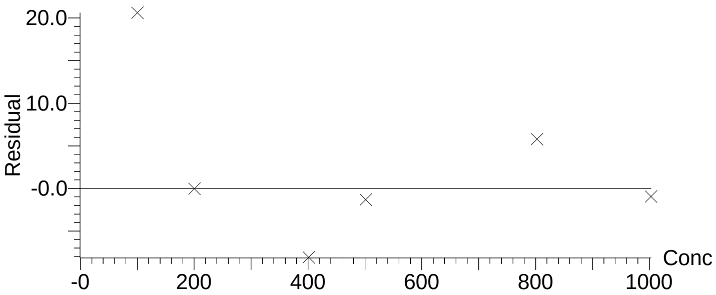

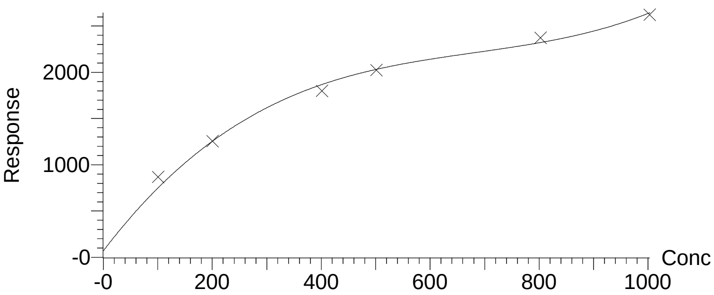

Compound name: ningmensuan
Correlation coefficient: r = 0.998196, r^2 = 0.996396
Calibration curve: 6.86151 * x + 7.63362
Response type: External Std, Area
Curve type: Linear, Origin: Include, Weighting: Null, Axis trans: None

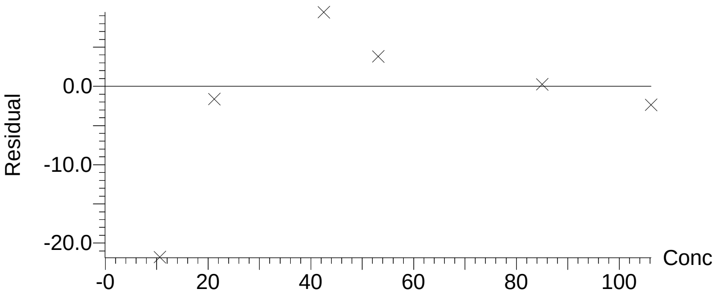

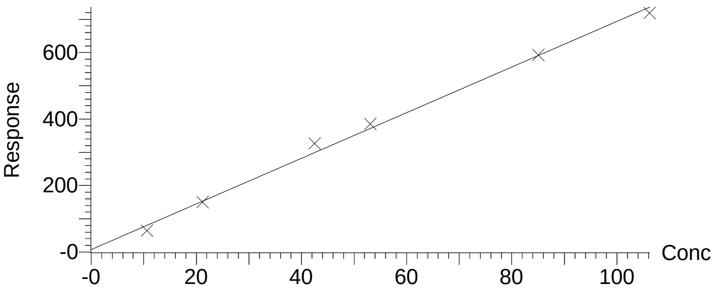

Compound name: pinguosuan
Coefficient of Determination: R^2 = 0.984709
Calibration curve: 9.85928e-006 * x^3 + -0.00971593 * x^2 + 3.27297 * x + 20.8616
Response type: External Std, Area
Curve type: 3rd Order, Origin: Include, Weighting: Null, Axis trans: None

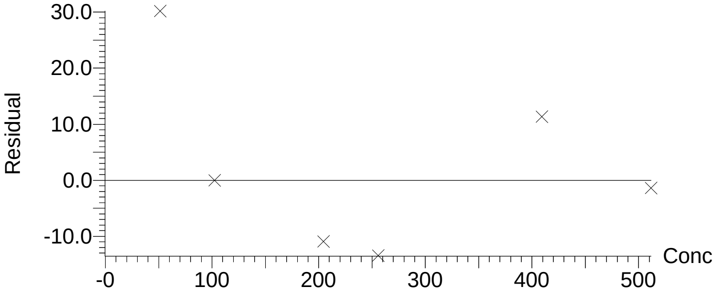

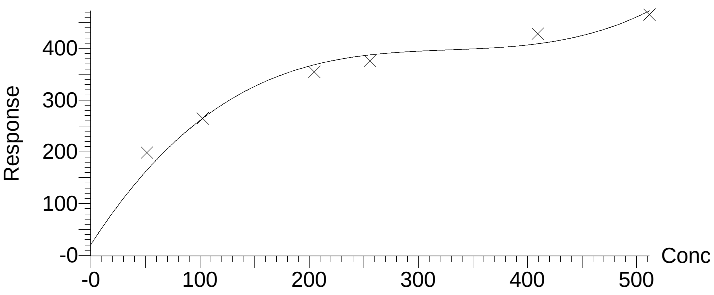

Compound name: huposaun
Coefficient of Determination: R^2 = 0.999608
Calibration curve: 3.18016e-005 * x^3 + -0.0146291 * x^2 + 2.726 * x + 0.728245
Response type: External Std, Area
Curve type: 3rd Order, Origin: Include, Weighting: Null, Axis trans: None

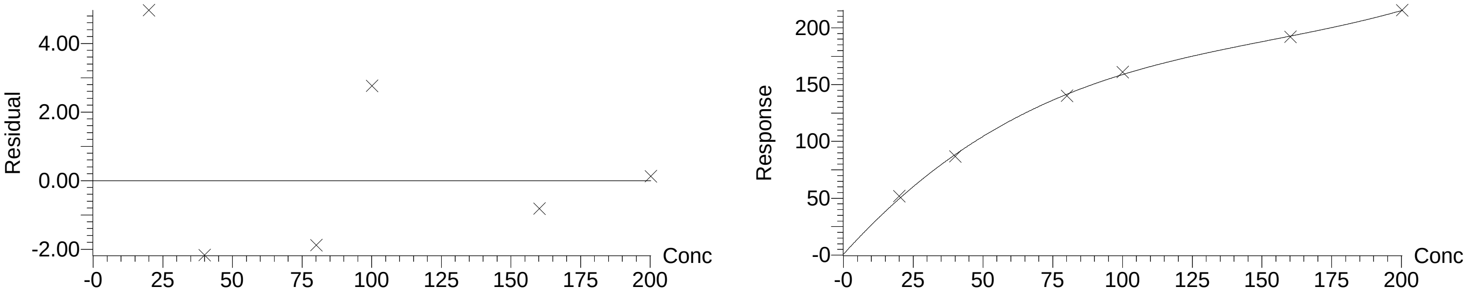

project_wangzhonghua_BeiMu
Dataset: Untitled
Last Altered: Friday, October 11, 2024 17:06:05 China Standard Time
Printed: Friday, October 11, 2024 17:06:40 China Standard Time

Method: F:\data\Wu_yueyan.PRO\MethDB\20241011_ organic acid .mdb 11 Oct 2024 15:52:39
Calibration: F:\data\zhanghuien.PRO\CurveDB\20241011_organic acid003.cdb 11 Oct 2024 15:39:56

Name: 202401011_youjisuan_sample100, Date: 11-Oct-2024, Time: 10:47:36, ID: , Description:

jiushisuan

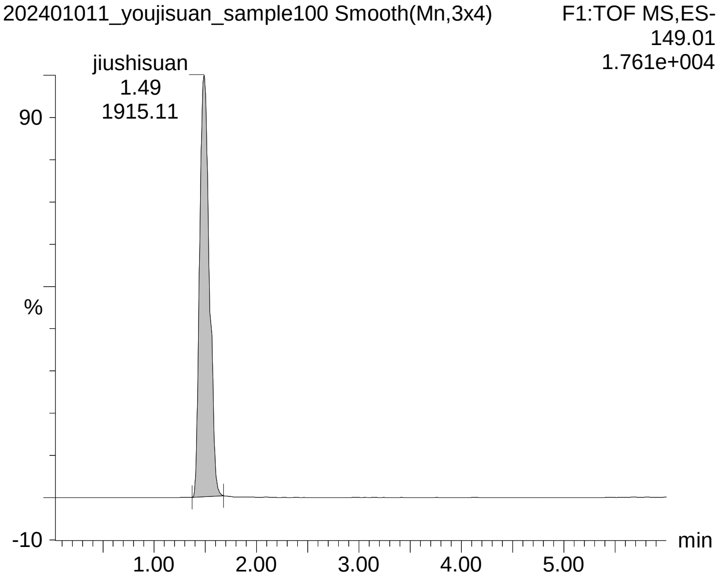

ningmensuan

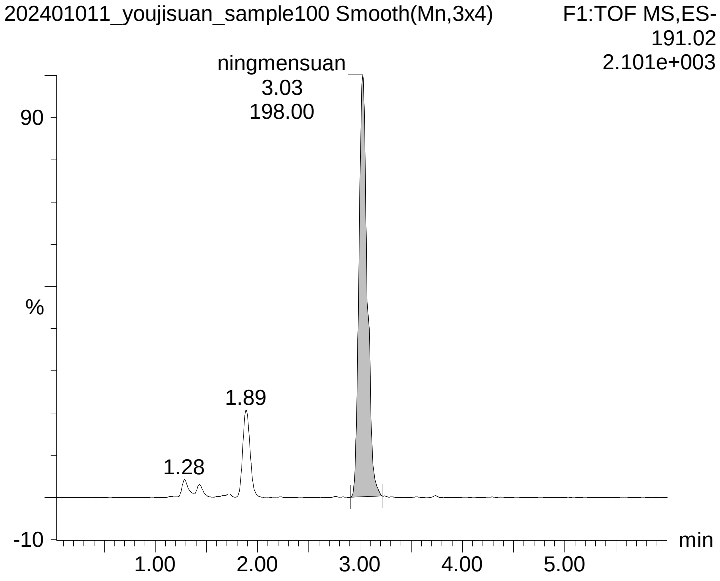

pinguosuan

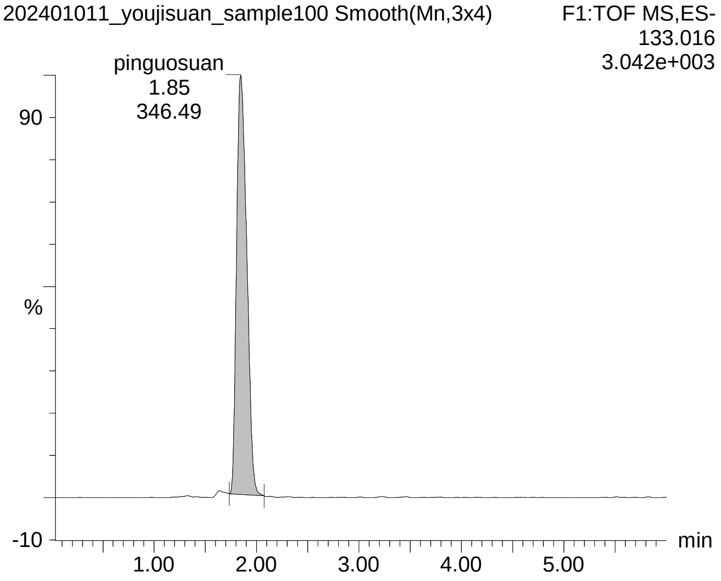

	#	Name	Sample Text	RT	Area	Std. Conc	Conc.
1	1	jiushisuan		1.49	1915.108		425.926413
2	2	ningmensuan		3.03	198.004		27.744695
3	3	pinguosuan		1.85	346.492		171.933953

Name: 202401011_youjisuan_sample101, Date: 11-Oct-2024, Time: 10:54:35, ID: , Description:

jiushisuan

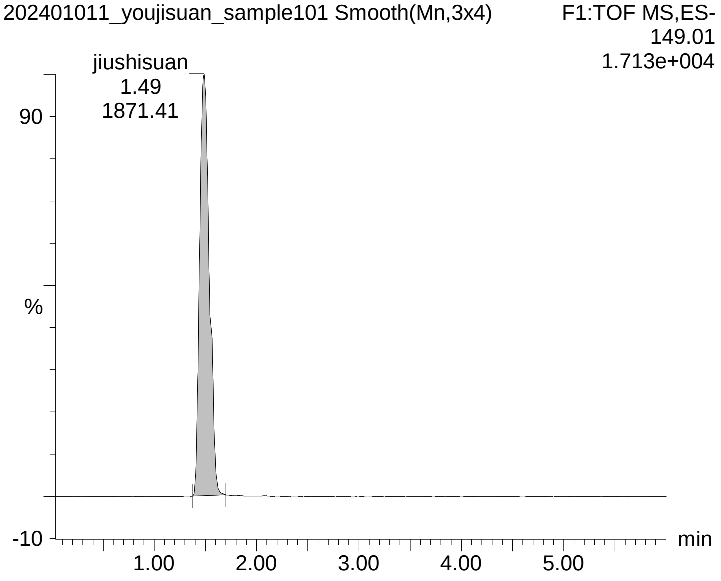

ningmensuan

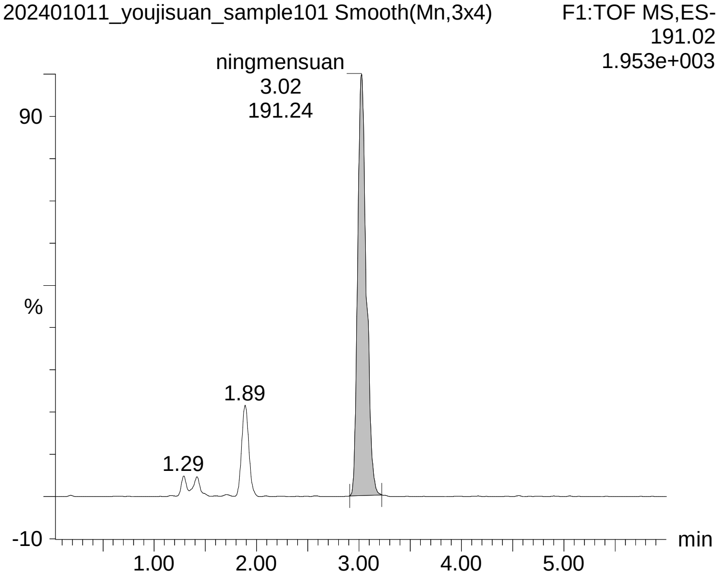

pinguosuan

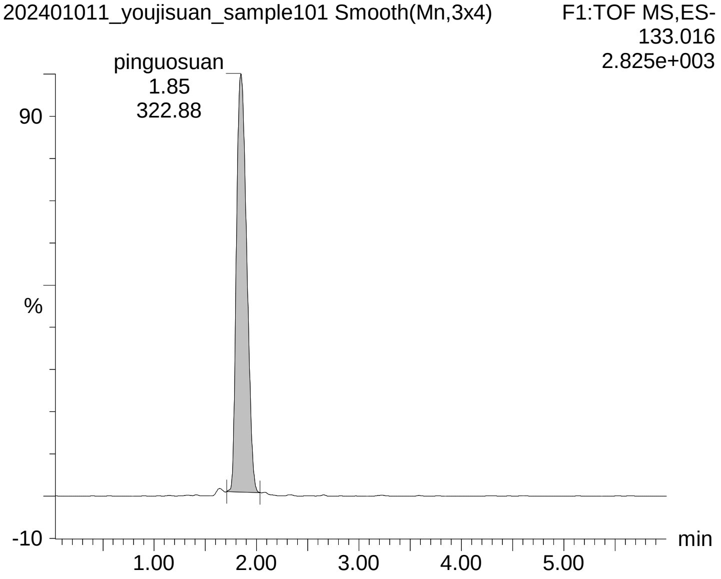

	#	Name	Sample Text	RT	Area	Std. Conc	Conc.
1	1	jiushisuan		1.49	1871.414		402.971662
2	2	ningmensuan		3.02	191.243		26.759343
3	3	pinguosuan		1.85	322.879		146.549859

Name: 202401011_youjisuan_sample102, Date: 11-Oct-2024, Time: 12:05:47, ID: , Description:

jiushisuan

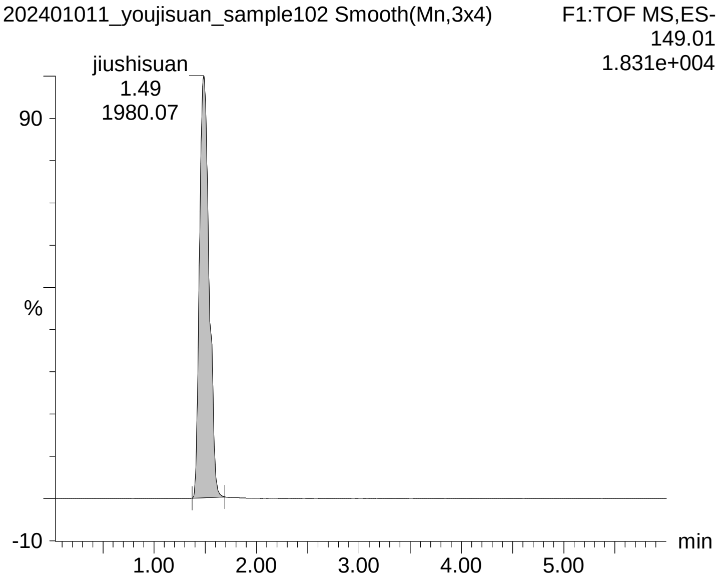

ningmensuan

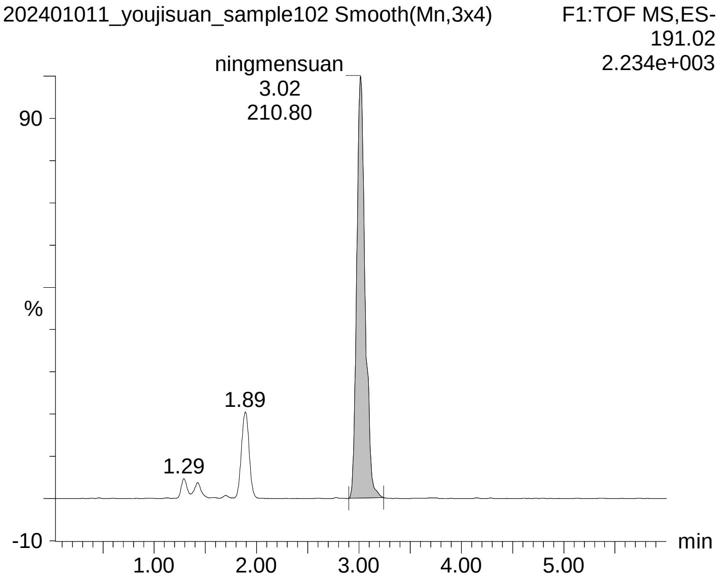

pinguosuan

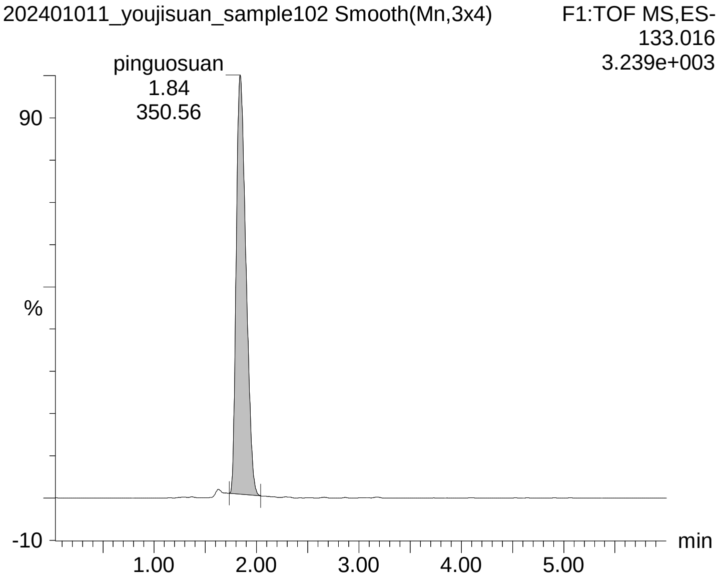

	#	Name	Sample Text	RT	Area	Std. Conc	Conc.
1	1	jiushisuan		1.49	1980.068		464.753344
2	2	ningmensuan		3.02	210.804		29.610175
3	3	pinguosuan		1.84	350.558		177.129847

project_wangzhonghua_BeiMu
Dataset: Untitled
Last Altered: Friday, October 11, 2024 17:06:05 China Standard Time
Printed: Friday, October 11, 2024 17:06:40 China Standard Time

Name: 202401011_youjisuan_sample103, Date: 11-Oct-2024, Time: 12:12:44, ID: , Description:

jiushisuan

202401011_youjisuan_sample103 Smooth(Mn,3x4) F1:TOF MS,ES- 149.01 1.987e+004

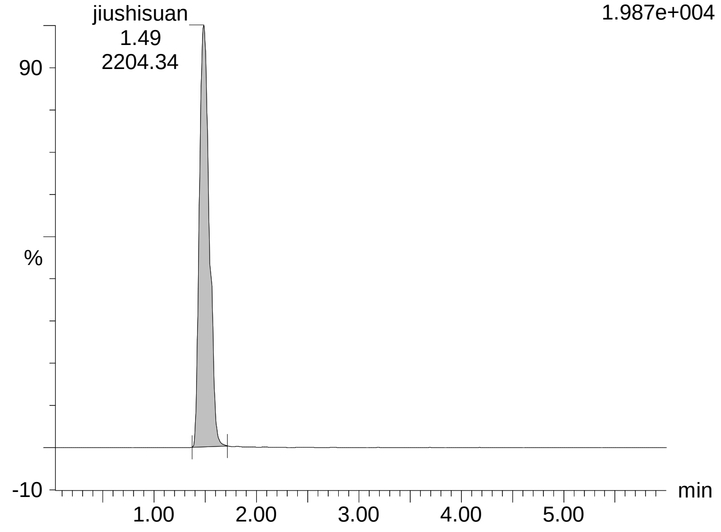

ningmensuan

202401011_youjisuan_sample103 Smooth(Mn,3x4) F1:TOF MS,ES- 191.02 4.202e+003

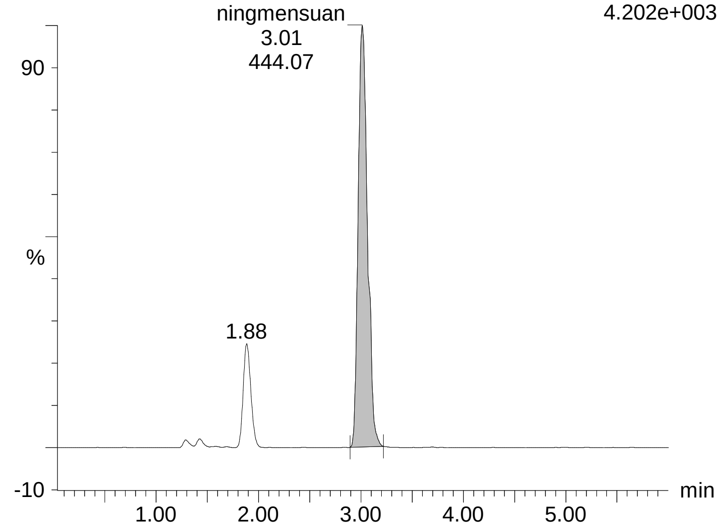

pinguosuan

202401011_youjisuan_sample103 Smooth(Mn,3x4) F1:TOF MS,ES- 133.016 2.942e+003

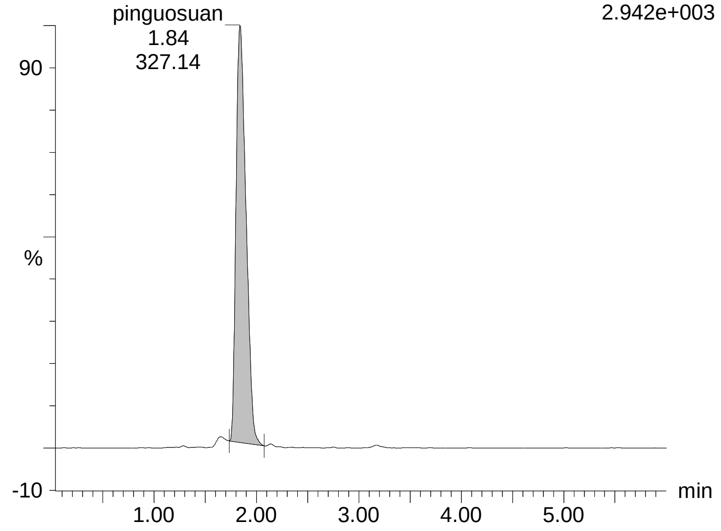

	#	Name	Sample Text	RT	Area	Std. Conc	Conc.
1	1	jiushisuan		1.49	2204.345		671.871886
2	2	ningmensuan		3.01	444.071		63.606650
3	3	pinguosuan		1.84	327.138		150.650679

Name: 202401011_youjisuan_sample104, Date: 11-Oct-2024, Time: 12:19:44, ID: , Description:

jiushisuan

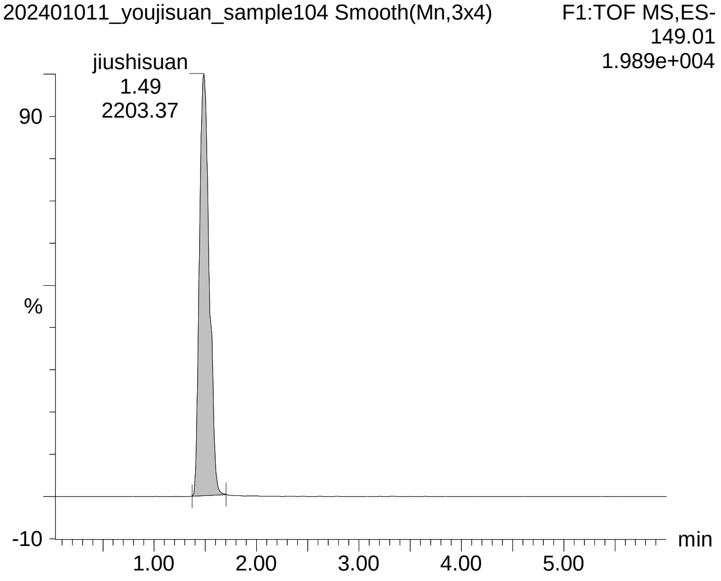

ningmensuan

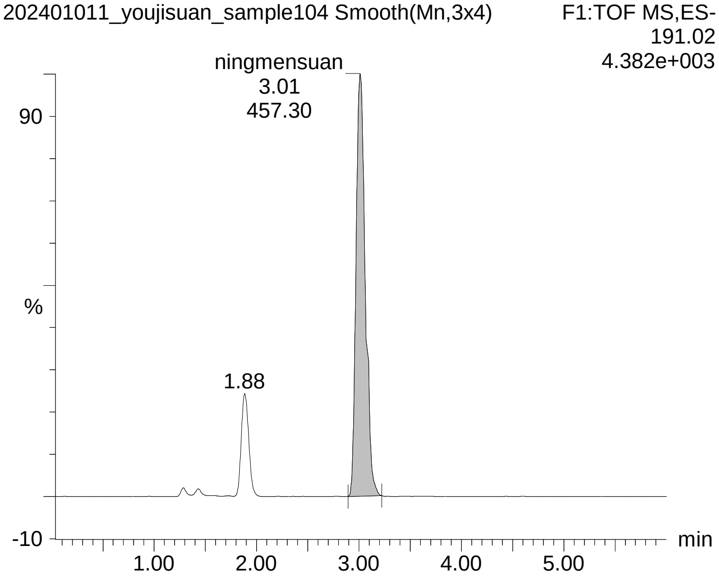

pinguosuan

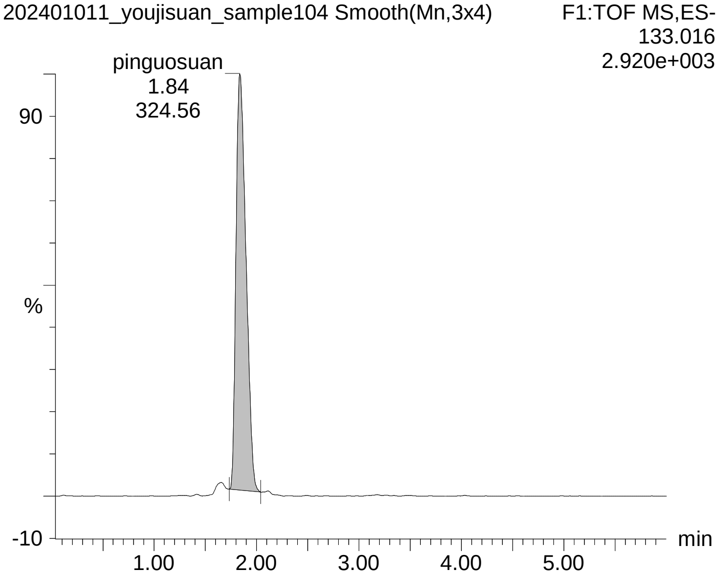

	#	Name	Sample Text	RT	Area	Std. Conc	Conc.
1	1	jiushisuan		1.49	2203.368		670.705871
2	2	ningmensuan		3.01	457.297		65.534215
3	3	pinguosuan		1.84	324.557		148.145044

project_wangzhonghua_BeiMu
Dataset: Untitled
Last Altered: Friday, October 11, 2024 17:06:05 China Standard Time
Printed: Friday, October 11, 2024 17:06:40 China Standard Time

Name: 202401011_youjisuan_sample105, Date: 11-Oct-2024, Time: 12:26:44, ID: , Description:

jiushisuan

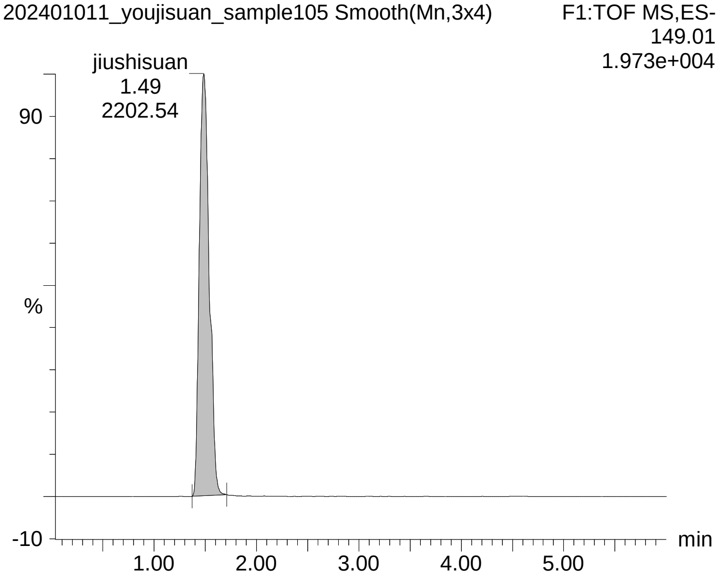

ningmensuan

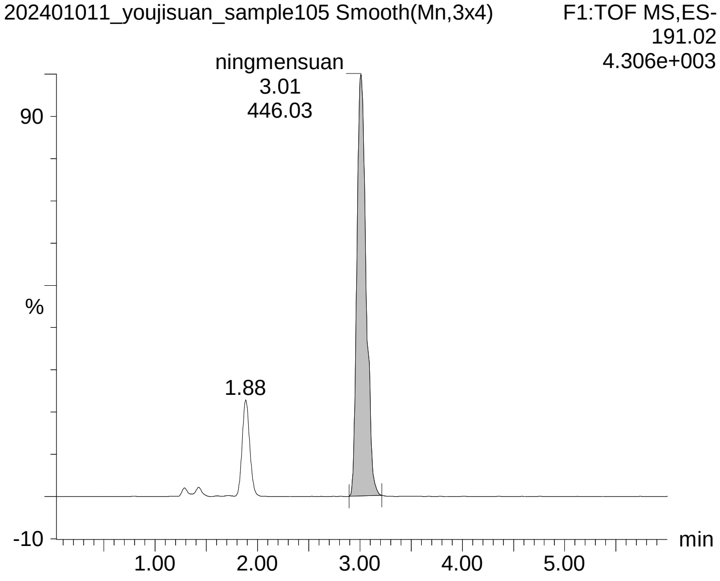

pinguosuan

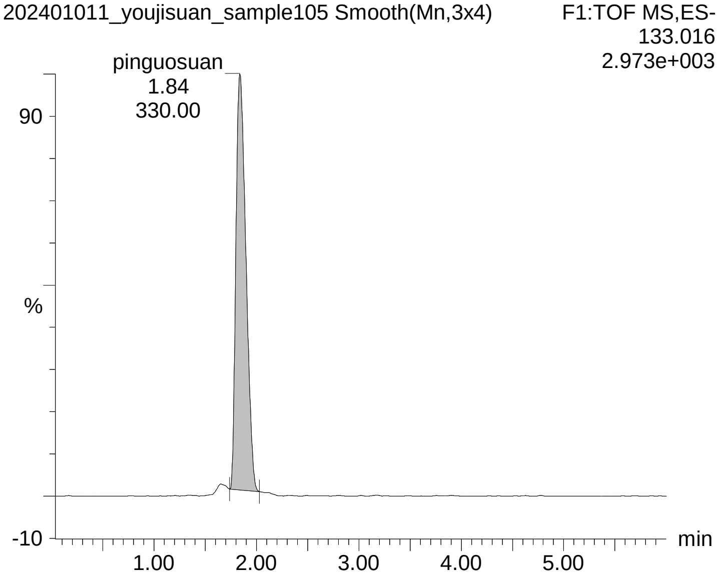

	#	Name	Sample Text	RT	Area	Std. Conc	Conc.
1	1	jiushisuan		1.49	2202.540		669.718128
2	2	ningmensuan		3.01	446.028		63.891864
3	3	pinguosuan		1.84	330.003		153.510315

project_wangzhonghua_BeiMu
Dataset: Untitled
Last Altered: Friday, October 11, 2024 17:06:05 China Standard Time
Printed: Friday, October 11, 2024 17:06:40 China Standard Time

Name: 202401011_youjisuan_sample106, Date: 11-Oct-2024, Time: 12:33:38, ID: , Description:

jiushisuan

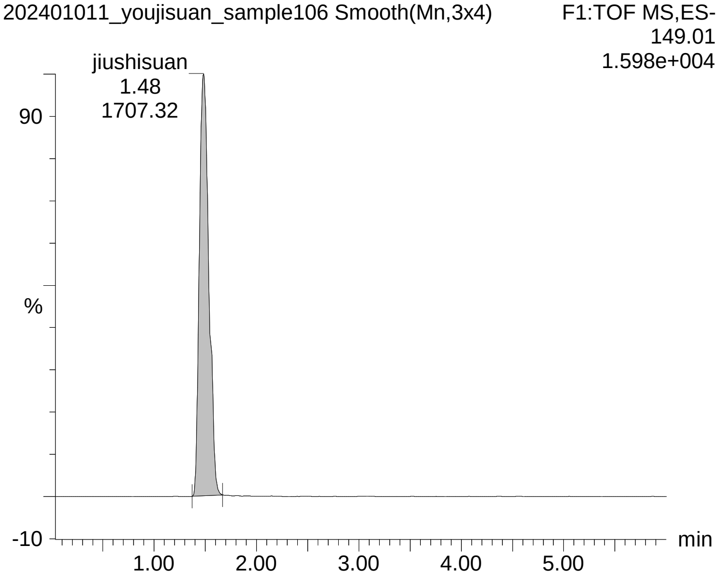

ningmensuan

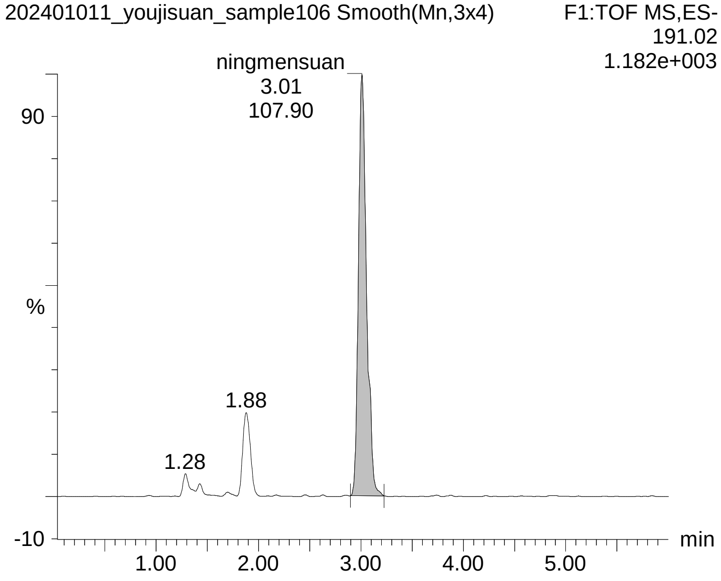

pinguosuan

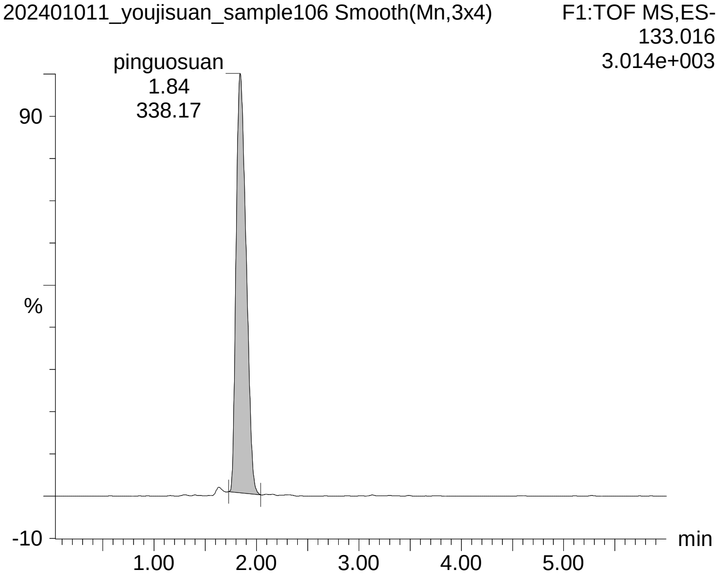

	#	Name	Sample Text	RT	Area	Std. Conc	Conc.
1	1	jiushisuan		1.48	1707.319		332.088851
2	2	ningmensuan		3.01	107.900		14.612884
3	3	pinguosuan		1.84	338.172		162.174042

Name: 202401011_youjisuan_sample107, Date: 11-Oct-2024, Time: 12:40:36, ID: , Description:

jiushisuan

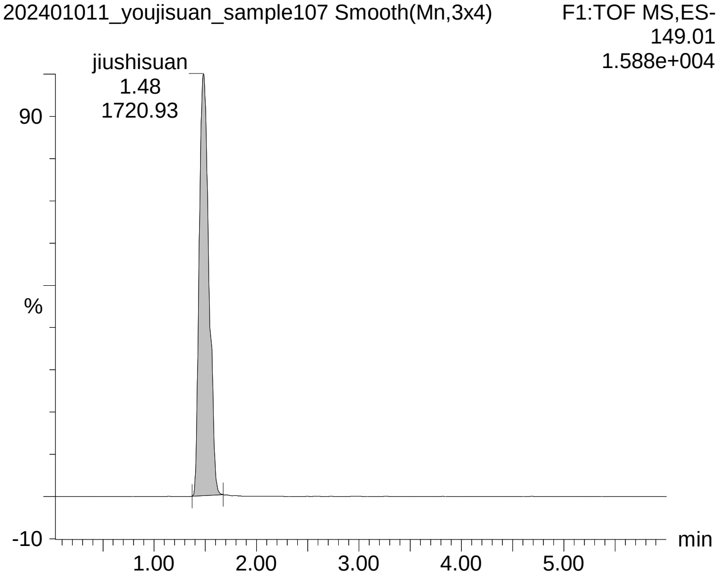

ningmensuan

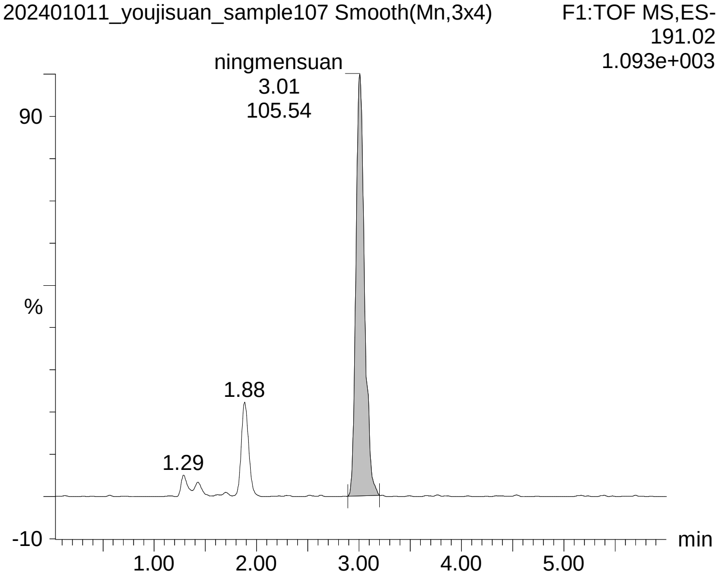

pinguosuan

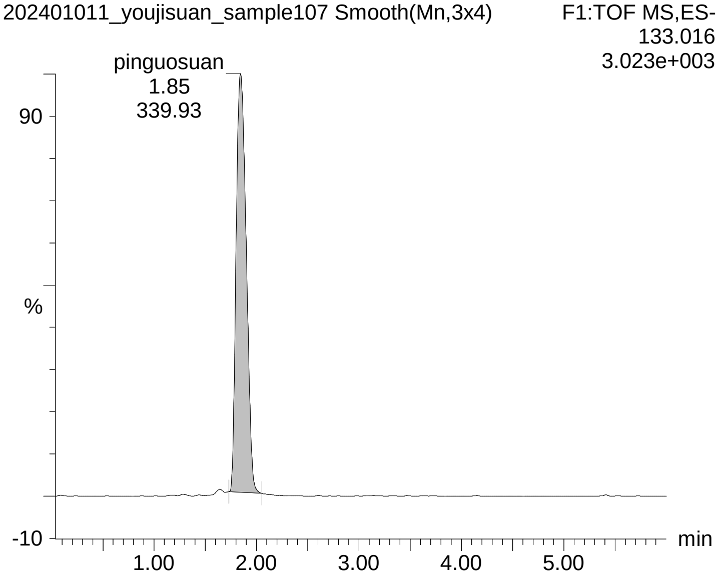

	#	Name	Sample Text	RT	Area	Std. Conc	Conc.
1	1	jiushisuan		1.48	1720.925		337.258255
2	2	ningmensuan		3.01	105.540		14.268936
3	3	pinguosuan		1.85	339.934		164.154181

Name: 202401011_youjisuan_sample108, Date: 11-Oct-2024, Time: 12:47:36, ID: , Description:

jiushisuan

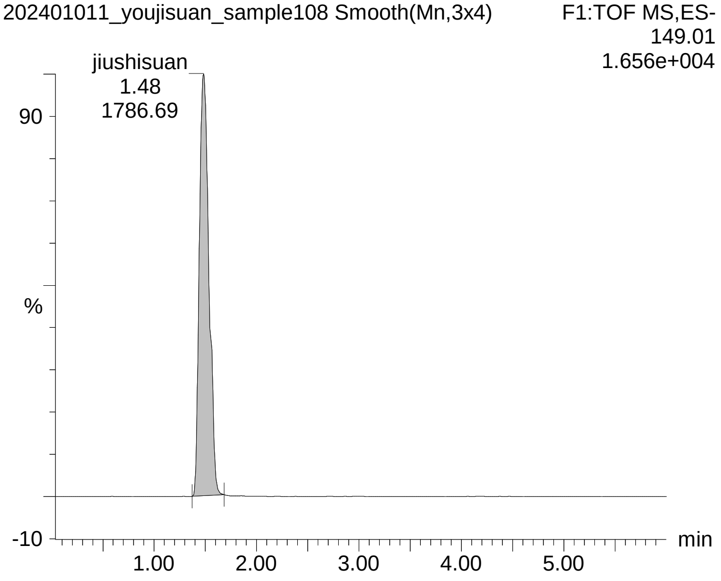

ningmensuan

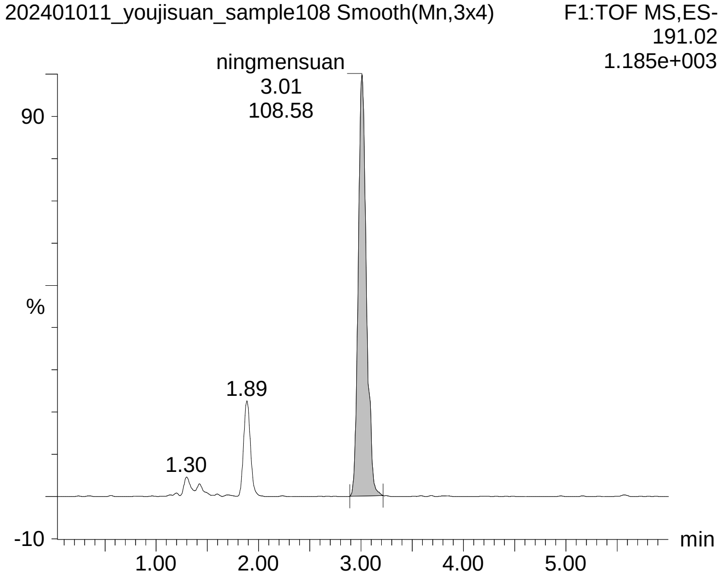

pinguosuan

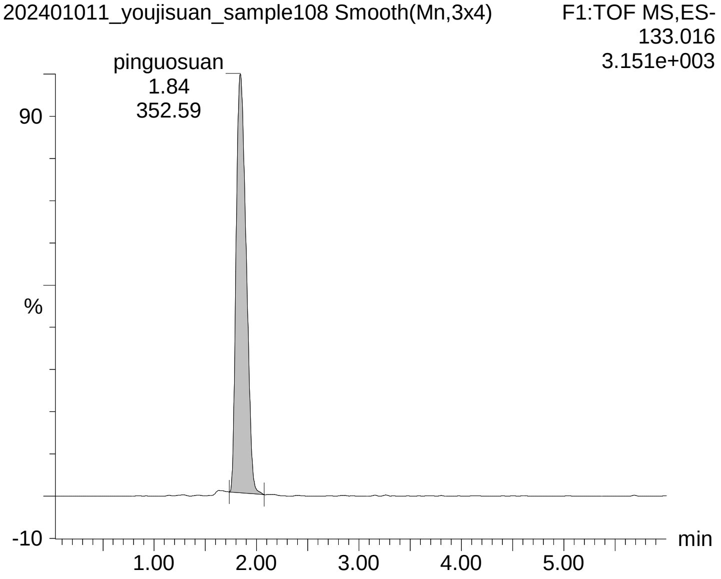

	#	Name	Sample Text	RT	Area	Std. Conc	Conc.
1	1	jiushisuan		1.48	1786.686		363.853670
2	2	ningmensuan		3.01	108.577		14.711550
3	3	pinguosuan		1.84	352.590		179.850399

Name: 202401011_youjisuan_sample109, Date: 11-Oct-2024, Time: 12:54:37, ID: , Description:

jiushisuan

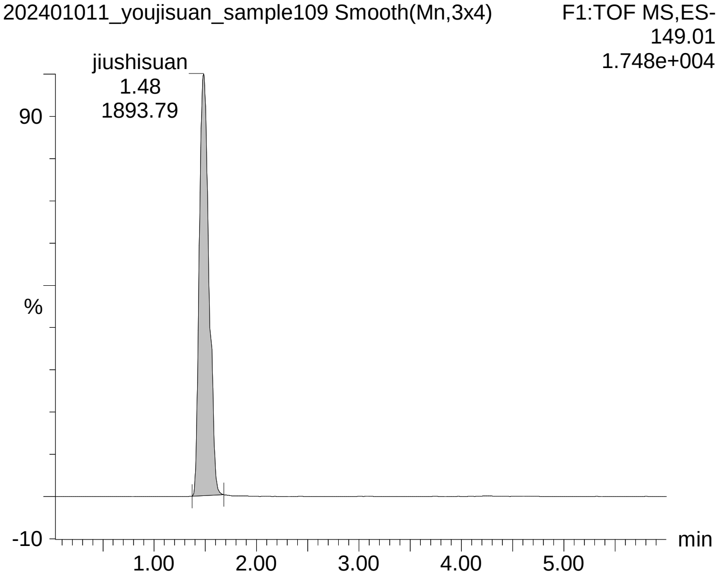

ningmensuan

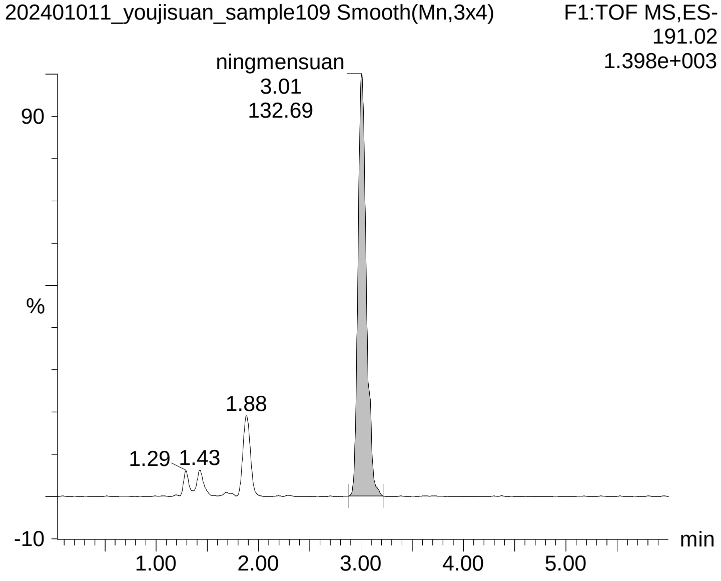

pinguosuan

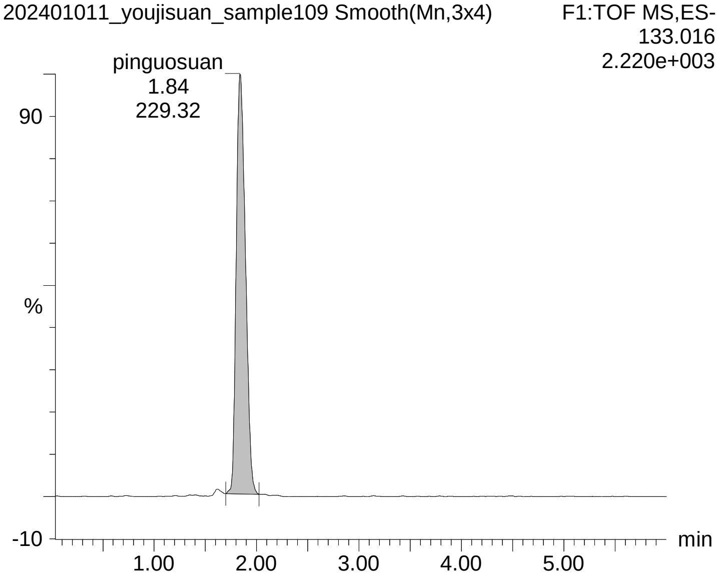

	#	Name	Sample Text	RT	Area	Std. Conc	Conc.
1	1	jiushisuan		1.48	1893.788		414.450580
2	2	ningmensuan		3.01	132.687		18.225356
3	3	pinguosuan		1.84	229.315		81.980543

Name: 202401011_youjisuan_sample110, Date: 11-Oct-2024, Time: 13:01:37, ID: , Description:

jiushisuan

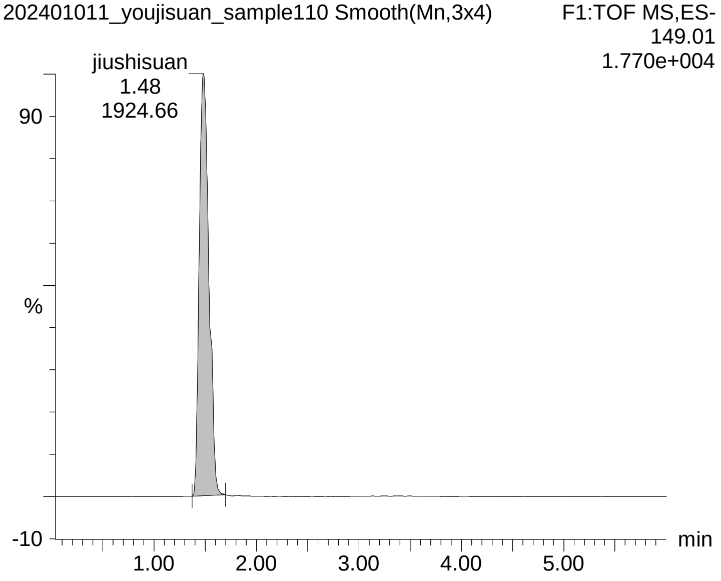

ningmensuan

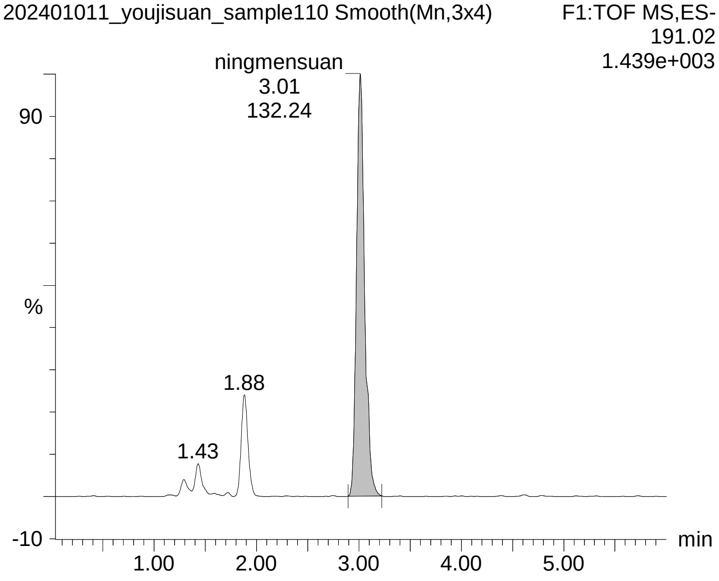

pinguosuan

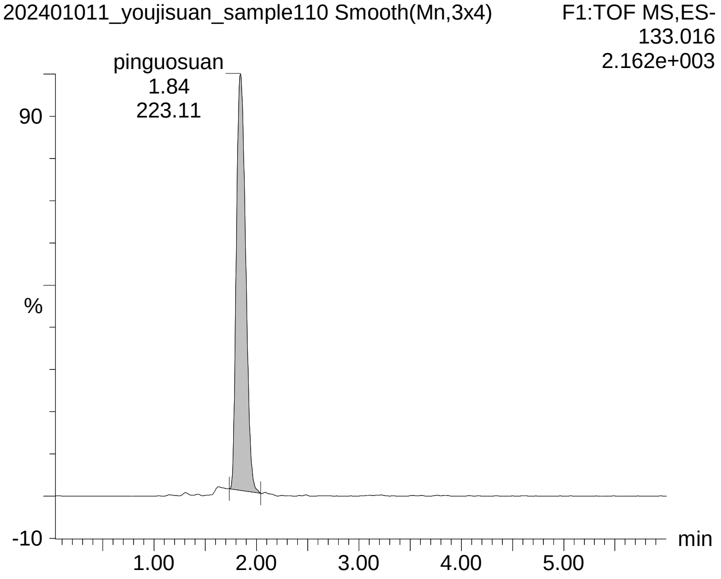

	#	Name	Sample Text	RT	Area	Std. Conc	Conc.
1	1	jiushisuan		1.48	1924.661		431.253404
2	2	ningmensuan		3.01	132.236		18.159627
3	3	pinguosuan		1.84	223.108		78.718193

Name: 202401011_youjisuan_sample111, Date: 11-Oct-2024, Time: 13:09:36, ID: , Description:

jiushisuan

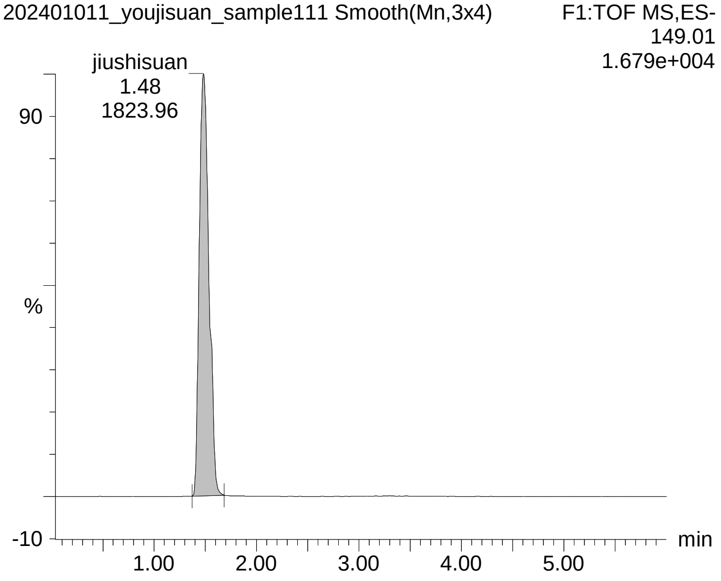

ningmensuan

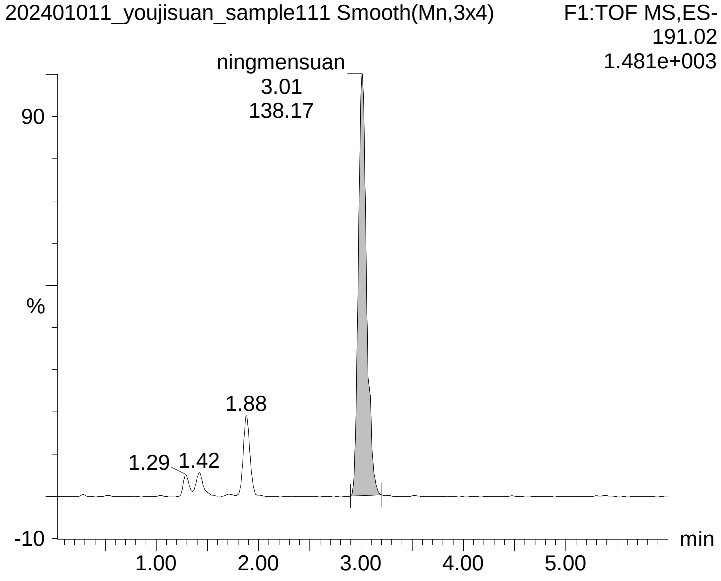

pinguosuan

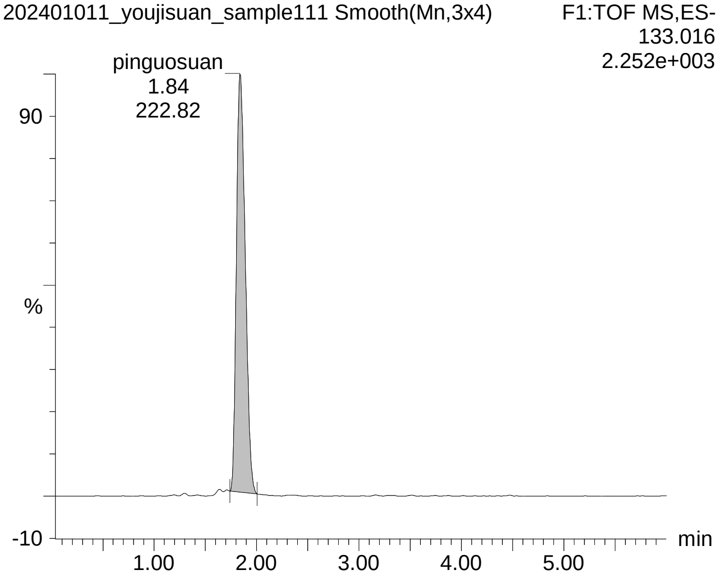

	#	Name	Sample Text	RT	Area	Std. Conc	Conc.
1	1	jiushisuan		1.48	1823.962		380.296857
2	2	ningmensuan		3.01	138.169		19.024306
3	3	pinguosuan		1.84	222.820		78.568794

Name: 202401011_youjisuan_sample112, Date: 11-Oct-2024, Time: 13:16:34, ID: , Description:

jiushisuan

202401011_youjisuan_sample112 Smooth(Mn,3x4) F1:TOF MS,ES- 149.01 1.912e+004

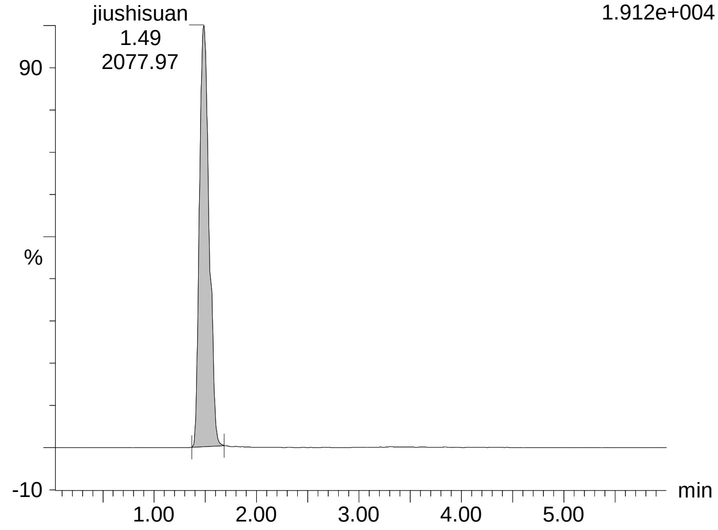

ningmensuan

202401011_youjisuan_sample112 Smooth(Mn,3x4) F1:TOF MS,ES- 191.02 2.179e+003

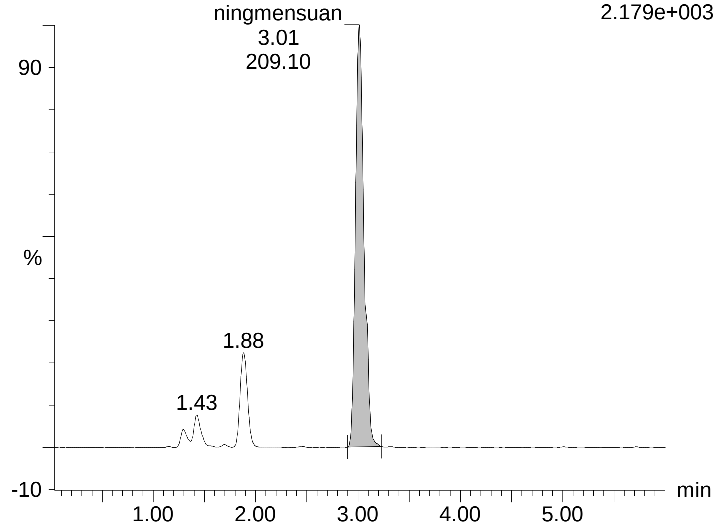

pinguosuan

202401011_youjisuan_sample112 Smooth(Mn,3x4) F1:TOF MS,ES- 133.016 3.237e+003

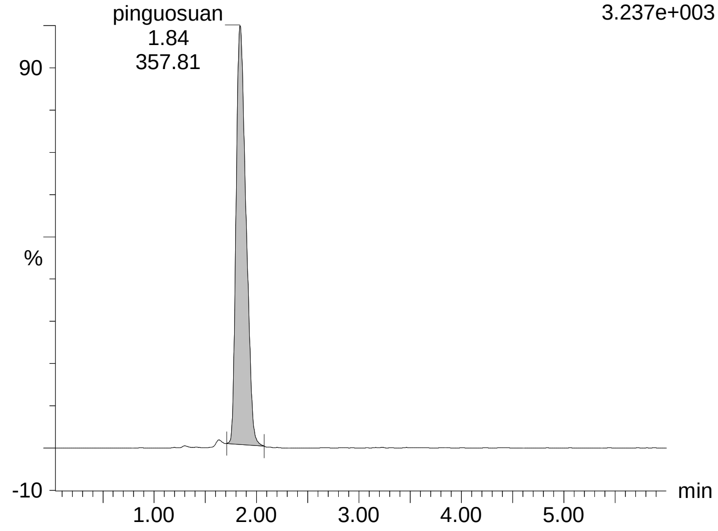

	#	Name	Sample Text	RT	Area	Std. Conc	Conc.
1	1	jiushisuan		1.49	2077.974		538.431408
2	2	ningmensuan		3.01	209.102		29.362125
3	3	pinguosuan		1.84	357.811		187.279719

Name: 202401011_youjisuan_sample113, Date: 11-Oct-2024, Time: 13:23:34, ID: , Description:

jiushisuan

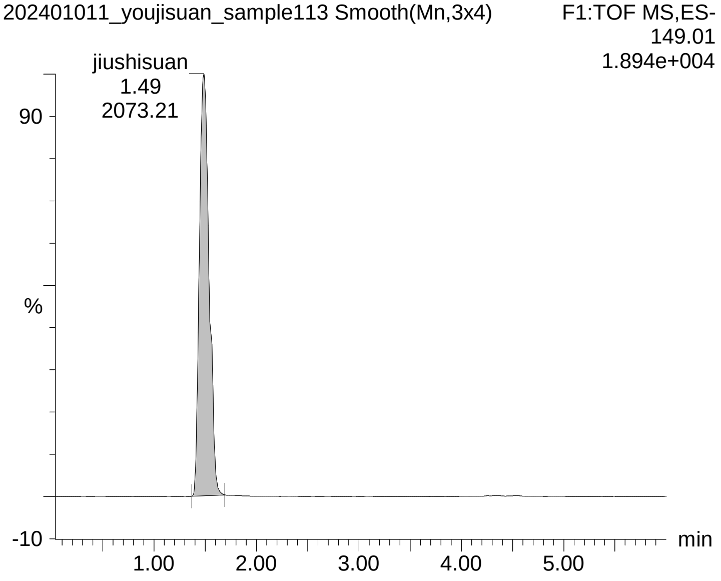

ningmensuan

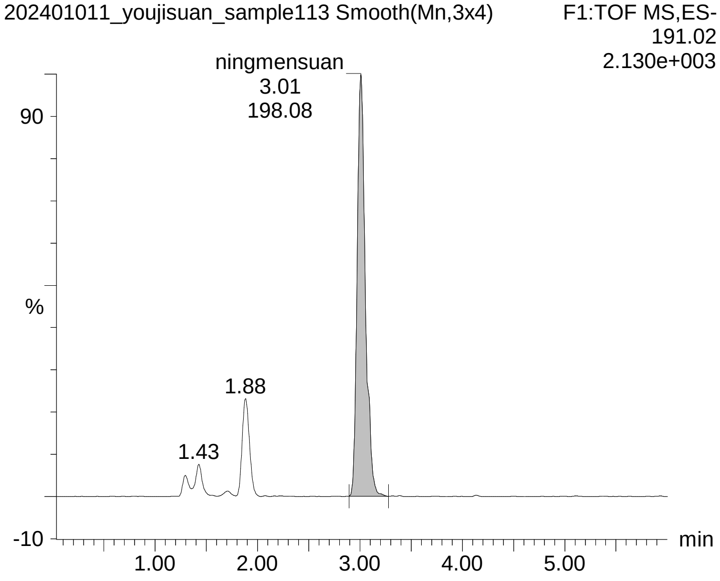

pinguosuan

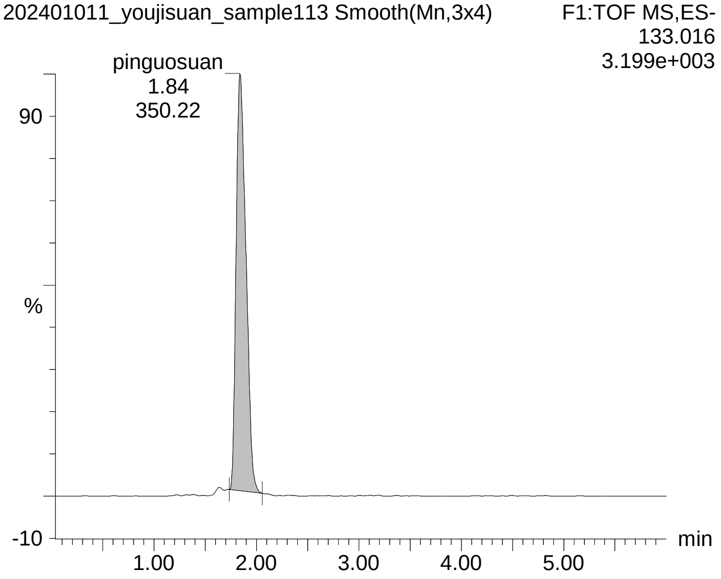

	#	Name	Sample Text	RT	Area	Std. Conc	Conc.
1	1	jiushisuan		1.49	2073.208		534.298982
2	2	ningmensuan		3.01	198.082		27.756063
3	3	pinguosuan		1.84	350.220		176.685697

Name: 202401011_youjisuan_sample114, Date: 11-Oct-2024, Time: 13:30:34, ID: , Description:

jiushisuan

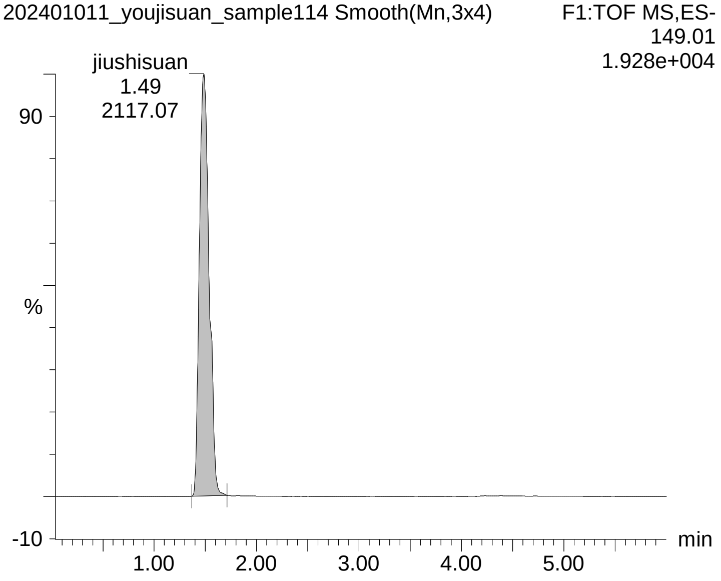

ningmensuan

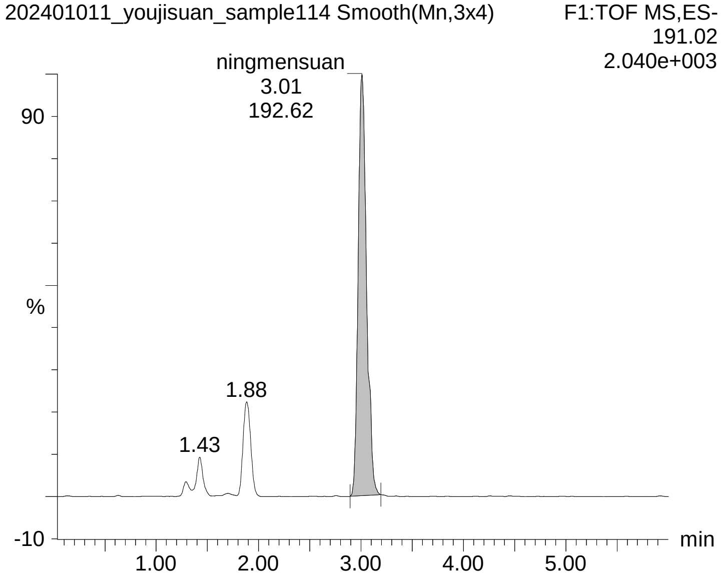

pinguosuan

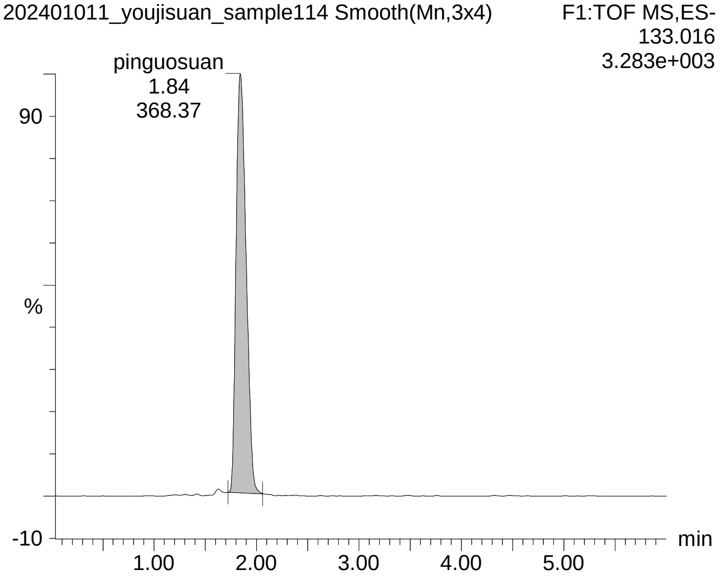

	#	Name	Sample Text	RT	Area	Std. Conc	Conc.
1	1	jiushisuan		1.49	2117.075		574.871706
2	2	ningmensuan		3.01	192.624		26.960611
3	3	pinguosuan		1.84	368.366		204.846314

project_wangzhonghua_BeiMu
Dataset: Untitled
Last Altered: Friday, October 11, 2024 17:06:05 China Standard Time
Printed: Friday, October 11, 2024 17:06:40 China Standard Time

Name: 202401011_youjisuan_sample115, Date: 11-Oct-2024, Time: 13:37:28, ID: , Description:

jiushisuan

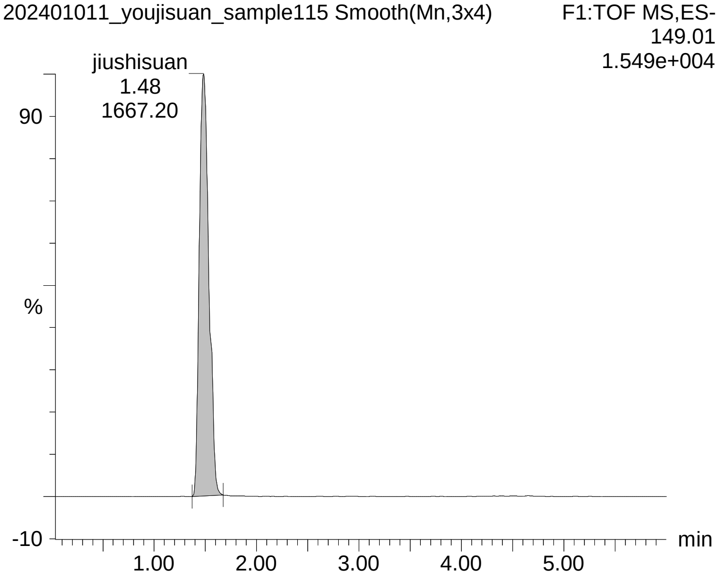

ningmensuan

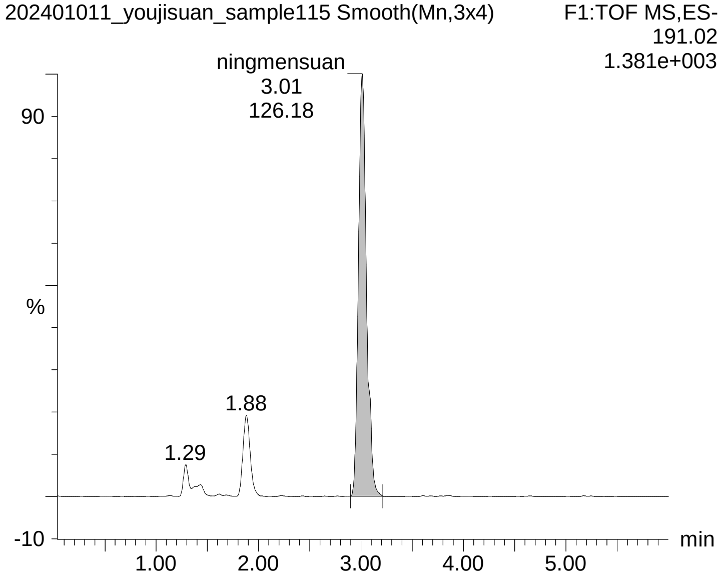

pinguosuan

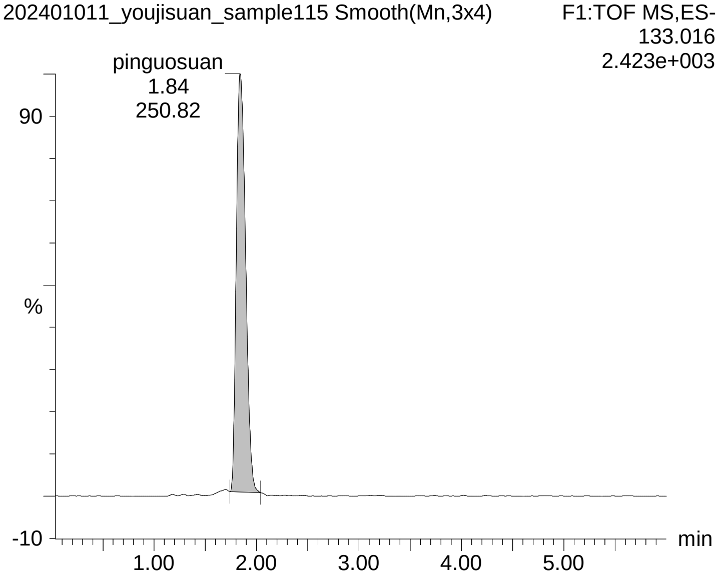

	#	Name	Sample Text	RT	Area	Std. Conc	Conc.
1	1	jiushisuan		1.48	1667.199		317.425587
2	2	ningmensuan		3.01	126.183		17.277459
3	3	pinguosuan		1.84	250.822		93.977723

Name: 202401011_youjisuan_sample116, Date: 11-Oct-2024, Time: 13:44:28, ID: , Description:

jiushisuan

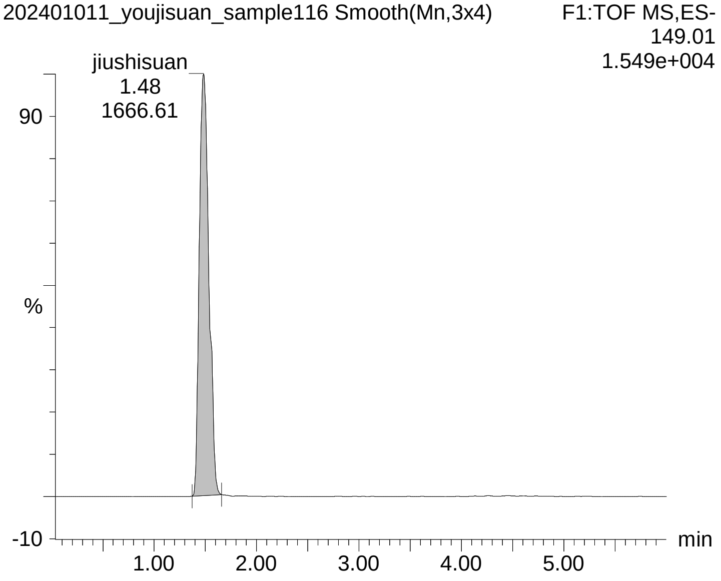

ningmensuan

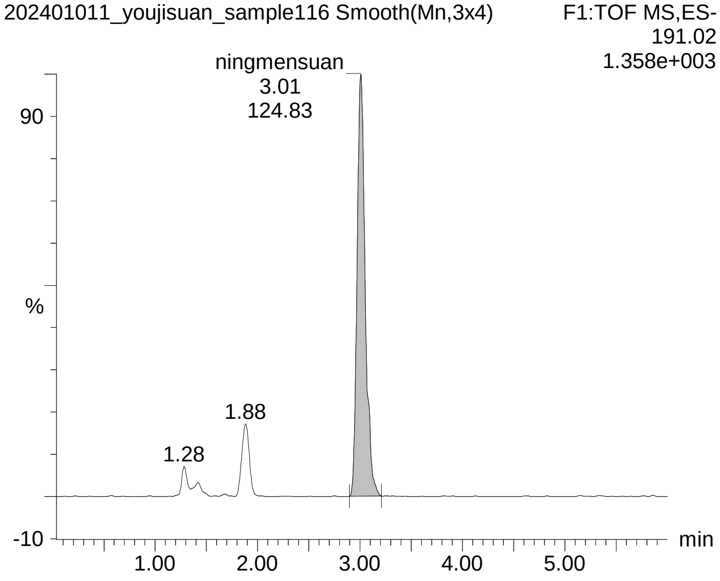

pinguosuan

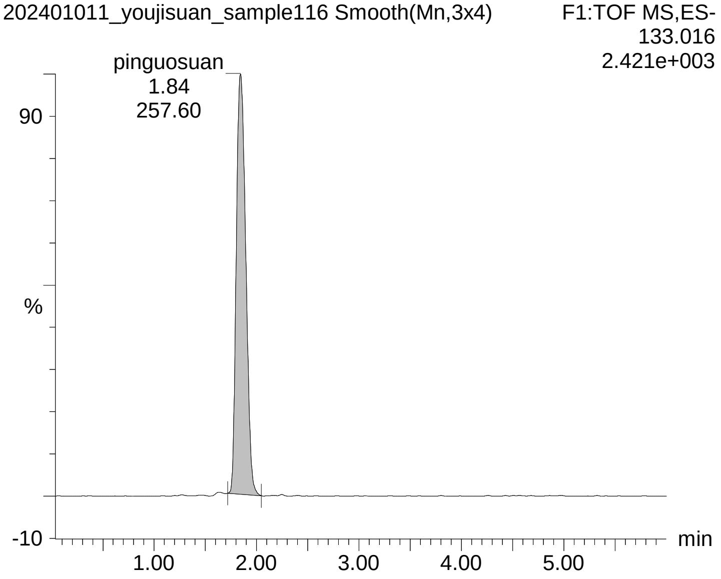

	#	Name	Sample Text	RT	Area	Std. Conc	Conc.
1	1	jiushisuan		1.48	1666.607		317.215330
2	2	ningmensuan		3.01	124.829		17.080126
3	3	pinguosuan		1.84	257.596		98.009308

project_wangzhonghua_BeiMu
Dataset: Untitled
Last Altered: Friday, October 11, 2024 17:06:05 China Standard Time
Printed: Friday, October 11, 2024 17:06:40 China Standard Time

Name: 202401011_youjisuan_sample117, Date: 11-Oct-2024, Time: 13:51:28, ID: , Description:

jiushisuan

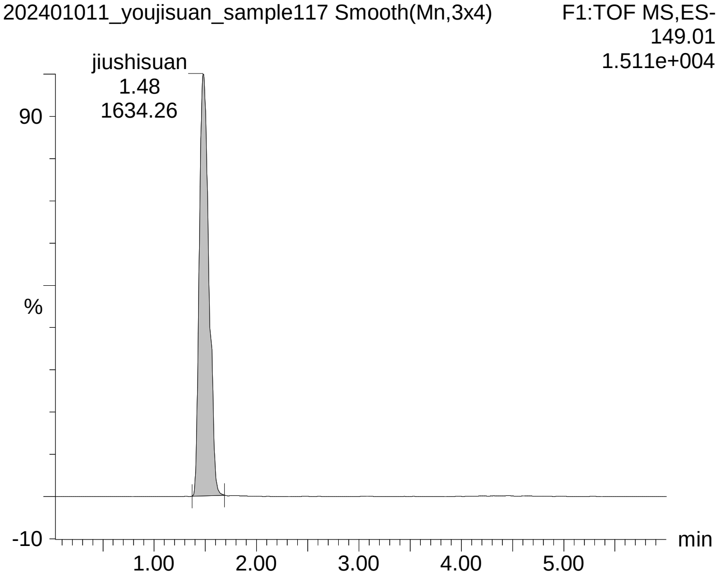

ningmensuan

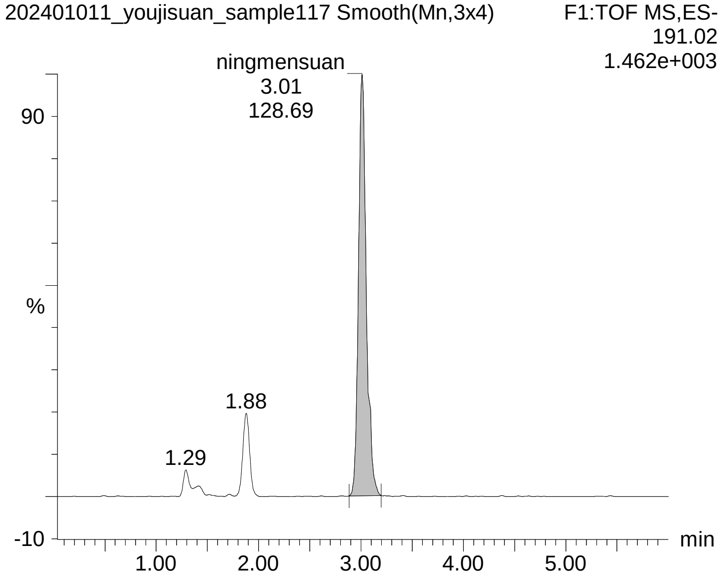

pinguosuan

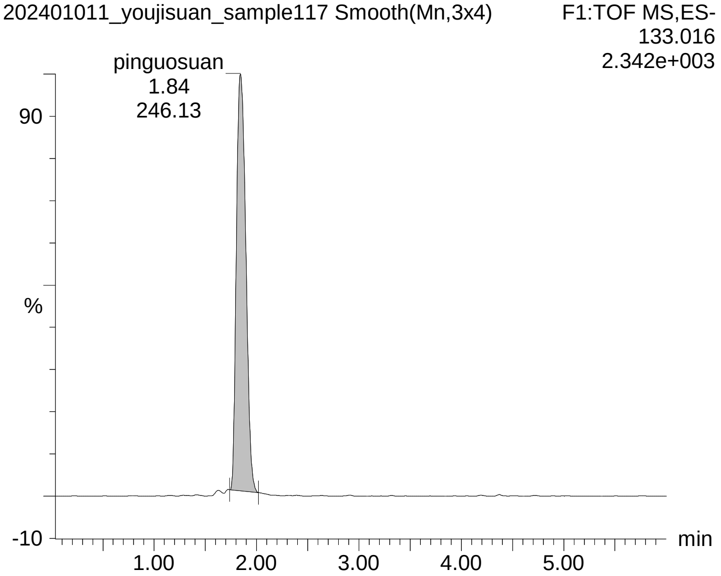

	#	Name	Sample Text	RT	Area	Std. Conc	Conc.
1	1	jiushisuan		1.48	1634.257		305.976045
2	2	ningmensuan		3.01	128.691		17.642977
3	3	pinguosuan		1.84	246.135		91.263665

Name: 202401011_youjisuan_sample118, Date: 11-Oct-2024, Time: 13:58:28, ID: , Description:

jiushisuan

202401011_youjisuan_sample118 Smooth(Mn,3x4) F1:TOF MS,ES- 149.01 2.017e+004

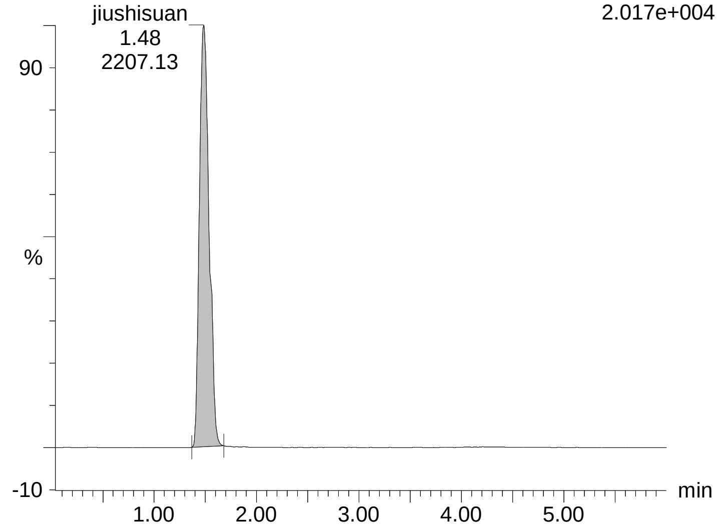

ningmensuan

202401011_youjisuan_sample118 Smooth(Mn,3x4) F1:TOF MS,ES- 191.02 1.841e+003

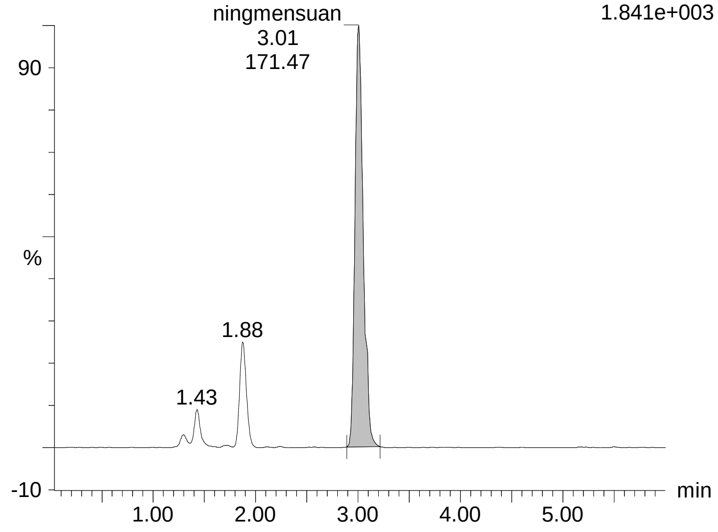

pinguosuan

202401011_youjisuan_sample118 Smooth(Mn,3x4) F1:TOF MS,ES- 133.016 2.038e+003

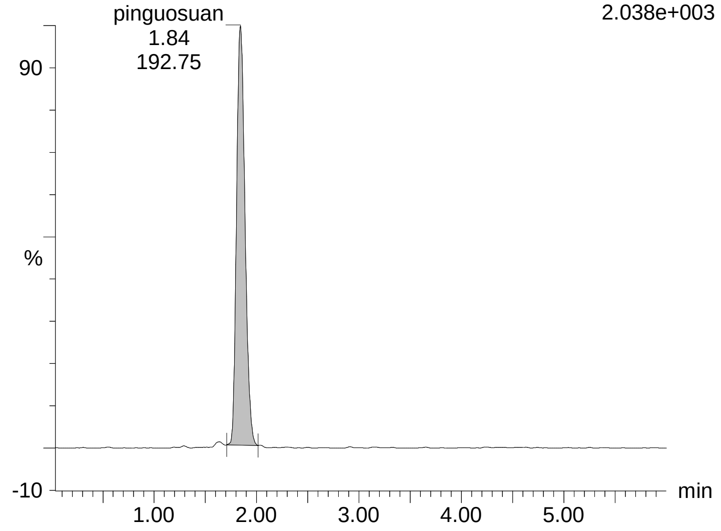

	#	Name	Sample Text	RT	Area	Std. Conc	Conc.
1	1	jiushisuan		1.48	2207.131		675.199533
2	2	ningmensuan		3.01	171.474		23.878197
3	3	pinguosuan		1.84	192.745		63.825746

Name: 202401011_youjisuan_sample119, Date: 11-Oct-2024, Time: 14:05:28, ID: , Description:

jiushisuan

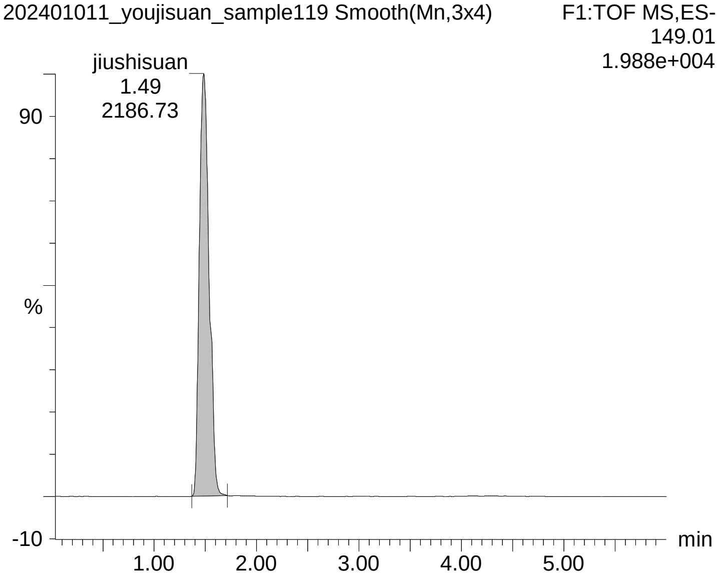

ningmensuan

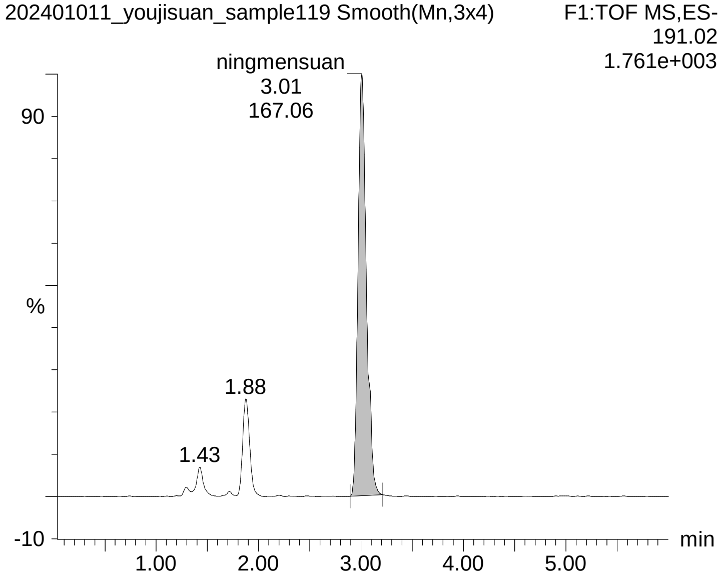

pinguosuan

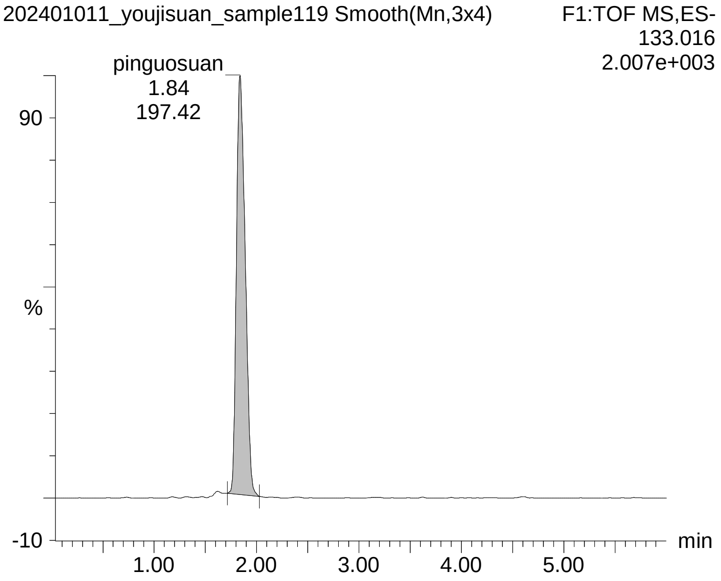

	#	Name	Sample Text	RT	Area	Std. Conc	Conc.
1	1	jiushisuan		1.49	2186.733		650.984531
2	2	ningmensuan		3.01	167.056		23.234315
3	3	pinguosuan		1.84	197.418		66.013343

Name: 202401011_youjisuan_sample120, Date: 11-Oct-2024, Time: 14:12:29, ID: , Description:

jiushisuan

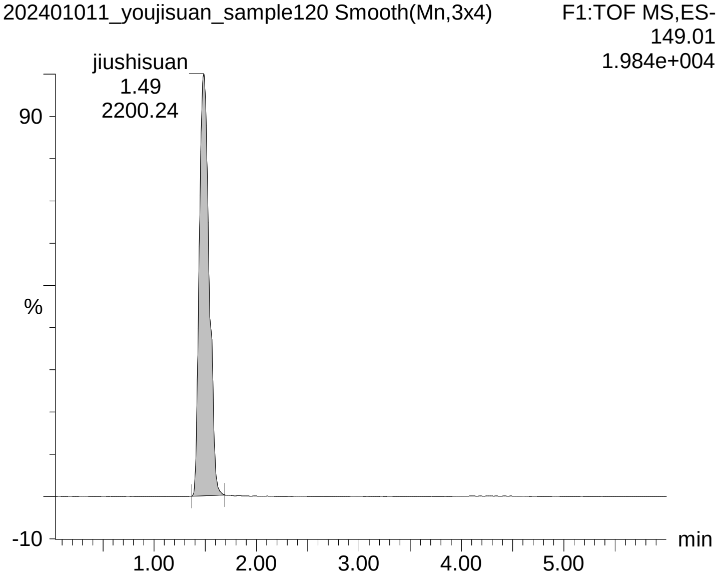

ningmensuan

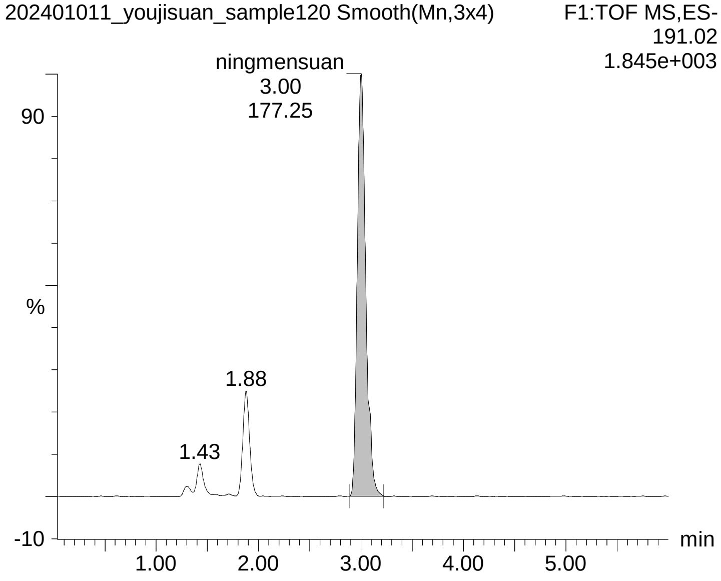

pinguosuan

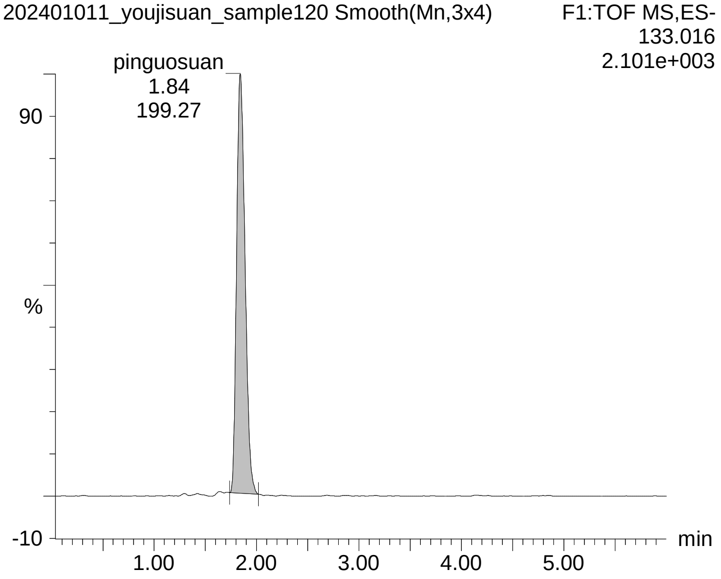

	#	Name	Sample Text	RT	Area	Std. Conc	Conc.
1	1	jiushisuan		1.49	2200.239		666.975646
2	2	ningmensuan		3.00	177.252		24.720286
3	3	pinguosuan		1.84	199.274		66.892010

project_wangzhonghua_BeiMu
Dataset: Untitled
Last Altered: Friday, October 11, 2024 17:06:05 China Standard Time
Printed: Friday, October 11, 2024 17:06:40 China Standard Time

Name: 202401011_youjisuan_sample121, Date: 11-Oct-2024, Time: 14:19:30, ID: , Description:

jiushisuan

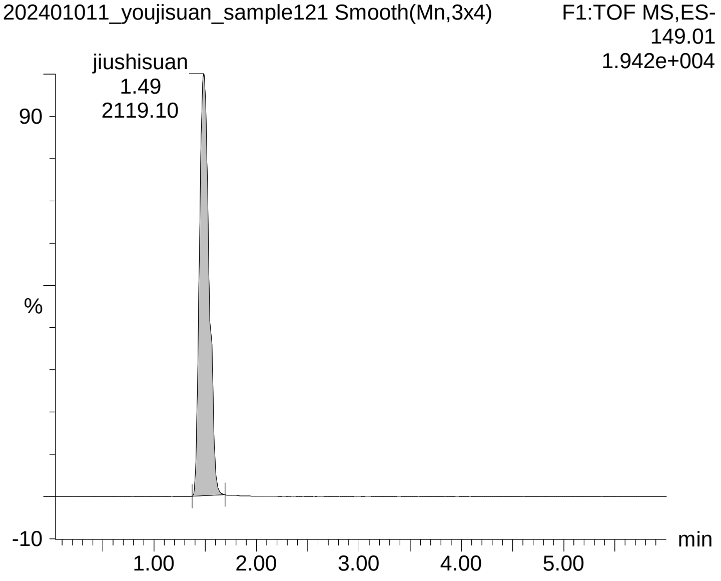

ningmensuan

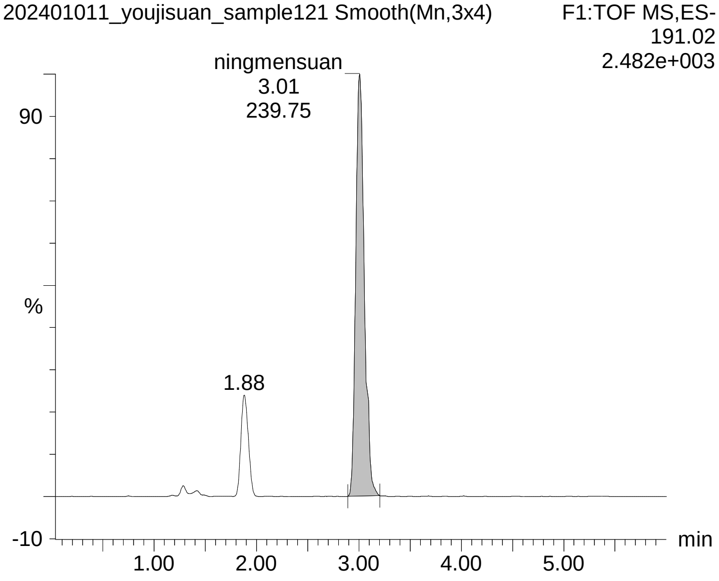

pinguosuan

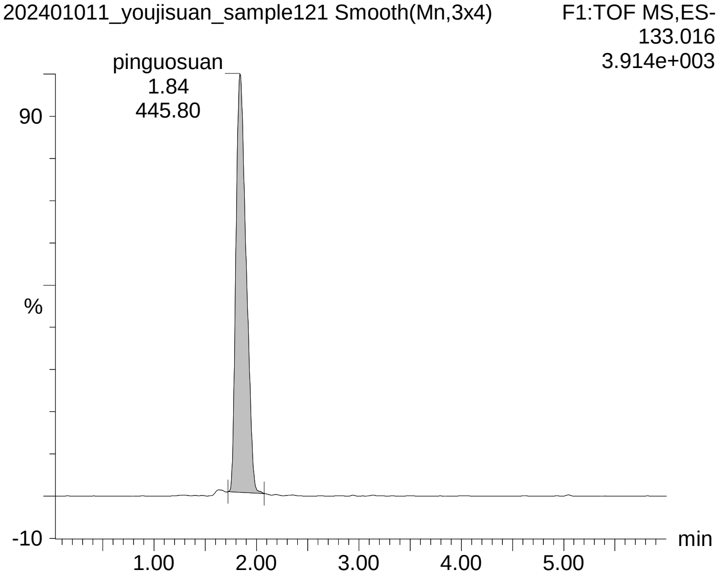

	#	Name	Sample Text	RT	Area	Std. Conc	Conc.
1	1	jiushisuan		1.49	2119.103		576.889621
2	2	ningmensuan		3.01	239.750		33.828783
3	3	pinguosuan		1.84	445.799		482.722732

Name: 202401011_youjisuan_sample122, Date: 11-Oct-2024, Time: 14:27:26, ID: , Description:

jiushisuan

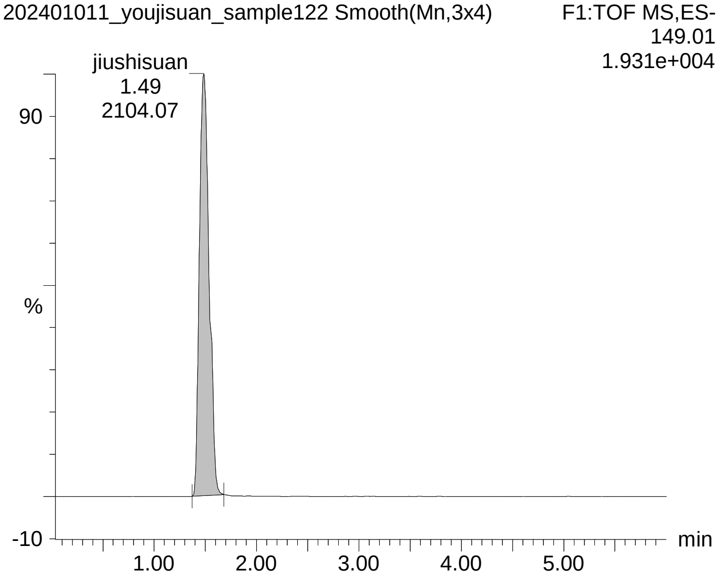

ningmensuan

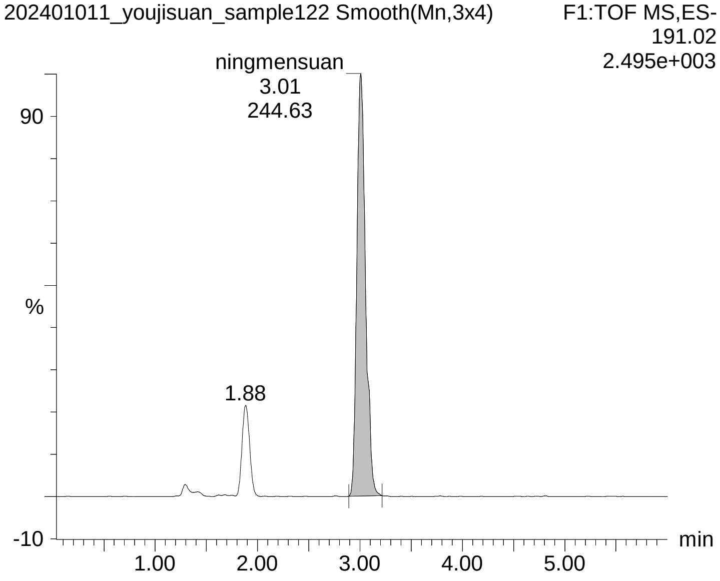

pinguosuan

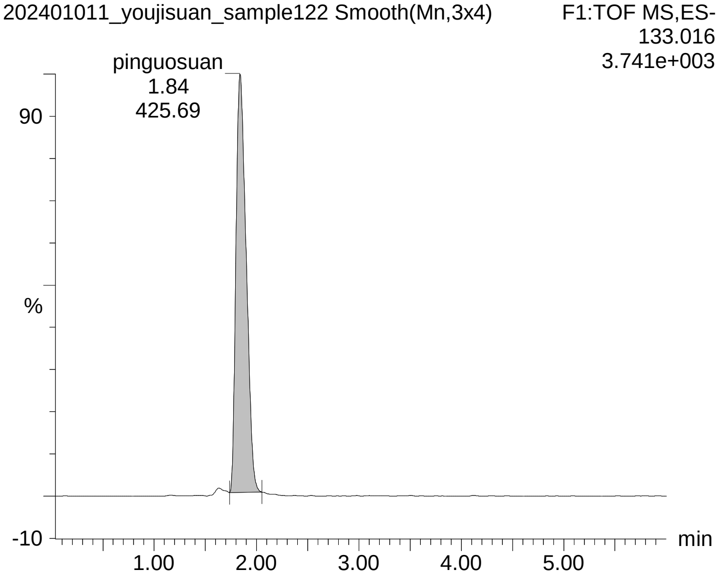

	#	Name	Sample Text	RT	Area	Std. Conc	Conc.
1	1	jiushisuan		1.49	2104.065		562.230801
2	2	ningmensuan		3.01	244.627		34.539560
3	3	pinguosuan		1.84	425.688		451.976229

Name: 202401011_youjisuan_sample123, Date: 11-Oct-2024, Time: 14:34:26, ID: , Description:

jiushisuan

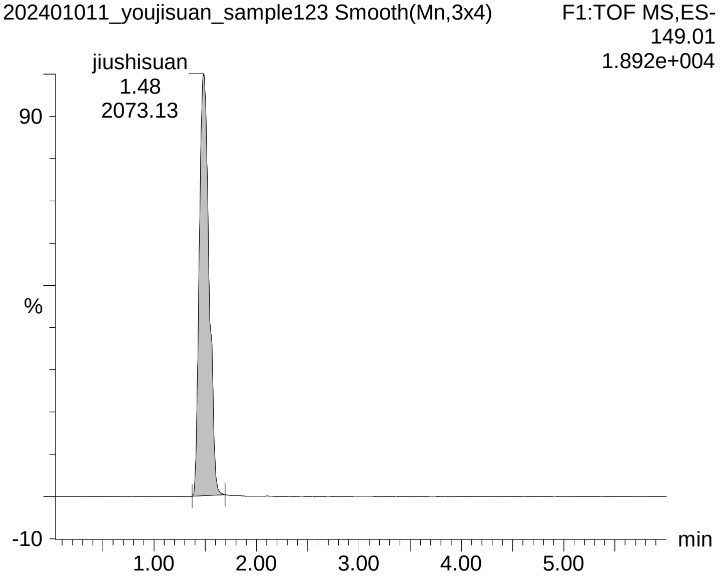

ningmensuan

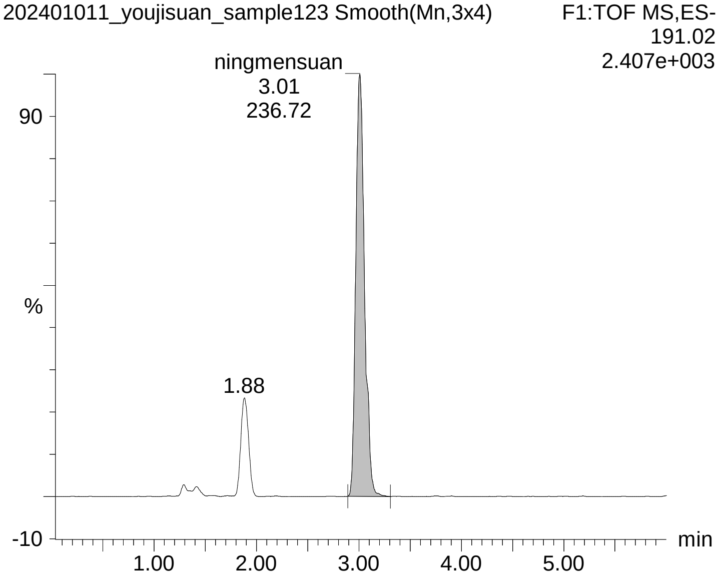

pinguosuan

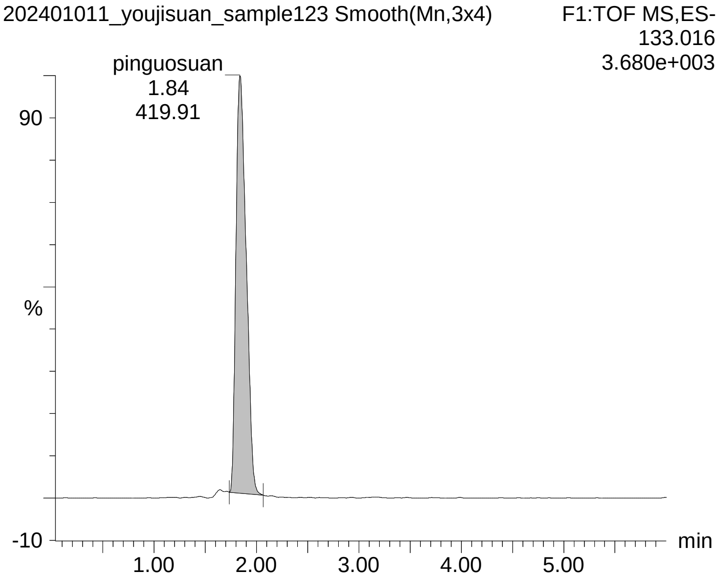

	#	Name	Sample Text	RT	Area	Std. Conc	Conc.
1	1	jiushisuan		1.48	2073.130		534.231884
2	2	ningmensuan		3.01	236.723		33.387626
3	3	pinguosuan		1.84	419.908		440.200049

Name: 202401011_youjisuan_sample124, Date: 11-Oct-2024, Time: 14:41:29, ID: , Description:

jiushisuan

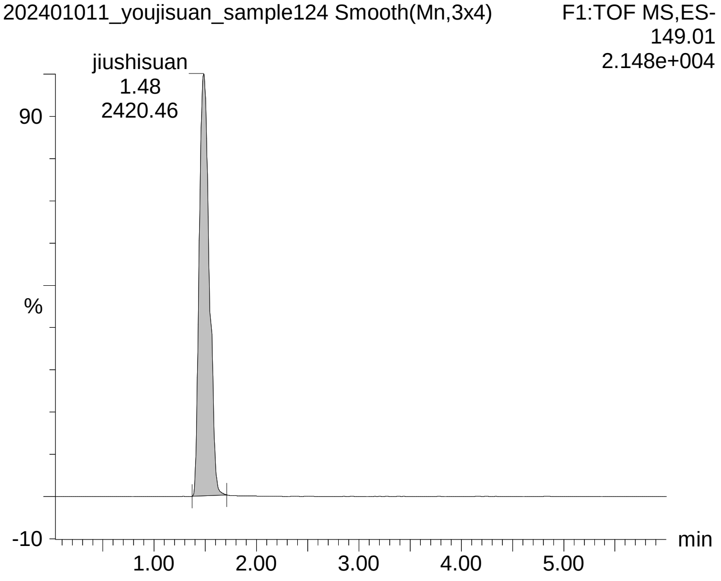

ningmensuan

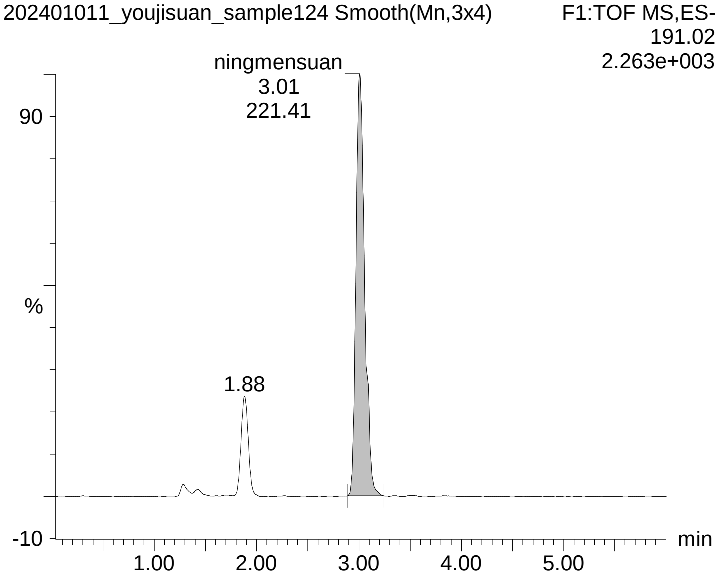

pinguosuan

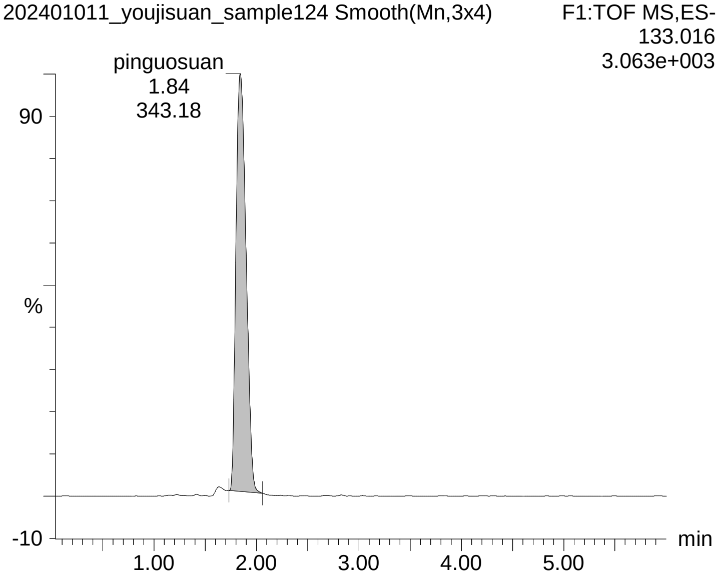

	#	Name	Sample Text	RT	Area	Std. Conc	Conc.
1	1	jiushisuan		1.48	2420.460		882.871324
2	2	ningmensuan		3.01	221.415		31.156629
3	3	pinguosuan		1.84	343.179		167.918616

Name: 202401011_youjisuan_sample125, Date: 11-Oct-2024, Time: 14:48:27, ID: , Description:

jiushisuan

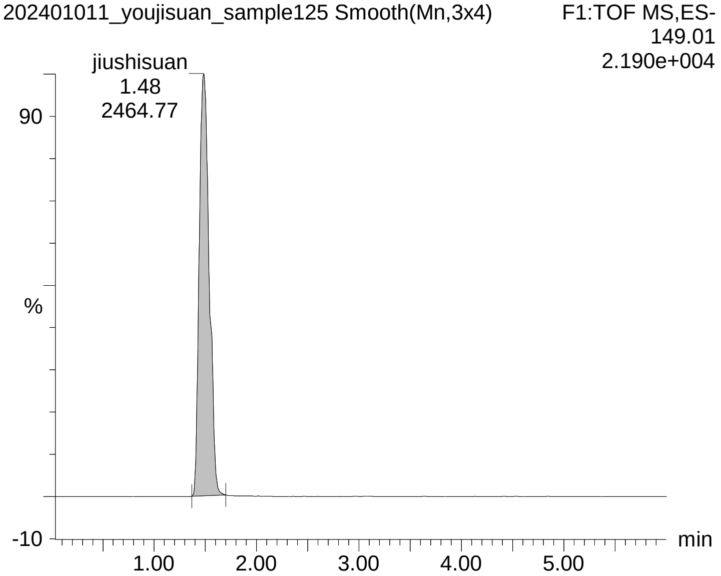

ningmensuan

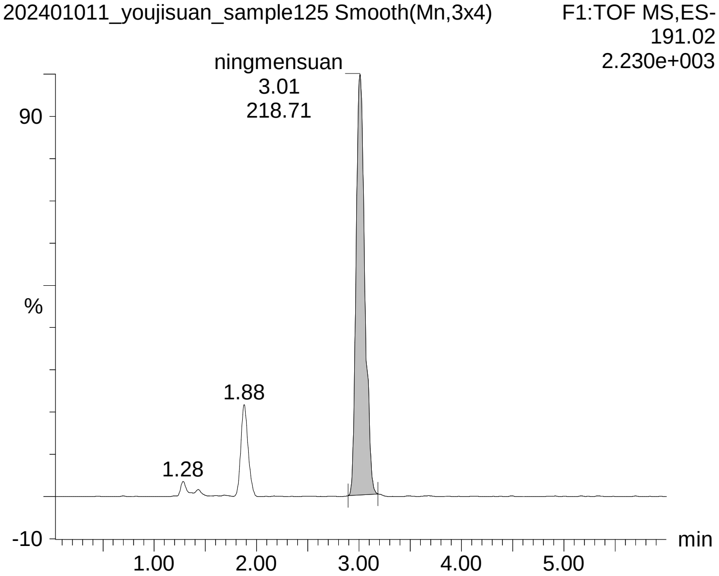

pinguosuan

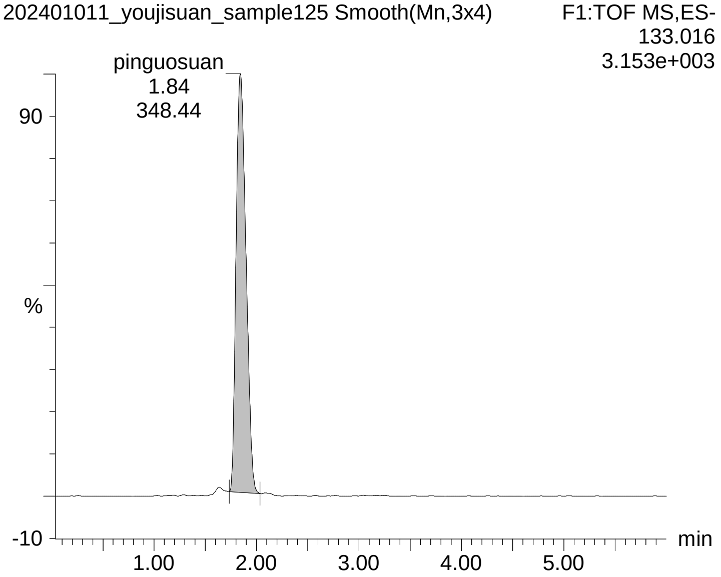

	#	Name	Sample Text	RT	Area	Std. Conc	Conc.
1	1	jiushisuan		1.48	2464.773		911.992843
2	2	ningmensuan		3.01	218.707		30.761963
3	3	pinguosuan		1.84	348.441		174.385395

Name: 202401011_youjisuan_sample126, Date: 11-Oct-2024, Time: 14:55:27, ID: , Description:

jiushisuan

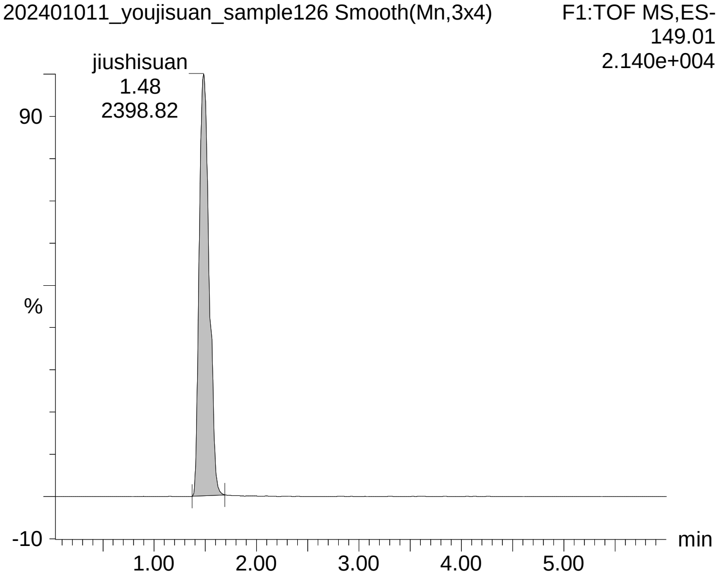

ningmensuan

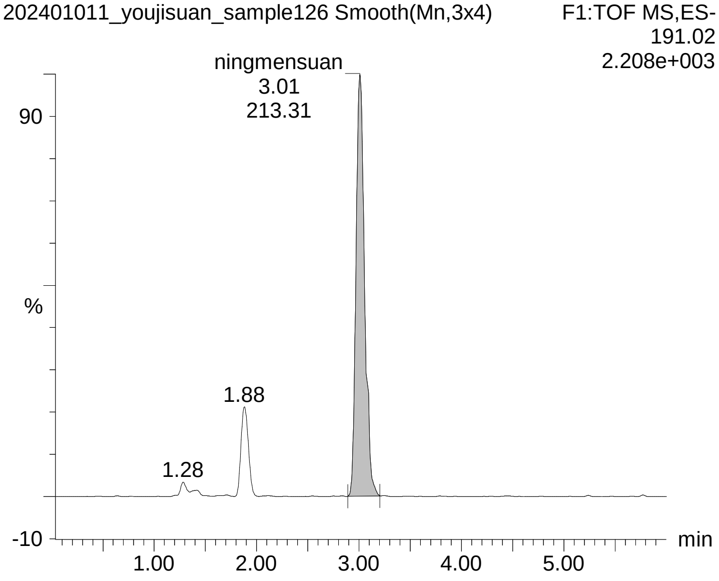

pinguosuan

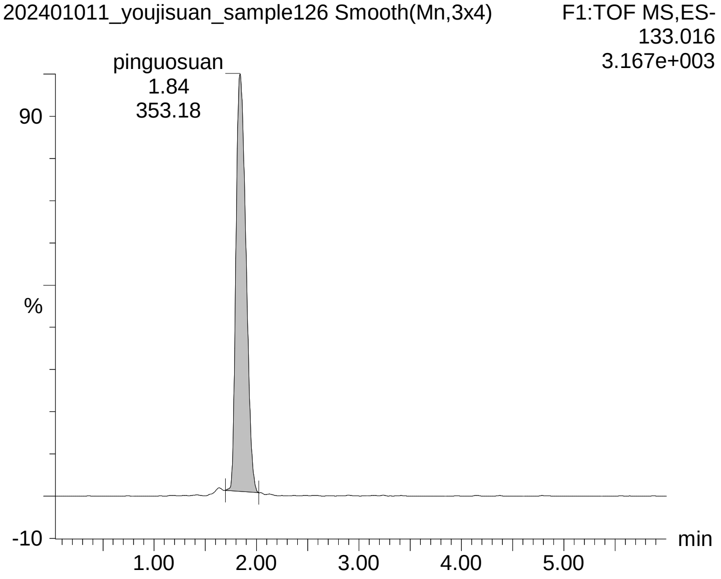

	#	Name	Sample Text	RT	Area	Std. Conc	Conc.
1	1	jiushisuan		1.48	2398.818		867.253836
2	2	ningmensuan		3.01	213.307		29.974964
3	3	pinguosuan		1.84	353.175		180.650275

Name: 202401011_youjisuan_sample127, Date: 11-Oct-2024, Time: 15:02:27, ID: , Description:

jiushisuan

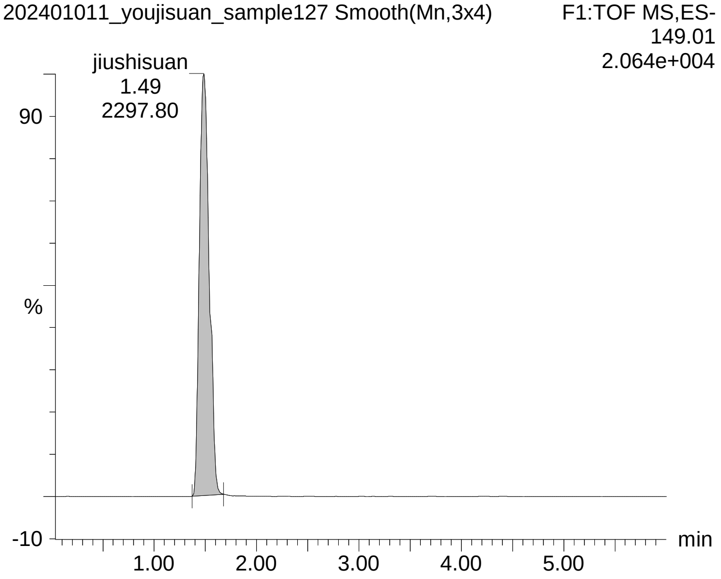

ningmensuan

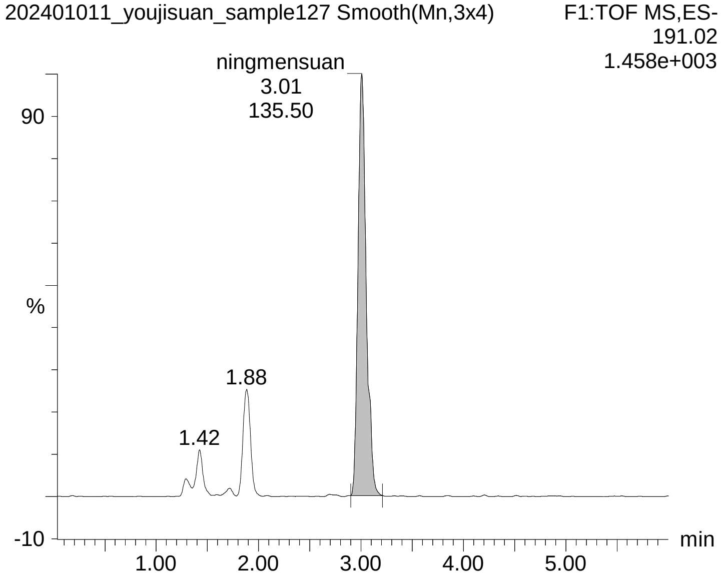

pinguosuan

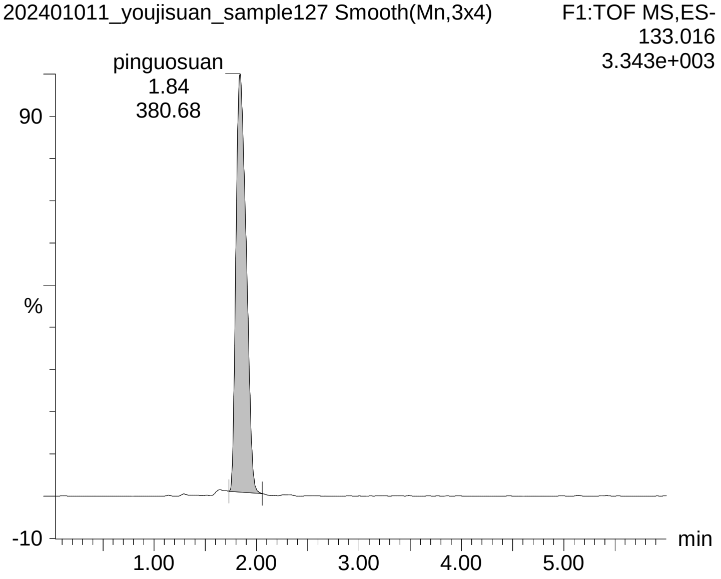

	#	Name	Sample Text	RT	Area	Std. Conc	Conc.
1	1	jiushisuan		1.49	2297.797		778.444248
2	2	ningmensuan		3.01	135.502		18.635616
3	3	pinguosuan		1.84	380.680		232.916371

Name: 202401011_youjisuan_sample128, Date: 11-Oct-2024, Time: 15:09:27, ID: , Description:

jiushisuan

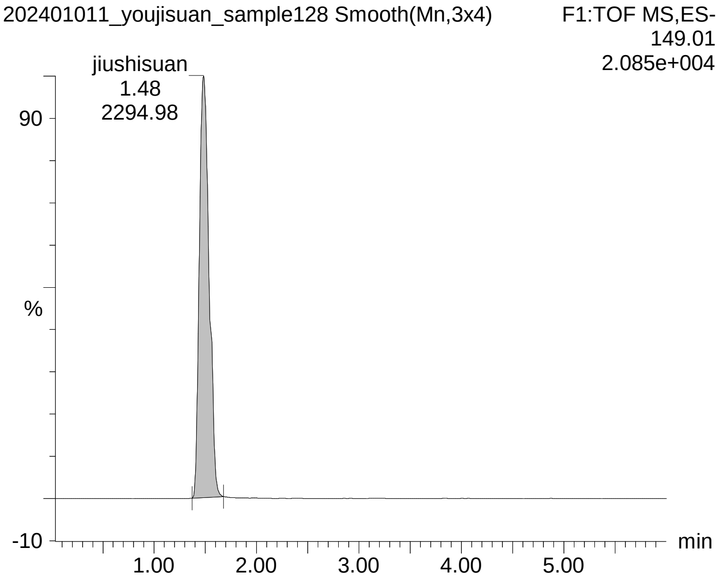

ningmensuan

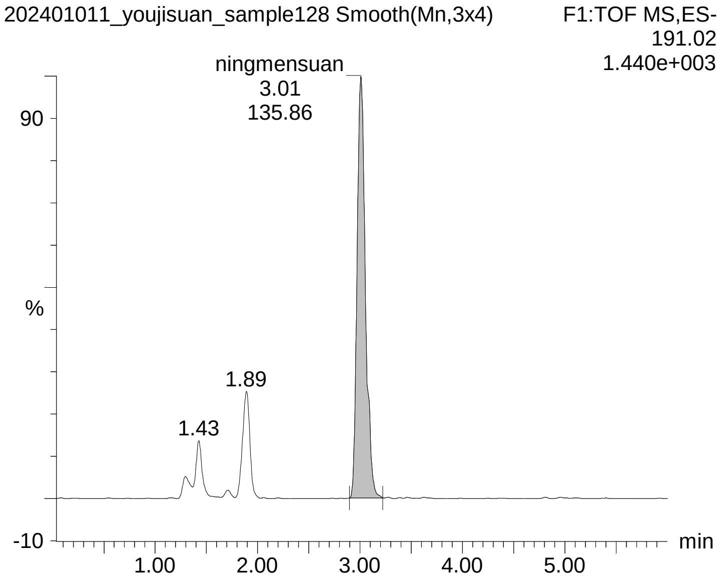

pinguosuan

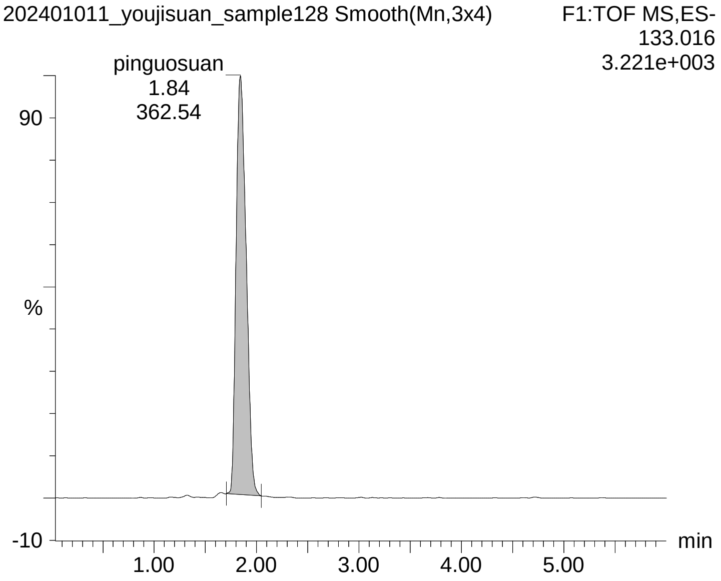

	#	Name	Sample Text	RT	Area	Std. Conc	Conc.
1	1	jiushisuan		1.48	2294.982		775.536626
2	2	ningmensuan		3.01	135.858		18.687499
3	3	pinguosuan		1.84	362.537		194.653478

project_wangzhonghua_BeiMu
Dataset: Untitled
Last Altered: Friday, October 11, 2024 17:06:05 China Standard Time
Printed: Friday, October 11, 2024 17:06:40 China Standard Time

Name: 202401011_youjisuan_sample129, Date: 11-Oct-2024, Time: 15:16:28, ID: , Description:

jiushisuan

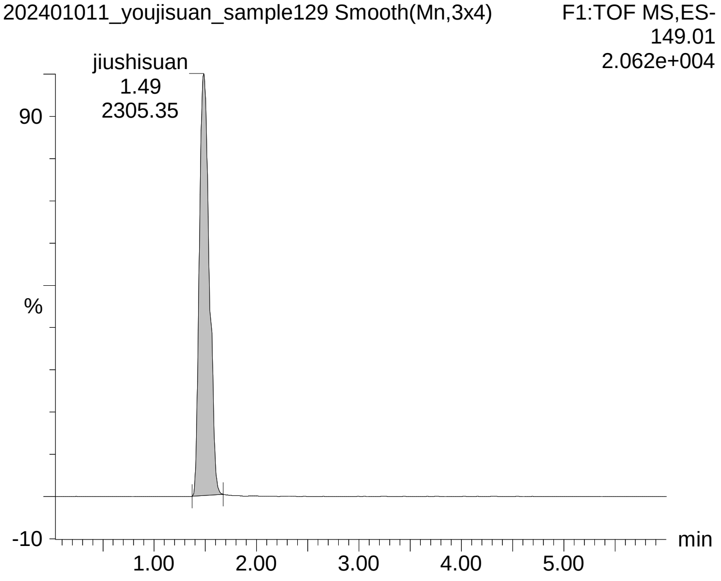

ningmensuan

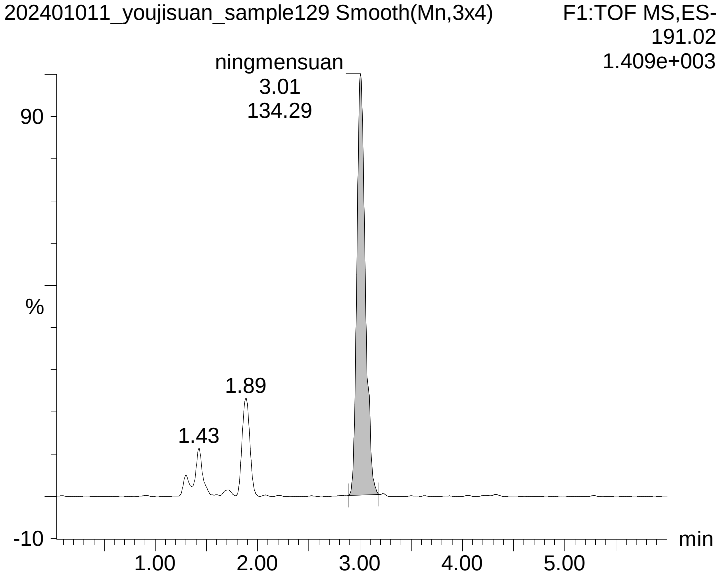

pinguosuan

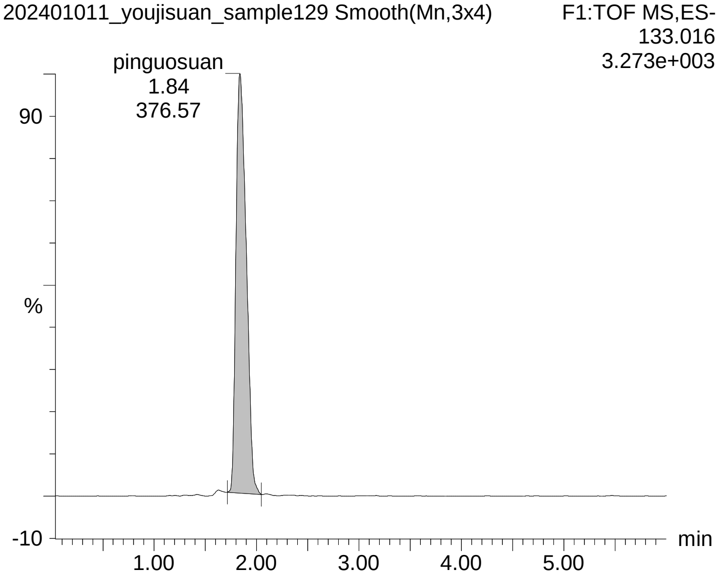

	#	Name	Sample Text	RT	Area	Std. Conc	Conc.
1	1	jiushisuan		1.49	2305.352		786.126384
2	2	ningmensuan		3.01	134.294		18.459561
3	3	pinguosuan		1.84	376.567		222.179160

Name: 202401011_youjisuan_sample130, Date: 11-Oct-2024, Time: 15:23:28, ID: , Description:

jiushisuan

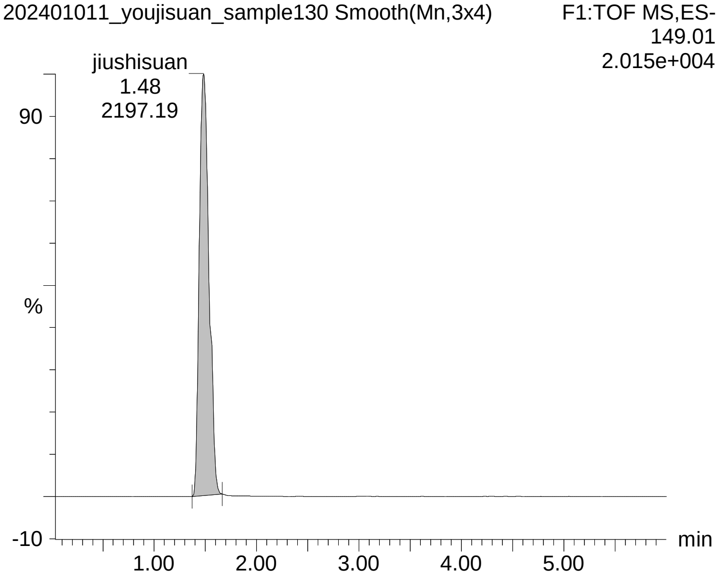

ningmensuan

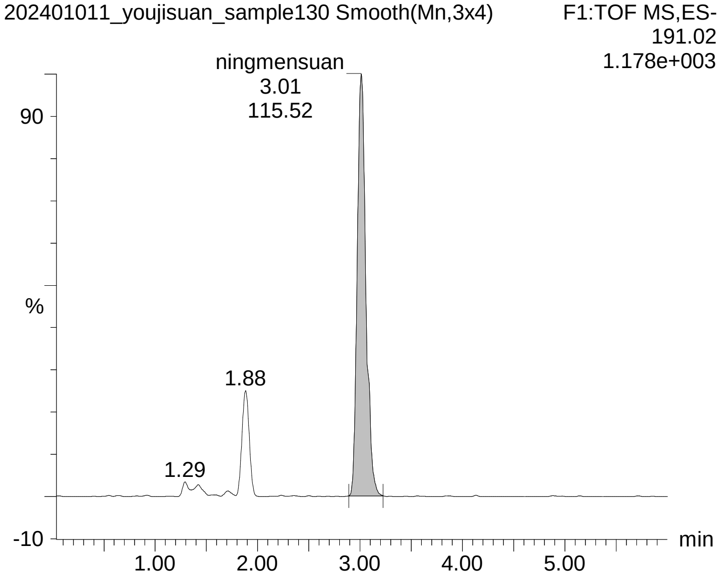

pinguosuan

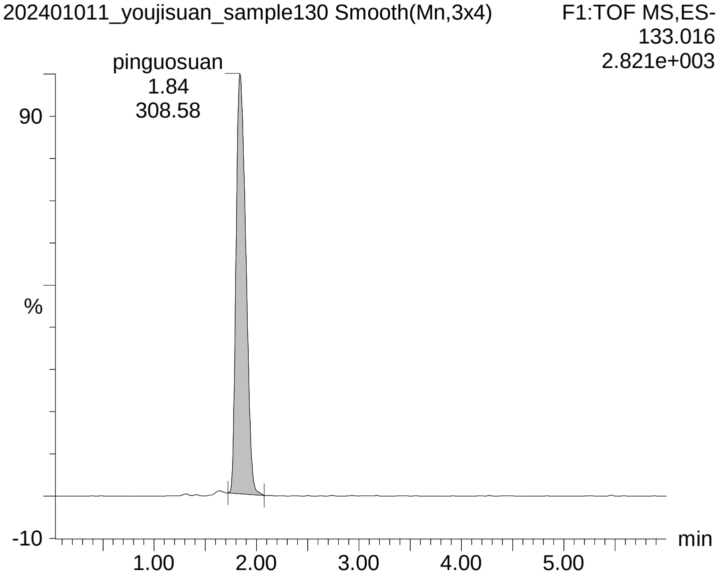

	#	Name	Sample Text	RT	Area	Std. Conc	Conc.
1	1	jiushisuan		1.48	2197.194		663.352911
2	2	ningmensuan		3.01	115.524		15.724010
3	3	pinguosuan		1.84	308.583		133.899939

Name: 202401011_youjisuan_sample131, Date: 11-Oct-2024, Time: 15:30:28, ID: , Description:

jiushisuan

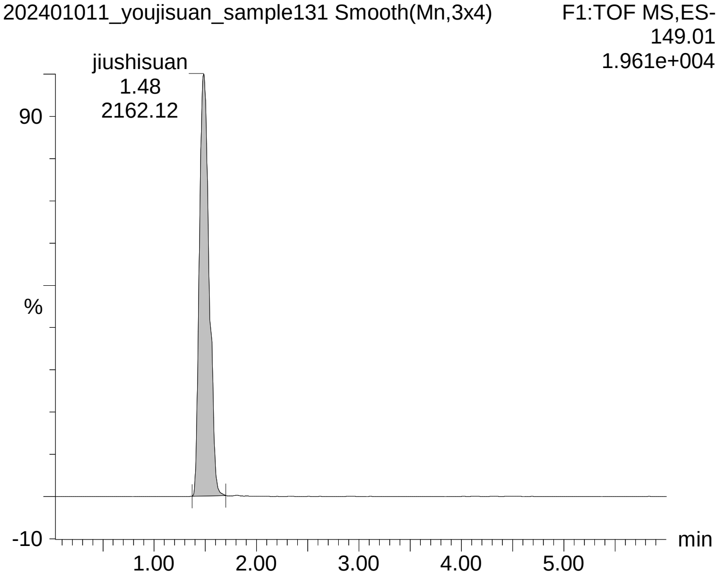

ningmensuan

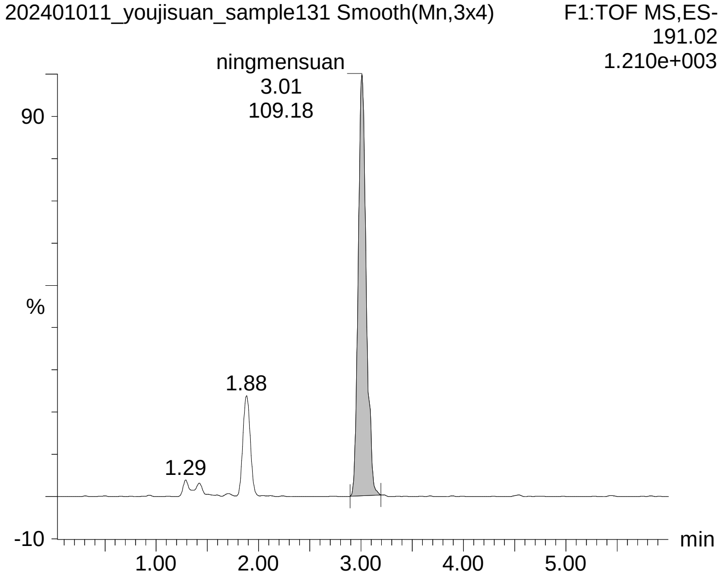

pinguosuan

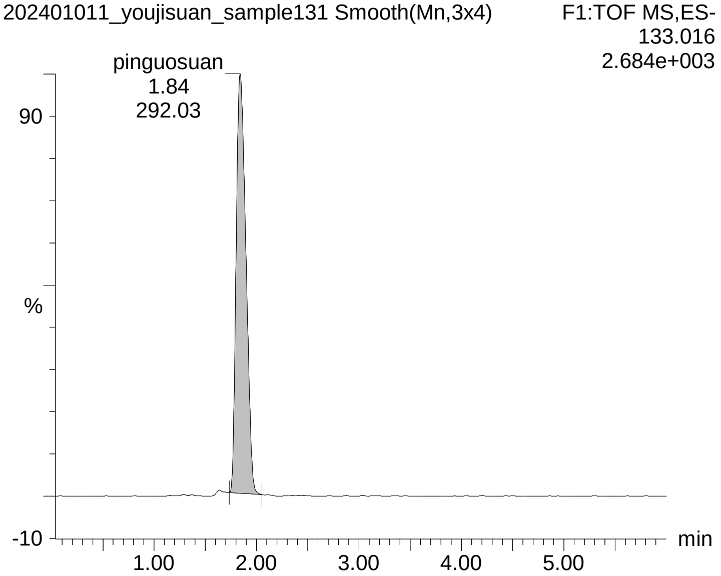

	#	Name	Sample Text	RT	Area	Std. Conc	Conc.
1	1	jiushisuan		1.48	2162.121		622.633299
2	2	ningmensuan		3.01	109.180		14.799432
3	3	pinguosuan		1.84	292.030		120.943412

Name: 202401011_youjisuan_sample132, Date: 11-Oct-2024, Time: 15:37:28, ID: , Description:

jiushisuan

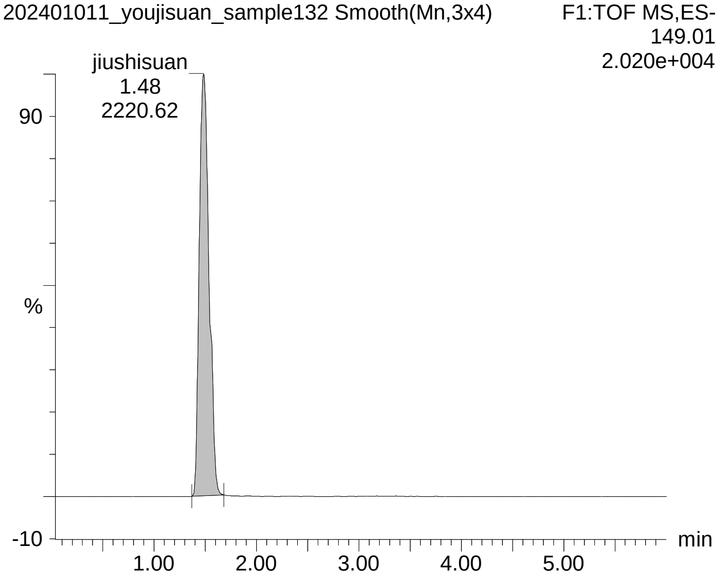

ningmensuan

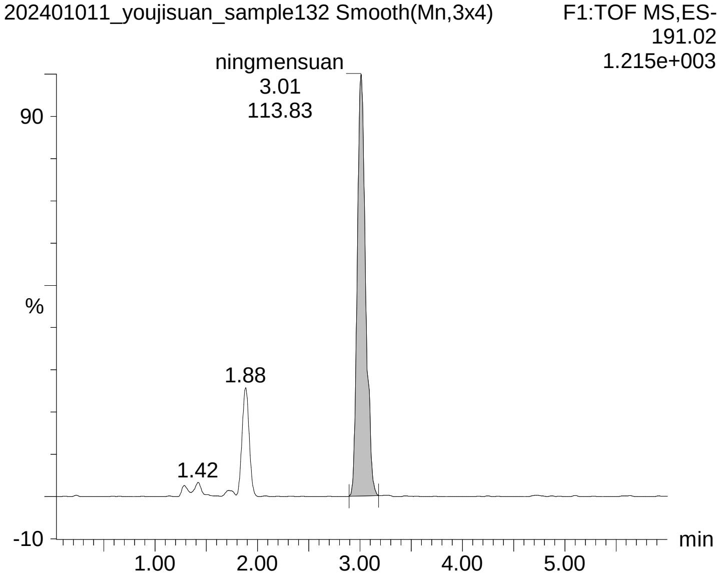

pinguosuan

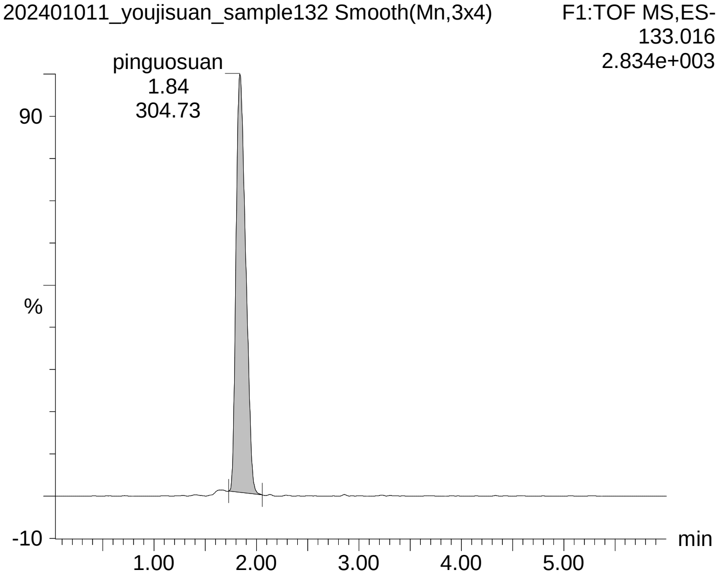

	#	Name	Sample Text	RT	Area	Std. Conc	Conc.
1	1	jiushisuan		1.48	2220.621		691.329714
2	2	ningmensuan		3.01	113.827		15.476688
3	3	pinguosuan		1.84	304.725		130.736758

Name: 202401011_youjisuan_sample133, Date: 11-Oct-2024, Time: 15:45:25, ID: , Description:

jiushisuan

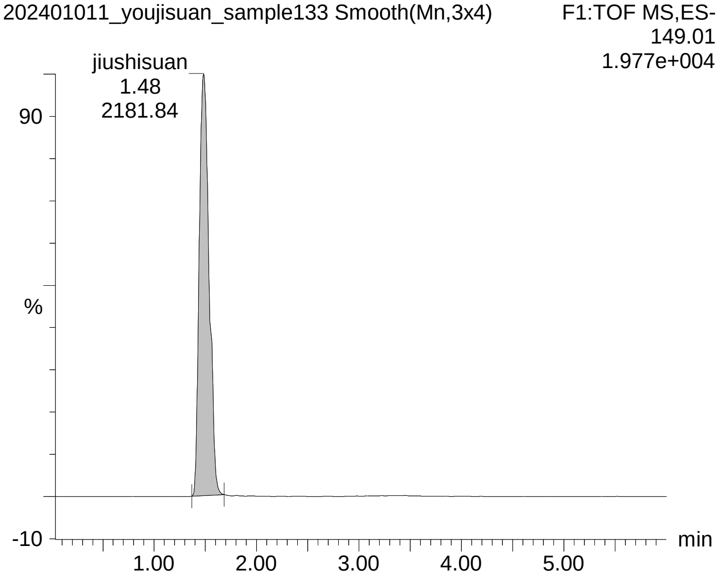

ningmensuan

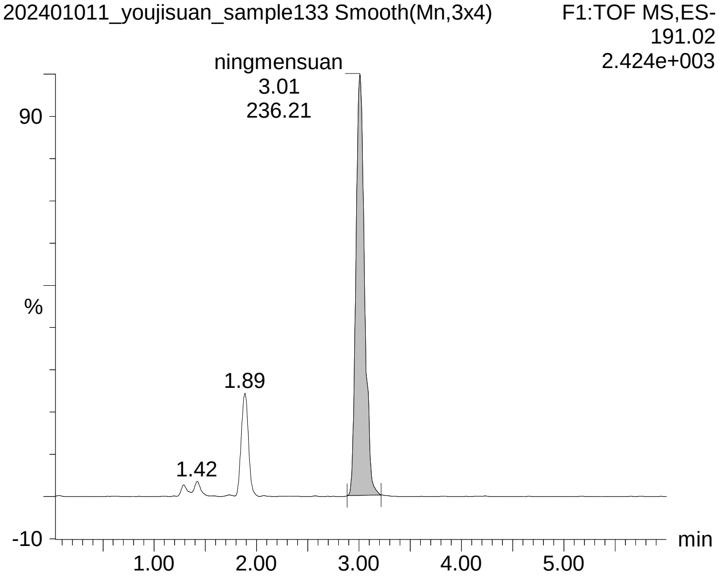

pinguosuan

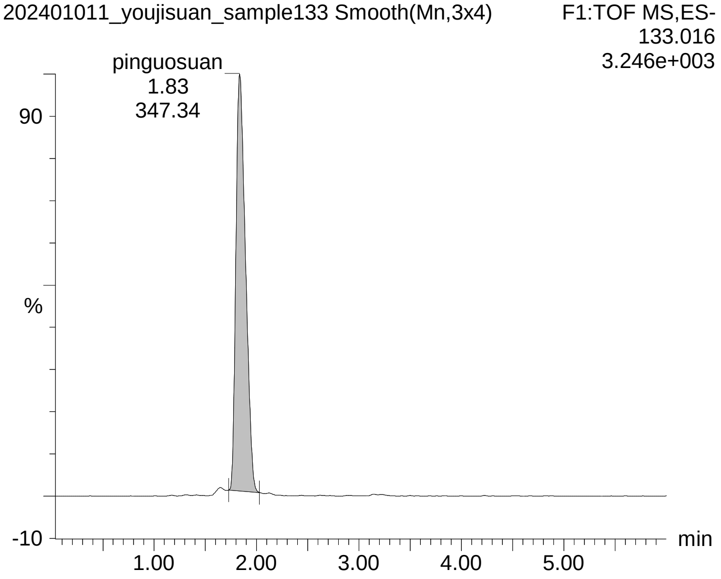

	#	Name	Sample Text	RT	Area	Std. Conc	Conc.
1	1	jiushisuan		1.48	2181.838		645.250571
2	2	ningmensuan		3.01	236.209		33.312715
3	3	pinguosuan		1.83	347.343		172.995807

Name: 202401011_youjisuan_sample134, Date: 11-Oct-2024, Time: 15:52:28, ID: , Description:

jiushisuan

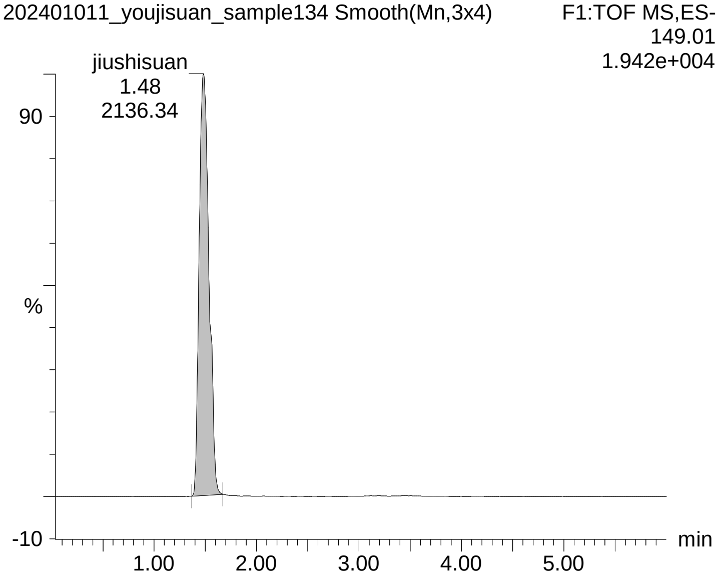

ningmensuan

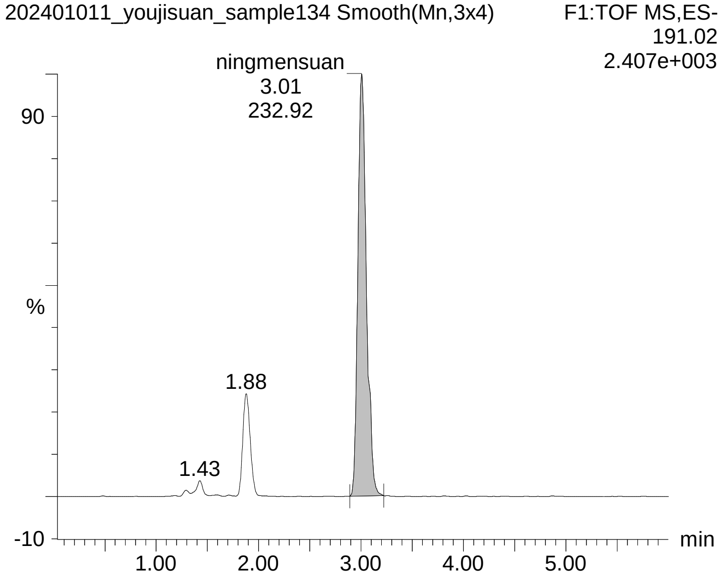

pinguosuan

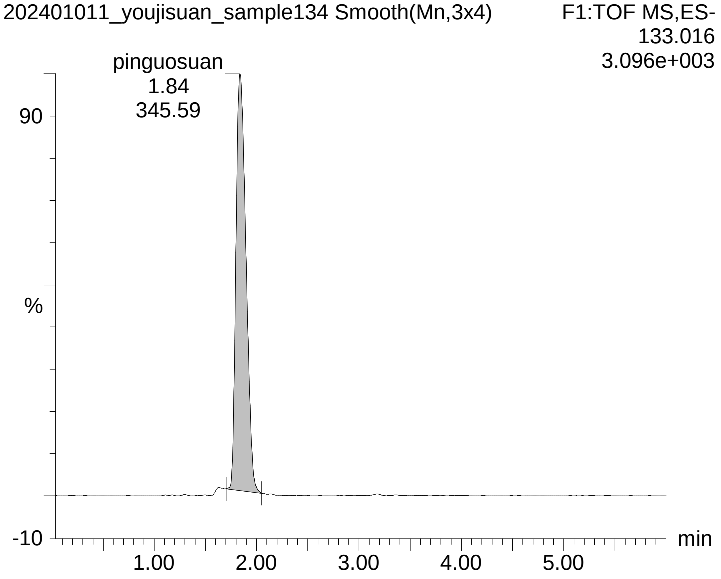

	#	Name	Sample Text	RT	Area	Std. Conc	Conc.
1	1	jiushisuan		1.48	2136.345		594.562141
2	2	ningmensuan		3.01	232.921		32.833520
3	3	pinguosuan		1.84	345.586		170.817524

Name: 202401011_youjisuan_sample135, Date: 11-Oct-2024, Time: 15:59:27, ID: , Description:

jiushisuan

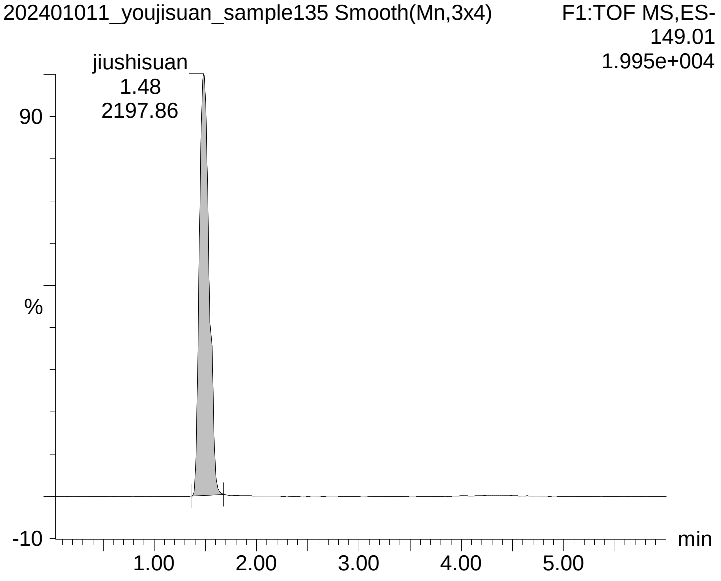

ningmensuan

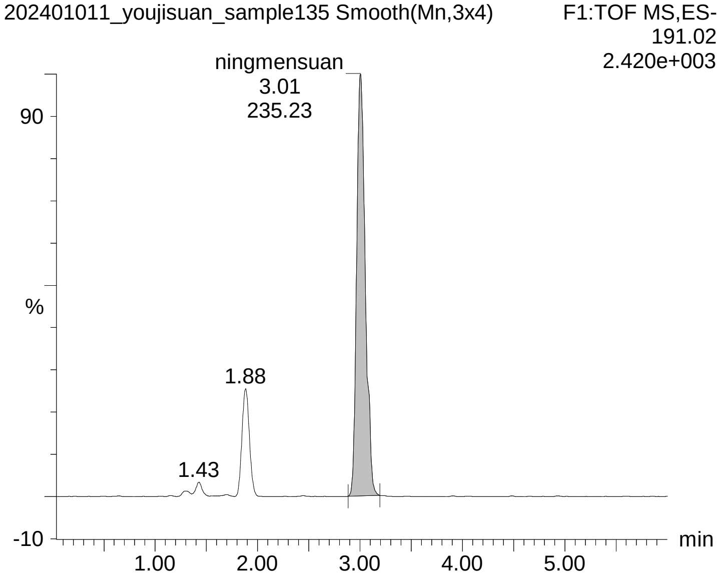

pinguosuan

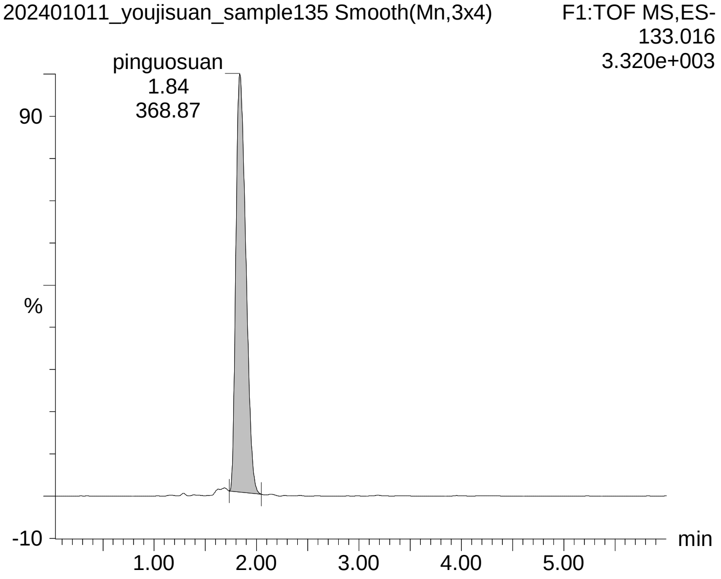

	#	Name	Sample Text	RT	Area	Std. Conc	Conc.
1	1	jiushisuan		1.48	2197.863		664.148136
2	2	ningmensuan		3.01	235.229		33.169890
3	3	pinguosuan		1.84	368.870		205.797070

Name: 202401011_youjisuan_sample136, Date: 11-Oct-2024, Time: 16:06:28, ID: , Description:

jiushisuan

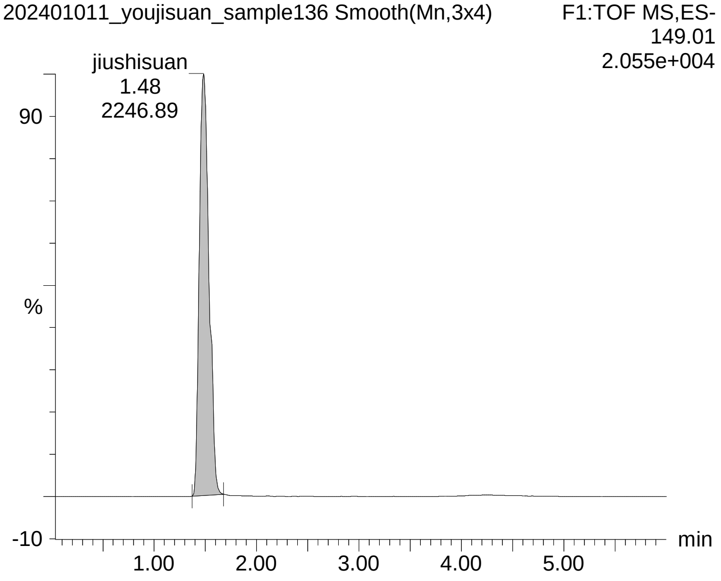

ningmensuan

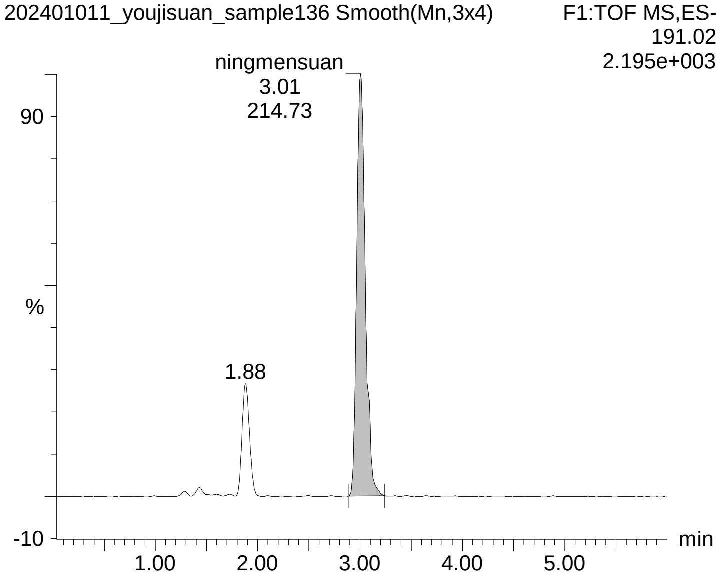

pinguosuan

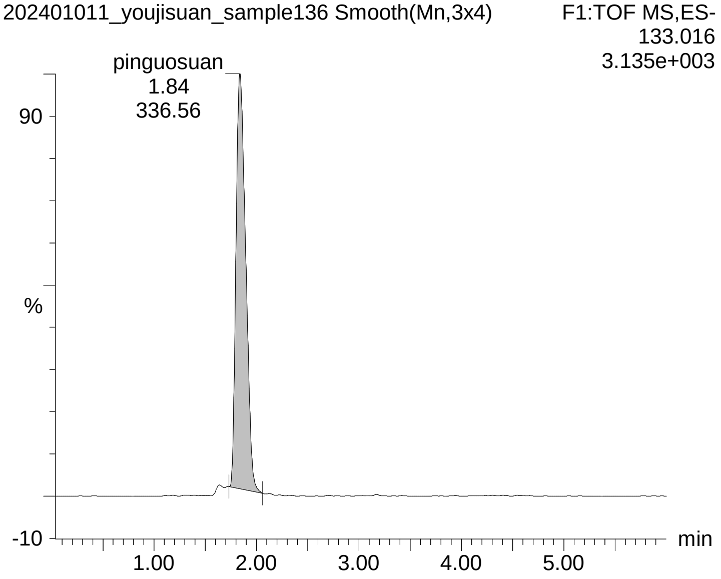

	#	Name	Sample Text	RT	Area	Std. Conc	Conc.
1	1	jiushisuan		1.48	2246.887		722.380545
2	2	ningmensuan		3.01	214.729		30.182207
3	3	pinguosuan		1.84	336.561		160.400388

project_wangzhonghua_BeiMu
Dataset: Untitled
Last Altered: Saturday, October 12, 2024 07:50:19 China Standard Time
Printed: Saturday, October 12, 2024 07:50:41 China Standard Time

Method: F:\data\Wu_yueyan.PRO\MethDB\20241011_ organic acid .mdb 11 Oct 2024 15:52:39
Calibration: F:\data\zhanghuien.PRO\CurveDB\20241011_organic acid003.cdb 11 Oct 2024 15:39:56

Compound name: jiushisuan
Coefficient of Determination: R^2 = 0.994685
Calibration curve: 4.94514e-006 * x^3 + -0.0101268 * x^2 + 7.74871 * x + 69.7696
Response type: External Std, Area
Curve type: 3rd Order, Origin: Include, Weighting: Null, Axis trans: None

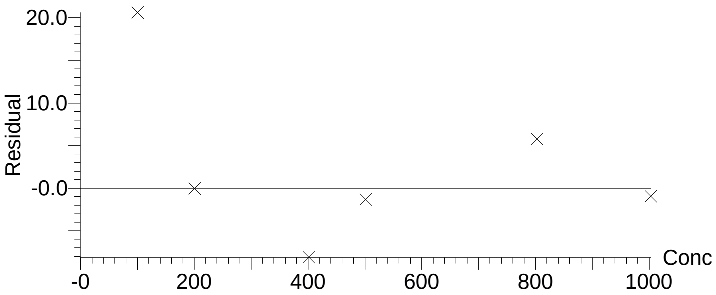

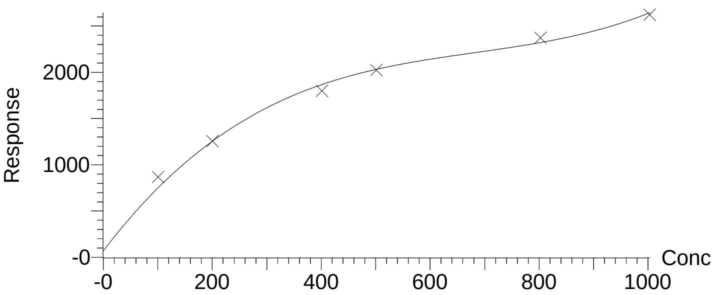

Compound name: ningmensuan
Correlation coefficient: r = 0.998196, r^2 = 0.996396
Calibration curve: 6.86151 * x + 7.63362
Response type: External Std, Area
Curve type: Linear, Origin: Include, Weighting: Null, Axis trans: None

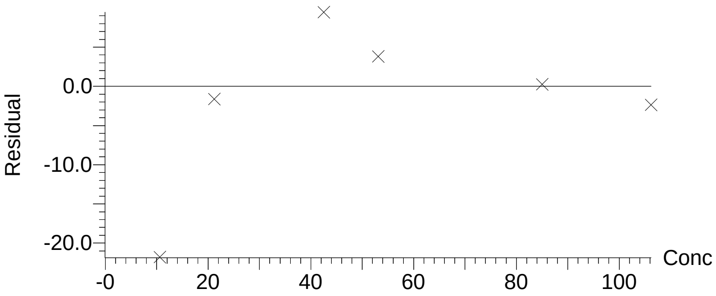

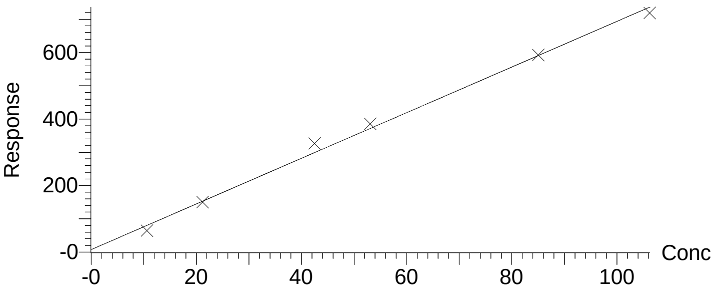

Compound name: pinguosuan
Coefficient of Determination: R^2 = 0.984709
Calibration curve: 9.85928e-006 * x^3 + -0.00971593 * x^2 + 3.27297 * x + 20.8616
Response type: External Std, Area
Curve type: 3rd Order, Origin: Include, Weighting: Null, Axis trans: None

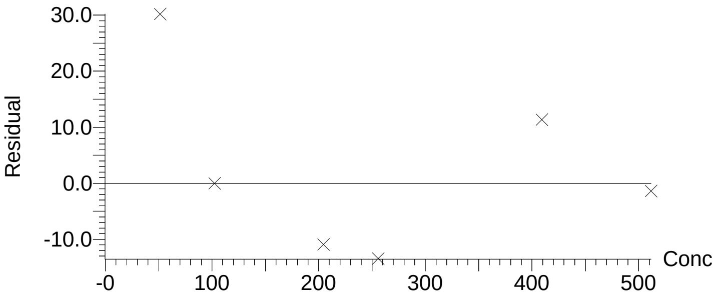

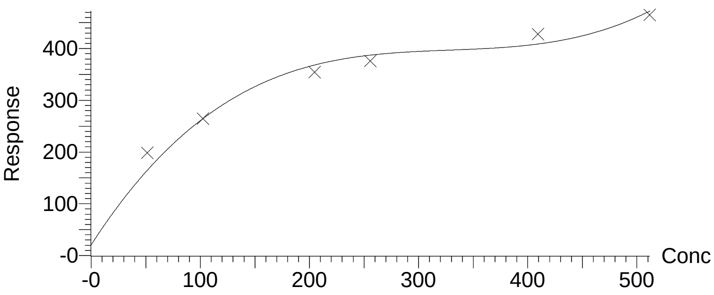

Compound name: huposaun
Coefficient of Determination: R^2 = 0.999608
Calibration curve: 3.18016e-005 * x^3 + -0.0146291 * x^2 + 2.726 * x + 0.728245
Response type: External Std, Area
Curve type: 3rd Order, Origin: Include, Weighting: Null, Axis trans: None

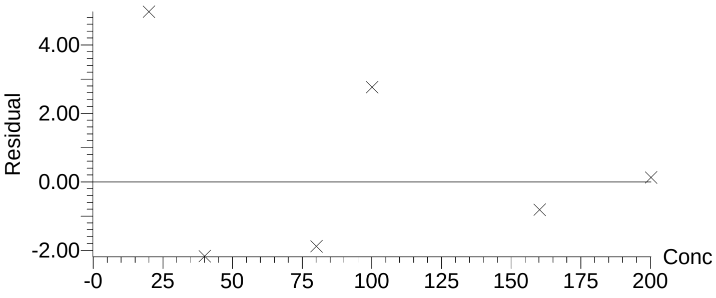

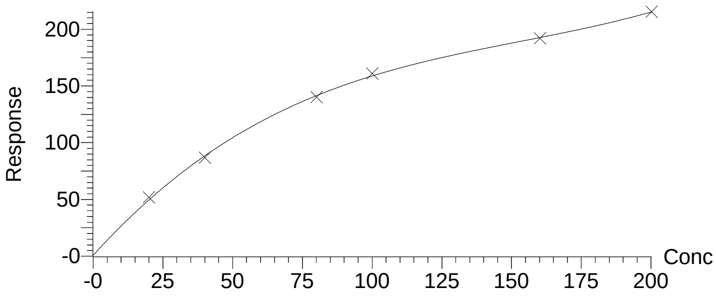

Method: F:\data\Wu_yueyan.PRO\MethDB\20241011_ organic acid .mdb 11 Oct 2024 15:52:39
Calibration: F:\data\zhanghuien.PRO\CurveDB\20241011_organic acid003.cdb 11 Oct 2024 15:39:56

Name: 202401011_youjisuan_sample137, Date: 11-Oct-2024, Time: 16:59:59, ID: , Description:

jiushisuan

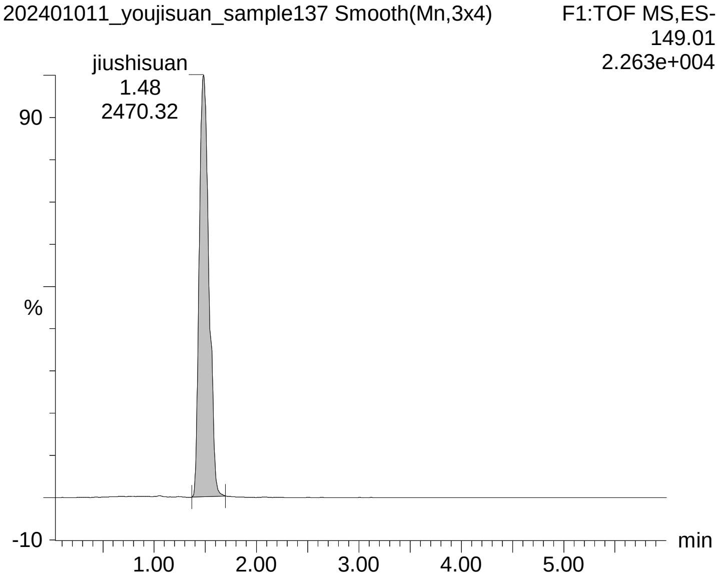

ningmensuan

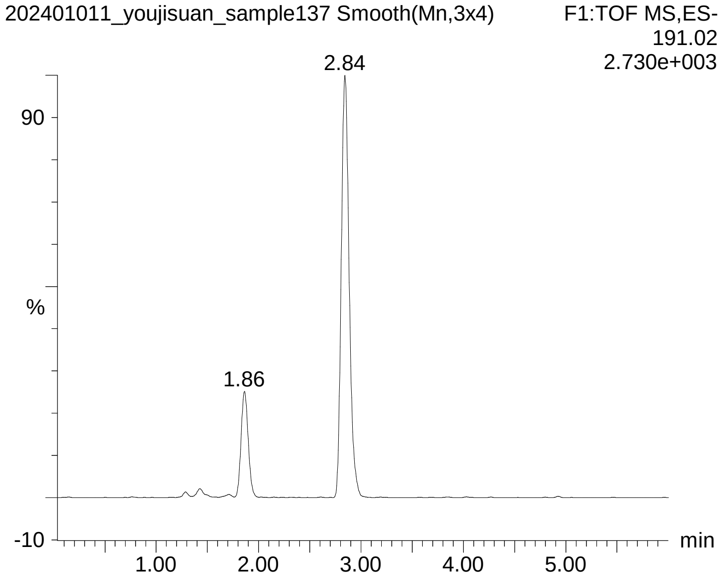

pinguosuan

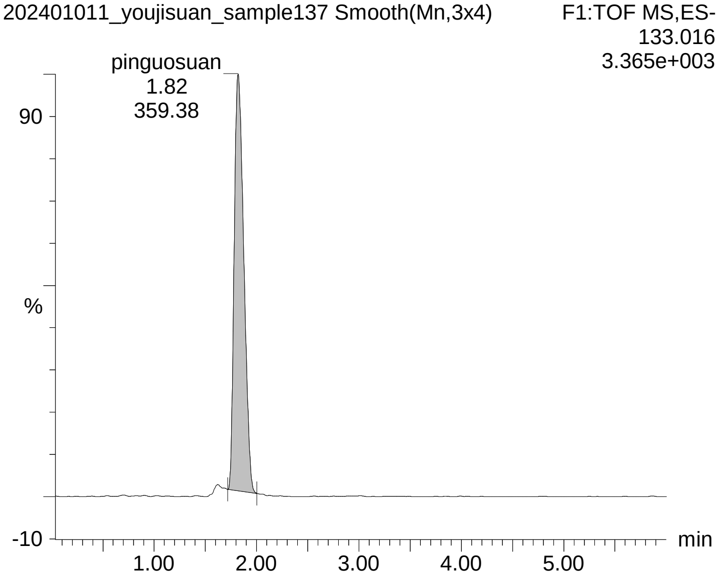

	#	Name	Sample Text	RT	Area	Std. Conc	Conc.
1	1	jiushisuan		1.48	2470.325		915.402692
2	2	ningmensuan					
3	3	pinguosuan		1.82	359.379		189.650679

Name: 202401011_youjisuan_sample138, Date: 11-Oct-2024, Time: 17:07:28, ID: , Description:

jiushisuan

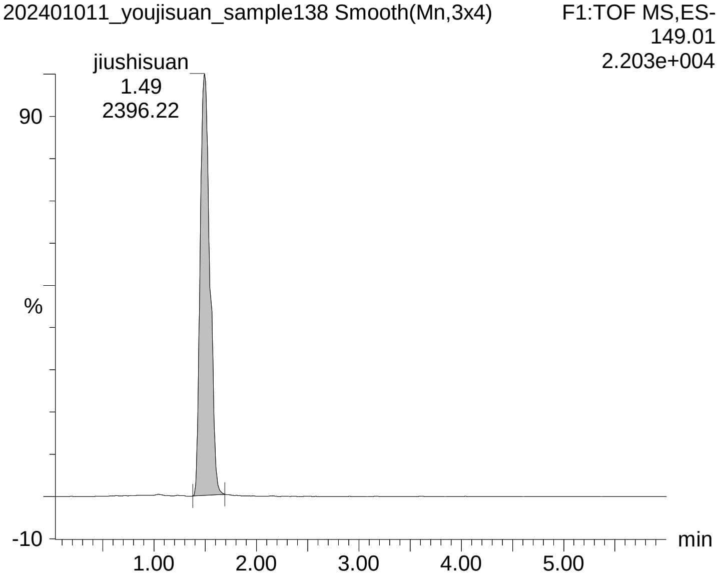

ningmensuan

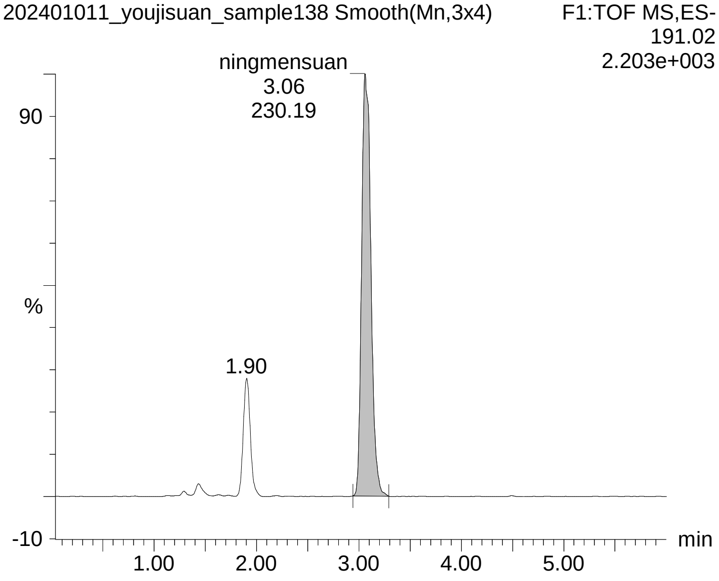

pinguosuan

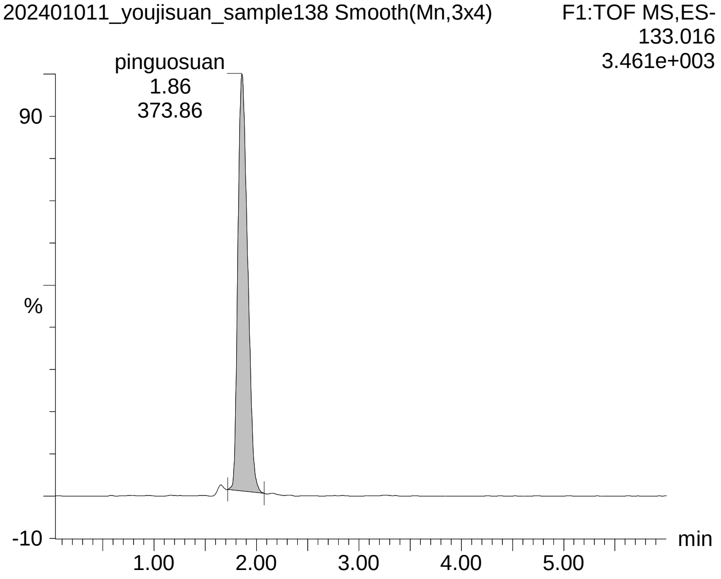

	#	Name	Sample Text	RT	Area	Std. Conc	Conc.
1	1	jiushisuan		1.49	2396.215		865.306142
2	2	ningmensuan		3.06	230.188		32.435211
3	3	pinguosuan		1.86	373.862		215.969218

Name: 202401011_youjisuan_sample139, Date: 11-Oct-2024, Time: 17:14:29, ID: , Description:

jiushisuan

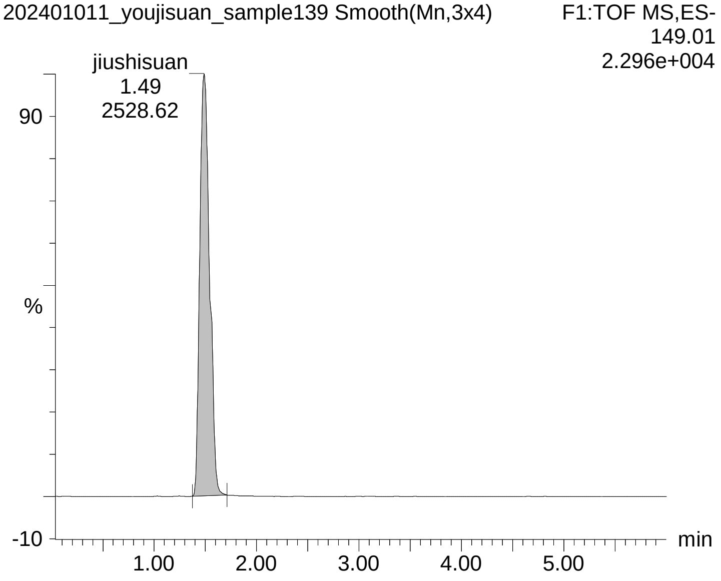

ningmensuan

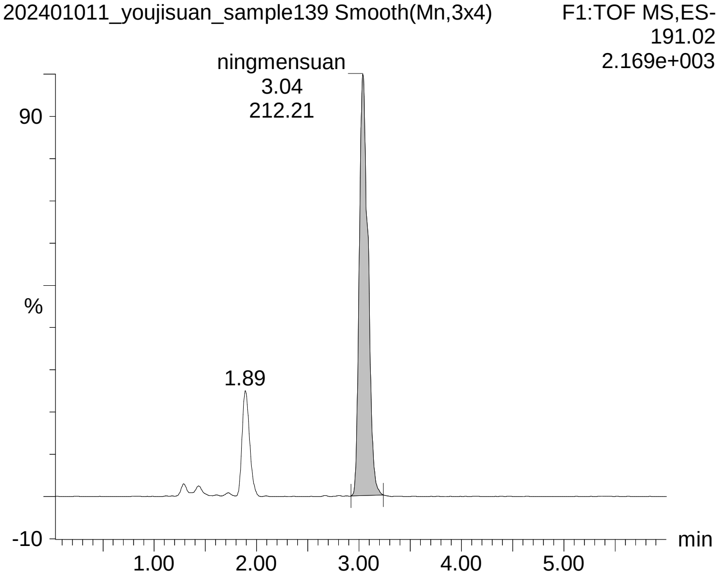

pinguosuan

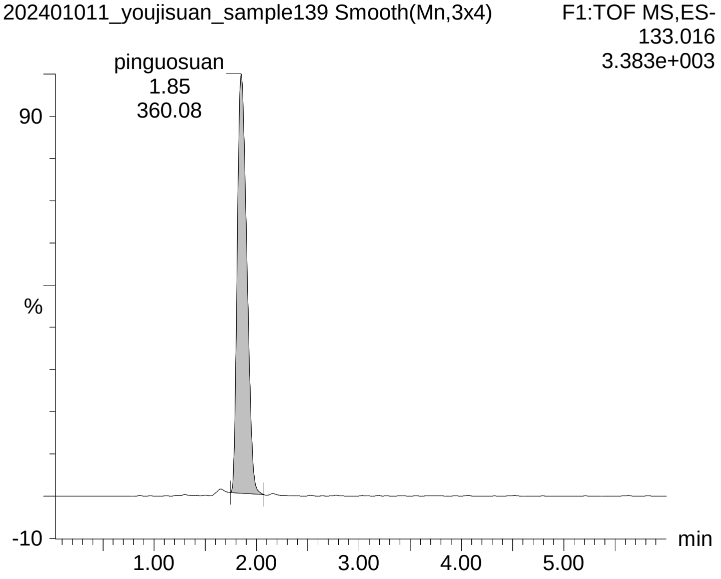

	#	Name	Sample Text	RT	Area	Std. Conc	Conc.
1	1	jiushisuan		1.49	2528.621		948.529646
2	2	ningmensuan		3.04	212.212		29.815378
3	3	pinguosuan		1.85	360.083		190.738630

Name: 202401011_youjisuan_sample140, Date: 11-Oct-2024, Time: 17:21:29, ID: , Description:

jiushisuan

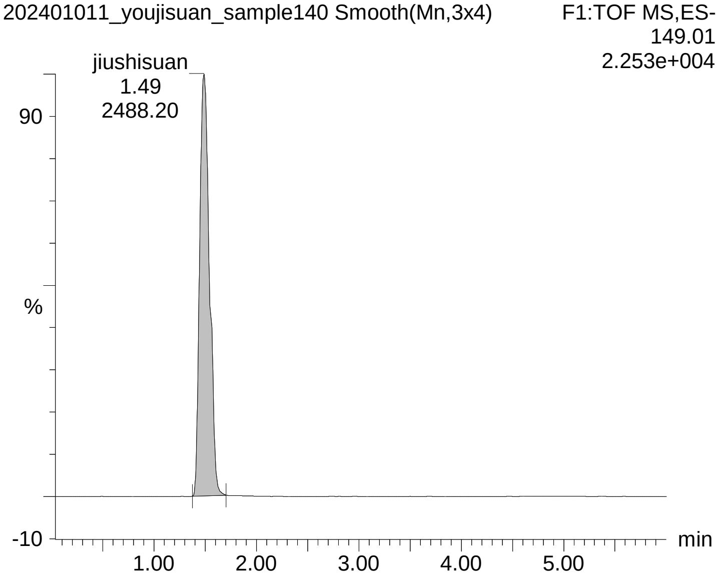

ningmensuan

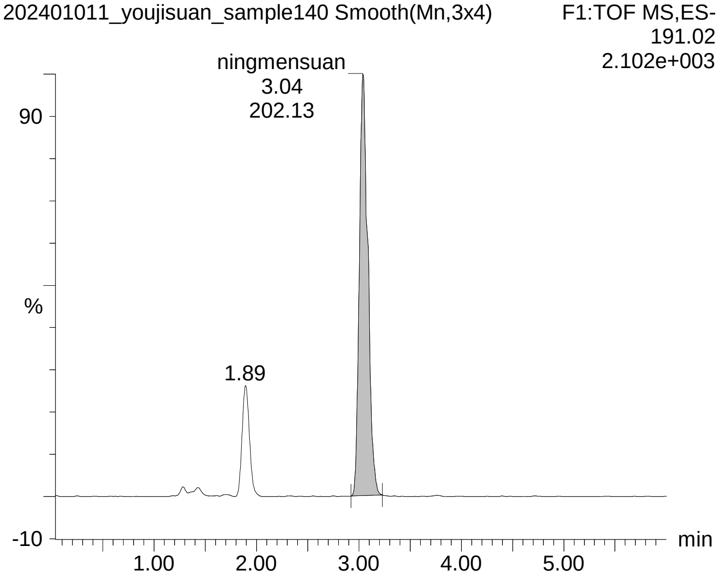

pinguosuan

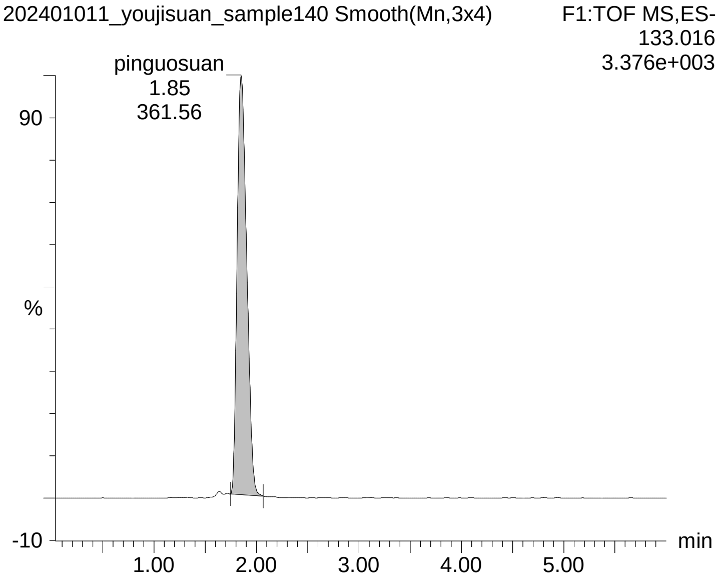

	#	Name	Sample Text	RT	Area	Std. Conc	Conc.
1	1	jiushisuan		1.49	2488.205		926.062557
2	2	ningmensuan		3.04	202.135		28.346750
3	3	pinguosuan		1.85	361.563		193.076061

project_wangzhonghua_BeiMu
Dataset: Untitled
Last Altered: Saturday, October 12, 2024 07:50:19 China Standard Time
Printed: Saturday, October 12, 2024 07:50:41 China Standard Time

Name: 202401011_youjisuan_sample141, Date: 11-Oct-2024, Time: 17:28:29, ID: , Description:

jiushisuan

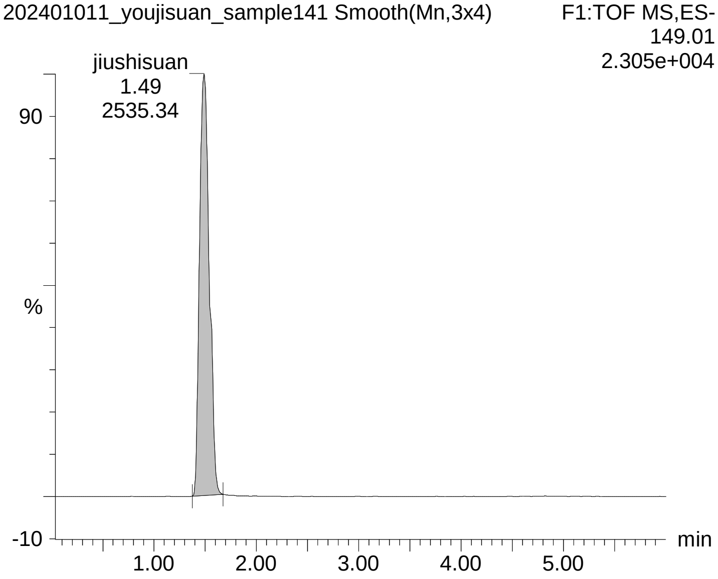

ningmensuan

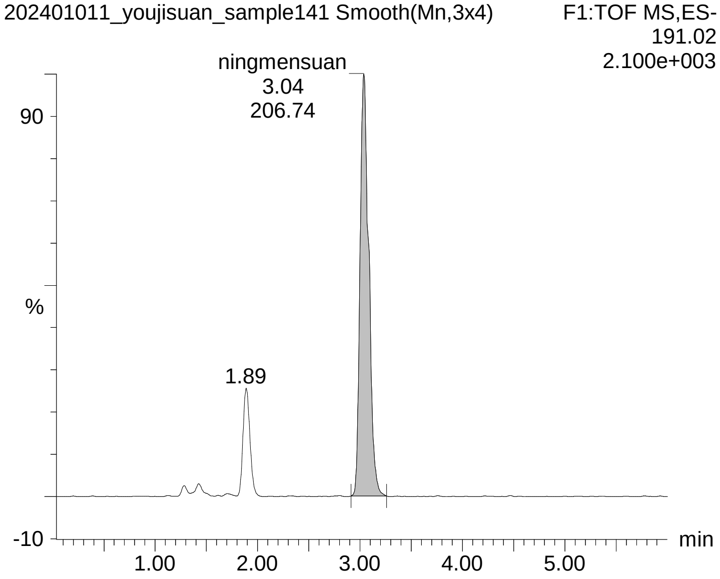

pinguosuan

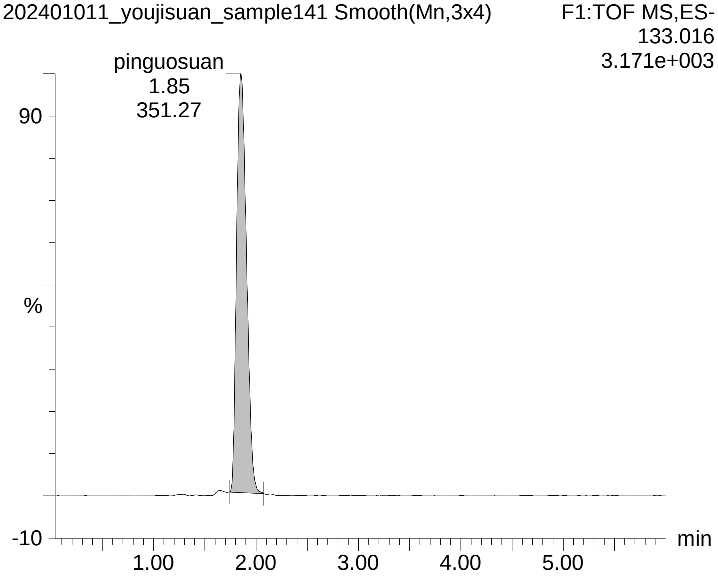

	#	Name	Sample Text	RT	Area	Std. Conc	Conc.
1	1	jiushisuan		1.49	2535.342		952.068769
2	2	ningmensuan		3.04	206.738		29.017594
3	3	pinguosuan		1.85	351.270		178.073144

Name: 202401011_youjisuan_sample142, Date: 11-Oct-2024, Time: 17:35:30, ID: , Description:

jiushisuan

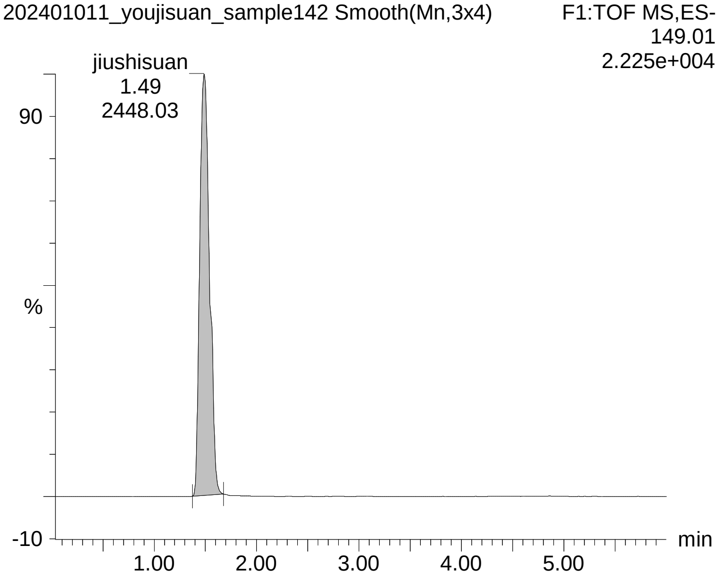

ningmensuan

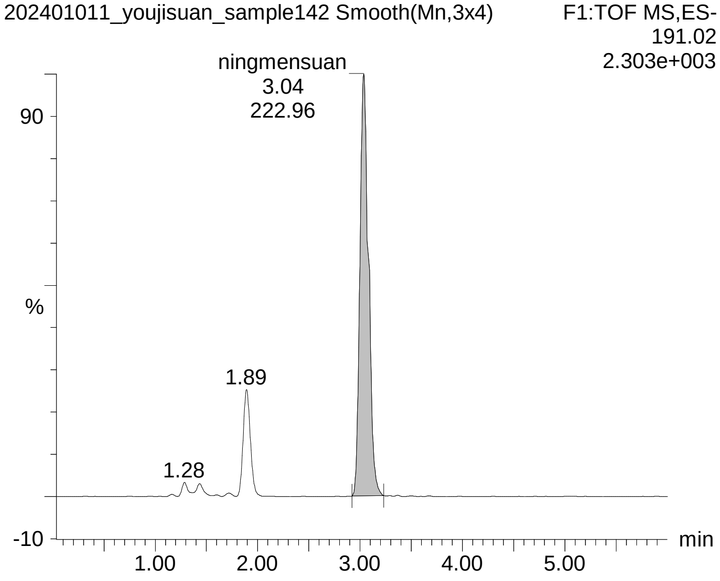

pinguosuan

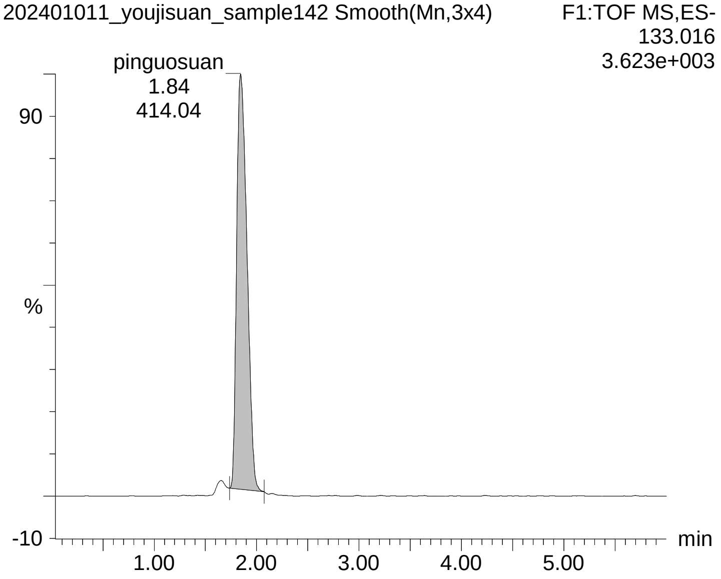

	#	Name	Sample Text	RT	Area	Std. Conc	Conc.
1	1	jiushisuan		1.49	2448.034		901.405849
2	2	ningmensuan		3.04	222.963		31.382235
3	3	pinguosuan		1.84	414.043		425.708947

project_wangzhonghua_BeiMu
Dataset: Untitled
Last Altered: Saturday, October 12, 2024 07:50:19 China Standard Time
Printed: Saturday, October 12, 2024 07:50:41 China Standard Time

Name: 202401011_youjisuan_sample143, Date: 11-Oct-2024, Time: 17:42:30, ID: , Description:

jiushisuan

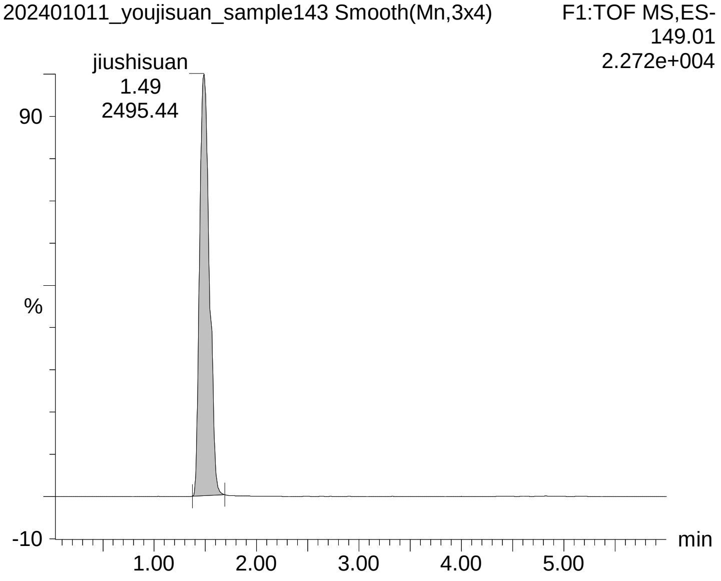

ningmensuan

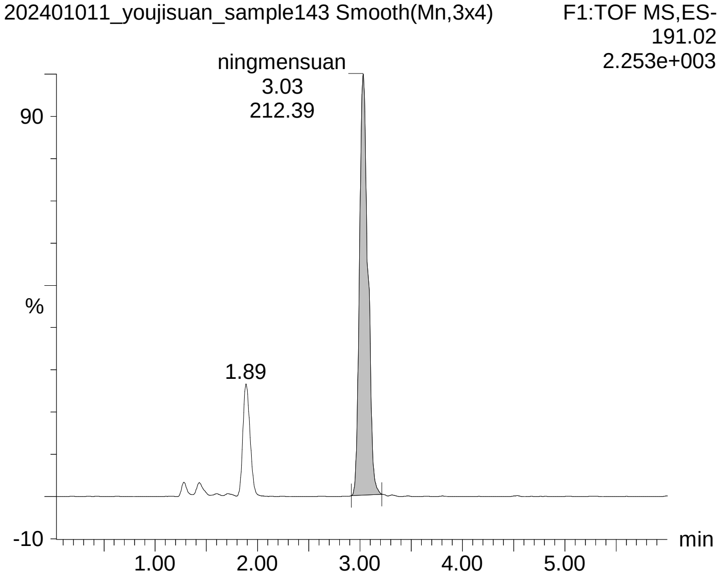

pinguosuan

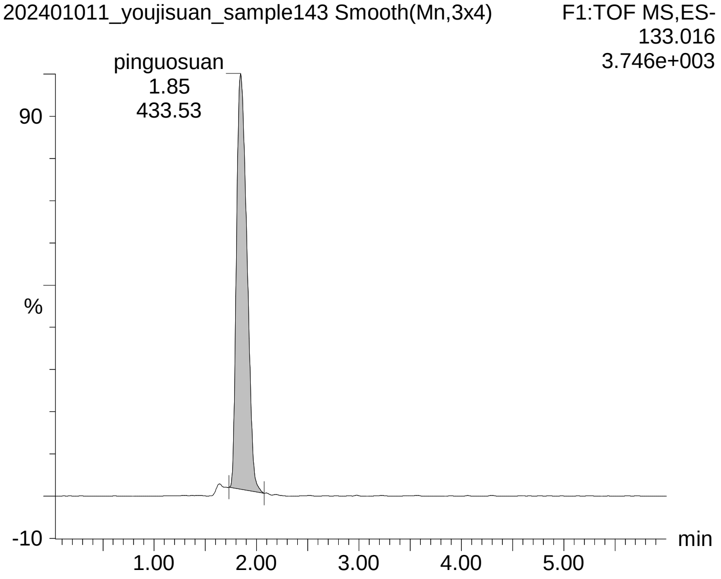

	#	Name	Sample Text	RT	Area	Std. Conc	Conc.
1	1	jiushisuan		1.49	2495.440		930.243564
2	2	ningmensuan		3.03	212.390		29.841320
3	3	pinguosuan		1.85	433.526		465.412980

project_wangzhonghua_BeiMu
Dataset: Untitled
Last Altered: Saturday, October 12, 2024 07:50:19 China Standard Time
Printed: Saturday, October 12, 2024 07:50:41 China Standard Time

Name: 202401011_youjisuan_sample144, Date: 11-Oct-2024, Time: 17:49:30, ID: , Description:

jiushisuan

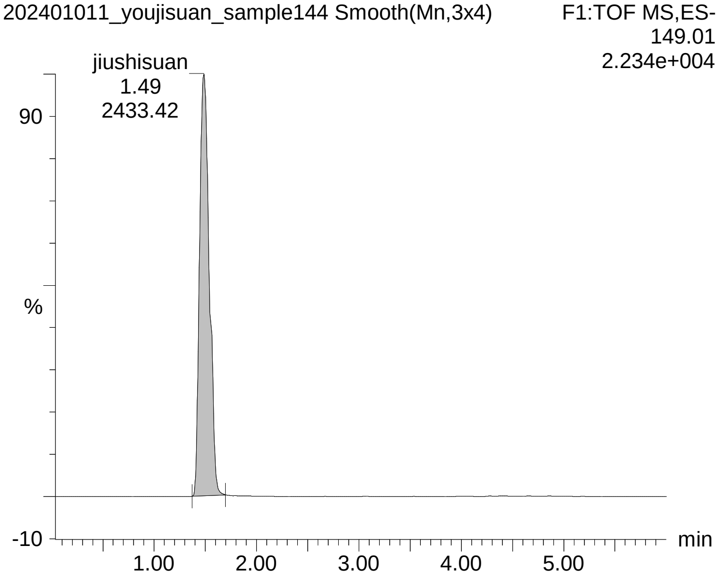

ningmensuan

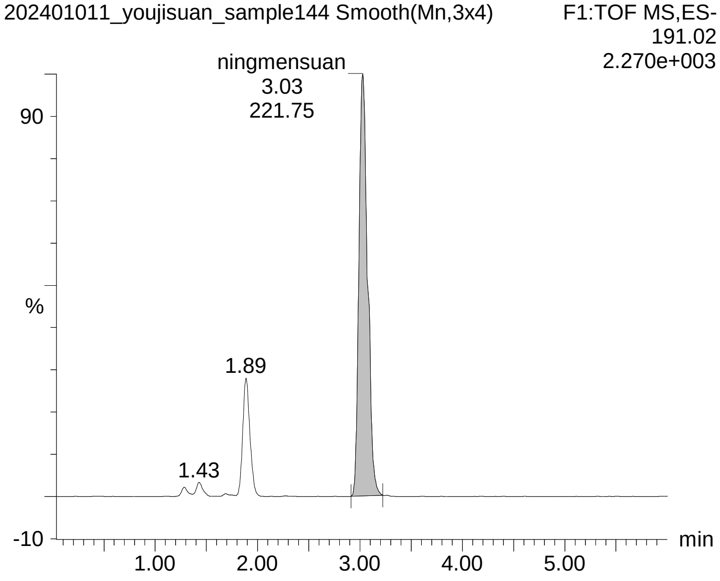

pinguosuan

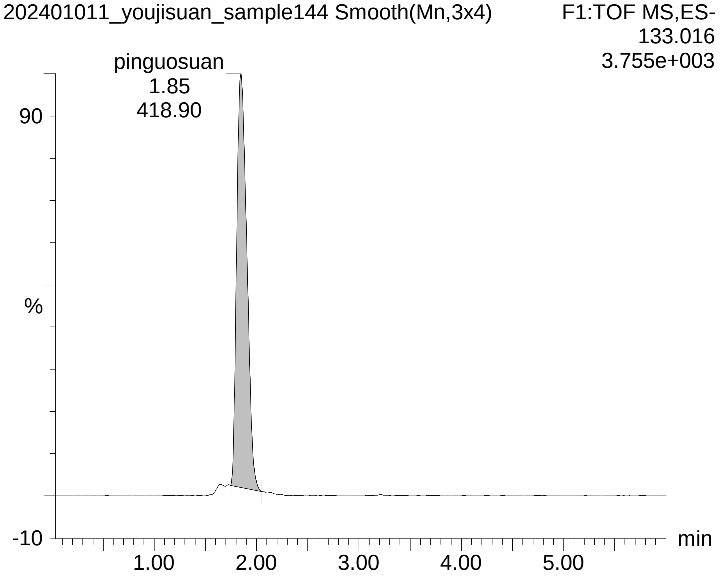

	#	Name	Sample Text	RT	Area	Std. Conc	Conc.
1	1	jiushisuan		1.49	2433.424		891.764429
2	2	ningmensuan		3.03	221.751		31.205598
3	3	pinguosuan		1.85	418.901		437.927343

project_wangzhonghua_BeiMu
Dataset: Untitled
Last Altered: Saturday, October 12, 2024 07:50:19 China Standard Time
Printed: Saturday, October 12, 2024 07:50:41 China Standard Time

Name: 202401011_youjisuan_sample145, Date: 11-Oct-2024, Time: 17:56:30, ID: , Description:

jiushisuan

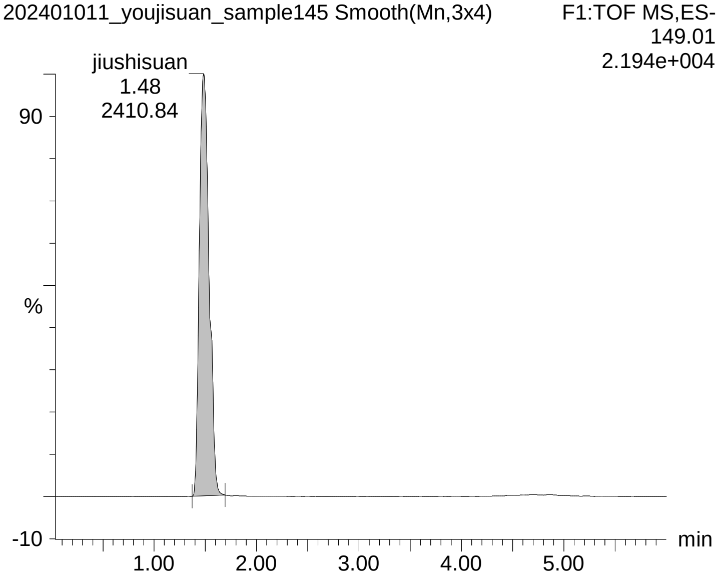

ningmensuan

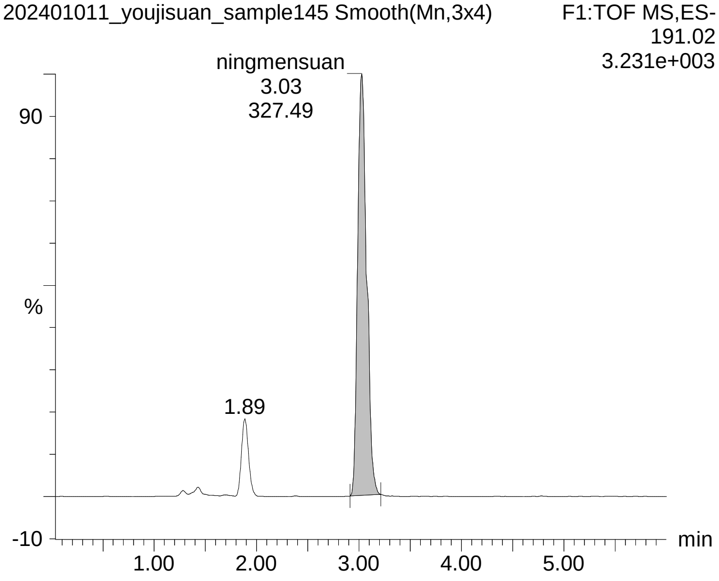

pinguosuan

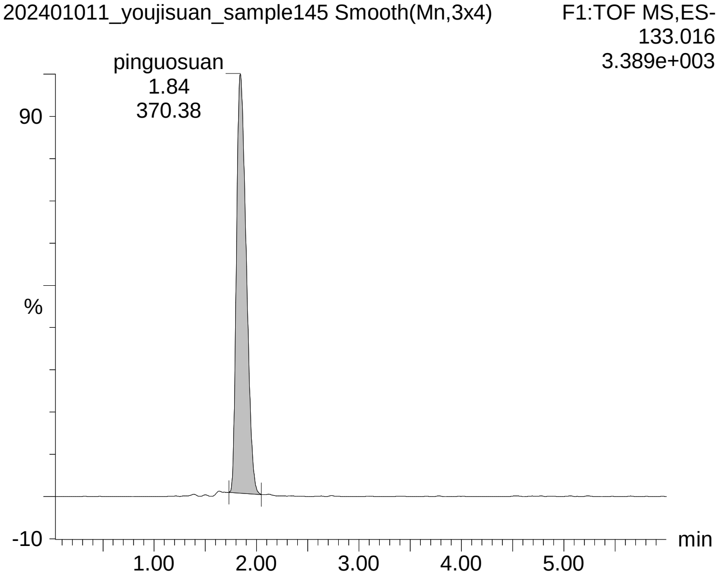

	#	Name	Sample Text	RT	Area	Std. Conc	Conc.
1	1	jiushisuan		1.48	2410.839		876.052358
2	2	ningmensuan		3.03	327.493		46.616501
3	3	pinguosuan		1.84	370.382		208.726743

Name: 202401011_youjisuan_sample146, Date: 11-Oct-2024, Time: 18:03:30, ID: , Description:

jiushisuan

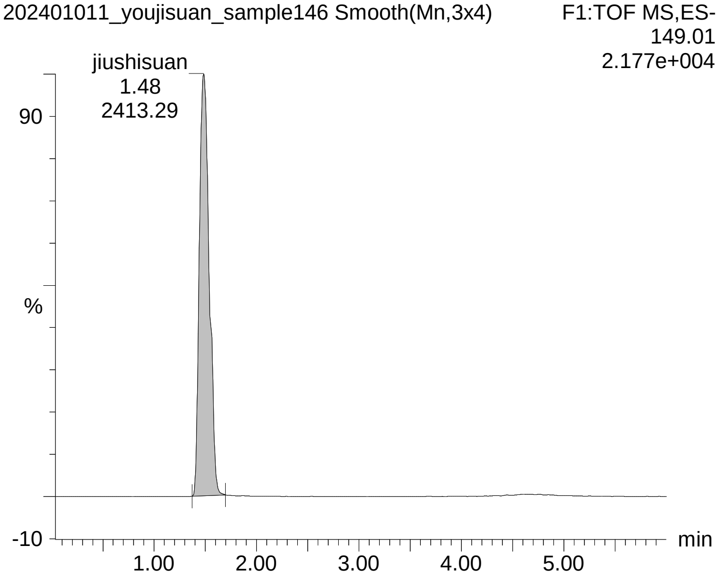

ningmensuan

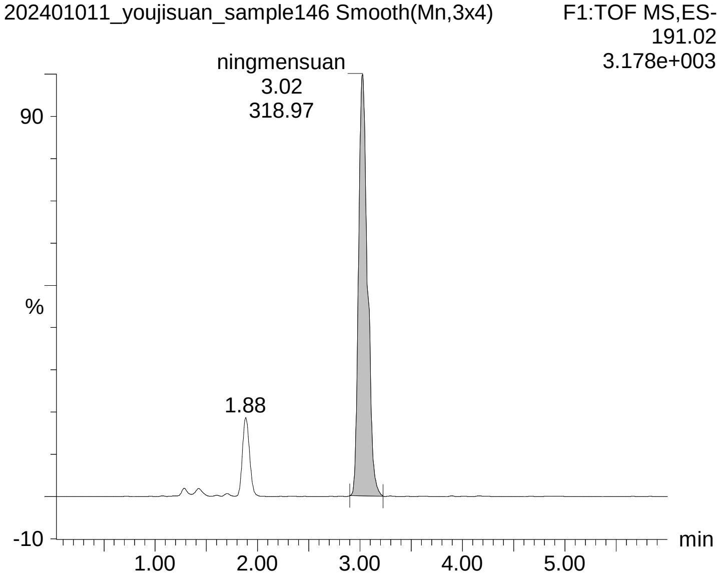

pinguosuan

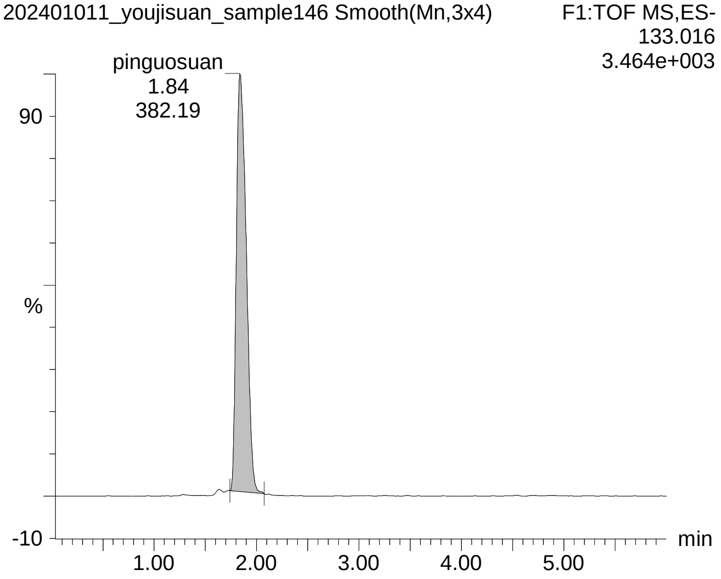

	#	Name	Sample Text	RT	Area	Std. Conc	Conc.
1	1	jiushisuan		1.48	2413.291		877.808586
2	2	ningmensuan		3.02	318.970		45.374354
3	3	pinguosuan		1.84	382.186		237.356013

Name: 202401011_youjisuan_sample147, Date: 11-Oct-2024, Time: 18:10:30, ID: , Description:

jiushisuan

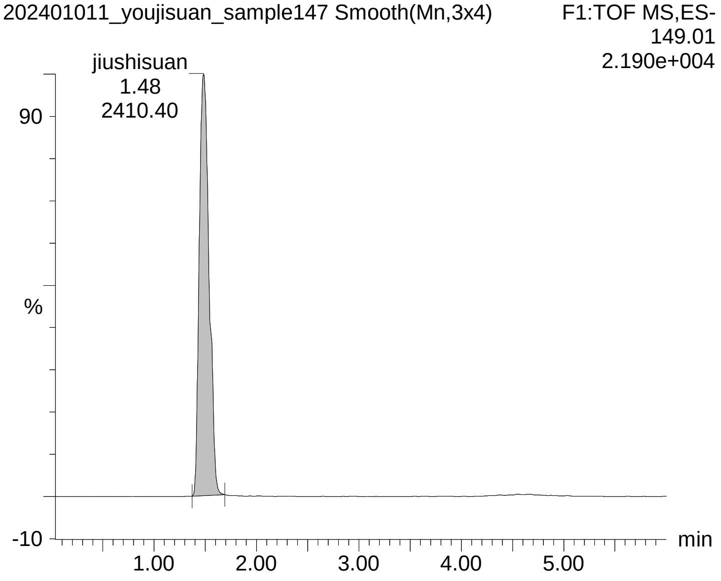

ningmensuan

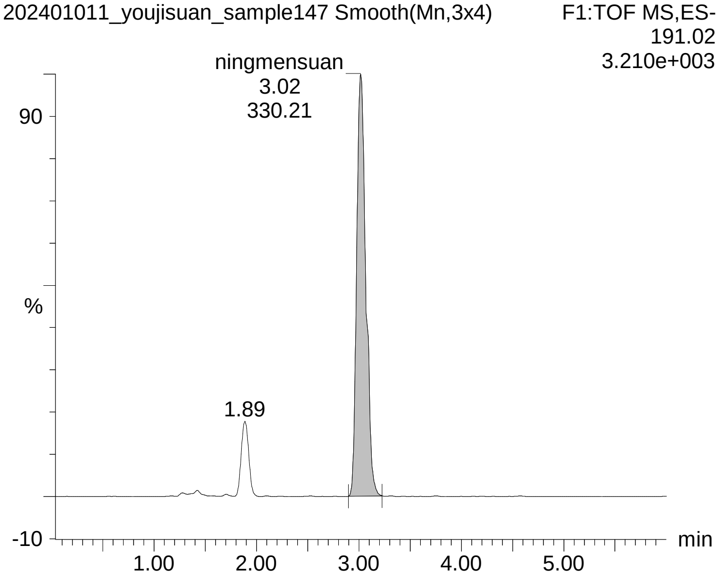

pinguosuan

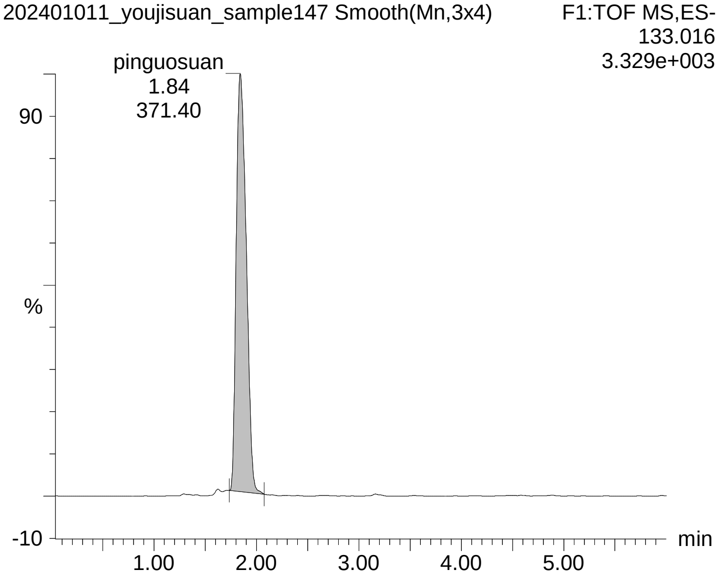

	#	Name	Sample Text	RT	Area	Std. Conc	Conc.
1	1	jiushisuan		1.48	2410.404		875.739458
2	2	ningmensuan		3.02	330.214		47.013061
3	3	pinguosuan		1.84	371.400		210.769104

Name: 202401011_youjisuan_sample148, Date: 11-Oct-2024, Time: 18:17:31, ID: , Description:

jiushisuan

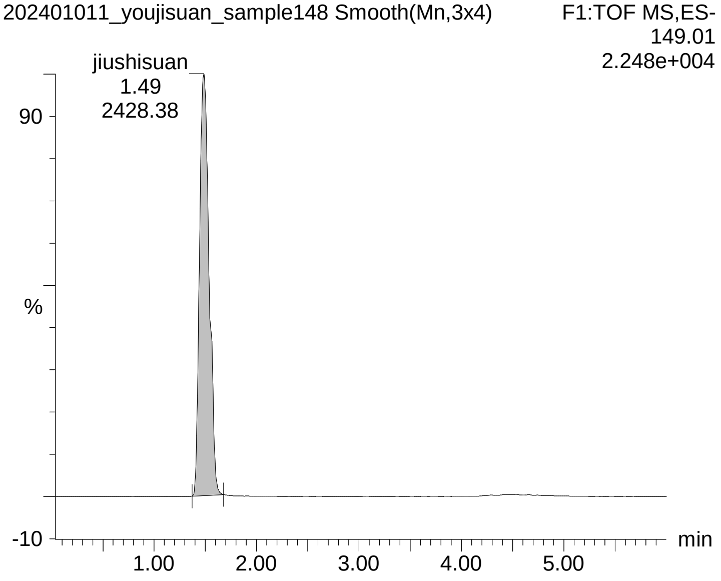

ningmensuan

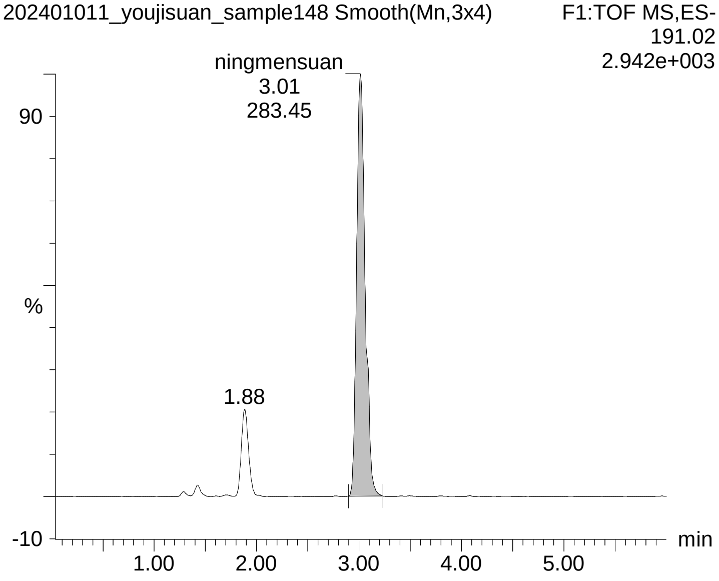

pinguosuan

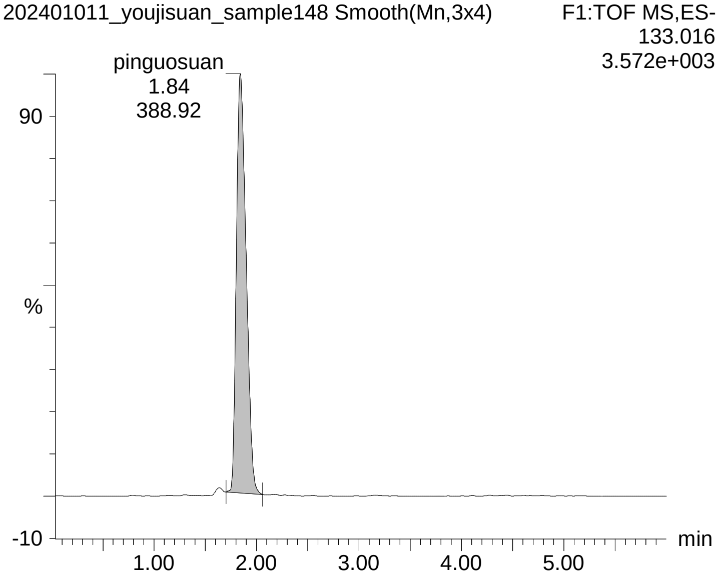

	#	Name	Sample Text	RT	Area	Std. Conc	Conc.
1	1	jiushisuan		1.49	2428.381		888.344070
2	2	ningmensuan		3.01	283.453		40.198085
3	3	pinguosuan		1.84	388.920		262.776226

Name: 202401011_youjisuan_sample149, Date: 11-Oct-2024, Time: 18:24:31, ID: , Description:

jiushisuan

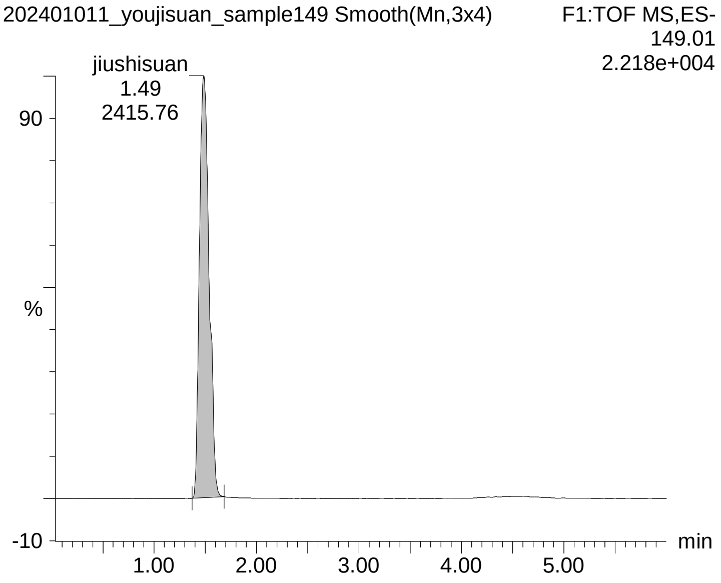

ningmensuan

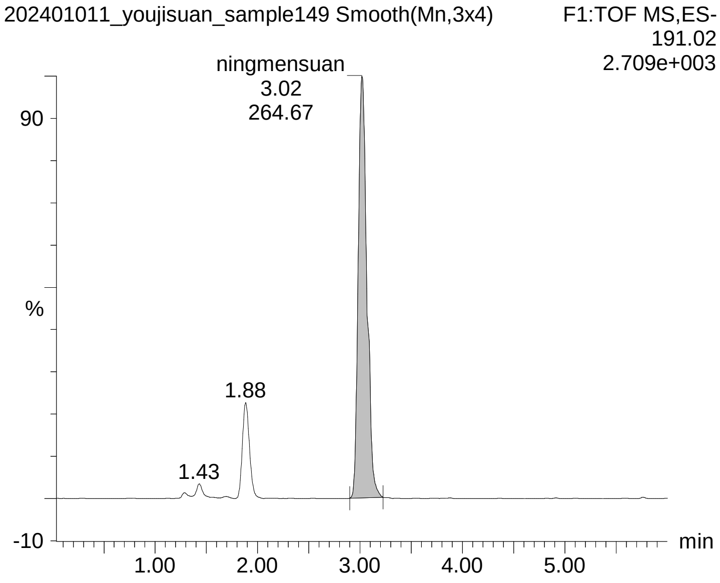

pinguosuan

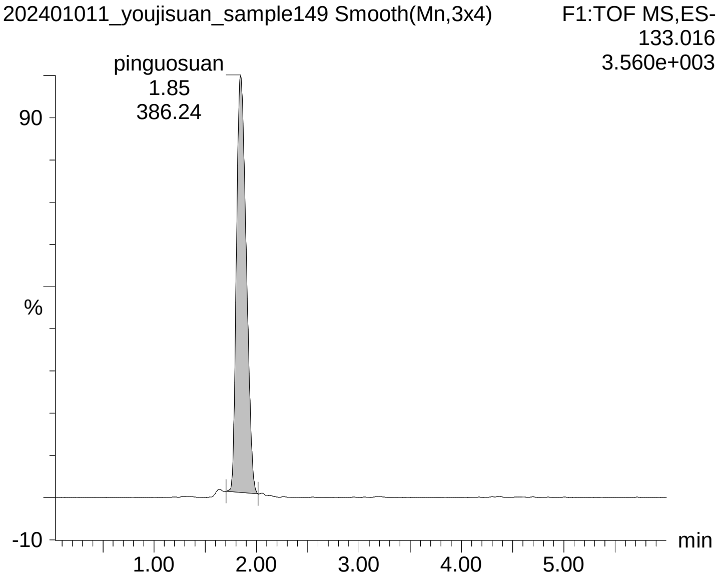

	#	Name	Sample Text	RT	Area	Std. Conc	Conc.
1	1	jiushisuan		1.49	2415.759		879.563493
2	2	ningmensuan		3.02	264.665		37.459910
3	3	pinguosuan		1.85	386.237		251.253067

Name: 202401011_youjisuan_sample150, Date: 11-Oct-2024, Time: 18:31:31, ID: , Description:

jiushisuan

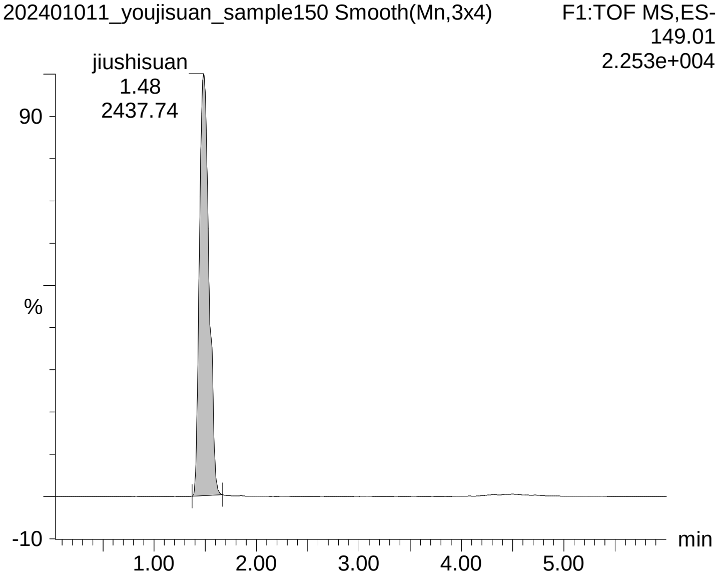

ningmensuan

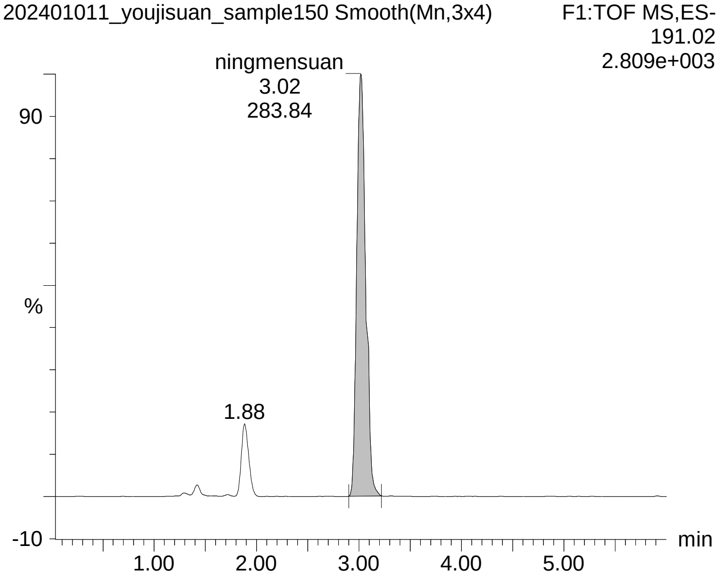

pinguosuan

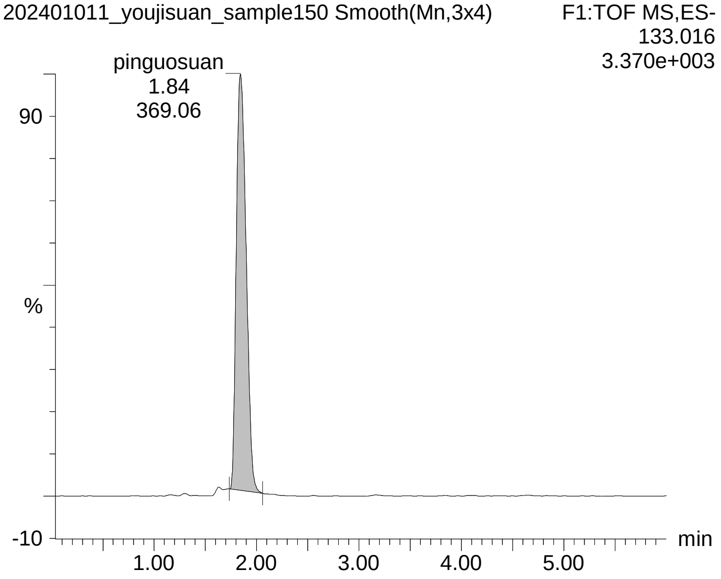

	#	Name	Sample Text	RT	Area	Std. Conc	Conc.
1	1	jiushisuan		1.48	2437.741		894.654048
2	2	ningmensuan		3.02	283.844		40.255069
3	3	pinguosuan		1.84	369.061		206.160653

project_wangzhonghua_BeiMu
Dataset: Untitled
Last Altered: Saturday, October 12, 2024 07:50:19 China Standard Time
Printed: Saturday, October 12, 2024 07:50:41 China Standard Time

Name: 202401011_youjisuan_sample151, Date: 11-Oct-2024, Time: 18:38:31, ID: , Description:

jiushisuan

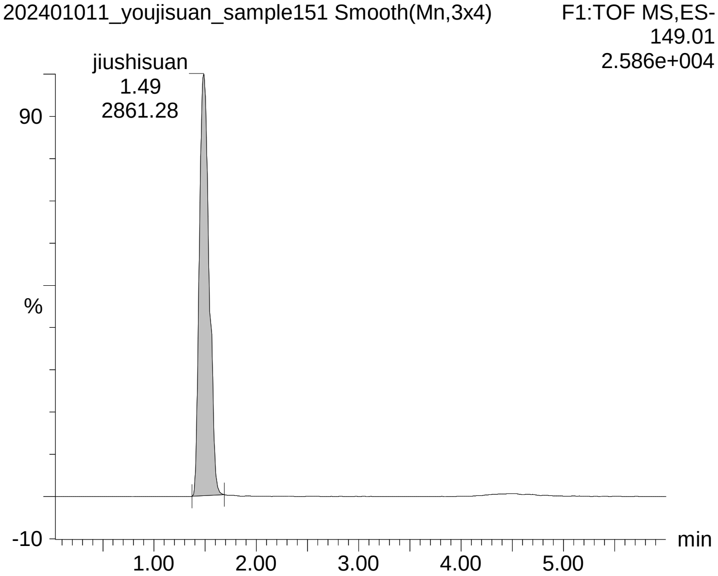

ningmensuan

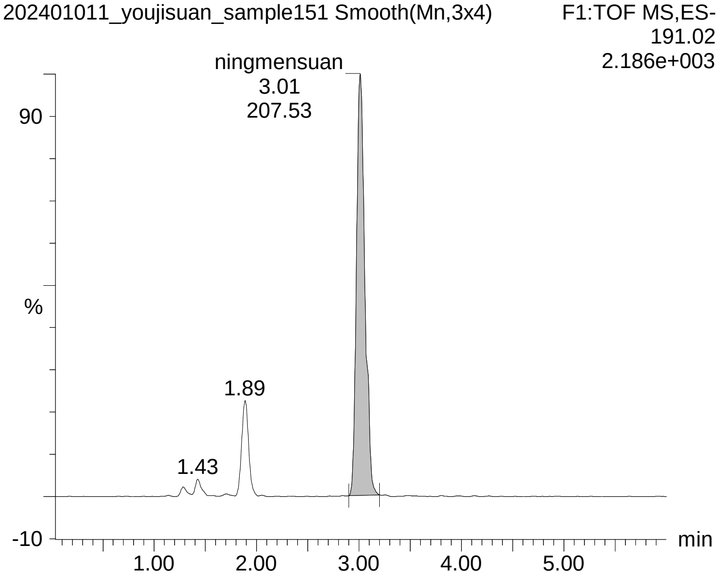

pinguosuan

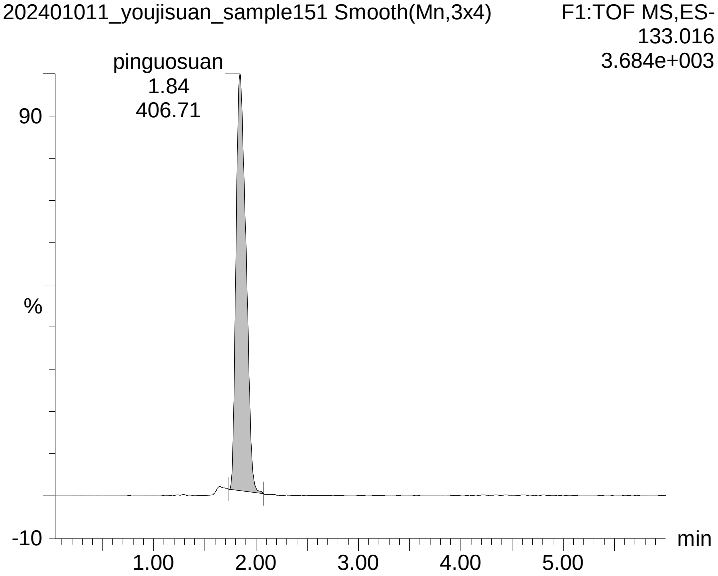

	#	Name	Sample Text	RT	Area	Std. Conc	Conc.
1	1	jiushisuan		1.49	2861.276		1081.694850
2	2	ningmensuan		3.01	207.530		29.133020
3	3	pinguosuan		1.84	406.714		400.931397

Name: 202401011_youjisuan_sample152, Date: 11-Oct-2024, Time: 18:45:31, ID: , Description:

jiushisuan

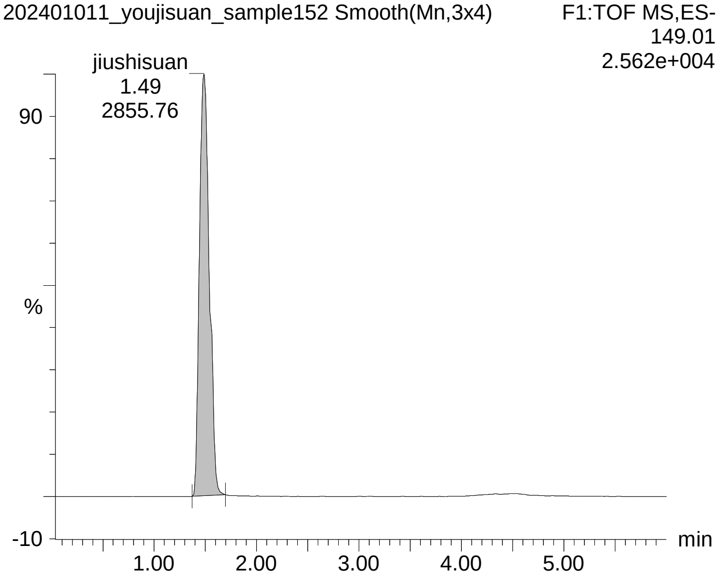

ningmensuan

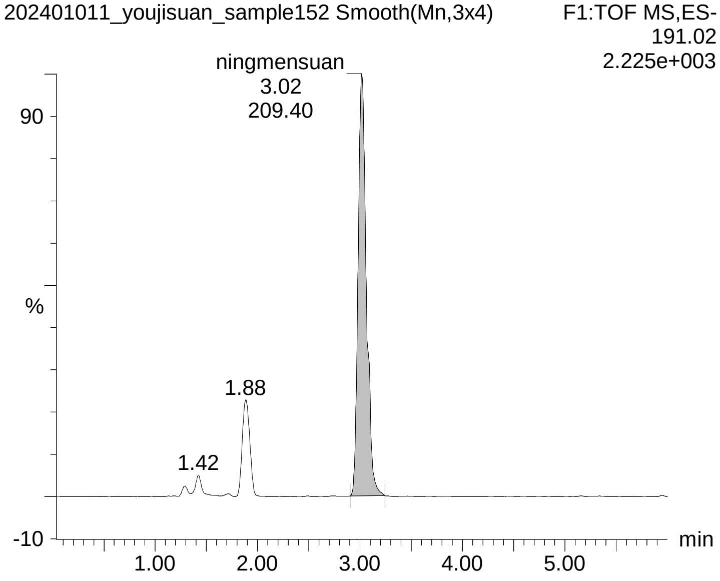

pinguosuan

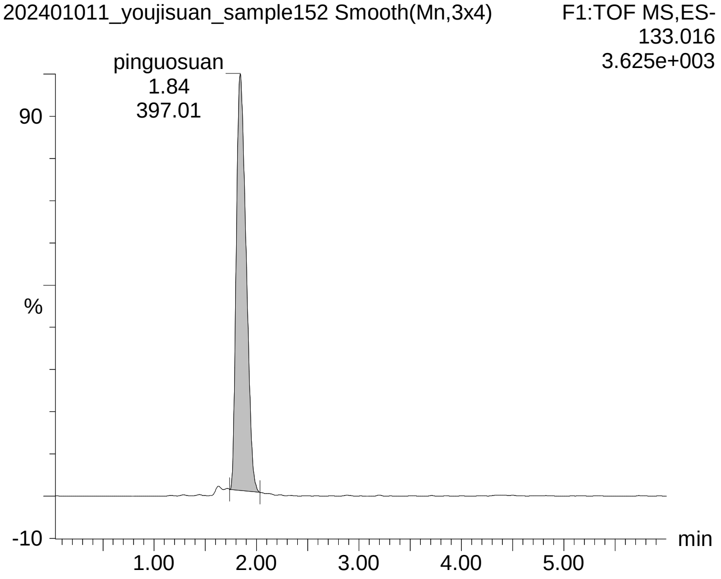

	#	Name	Sample Text	RT	Area	Std. Conc	Conc.
1	1	jiushisuan		1.49	2855.762		1079.965542
2	2	ningmensuan		3.02	209.404		29.406138
3	3	pinguosuan		1.84	397.008		327.758535

project_wangzhonghua_BeiMu
Dataset: Untitled
Last Altered: Saturday, October 12, 2024 07:50:19 China Standard Time
Printed: Saturday, October 12, 2024 07:50:41 China Standard Time

Name: 202401011_youjisuan_sample153, Date: 11-Oct-2024, Time: 18:52:32, ID: , Description:

jiushisuan

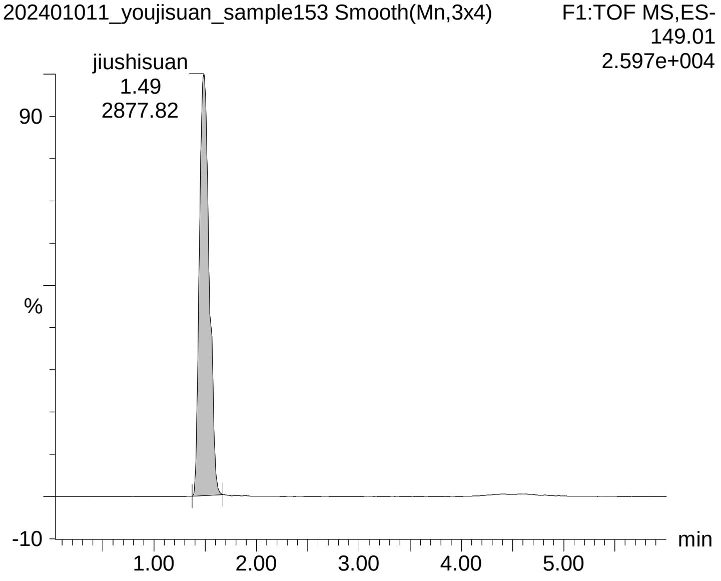

ningmensuan

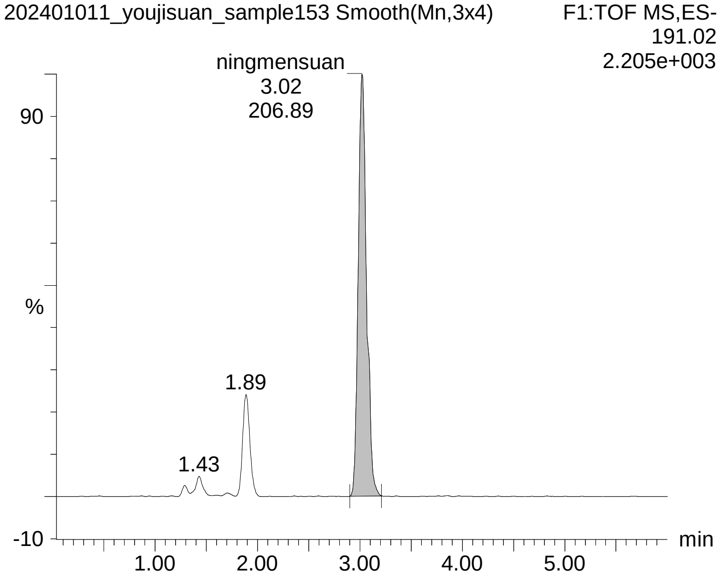

pinguosuan

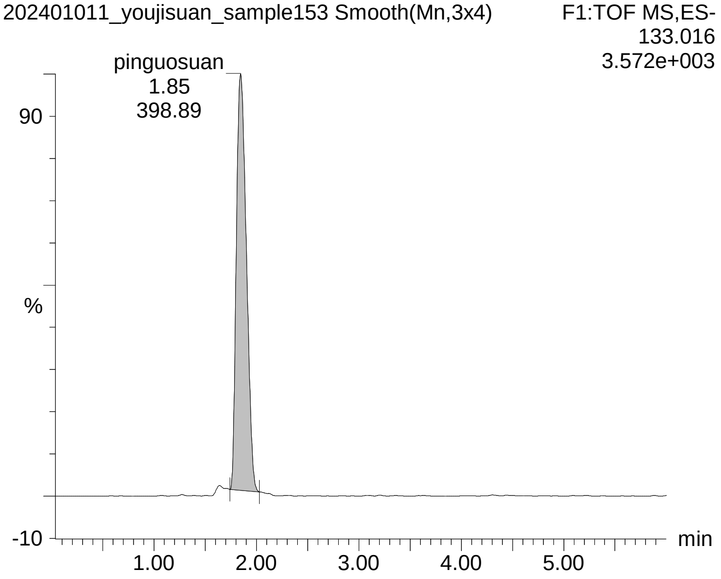

	#	Name	Sample Text	RT	Area	Std. Conc	Conc.
1	1	jiushisuan		1.49	2877.820		1086.818029
2	2	ningmensuan		3.02	206.887		29.039309
3	3	pinguosuan		1.85	398.890		349.715723

Name: 202401011_youjisuan_sample154, Date: 11-Oct-2024, Time: 18:59:27, ID: , Description:

jiushisuan

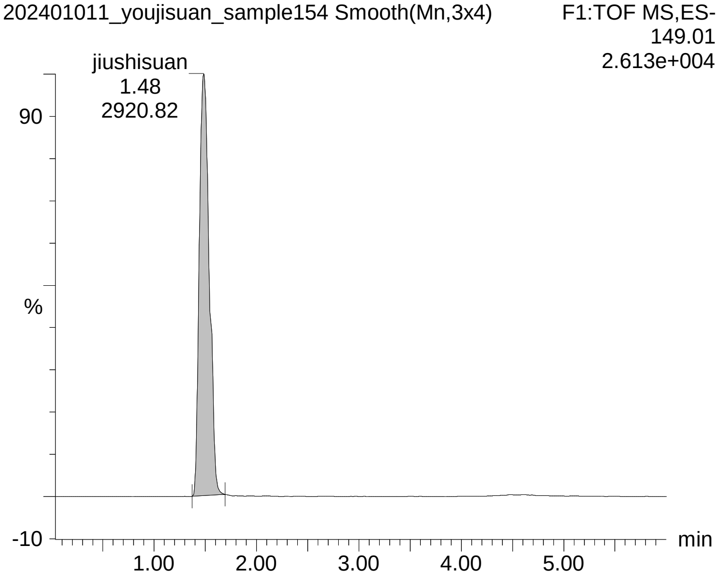

ningmensuan

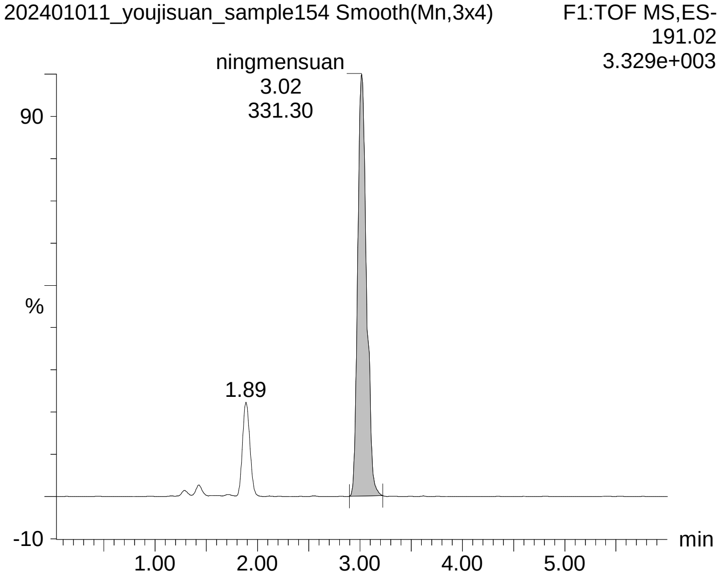

pinguosuan

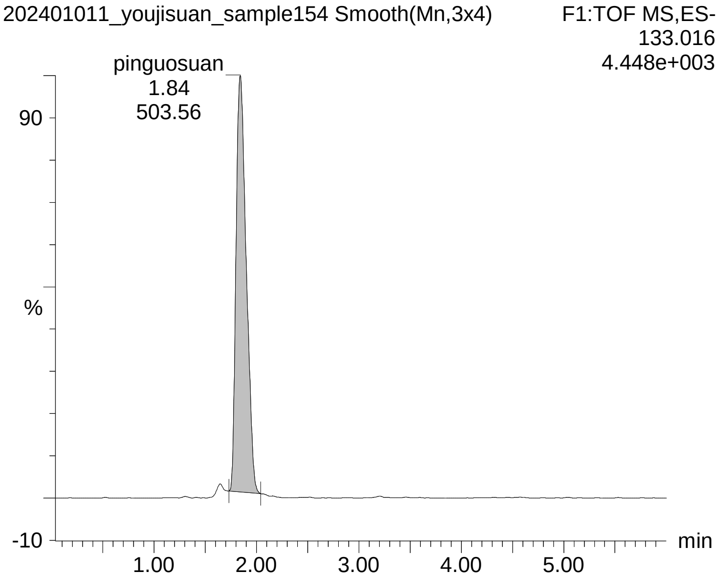

	#	Name	Sample Text	RT	Area	Std. Conc	Conc.
1	1	jiushisuan		1.48	2920.817		1099.699477
2	2	ningmensuan		3.02	331.295		47.170607
3	3	pinguosuan		1.84	503.557		537.096711

Name: 202401011_youjisuan_sample155, Date: 11-Oct-2024, Time: 19:06:25, ID: , Description:

jiushisuan

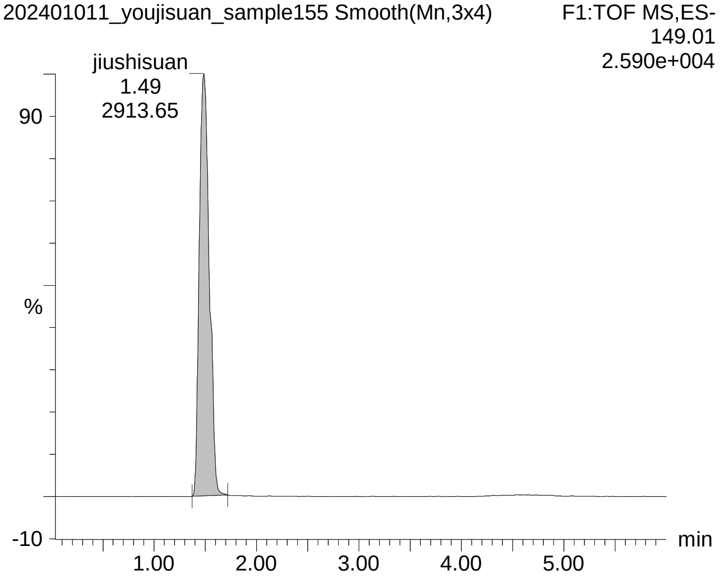

ningmensuan

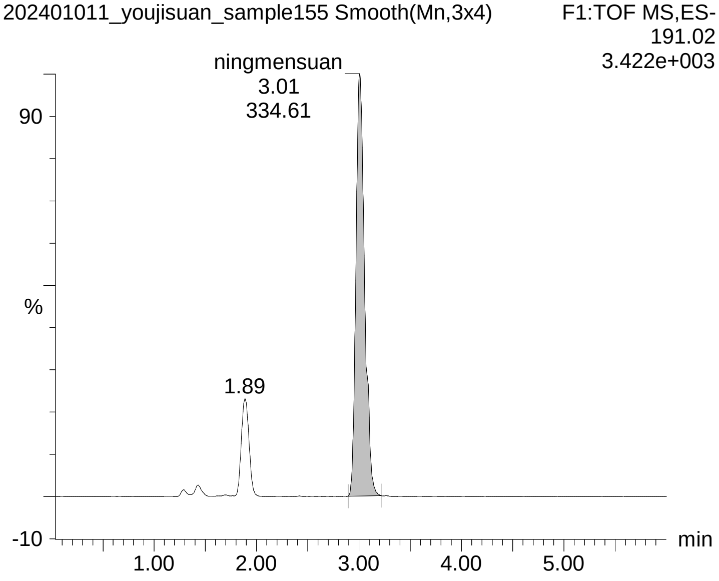

pinguosuan

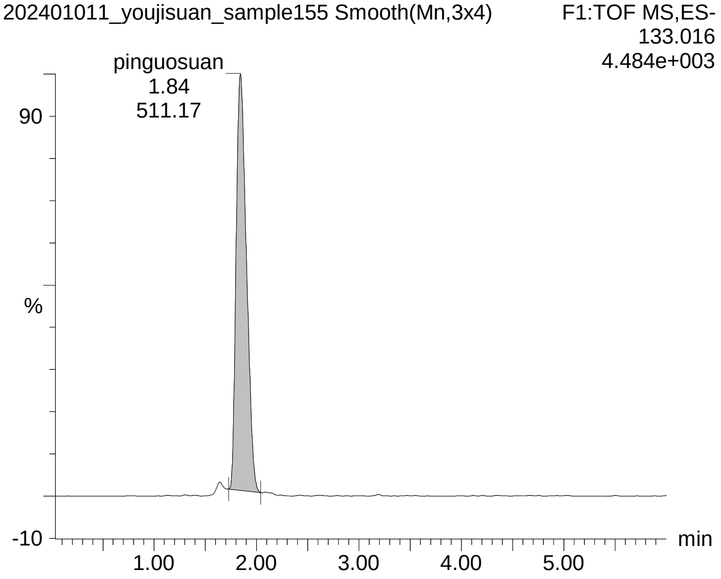

	#	Name	Sample Text	RT	Area	Std. Conc	Conc.
1	1	jiushisuan		1.49	2913.646		1097.592697
2	2	ningmensuan		3.01	334.609		47.653591
3	3	pinguosuan		1.84	511.168		542.523973

Name: 202401011_youjisuan_sample156, Date: 11-Oct-2024, Time: 19:13:26, ID: , Description:

jiushisuan

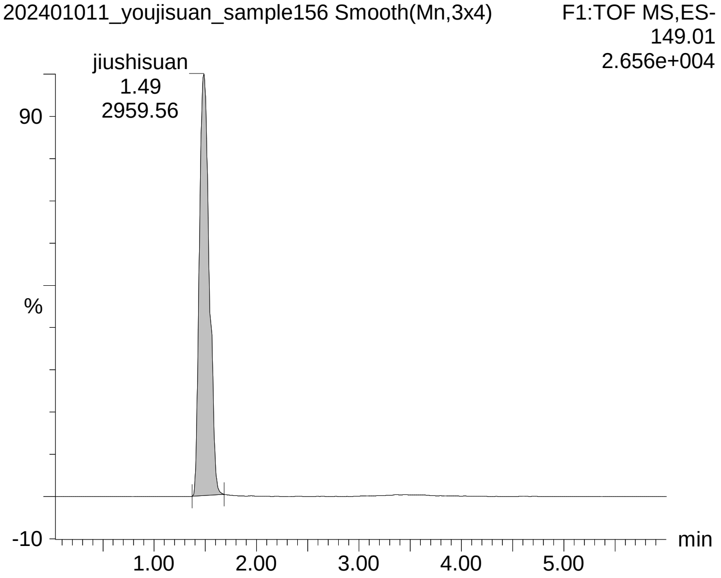

ningmensuan

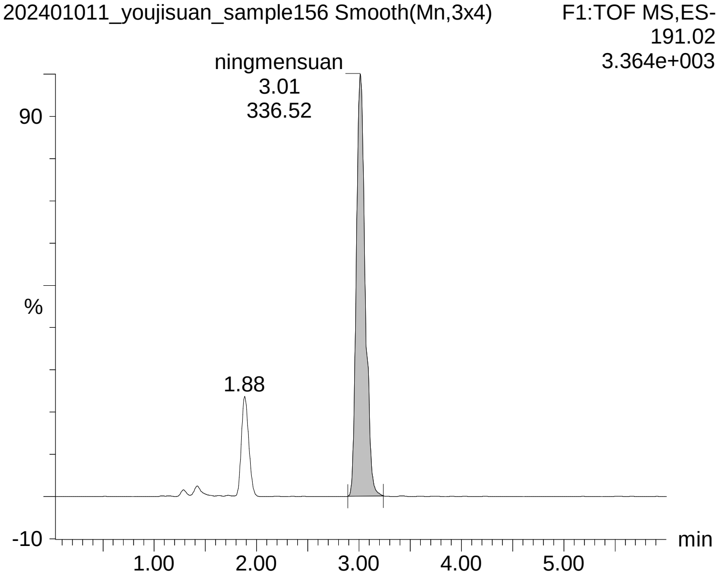

pinguosuan

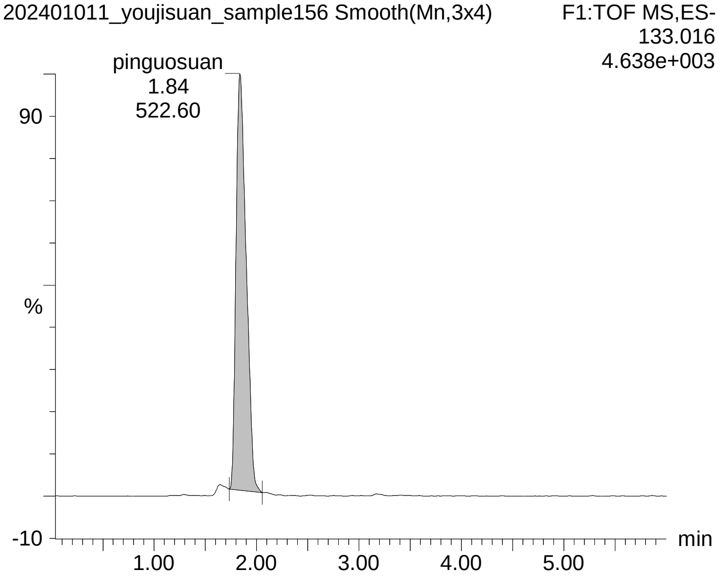

	#	Name	Sample Text	RT	Area	Std. Conc	Conc.
1	1	jiushisuan		1.49	2959.559		1110.812559
2	2	ningmensuan		3.01	336.518		47.931810
3	3	pinguosuan		1.84	522.595		550.215257

Name: 202401011_youjisuan_sample157, Date: 11-Oct-2024, Time: 19:21:23, ID: , Description:

jiushisuan

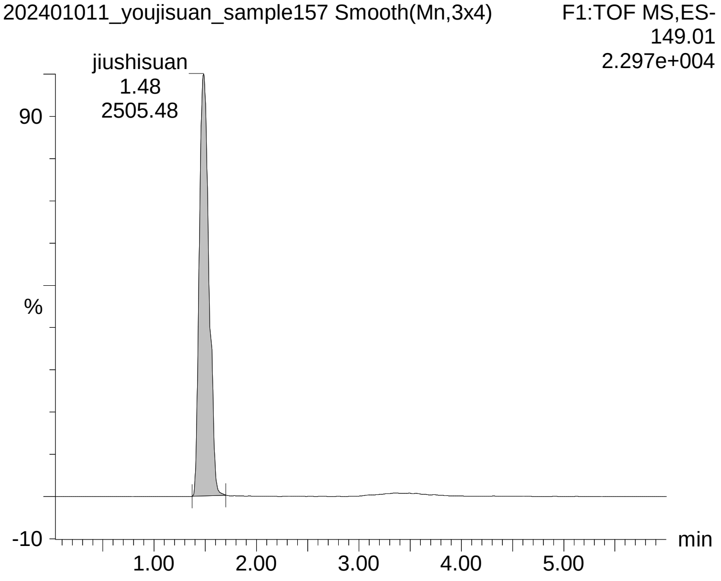

ningmensuan

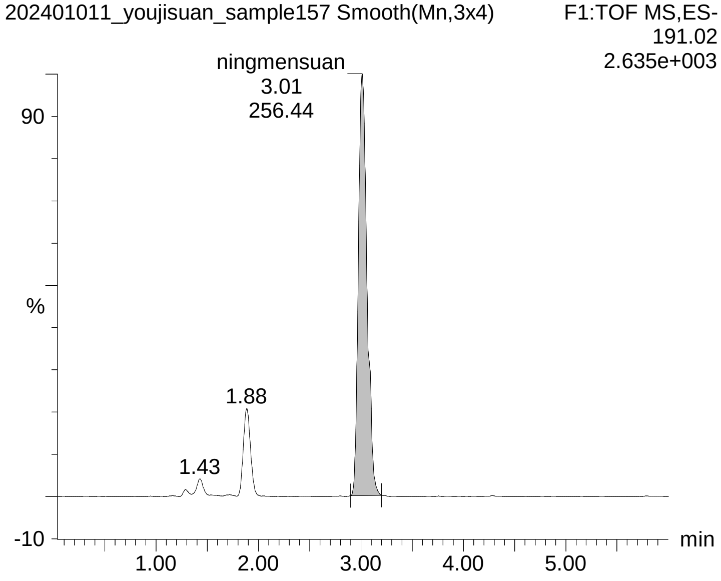

pinguosuan

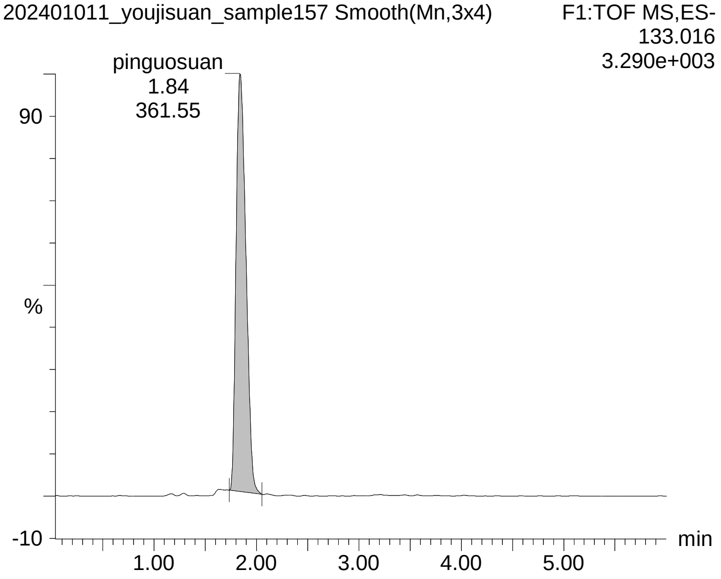

	#	Name	Sample Text	RT	Area	Std. Conc	Conc.
1	1	jiushisuan		1.48	2505.481		935.926859
2	2	ningmensuan		3.01	256.443		36.261631
3	3	pinguosuan		1.84	361.550		193.055222

Name: 202401011_youjisuan_sample158, Date: 11-Oct-2024, Time: 19:28:23, ID: , Description:

jiushisuan

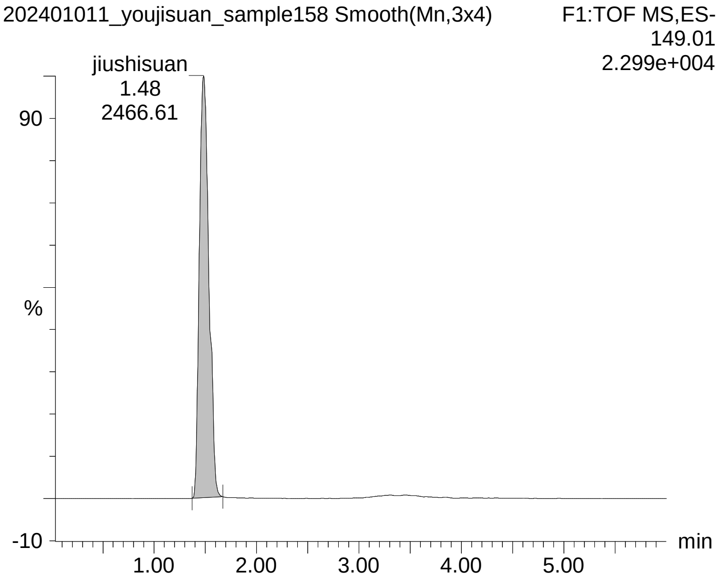

ningmensuan

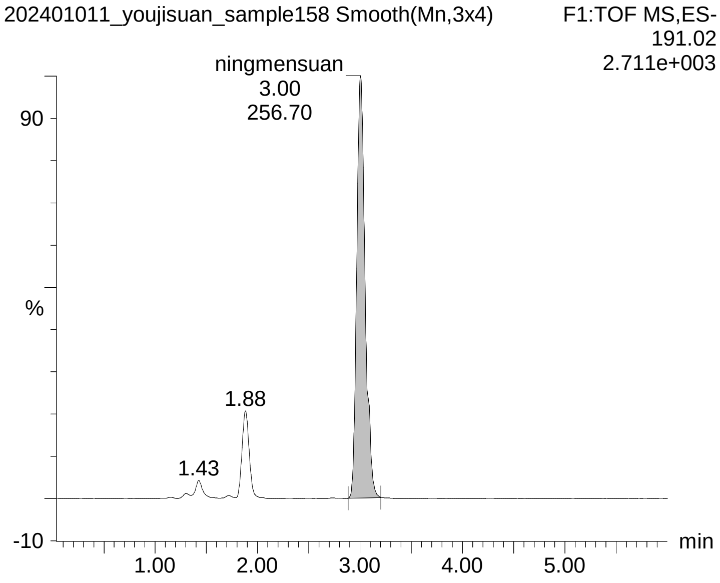

pinguosuan

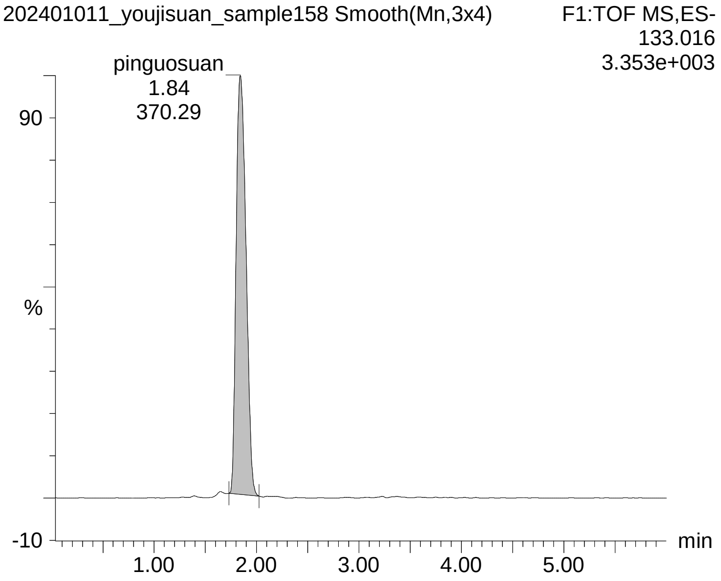

	#	Name	Sample Text	RT	Area	Std. Conc	Conc.
1	1	jiushisuan		1.48	2466.609		913.125879
2	2	ningmensuan		3.00	256.700		36.299086
3	3	pinguosuan		1.84	370.293		208.550944

Name: 202401011_youjisuan_sample159, Date: 11-Oct-2024, Time: 19:35:23, ID: , Description:

jiushisuan

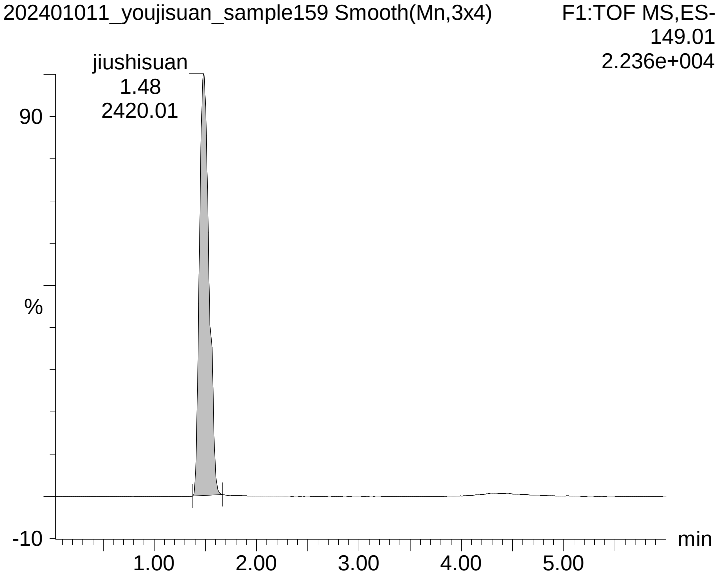

ningmensuan

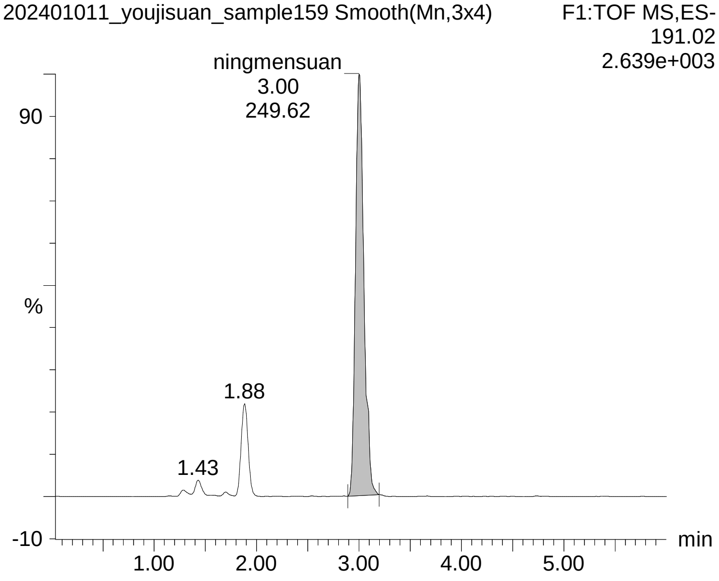

pinguosuan

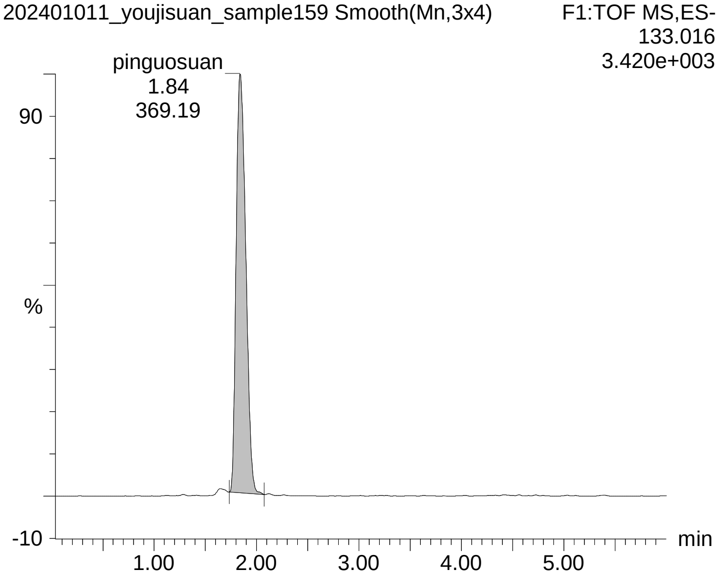

	#	Name	Sample Text	RT	Area	Std. Conc	Conc.
1	1	jiushisuan		1.48	2420.009		882.555941
2	2	ningmensuan		3.00	249.621		35.267388
3	3	pinguosuan		1.84	369.188		206.403421

project_wangzhonghua_BeiMu
Dataset: Untitled
Last Altered: Saturday, October 12, 2024 07:50:19 China Standard Time
Printed: Saturday, October 12, 2024 07:50:41 China Standard Time

Name: 202401011_youjisuan_sample160, Date: 11-Oct-2024, Time: 19:42:24, ID: , Description:

jiushisuan

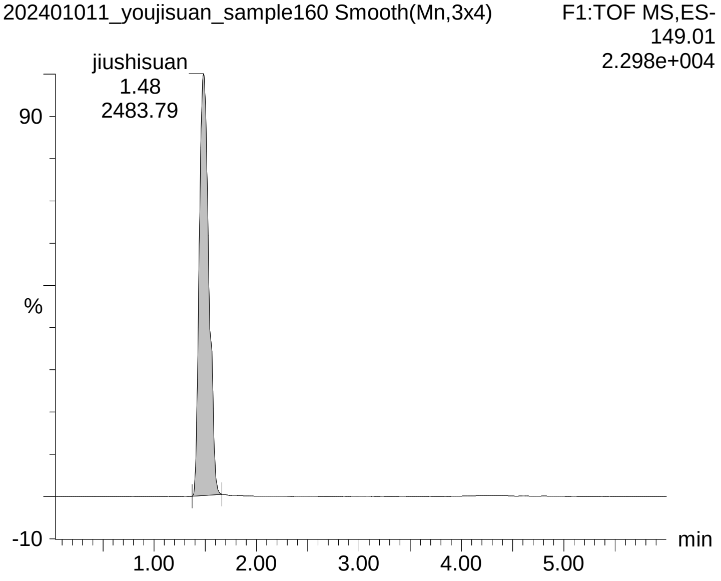

ningmensuan

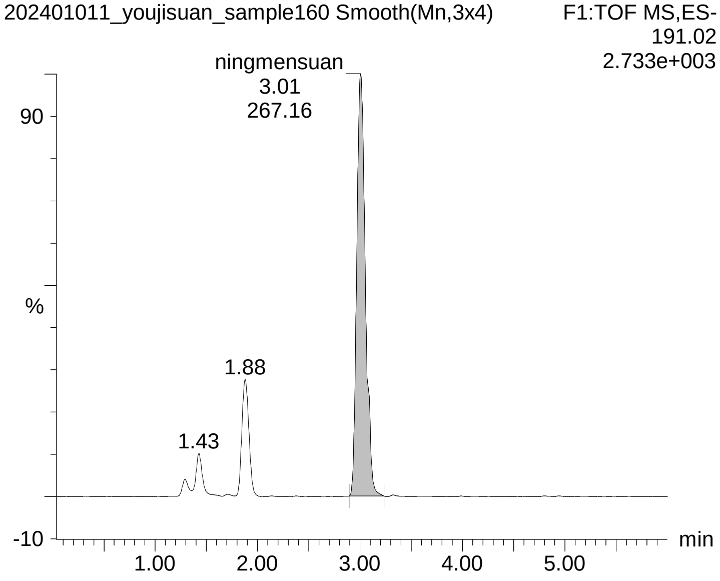

pinguosuan

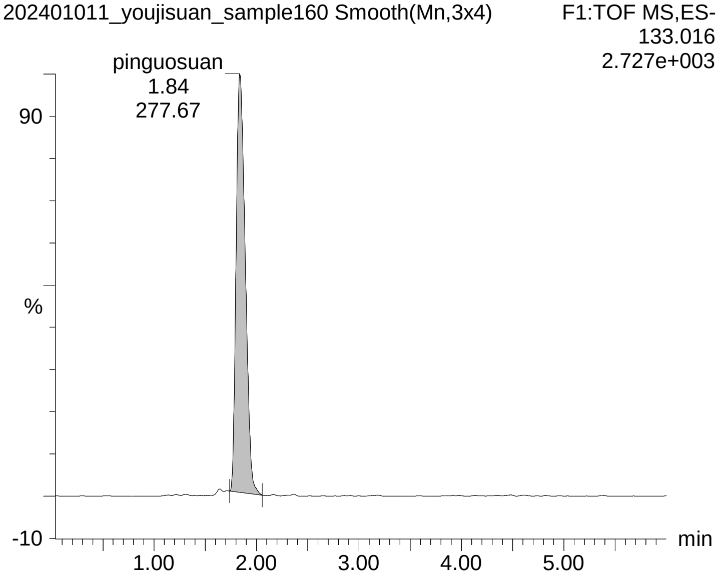

	#	Name	Sample Text	RT	Area	Std. Conc	Conc.
1	1	jiushisuan		1.48	2483.787		923.472779
2	2	ningmensuan		3.01	267.162		37.823824
3	3	pinguosuan		1.84	277.671		110.821507

project_wangzhonghua_BeiMu
Dataset: Untitled
Last Altered: Saturday, October 12, 2024 07:50:19 China Standard Time
Printed: Saturday, October 12, 2024 07:50:41 China Standard Time

Name: 202401011_youjisuan_sample161, Date: 11-Oct-2024, Time: 19:49:24, ID: , Description:

jiushisuan

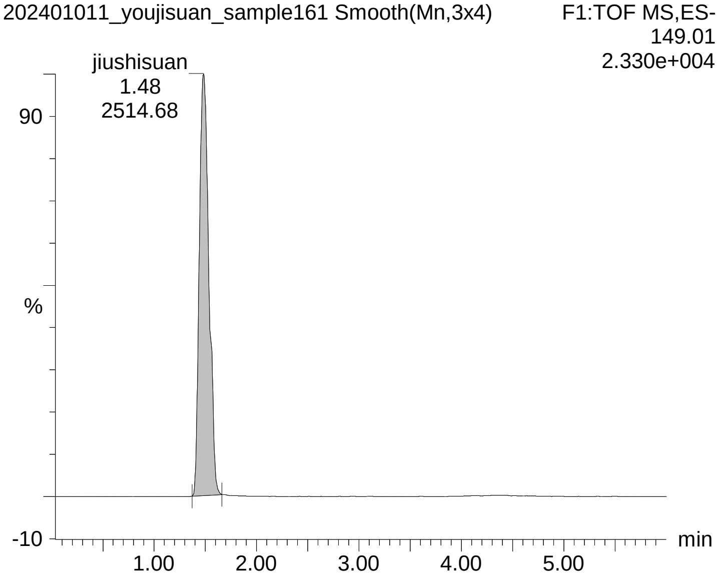

ningmensuan

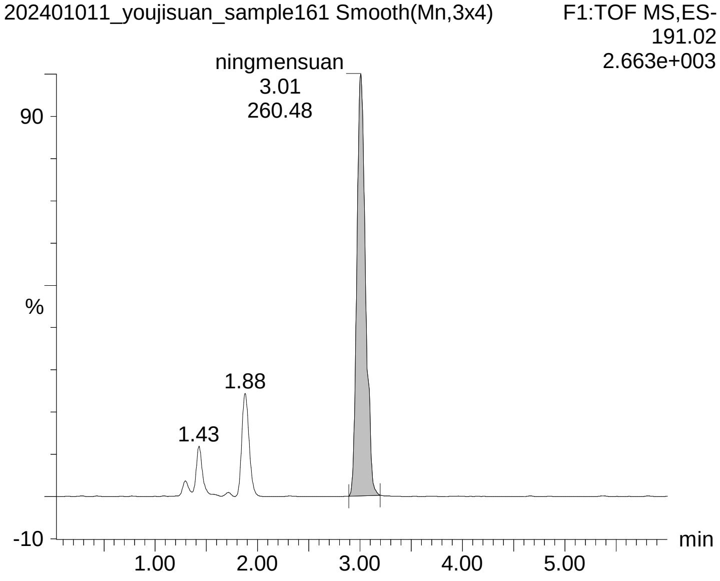

pinguosuan

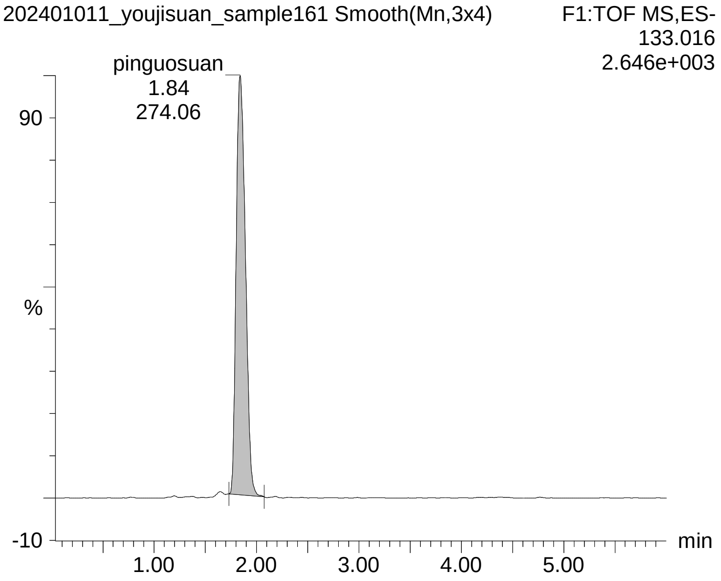

	#	Name	Sample Text	RT	Area	Std. Conc	Conc.
1	1	jiushisuan		1.48	2514.683		941.018663
2	2	ningmensuan		3.01	260.480		36.849986
3	3	pinguosuan		1.84	274.060		108.411498

project_wangzhonghua_BeiMu
Dataset: Untitled
Last Altered: Saturday, October 12, 2024 07:50:19 China Standard Time
Printed: Saturday, October 12, 2024 07:50:41 China Standard Time

Name: 202401011_youjisuan_sample162, Date: 11-Oct-2024, Time: 19:56:24, ID: , Description:

jiushisuan

202401011_youjisuan_sample162 Smooth(Mn,3x4) F1:TOF MS,ES- 149.01 2.366e+004

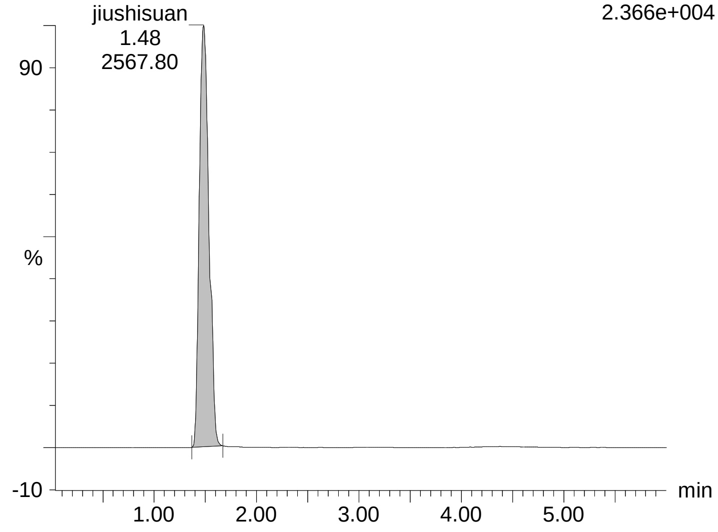

ningmensuan

202401011_youjisuan_sample162 Smooth(Mn,3x4) F1:TOF MS,ES- 191.02 2.716e+003

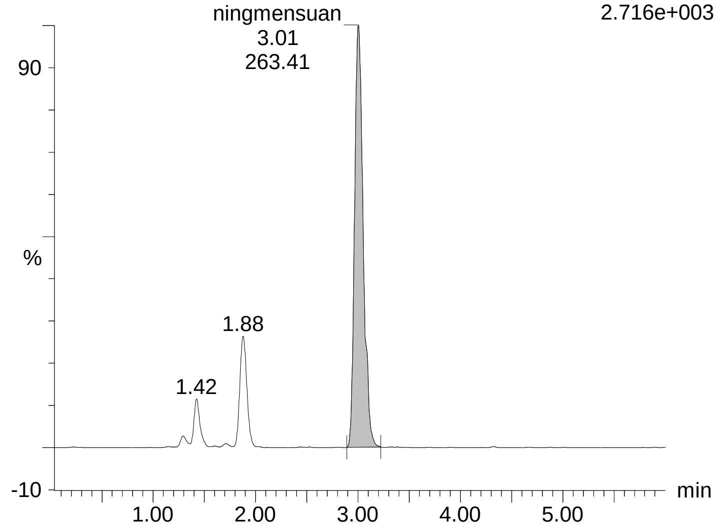

pinguosuan

202401011_youjisuan_sample162 Smooth(Mn,3x4) F1:TOF MS,ES- 133.016 2.587e+003

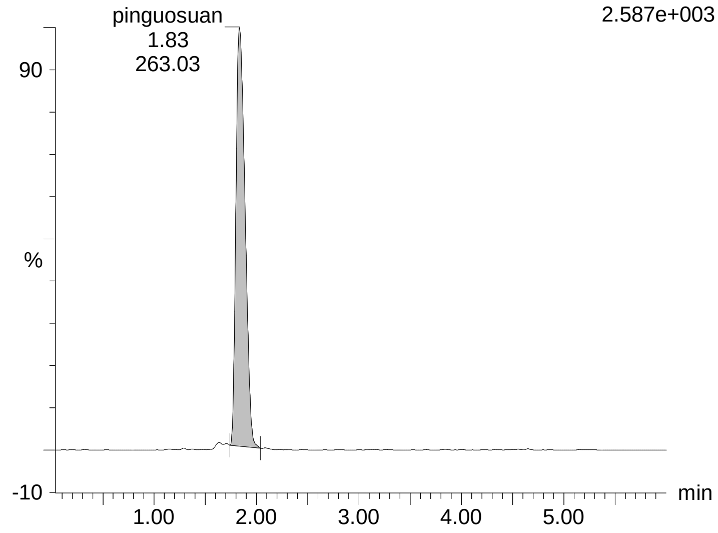

	#	Name	Sample Text	RT	Area	Std. Conc	Conc.
1	1	jiushisuan		1.48	2567.799		968.461183
2	2	ningmensuan		3.01	263.410		37.277006
3	3	pinguosuan		1.83	263.026		101.340620

project_wangzhonghua_BeiMu
Dataset: Untitled
Last Altered: Sunday, September 29, 2024 09:54:36 China Standard Time
Printed: Sunday, September 29, 2024 09:59:41 China Standard Time

Method: F:\data\Wu_yueyan.PRO\MethDB\20240918_ tang lei_ELSD .mdb 18 Sep 2024 14:15:05
Calibration: F:\data\zhanghuien.PRO\CurveDB\20240929_tanglei.cdb 29 Sep 2024 09:53:03

Compound name: guotang
Correlation coefficient: r = 0.990650, r^2 = 0.981387
Calibration curve: 1757.26 * x + -25179.6
Response type: External Std, Area
Curve type: Linear, Origin: Exclude, Weighting: Null, Axis trans: None

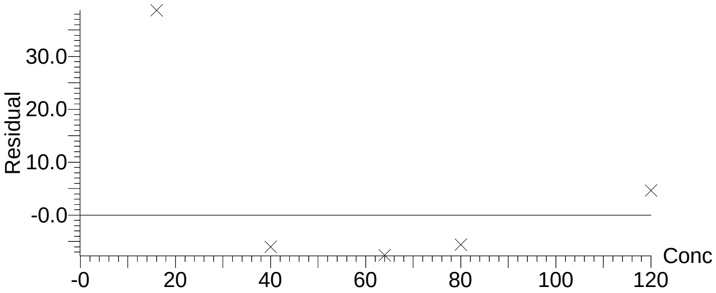

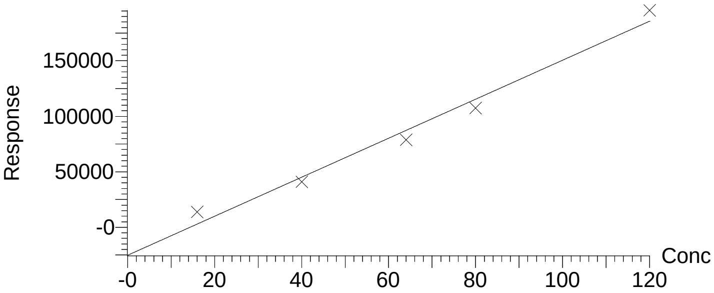

Compound name: putaotang
Correlation coefficient: r = 0.991188, r^2 = 0.982454
Calibration curve: 1708.63 * x + -24901.8
Response type: External Std, Area
Curve type: Linear, Origin: Exclude, Weighting: Null, Axis trans: None

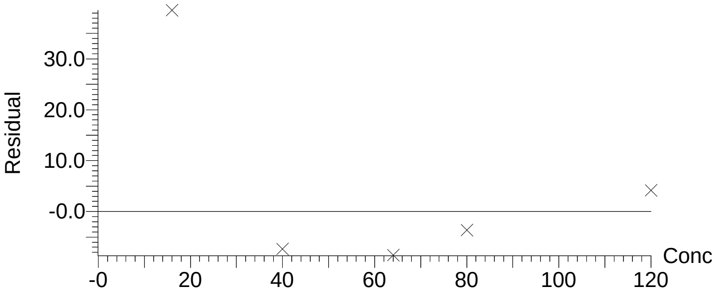

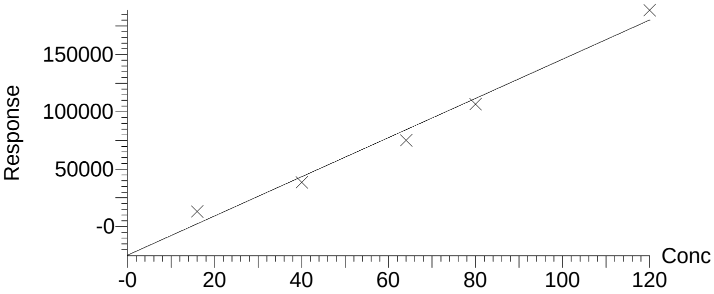

Compound name: zhetang
Correlation coefficient: r = 0.994858, r^2 = 0.989743
Calibration curve: 1866.58 * x + -37450
Response type: External Std, Area
Curve type: Linear, Origin: Exclude, Weighting: Null, Axis trans: None

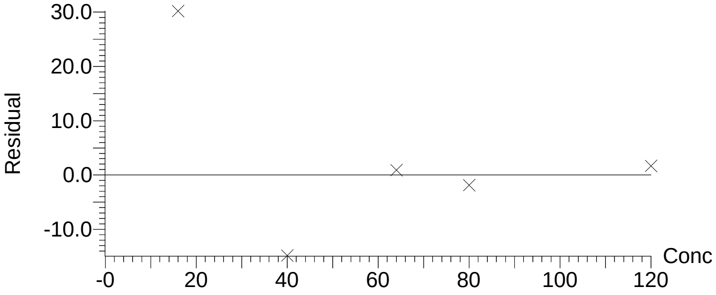

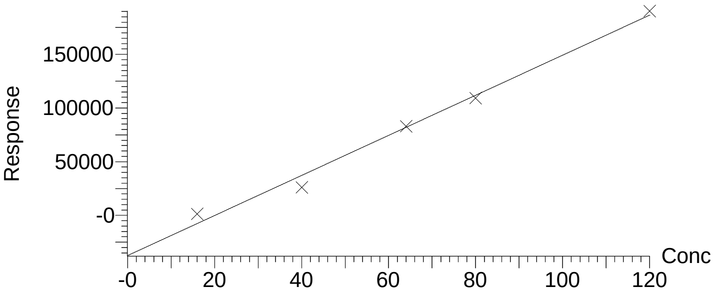

Compound name: mutang
Correlation coefficient: r = 0.991167, r^2 = 0.982411
Calibration curve: 1844.46 * x + -27630.8
Response type: External Std, Area
Curve type: Linear, Origin: Exclude, Weighting: Null, Axis trans: None

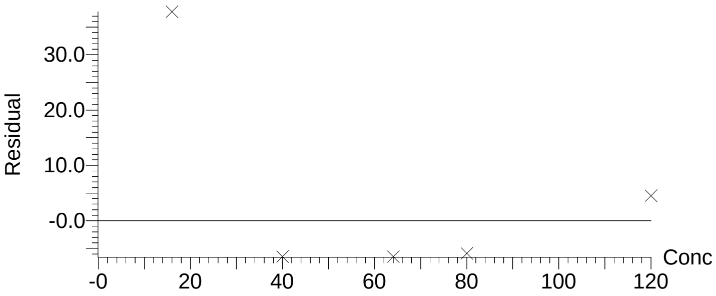

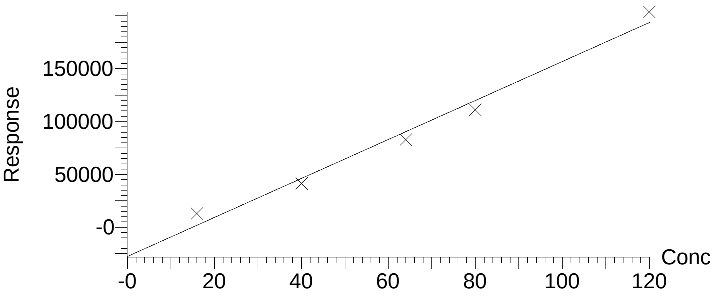

Method: F:\data\Wu_yueyan.PRO\MethDB\20240918_ tang lei_ELSD .mdb 18 Sep 2024 14:15:05
Calibration: F:\data\zhanghuien.PRO\CurveDB\20240929_tanglei.cdb 29 Sep 2024 09:53:03

Name: 20240913_Wu_sample_001, Date: 13-Sep-2024, Time: 14:00:48, ID: , Description:

guotang

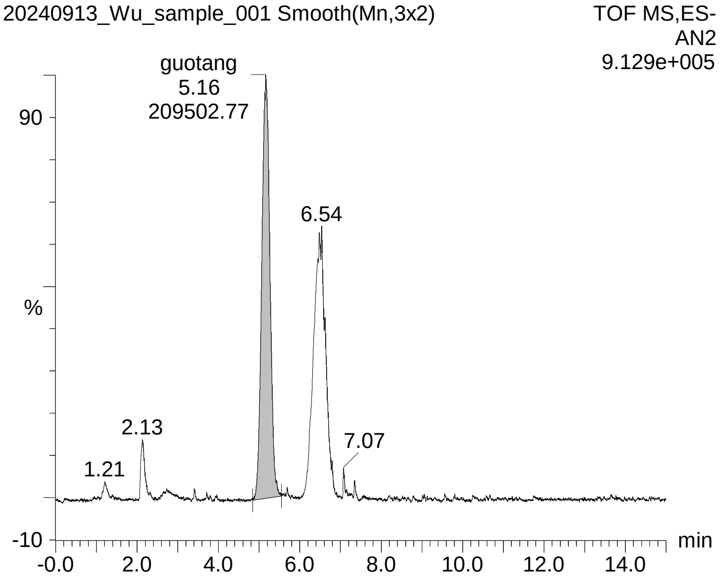

putaotang

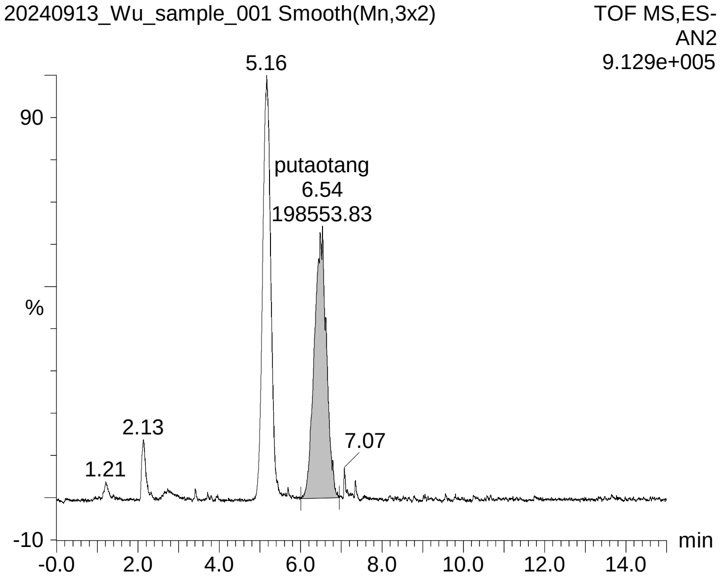

	#	Name	Sample Text	RT	Area	Std. Conc	Conc.
1	1	guotang		5.16	209502.766		133.550439
2	2	putaotang		6.54	198553.828		130.780785

Name: 20240913_Wu_sample_002, Date: 13-Sep-2024, Time: 18:17:01, ID: , Description:

guotang

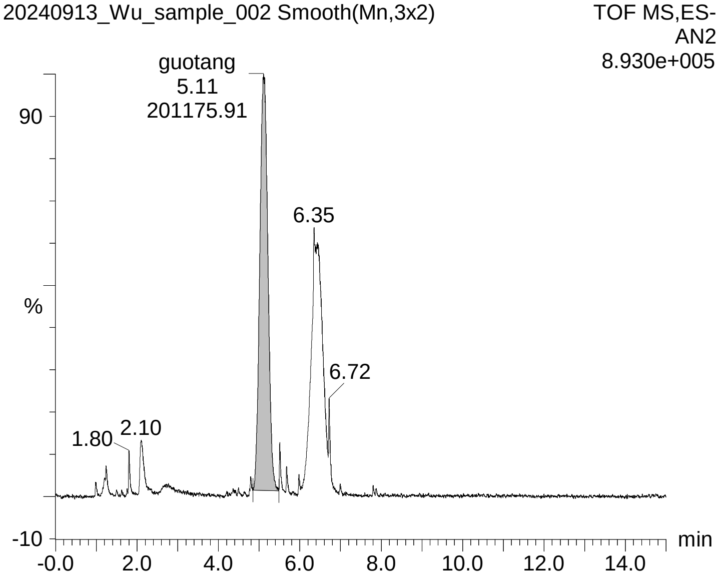

putaotang

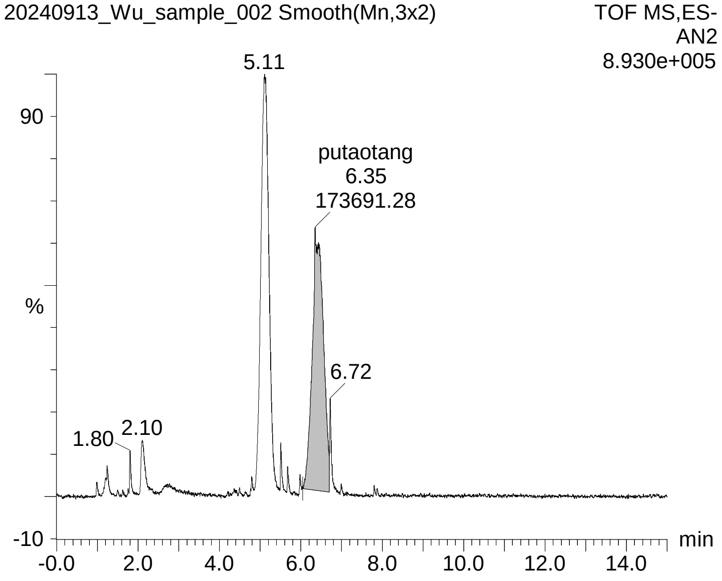

	#	Name	Sample Text	RT	Area	Std. Conc	Conc.
1	1	guotang		5.11	201175.906		128.811883
2	2	putaotang		6.35	173691.281		116.229605

Name: 20240913_Wu_sample_003, Date: 13-Sep-2024, Time: 18:32:59, ID: , Description:

guotang

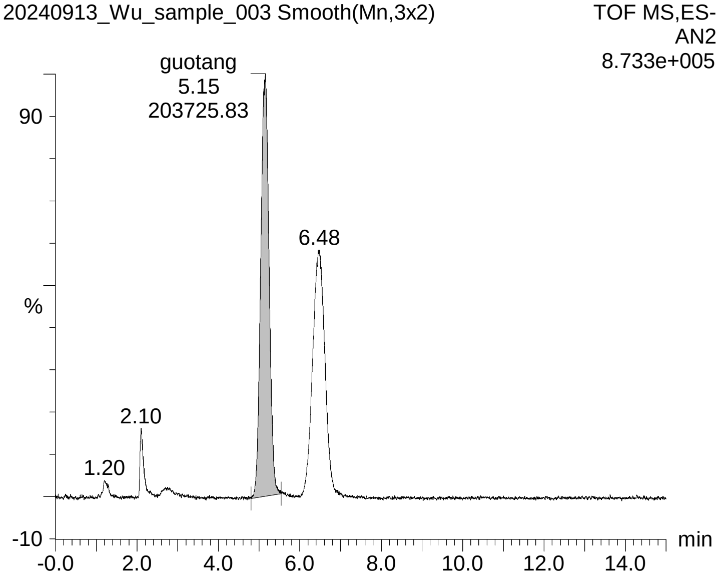

putaotang

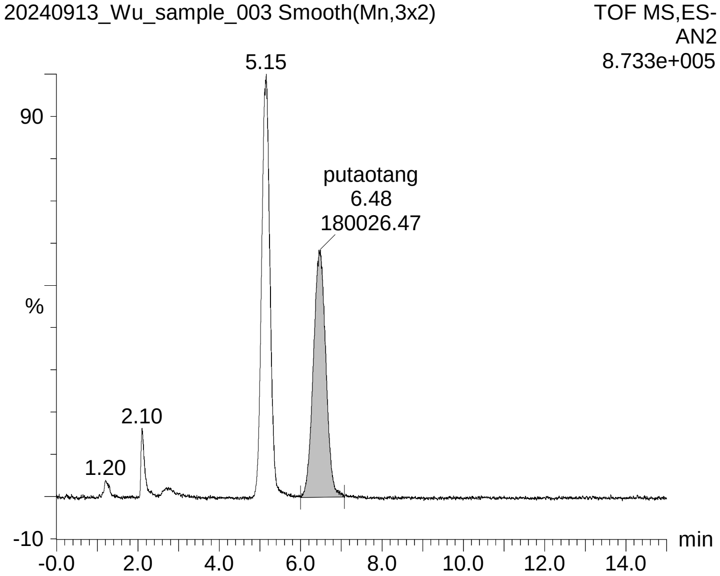

	#	Name	Sample Text	RT	Area	Std. Conc	Conc.
1	1	guotang		5.15	203725.828		130.262964
2	2	putaotang		6.48	180026.469		119.937369

project_wangzhonghua_BeiMu
Dataset: Untitled
Last Altered: Sunday, September 29, 2024 09:54:36 China Standard Time
Printed: Sunday, September 29, 2024 09:59:41 China Standard Time

Name: 20240913_Wu_sample_004, Date: 13-Sep-2024, Time: 18:49:01, ID: , Description:

guotang

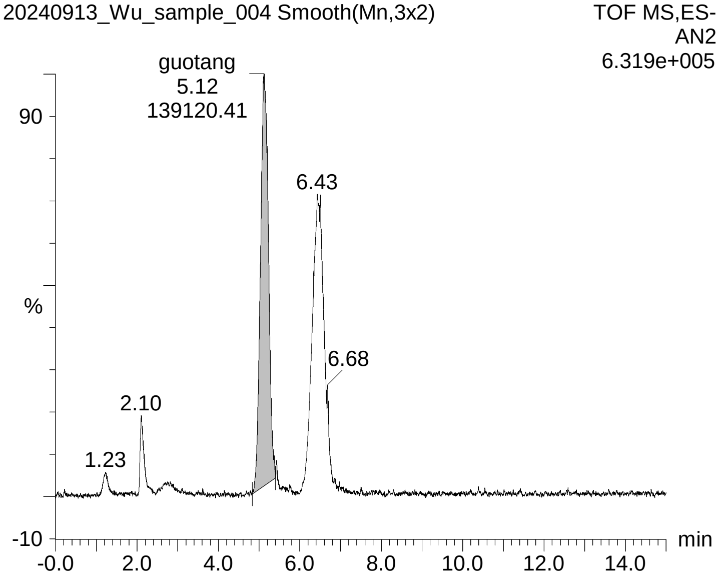

putaotang

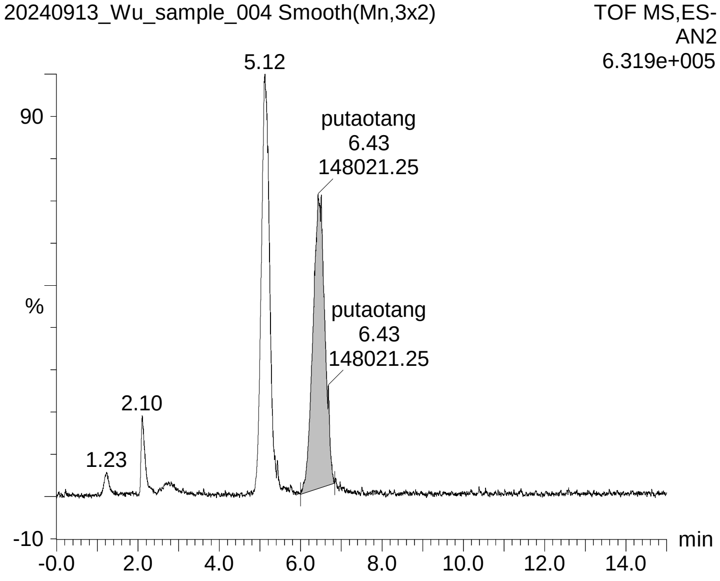

	#	Name	Sample Text	RT	Area	Std. Conc	Conc.
1	1	guotang		5.12	139120.406		93.498031
2	2	putaotang		6.43	148021.250		101.205833

Name: 20240913_Wu_sample_005, Date: 13-Sep-2024, Time: 19:05:03, ID: , Description:

guotang

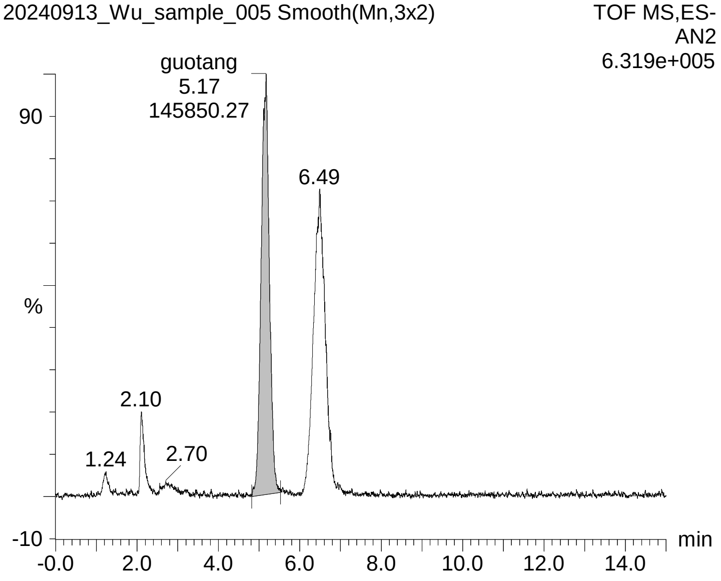

putaotang

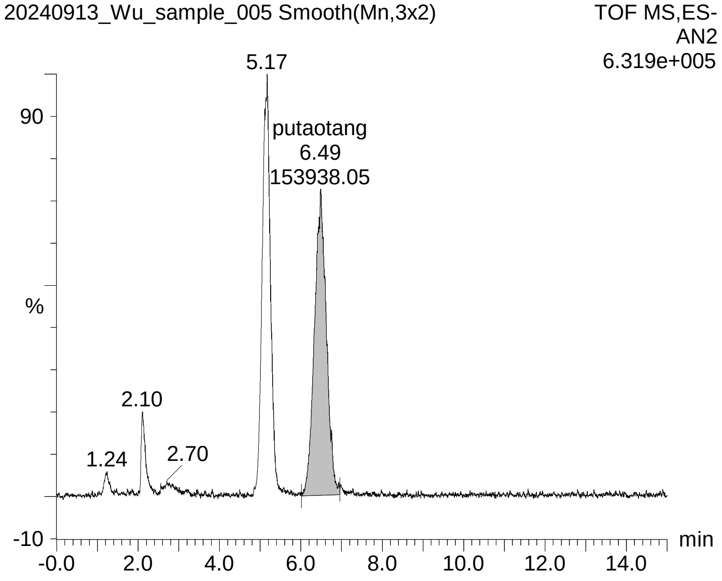

	#	Name	Sample Text	RT	Area	Std. Conc	Conc.
1	1	guotang		5.17	145850.266		97.327784
2	2	putaotang		6.49	153938.047		104.668727

project_wangzhonghua_BeiMu
Dataset: Untitled
Last Altered: Sunday, September 29, 2024 09:54:36 China Standard Time
Printed: Sunday, September 29, 2024 09:59:41 China Standard Time

Name: 20240913_Wu_sample_006, Date: 13-Sep-2024, Time: 19:21:05, ID: , Description:

guotang

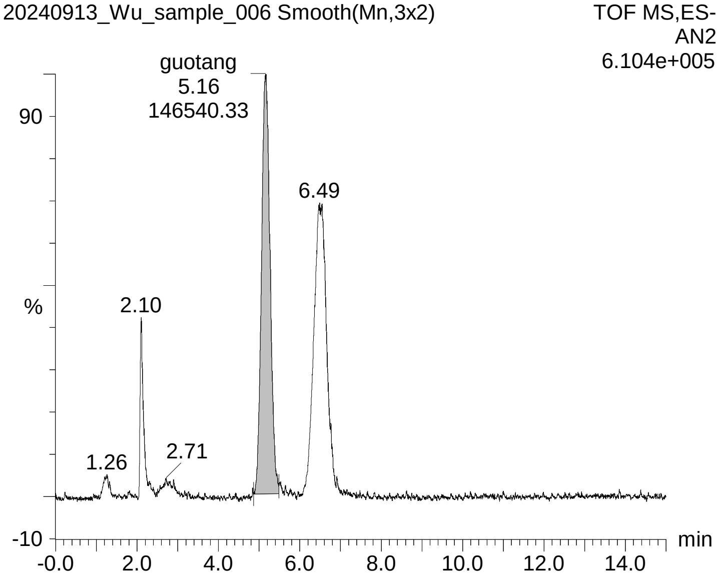

putaotang

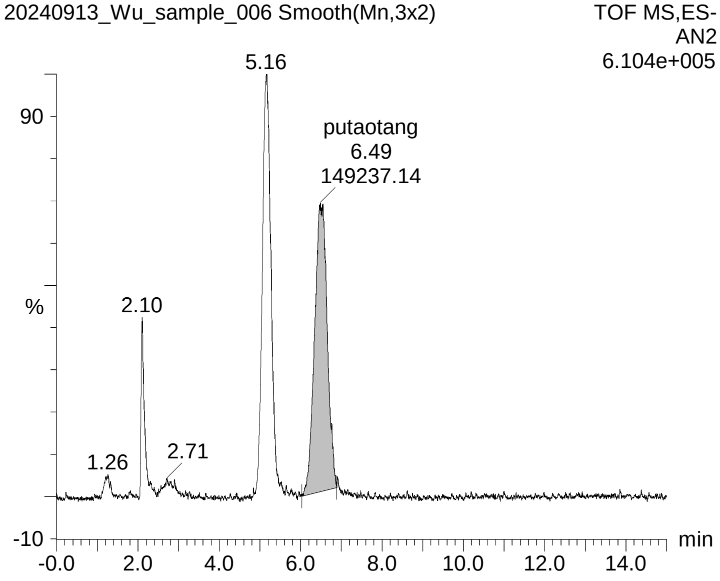

	#	Name	Sample Text	RT	Area	Std. Conc	Conc.
1	1	guotang		5.16	146540.328		97.720477
2	2	putaotang		6.49	149237.141		101.917451

Name: 20240913_Wu_sample_007, Date: 13-Sep-2024, Time: 19:37:06, ID: , Description:

guotang

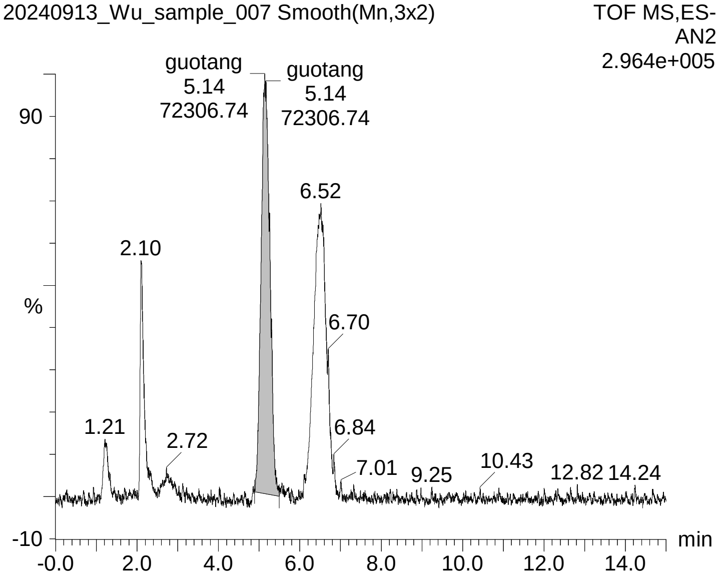

putaotang

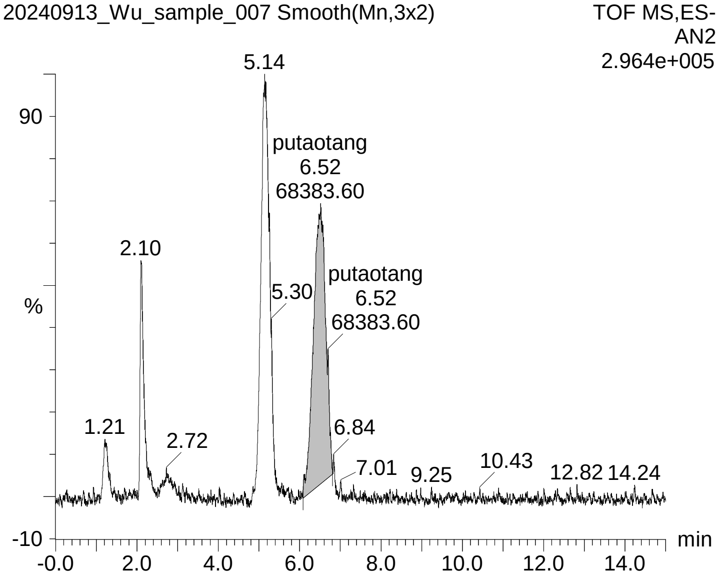

	#	Name	Sample Text	RT	Area	Std. Conc	Conc.
1	1	guotang		5.14	72306.742		55.476456
2	2	putaotang		6.52	68383.602		54.596701

Name: 20240913_Wu_sample_008, Date: 13-Sep-2024, Time: 19:53:08, ID: , Description:

guotang

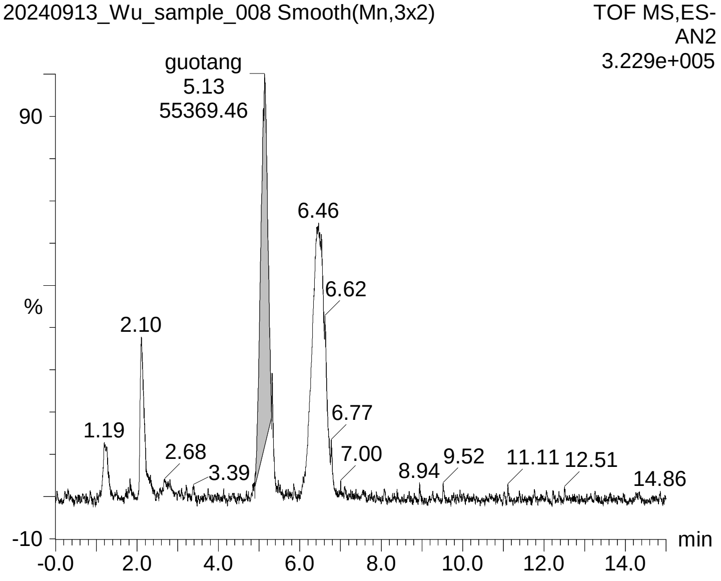

putaotang

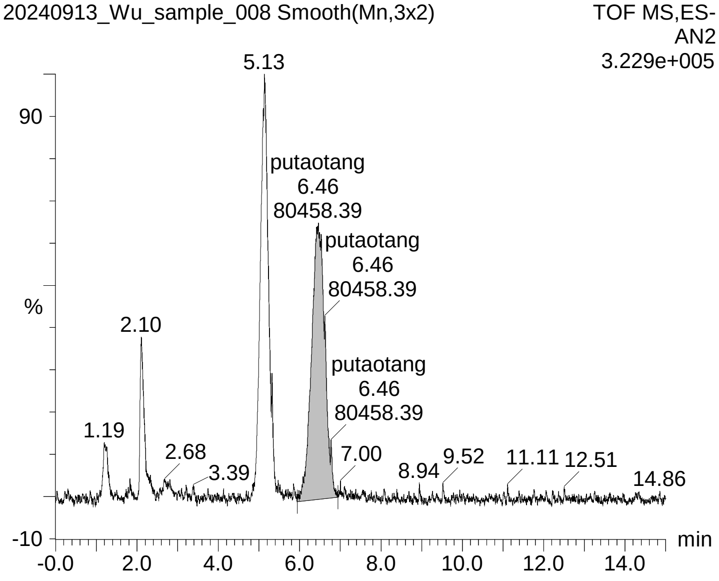

	#	Name	Sample Text	RT	Area	Std. Conc	Conc.
1	1	guotang		5.13	55369.457		45.837975
2	2	putaotang		6.46	80458.391		61.663653

Name: 20240913_Wu_sample_009, Date: 13-Sep-2024, Time: 20:09:10, ID: , Description:

guotang

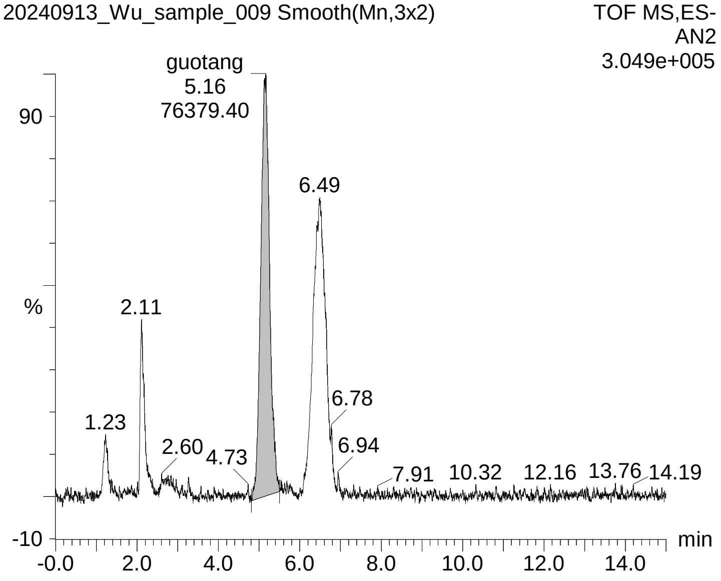

putaotang

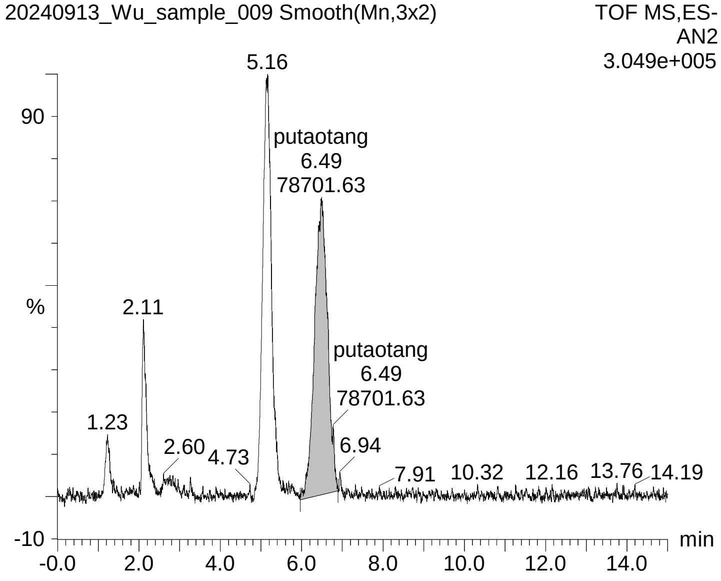

	#	Name	Sample Text	RT	Area	Std. Conc	Conc.
1	1	guotang		5.16	76379.398		57.794078
2	2	putaotang		6.49	78701.633		60.635484

project_wangzhonghua_BeiMu
Dataset: Untitled
Last Altered: Sunday, September 29, 2024 09:54:36 China Standard Time
Printed: Sunday, September 29, 2024 09:59:41 China Standard Time

Name: 20240913_Wu_sample_010, Date: 13-Sep-2024, Time: 20:25:06, ID: , Description:

guotang

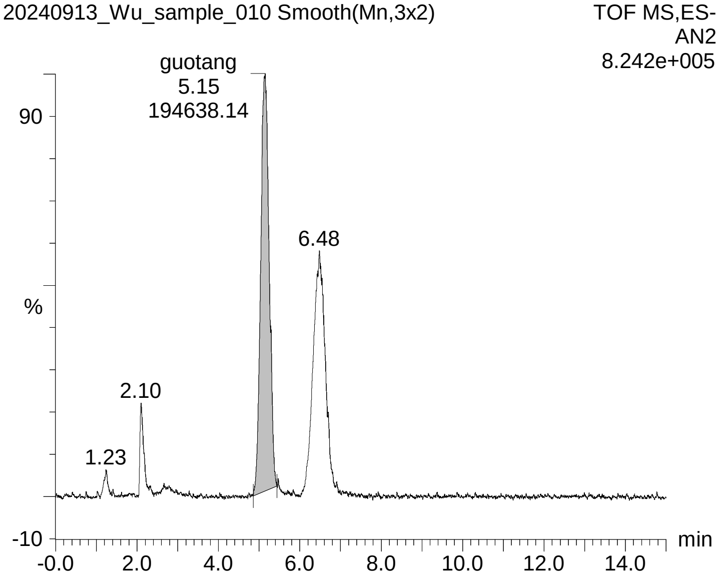

putaotang

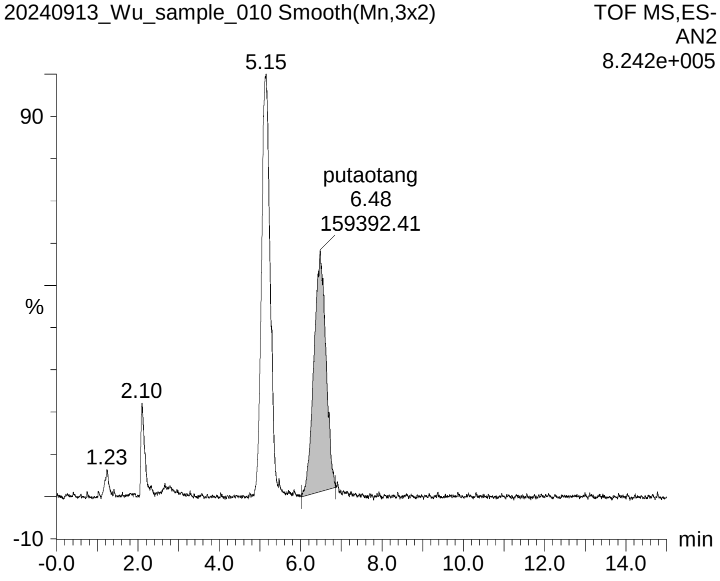

	#	Name	Sample Text	RT	Area	Std. Conc	Conc.
1	1	guotang		5.15	194638.141		125.091444
2	2	putaotang		6.48	159392.406		107.860973

Name: 20240913_Wu_sample_011, Date: 13-Sep-2024, Time: 20:41:07, ID: , Description:

guotang

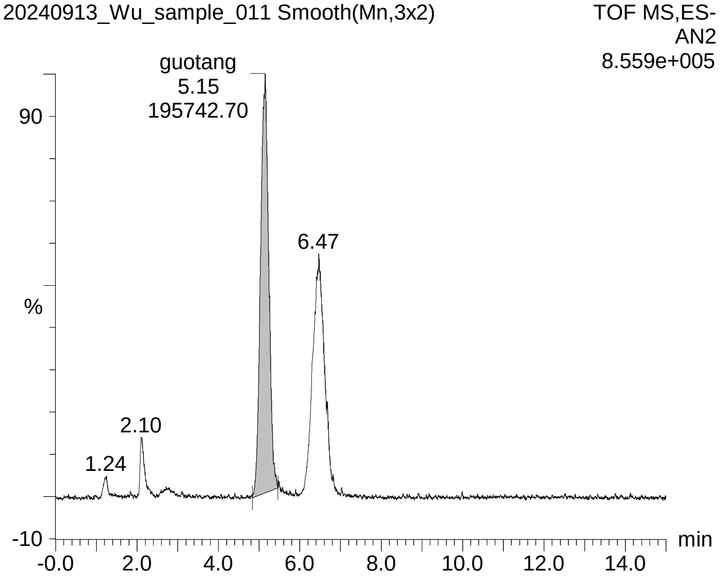

putaotang

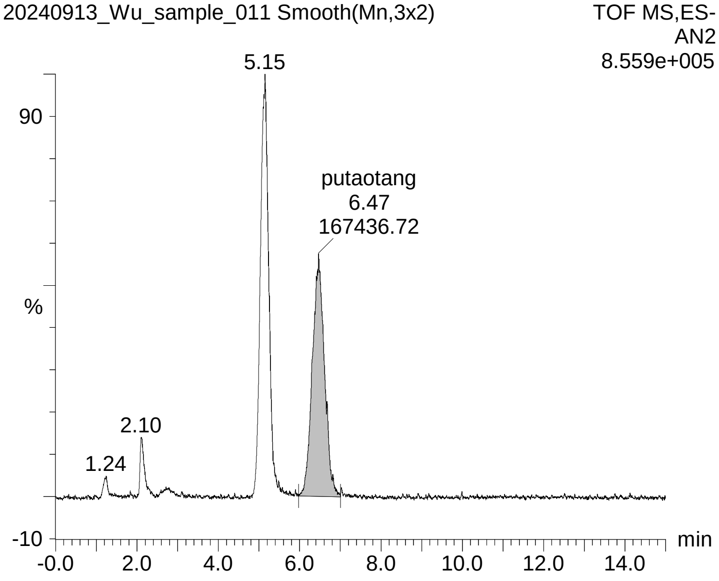

	#	Name	Sample Text	RT	Area	Std. Conc	Conc.
1	1	guotang		5.15	195742.703		125.720016
2	2	putaotang		6.47	167436.719		112.569028

Name: 20240913_Wu_sample_012, Date: 13-Sep-2024, Time: 20:57:06, ID: , Description:

guotang

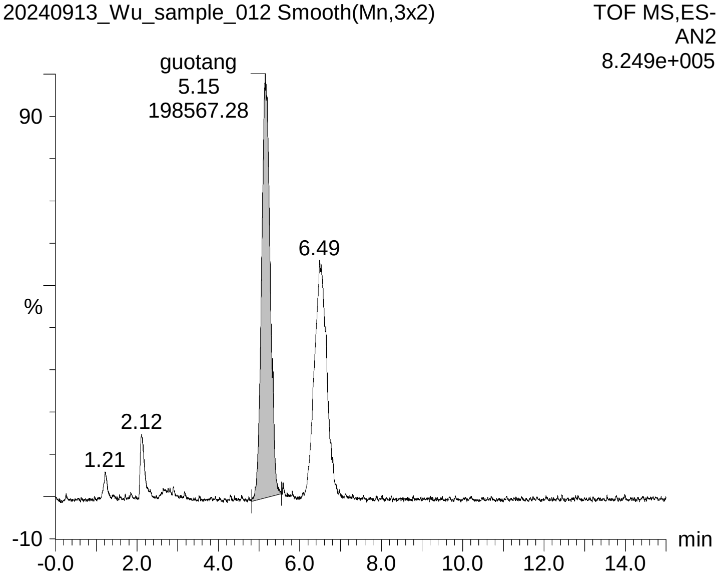

putaotang

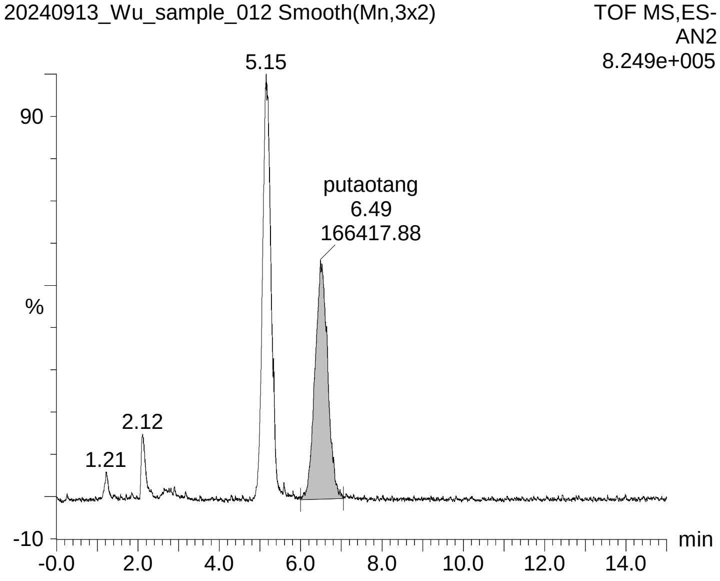

	#	Name	Sample Text	RT	Area	Std. Conc	Conc.
1	1	guotang		5.15	198567.281		127.327395
2	2	putaotang		6.49	166417.875		111.972735

project_wangzhonghua_BeiMu
Dataset: Untitled
Last Altered: Sunday, September 29, 2024 09:54:36 China Standard Time
Printed: Sunday, September 29, 2024 09:59:41 China Standard Time

Name: 20240913_Wu_sample_013, Date: 13-Sep-2024, Time: 21:13:08, ID: , Description:

guotang

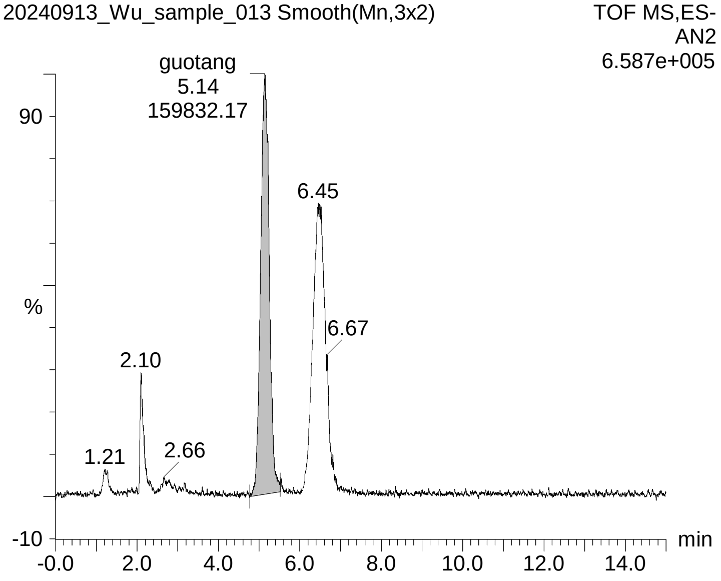

putaotang

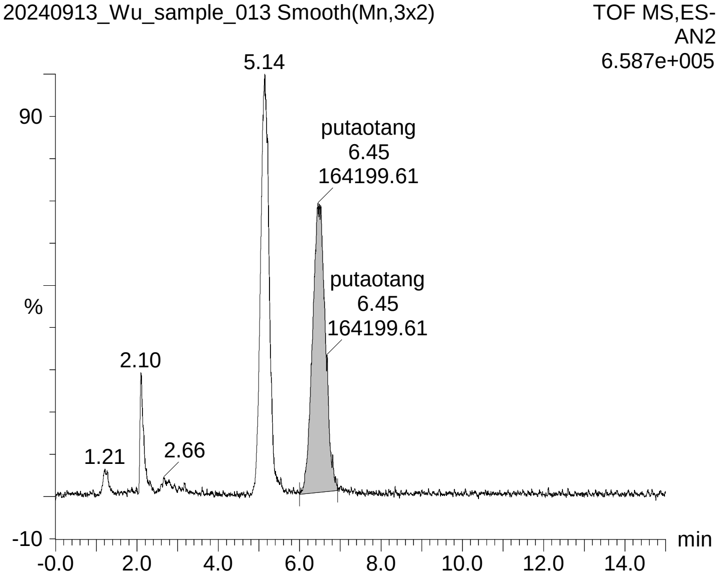

	#	Name	Sample Text	RT	Area	Std. Conc	Conc.
1	1	guotang		5.14	159832.172		105.284451
2	2	putaotang		6.45	164199.609		110.674461

Name: 20240913_Wu_sample_014, Date: 13-Sep-2024, Time: 21:29:10, ID: , Description:

guotang

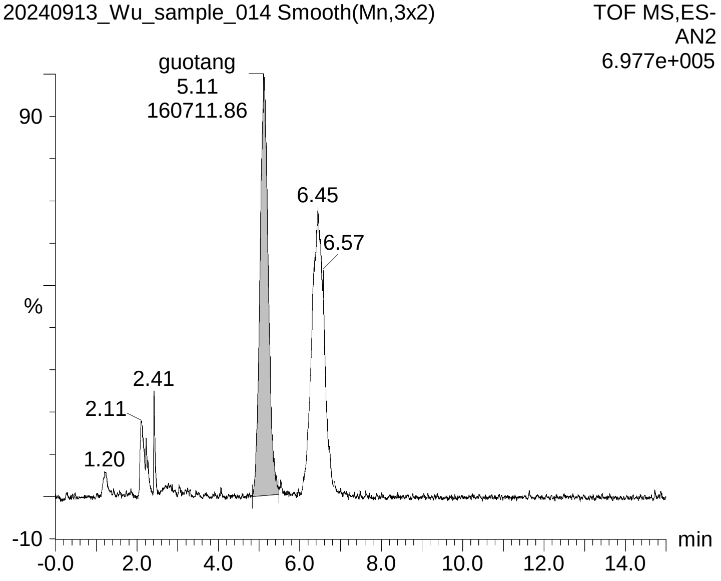

putaotang

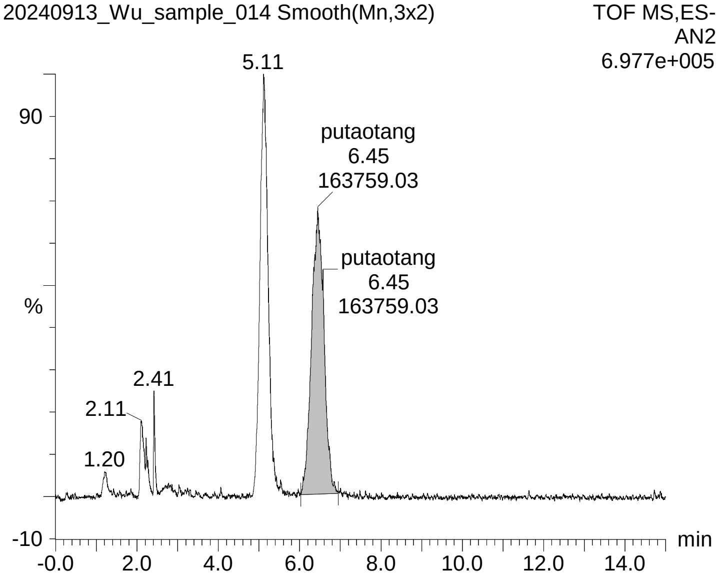

	#	Name	Sample Text	RT	Area	Std. Conc	Conc.
1	1	guotang		5.11	160711.859		105.785054
2	2	putaotang		6.45	163759.031		110.416606

Name: 20240913_Wu_sample_015, Date: 13-Sep-2024, Time: 21:45:12, ID: , Description:

guotang

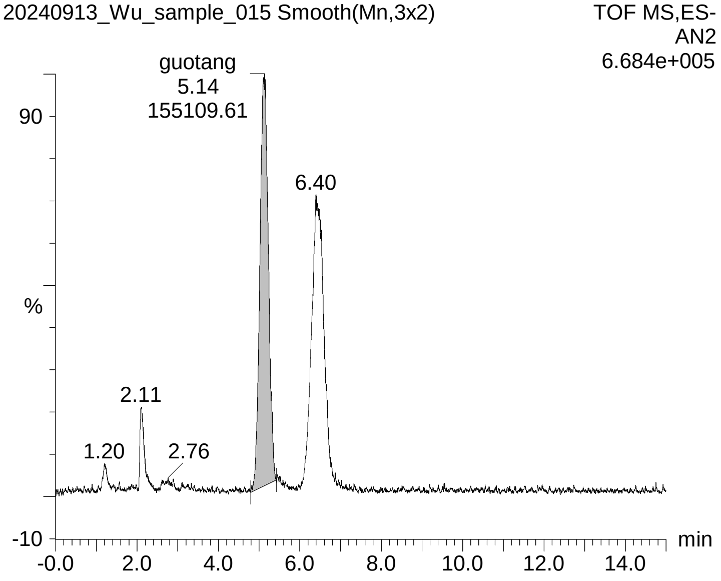

putaotang

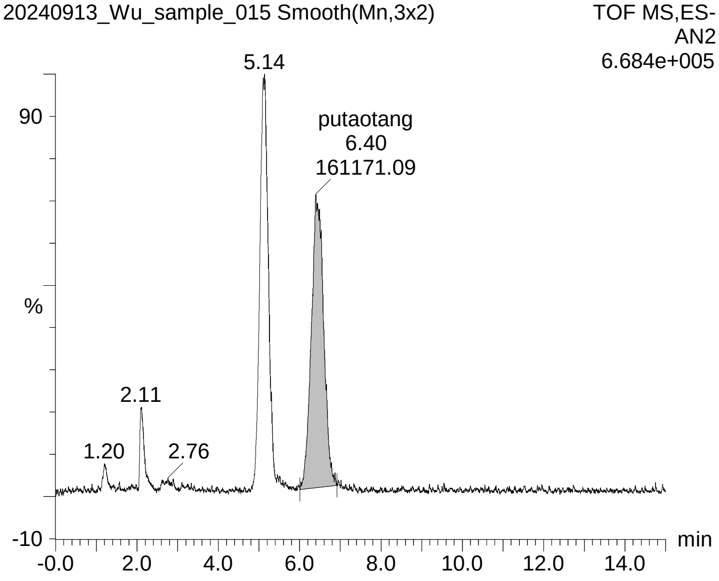

	#	Name	Sample Text	RT	Area	Std. Conc	Conc.
1	1	guotang		5.14	155109.609		102.596988
2	2	putaotang		6.40	161171.094		108.901977

project_wangzhonghua_BeiMu
Dataset: Untitled
Last Altered: Sunday, September 29, 2024 09:54:36 China Standard Time
Printed: Sunday, September 29, 2024 09:59:41 China Standard Time

Name: 20240913_Wu_sample_016, Date: 13-Sep-2024, Time: 22:01:14, ID: , Description:

guotang

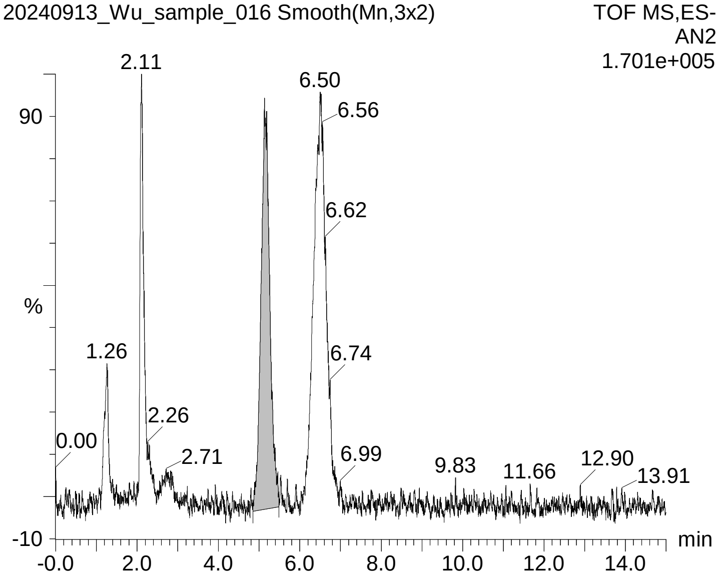

putaotang

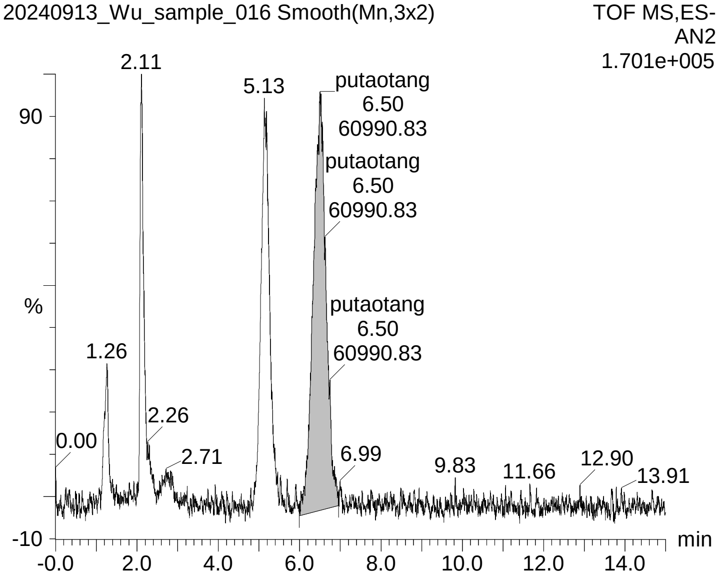

	#	Name	Sample Text	RT	Area	Std. Conc	Conc.
1	1	guotang		5.13	40412.832		37.326625
2	2	putaotang		6.50	60990.828		50.269969

project_wangzhonghua_BeiMu
Dataset: Untitled
Last Altered: Sunday, September 29, 2024 09:54:36 China Standard Time
Printed: Sunday, September 29, 2024 09:59:41 China Standard Time

Name: 20240913_Wu_sample_017, Date: 13-Sep-2024, Time: 22:17:18, ID: , Description:

guotang

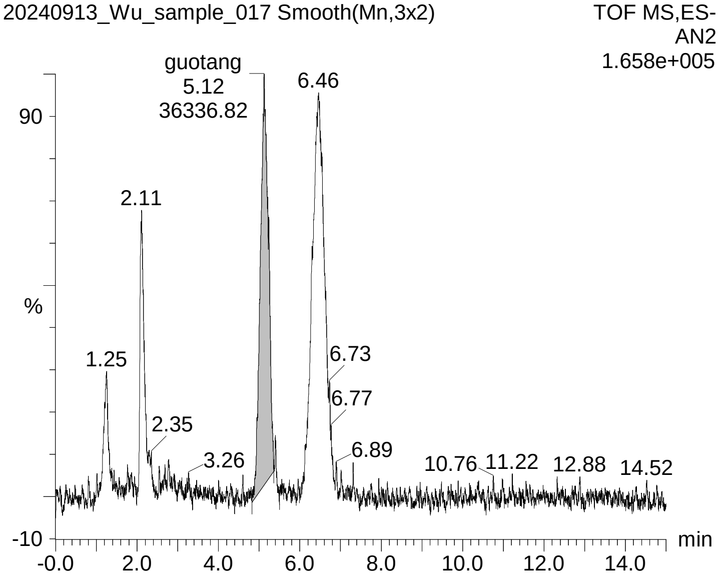

putaotang

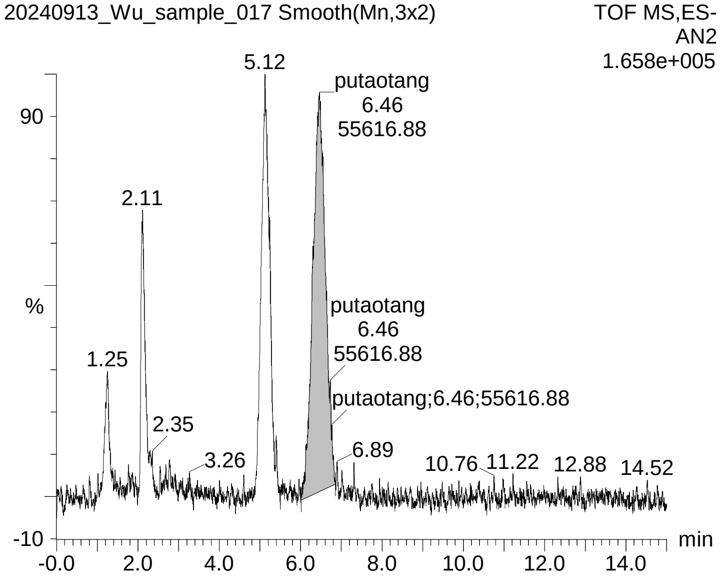

	#	Name	Sample Text	RT	Area	Std. Conc	Conc.
1	1	guotang		5.12	36336.816		35.007092
2	2	putaotang		6.46	55616.875		47.124782

project_wangzhonghua_BeiMu
Dataset: Untitled
Last Altered: Sunday, September 29, 2024 09:54:36 China Standard Time
Printed: Sunday, September 29, 2024 09:59:41 China Standard Time

Name: 20240913_Wu_sample_018, Date: 13-Sep-2024, Time: 22:33:17, ID: , Description:

guotang

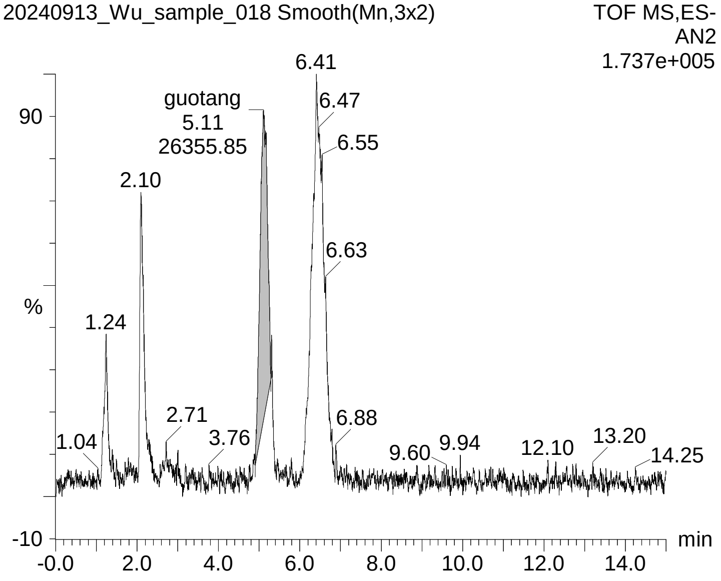

putaotang

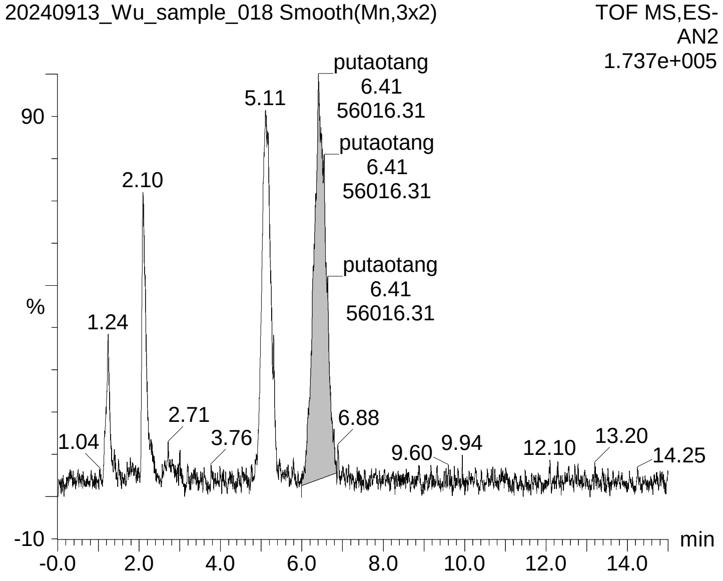

	#	Name	Sample Text	RT	Area	Std. Conc	Conc.
1	1	guotang		5.11	26355.848		29.327234
2	2	putaotang		6.41	56016.309		47.358556

Name: 20240913_Wu_sample_019, Date: 13-Sep-2024, Time: 22:49:19, ID: , Description:

guotang

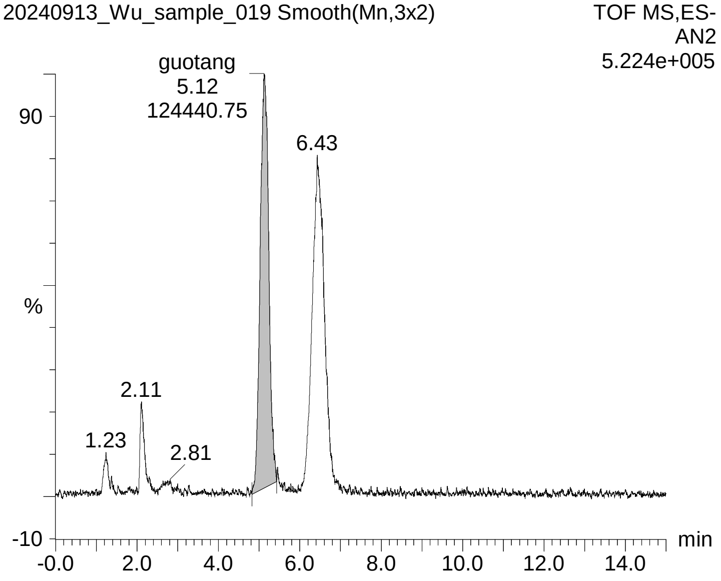

putaotang

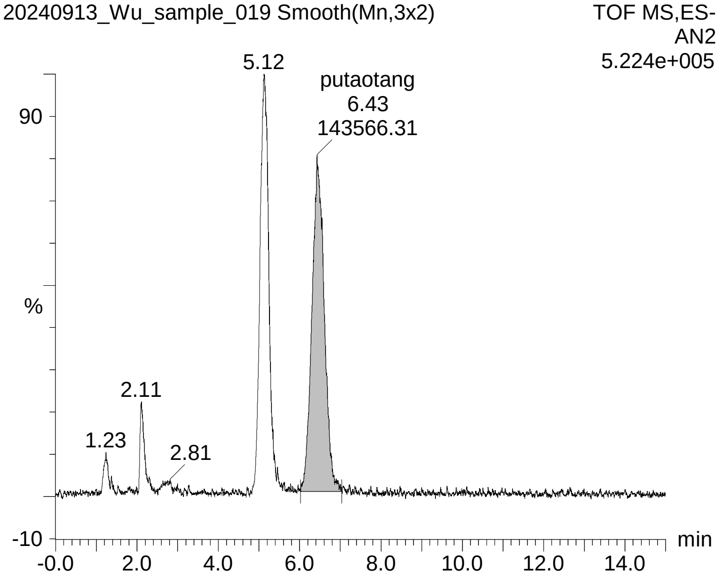

	#	Name	Sample Text	RT	Area	Std. Conc	Conc.
1	1	guotang		5.12	124440.750		85.144296
2	2	putaotang		6.43	143566.313		98.598514

project_wangzhonghua_BeiMu
Dataset: Untitled
Last Altered: Sunday, September 29, 2024 09:54:36 China Standard Time
Printed: Sunday, September 29, 2024 09:59:41 China Standard Time

Name: 20240913_Wu_sample_020, Date: 13-Sep-2024, Time: 23:05:21, ID: , Description:

guotang

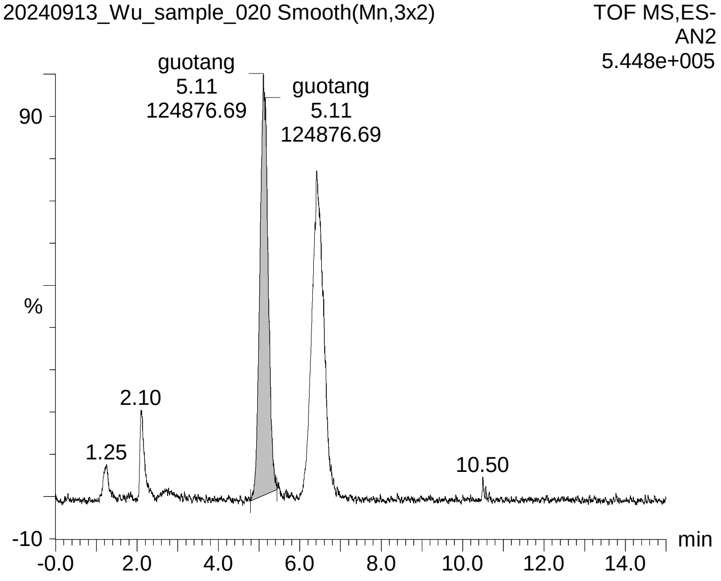

putaotang

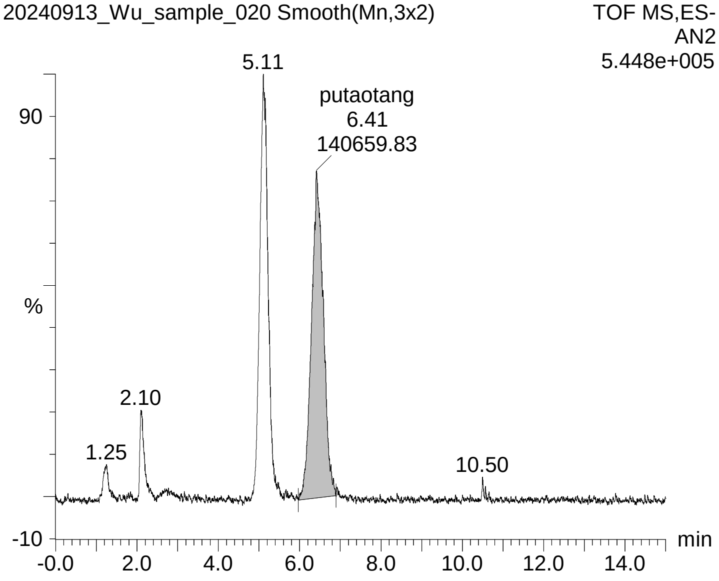

	#	Name	Sample Text	RT	Area	Std. Conc	Conc.
1	1	guotang		5.11	124876.688		85.392375
2	2	putaotang		6.41	140659.828		96.897450

project_wangzhonghua_BeiMu
Dataset: Untitled
Last Altered: Sunday, September 29, 2024 09:54:36 China Standard Time
Printed: Sunday, September 29, 2024 09:59:41 China Standard Time

Name: 20240913_Wu_sample_021, Date: 13-Sep-2024, Time: 23:21:23, ID: , Description:

guotang

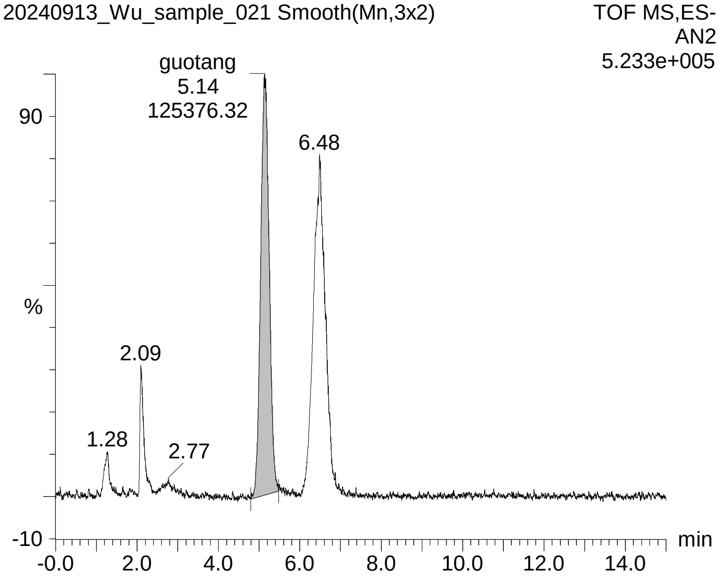

putaotang

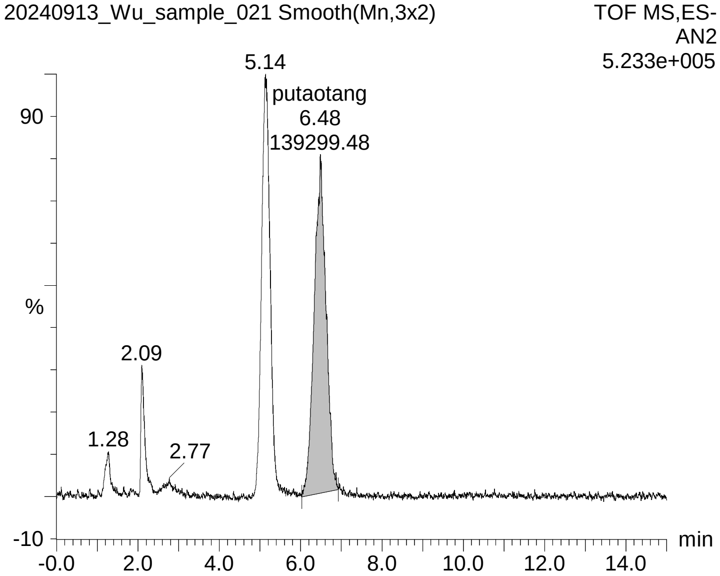

	#	Name	Sample Text	RT	Area	Std. Conc	Conc.
1	1	guotang		5.14	125376.320		85.676700
2	2	putaotang		6.48	139299.484		96.101288

Name: 20240913_Wu_sample_022, Date: 13-Sep-2024, Time: 23:37:24, ID: , Description:

guotang

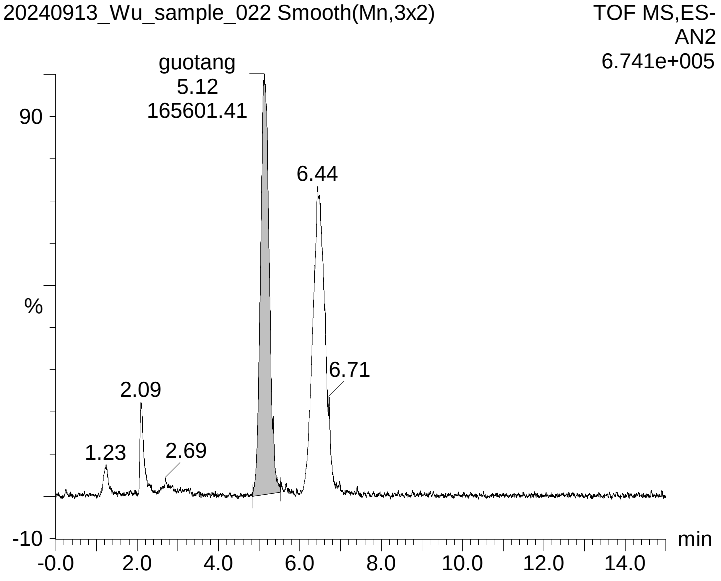

putaotang

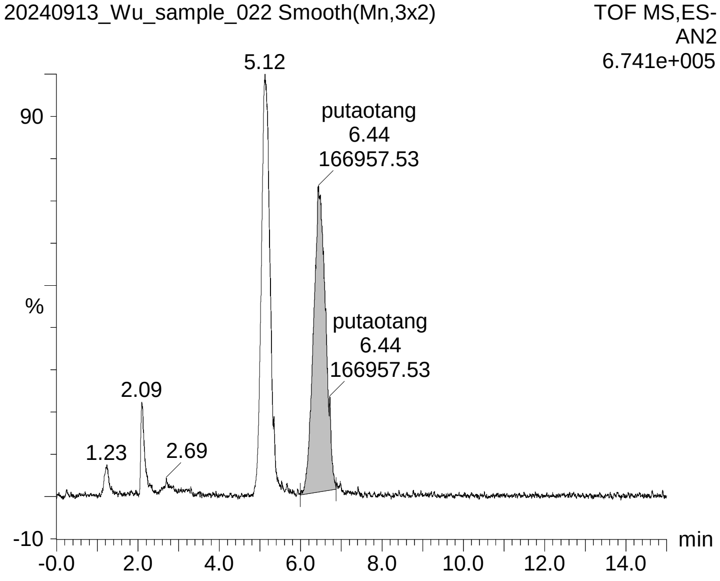

	#	Name	Sample Text	RT	Area	Std. Conc	Conc.
1	1	guotang		5.12	165601.406		108.567543
2	2	putaotang		6.44	166957.531		112.288576

Name: 20240913_Wu_sample_023, Date: 13-Sep-2024, Time: 23:53:26, ID: , Description:

guotang

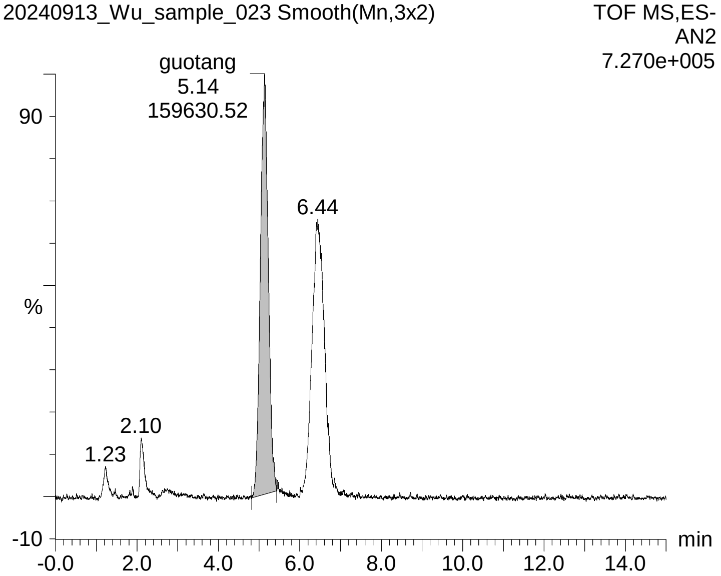

putaotang

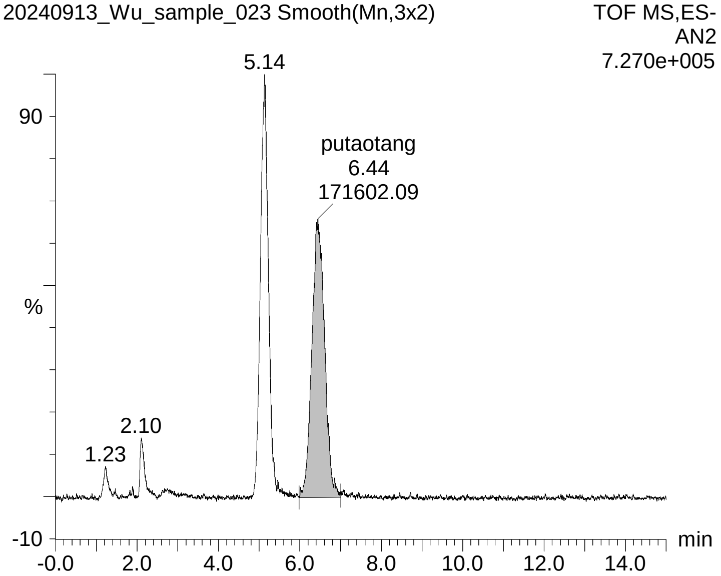

	#	Name	Sample Text	RT	Area	Std. Conc	Conc.
1	1	guotang		5.14	159630.516		105.169695
2	2	putaotang		6.44	171602.094		115.006877

Name: 20240913_Wu_sample_024, Date: 14-Sep-2024, Time: 00:09:28, ID: , Description:

guotang

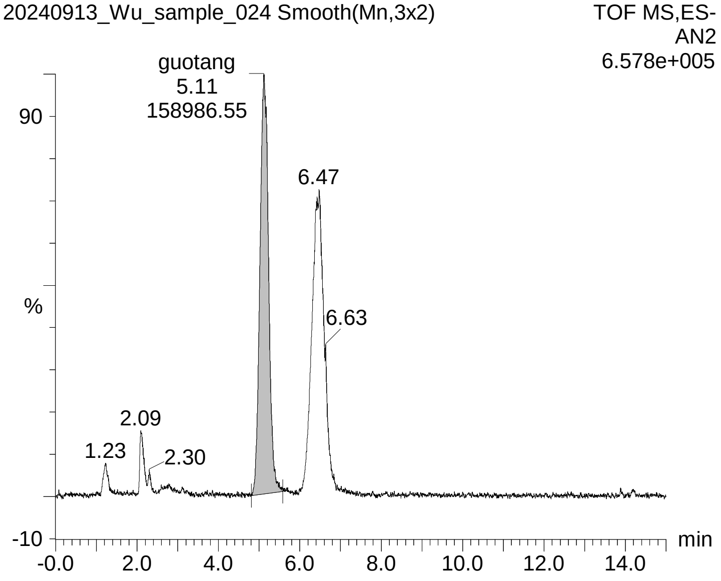

putaotang

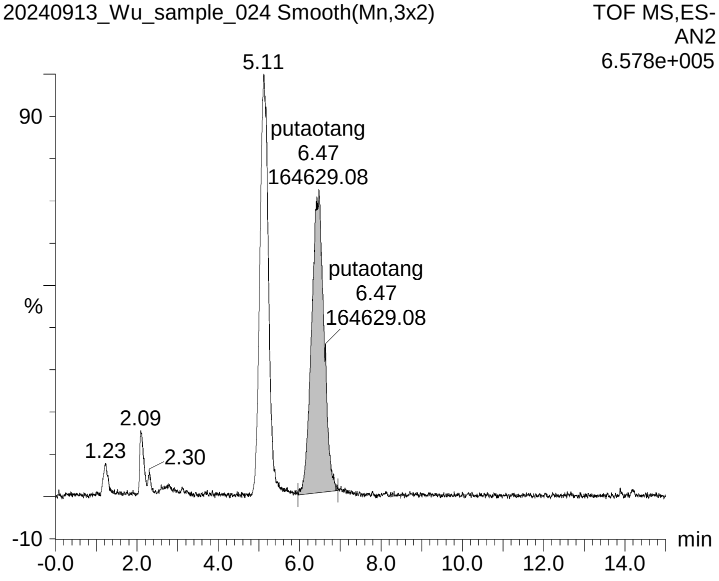

	#	Name	Sample Text	RT	Area	Std. Conc	Conc.
1	1	guotang		5.11	158986.547		104.803233
2	2	putaotang		6.47	164629.078		110.925814

project_wangzhonghua_BeiMu
Dataset: Untitled
Last Altered: Sunday, September 29, 2024 09:54:36 China Standard Time
Printed: Sunday, September 29, 2024 09:59:41 China Standard Time

Name: 20240913_Wu_sample_025, Date: 14-Sep-2024, Time: 00:25:30, ID: , Description:

guotang

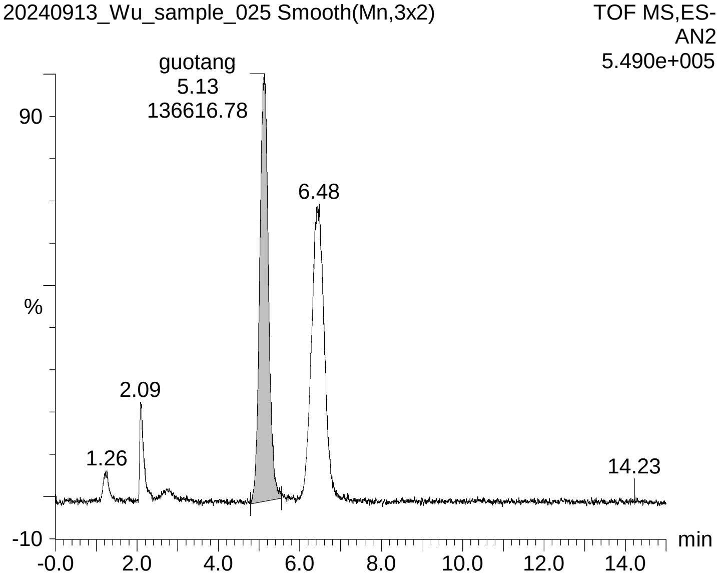

putaotang

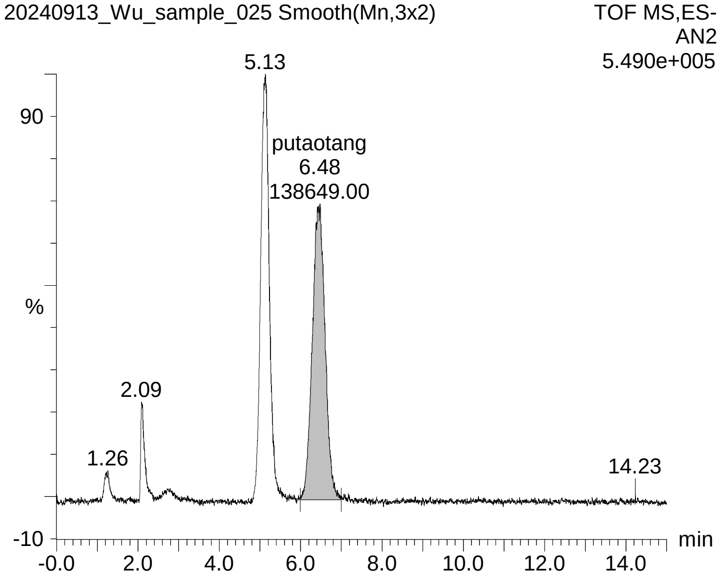

	#	Name	Sample Text	RT	Area	Std. Conc	Conc.
1	1	guotang		5.13	136616.781		92.073296
2	2	putaotang		6.48	138649.000		95.720583

Name: 20240913_Wu_sample_026, Date: 14-Sep-2024, Time: 00:41:31, ID: , Description:

guotang

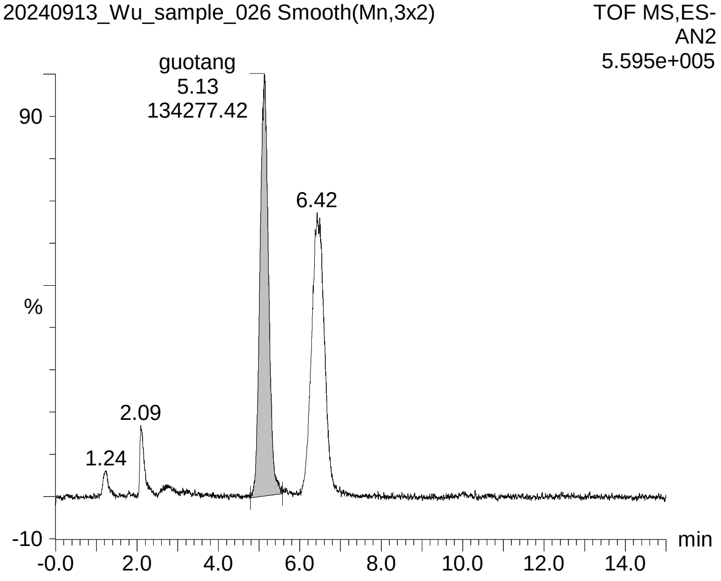

putaotang

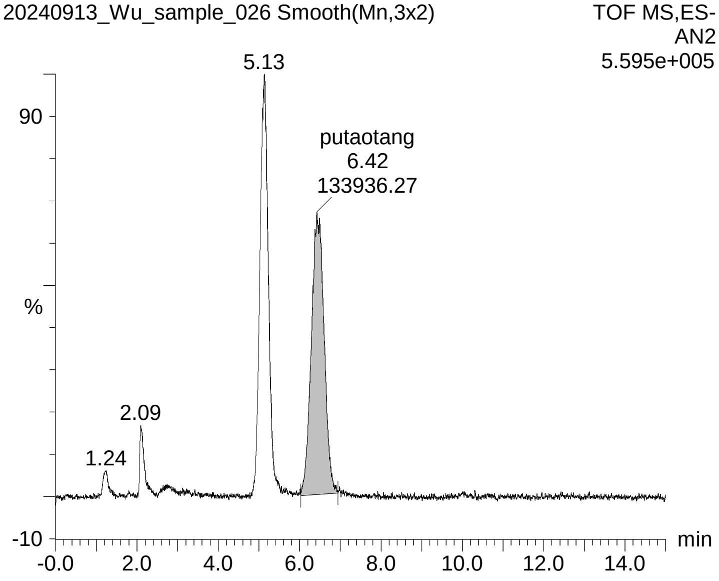

	#	Name	Sample Text	RT	Area	Std. Conc	Conc.
1	1	guotang		5.13	134277.422		90.742039
2	2	putaotang		6.42	133936.266		92.962384

project_wangzhonghua_BeiMu
Dataset: Untitled
Last Altered: Sunday, September 29, 2024 09:54:36 China Standard Time
Printed: Sunday, September 29, 2024 09:59:41 China Standard Time

Name: 20240913_Wu_sample_027, Date: 14-Sep-2024, Time: 00:57:33, ID: , Description:

guotang

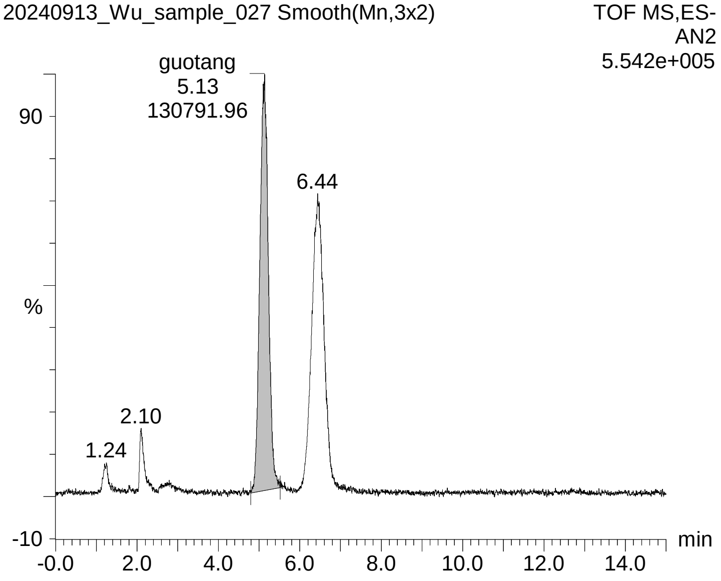

putaotang

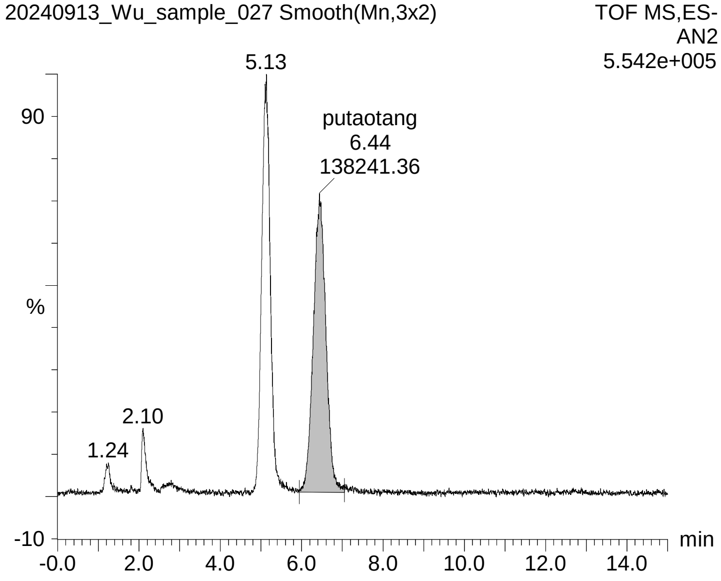

	#	Name	Sample Text	RT	Area	Std. Conc	Conc.
1	1	guotang		5.13	130791.961		88.758572
2	2	putaotang		6.44	138241.359		95.482005

Name: 20240913_Wu_sample_028, Date: 14-Sep-2024, Time: 01:13:35, ID: , Description:

guotang

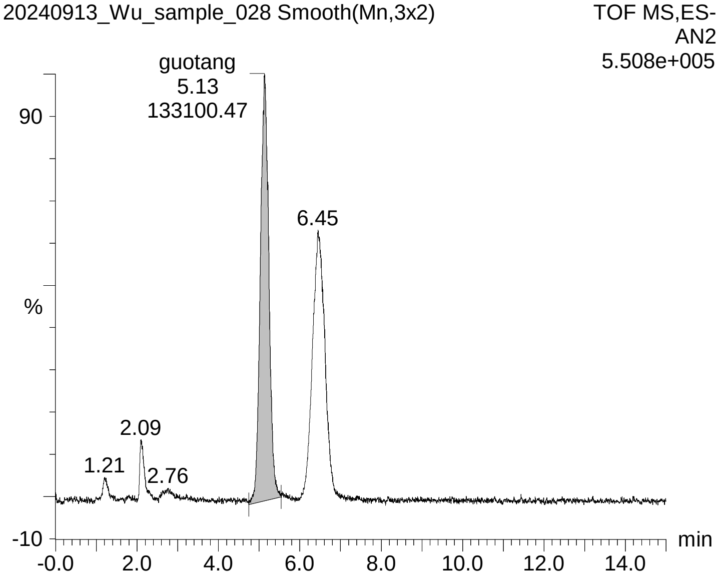

putaotang

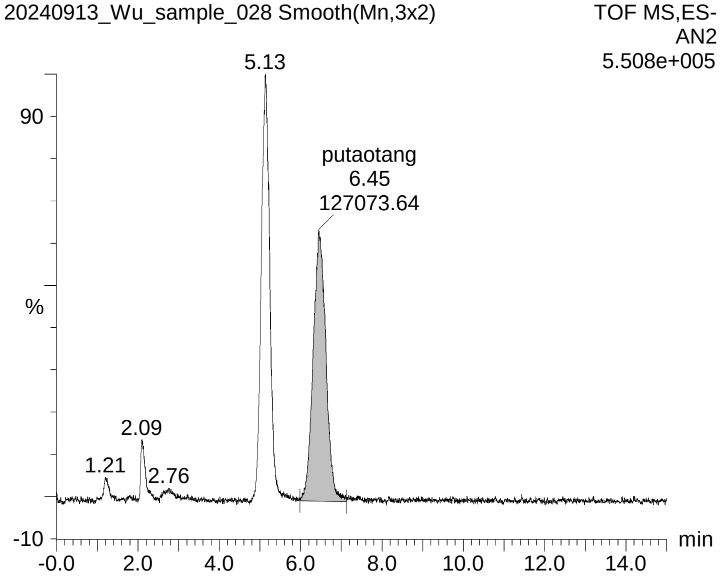

	#	Name	Sample Text	RT	Area	Std. Conc	Conc.
1	1	guotang		5.13	133100.469		90.072272
2	2	putaotang		6.45	127073.641		88.945929

project_wangzhonghua_BeiMu
Dataset: Untitled
Last Altered: Sunday, September 29, 2024 09:54:36 China Standard Time
Printed: Sunday, September 29, 2024 09:59:41 China Standard Time

Name: 20240913_Wu_sample_029, Date: 14-Sep-2024, Time: 01:29:38, ID: , Description:

guotang

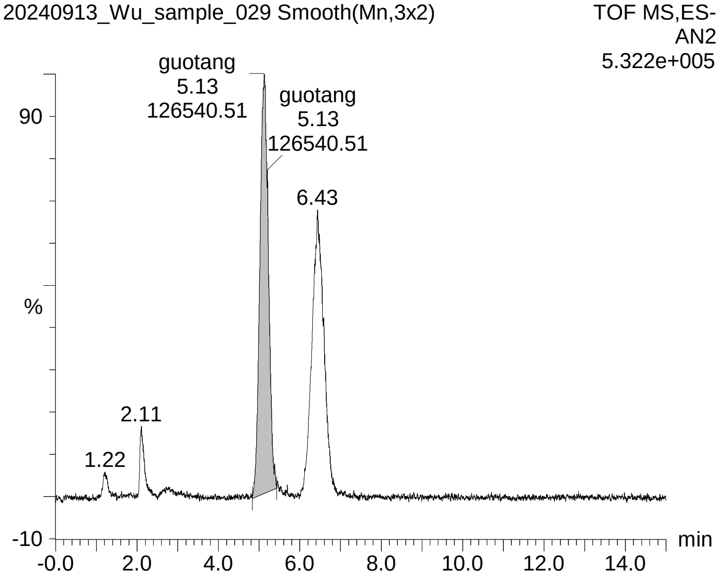

putaotang

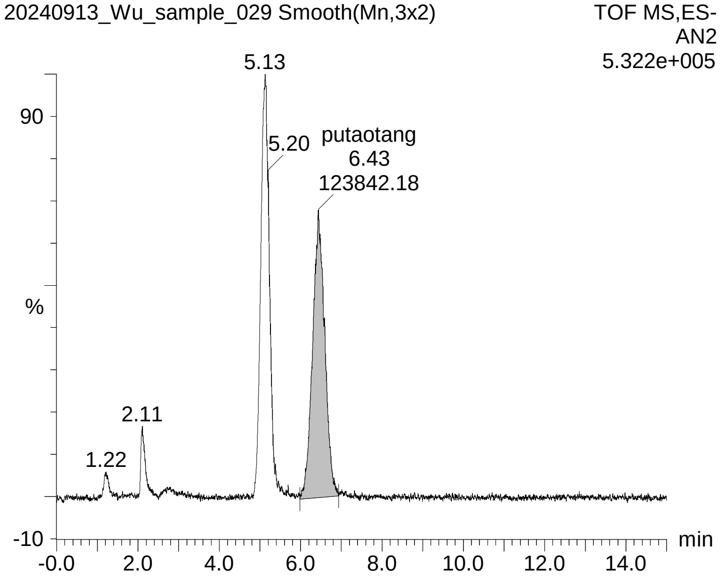

	#	Name	Sample Text	RT	Area	Std. Conc	Conc.
1	1	guotang		5.13	126540.508		86.339203
2	2	putaotang		6.43	123842.180		87.054668

Name: 20240913_Wu_sample_030, Date: 14-Sep-2024, Time: 01:45:40, ID: , Description:

guotang

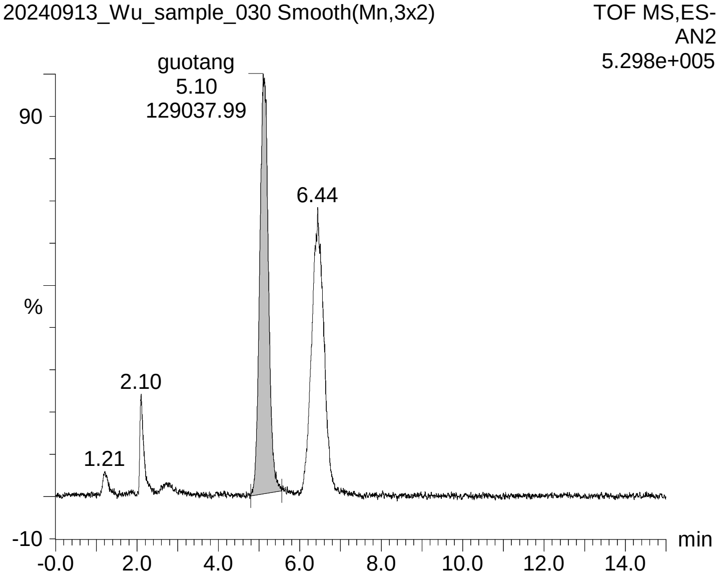

putaotang

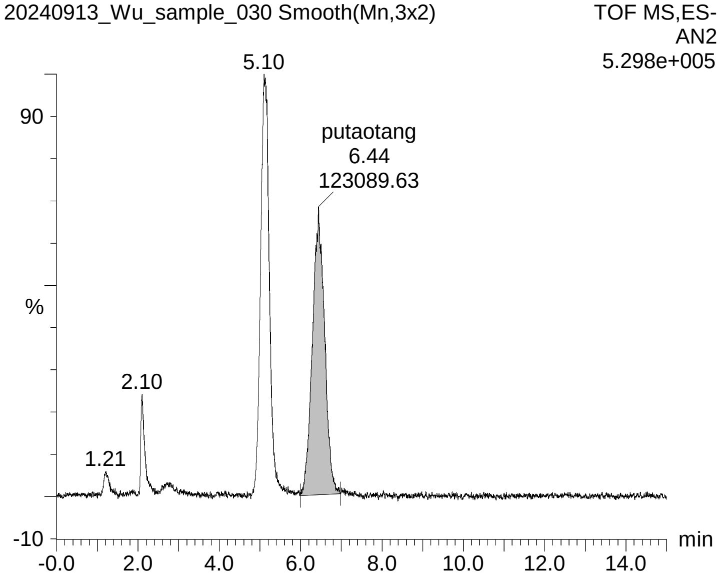

	#	Name	Sample Text	RT	Area	Std. Conc	Conc.
1	1	guotang		5.10	129037.992		87.760443
2	2	putaotang		6.44	123089.625		86.614224

Name: 20240913_Wu_sample_031, Date: 14-Sep-2024, Time: 02:01:41, ID: , Description:

guotang

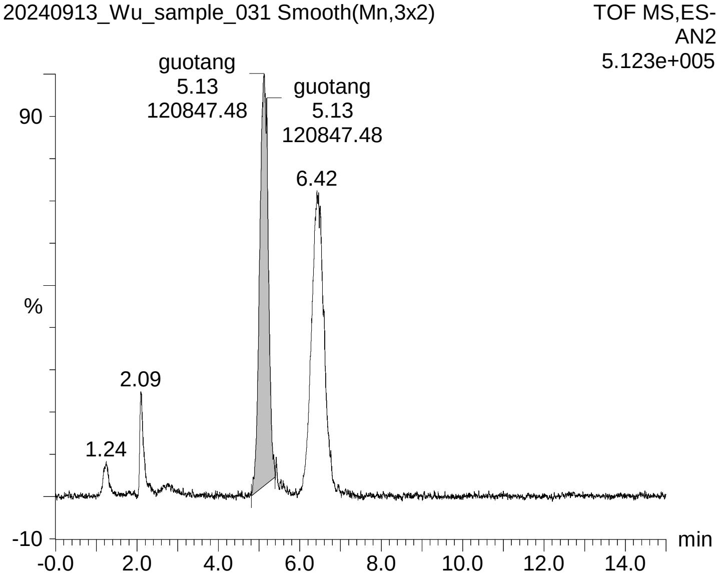

putaotang

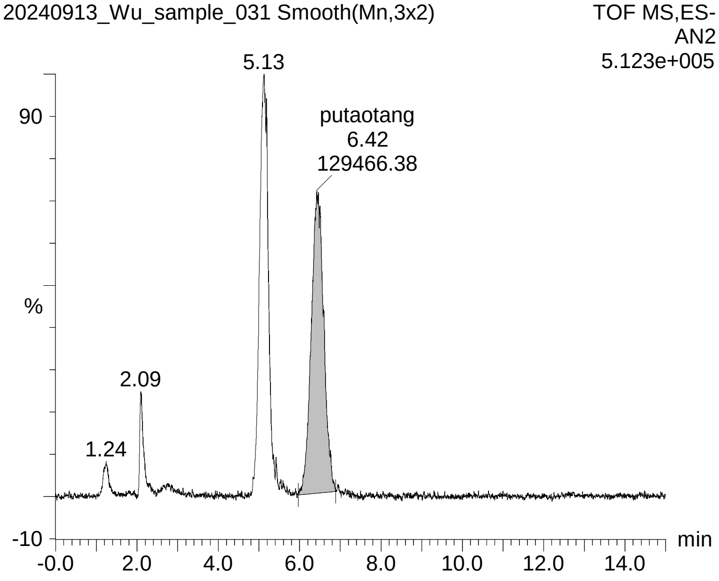

	#	Name	Sample Text	RT	Area	Std. Conc	Conc.
1	1	guotang		5.13	120847.477		83.099476
2	2	putaotang		6.42	129466.383		90.346318

project_wangzhonghua_BeiMu
Dataset: Untitled
Last Altered: Sunday, September 29, 2024 09:54:36 China Standard Time
Printed: Sunday, September 29, 2024 09:59:41 China Standard Time

Name: 20240913_Wu_sample_032, Date: 14-Sep-2024, Time: 02:17:43, ID: , Description:

guotang

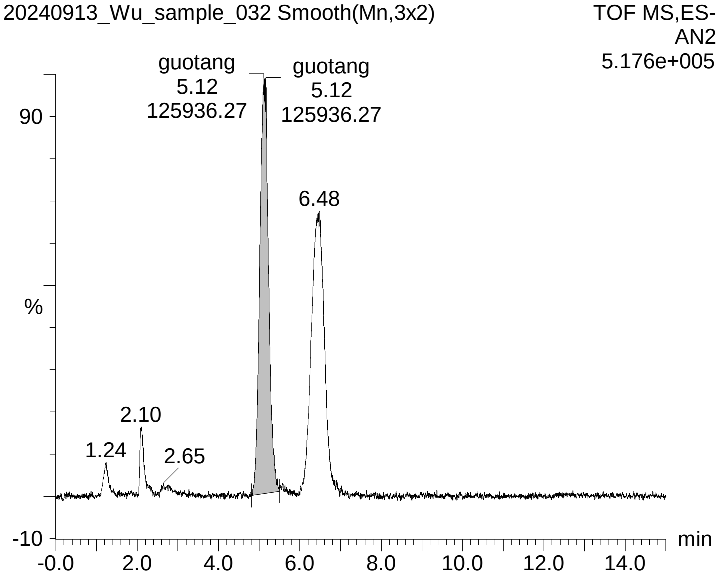

putaotang

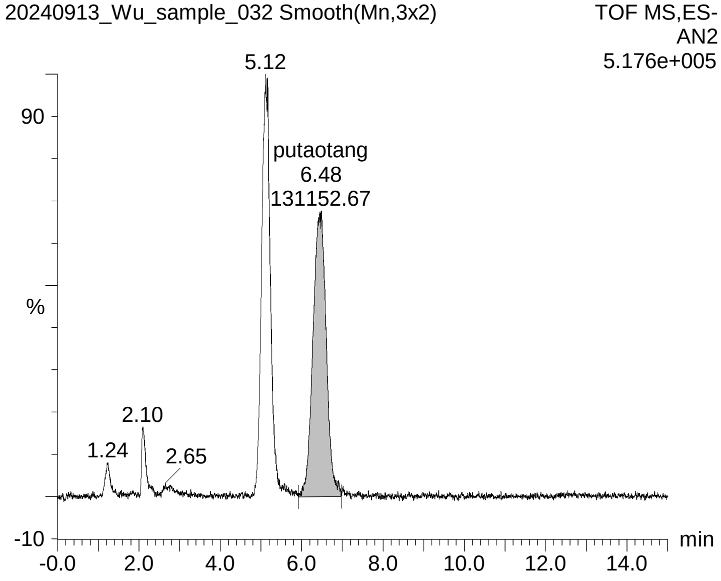

	#	Name	Sample Text	RT	Area	Std. Conc	Conc.
1	1	guotang		5.12	125936.273		85.995351
2	2	putaotang		6.48	131152.672		91.333244

Name: 20240913_Wu_sample_033, Date: 14-Sep-2024, Time: 02:33:45, ID: , Description:

guotang

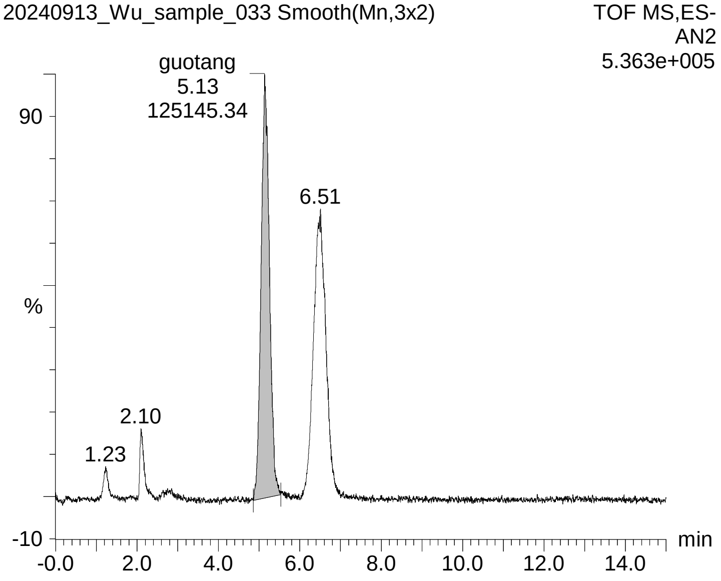

putaotang

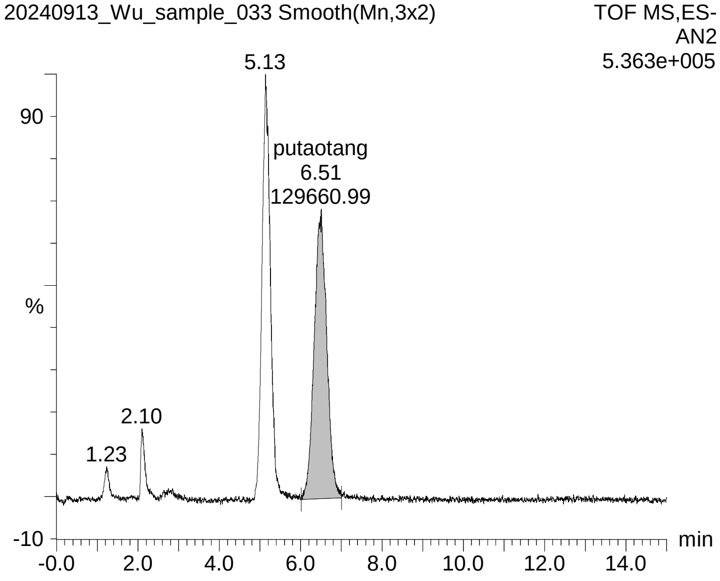

	#	Name	Sample Text	RT	Area	Std. Conc	Conc.
1	1	guotang		5.13	125145.344		85.545258
2	2	putaotang		6.51	129660.992		90.460216

Name: 20240913_Wu_sample_034, Date: 14-Sep-2024, Time: 02:49:47, ID: , Description:

guotang

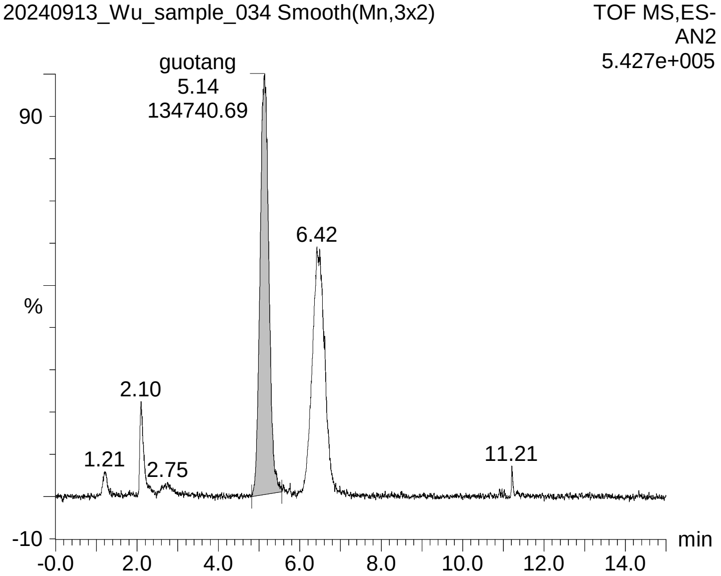

putaotang

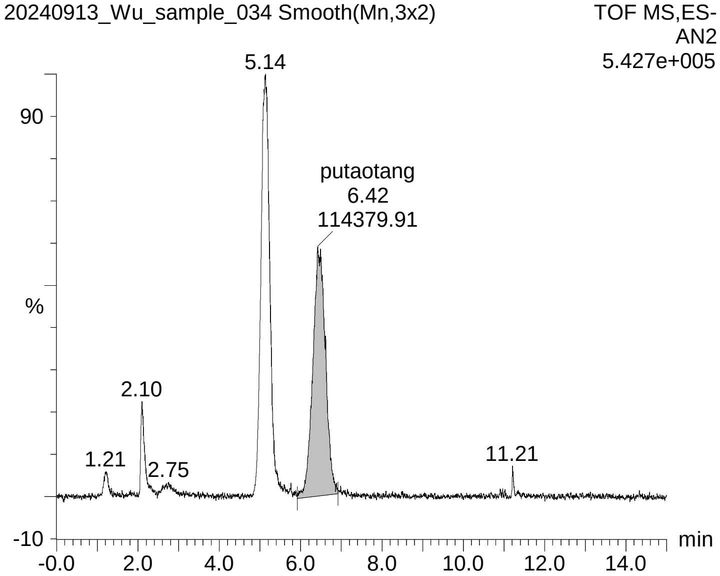

	#	Name	Sample Text	RT	Area	Std. Conc	Conc.
1	1	guotang		5.14	134740.688		91.005670
2	2	putaotang		6.42	114379.906		81.516730

project_wangzhonghua_BeiMu
Dataset: Untitled
Last Altered: Sunday, September 29, 2024 09:54:36 China Standard Time
Printed: Sunday, September 29, 2024 09:59:41 China Standard Time

Name: 20240913_Wu_sample_035, Date: 14-Sep-2024, Time: 03:05:48, ID: , Description:

guotang

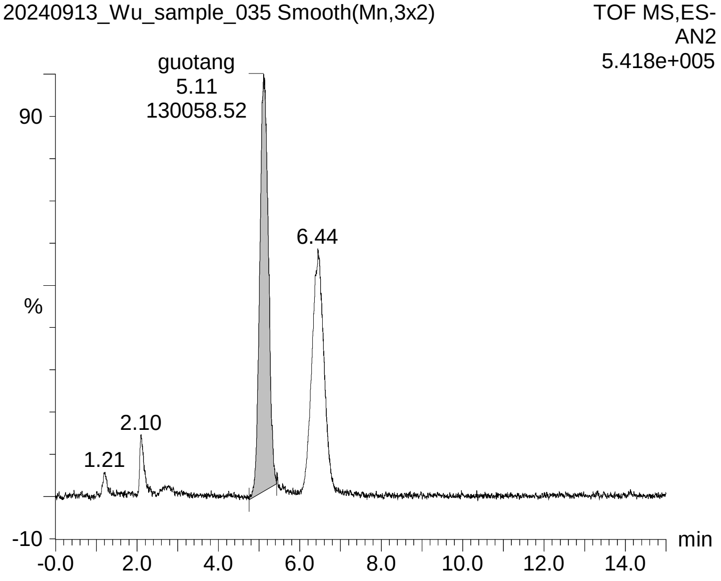

putaotang

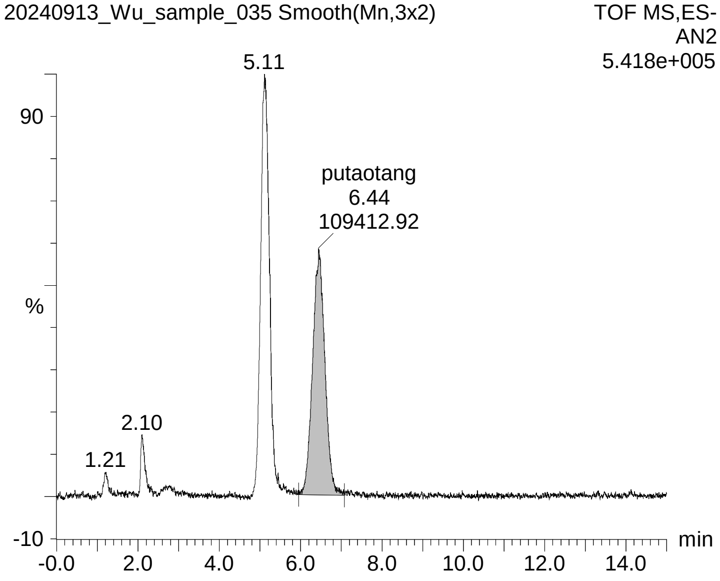

	#	Name	Sample Text	RT	Area	Std. Conc	Conc.
1	1	guotang		5.11	130058.523		88.341195
2	2	putaotang		6.44	109412.922		78.609728

Name: 20240913_Wu_sample_036, Date: 14-Sep-2024, Time: 03:21:45, ID: , Description:

guotang

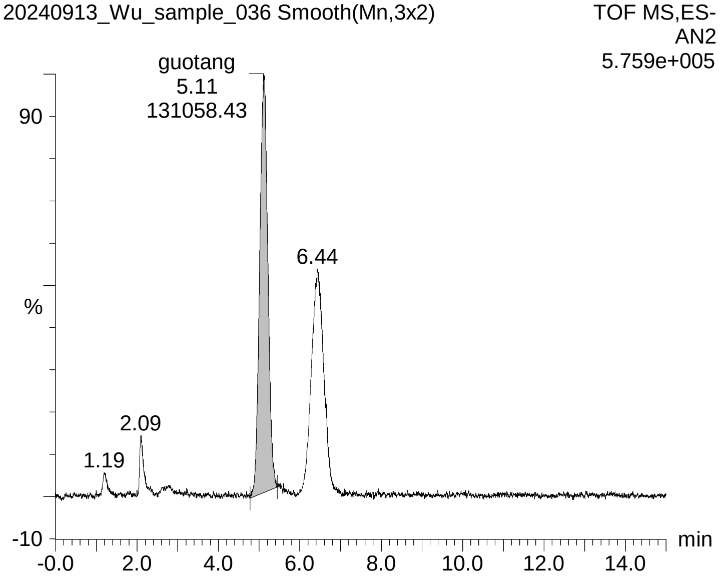

putaotang

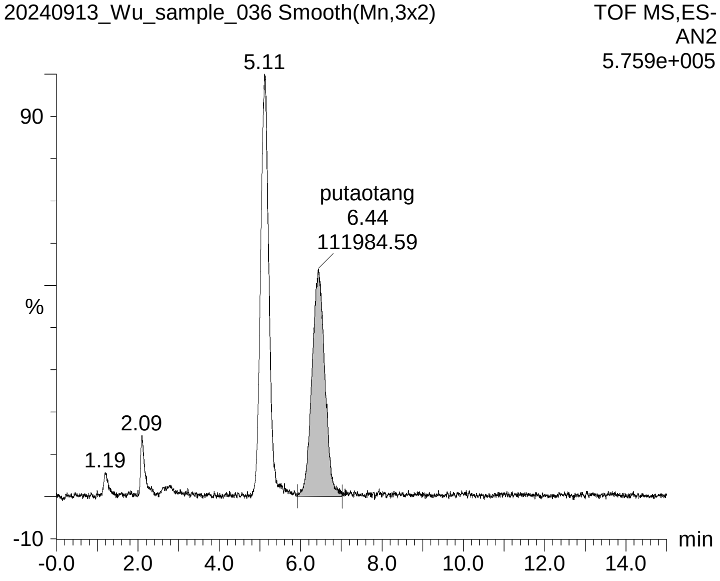

	#	Name	Sample Text	RT	Area	Std. Conc	Conc.
1	1	guotang		5.11	131058.430		88.910211
2	2	putaotang		6.44	111984.586		80.114833

Name: 20240913_Wu_sample_037, Date: 14-Sep-2024, Time: 03:37:44, ID: , Description:

guotang

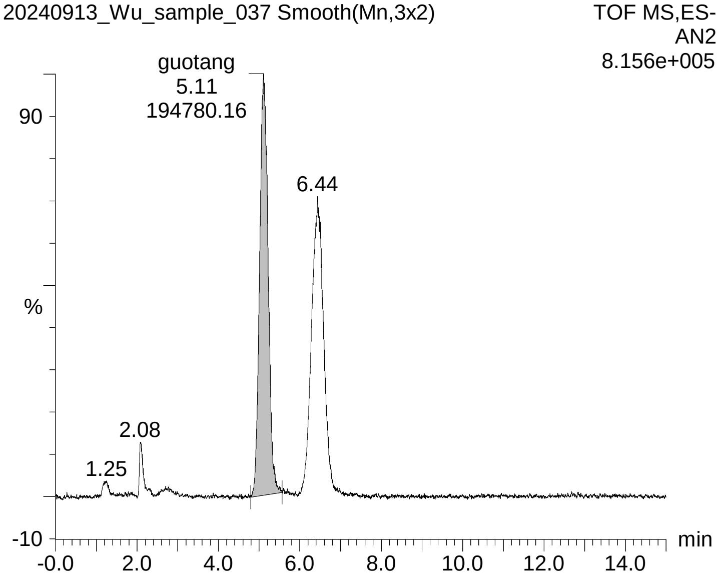

putaotang

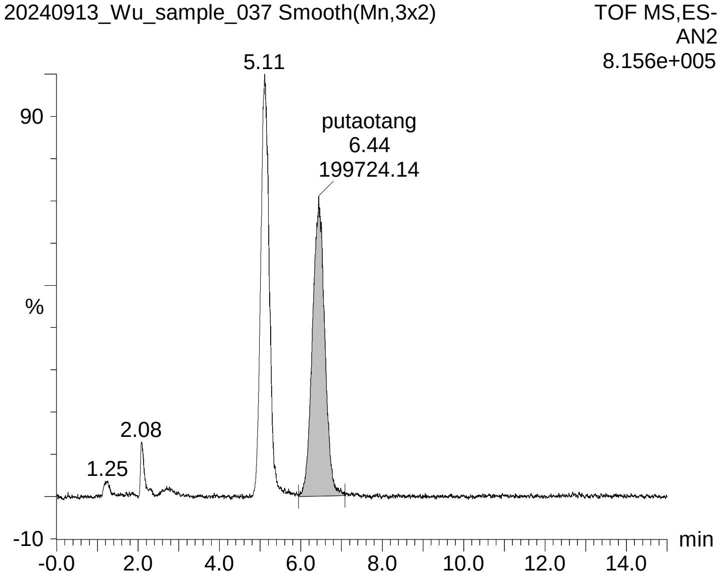

	#	Name	Sample Text	RT	Area	Std. Conc	Conc.
1	1	guotang		5.11	194780.156		125.172260
2	2	putaotang		6.44	199724.141		131.465728

Name: 20240913_Wu_sample_038, Date: 14-Sep-2024, Time: 03:53:45, ID: , Description:

guotang

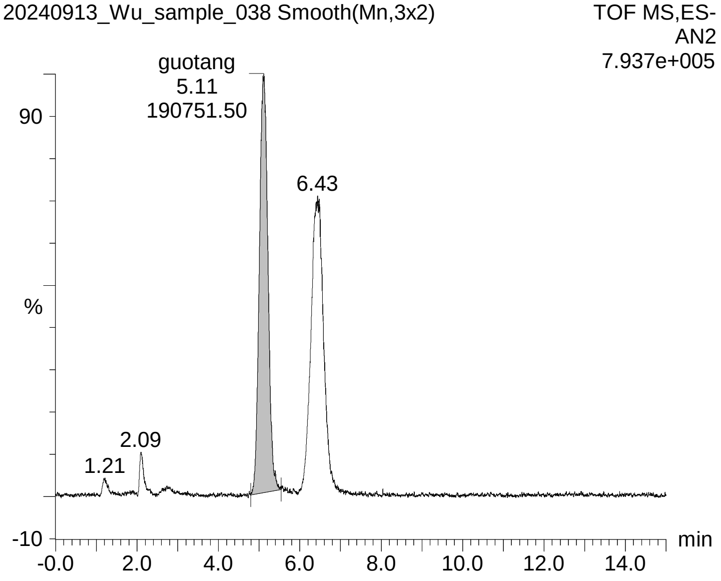

putaotang

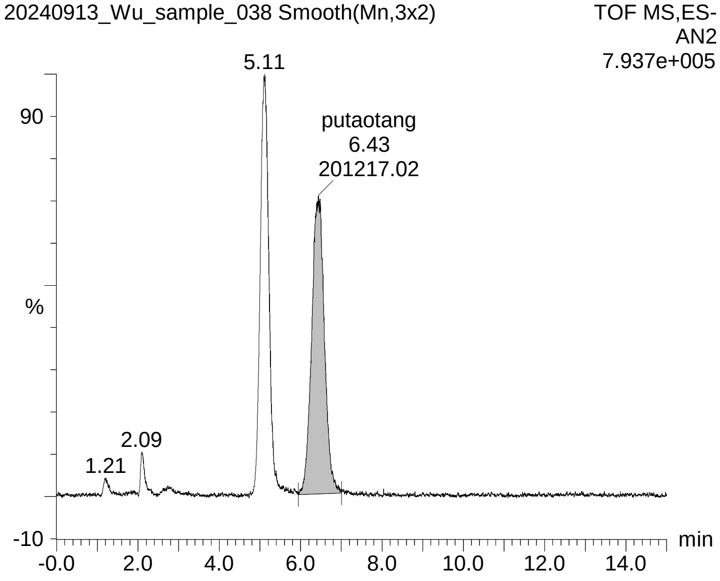

	#	Name	Sample Text	RT	Area	Std. Conc	Conc.
1	1	guotang		5.11	190751.500		122.879678
2	2	putaotang		6.43	201217.016		132.339456

project_wangzhonghua_BeiMu
Dataset: Untitled
Last Altered: Sunday, September 29, 2024 09:54:36 China Standard Time
Printed: Sunday, September 29, 2024 09:59:41 China Standard Time

Name: 20240913_Wu_sample_039, Date: 14-Sep-2024, Time: 04:09:47, ID: , Description:

guotang

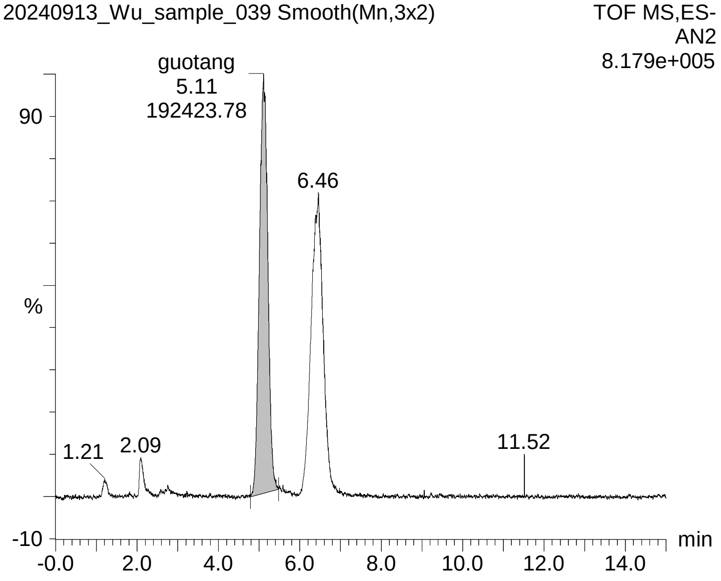

putaotang

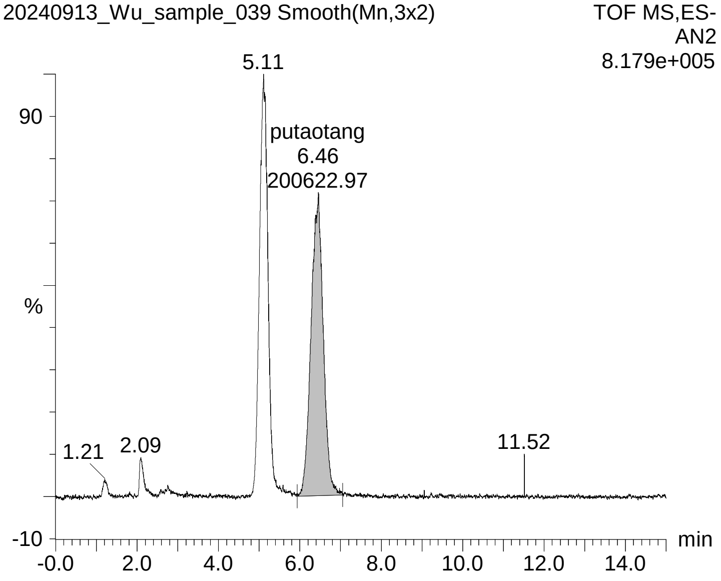

	#	Name	Sample Text	RT	Area	Std. Conc	Conc.
1	1	guotang		5.11	192423.781		123.831321
2	2	putaotang		6.46	200622.969		131.991781

Name: 20240913_Wu_sample_040, Date: 14-Sep-2024, Time: 04:25:49, ID: , Description:

guotang

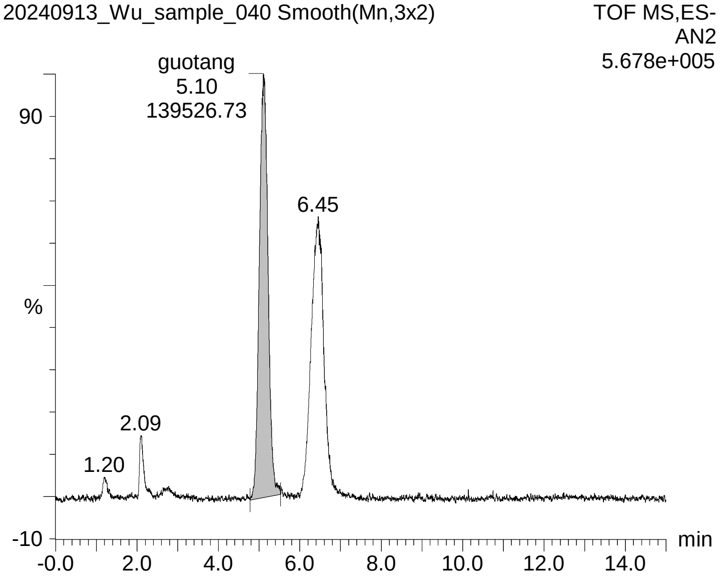

putaotang

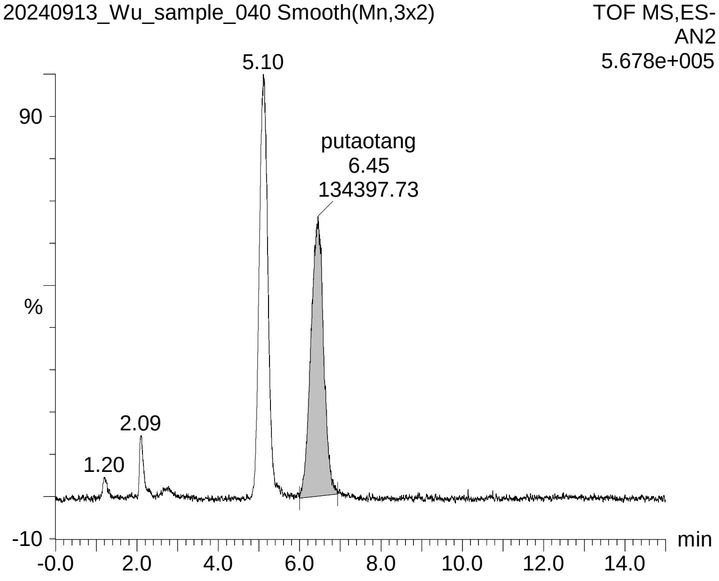

	#	Name	Sample Text	RT	Area	Std. Conc	Conc.
1	1	guotang		5.10	139526.734		93.729259
2	2	putaotang		6.45	134397.734		93.232465

project_wangzhonghua_BeiMu
Dataset: Untitled
Last Altered: Sunday, September 29, 2024 09:54:36 China Standard Time
Printed: Sunday, September 29, 2024 09:59:41 China Standard Time

Name: 20240913_Wu_sample_041, Date: 14-Sep-2024, Time: 04:41:50, ID: , Description:

guotang

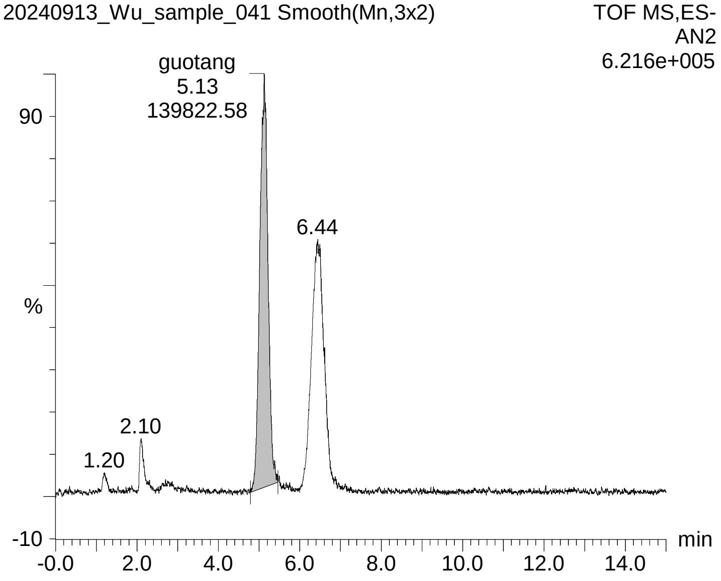

putaotang

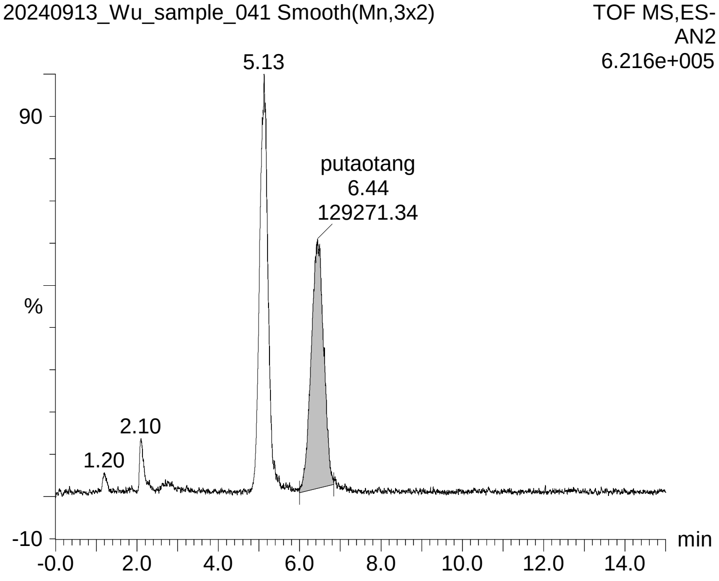

	#	Name	Sample Text	RT	Area	Std. Conc	Conc.
1	1	guotang		5.13	139822.578		93.897615
2	2	putaotang		6.44	129271.344		90.232168

project_wangzhonghua_BeiMu
Dataset: Untitled
Last Altered: Sunday, September 29, 2024 09:54:36 China Standard Time
Printed: Sunday, September 29, 2024 09:59:41 China Standard Time

Name: 20240913_Wu_sample_042, Date: 14-Sep-2024, Time: 04:57:52, ID: , Description:

guotang

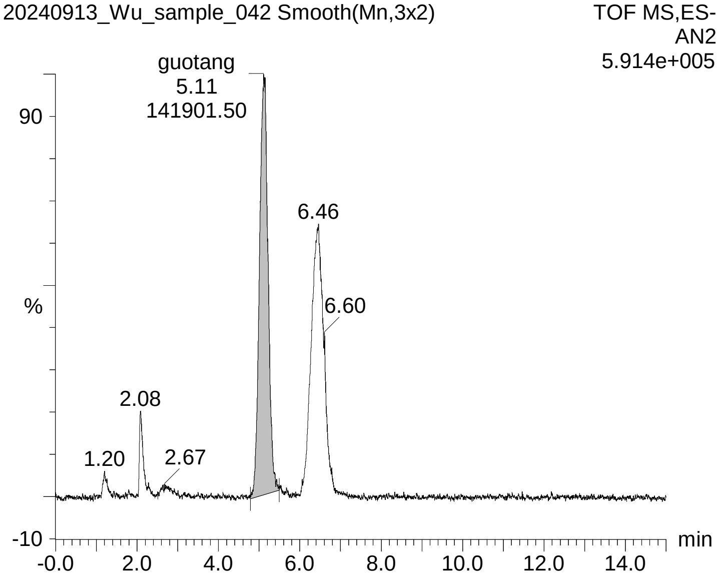

putaotang

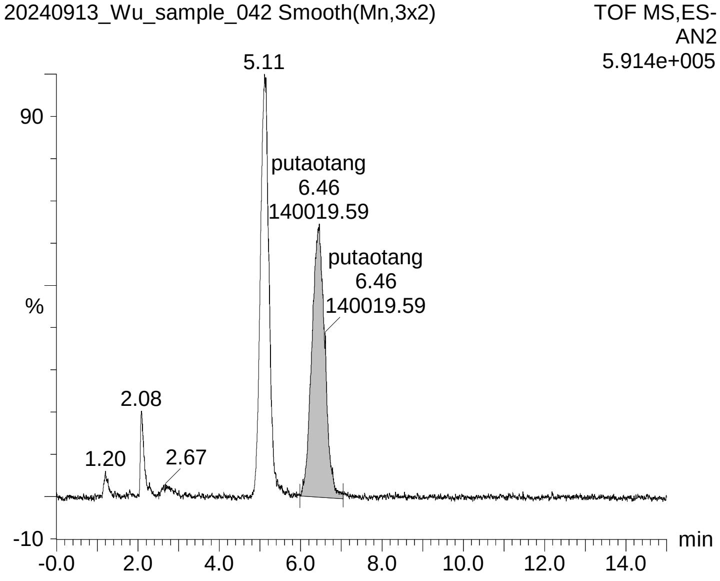

	#	Name	Sample Text	RT	Area	Std. Conc	Conc.
1	1	guotang		5.11	141901.500		95.080665
2	2	putaotang		6.46	140019.594		96.522743

project_wangzhonghua_BeiMu
Dataset: Untitled
Last Altered: Sunday, September 29, 2024 09:54:36 China Standard Time
Printed: Sunday, September 29, 2024 09:59:41 China Standard Time

Name: 20240913_Wu_sample_043, Date: 14-Sep-2024, Time: 05:13:54, ID: , Description:

guotang

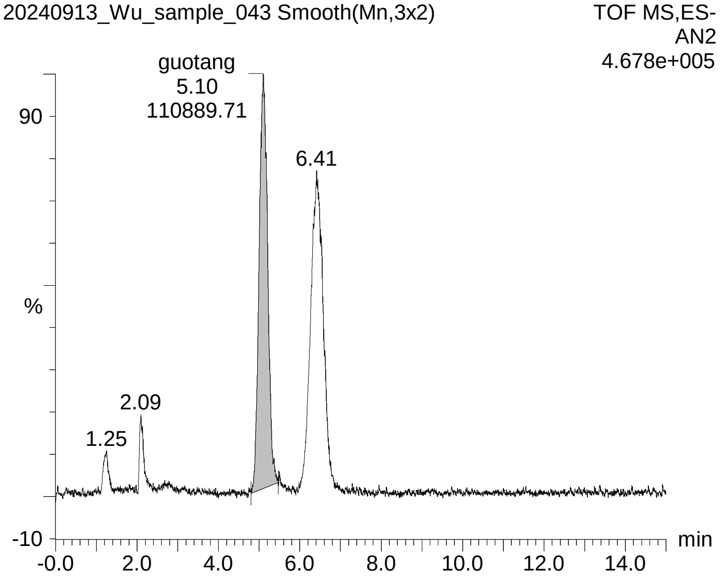

putaotang

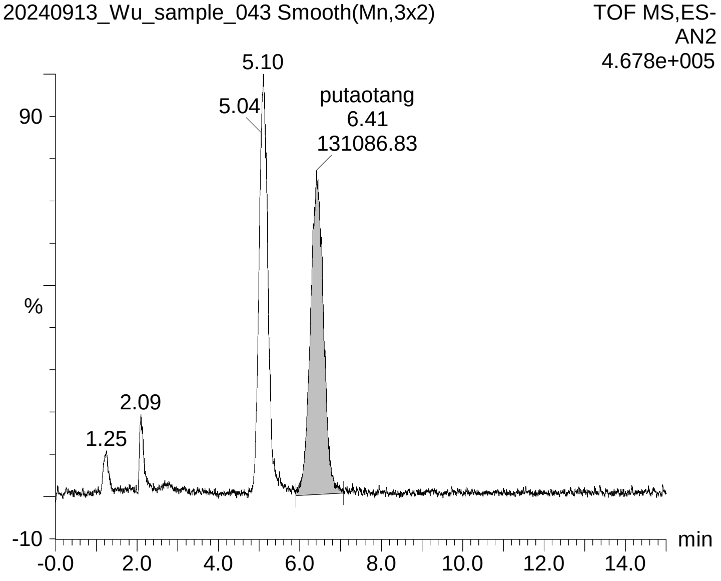

	#	Name	Sample Text	RT	Area	Std. Conc	Conc.
1	1	guotang		5.10	110889.711		77.432822
2	2	putaotang		6.41	131086.828		91.294708

project_wangzhonghua_BeiMu
Dataset: Untitled
Last Altered: Sunday, September 29, 2024 09:54:36 China Standard Time
Printed: Sunday, September 29, 2024 09:59:41 China Standard Time

Name: 20240913_Wu_sample_044, Date: 14-Sep-2024, Time: 05:29:55, ID: , Description:

guotang

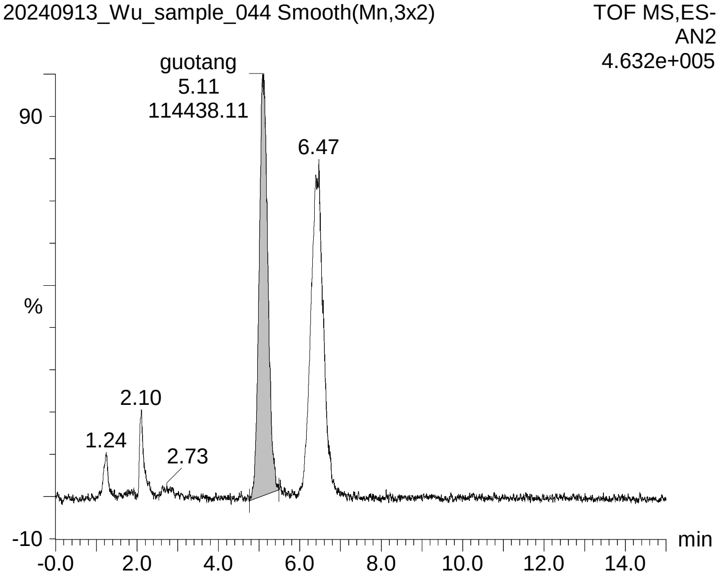

putaotang

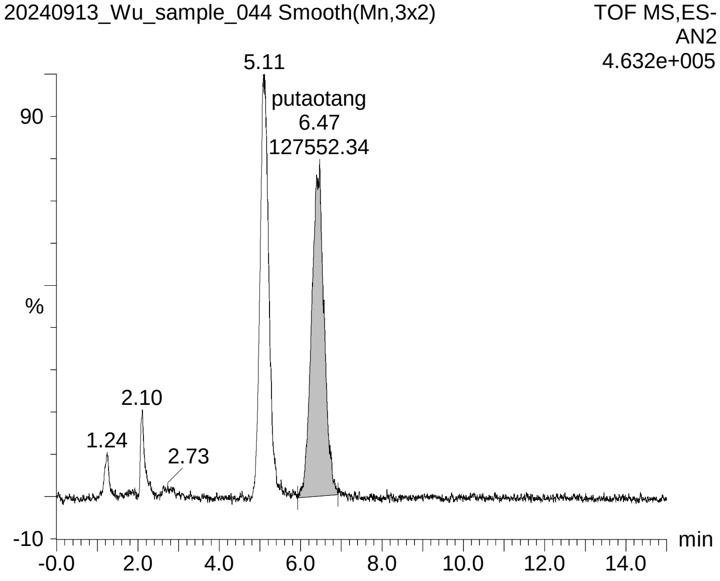

	#	Name	Sample Text	RT	Area	Std. Conc	Conc.
1	1	guotang		5.11	114438.109		79.452104
2	2	putaotang		6.47	127552.344		89.226098

Name: 20240913_Wu_sample_045, Date: 14-Sep-2024, Time: 05:45:57, ID: , Description:

guotang

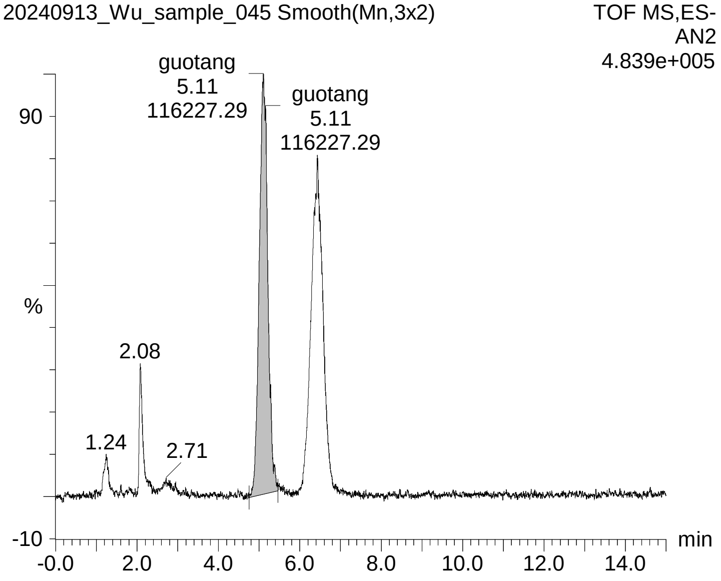

putaotang

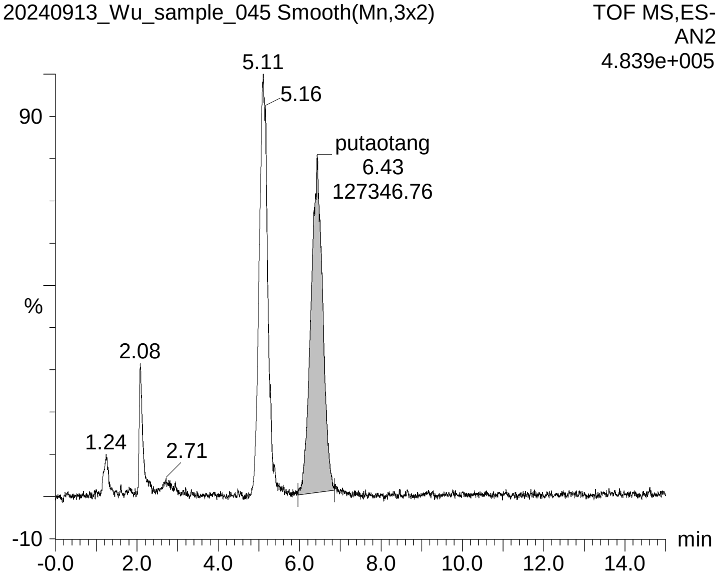

	#	Name	Sample Text	RT	Area	Std. Conc	Conc.
1	1	guotang		5.11	116227.289		80.470271
2	2	putaotang		6.43	127346.758		89.105775

Name: 20240913_Wu_sample_046, Date: 14-Sep-2024, Time: 06:01:59, ID: , Description:

guotang

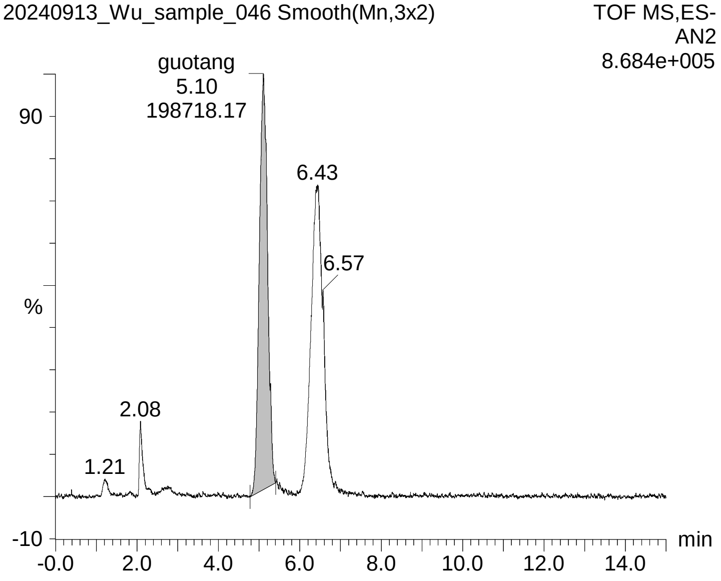

putaotang

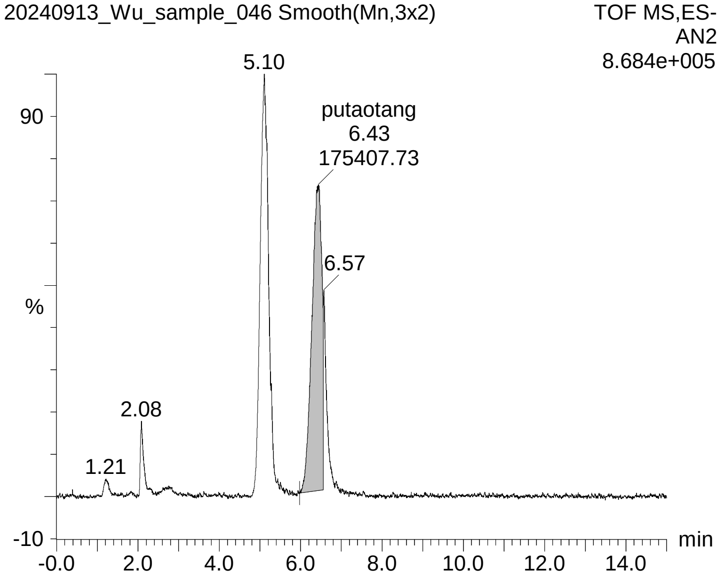

	#	Name	Sample Text	RT	Area	Std. Conc	Conc.
1	1	guotang		5.10	198718.172		127.413263
2	2	putaotang		6.43	175407.734		117.234185

project_wangzhonghua_BeiMu
Dataset: Untitled
Last Altered: Sunday, September 29, 2024 09:54:36 China Standard Time
Printed: Sunday, September 29, 2024 09:59:41 China Standard Time

Name: 20240913_Wu_sample_047, Date: 14-Sep-2024, Time: 06:18:01, ID: , Description:

guotang

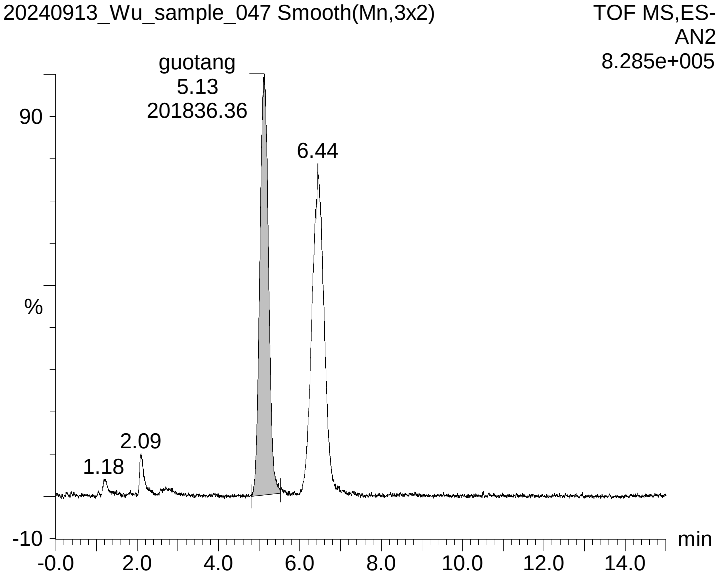

putaotang

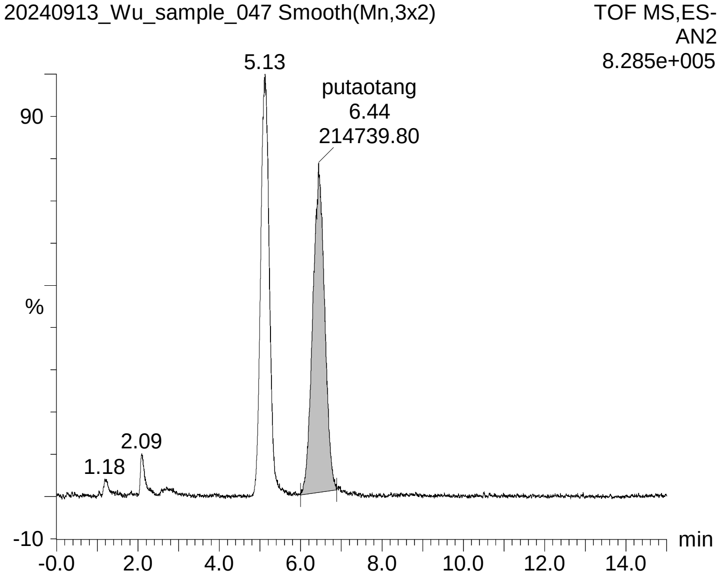

	#	Name	Sample Text	RT	Area	Std. Conc	Conc.
1	1	guotang		5.13	201836.359		129.187726
2	2	putaotang		6.44	214739.797		140.253867

Name: 20240913_Wu_sample_048, Date: 14-Sep-2024, Time: 06:34:02, ID: , Description:

guotang

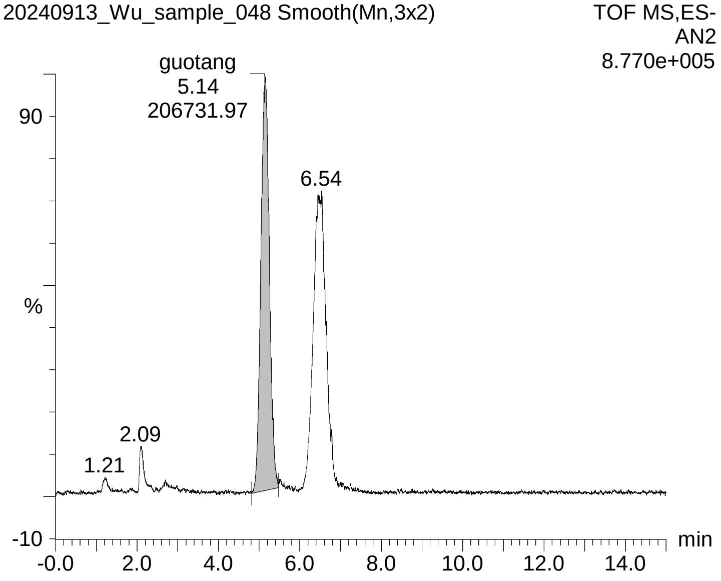

putaotang

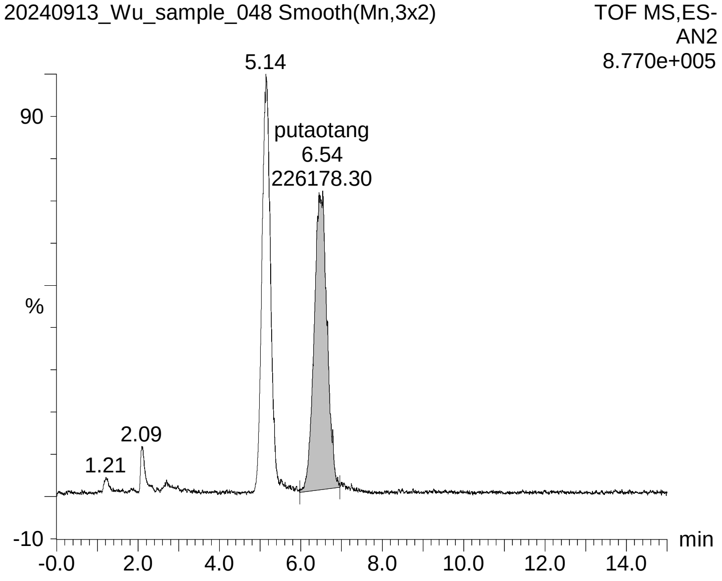

	#	Name	Sample Text	RT	Area	Std. Conc	Conc.
1	1	guotang		5.14	206731.969		131.973665
2	2	putaotang		6.54	226178.297		146.948421

project_wangzhonghua_BeiMu
Dataset: Untitled
Last Altered: Sunday, September 29, 2024 09:54:36 China Standard Time
Printed: Sunday, September 29, 2024 09:59:41 China Standard Time

Name: 20240913_Wu_sample_049, Date: 14-Sep-2024, Time: 06:50:04, ID: , Description:

guotang

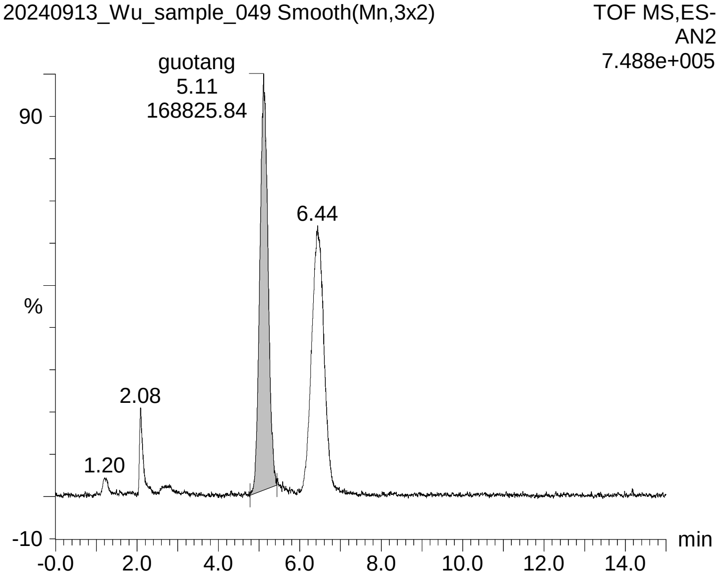

putaotang

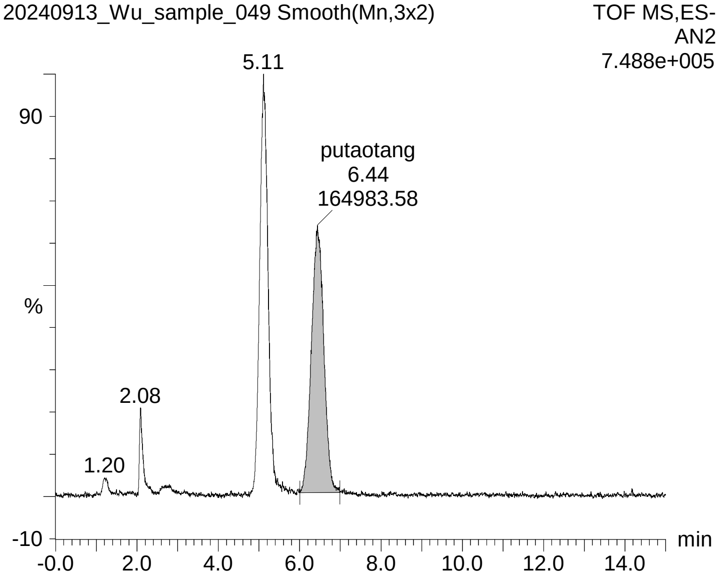

	#	Name	Sample Text	RT	Area	Std. Conc	Conc.
1	1	guotang		5.11	168825.844		110.402470
2	2	putaotang		6.44	164983.578		111.133291

project_wangzhonghua_BeiMu
Dataset: Untitled
Last Altered: Sunday, September 29, 2024 09:54:36 China Standard Time
Printed: Sunday, September 29, 2024 09:59:41 China Standard Time

Name: 20240913_Wu_sample_050, Date: 14-Sep-2024, Time: 07:06:06, ID: , Description:

guotang

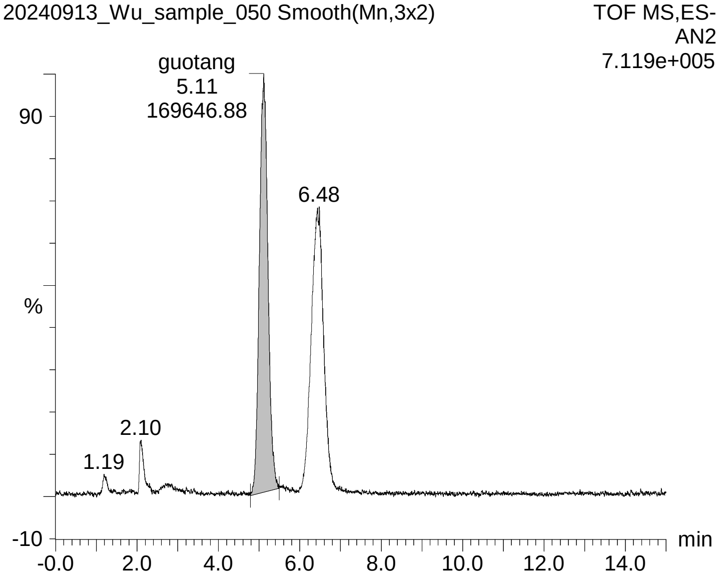

putaotang

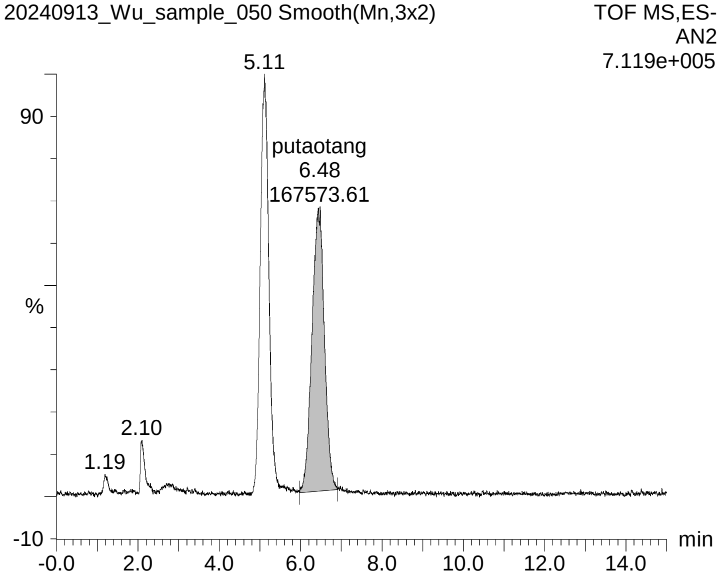

	#	Name	Sample Text	RT	Area	Std. Conc	Conc.
1	1	guotang		5.11	169646.875		110.869693
2	2	putaotang		6.48	167573.609		112.649145

Name: 20240913_Wu_sample_051, Date: 14-Sep-2024, Time: 07:22:08, ID: , Description:

guotang

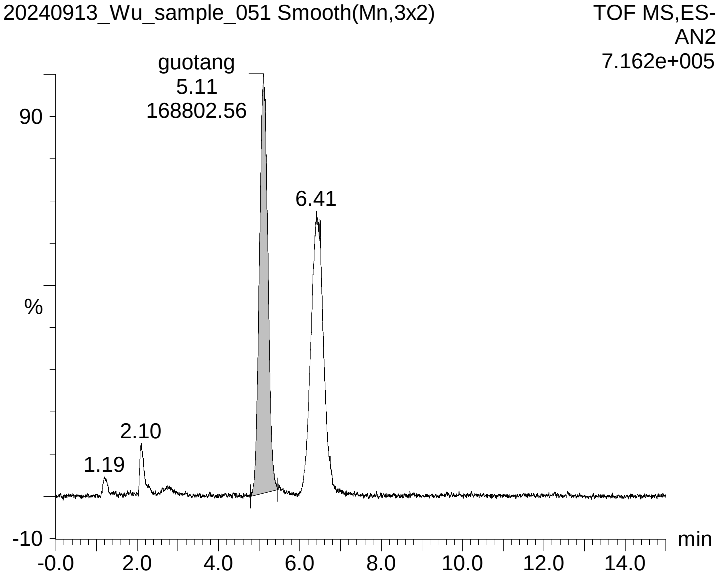

putaotang

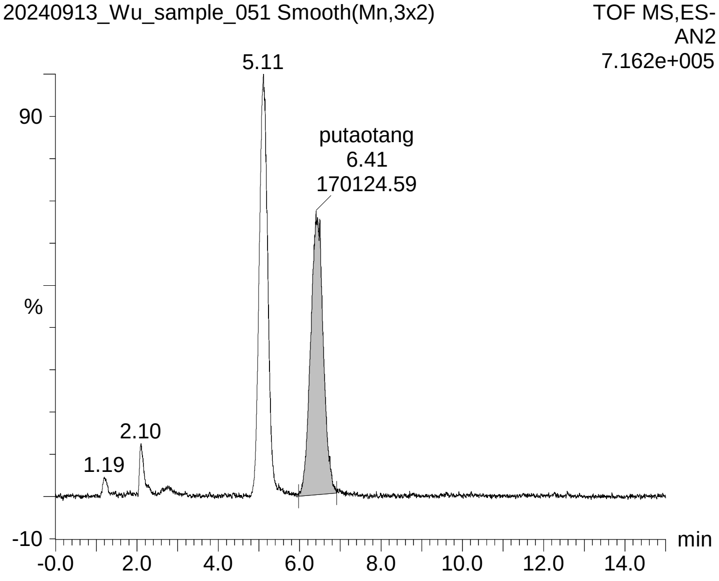

	#	Name	Sample Text	RT	Area	Std. Conc	Conc.
1	1	guotang		5.11	168802.563		110.389222
2	2	putaotang		6.41	170124.594		114.142148

project_wangzhonghua_BeiMu
Dataset: Untitled
Last Altered: Sunday, September 29, 2024 09:54:36 China Standard Time
Printed: Sunday, September 29, 2024 09:59:41 China Standard Time

Name: 20240913_Wu_sample_052, Date: 14-Sep-2024, Time: 07:38:09, ID: , Description:

guotang

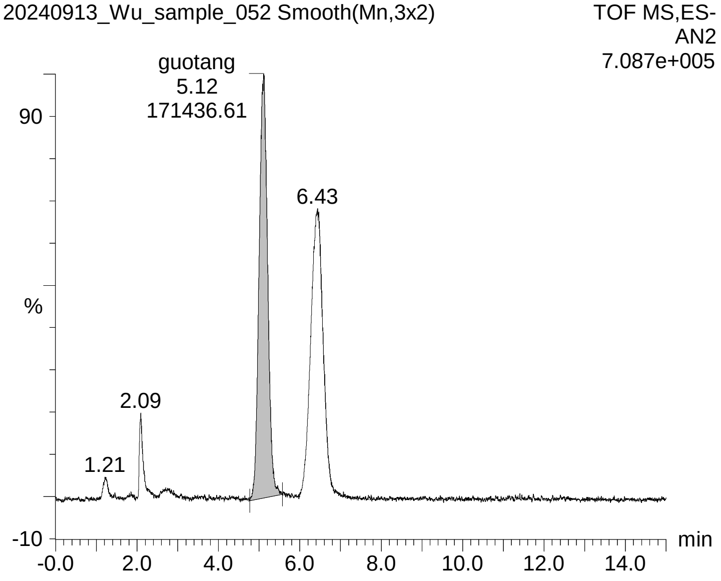

putaotang

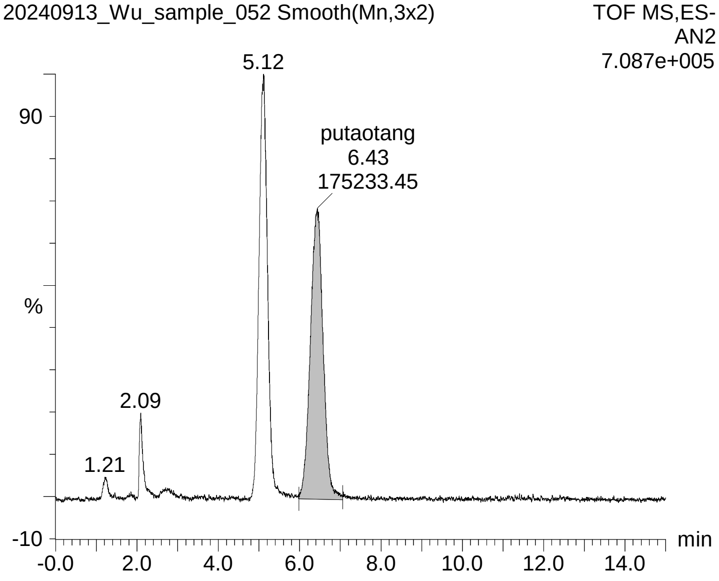

	#	Name	Sample Text	RT	Area	Std. Conc	Conc.
1	1	guotang		5.12	171436.609		111.888175
2	2	putaotang		6.43	175233.453		117.132184

Name: 20240913_Wu_sample_053, Date: 14-Sep-2024, Time: 07:54:11, ID: , Description:

guotang

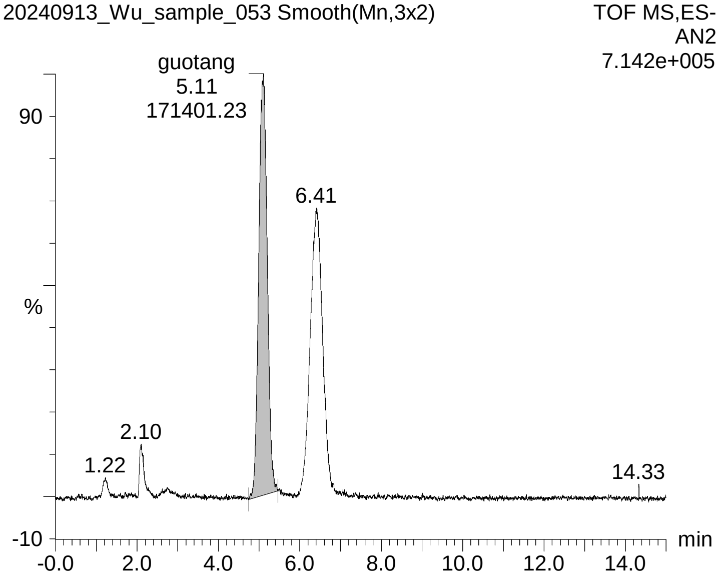

putaotang

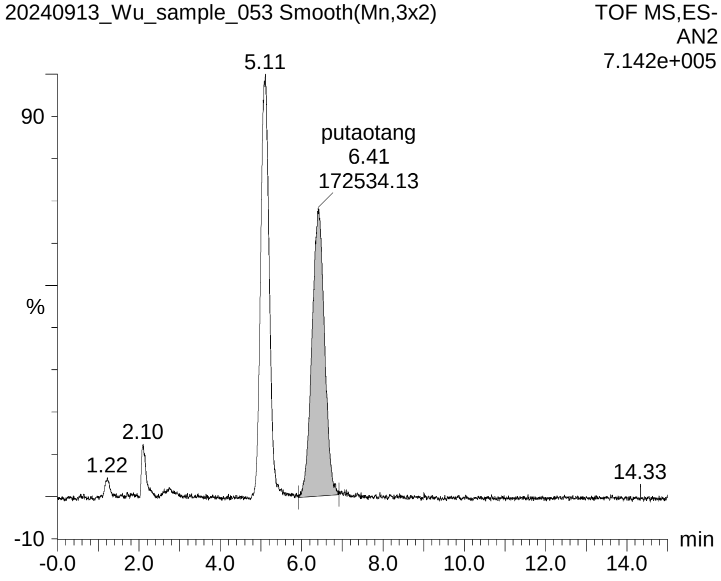

	#	Name	Sample Text	RT	Area	Std. Conc	Conc.
1	1	guotang		5.11	171401.234		111.868044
2	2	putaotang		6.41	172534.125		115.552362

project_wangzhonghua_BeiMu
Dataset: Untitled
Last Altered: Sunday, September 29, 2024 09:54:36 China Standard Time
Printed: Sunday, September 29, 2024 09:59:41 China Standard Time

Name: 20240913_Wu_sample_054, Date: 14-Sep-2024, Time: 08:10:13, ID: , Description:

guotang

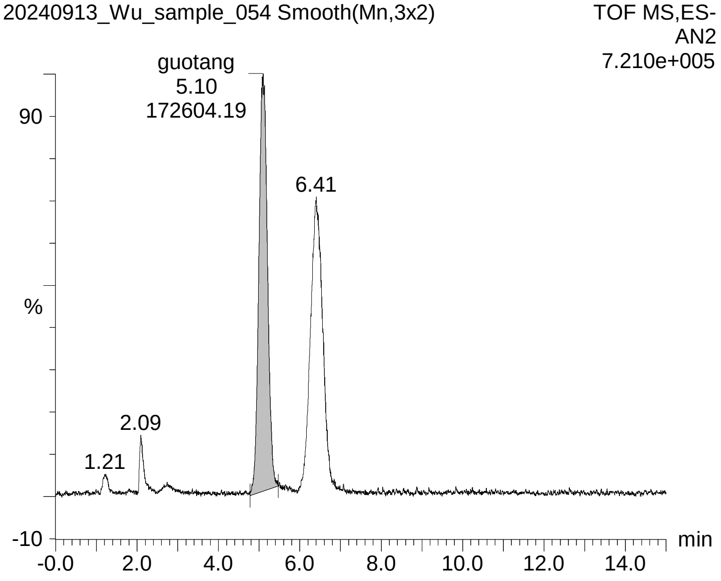

putaotang

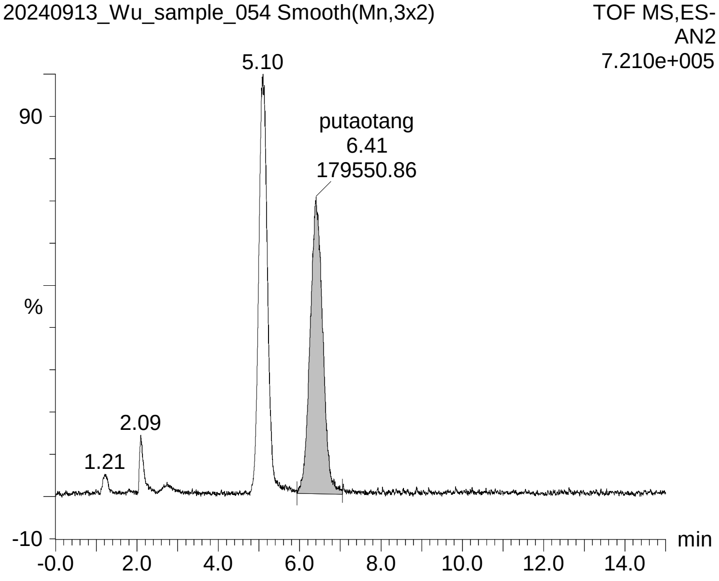

	#	Name	Sample Text	RT	Area	Std. Conc	Conc.
1	1	guotang		5.10	172604.188		112.552608
2	2	putaotang		6.41	179550.859		119.659011

project_wangzhonghua_BeiMu
Dataset: Untitled
Last Altered: Sunday, September 29, 2024 09:54:36 China Standard Time
Printed: Sunday, September 29, 2024 09:59:41 China Standard Time

Name: 20240913_Wu_sample_055, Date: 14-Sep-2024, Time: 08:26:15, ID: , Description:

guotang

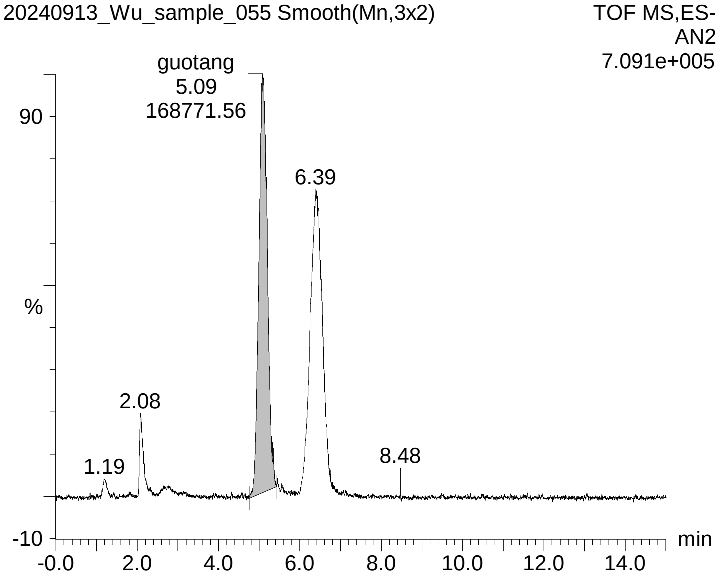

putaotang

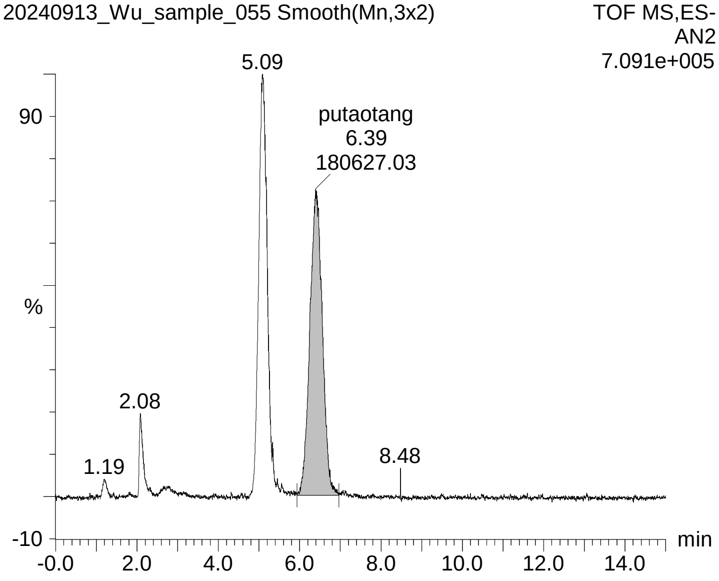

	#	Name	Sample Text	RT	Area	Std. Conc	Conc.
1	1	guotang		5.09	168771.563		110.371580
2	2	putaotang		6.39	180627.031		120.288857

project_wangzhonghua_BeiMu
Dataset: Untitled
Last Altered: Sunday, September 29, 2024 09:54:36 China Standard Time
Printed: Sunday, September 29, 2024 09:59:41 China Standard Time

Name: 20240913_Wu_sample_056, Date: 14-Sep-2024, Time: 08:42:17, ID: , Description:

guotang

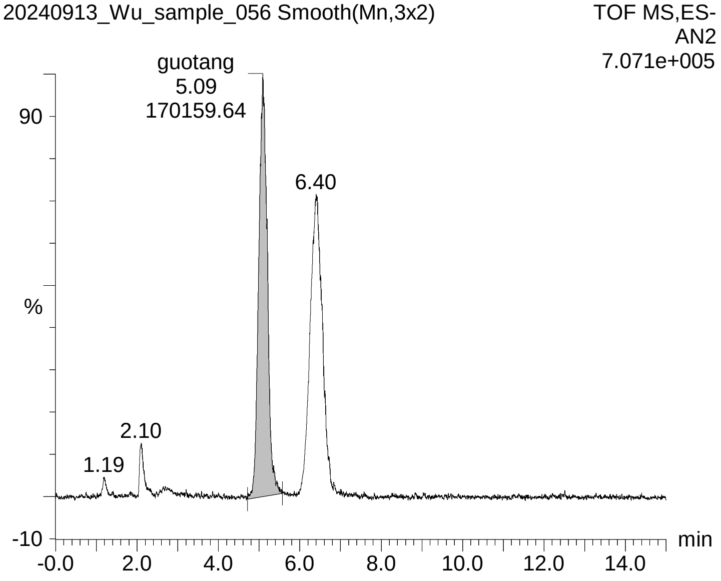

putaotang

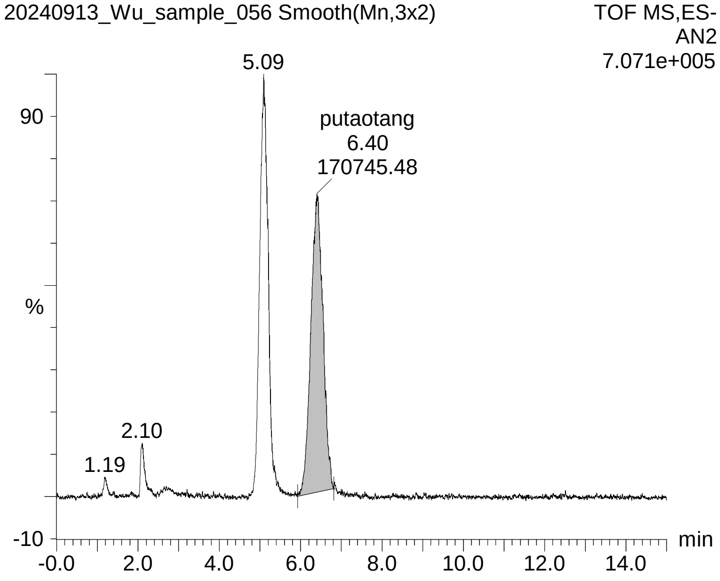

	#	Name	Sample Text	RT	Area	Std. Conc	Conc.
1	1	guotang		5.09	170159.641		111.161492
2	2	putaotang		6.40	170745.484		114.505533

project_wangzhonghua_BeiMu
Dataset: Untitled
Last Altered: Sunday, September 29, 2024 09:54:36 China Standard Time
Printed: Sunday, September 29, 2024 09:59:41 China Standard Time

Name: 20240913_Wu_sample_057, Date: 14-Sep-2024, Time: 08:58:20, ID: , Description:

guotang

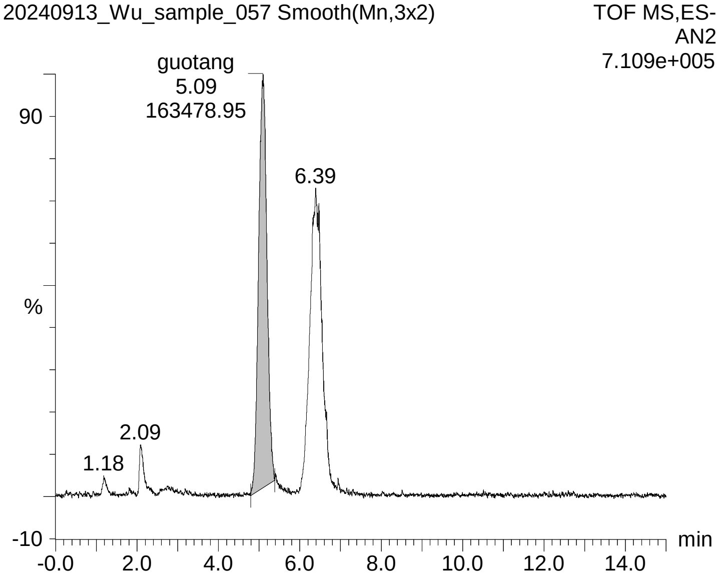

putaotang

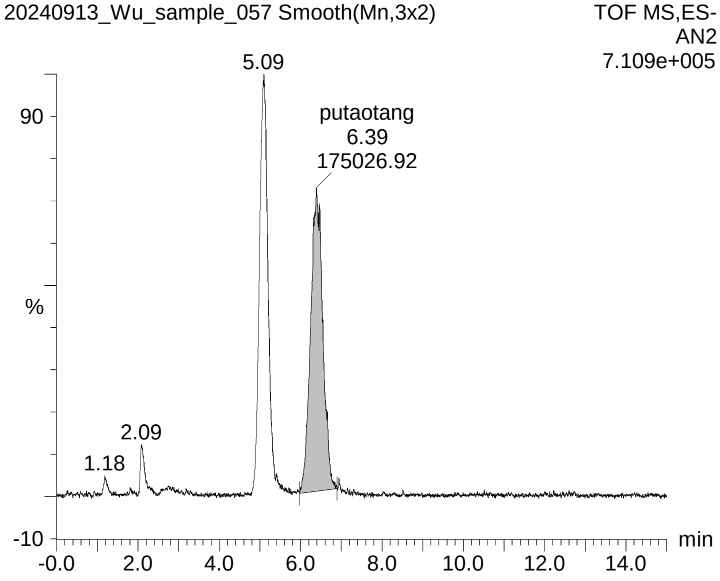

	#	Name	Sample Text	RT	Area	Std. Conc	Conc.
1	1	guotang		5.09	163478.953		107.359721
2	2	putaotang		6.39	175026.922		117.011309

project_wangzhonghua_BeiMu
Dataset: Untitled
Last Altered: Sunday, September 29, 2024 09:54:36 China Standard Time
Printed: Sunday, September 29, 2024 09:59:41 China Standard Time

Name: 20240913_Wu_sample_058, Date: 14-Sep-2024, Time: 09:14:22, ID: , Description:

guotang

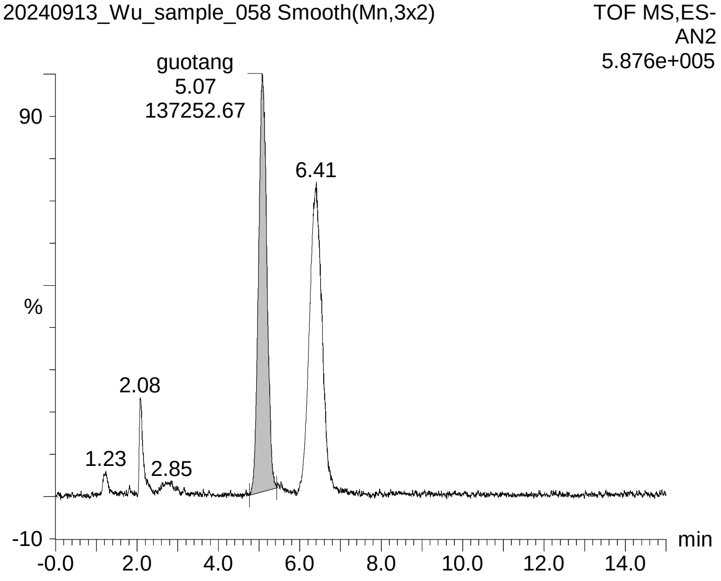

putaotang

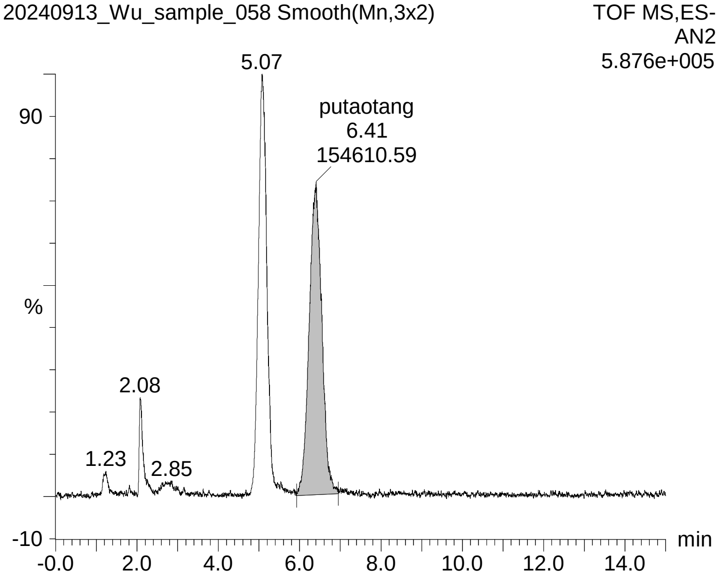

	#	Name	Sample Text	RT	Area	Std. Conc	Conc.
1	1	guotang		5.07	137252.672		92.435161
2	2	putaotang		6.41	154610.594		105.062346

Name: 20240913_Wu_sample_059, Date: 14-Sep-2024, Time: 09:30:23, ID: , Description:

guotang

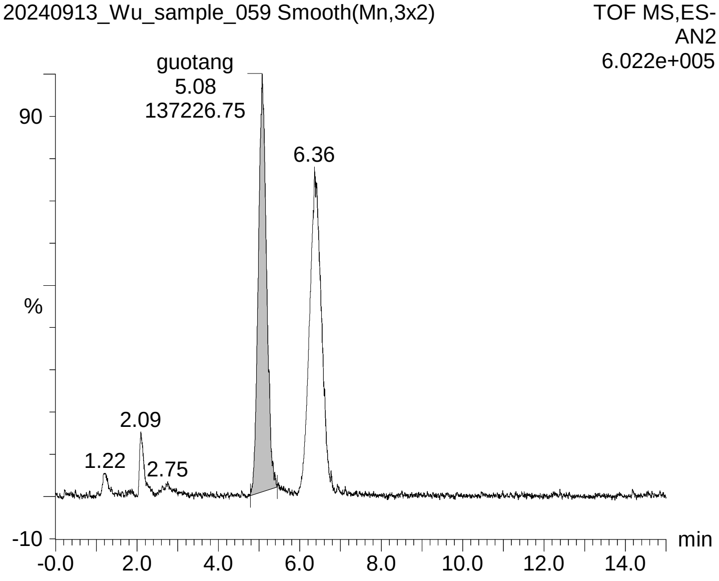

putaotang

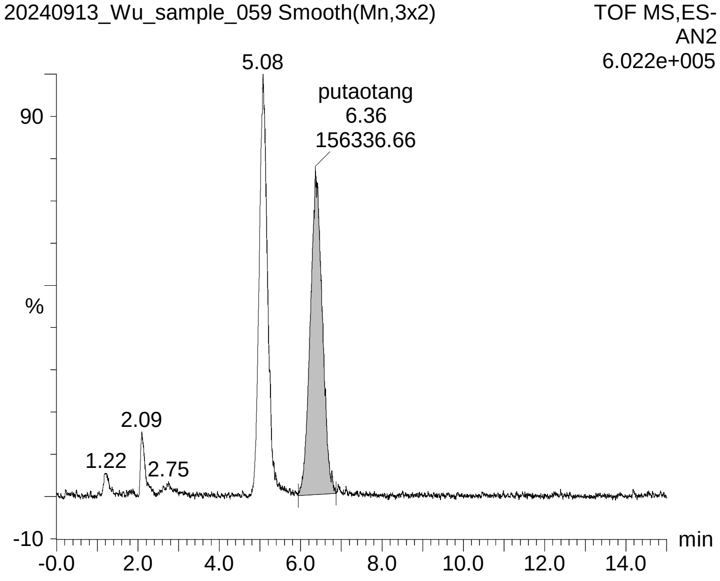

	#	Name	Sample Text	RT	Area	Std. Conc	Conc.
1	1	guotang		5.08	137226.750		92.420410
2	2	putaotang		6.36	156336.656		106.072549

Name: 20240913_Wu_sample_060, Date: 14-Sep-2024, Time: 09:46:25, ID: , Description:

guotang

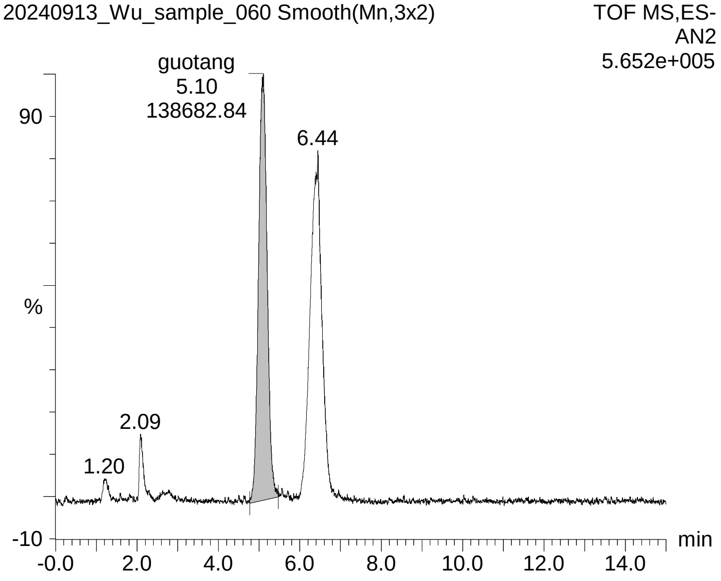

putaotang

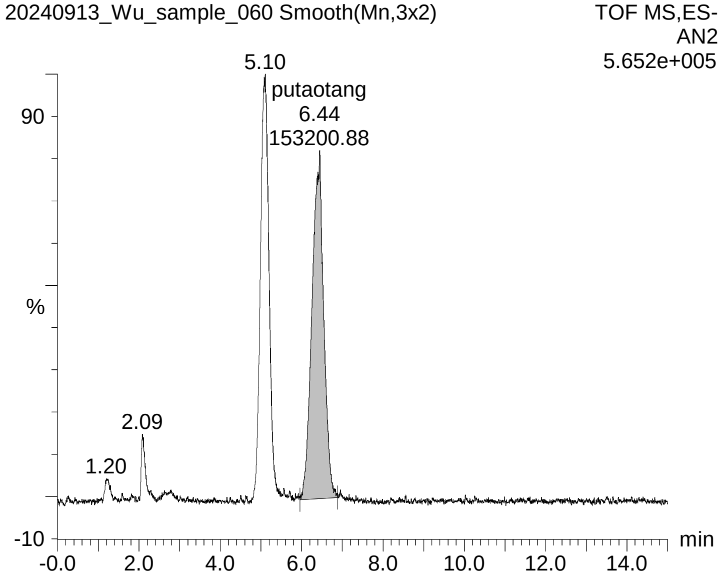

	#	Name	Sample Text	RT	Area	Std. Conc	Conc.
1	1	guotang		5.10	138682.844		93.249028
2	2	putaotang		6.44	153200.875		104.237286

project_wangzhonghua_BeiMu
Dataset: Untitled
Last Altered: Sunday, September 29, 2024 09:54:36 China Standard Time
Printed: Sunday, September 29, 2024 09:59:41 China Standard Time

Name: 20240913_Wu_sample_061, Date: 14-Sep-2024, Time: 10:02:27, ID: , Description:

guotang

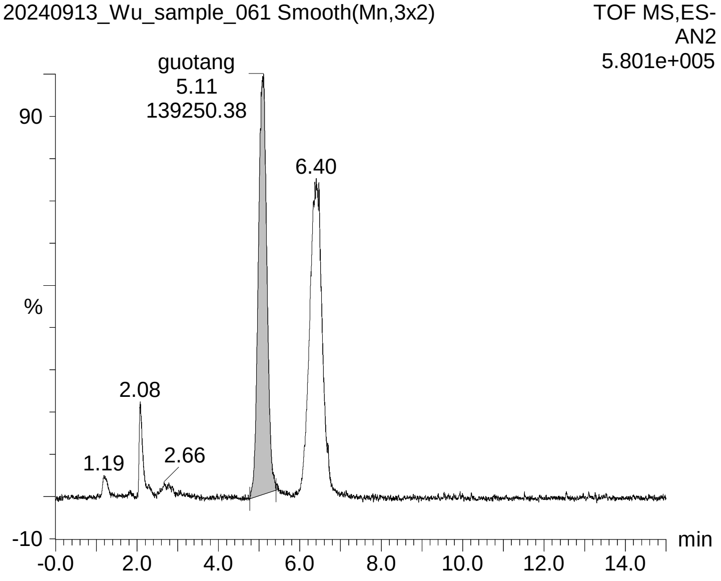

putaotang

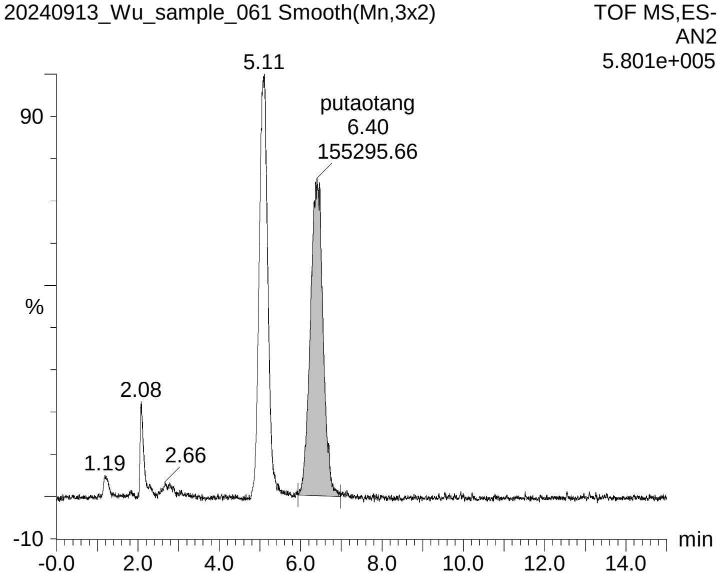

	#	Name	Sample Text	RT	Area	Std. Conc	Conc.
1	1	guotang		5.11	139250.375		93.571992
2	2	putaotang		6.40	155295.656		105.463289

project_wangzhonghua_BeiMu
Dataset: Untitled
Last Altered: Sunday, September 29, 2024 09:54:36 China Standard Time
Printed: Sunday, September 29, 2024 09:59:41 China Standard Time

Name: 20240913_Wu_sample_062, Date: 14-Sep-2024, Time: 10:18:29, ID: , Description:

guotang

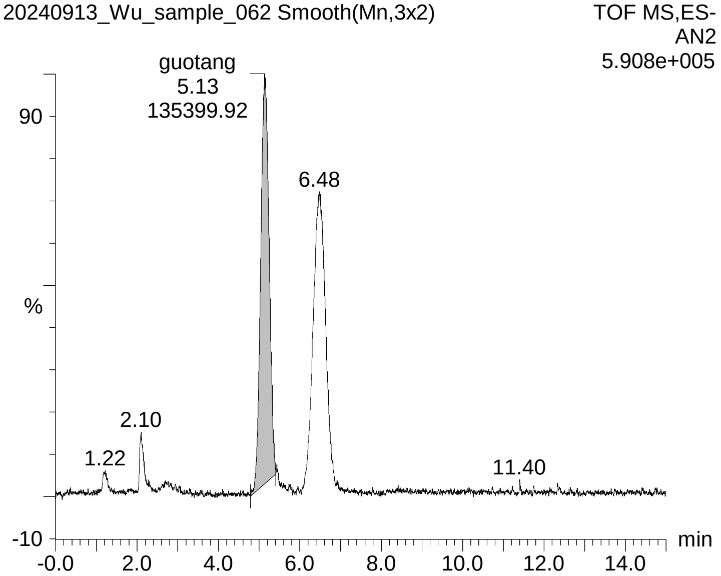

putaotang

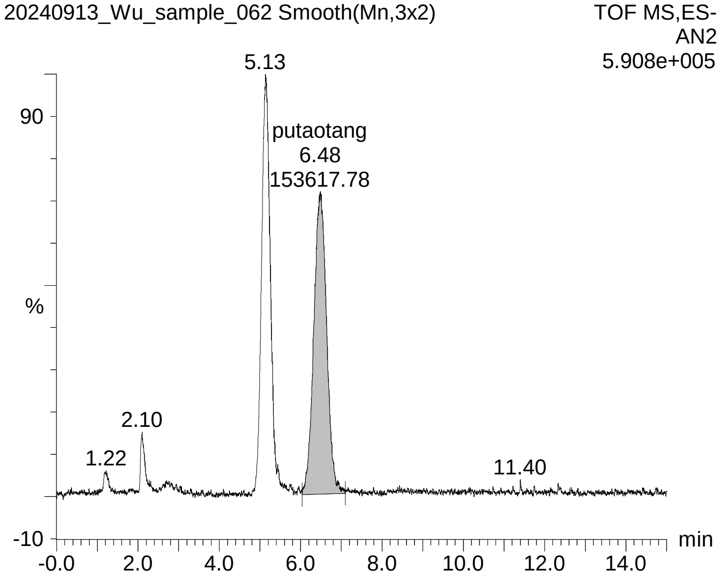

	#	Name	Sample Text	RT	Area	Std. Conc	Conc.
1	1	guotang		5.13	135399.922		91.380819
2	2	putaotang		6.48	153617.781		104.481287

Name: 20240913_Wu_sample_063, Date: 14-Sep-2024, Time: 10:34:30, ID: , Description:

guotang

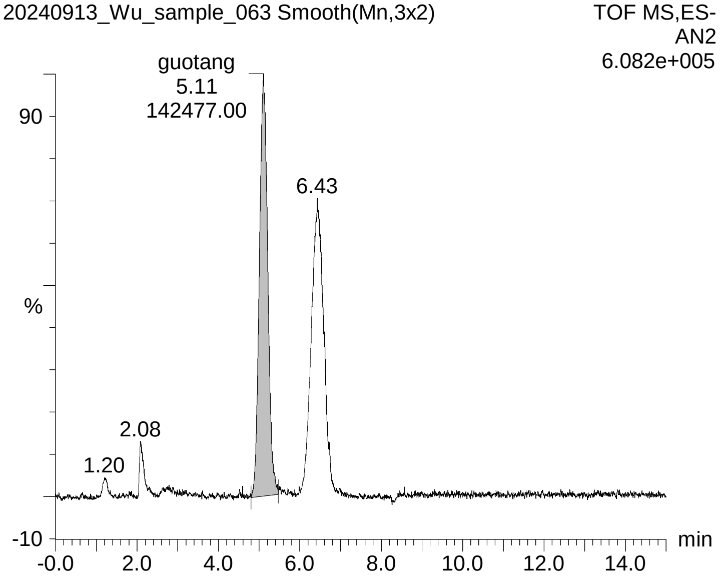

putaotang

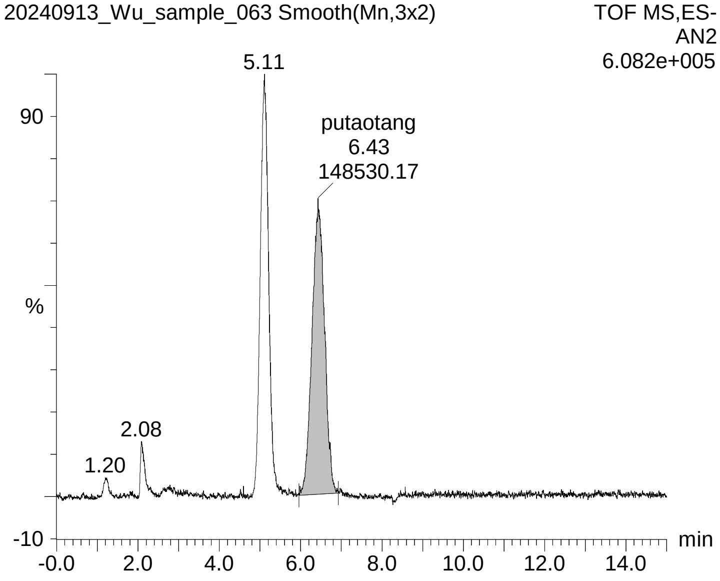

	#	Name	Sample Text	RT	Area	Std. Conc	Conc.
1	1	guotang		5.11	142477.000		95.408164
2	2	putaotang		6.43	148530.172		101.503687

project_wangzhonghua_BeiMu
Dataset: Untitled
Last Altered: Sunday, September 29, 2024 09:54:36 China Standard Time
Printed: Sunday, September 29, 2024 09:59:41 China Standard Time

Name: 20240913_Wu_sample_064, Date: 14-Sep-2024, Time: 10:50:33, ID: , Description:

guotang

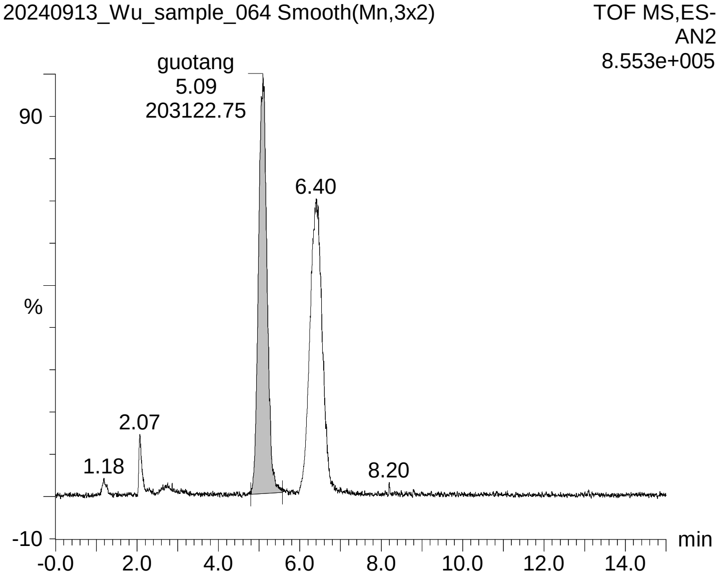

putaotang

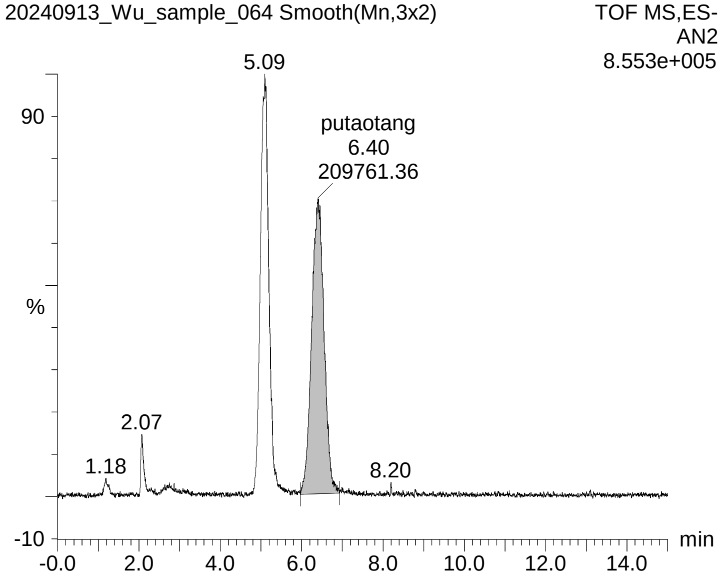

	#	Name	Sample Text	RT	Area	Std. Conc	Conc.
1	1	guotang		5.09	203122.750		129.919771
2	2	putaotang		6.40	209761.359		137.340161

project_wangzhonghua_BeiMu
Dataset: Untitled
Last Altered: Sunday, September 29, 2024 09:54:36 China Standard Time
Printed: Sunday, September 29, 2024 09:59:41 China Standard Time

Name: 20240913_Wu_sample_065, Date: 14-Sep-2024, Time: 11:06:35, ID: , Description:

guotang

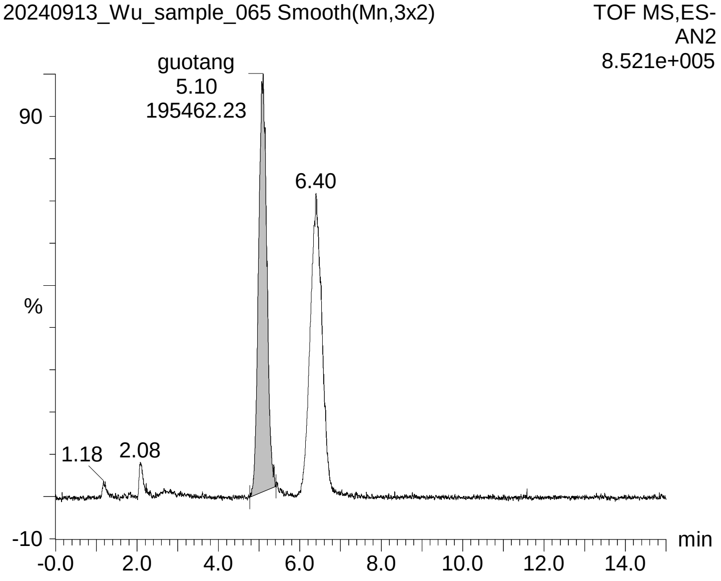

putaotang

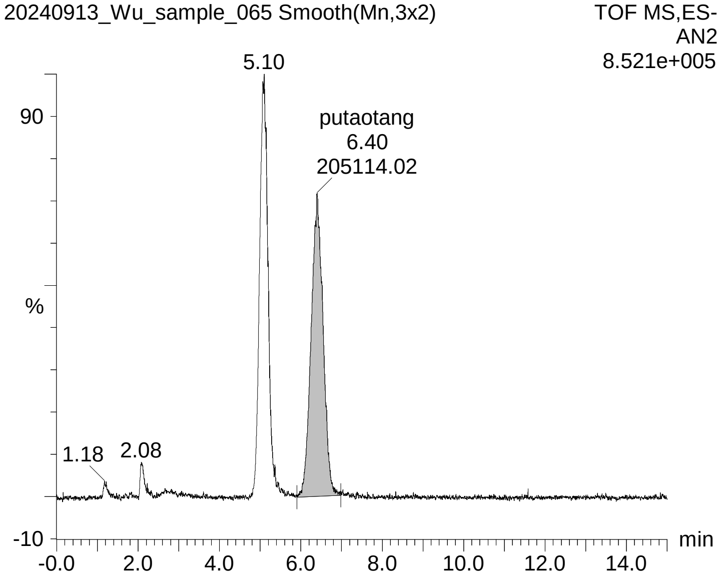

	#	Name	Sample Text	RT	Area	Std. Conc	Conc.
1	1	guotang		5.10	195462.234		125.560410
2	2	putaotang		6.40	205114.016		134.620234

project_wangzhonghua_BeiMu
Dataset: Untitled
Last Altered: Sunday, September 29, 2024 09:54:36 China Standard Time
Printed: Sunday, September 29, 2024 09:59:41 China Standard Time

Name: 20240913_Wu_sample_066, Date: 14-Sep-2024, Time: 11:22:37, ID: , Description:

guotang

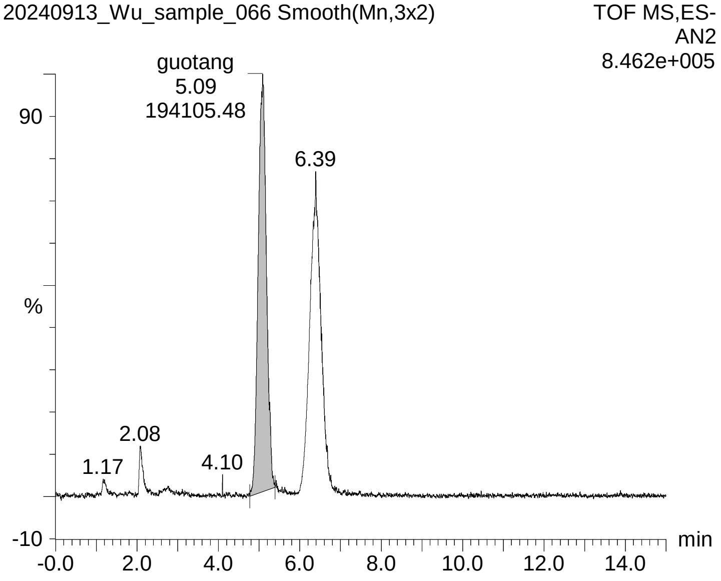

putaotang

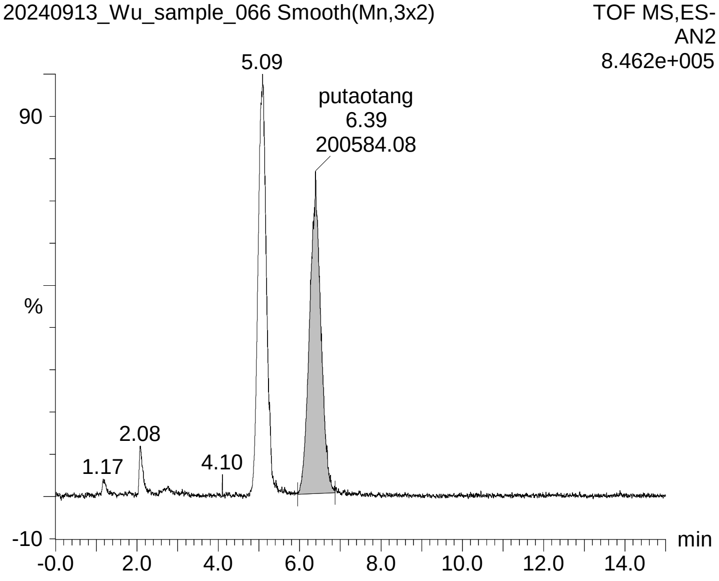

	#	Name	Sample Text	RT	Area	Std. Conc	Conc.
1	1	guotang		5.09	194105.484		124.788326
2	2	putaotang		6.39	200584.078		131.969019

Name: 20240913_Wu_sample_067, Date: 14-Sep-2024, Time: 11:38:39, ID: , Description:

guotang

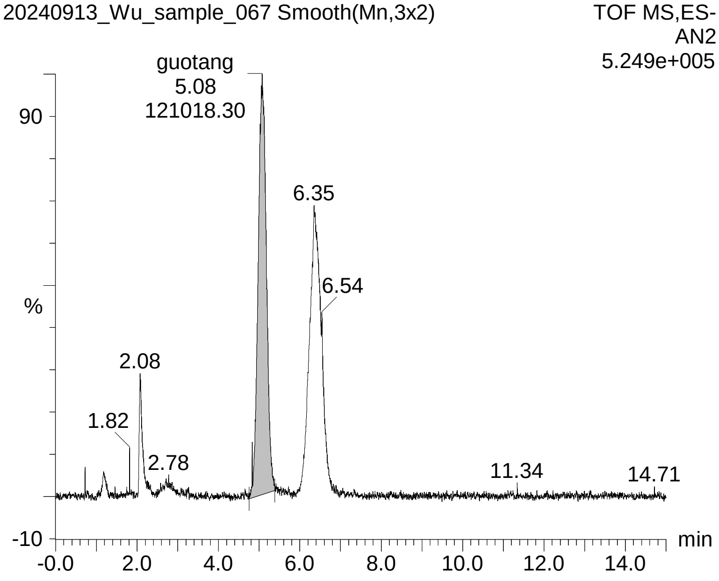

putaotang

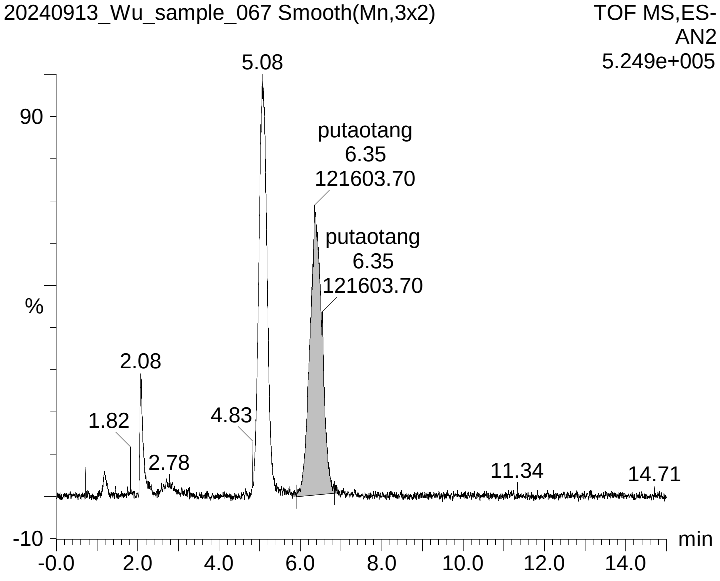

	#	Name	Sample Text	RT	Area	Std. Conc	Conc.
1	1	guotang		5.08	121018.297		83.196684
2	2	putaotang		6.35	121603.703		85.744566

Name: 20240913_Wu_sample_068, Date: 14-Sep-2024, Time: 11:54:36, ID: , Description:

guotang

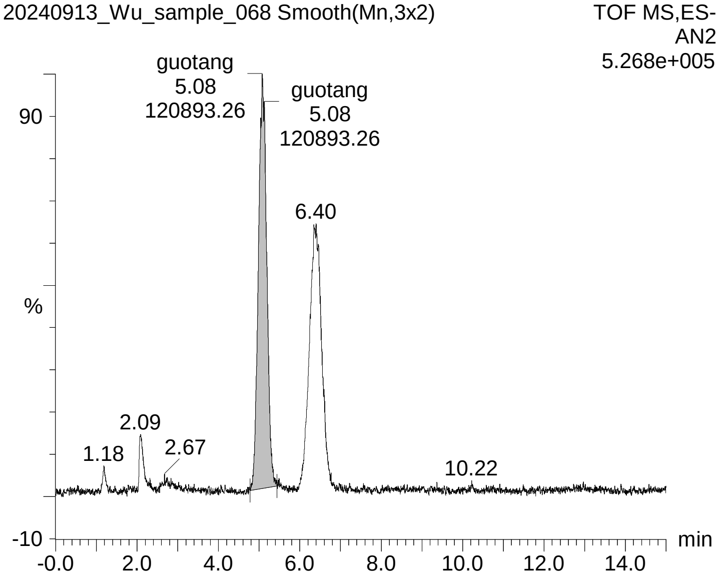

putaotang

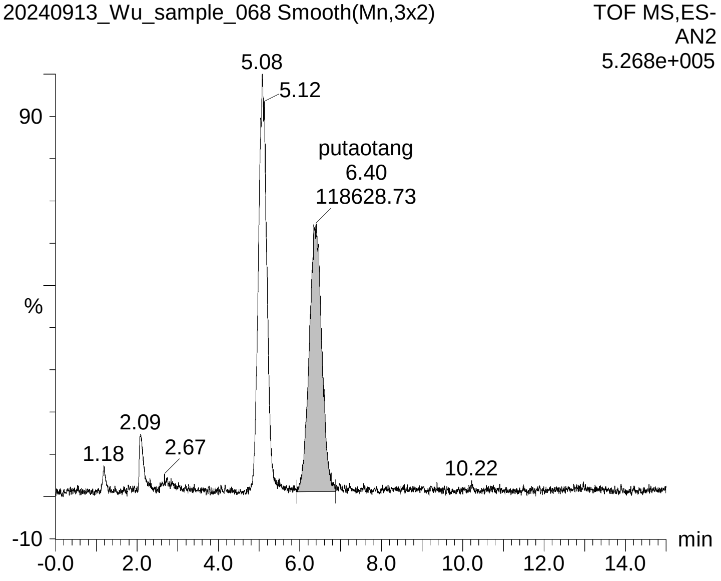

	#	Name	Sample Text	RT	Area	Std. Conc	Conc.
1	1	guotang		5.08	120893.258		83.125529
2	2	putaotang		6.40	118628.727		84.003416

project_wangzhonghua_BeiMu
Dataset: Untitled
Last Altered: Sunday, September 29, 2024 09:54:36 China Standard Time
Printed: Sunday, September 29, 2024 09:59:41 China Standard Time

Name: 20240913_Wu_sample_069, Date: 14-Sep-2024, Time: 12:10:34, ID: , Description:

guotang

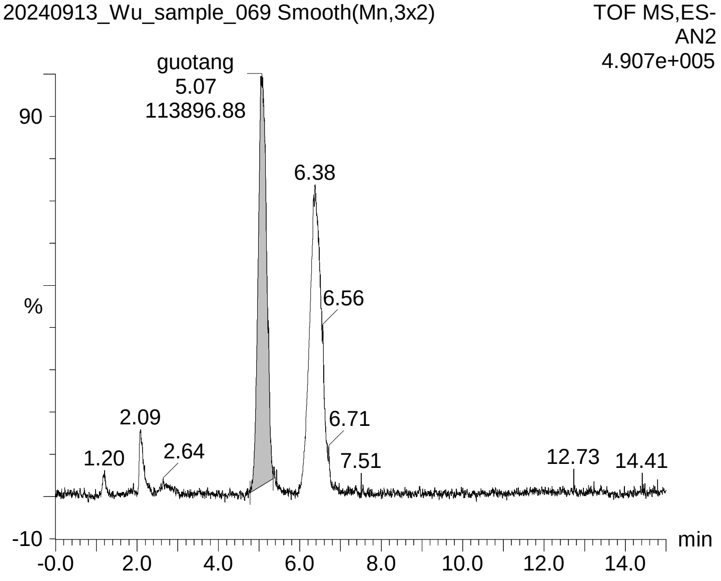

putaotang

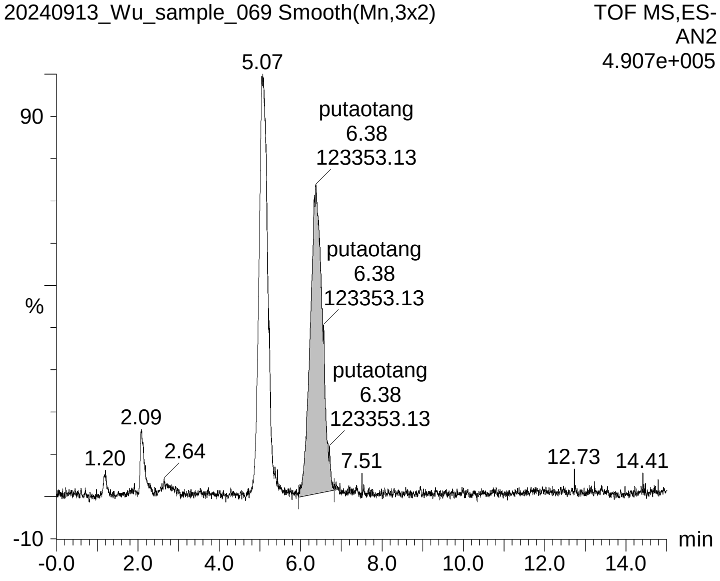

	#	Name	Sample Text	RT	Area	Std. Conc	Conc.
1	1	guotang		5.07	113896.883		79.144110
2	2	putaotang		6.38	123353.125		86.768442

project_wangzhonghua_BeiMu
Dataset: Untitled
Last Altered: Sunday, September 29, 2024 09:54:36 China Standard Time
Printed: Sunday, September 29, 2024 09:59:41 China Standard Time

Name: 20240913_Wu_sample_070, Date: 14-Sep-2024, Time: 12:26:36, ID: , Description:

guotang

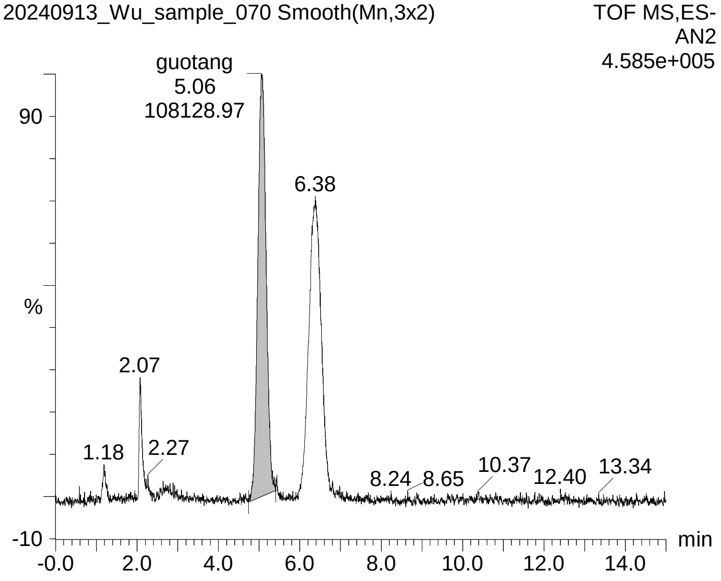

putaotang

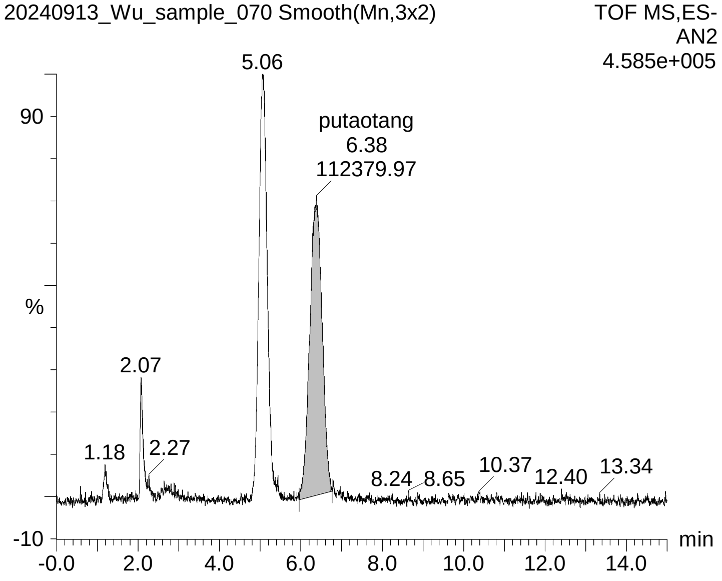

	#	Name	Sample Text	RT	Area	Std. Conc	Conc.
1	1	guotang		5.06	108128.969		75.861769
2	2	putaotang		6.38	112379.969		80.346237

Name: 20240913_Wu_sample_071, Date: 14-Sep-2024, Time: 12:42:37, ID: , Description:

guotang

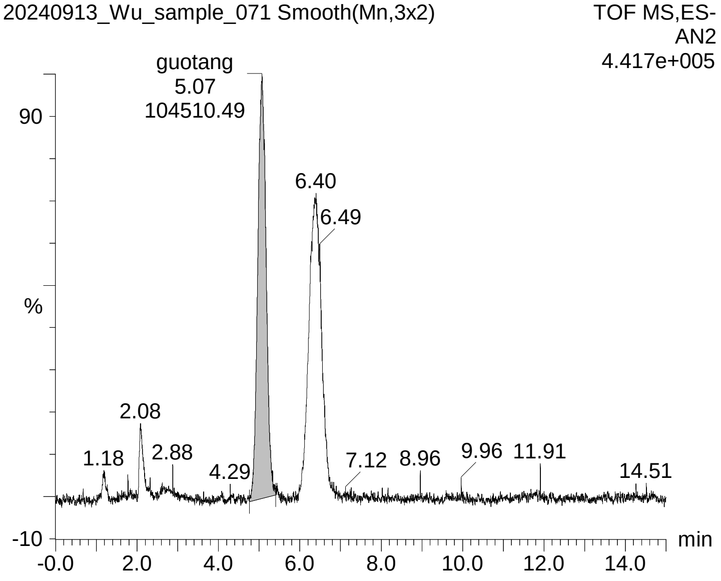

putaotang

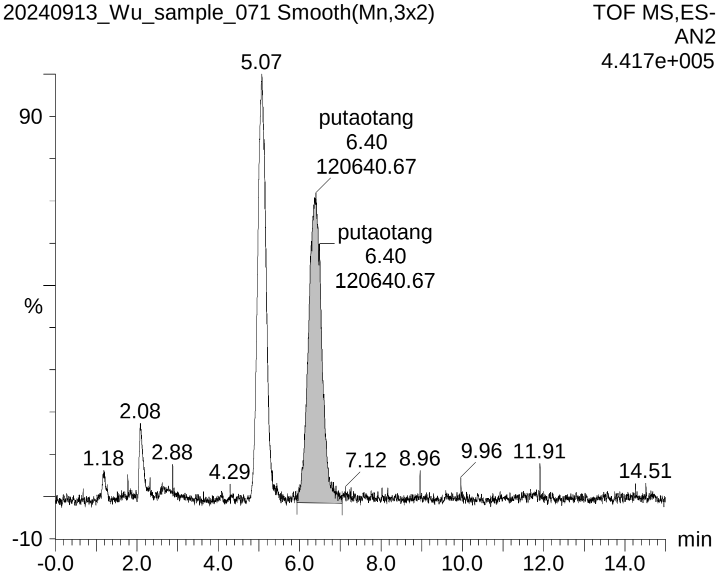

	#	Name	Sample Text	RT	Area	Std. Conc	Conc.
1	1	guotang		5.07	104510.492		73.802607
2	2	putaotang		6.40	120640.672		85.180938

Name: 20240913_Wu_sample_072, Date: 14-Sep-2024, Time: 12:58:46, ID: , Description:

guotang

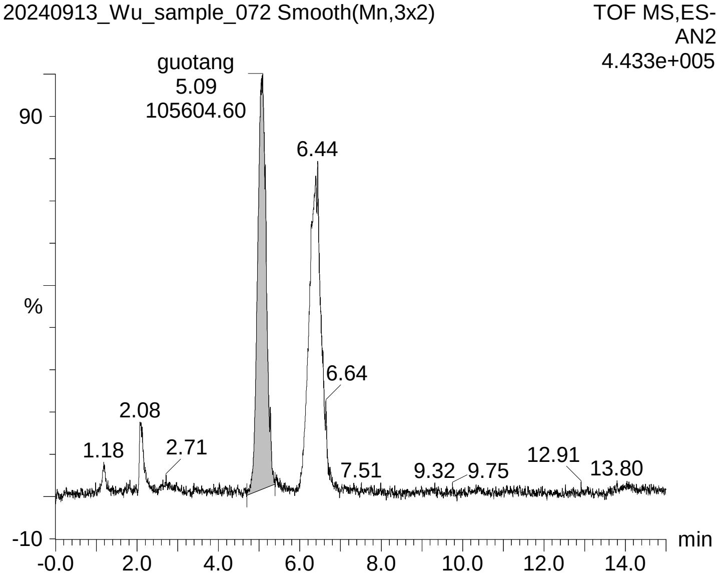

putaotang

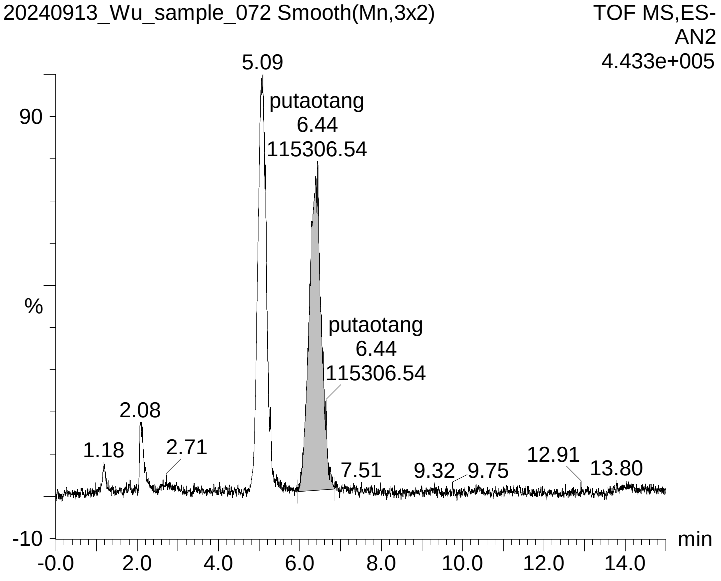

	#	Name	Sample Text	RT	Area	Std. Conc	Conc.
1	1	guotang		5.09	105604.602		74.425231
2	2	putaotang		6.44	115306.539		82.059056

Name: 20240913_Wu_sample_073, Date: 14-Sep-2024, Time: 13:14:45, ID: , Description:

guotang

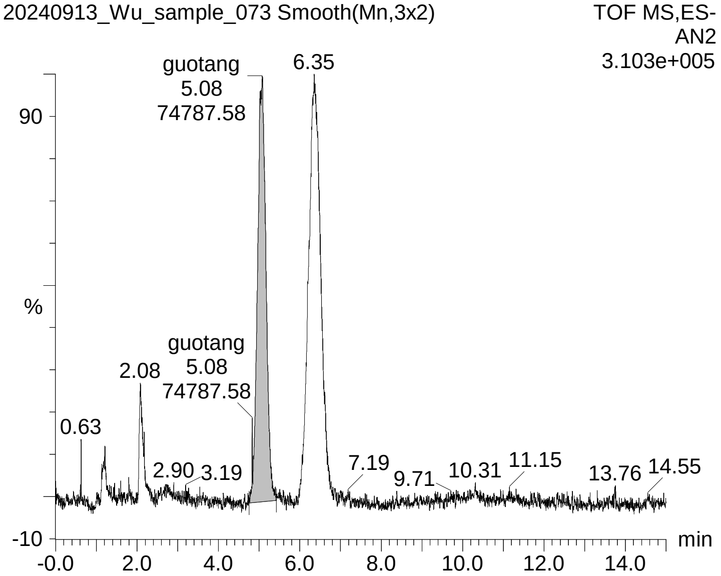

putaotang

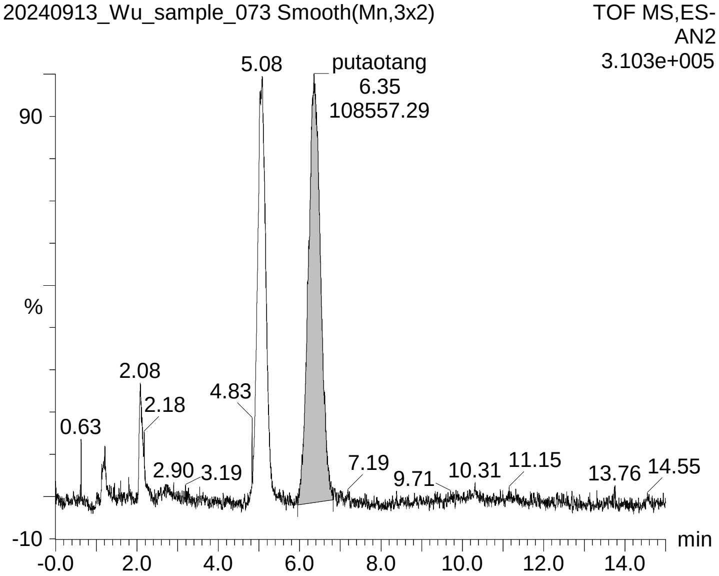

	#	Name	Sample Text	RT	Area	Std. Conc	Conc.
1	1	guotang		5.08	74787.578		56.888222
2	2	putaotang		6.35	108557.289		78.108956

project_wangzhonghua_BeiMu
Dataset: Untitled
Last Altered: Sunday, September 29, 2024 09:54:36 China Standard Time
Printed: Sunday, September 29, 2024 09:59:41 China Standard Time

Name: 20240913_Wu_sample_074, Date: 14-Sep-2024, Time: 13:30:44, ID: , Description:

guotang

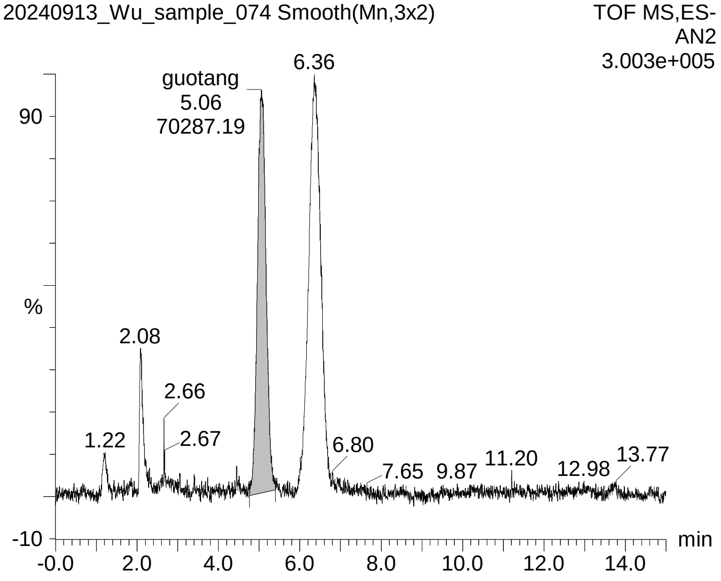

putaotang

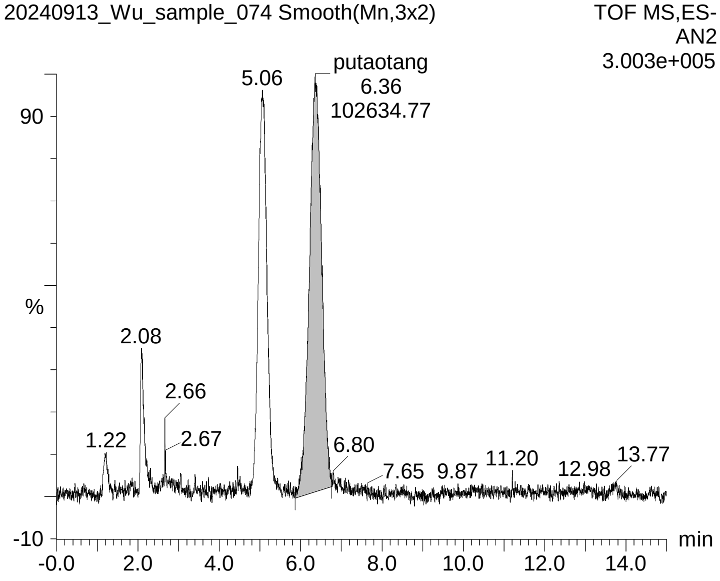

	#	Name	Sample Text	RT	Area	Std. Conc	Conc.
1	1	guotang		5.06	70287.188		54.327191
2	2	putaotang		6.36	102634.766		74.642710

Name: 20240913_Wu_sample_075, Date: 14-Sep-2024, Time: 13:46:46, ID: , Description:

guotang

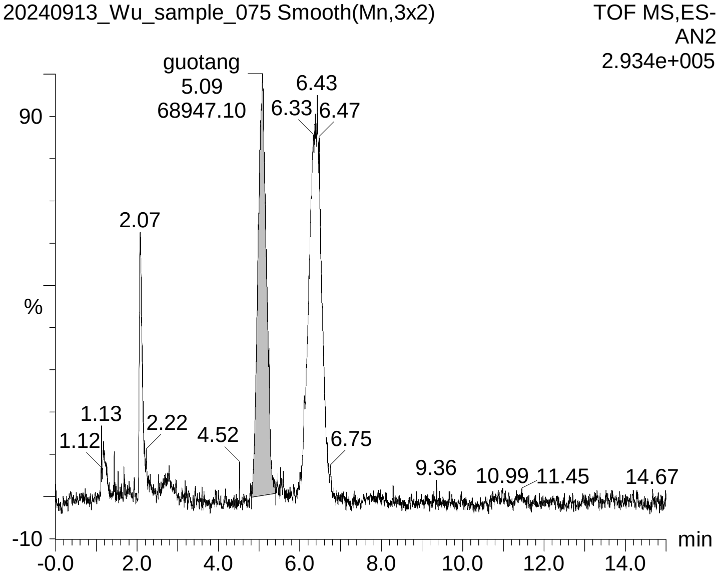

putaotang

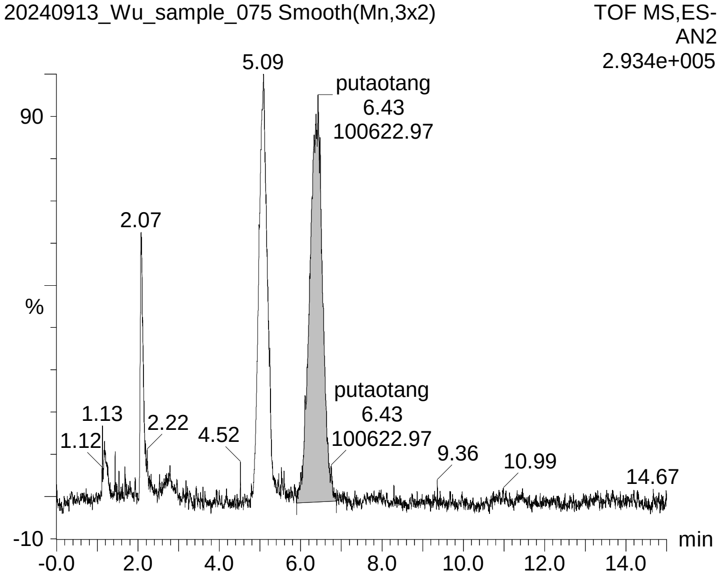

	#	Name	Sample Text	RT	Area	Std. Conc	Conc.
1	1	guotang		5.09	68947.102		53.564590
2	2	putaotang		6.43	100622.969		73.465276

project_wangzhonghua_BeiMu
Dataset: Untitled
Last Altered: Sunday, September 29, 2024 09:54:36 China Standard Time
Printed: Sunday, September 29, 2024 09:59:41 China Standard Time

Name: 20240913_Wu_sample_076, Date: 14-Sep-2024, Time: 14:02:48, ID: , Description:

guotang

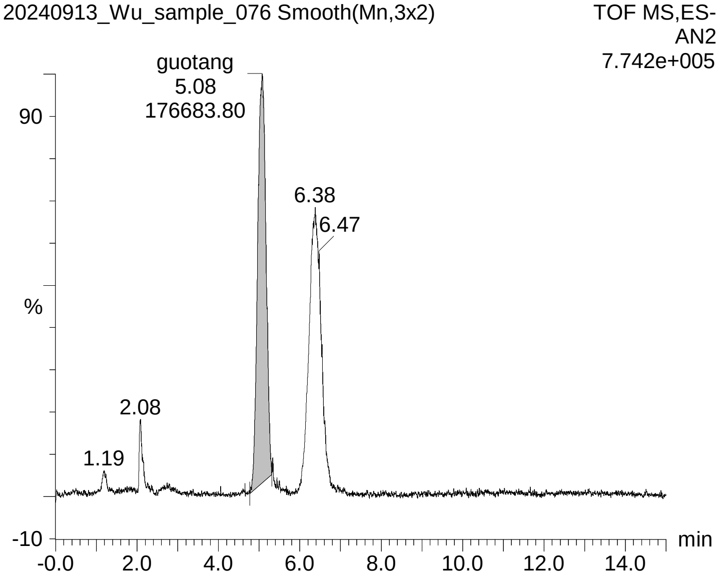

putaotang

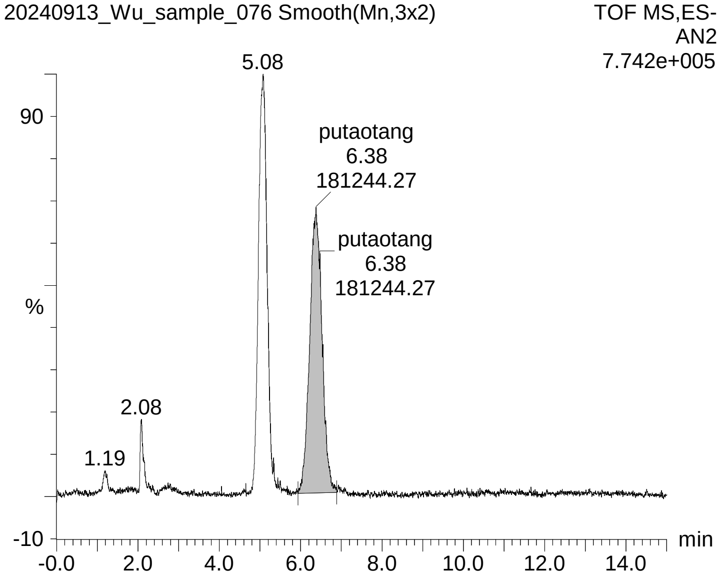

	#	Name	Sample Text	RT	Area	Std. Conc	Conc.
1	1	guotang		5.08	176683.797		114.874186
2	2	putaotang		6.38	181244.266		120.650103

project_wangzhonghua_BeiMu
Dataset: Untitled
Last Altered: Sunday, September 29, 2024 09:54:36 China Standard Time
Printed: Sunday, September 29, 2024 09:59:41 China Standard Time

Name: 20240913_Wu_sample_077, Date: 14-Sep-2024, Time: 14:18:44, ID: , Description:

guotang

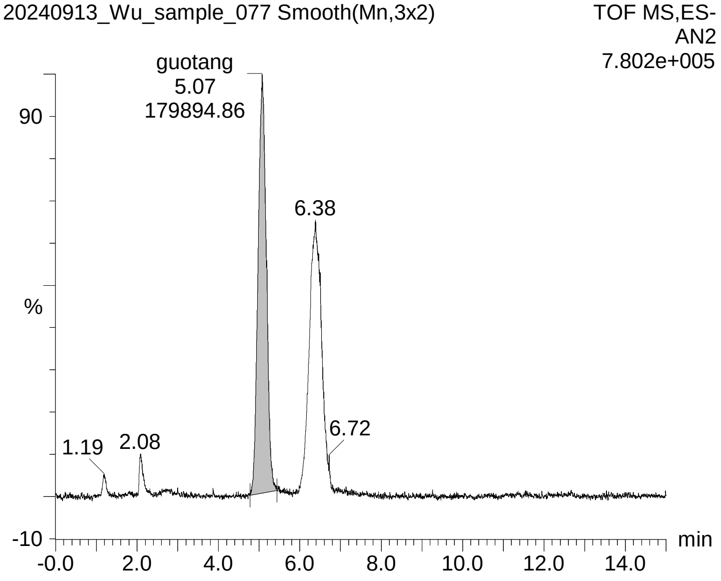

putaotang

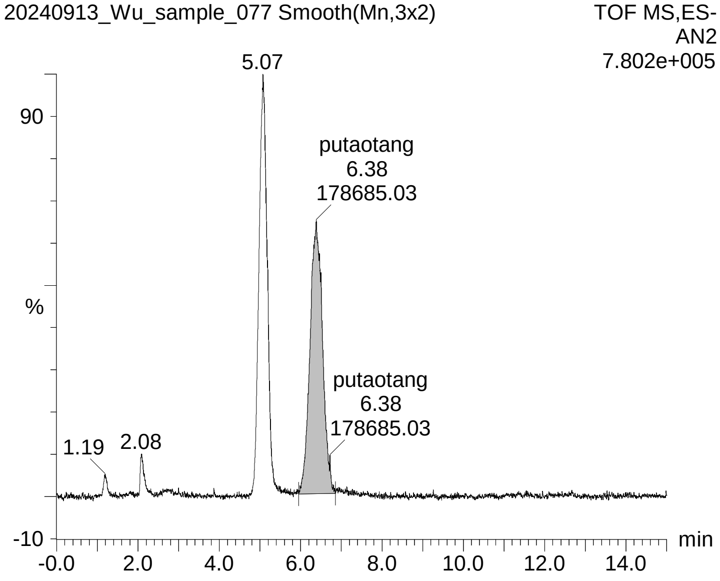

	#	Name	Sample Text	RT	Area	Std. Conc	Conc.
1	1	guotang		5.07	179894.859		116.701502
2	2	putaotang		6.38	178685.031		119.152272

project_wangzhonghua_BeiMu
Dataset: Untitled
Last Altered: Sunday, September 29, 2024 09:54:36 China Standard Time
Printed: Sunday, September 29, 2024 09:59:41 China Standard Time

Name: 20240913_Wu_sample_078, Date: 14-Sep-2024, Time: 14:34:44, ID: , Description:

guotang

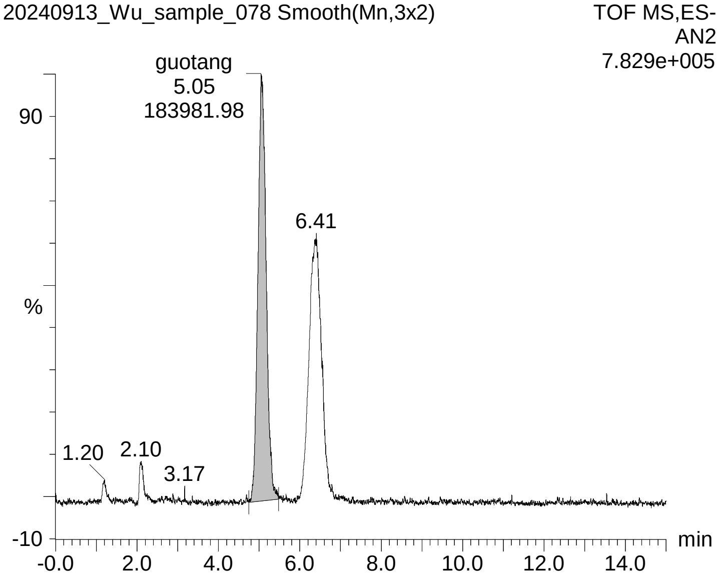

putaotang

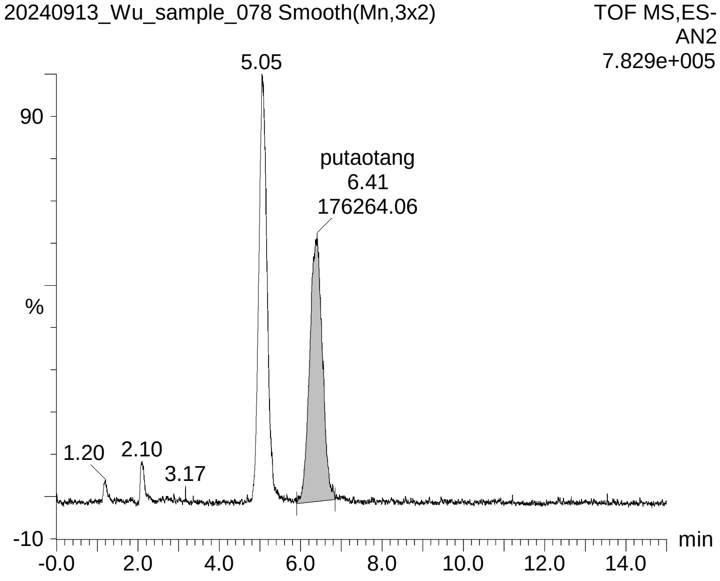

	#	Name	Sample Text	RT	Area	Std. Conc	Conc.
1	1	guotang		5.05	183981.984		119.027357
2	2	putaotang		6.41	176264.063		117.735364

project_wangzhonghua_BeiMu
Dataset: Untitled
Last Altered: Sunday, September 29, 2024 09:54:36 China Standard Time
Printed: Sunday, September 29, 2024 09:59:41 China Standard Time

Name: 20240913_Wu_sample_079, Date: 14-Sep-2024, Time: 14:50:46, ID: , Description:

guotang

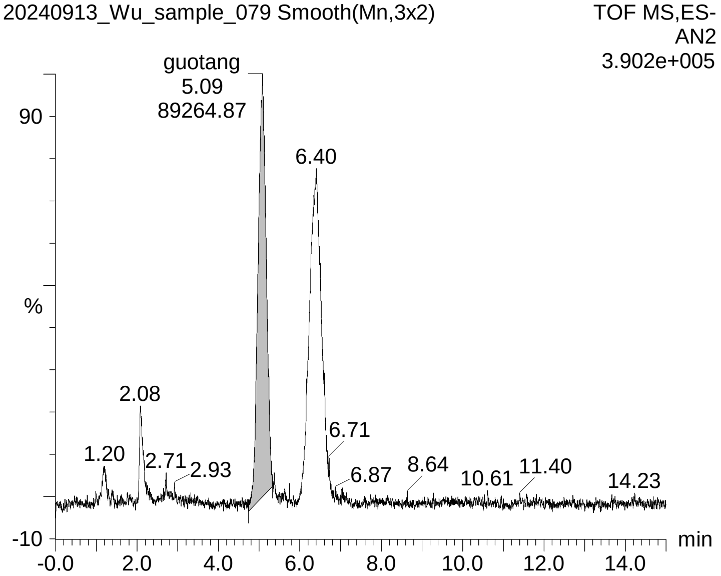

putaotang

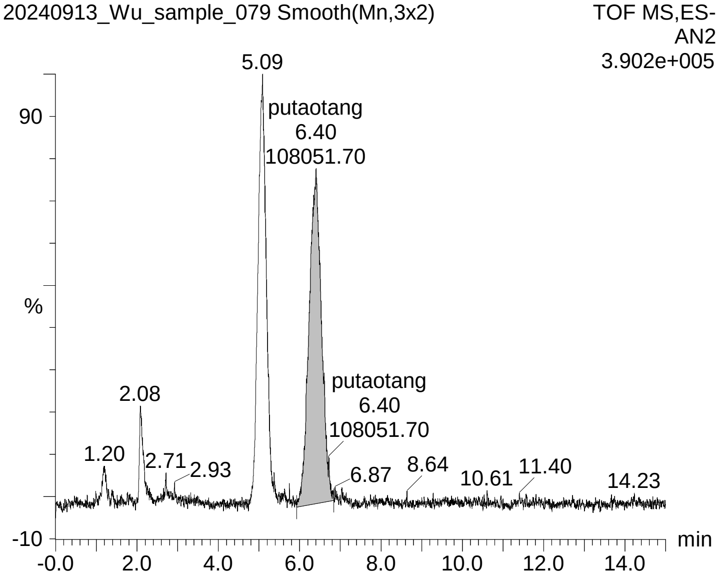

	#	Name	Sample Text	RT	Area	Std. Conc	Conc.
1	1	guotang		5.09	89264.867		65.126797
2	2	putaotang		6.40	108051.703		77.813054

Name: 20240913_Wu_sample_080, Date: 14-Sep-2024, Time: 15:06:48, ID: , Description:

guotang

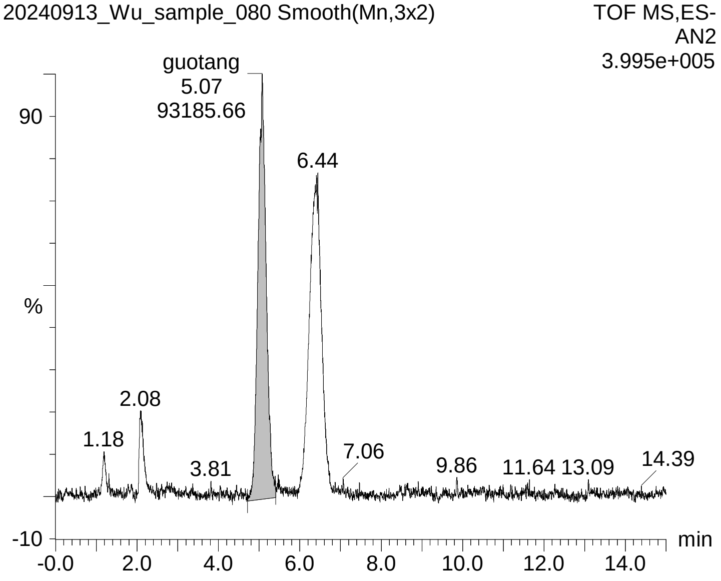

putaotang

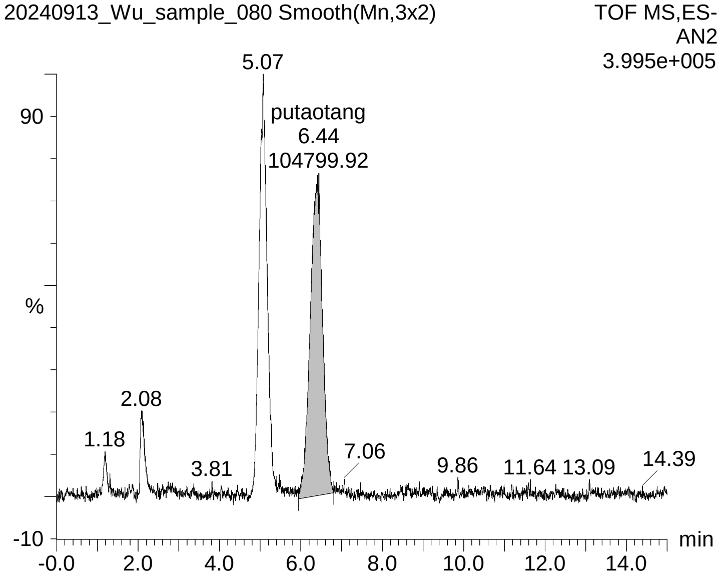

	#	Name	Sample Text	RT	Area	Std. Conc	Conc.
1	1	guotang		5.07	93185.656		67.357995
2	2	putaotang		6.44	104799.922		75.909900

project_wangzhonghua_BeiMu
Dataset: Untitled
Last Altered: Sunday, September 29, 2024 09:54:36 China Standard Time
Printed: Sunday, September 29, 2024 09:59:41 China Standard Time

Name: 20240913_Wu_sample_081, Date: 14-Sep-2024, Time: 15:22:50, ID: , Description:

guotang

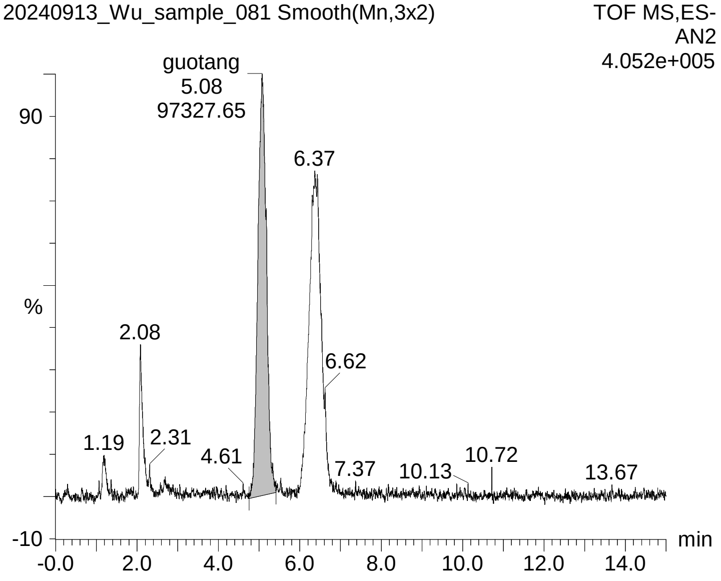

putaotang

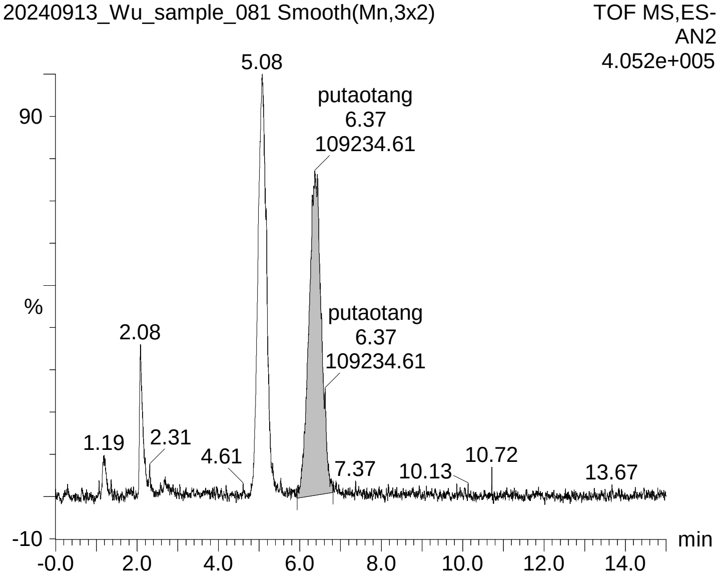

	#	Name	Sample Text	RT	Area	Std. Conc	Conc.
1	1	guotang		5.08	97327.648		69.715074
2	2	putaotang		6.37	109234.609		78.505368

Name: 20240913_Wu_sample_082, Date: 14-Sep-2024, Time: 15:38:51, ID: , Description:

guotang

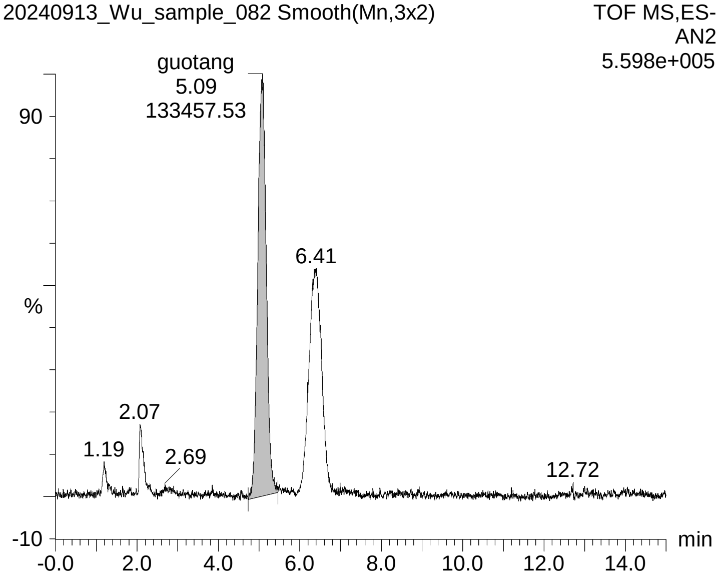

putaotang

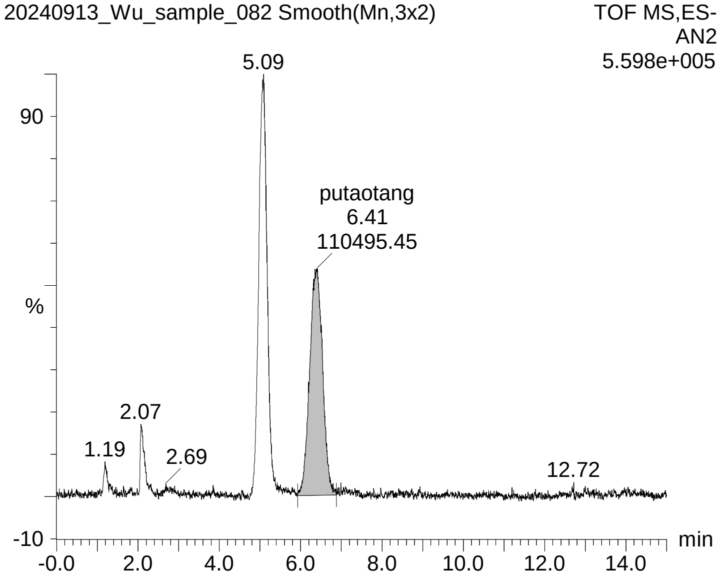

	#	Name	Sample Text	RT	Area	Std. Conc	Conc.
1	1	guotang		5.09	133457.531		90.275465
2	2	putaotang		6.41	110495.453		79.243295

project_wangzhonghua_BeiMu
Dataset: Untitled
Last Altered: Sunday, September 29, 2024 09:54:36 China Standard Time
Printed: Sunday, September 29, 2024 09:59:41 China Standard Time

Name: 20240913_Wu_sample_083, Date: 14-Sep-2024, Time: 15:54:54, ID: , Description:

guotang

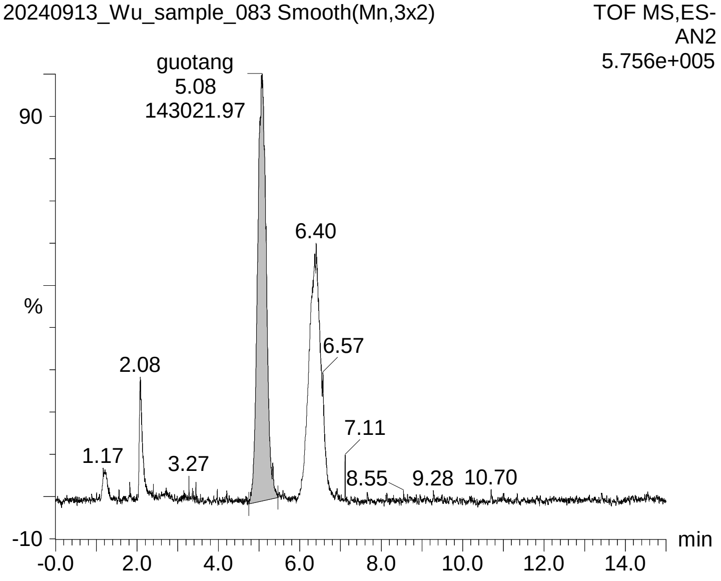

putaotang

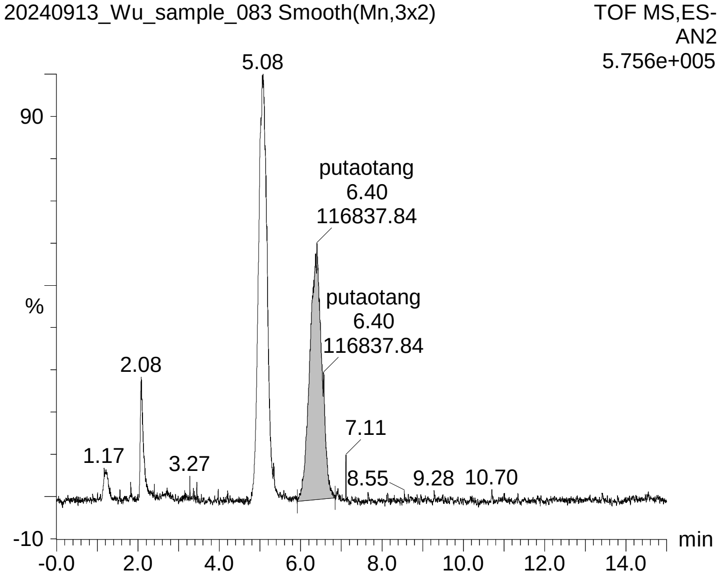

	#	Name	Sample Text	RT	Area	Std. Conc	Conc.
1	1	guotang		5.08	143021.969		95.718289
2	2	putaotang		6.40	116837.836		82.955271

project_wangzhonghua_BeiMu
Dataset: Untitled
Last Altered: Sunday, September 29, 2024 09:54:36 China Standard Time
Printed: Sunday, September 29, 2024 09:59:41 China Standard Time

Name: 20240913_Wu_sample_084, Date: 14-Sep-2024, Time: 16:10:56, ID: , Description:

guotang

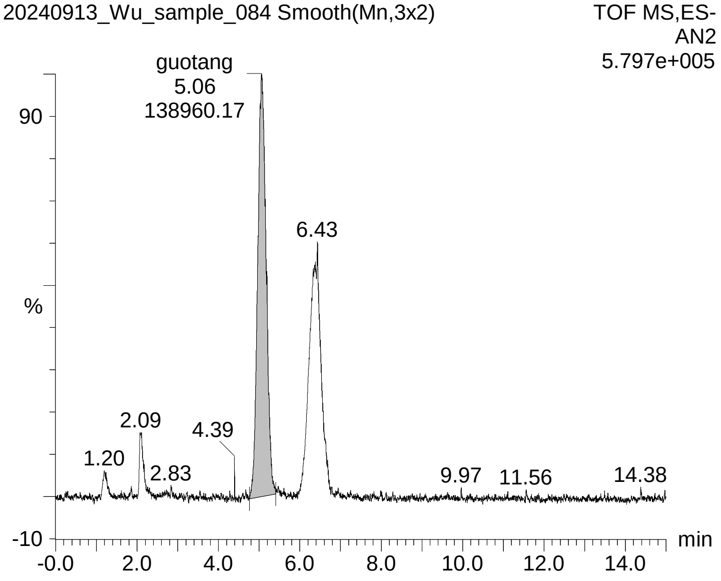

putaotang

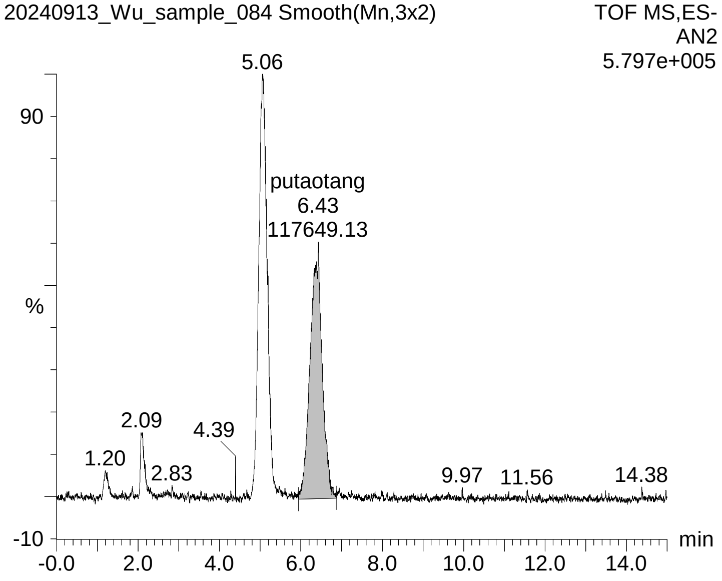

	#	Name	Sample Text	RT	Area	Std. Conc	Conc.
1	1	guotang		5.06	138960.172		93.406847
2	2	putaotang		6.43	117649.133		83.430094

project_wangzhonghua_BeiMu
Dataset: Untitled
Last Altered: Sunday, September 29, 2024 09:54:36 China Standard Time
Printed: Sunday, September 29, 2024 09:59:41 China Standard Time

Name: 20240913_Wu_sample_085, Date: 14-Sep-2024, Time: 16:26:59, ID: , Description:

guotang

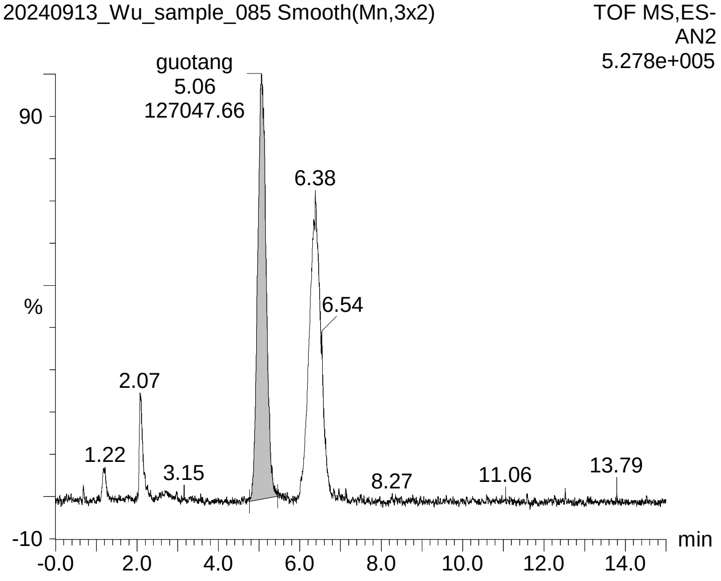

putaotang

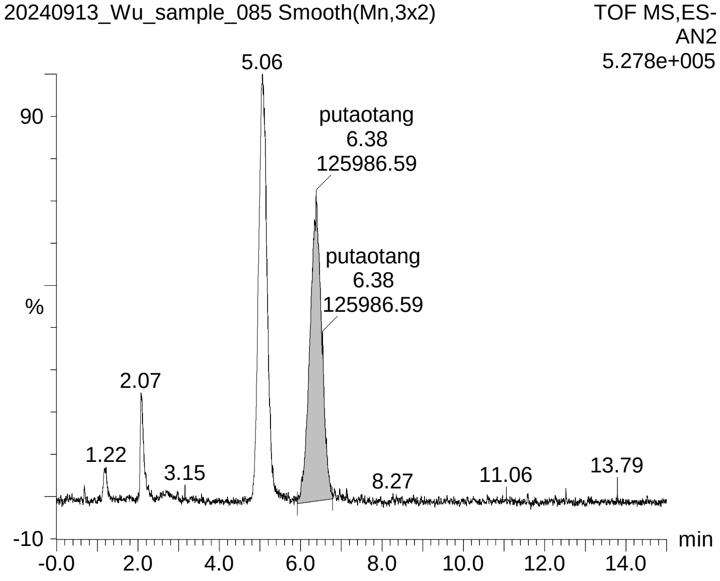

	#	Name	Sample Text	RT	Area	Std. Conc	Conc.
1	1	guotang		5.06	127047.664		86.627809
2	2	putaotang		6.38	125986.594		88.309719

project_wangzhonghua_BeiMu
Dataset: Untitled
Last Altered: Sunday, September 29, 2024 09:54:36 China Standard Time
Printed: Sunday, September 29, 2024 09:59:41 China Standard Time

Name: 20240913_Wu_sample_086, Date: 14-Sep-2024, Time: 16:43:01, ID: , Description:

guotang

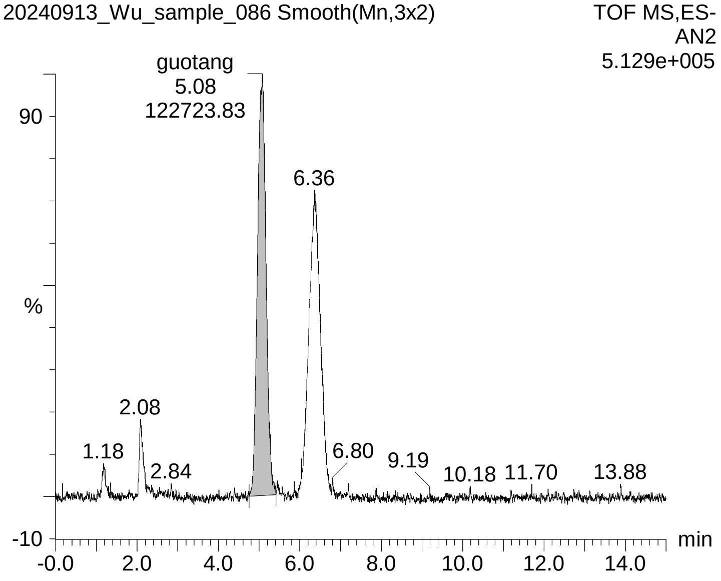

putaotang

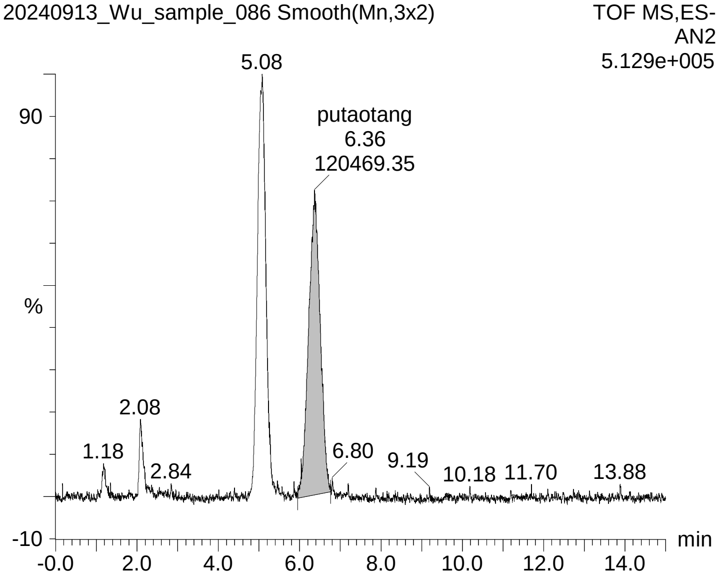

	#	Name	Sample Text	RT	Area	Std. Conc	Conc.
1	1	guotang		5.08	122723.828		84.167249
2	2	putaotang		6.36	120469.352		85.080670

Name: 20240913_Wu_sample_087, Date: 14-Sep-2024, Time: 16:59:02, ID: , Description:

guotang

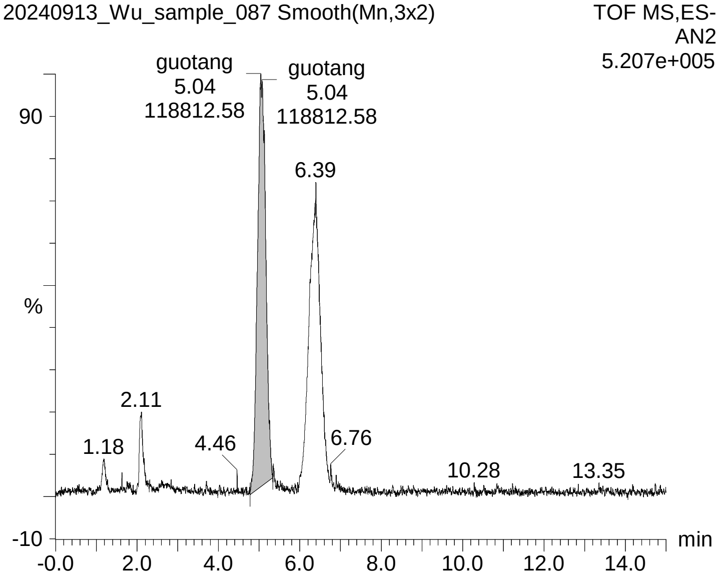

putaotang

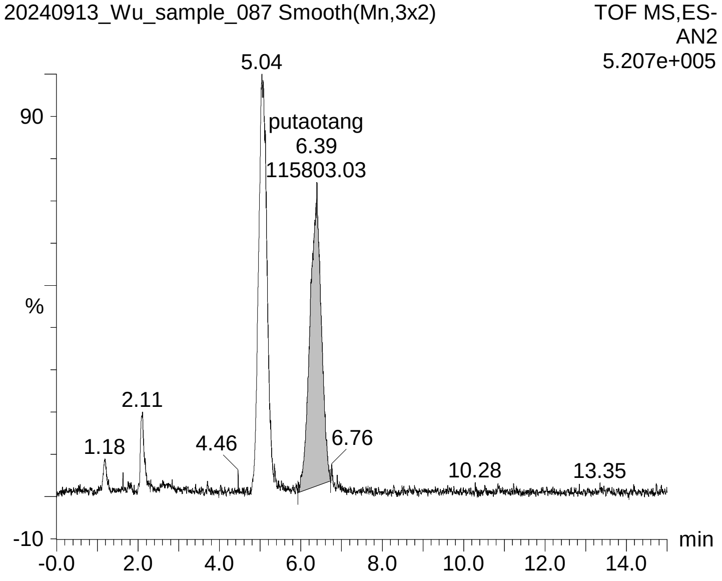

	#	Name	Sample Text	RT	Area	Std. Conc	Conc.
1	1	guotang		5.04	118812.578		81.941478
2	2	putaotang		6.39	115803.031		82.349635

project_wangzhonghua_BeiMu
Dataset: Untitled
Last Altered: Sunday, September 29, 2024 09:54:36 China Standard Time
Printed: Sunday, September 29, 2024 09:59:41 China Standard Time

Name: 20240913_Wu_sample_088, Date: 14-Sep-2024, Time: 17:15:04, ID: , Description:

guotang

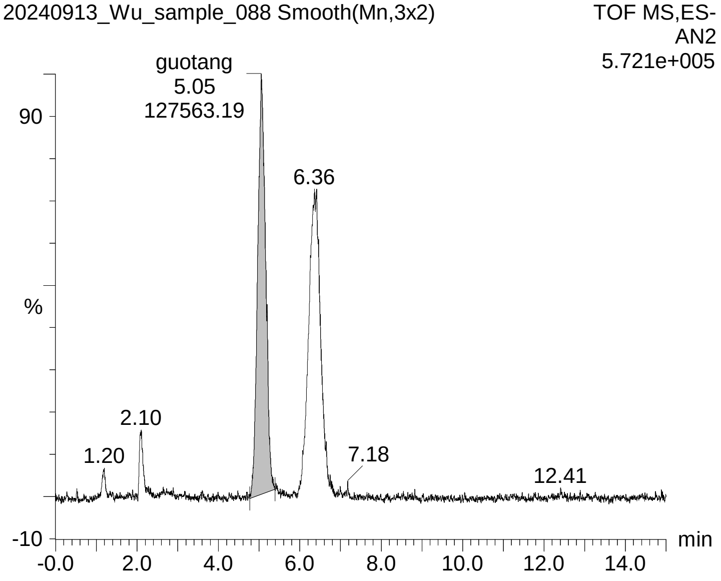

putaotang

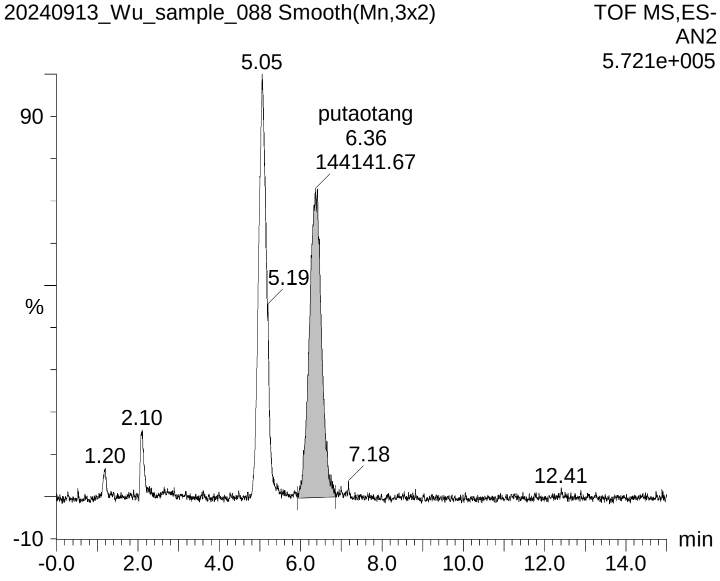

	#	Name	Sample Text	RT	Area	Std. Conc	Conc.
1	1	guotang		5.05	127563.188		86.921178
2	2	putaotang		6.36	144141.672		98.935252

Name: 20240913_Wu_sample_089, Date: 14-Sep-2024, Time: 17:31:06, ID: , Description:

guotang

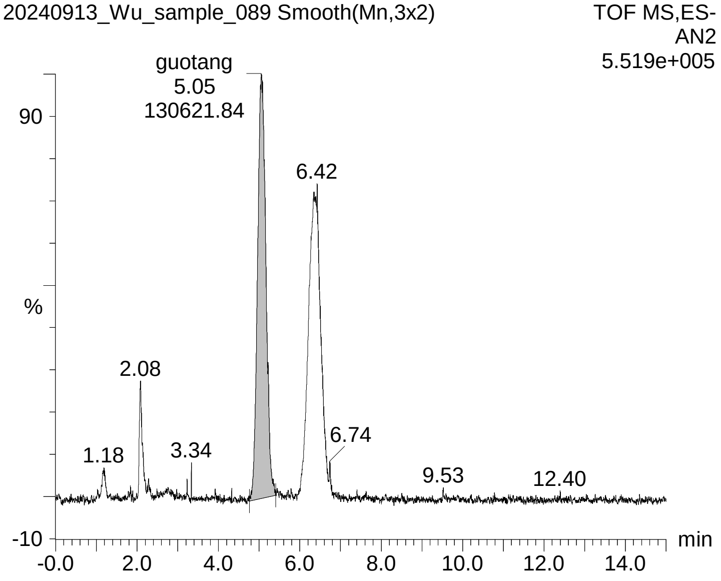

putaotang

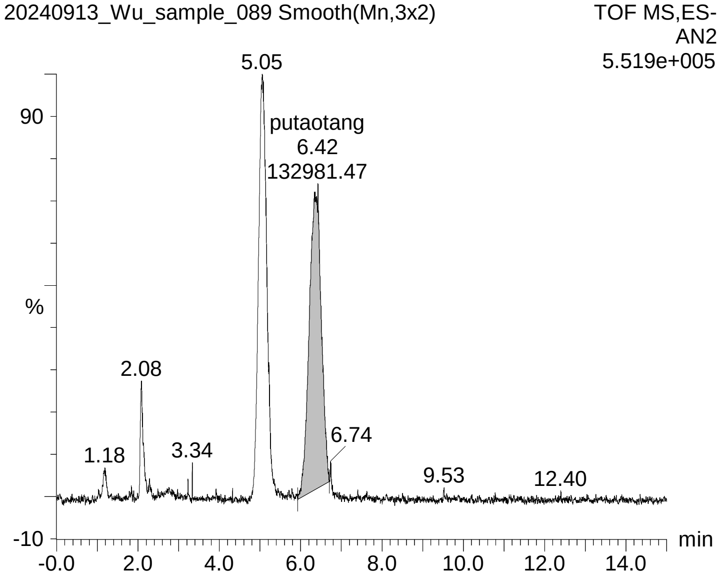

	#	Name	Sample Text	RT	Area	Std. Conc	Conc.
1	1	guotang		5.05	130621.836		88.661759
2	2	putaotang		6.42	132981.469		92.403575

Name: 20240913_Wu_sample_090, Date: 14-Sep-2024, Time: 17:47:09, ID: , Description:

guotang

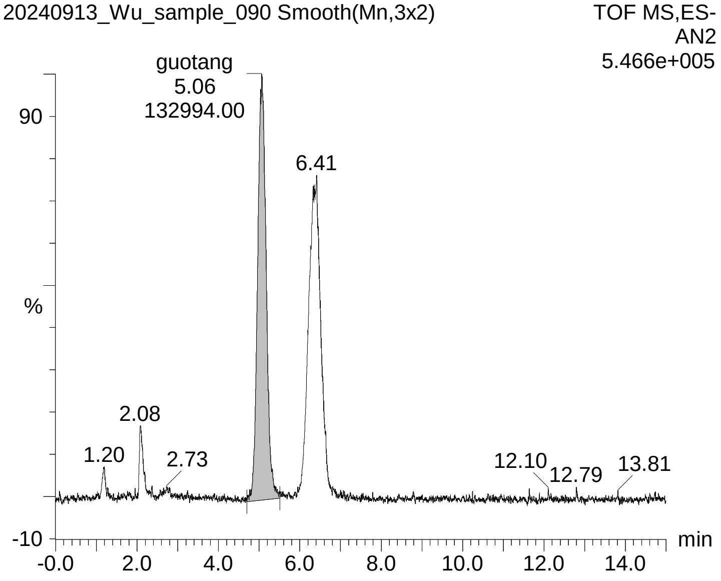

putaotang

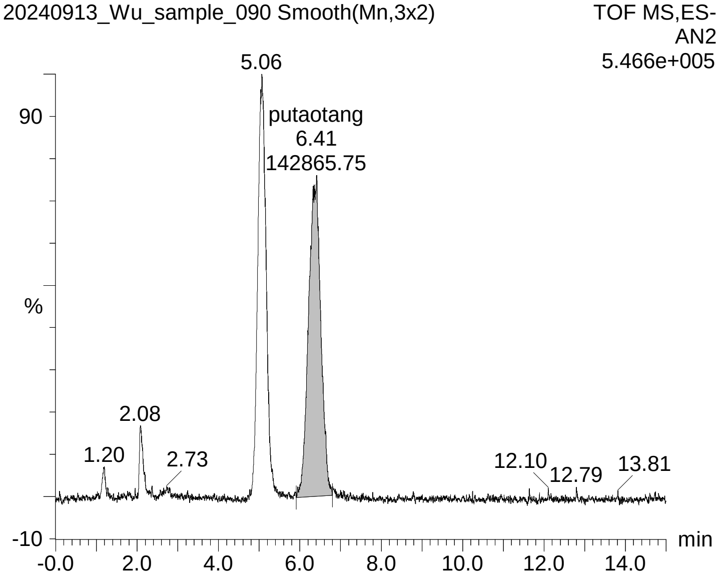

	#	Name	Sample Text	RT	Area	Std. Conc	Conc.
1	1	guotang		5.06	132994.000		90.011684
2	2	putaotang		6.41	142865.750		98.188499

Name: 20240914_Wu_sample_091, Date: 14-Sep-2024, Time: 18:03:13, ID: , Description:

guotang

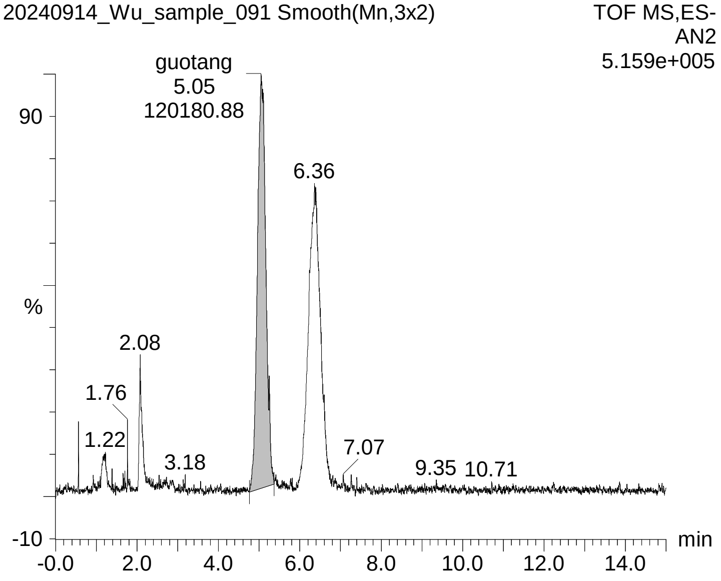

putaotang

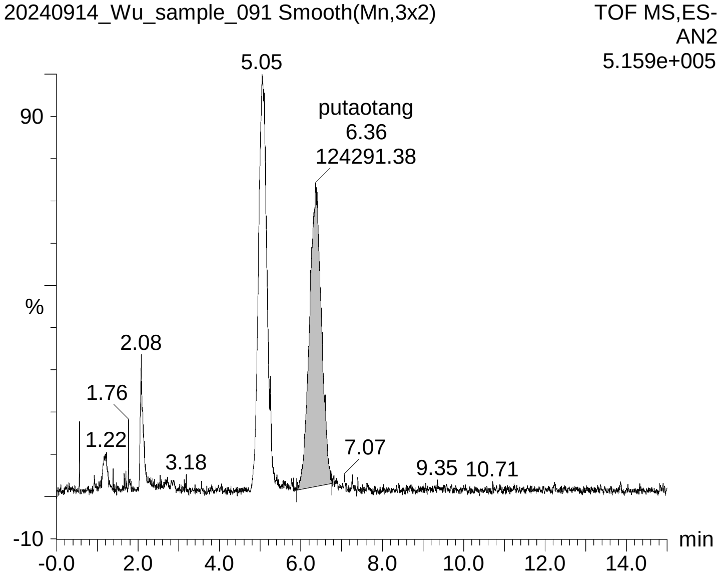

	#	Name	Sample Text	RT	Area	Std. Conc	Conc.
1	1	guotang		5.05	120180.875		82.720134
2	2	putaotang		6.36	124291.375		87.317566

project_wangzhonghua_BeiMu
Dataset: Untitled
Last Altered: Sunday, September 29, 2024 09:54:36 China Standard Time
Printed: Sunday, September 29, 2024 09:59:41 China Standard Time

Name: 20240914_Wu_sample_092, Date: 14-Sep-2024, Time: 18:19:11, ID: , Description:

guotang

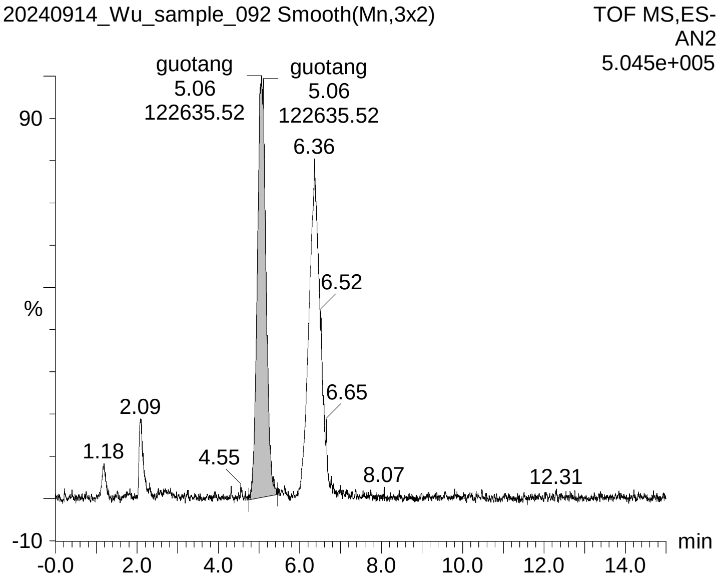

putaotang

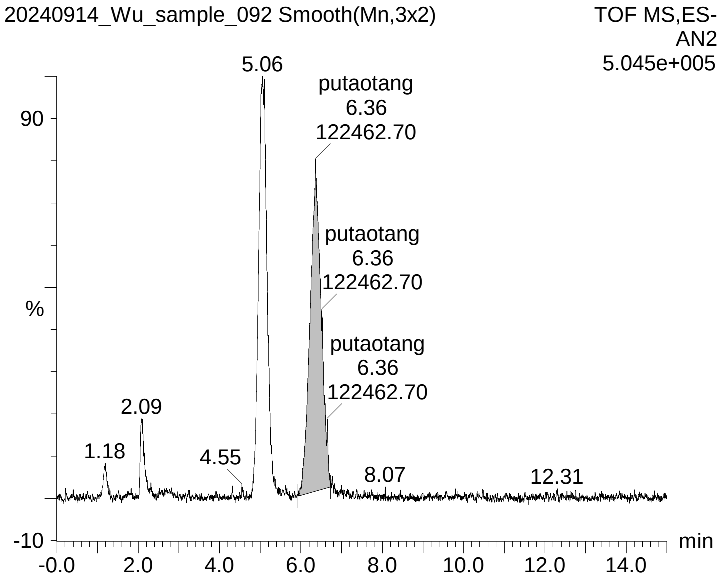

	#	Name	Sample Text	RT	Area	Std. Conc	Conc.
1	1	guotang		5.06	122635.516		84.116993
2	2	putaotang		6.36	122462.695		86.247304

Name: 20240914_Wu_sample_093, Date: 14-Sep-2024, Time: 18:35:13, ID: , Description:

guotang

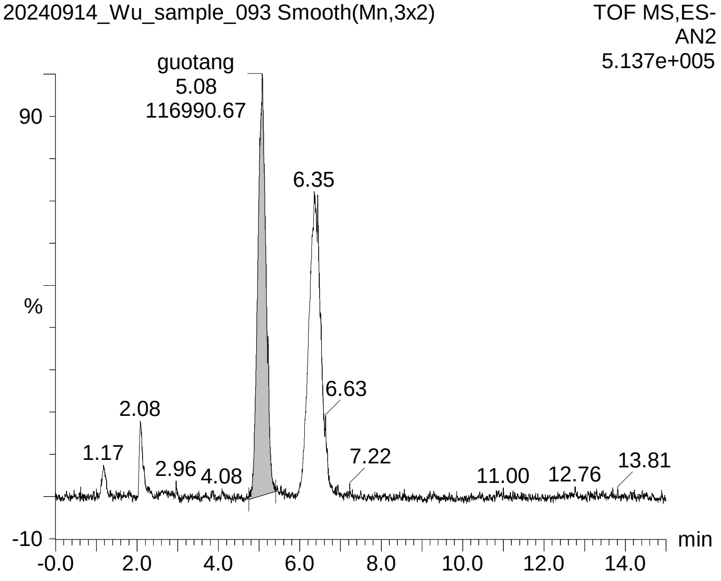

putaotang

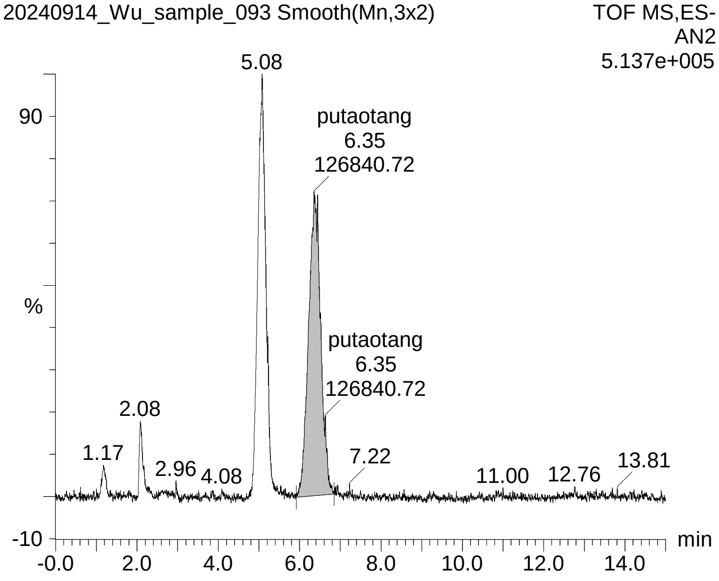

	#	Name	Sample Text	RT	Area	Std. Conc	Conc.
1	1	guotang		5.08	116990.672		80.904688
2	2	putaotang		6.35	126840.719		88.809608

project_wangzhonghua_BeiMu
Dataset: Untitled
Last Altered: Sunday, September 29, 2024 09:54:36 China Standard Time
Printed: Sunday, September 29, 2024 09:59:41 China Standard Time

Name: 20240914_Wu_sample_094, Date: 14-Sep-2024, Time: 18:51:17, ID: , Description:

guotang

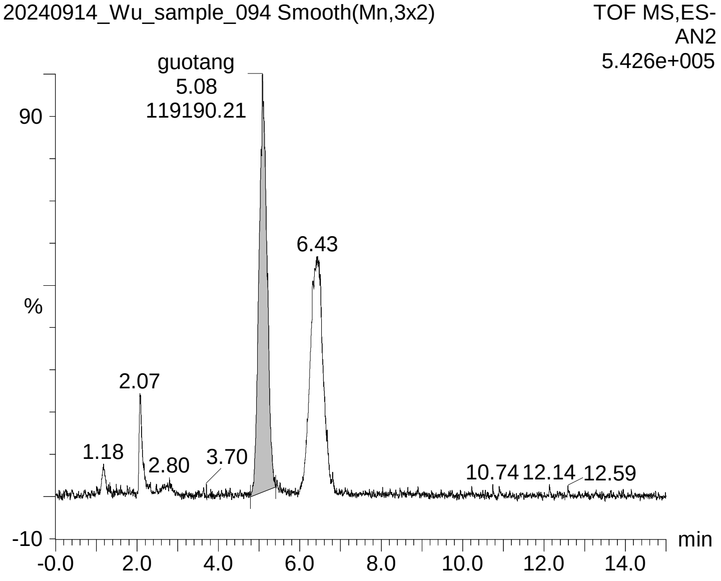

putaotang

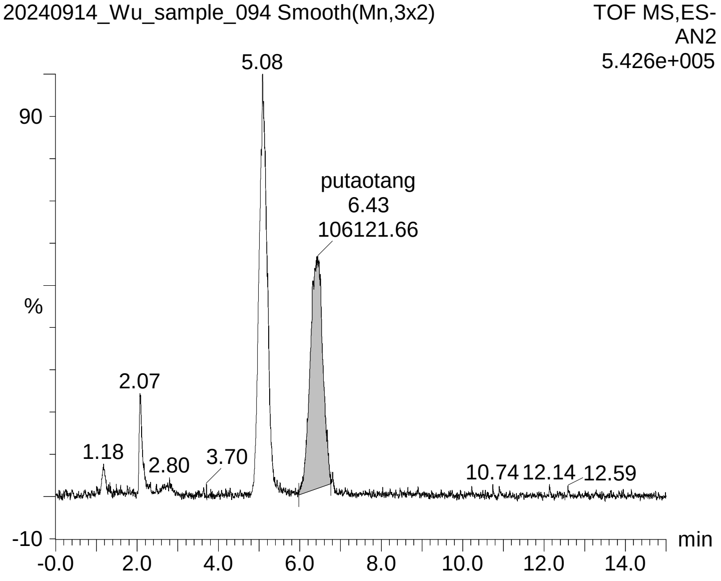

	#	Name	Sample Text	RT	Area	Std. Conc	Conc.
1	1	guotang		5.08	119190.211		82.156378
2	2	putaotang		6.43	106121.656		76.683465

project_wangzhonghua_BeiMu
Dataset: Untitled
Last Altered: Sunday, September 29, 2024 09:54:36 China Standard Time
Printed: Sunday, September 29, 2024 09:59:41 China Standard Time

Name: 20240914_Wu_sample_095, Date: 14-Sep-2024, Time: 19:07:19, ID: , Description:

guotang

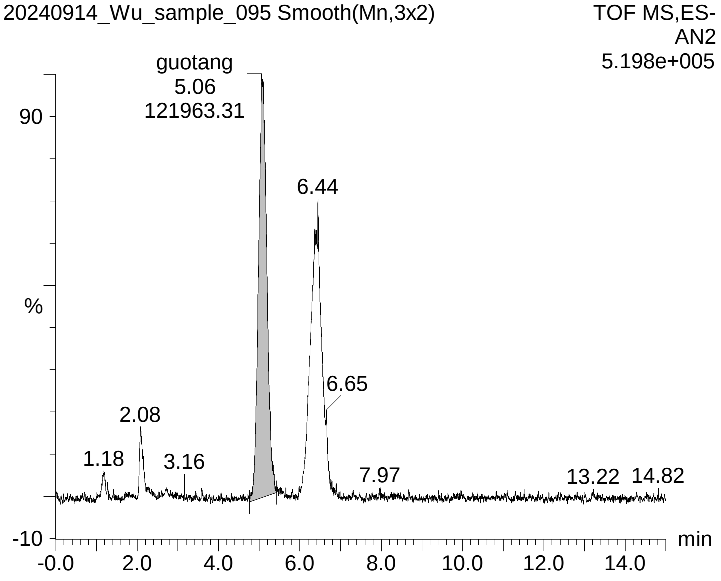

putaotang

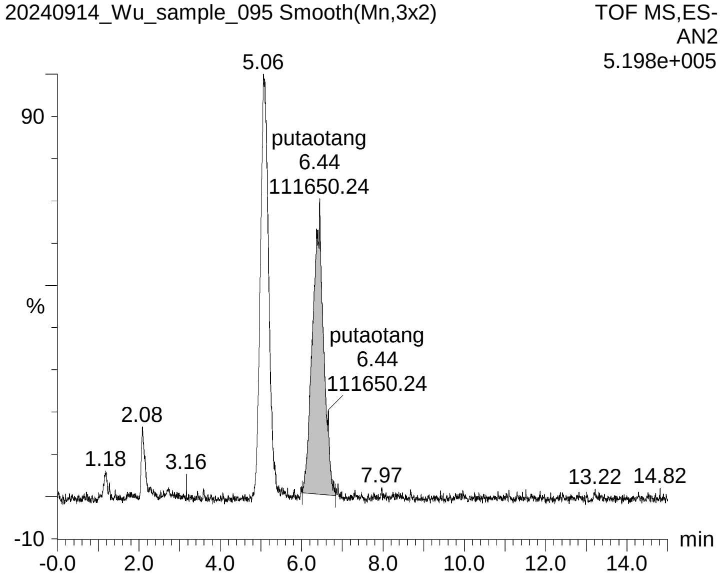

	#	Name	Sample Text	RT	Area	Std. Conc	Conc.
1	1	guotang		5.06	121963.313		83.734464
2	2	putaotang		6.44	111650.242		79.919153

project_wangzhonghua_BeiMu
Dataset: Untitled
Last Altered: Sunday, September 29, 2024 09:54:36 China Standard Time
Printed: Sunday, September 29, 2024 09:59:41 China Standard Time

Name: 20240914_Wu_sample_096, Date: 14-Sep-2024, Time: 19:23:20, ID: , Description:

guotang

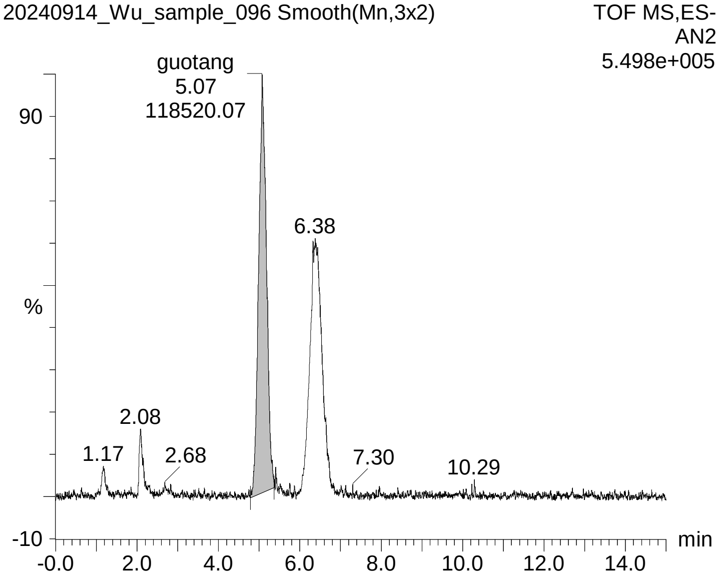

putaotang

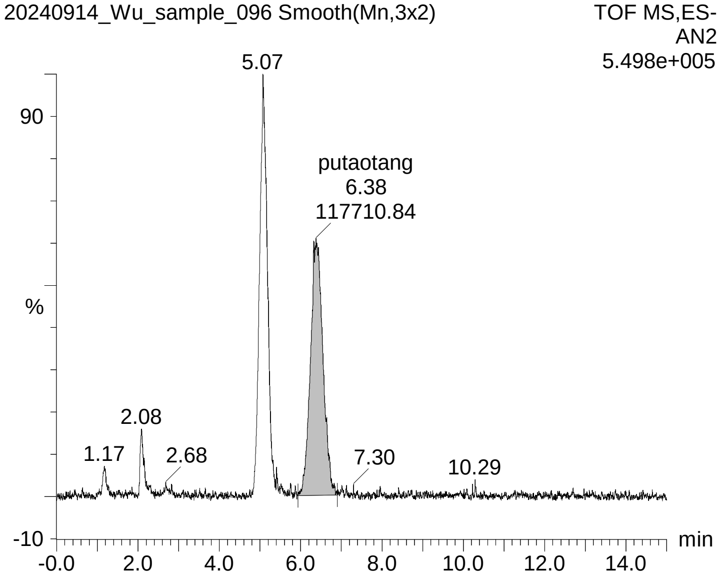

	#	Name	Sample Text	RT	Area	Std. Conc	Conc.
1	1	guotang		5.07	118520.070		81.775021
2	2	putaotang		6.38	117710.836		83.466207

Name: 20240914_Wu_sample_097, Date: 14-Sep-2024, Time: 19:39:22, ID: , Description:

guotang

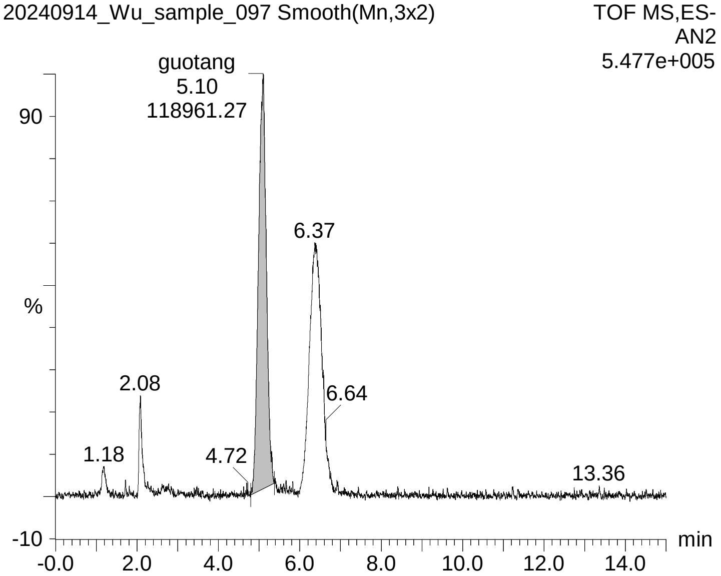

putaotang

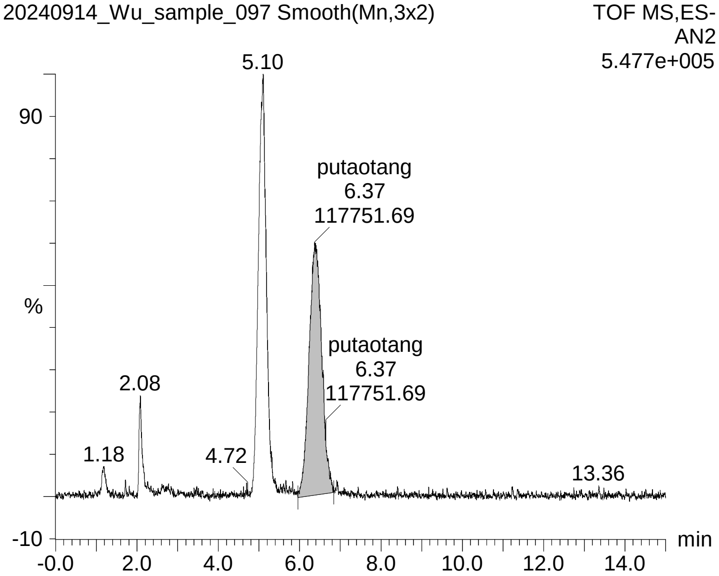

	#	Name	Sample Text	RT	Area	Std. Conc	Conc.
1	1	guotang		5.10	118961.273		82.026096
2	2	putaotang		6.37	117751.688		83.490116

project_wangzhonghua_BeiMu
Dataset: Untitled
Last Altered: Sunday, September 29, 2024 09:54:36 China Standard Time
Printed: Sunday, September 29, 2024 09:59:41 China Standard Time

Name: 20240914_Wu_sample_098, Date: 14-Sep-2024, Time: 19:55:24, ID: , Description:

guotang

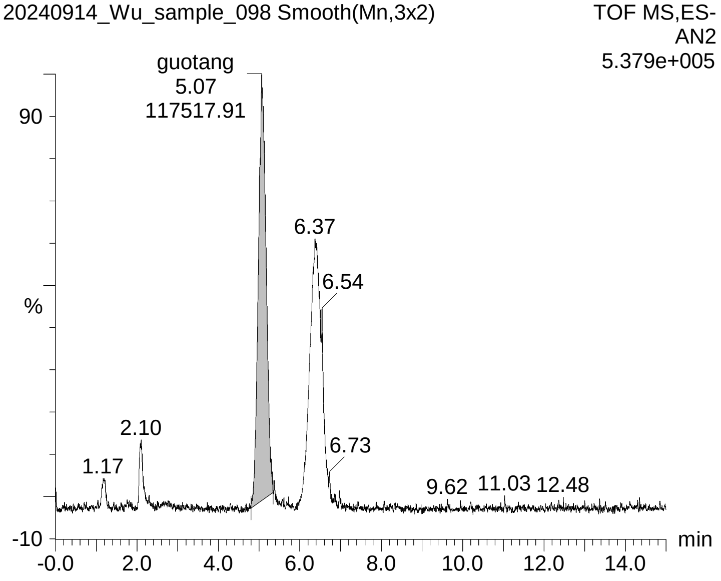

putaotang

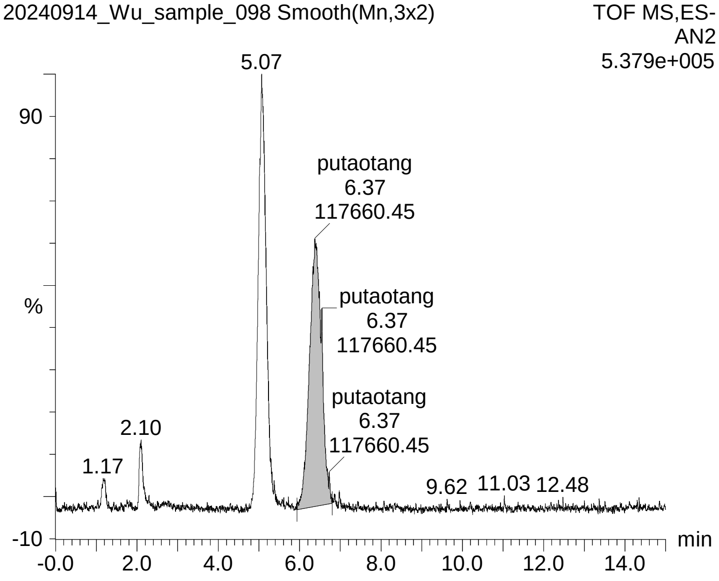

	#	Name	Sample Text	RT	Area	Std. Conc	Conc.
1	1	guotang		5.07	117517.906		81.204721
2	2	putaotang		6.37	117660.453		83.436720

project_wangzhonghua_BeiMu
Dataset: Untitled
Last Altered: Sunday, September 29, 2024 09:54:36 China Standard Time
Printed: Sunday, September 29, 2024 09:59:41 China Standard Time

Name: 20240914_Wu_sample_099, Date: 14-Sep-2024, Time: 20:11:26, ID: , Description:

guotang

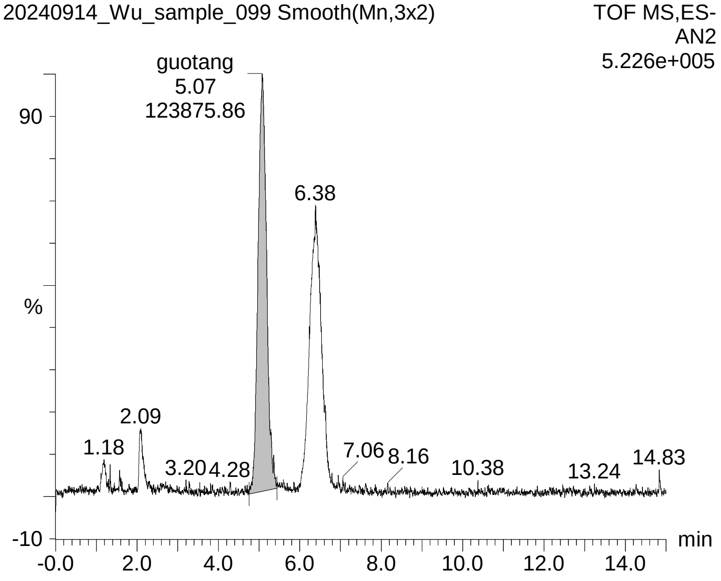

putaotang

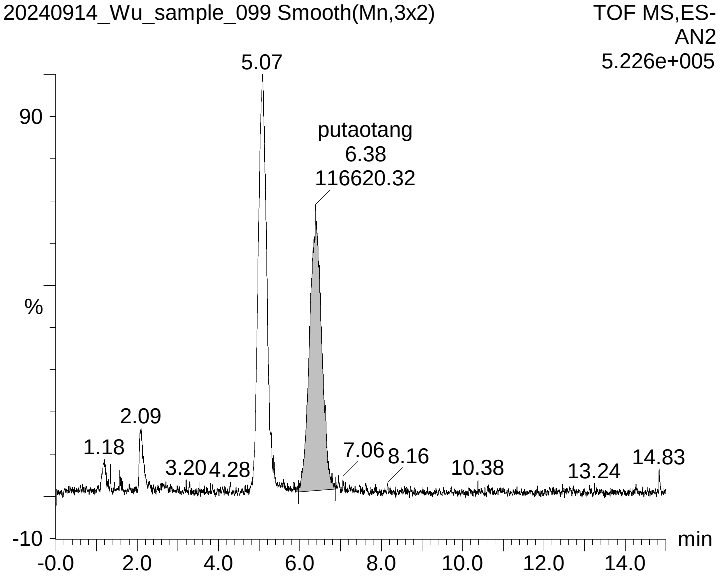

	#	Name	Sample Text	RT	Area	Std. Conc	Conc.
1	1	guotang		5.07	123875.859		84.822834
2	2	putaotang		6.38	116620.320		82.827966

project_wangzhonghua_BeiMu
Dataset: Untitled
Last Altered: Sunday, September 29, 2024 09:54:36 China Standard Time
Printed: Sunday, September 29, 2024 09:59:41 China Standard Time

Name: 20240914_Wu_sample_100, Date: 14-Sep-2024, Time: 20:27:29, ID: , Description:

guotang

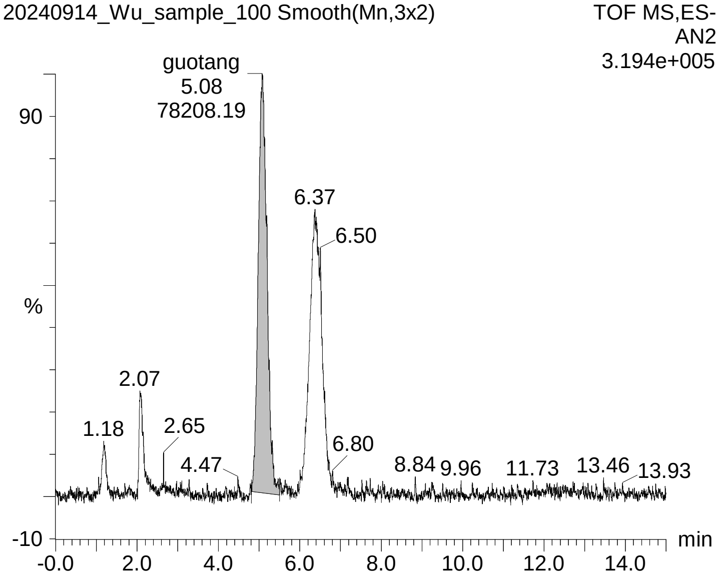

putaotang

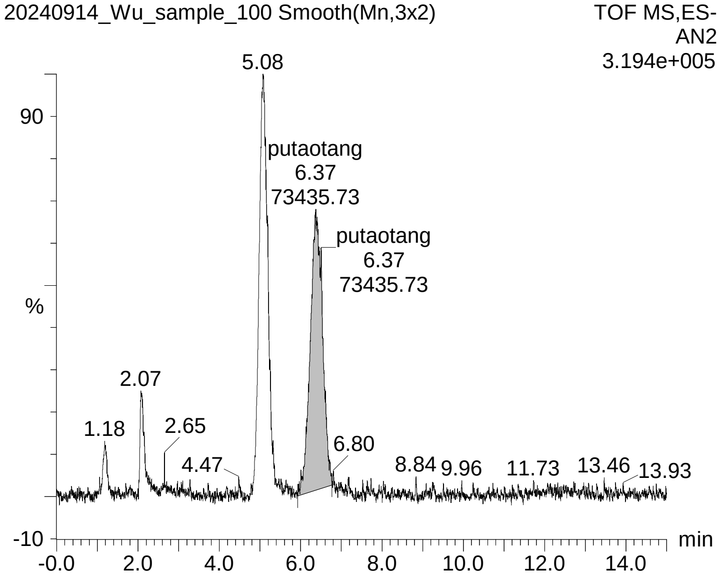

	#	Name	Sample Text	RT	Area	Std. Conc	Conc.
1	1	guotang		5.08	78208.188		58.834785
2	2	putaotang		6.37	73435.734		57.553537

project_wangzhonghua_BeiMu
Dataset: Untitled
Last Altered: Sunday, September 29, 2024 09:54:36 China Standard Time
Printed: Sunday, September 29, 2024 09:59:41 China Standard Time

Name: 20240914_Wu_sample_101, Date: 14-Sep-2024, Time: 20:43:25, ID: , Description:

guotang

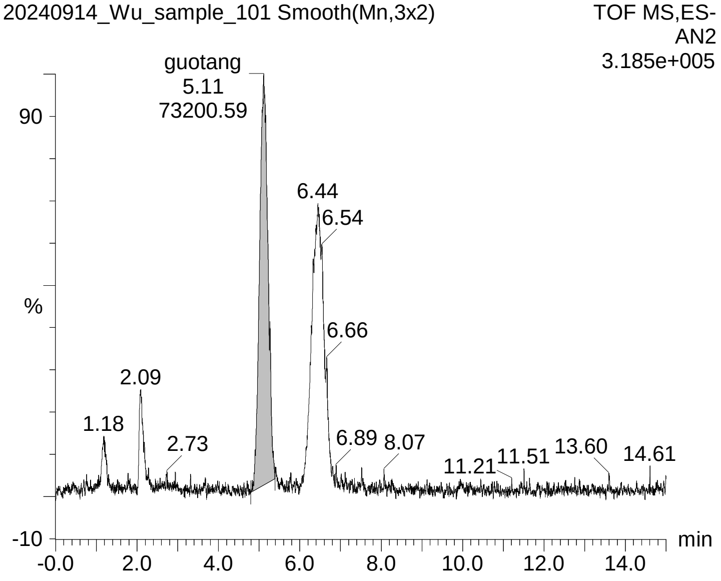

putaotang

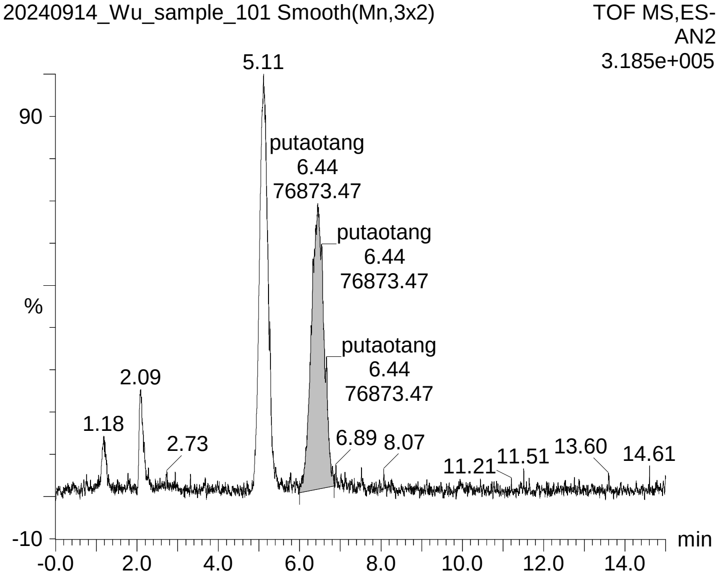

	#	Name	Sample Text	RT	Area	Std. Conc	Conc.
1	1	guotang		5.11	73200.586		55.985115
2	2	putaotang		6.44	76873.469		59.565523

Name: 20240914_Wu_sample_102, Date: 14-Sep-2024, Time: 20:59:25, ID: , Description:

guotang

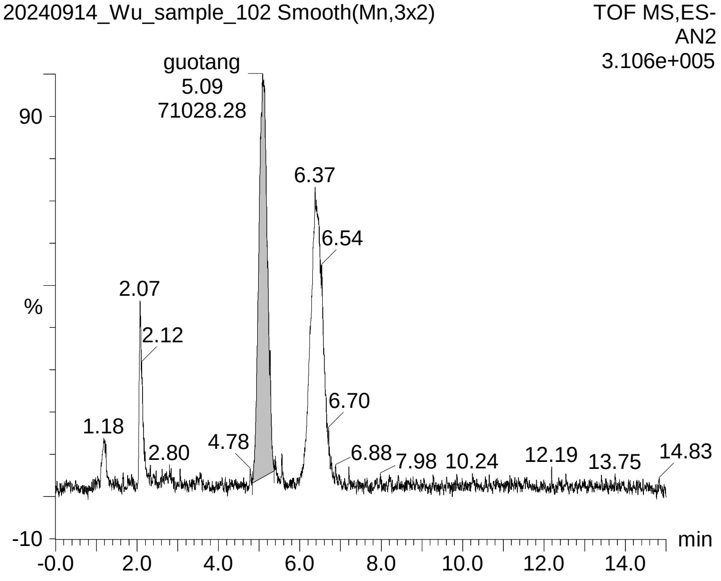

putaotang

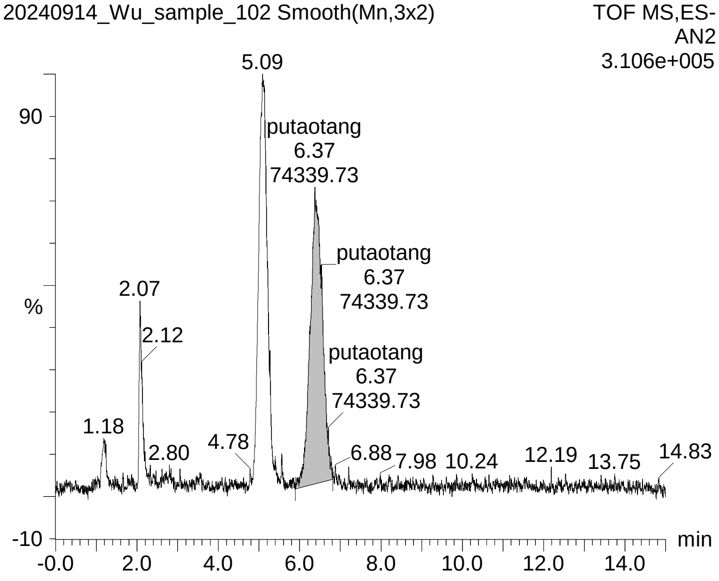

	#	Name	Sample Text	RT	Area	Std. Conc	Conc.
1	1	guotang		5.09	71028.281		54.748924
2	2	putaotang		6.37	74339.727		58.082613

project_wangzhonghua_BeiMu
Dataset: Untitled
Last Altered: Sunday, September 29, 2024 09:54:36 China Standard Time
Printed: Sunday, September 29, 2024 09:59:41 China Standard Time

Name: 20240914_Wu_sample_103, Date: 14-Sep-2024, Time: 21:15:27, ID: , Description:

guotang

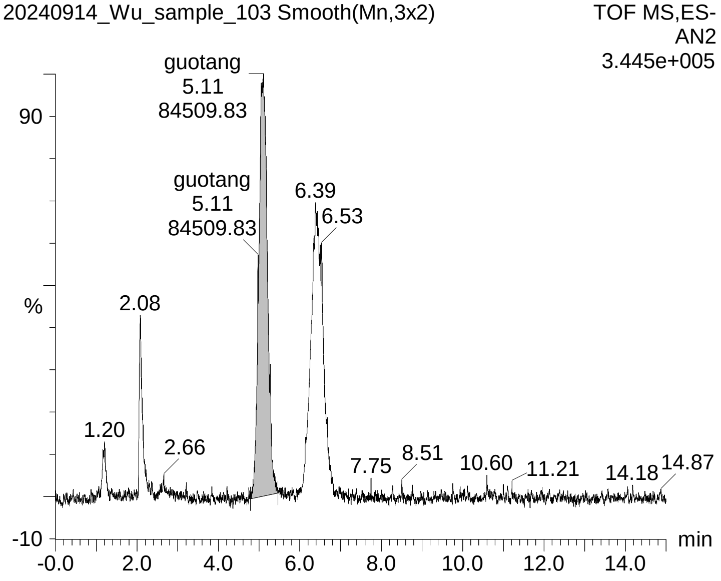

putaotang

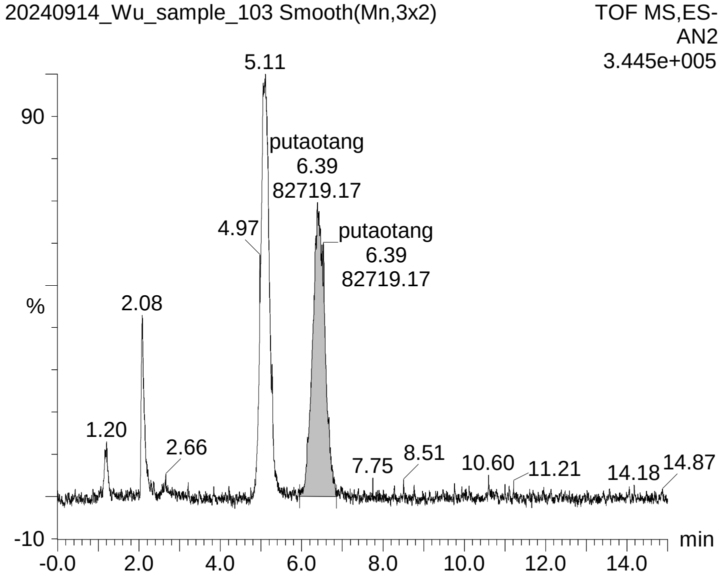

	#	Name	Sample Text	RT	Area	Std. Conc	Conc.
1	1	guotang		5.11	84509.828		62.420852
2	2	putaotang		6.39	82719.172		62.986809

project_wangzhonghua_BeiMu
Dataset: Untitled
Last Altered: Sunday, September 29, 2024 09:54:36 China Standard Time
Printed: Sunday, September 29, 2024 09:59:41 China Standard Time

Name: 20240914_Wu_sample_104, Date: 14-Sep-2024, Time: 21:31:28, ID: , Description:

guotang

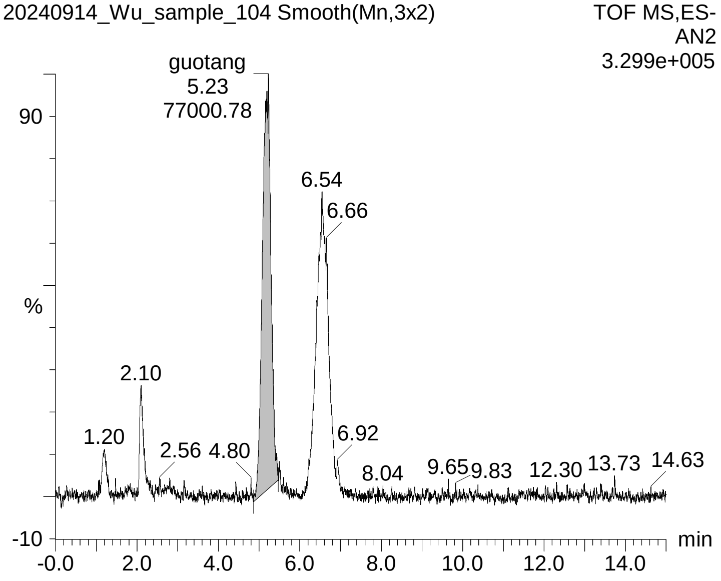

putaotang

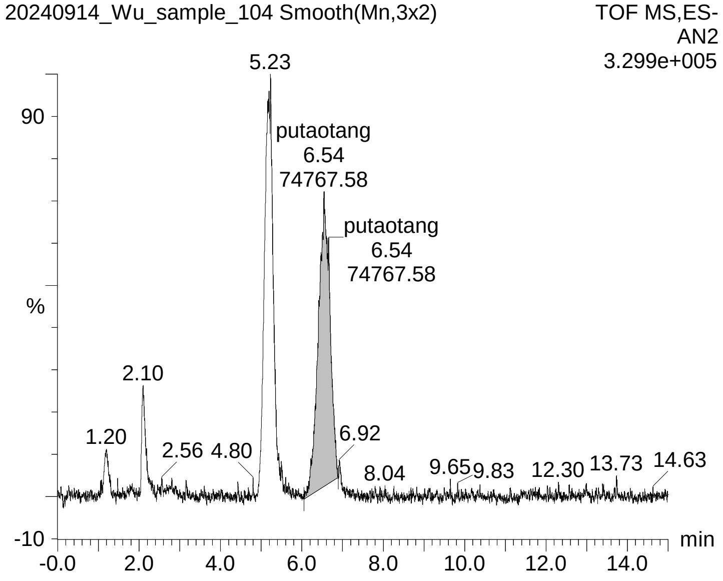

	#	Name	Sample Text	RT	Area	Std. Conc	Conc.
1	1	guotang		5.23	77000.781		58.147687
2	2	putaotang		6.54	74767.578		58.333019

Name: 20240914_Wu_sample_105, Date: 14-Sep-2024, Time: 21:47:30, ID: , Description:

guotang

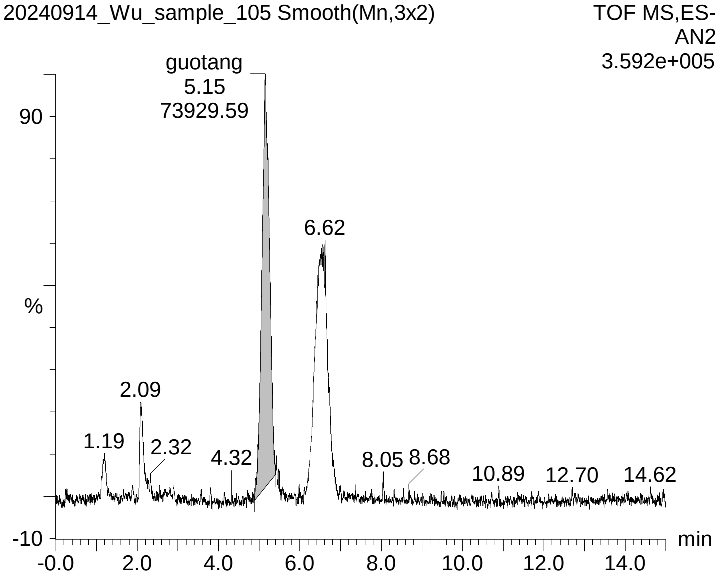

putaotang

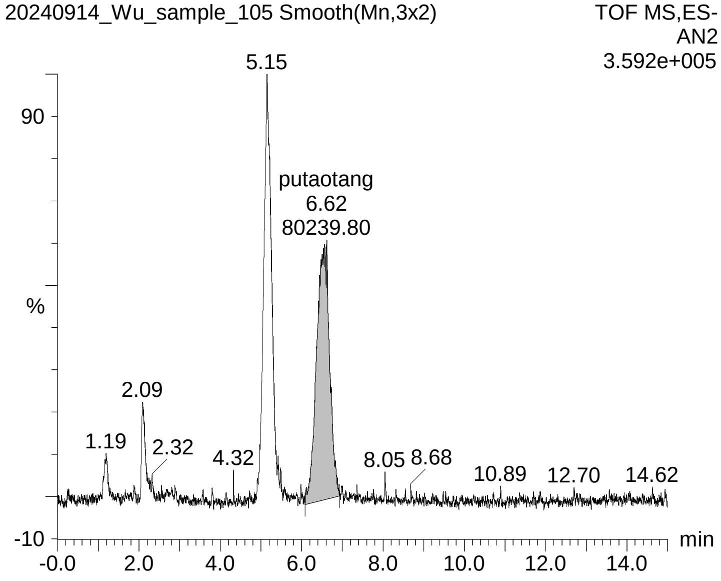

	#	Name	Sample Text	RT	Area	Std. Conc	Conc.
1	1	guotang		5.15	73929.586		56.399966
2	2	putaotang		6.62	80239.805		61.535722

project_wangzhonghua_BeiMu
Dataset: Untitled
Last Altered: Sunday, September 29, 2024 09:54:36 China Standard Time
Printed: Sunday, September 29, 2024 09:59:41 China Standard Time

Name: 20240914_Wu_sample_106, Date: 14-Sep-2024, Time: 22:03:32, ID: , Description:

guotang

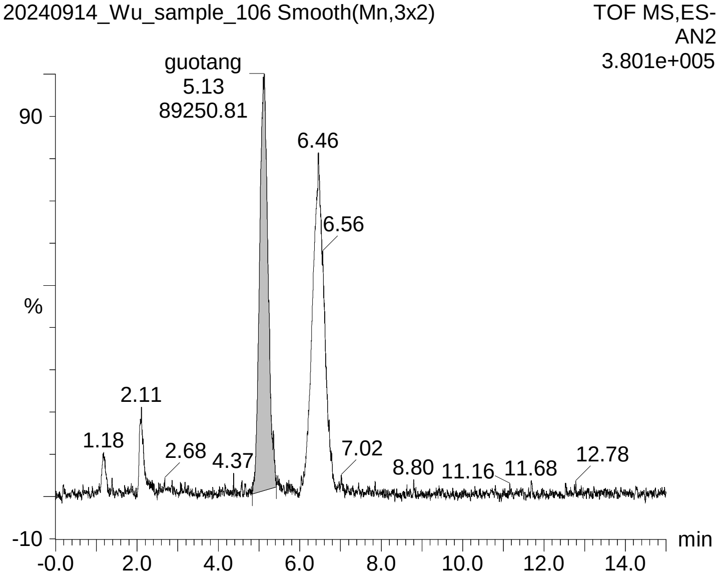

putaotang

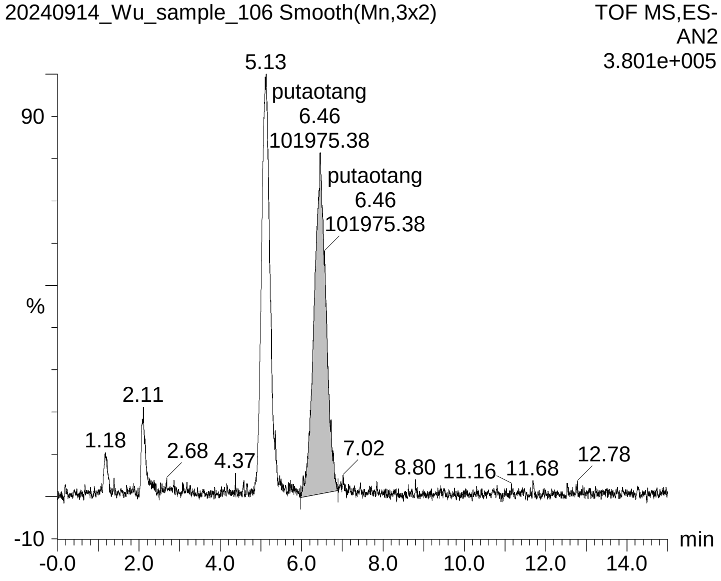

	#	Name	Sample Text	RT	Area	Std. Conc	Conc.
1	1	guotang		5.13	89250.813		65.118799
2	2	putaotang		6.46	101975.375		74.256792

project_wangzhonghua_BeiMu
Dataset: Untitled
Last Altered: Sunday, September 29, 2024 09:54:36 China Standard Time
Printed: Sunday, September 29, 2024 09:59:41 China Standard Time

Name: 20240914_Wu_sample_107, Date: 14-Sep-2024, Time: 22:19:34, ID: , Description:

guotang

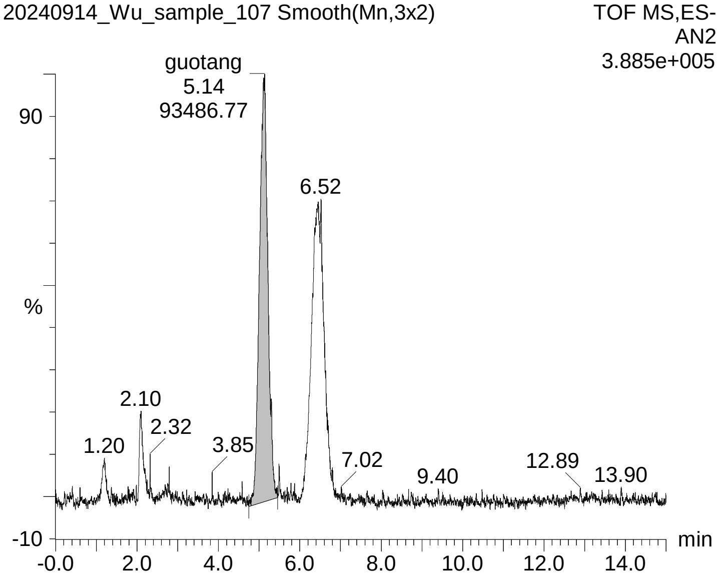

putaotang

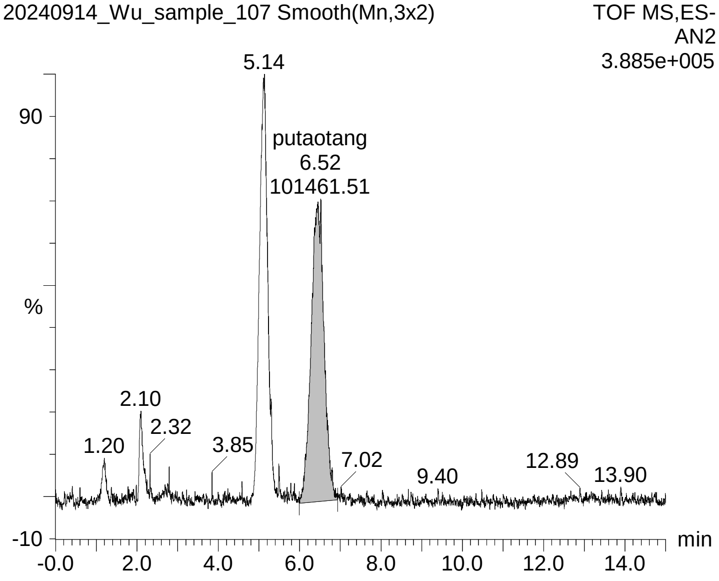

	#	Name	Sample Text	RT	Area	Std. Conc	Conc.
1	1	guotang		5.14	93486.773		67.529352
2	2	putaotang		6.52	101461.508		73.956043

project_wangzhonghua_BeiMu
Dataset: Untitled
Last Altered: Sunday, September 29, 2024 09:54:36 China Standard Time
Printed: Sunday, September 29, 2024 09:59:41 China Standard Time

Name: 20240914_Wu_sample_108, Date: 14-Sep-2024, Time: 22:35:37, ID: , Description:

guotang

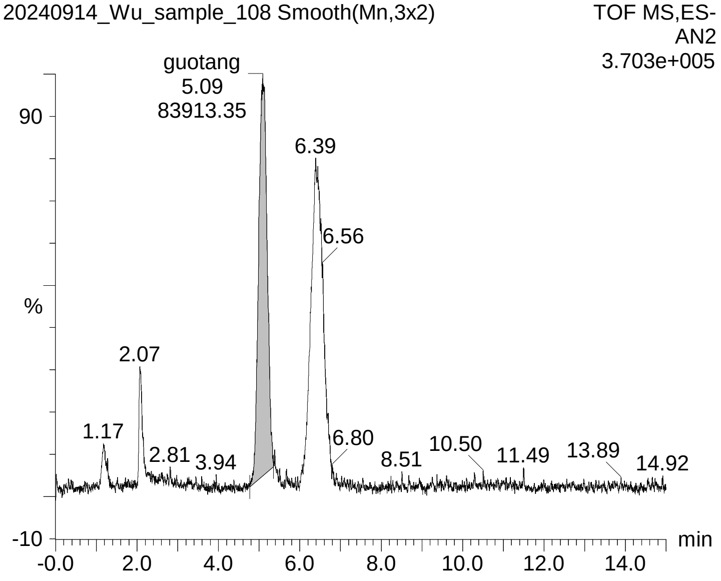

putaotang

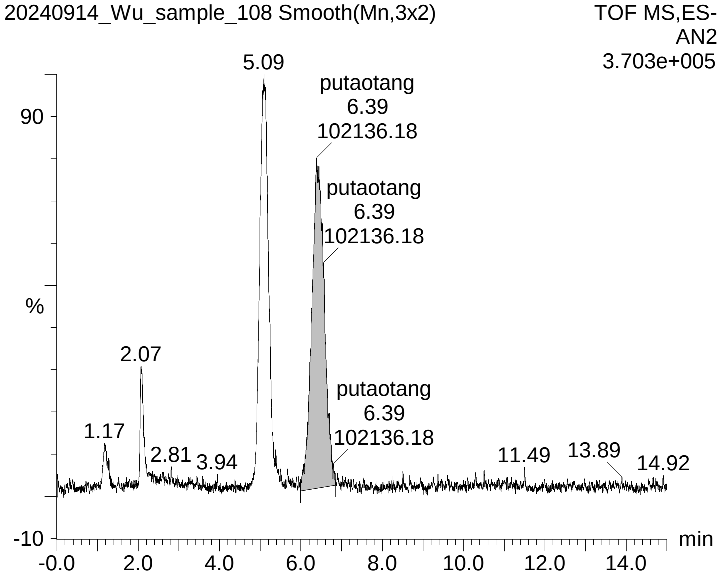

	#	Name	Sample Text	RT	Area	Std. Conc	Conc.
1	1	guotang		5.09	83913.352		62.081416
2	2	putaotang		6.39	102136.180		74.350905

Name: 20240914_Wu_sample_109, Date: 14-Sep-2024, Time: 22:51:34, ID: , Description:

guotang

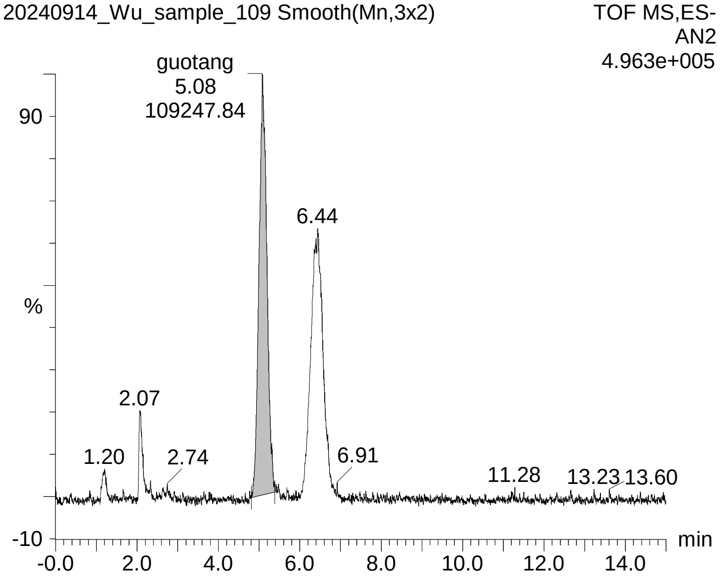

putaotang

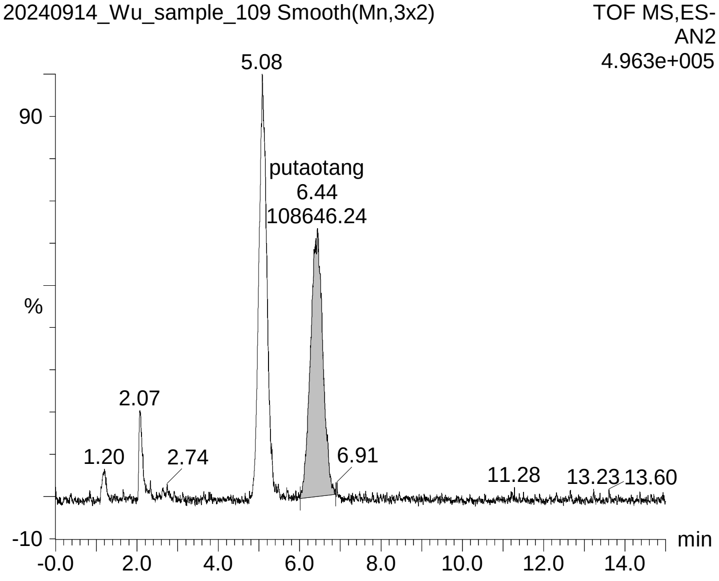

	#	Name	Sample Text	RT	Area	Std. Conc	Conc.
1	1	guotang		5.08	109247.836		76.498482
2	2	putaotang		6.44	108646.242		78.161017

project_wangzhonghua_BeiMu
Dataset: Untitled
Last Altered: Sunday, September 29, 2024 09:54:36 China Standard Time
Printed: Sunday, September 29, 2024 09:59:41 China Standard Time

Name: 20240914_Wu_sample_110, Date: 14-Sep-2024, Time: 23:07:32, ID: , Description:

guotang

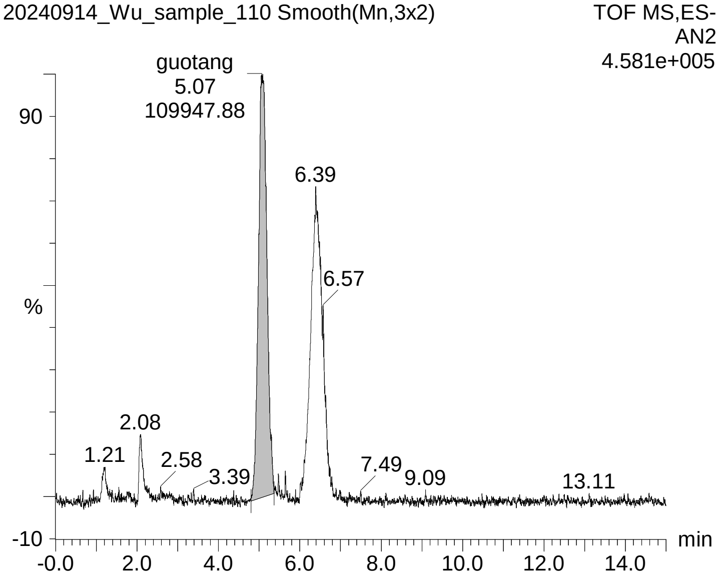

putaotang

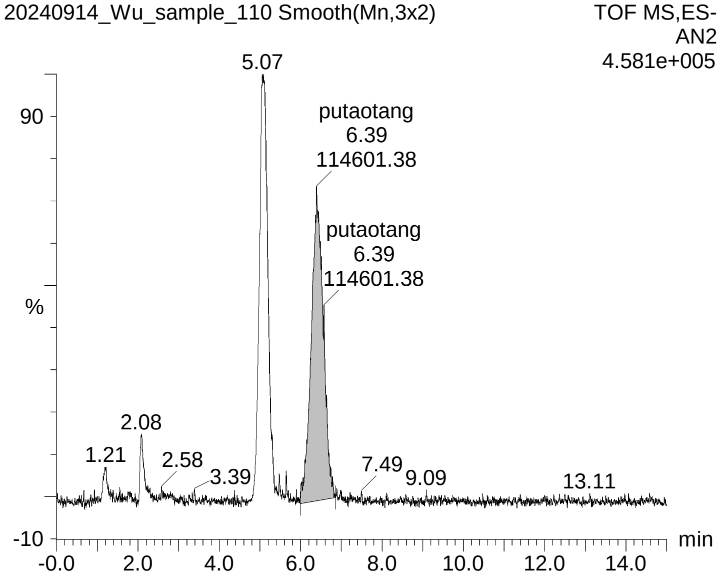

	#	Name	Sample Text	RT	Area	Std. Conc	Conc.
1	1	guotang		5.07	109947.875		76.896852
2	2	putaotang		6.39	114601.383		81.646353

project_wangzhonghua_BeiMu
Dataset: Untitled
Last Altered: Sunday, September 29, 2024 09:54:36 China Standard Time
Printed: Sunday, September 29, 2024 09:59:41 China Standard Time

Name: 20240914_Wu_sample_111, Date: 14-Sep-2024, Time: 23:23:34, ID: , Description:

guotang

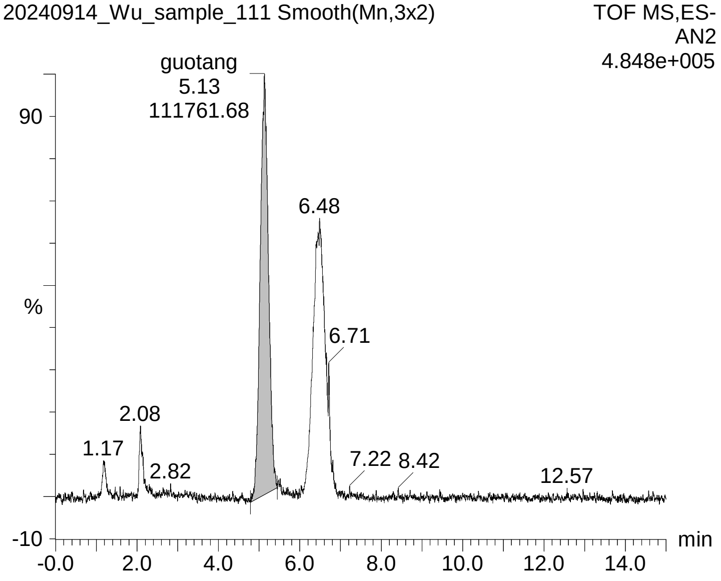

putaotang

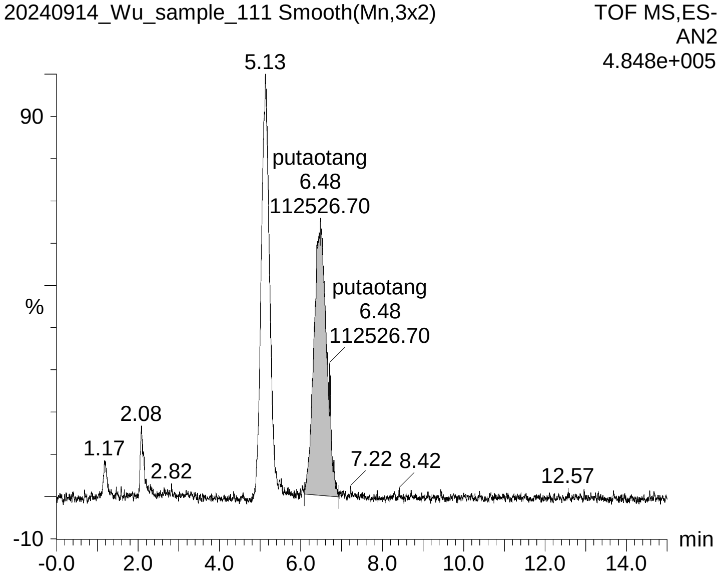

	#	Name	Sample Text	RT	Area	Std. Conc	Conc.
1	1	guotang		5.13	111761.680		77.929032
2	2	putaotang		6.48	112526.703		80.432115

project_wangzhonghua_BeiMu
Dataset: Untitled
Last Altered: Sunday, September 29, 2024 09:54:36 China Standard Time
Printed: Sunday, September 29, 2024 09:59:41 China Standard Time

Name: 20240914_Wu_sample_112, Date: 14-Sep-2024, Time: 23:39:37, ID: , Description:

guotang

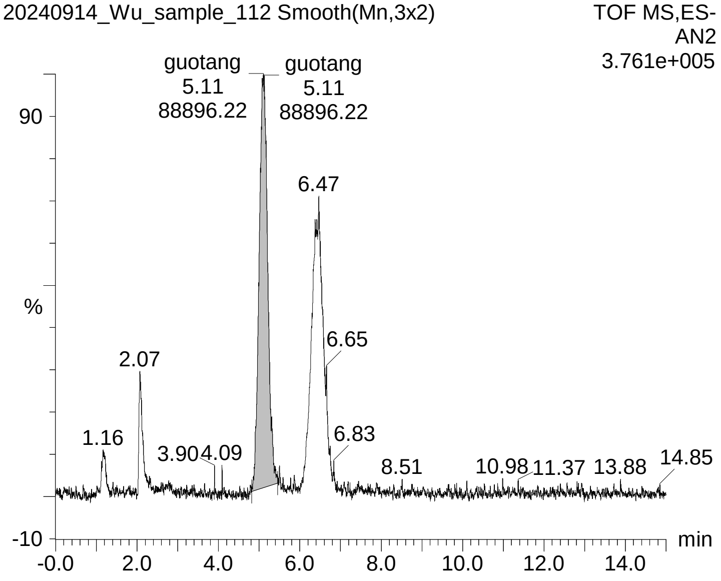

putaotang

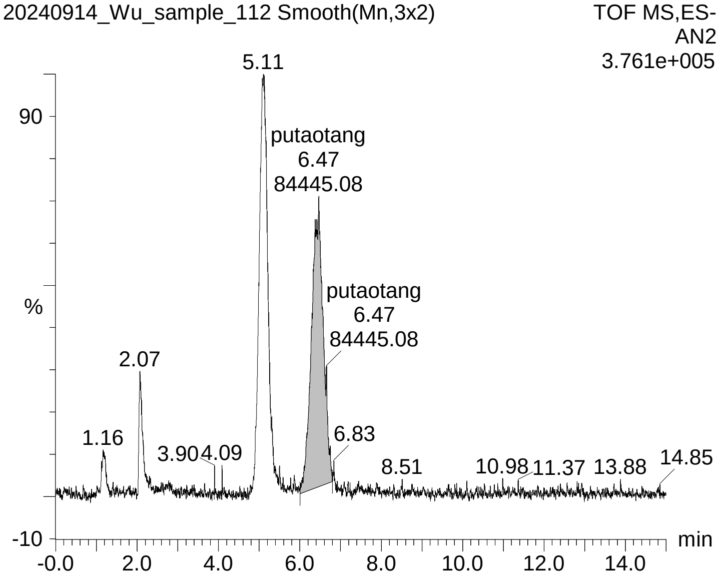

	#	Name	Sample Text	RT	Area	Std. Conc	Conc.
1	1	guotang		5.11	88896.219		64.917011
2	2	putaotang		6.47	84445.078		63.996921

project_wangzhonghua_BeiMu
Dataset: Untitled
Last Altered: Sunday, September 29, 2024 09:54:36 China Standard Time
Printed: Sunday, September 29, 2024 09:59:41 China Standard Time

Name: 20240914_Wu_sample_113, Date: 14-Sep-2024, Time: 23:55:39, ID: , Description:

guotang

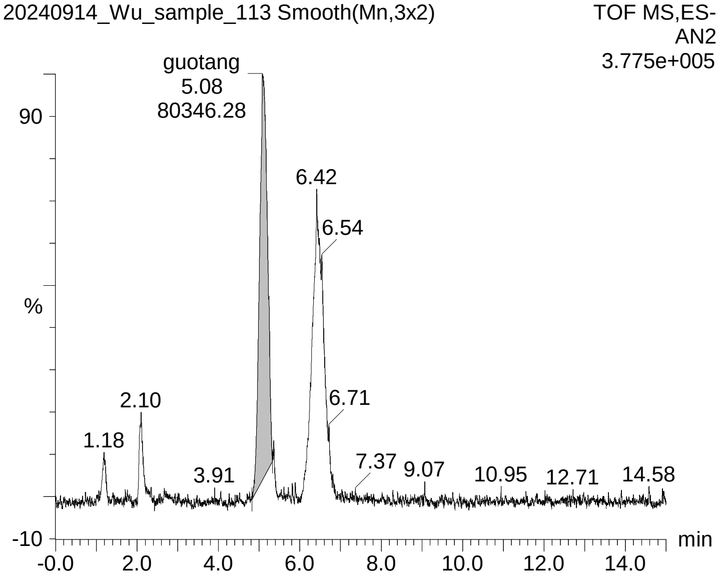

putaotang

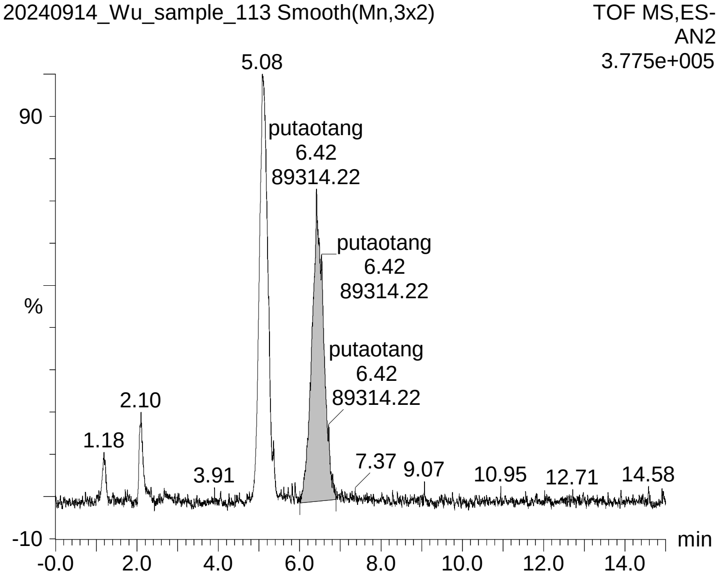

	#	Name	Sample Text	RT	Area	Std. Conc	Conc.
1	1	guotang		5.08	80346.281		60.051507
2	2	putaotang		6.42	89314.219		66.846659

project_wangzhonghua_BeiMu
Dataset: Untitled
Last Altered: Sunday, September 29, 2024 09:54:36 China Standard Time
Printed: Sunday, September 29, 2024 09:59:41 China Standard Time

Name: 20240914_Wu_sample_114, Date: 15-Sep-2024, Time: 00:11:41, ID: , Description:

guotang

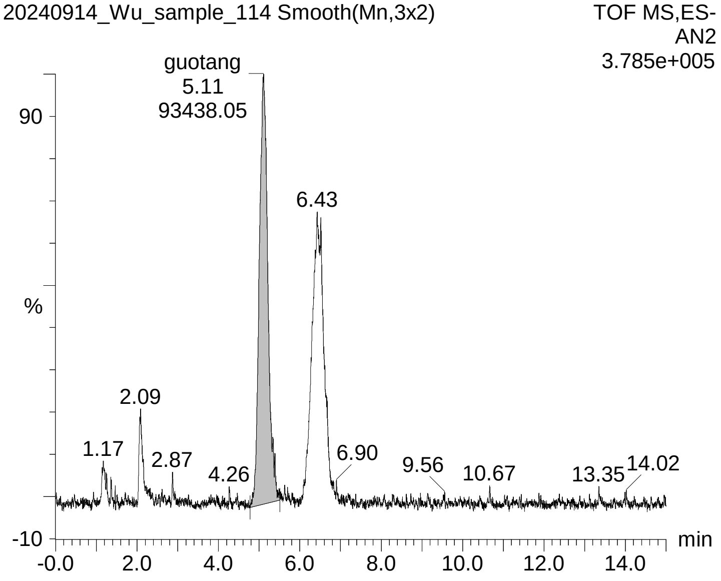

putaotang

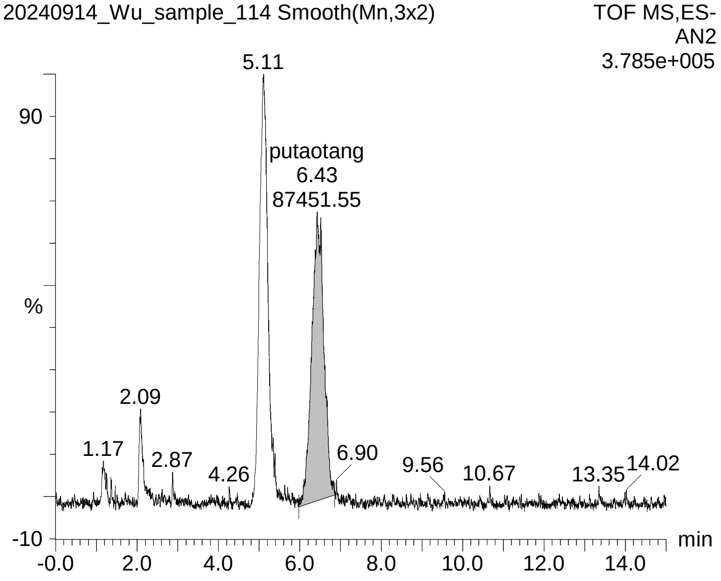

	#	Name	Sample Text	RT	Area	Std. Conc	Conc.
1	1	guotang		5.11	93438.055		67.501628
2	2	putaotang		6.43	87451.555		65.756507

project_wangzhonghua_BeiMu
Dataset: Untitled
Last Altered: Sunday, September 29, 2024 09:54:36 China Standard Time
Printed: Sunday, September 29, 2024 09:59:41 China Standard Time

Name: 20240914_Wu_sample_115, Date: 15-Sep-2024, Time: 00:27:42, ID: , Description:

guotang

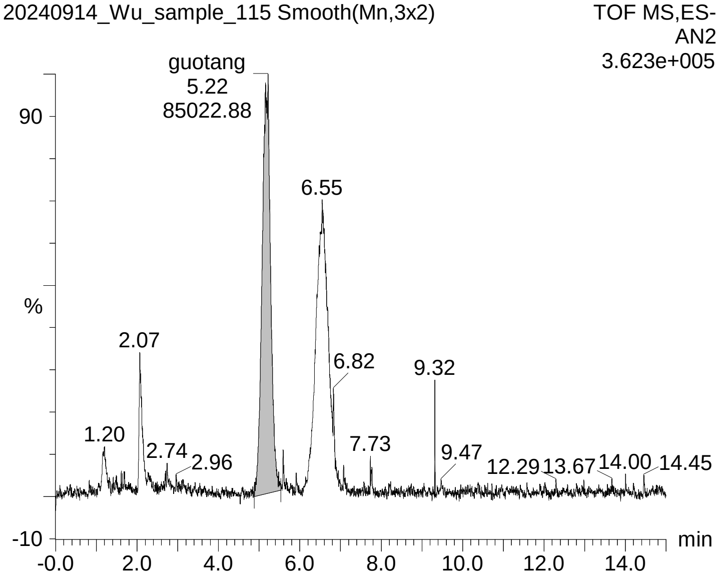

putaotang

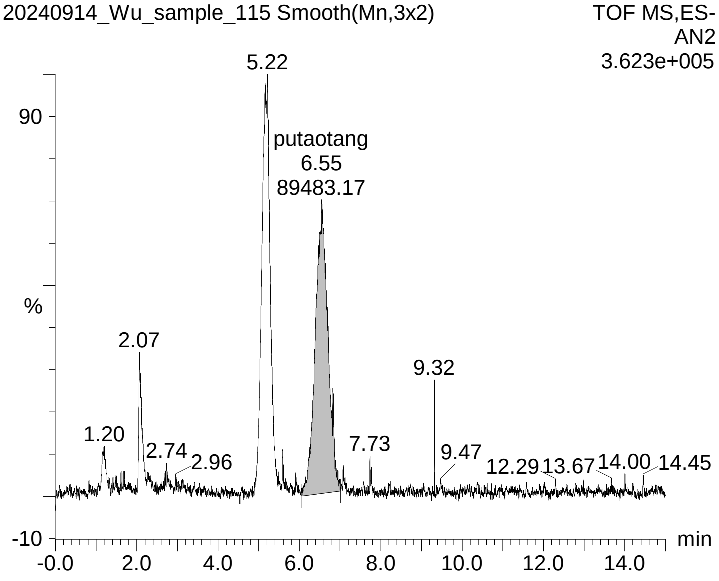

	#	Name	Sample Text	RT	Area	Std. Conc	Conc.
1	1	guotang		5.22	85022.883		62.712816
2	2	putaotang		6.55	89483.172		66.945542

project_wangzhonghua_BeiMu
Dataset: Untitled
Last Altered: Sunday, September 29, 2024 09:54:36 China Standard Time
Printed: Sunday, September 29, 2024 09:59:41 China Standard Time

Name: 20240914_Wu_sample_116, Date: 15-Sep-2024, Time: 00:43:45, ID: , Description:

guotang

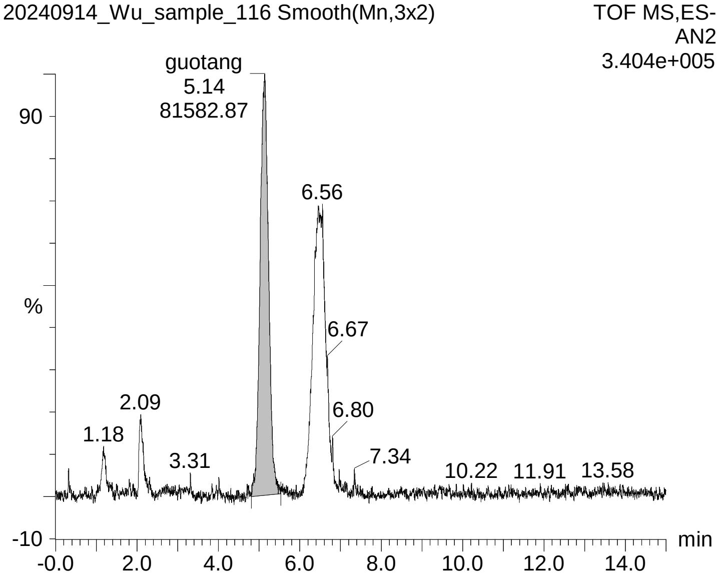

putaotang

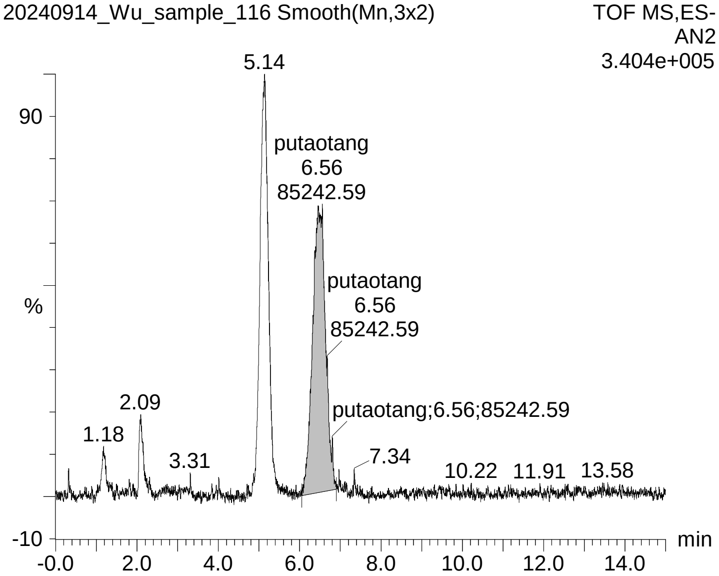

	#	Name	Sample Text	RT	Area	Std. Conc	Conc.
1	1	guotang		5.14	81582.867		60.755210
2	2	putaotang		6.56	85242.586		64.463675

project_wangzhonghua_BeiMu
Dataset: Untitled
Last Altered: Sunday, September 29, 2024 09:54:36 China Standard Time
Printed: Sunday, September 29, 2024 09:59:41 China Standard Time

Name: 20240914_Wu_sample_117, Date: 15-Sep-2024, Time: 00:59:46, ID: , Description:

guotang

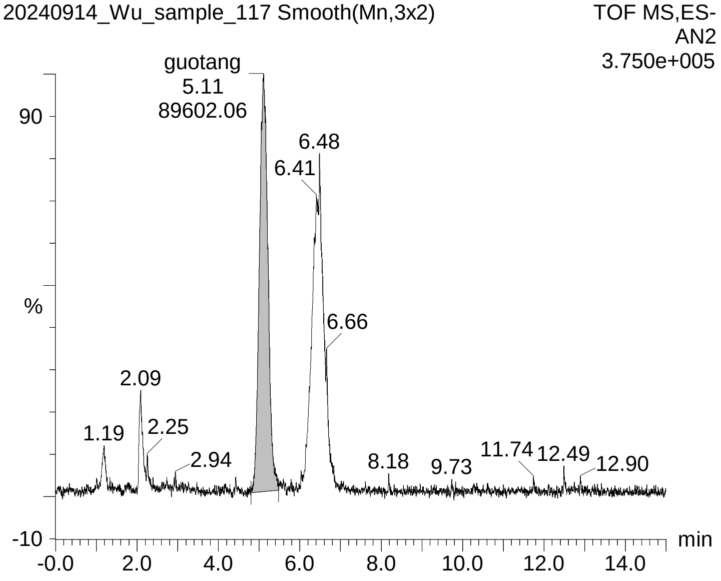

putaotang

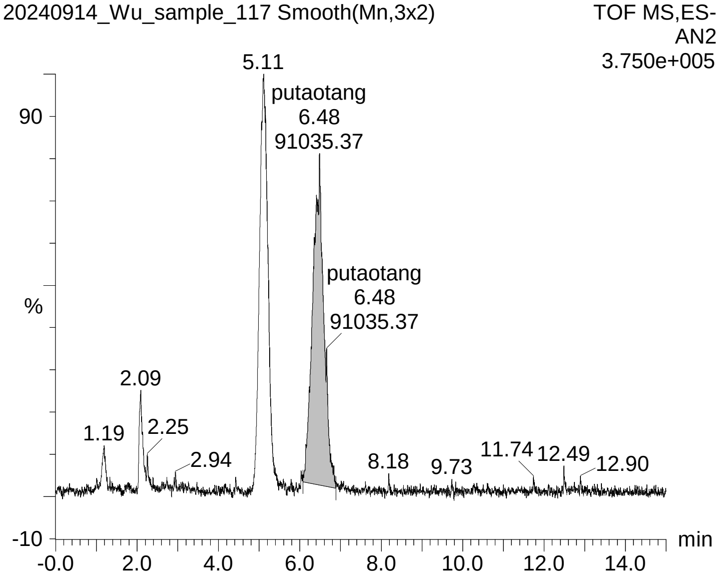

	#	Name	Sample Text	RT	Area	Std. Conc	Conc.
1	1	guotang		5.11	89602.063		65.318684
2	2	putaotang		6.48	91035.367		67.853987

project_wangzhonghua_BeiMu
Dataset: Untitled
Last Altered: Sunday, September 29, 2024 09:54:36 China Standard Time
Printed: Sunday, September 29, 2024 09:59:41 China Standard Time

Name: 20240914_Wu_sample_118, Date: 15-Sep-2024, Time: 01:15:48, ID: , Description:

guotang

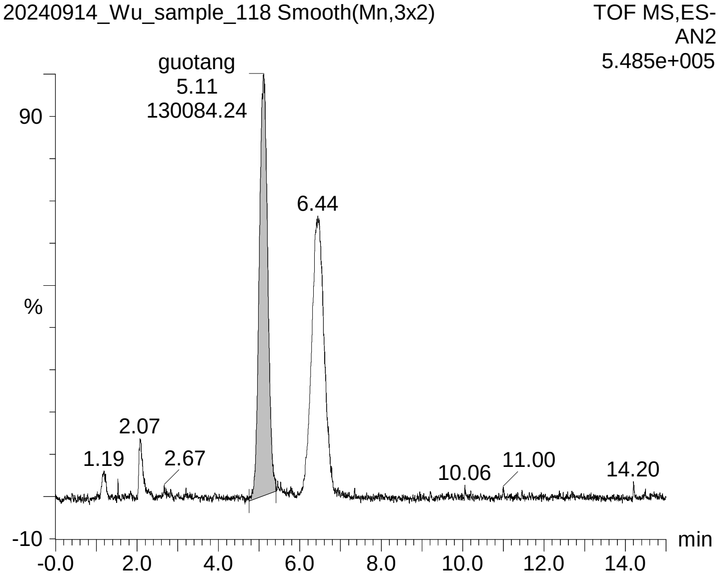

putaotang

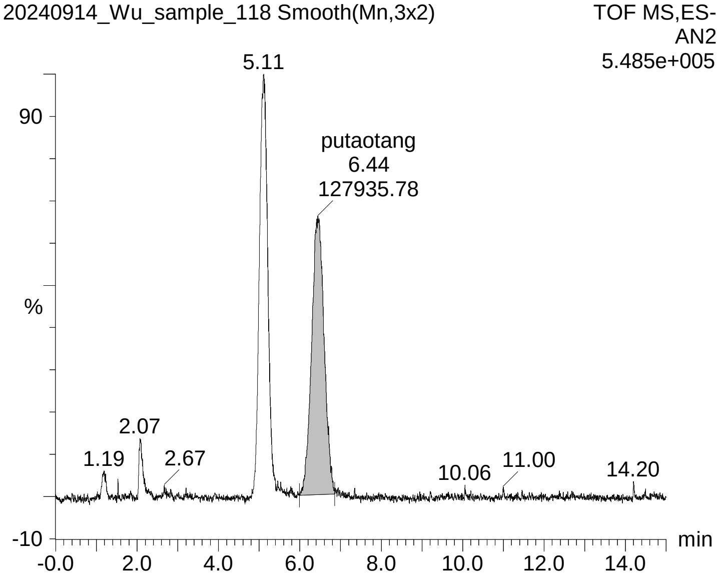

	#	Name	Sample Text	RT	Area	Std. Conc	Conc.
1	1	guotang		5.11	130084.242		88.355831
2	2	putaotang		6.44	127935.781		89.450510

project_wangzhonghua_BeiMu
Dataset: Untitled
Last Altered: Sunday, September 29, 2024 09:54:36 China Standard Time
Printed: Sunday, September 29, 2024 09:59:41 China Standard Time

Name: 20240914_Wu_sample_119, Date: 15-Sep-2024, Time: 01:31:50, ID: , Description:

guotang

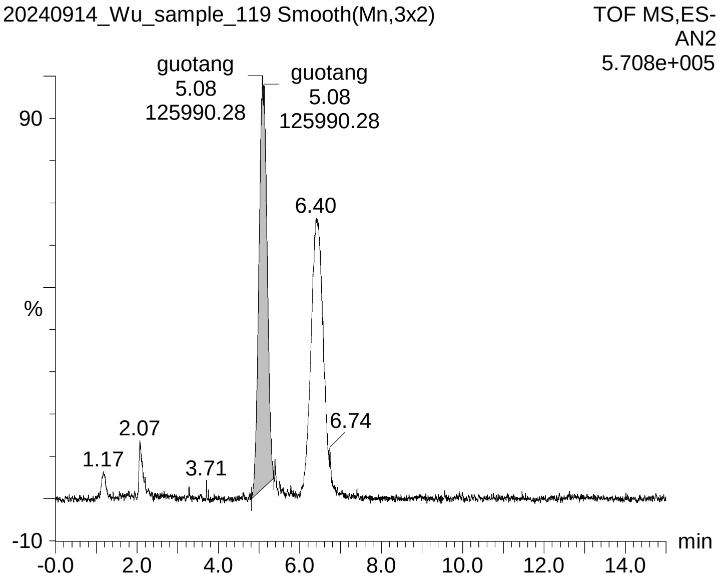

putaotang

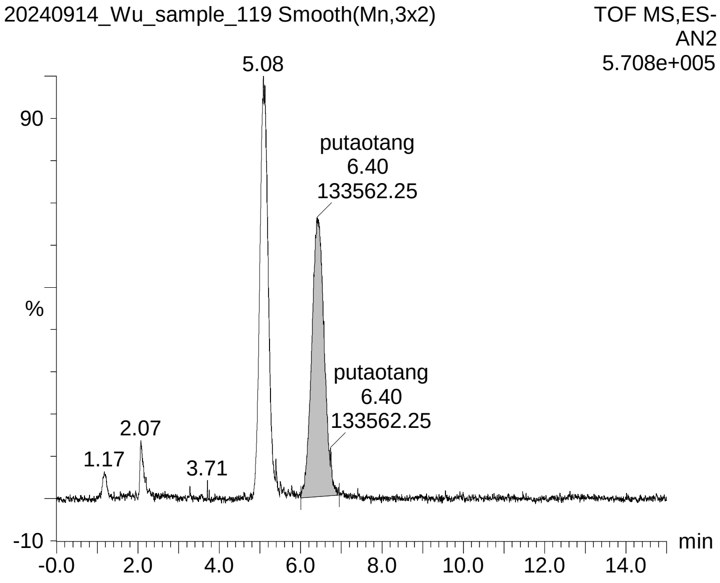

	#	Name	Sample Text	RT	Area	Std. Conc	Conc.
1	1	guotang		5.08	125990.281		86.026086
2	2	putaotang		6.40	133562.250		92.743486

project_wangzhonghua_BeiMu
Dataset: Untitled
Last Altered: Sunday, September 29, 2024 09:54:36 China Standard Time
Printed: Sunday, September 29, 2024 09:59:41 China Standard Time

Name: 20240914_Wu_sample_120, Date: 15-Sep-2024, Time: 01:47:51, ID: , Description:

guotang **putaotang**

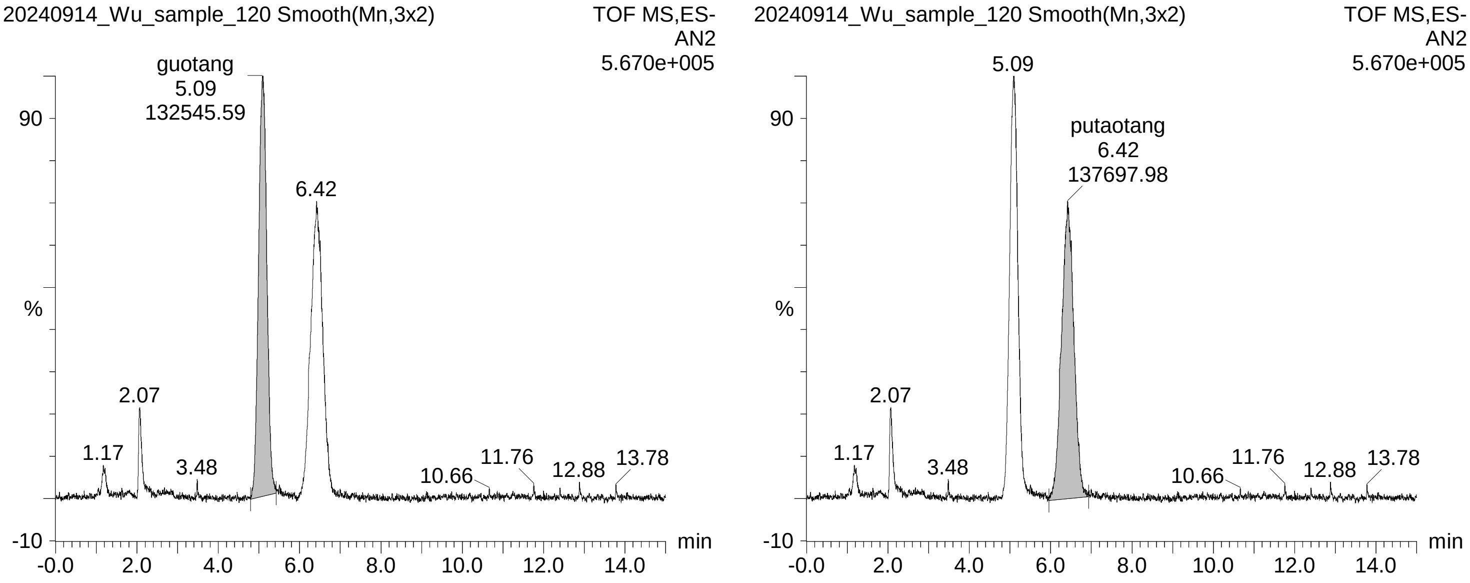

	#	Name	Sample Text	RT	Area	Std. Conc	Conc.
1	1	guotang		5.09	132545.594		89.756510
2	2	putaotang		6.42	137697.984		95.163986

project_wangzhonghua_BeiMu
Dataset: Untitled
Last Altered: Sunday, September 29, 2024 09:54:36 China Standard Time
Printed: Sunday, September 29, 2024 09:59:41 China Standard Time

Name: 20240914_Wu_sample_121, Date: 15-Sep-2024, Time: 02:03:53, ID: , Description:

guotang

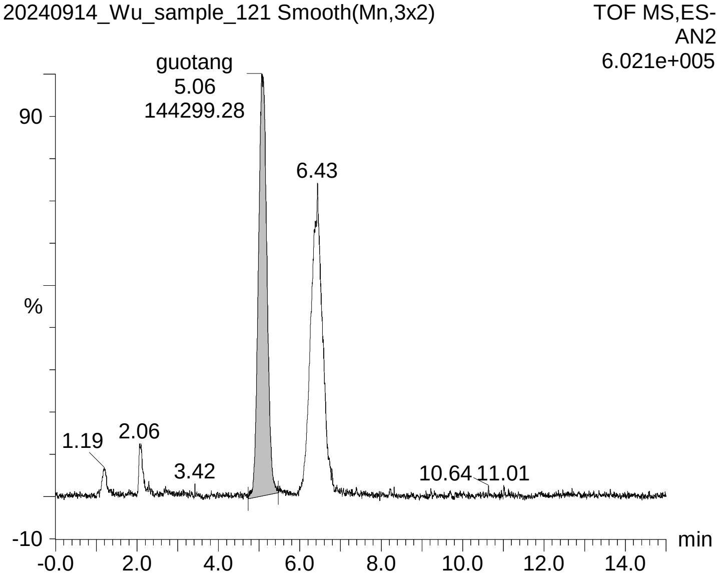

putaotang

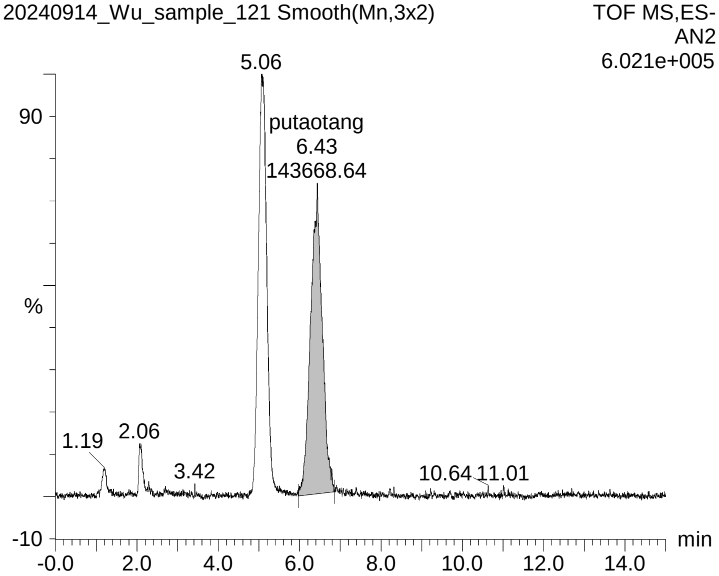

	#	Name	Sample Text	RT	Area	Std. Conc	Conc.
1	1	guotang		5.06	144299.281		96.445167
2	2	putaotang		6.43	143668.641		98.658403

Name: 20240914_Wu_sample_122, Date: 15-Sep-2024, Time: 02:19:55, ID: , Description:

guotang

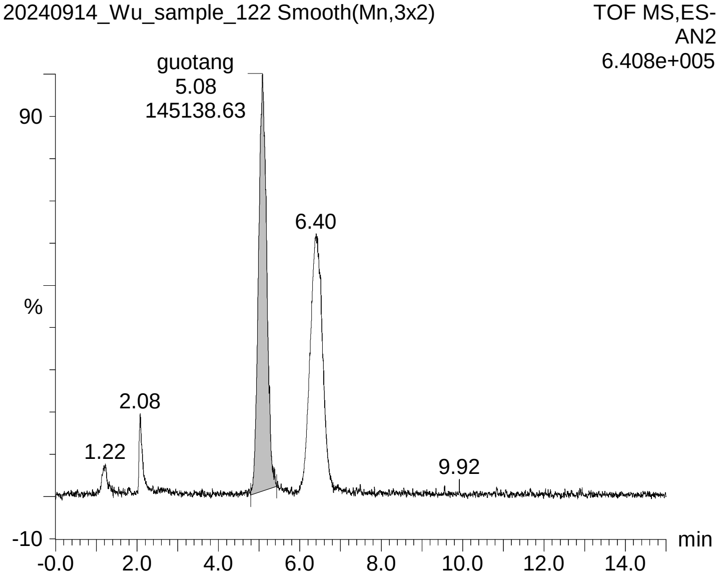

putaotang

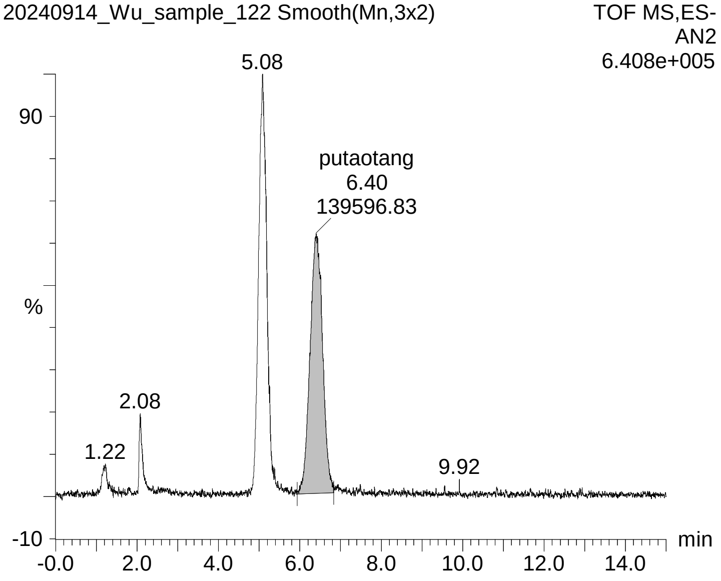

	#	Name	Sample Text	RT	Area	Std. Conc	Conc.
1	1	guotang		5.08	145138.625		96.922812
2	2	putaotang		6.40	139596.828		96.275313

Name: 20240914_Wu_sample_123, Date: 15-Sep-2024, Time: 02:35:57, ID: , Description:

guotang

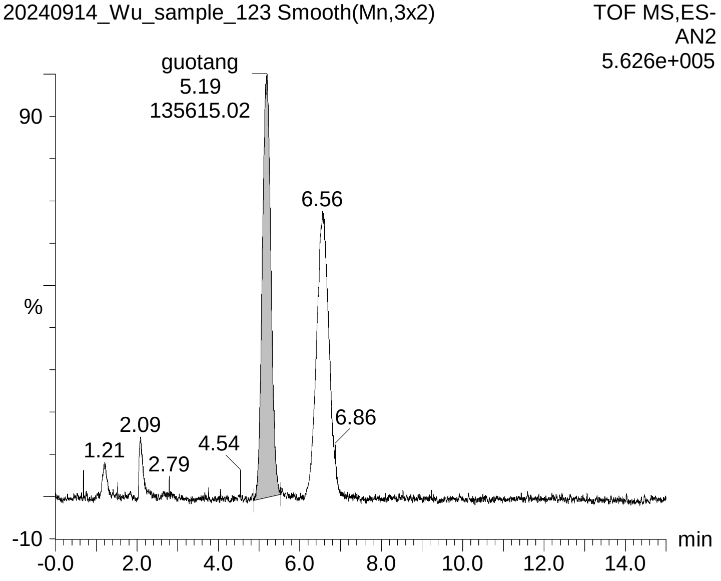

putaotang

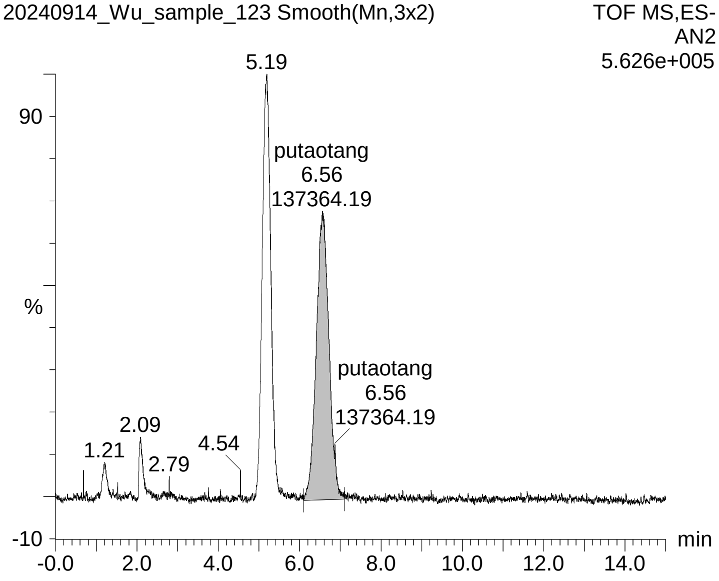

	#	Name	Sample Text	RT	Area	Std. Conc	Conc.
1	1	guotang		5.19	135615.016		91.503222
2	2	putaotang		6.56	137364.188		94.968627

project_wangzhonghua_BeiMu
Dataset: Untitled
Last Altered: Sunday, September 29, 2024 09:54:36 China Standard Time
Printed: Sunday, September 29, 2024 09:59:41 China Standard Time

Name: 20240914_Wu_sample_124, Date: 15-Sep-2024, Time: 02:52:00, ID: , Description:

guotang

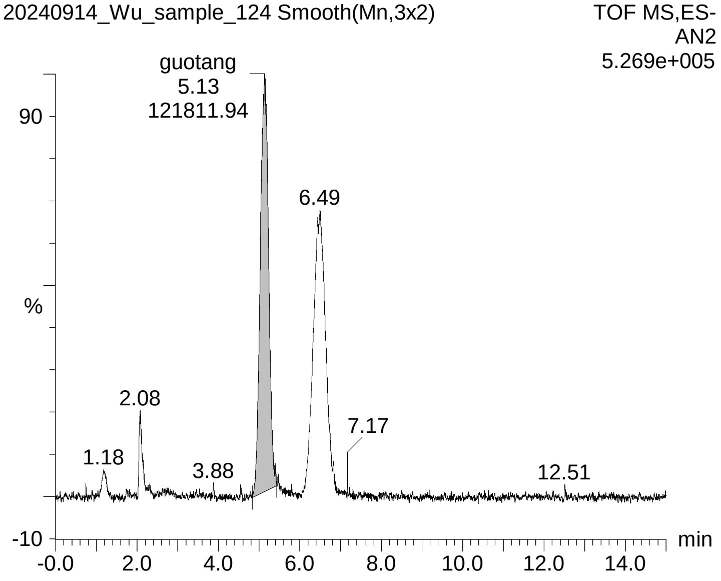

putaotang

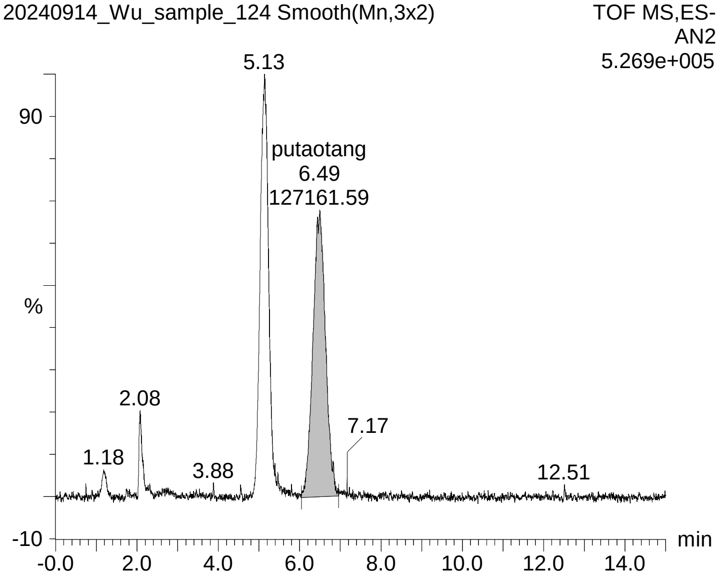

	#	Name	Sample Text	RT	Area	Std. Conc	Conc.
1	1	guotang		5.13	121811.938		83.648321
2	2	putaotang		6.49	127161.594		88.997405

project_wangzhonghua_BeiMu
Dataset: Untitled
Last Altered: Sunday, September 29, 2024 09:54:36 China Standard Time
Printed: Sunday, September 29, 2024 09:59:41 China Standard Time

Name: 20240914_Wu_sample_125, Date: 15-Sep-2024, Time: 03:08:02, ID: , Description:

guotang

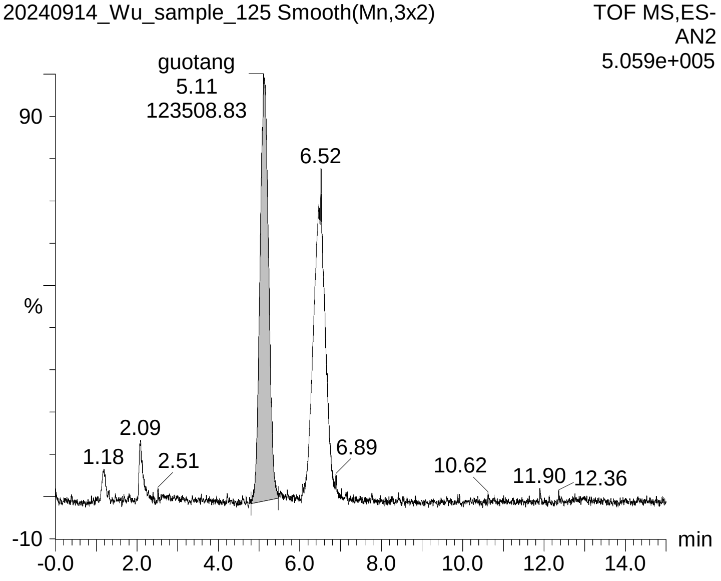

putaotang

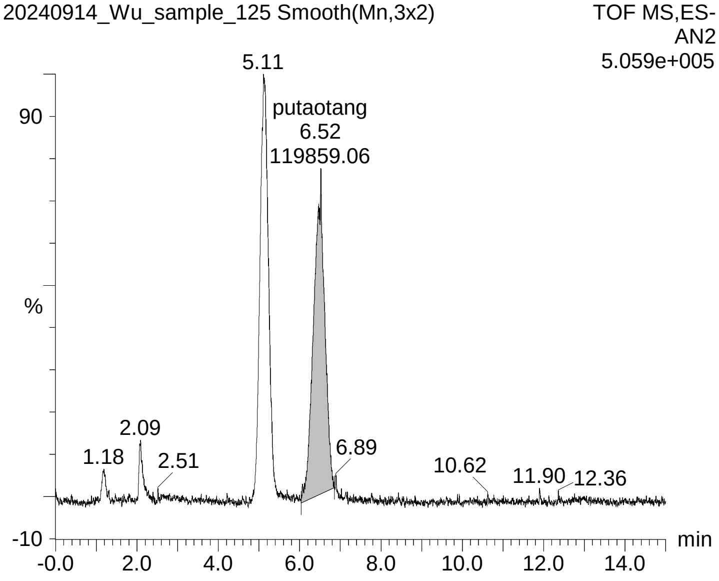

	#	Name	Sample Text	RT	Area	Std. Conc	Conc.
1	1	guotang		5.11	123508.828		84.613968
2	2	putaotang		6.52	119859.063		84.723489

Name: 20240914_Wu_sample_126, Date: 15-Sep-2024, Time: 03:23:59, ID: , Description:

guotang

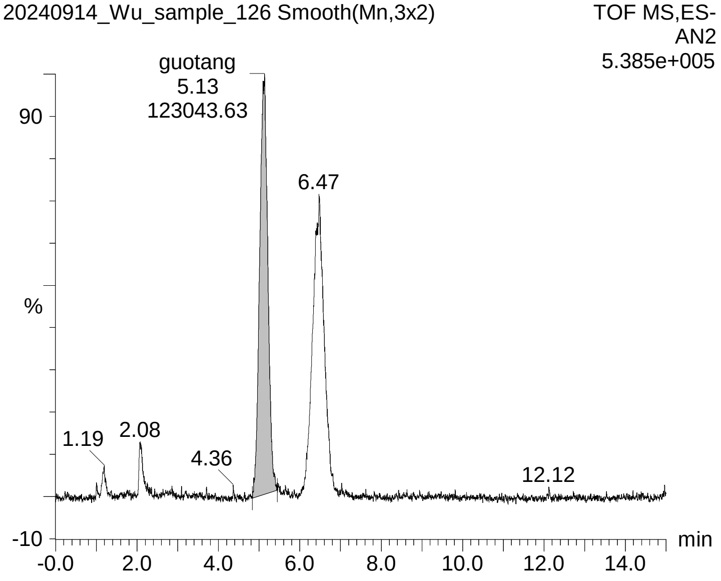

putaotang

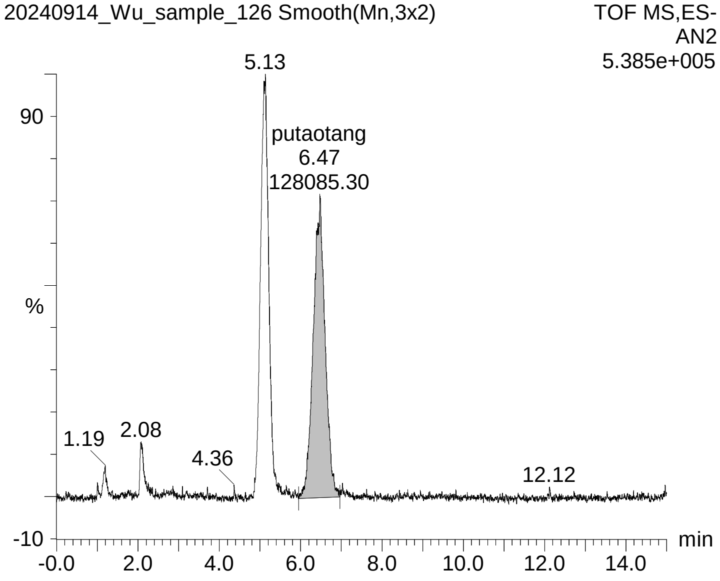

	#	Name	Sample Text	RT	Area	Std. Conc	Conc.
1	1	guotang		5.13	123043.633		84.349240
2	2	putaotang		6.47	128085.297		89.538016

project_wangzhonghua_BeiMu
Dataset: Untitled
Last Altered: Sunday, September 29, 2024 09:54:36 China Standard Time
Printed: Sunday, September 29, 2024 09:59:41 China Standard Time

Name: 20240914_Wu_sample_127, Date: 15-Sep-2024, Time: 03:39:57, ID: , Description:

guotang

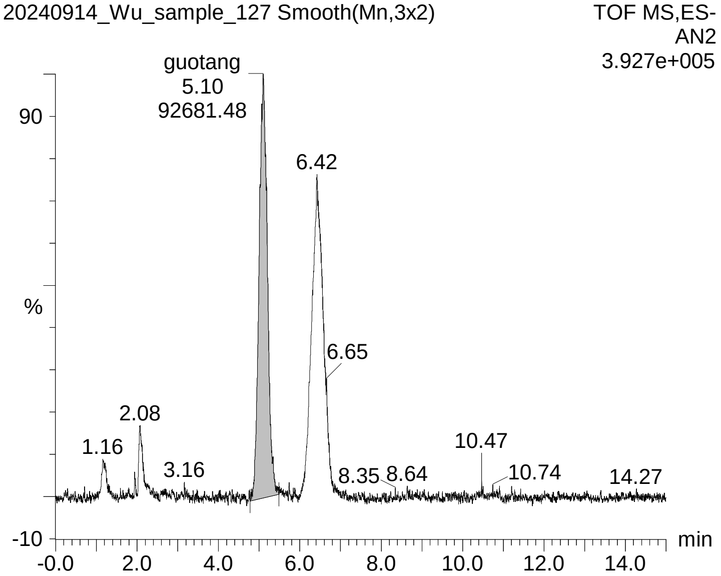

putaotang

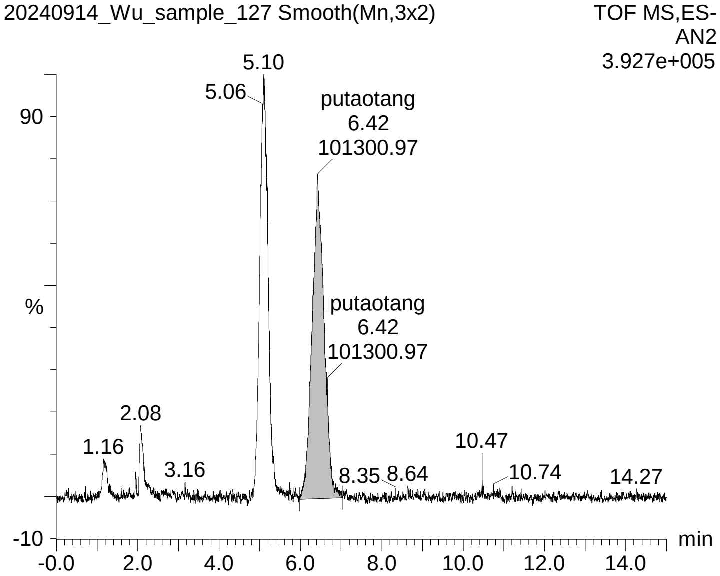

	#	Name	Sample Text	RT	Area	Std. Conc	Conc.
1	1	guotang		5.10	92681.477		67.071083
2	2	putaotang		6.42	101300.969		73.862085

project_wangzhonghua_BeiMu
Dataset: Untitled
Last Altered: Sunday, September 29, 2024 09:54:36 China Standard Time
Printed: Sunday, September 29, 2024 09:59:41 China Standard Time

Name: 20240914_Wu_sample_128, Date: 15-Sep-2024, Time: 03:55:59, ID: , Description:

guotang

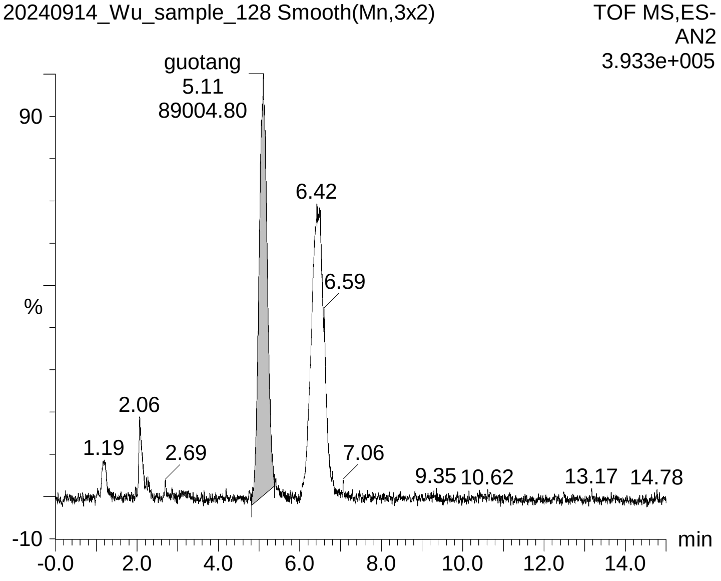

putaotang

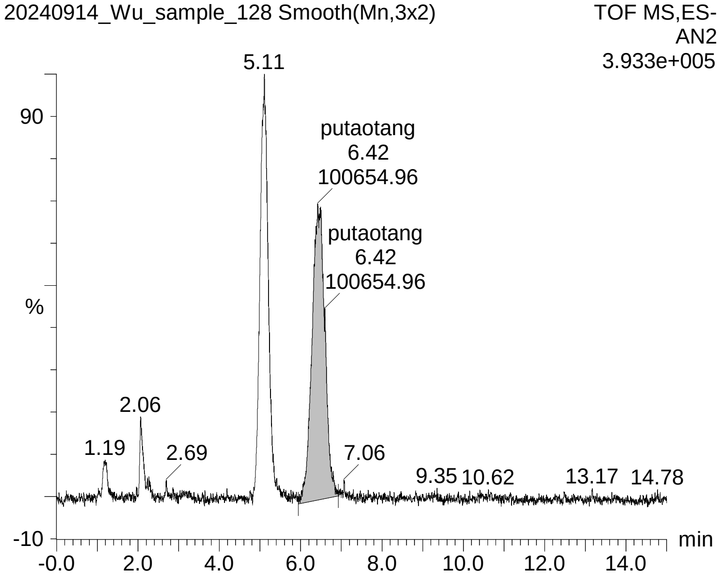

	#	Name	Sample Text	RT	Area	Std. Conc	Conc.
1	1	guotang		5.11	89004.805		64.978803
2	2	putaotang		6.42	100654.961		73.483999

project_wangzhonghua_BeiMu
Dataset: Untitled
Last Altered: Sunday, September 29, 2024 09:54:36 China Standard Time
Printed: Sunday, September 29, 2024 09:59:41 China Standard Time

Name: 20240914_Wu_sample_129, Date: 15-Sep-2024, Time: 04:12:01, ID: , Description:

guotang

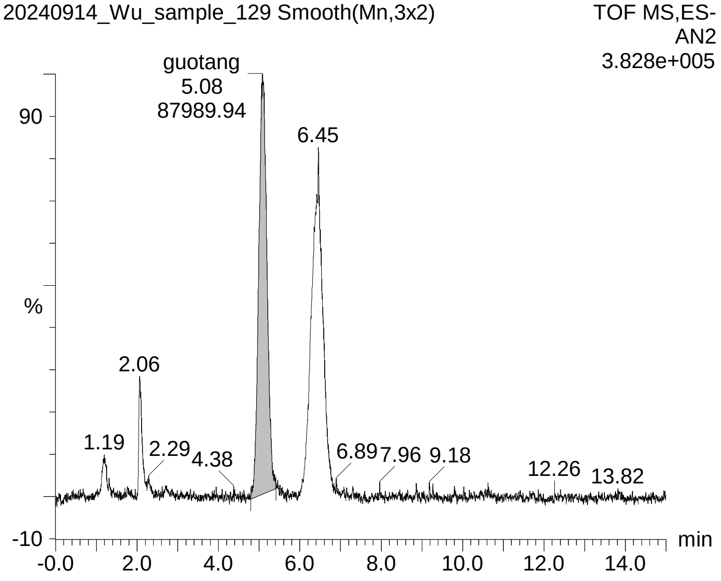

putaotang

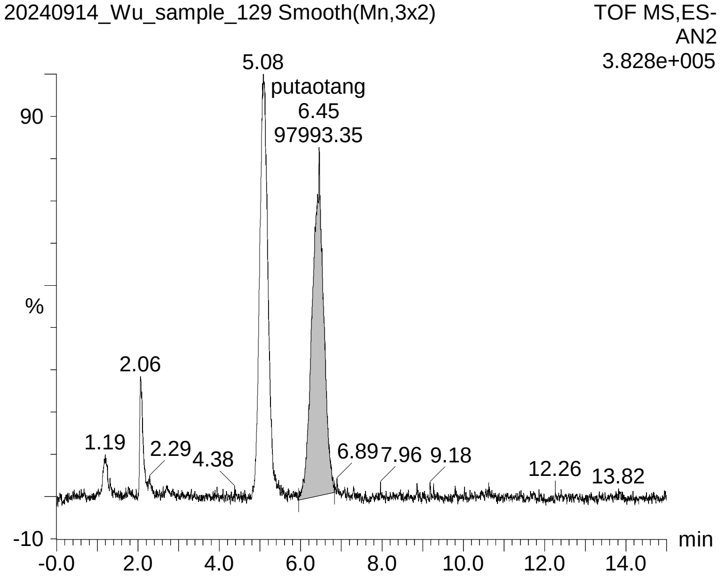

	#	Name	Sample Text	RT	Area	Std. Conc	Conc.
1	1	guotang		5.08	87989.938		64.401274
2	2	putaotang		6.45	97993.352		71.926253

project_wangzhonghua_BeiMu
Dataset: Untitled
Last Altered: Sunday, September 29, 2024 09:54:36 China Standard Time
Printed: Sunday, September 29, 2024 09:59:41 China Standard Time

Name: 20240914_Wu_sample_130, Date: 15-Sep-2024, Time: 04:28:03, ID: , Description:

guotang

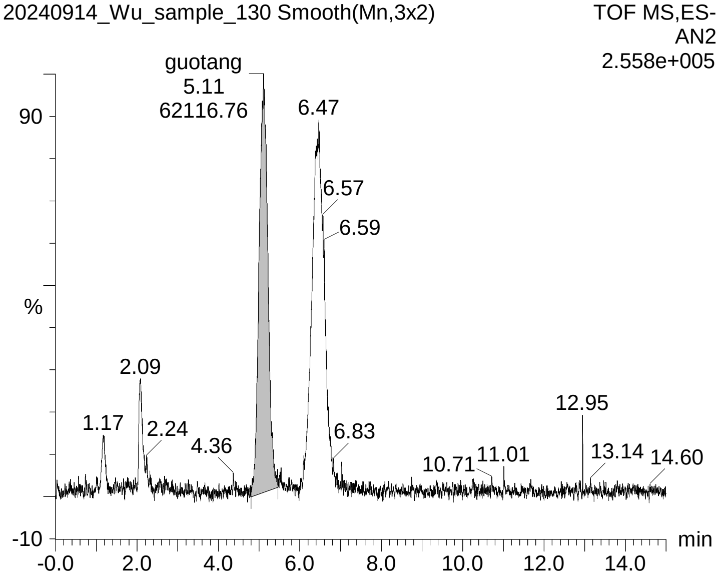

putaotang

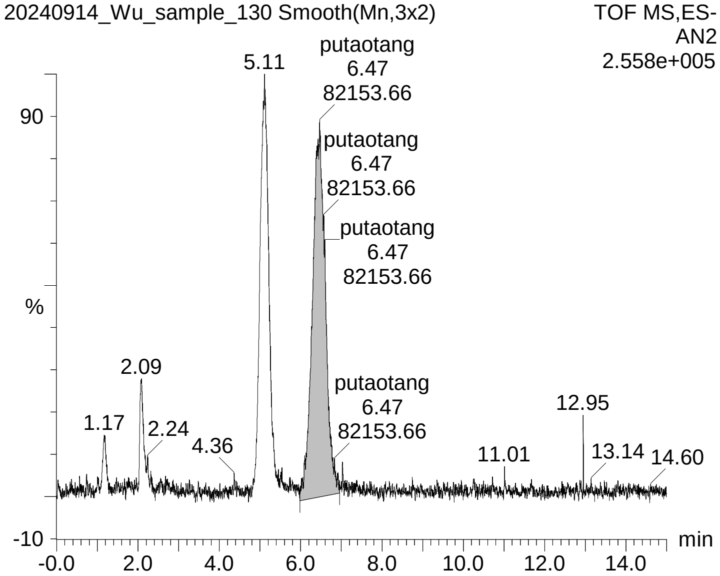

	#	Name	Sample Text	RT	Area	Std. Conc	Conc.
1	1	guotang		5.11	62116.758		49.677654
2	2	putaotang		6.47	82153.664		62.655837

Name: 20240914_Wu_sample_131, Date: 15-Sep-2024, Time: 04:44:04, ID: , Description:

guotang

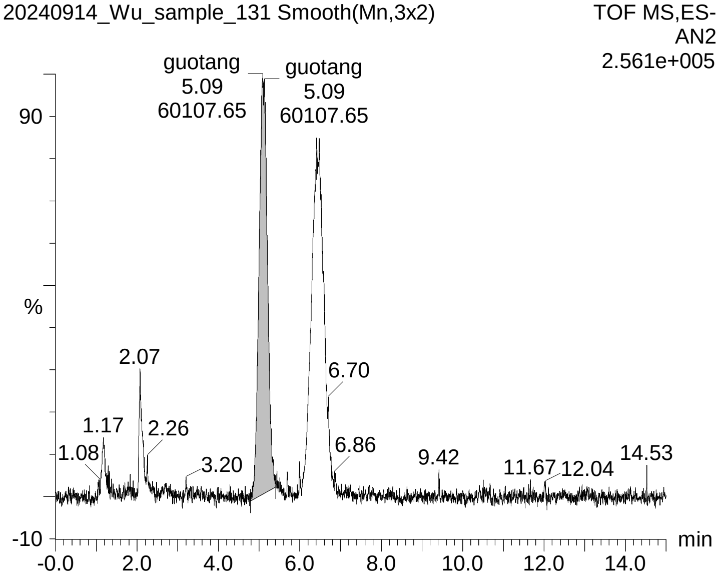

putaotang

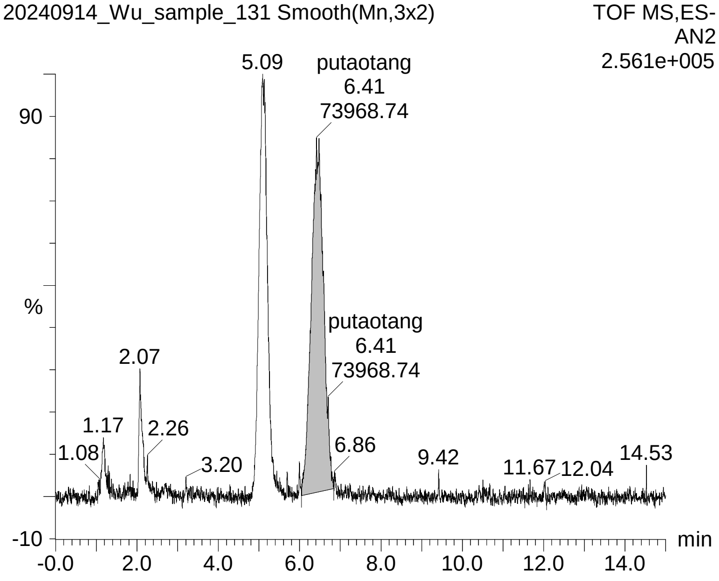

	#	Name	Sample Text	RT	Area	Std. Conc	Conc.
1	1	guotang		5.09	60107.652		48.534334
2	2	putaotang		6.41	73968.742		57.865488

Name: 20240914_Wu_sample_132, Date: 15-Sep-2024, Time: 05:00:01, ID: , Description:

guotang

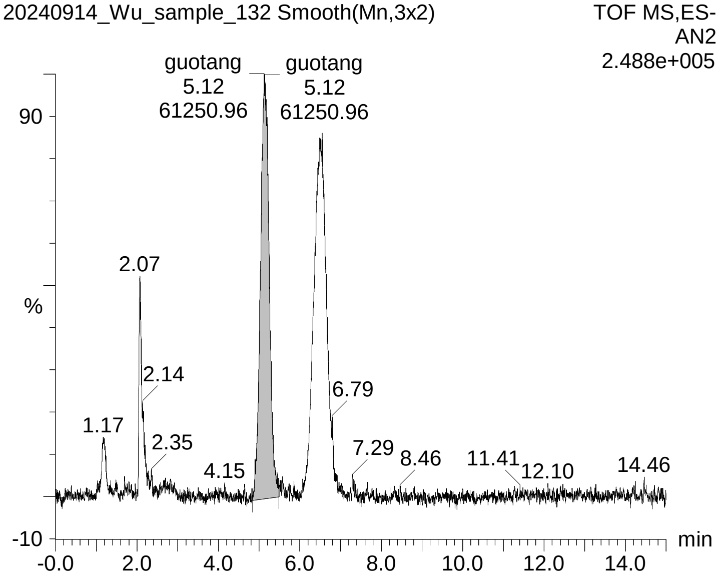

putaotang

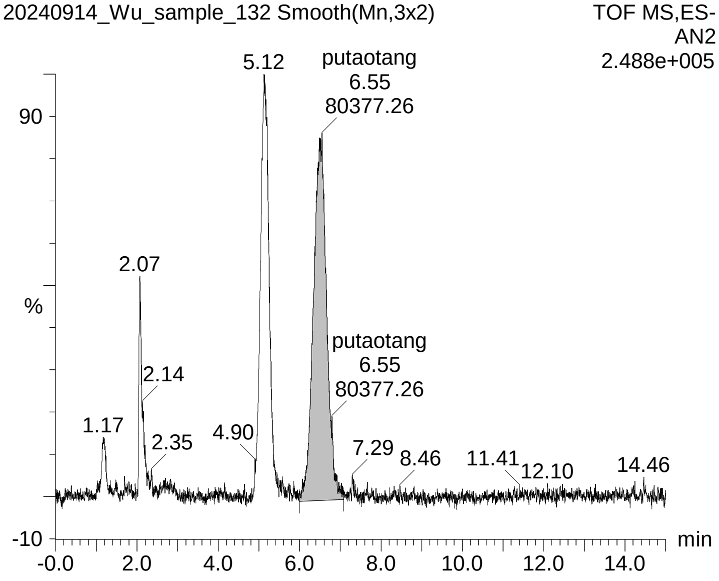

	#	Name	Sample Text	RT	Area	Std. Conc	Conc.
1	1	guotang		5.12	61250.965		49.184958
2	2	putaotang		6.55	80377.258		61.616169

project_wangzhonghua_BeiMu
Dataset: Untitled
Last Altered: Sunday, September 29, 2024 09:54:36 China Standard Time
Printed: Sunday, September 29, 2024 09:59:41 China Standard Time

Name: 20240914_Wu_sample_133, Date: 15-Sep-2024, Time: 05:16:00, ID: , Description:

guotang

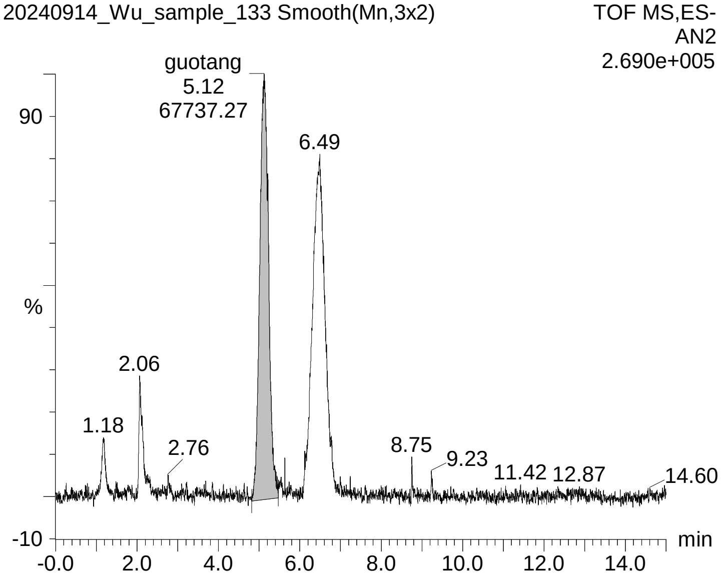

putaotang

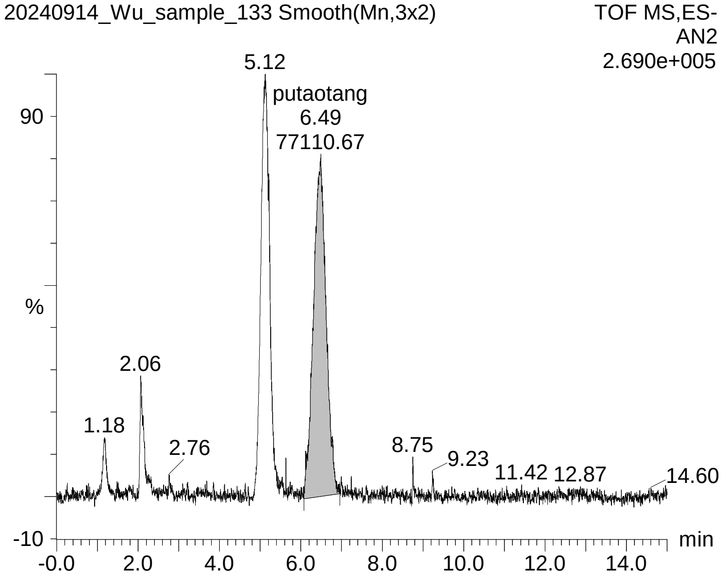

	#	Name	Sample Text	RT	Area	Std. Conc	Conc.
1	1	guotang		5.12	67737.273		52.876114
2	2	putaotang		6.49	77110.672		59.704350

Dataset: Untitled
Last Altered: Sunday, September 29, 2024 09:54:36 China Standard Time
Printed: Sunday, September 29, 2024 09:59:41 China Standard Time

Name: 20240914_Wu_sample_134, Date: 15-Sep-2024, Time: 05:32:01, ID: , Description:

guotang

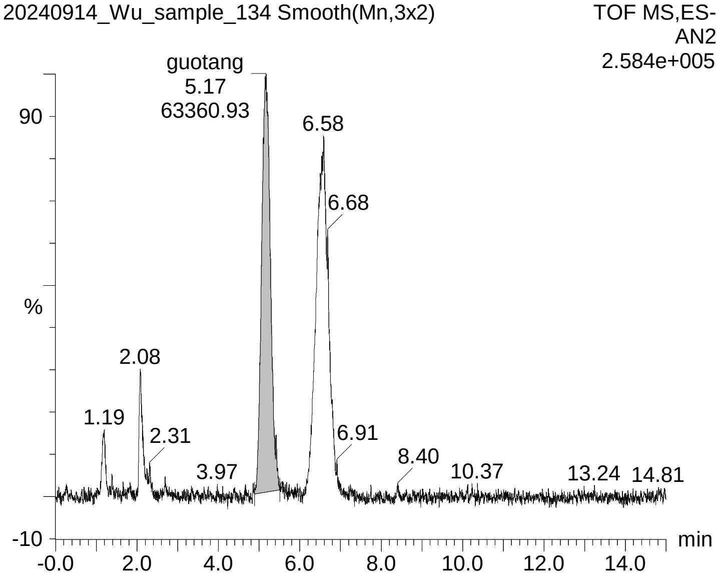

putaotang

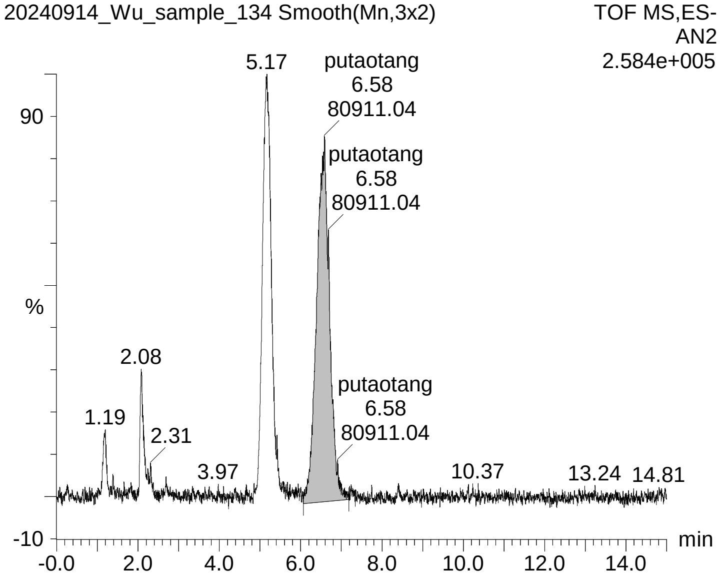

	#	Name	Sample Text	RT	Area	Std. Conc	Conc.
1	1	guotang		5.17	63360.926		50.385671
2	2	putaotang		6.58	80911.039		61.928572

project_wangzhonghua_BeiMu
Dataset: Untitled
Last Altered: Sunday, September 29, 2024 09:54:36 China Standard Time
Printed: Sunday, September 29, 2024 09:59:41 China Standard Time

Name: 20240914_Wu_sample_135, Date: 15-Sep-2024, Time: 05:48:03, ID: , Description:

guotang

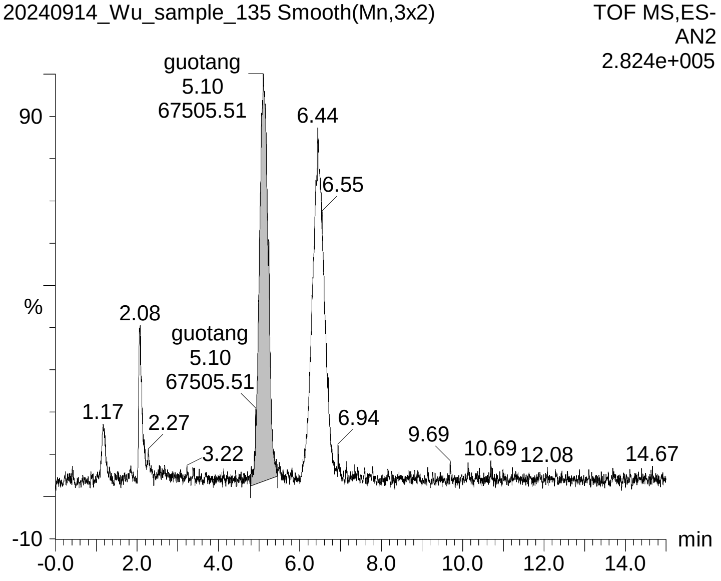

putaotang

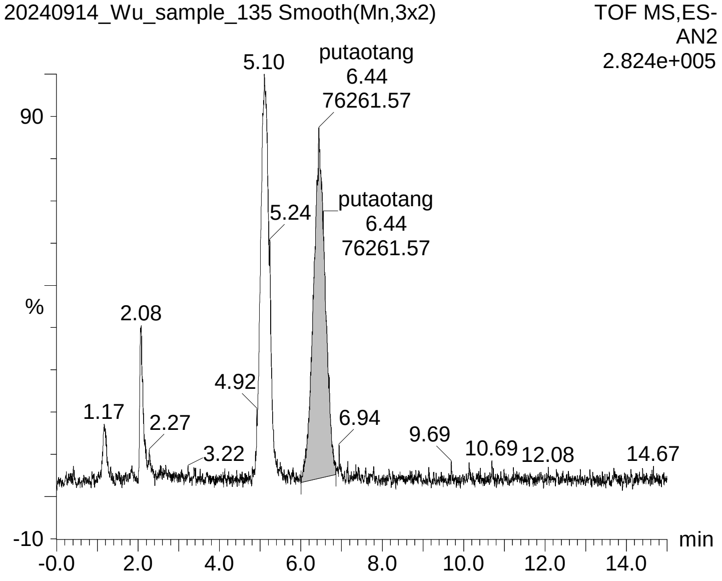

	#	Name	Sample Text	RT	Area	Std. Conc	Conc.
1	1	guotang		5.10	67505.508		52.744223
2	2	putaotang		6.44	76261.570		59.207400

project_wangzhonghua_BeiMu
Dataset: Untitled
Last Altered: Sunday, September 29, 2024 09:54:36 China Standard Time
Printed: Sunday, September 29, 2024 09:59:41 China Standard Time

Name: 20240914_Wu_sample_136, Date: 15-Sep-2024, Time: 06:04:05, ID: , Description:

guotang

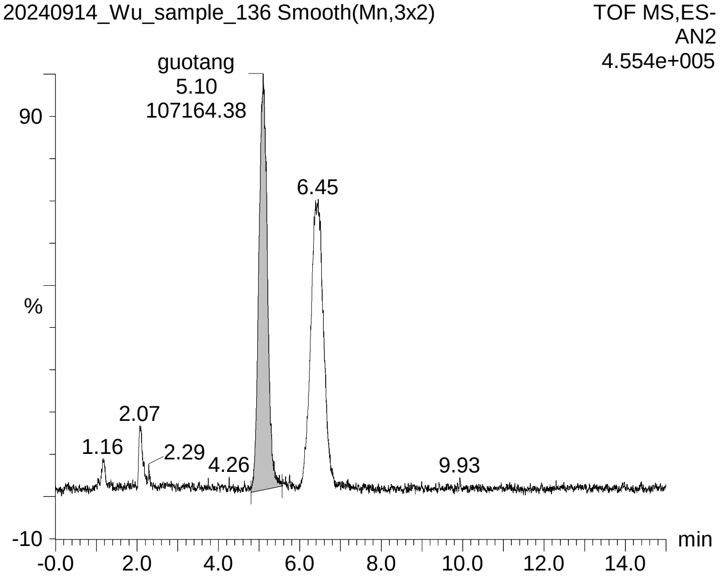

putaotang

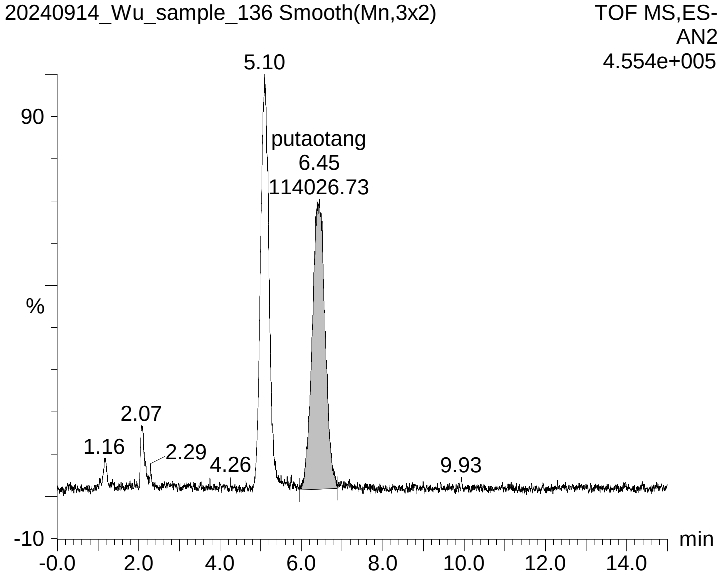

	#	Name	Sample Text	RT	Area	Std. Conc	Conc.
1	1	guotang		5.10	107164.383		75.312854
2	2	putaotang		6.45	114026.727		81.310027

project_wangzhonghua_BeiMu
Dataset: Untitled
Last Altered: Sunday, September 29, 2024 09:54:36 China Standard Time
Printed: Sunday, September 29, 2024 09:59:41 China Standard Time

Name: 20240914_Wu_sample_137, Date: 15-Sep-2024, Time: 06:20:07, ID: , Description:

guotang

20240914_Wu_sample_137 Smooth(Mn,3x2) TOF MS,ES- AN2 4.226e+005

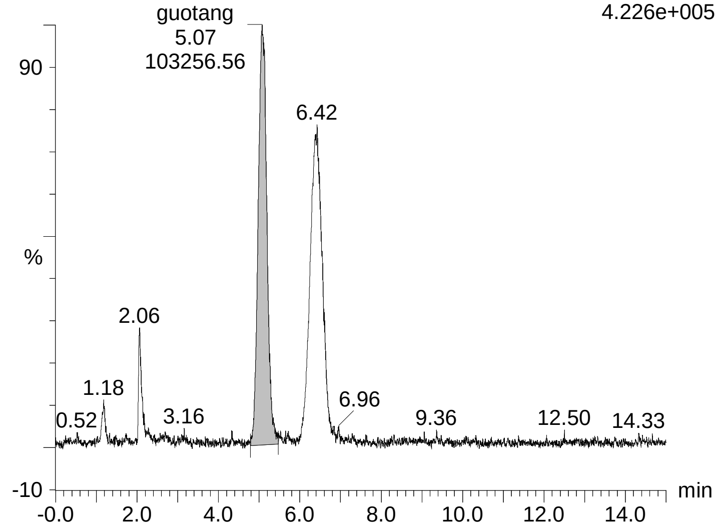

putaotang

20240914_Wu_sample_137 Smooth(Mn,3x2) TOF MS,ES- AN2 4.226e+005

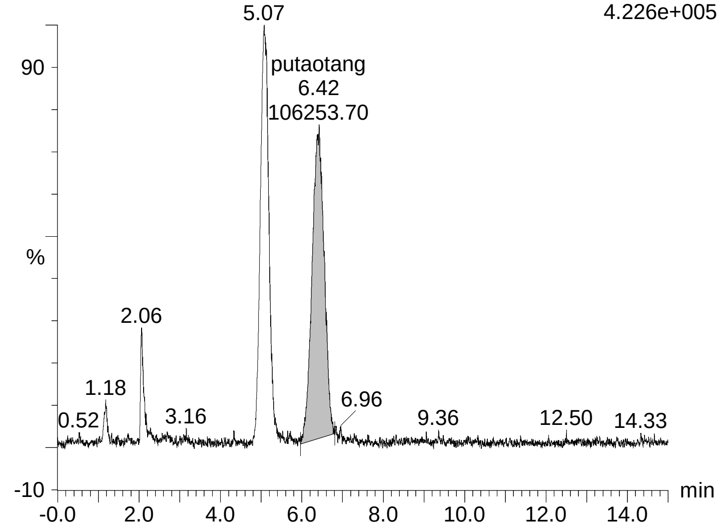

	#	Name	Sample Text	RT	Area	Std. Conc	Conc.
1	1	guotang		5.07	103256.563		73.089035
2	2	putaotang		6.42	106253.703		76.760747

project_wangzhonghua_BeiMu
Dataset: Untitled
Last Altered: Sunday, September 29, 2024 09:54:36 China Standard Time
Printed: Sunday, September 29, 2024 09:59:41 China Standard Time

Name: 20240914_Wu_sample_138, Date: 15-Sep-2024, Time: 06:36:09, ID: , Description:

guotang

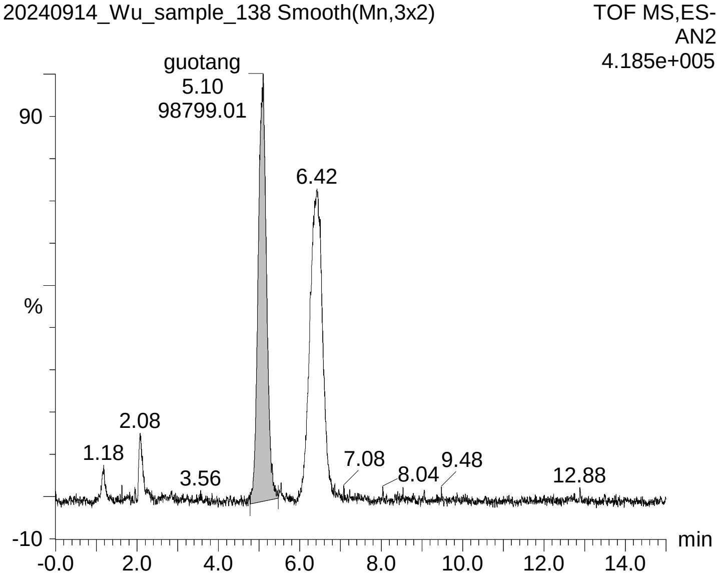

putaotang

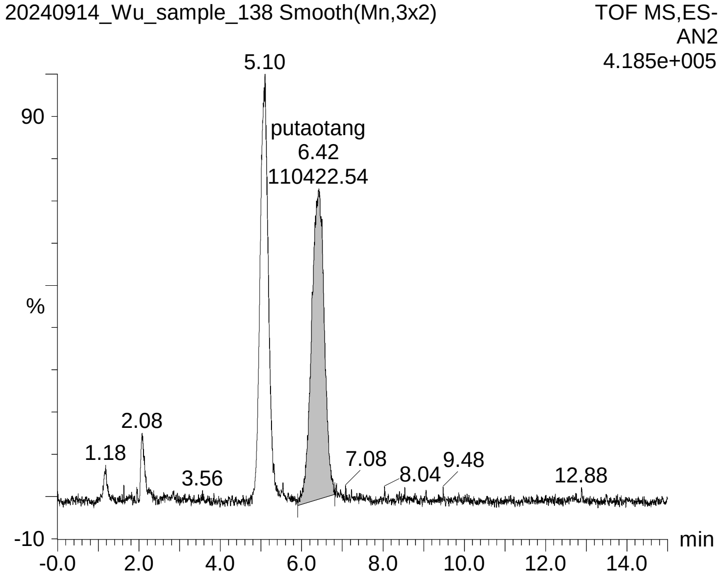

	#	Name	Sample Text	RT	Area	Std. Conc	Conc.
1	1	guotang		5.10	98799.008		70.552379
2	2	putaotang		6.42	110422.539		79.200621

project_wangzhonghua_BeiMu
Dataset: Untitled
Last Altered: Sunday, September 29, 2024 09:54:36 China Standard Time
Printed: Sunday, September 29, 2024 09:59:41 China Standard Time

Name: 20240914_Wu_sample_139, Date: 15-Sep-2024, Time: 06:52:13, ID: , Description:

guotang

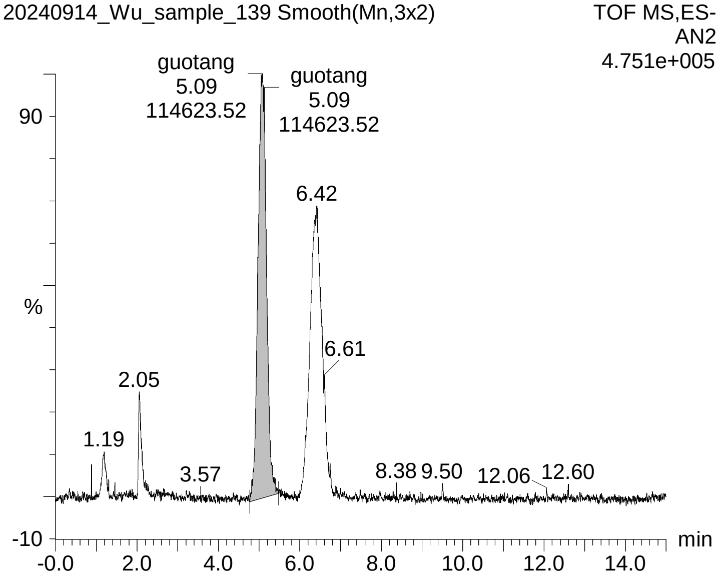

putaotang

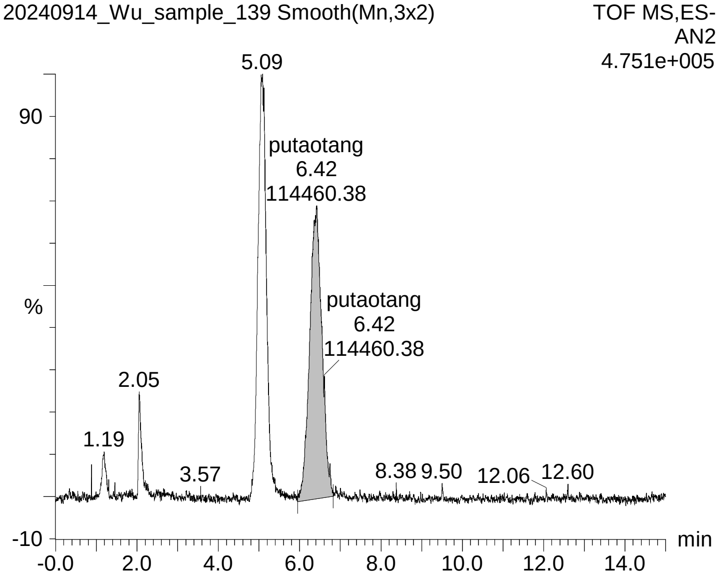

	#	Name	Sample Text	RT	Area	Std. Conc	Conc.
1	1	guotang		5.09	114623.523		79.557618
2	2	putaotang		6.42	114460.375		81.563826

project_wangzhonghua_BeiMu
Dataset: Untitled
Last Altered: Sunday, September 29, 2024 09:54:36 China Standard Time
Printed: Sunday, September 29, 2024 09:59:41 China Standard Time

Name: 20240914_Wu_sample_140, Date: 15-Sep-2024, Time: 07:08:12, ID: , Description:

guotang

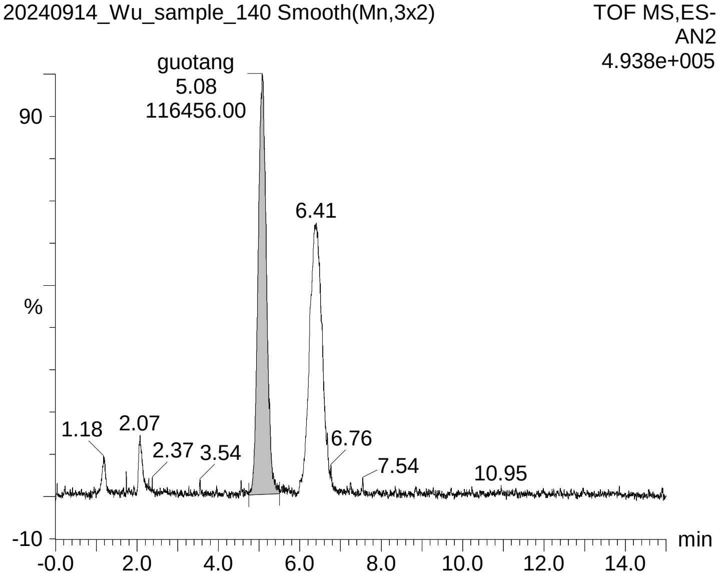

putaotang

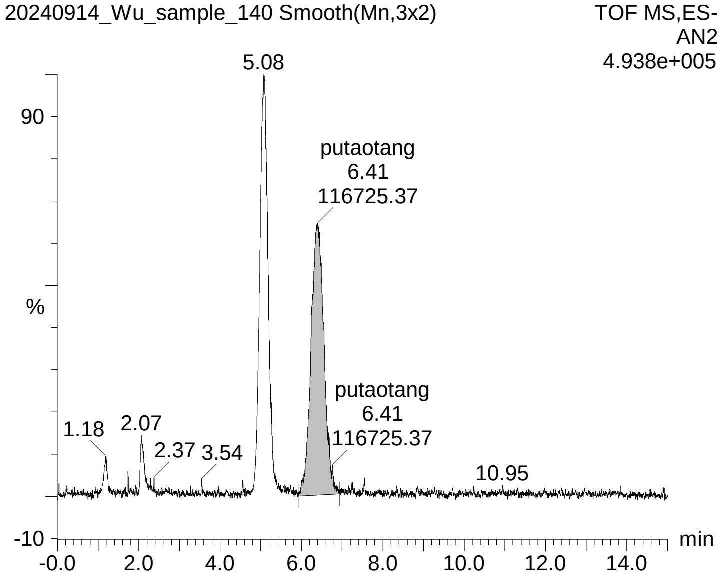

	#	Name	Sample Text	RT	Area	Std. Conc	Conc.
1	1	guotang		5.08	116456.000		80.600423
2	2	putaotang		6.41	116725.367		82.889446

project_wangzhonghua_BeiMu
Dataset: Untitled
Last Altered: Sunday, September 29, 2024 09:54:36 China Standard Time
Printed: Sunday, September 29, 2024 09:59:41 China Standard Time

Name: 20240914_Wu_sample_141, Date: 15-Sep-2024, Time: 07:24:13, ID: , Description:

guotang

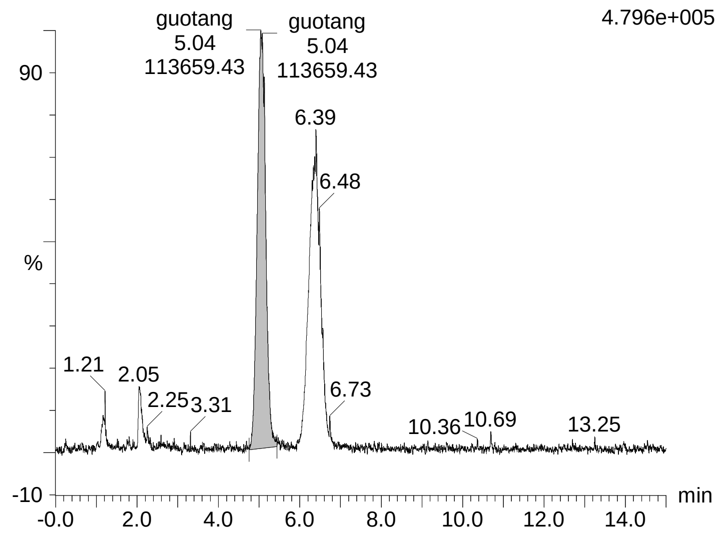

putaotang

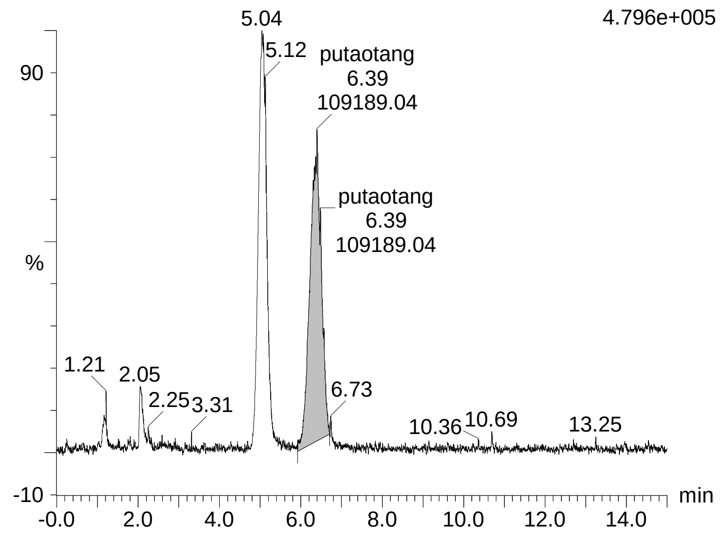

	#	Name	Sample Text	RT	Area	Std. Conc	Conc.
1	1	guotang		5.04	113659.430		79.008982
2	2	putaotang		6.39	109189.039		78.478697

Name: 20240914_Wu_sample_142, Date: 15-Sep-2024, Time: 07:40:15, ID: , Description:

guotang

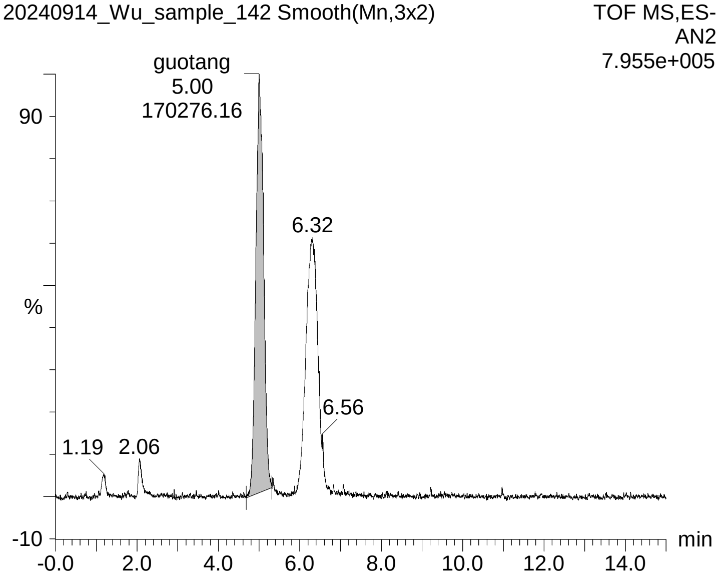

putaotang

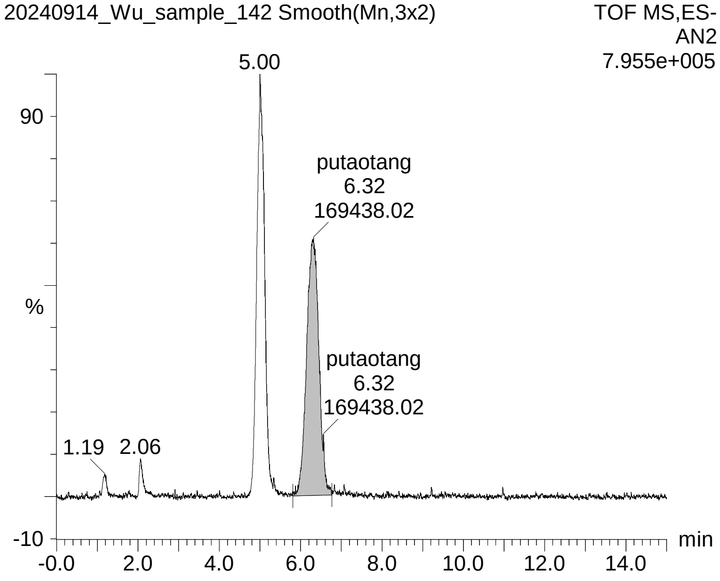

	#	Name	Sample Text	RT	Area	Std. Conc	Conc.
1	1	guotang		5.00	170276.156		111.227797
2	2	putaotang		6.32	169438.016		113.740318

Name: 20240914_Wu_sample_143, Date: 15-Sep-2024, Time: 07:56:17, ID: , Description:

guotang

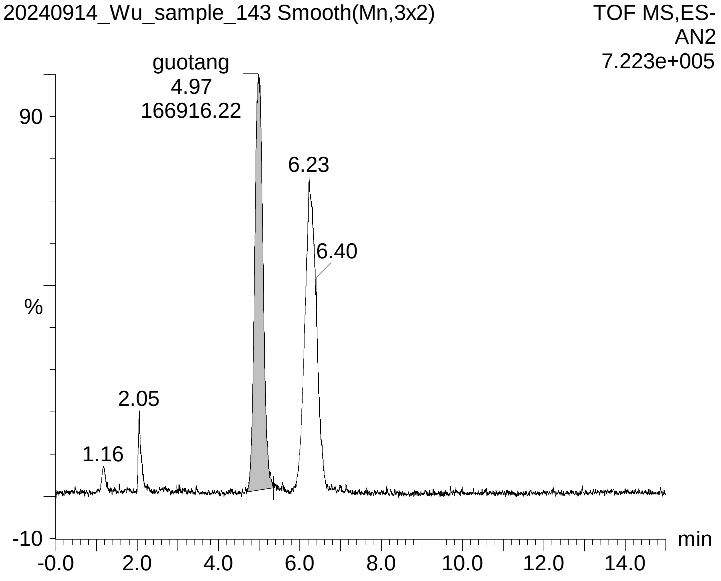

putaotang

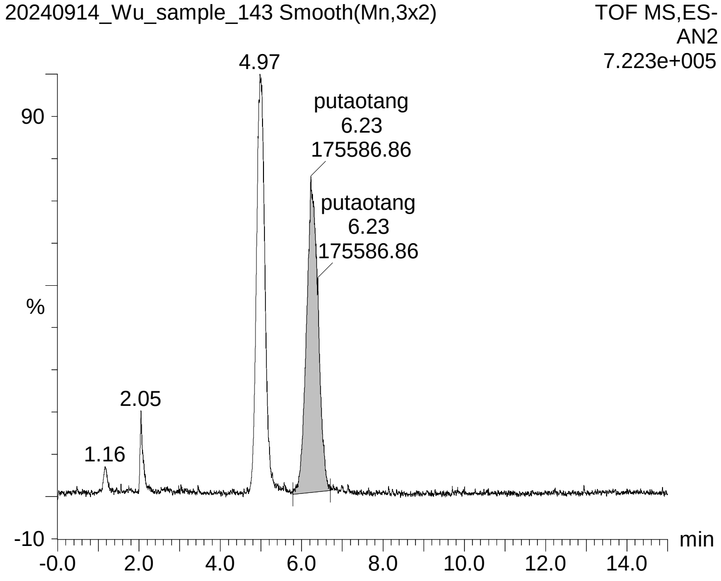

	#	Name	Sample Text	RT	Area	Std. Conc	Conc.
1	1	guotang		4.97	166916.219		109.315762
2	2	putaotang		6.23	175586.859		117.339021

project_wangzhonghua_BeiMu
Dataset: Untitled
Last Altered: Sunday, September 29, 2024 09:54:36 China Standard Time
Printed: Sunday, September 29, 2024 09:59:41 China Standard Time

Name: 20240914_Wu_sample_144, Date: 15-Sep-2024, Time: 08:12:18, ID: , Description:

guotang

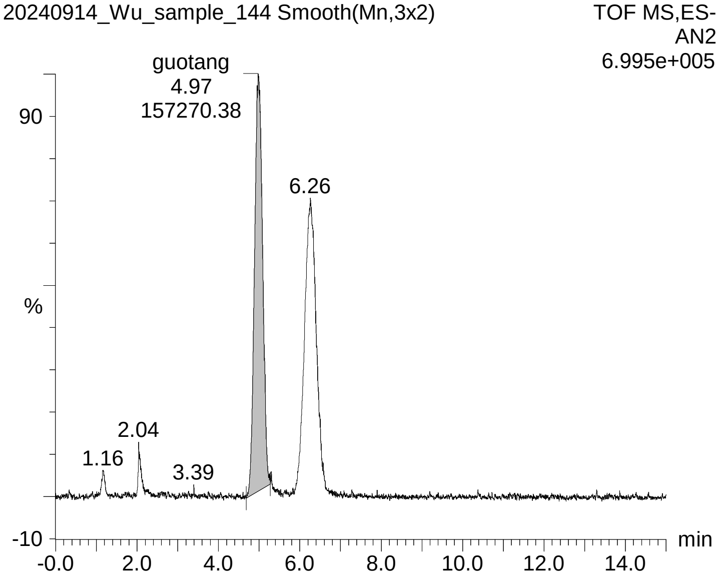

putaotang

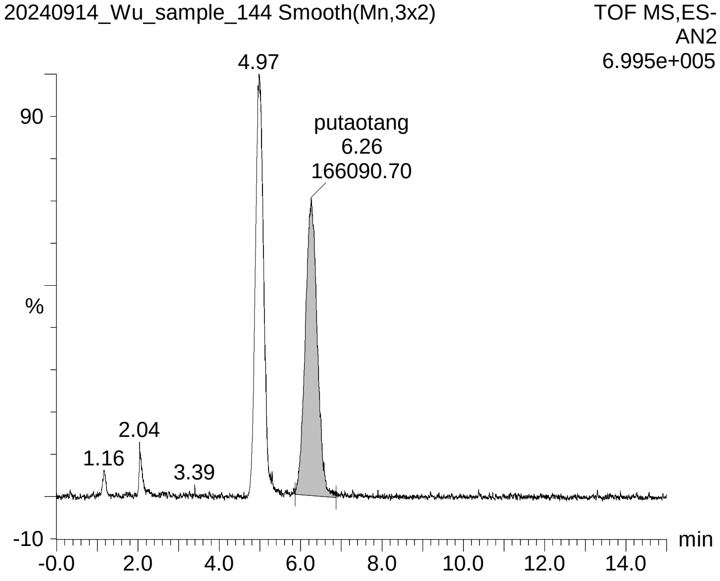

	#	Name	Sample Text	RT	Area	Std. Conc	Conc.
1	1	guotang		4.97	157270.375		103.826613
2	2	putaotang		6.26	166090.703		111.781252

project_wangzhonghua_BeiMu
Dataset: Untitled
Last Altered: Monday, January 13, 2025 09:06:49 China Standard Time
Printed: Monday, January 13, 2025 09:08:10 China Standard Time

Method: F:\data\Wu_yueyan.PRO\MethDB\20241011_ organic acid .mdb 22 Nov 2024 07:57:58
Calibration: 13 Jan 2025 09:06:49

Compound name: jiushisuan
Correlation coefficient: r = 0.935044, r^2 = 0.874307
Calibration curve: 2.31942 * x + 563.917
Response type: External Std, Area
Curve type: Linear, Origin: Include, Weighting: Null, Axis trans: None

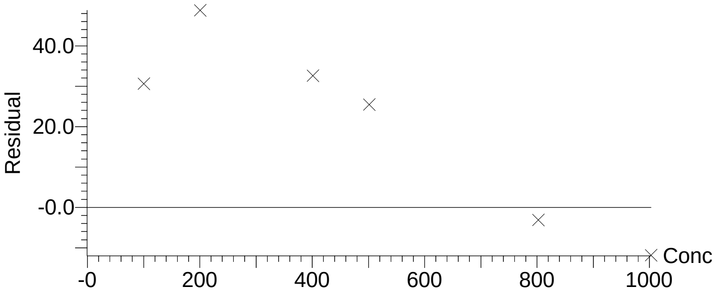

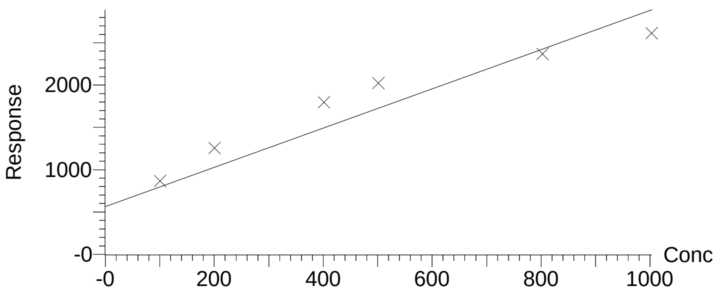

Compound name: ningmensuan
Correlation coefficient: r = 0.997706, r^2 = 0.995417
Calibration curve: 6.56735 * x + 9.93111
Response type: External Std, Area
Curve type: Linear, Origin: Include, Weighting: Null, Axis trans: None

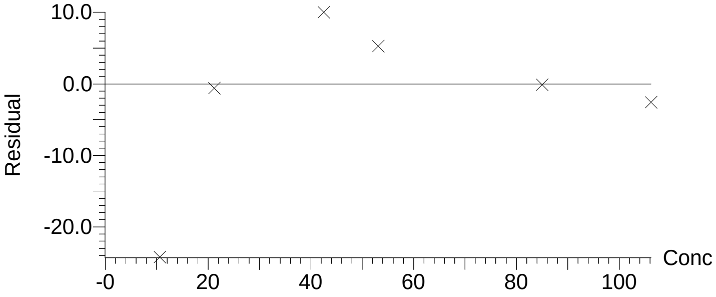

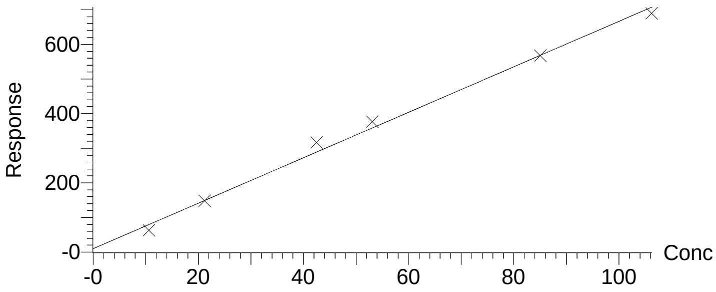

Compound name: pinguosuan
Correlation coefficient: r = 0.886871, r^2 = 0.786540
Calibration curve: 0.741965 * x + 132.773
Response type: External Std, Area
Curve type: Linear, Origin: Include, Weighting: Null, Axis trans: None

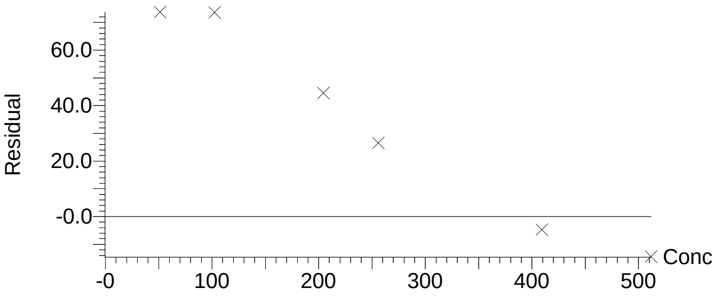

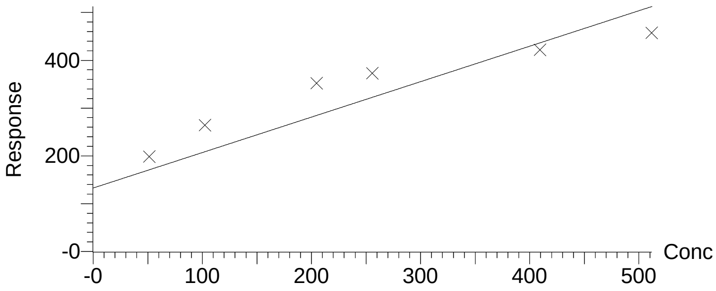

Compound name: huposaun
Correlation coefficient: r = 0.957214, r^2 = 0.916259
Calibration curve: 1.01253 * x + 34.1682
Response type: External Std, Area
Curve type: Linear, Origin: Include, Weighting: Null, Axis trans: None

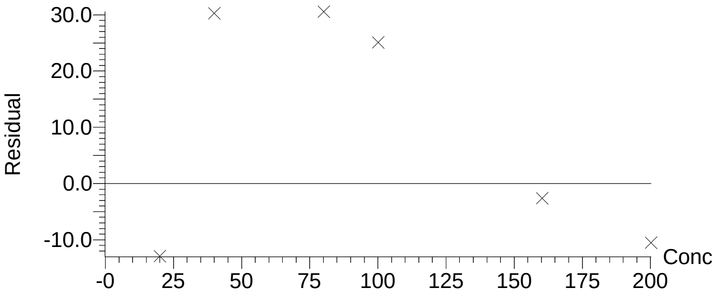

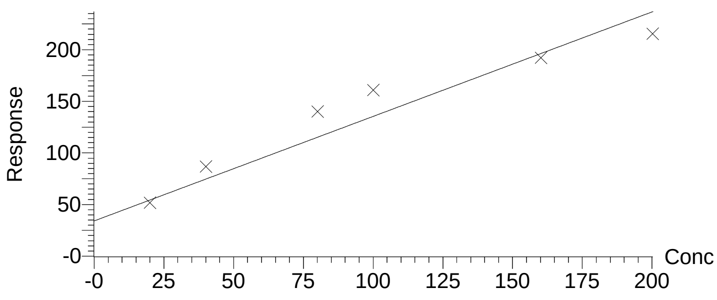

Method: F:\data\Wu_yueyan.PRO\MethDB\20241011_ organic acid .mdb 22 Nov 2024 07:57:58
Calibration: 13 Jan 2025 09:06:49

Name: 202401011_youjisuan_STD001a, Date: 11-Oct-2024, Time: 11:22:39, ID: , Description:

Name: 202401011_youjisuan_STD001a, Date: 11-Oct-2024, Time: 11:22:39, ID: , Description:

jiushisuan

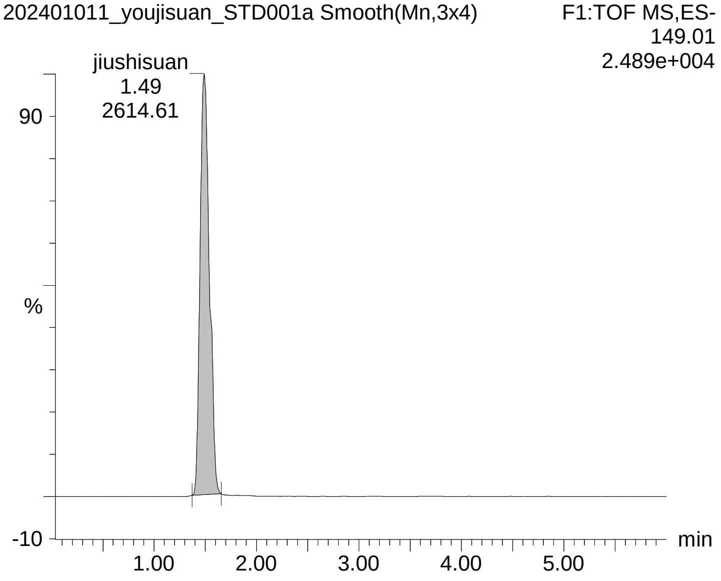

ningmensuan

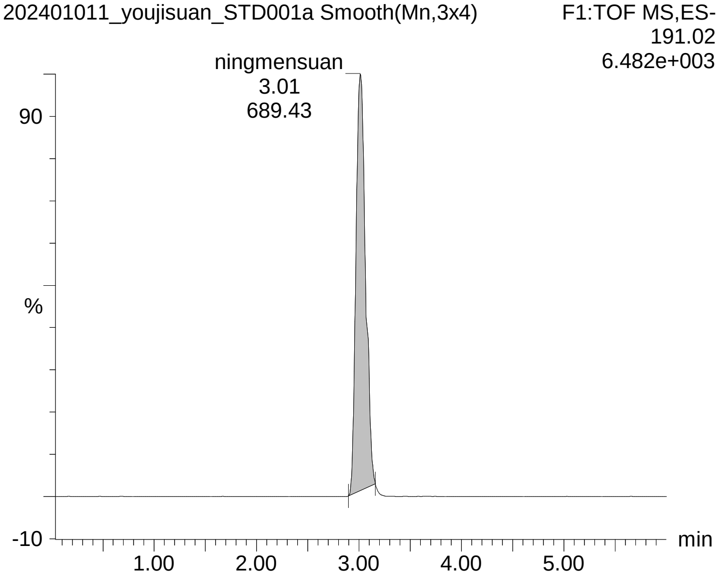

pinguosuan

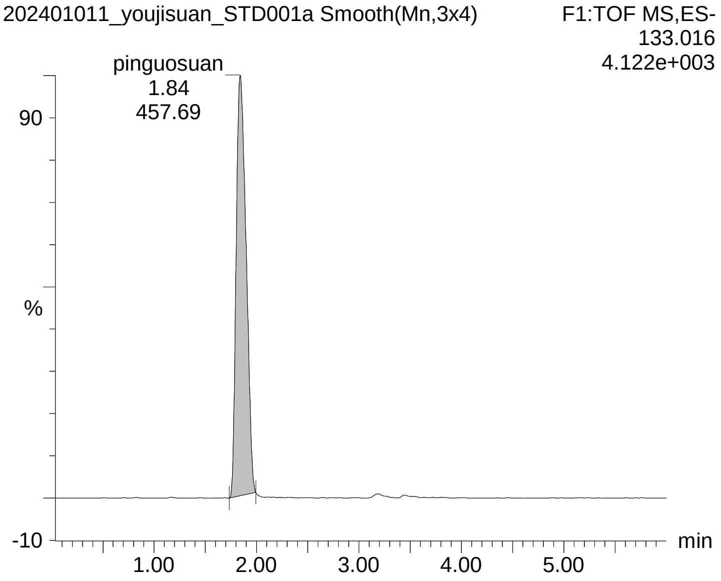

huposaun

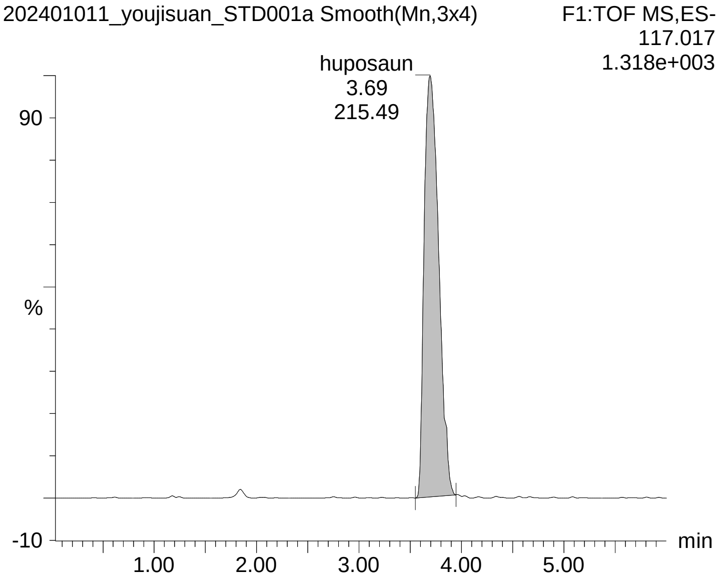

	#	Name	Sample Text	RT	Area	Std. Conc	Conc.
1	-1						
2	1	jiushisuan		1.49	2614.611	1002.800	884.141729
3	2	ningmensuan		3.01	689.427	106.200	103.465690
4	3	pinguosuan		1.84	457.691	511.600	437.916009
5	4	huposaun		3.69	215.493	200.200	179.081166

Name: 202401011_youjisuan_STD002a, Date: 11-Oct-2024, Time: 11:29:36, ID: , Description:

project_wangzhonghua_BeiMu
Dataset: Untitled
Last Altered: Monday, January 13, 2025 09:06:49 China Standard Time
Printed: Monday, January 13, 2025 09:08:10 China Standard Time

Name: 202401011_youjisuan_STD002a, Date: 11-Oct-2024, Time: 11:29:36, ID: , Description:

jiushisuan

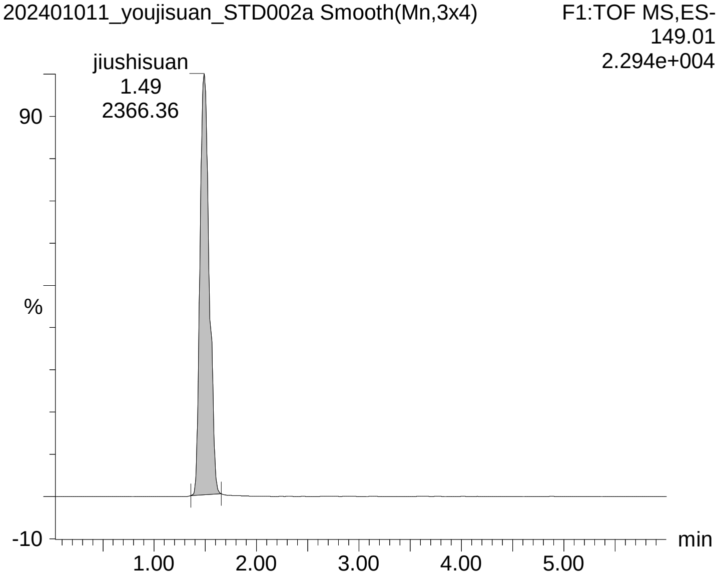

ningmensuan

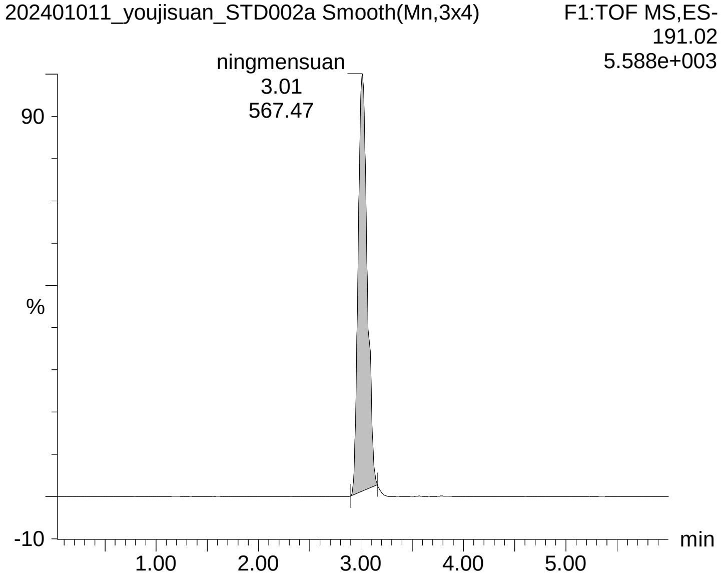

pinguosuan

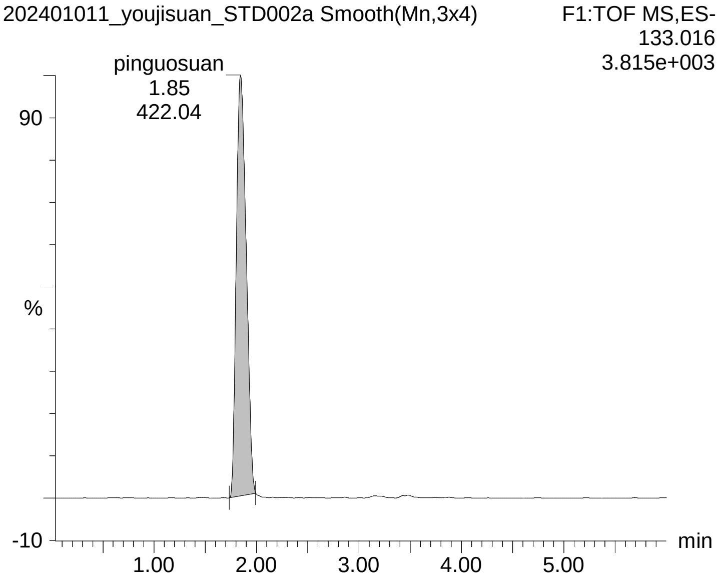

huposaun

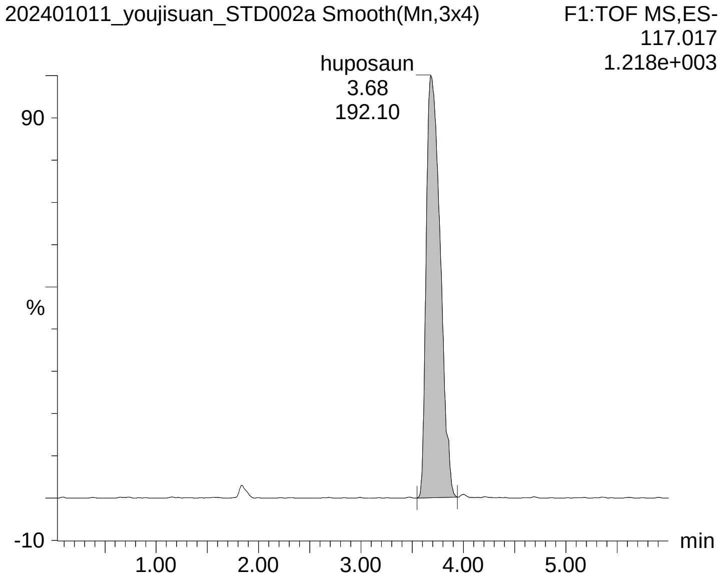

	#	Name	Sample Text	RT	Area	Std. Conc	Conc.
1	-1						
2	1	jiushisuan		1.49	2366.364	802.200	777.111840
3	2	ningmensuan		3.01	567.474	85.000	84.896113
4	3	pinguosuan		1.85	422.038	409.300	389.863853
5	4	huposaun		3.68	192.104	160.200	155.981573

Name: 202401011_youjisuan_STD003a, Date: 11-Oct-2024, Time: 11:36:38, ID: , Description:

Name: 202401011_youjisuan_STD003a, Date: 11-Oct-2024, Time: 11:36:38, ID: , Description:

jiushisuan

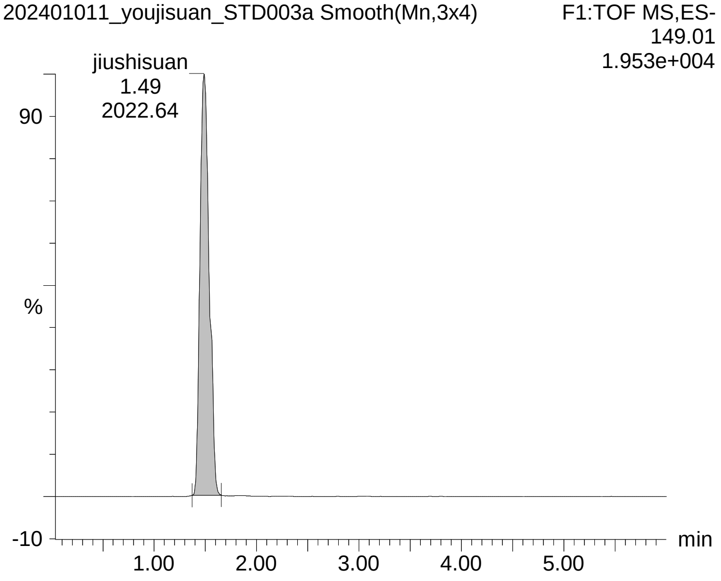

ningmensuan

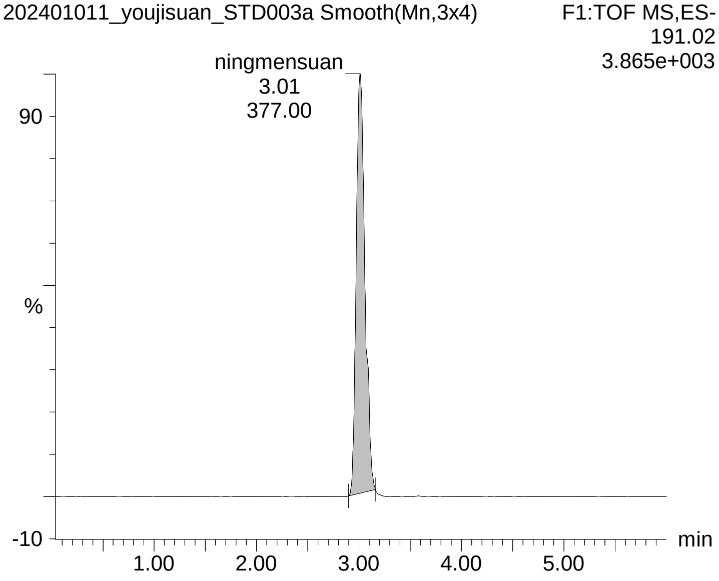

pinguosuan

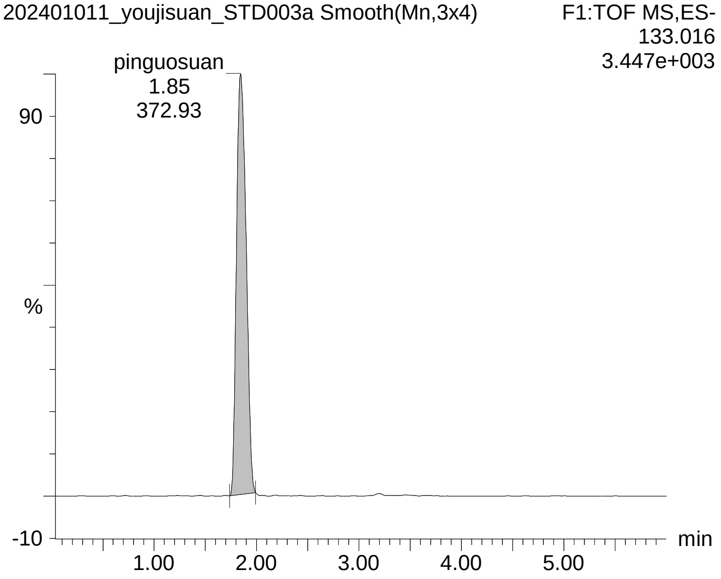

huposaun

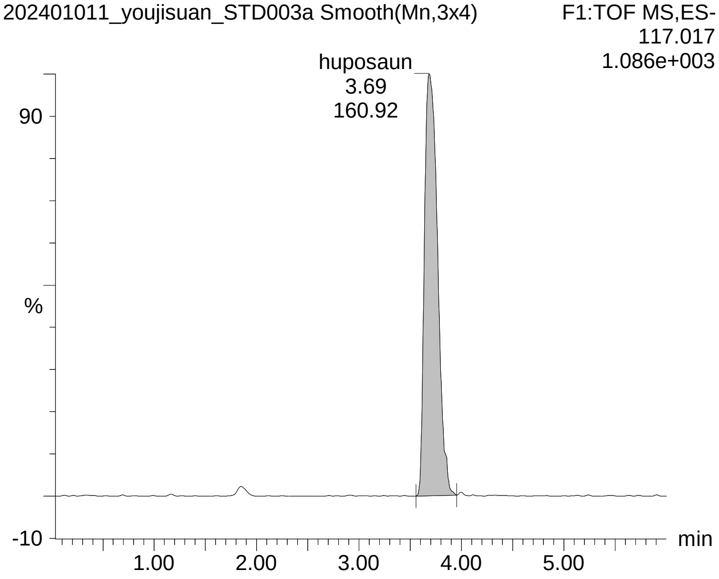

	#	Name	Sample Text	RT	Area	Std. Conc	Conc.
1	-1						
2	1	jiushisuan		1.49	2022.638	501.400	628.916873
3	2	ningmensuan		3.01	376.995	53.100	55.892198
4	3	pinguosuan		1.85	372.925	255.800	323.670670
5	4	huposaun		3.69	160.922	100.100	125.185409

Name: 202401011_youjisuan_STD004a, Date: 11-Oct-2024, Time: 11:43:38, ID: , Description:

Name: 202401011_youjisuan_STD004a, Date: 11-Oct-2024, Time: 11:43:38, ID: , Description:

jiushisuan

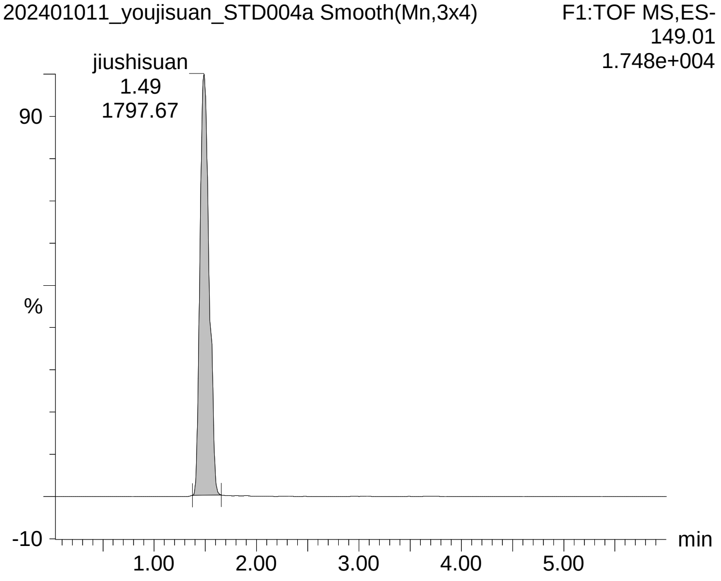

ningmensuan

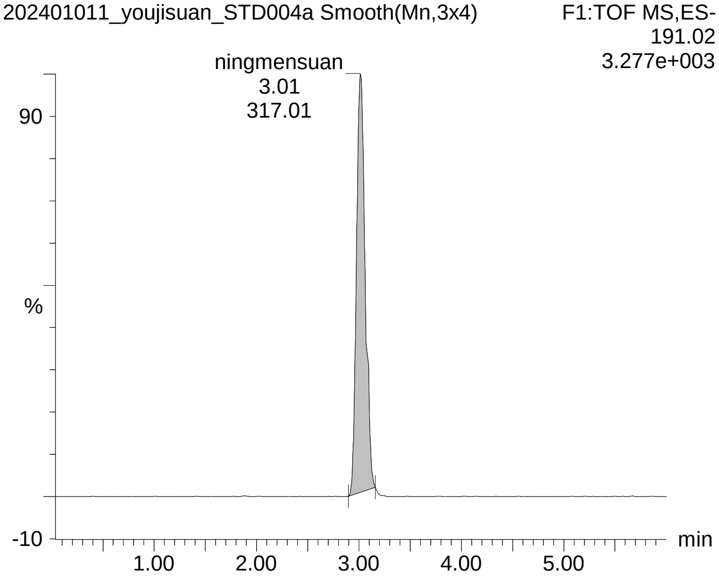

pinguosuan

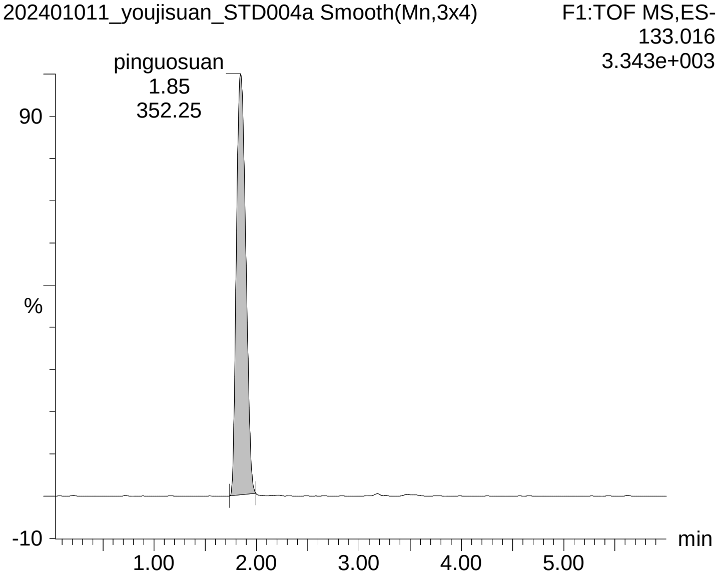

huposaun

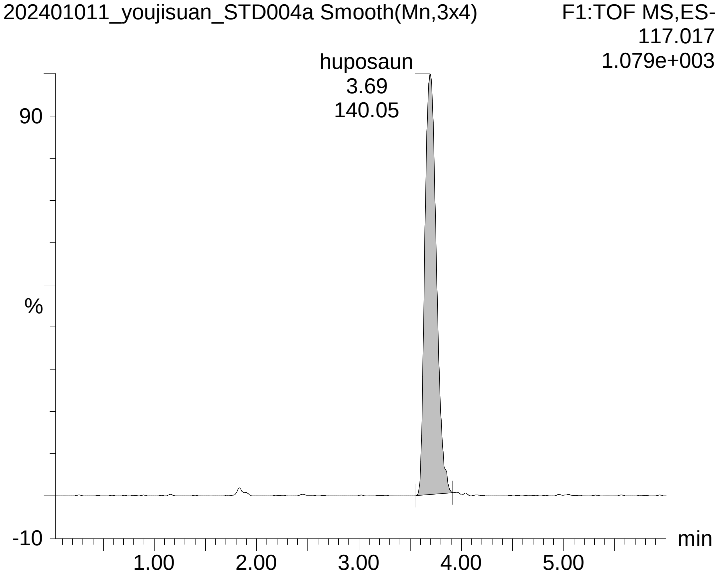

	#	Name	Sample Text	RT	Area	Std. Conc	Conc.
1	-1						
2	1	jiushisuan		1.49	1797.670	401.100	531.923554
3	2	ningmensuan		3.01	317.008	42.500	46.758079
4	3	pinguosuan		1.85	352.248	204.600	295.802764
5	4	huposaun		3.69	140.047	80.100	104.568710

Name: 202401011_youjisuan_STD005a, Date: 11-Oct-2024, Time: 11:51:43, ID: , Description:

project_wangzhonghua_BeiMu
Dataset: Untitled
Last Altered: Monday, January 13, 2025 09:06:49 China Standard Time
Printed: Monday, January 13, 2025 09:08:10 China Standard Time

Name: 202401011_youjisuan_STD005a, Date: 11-Oct-2024, Time: 11:51:43, ID: , Description:

jiushisuan

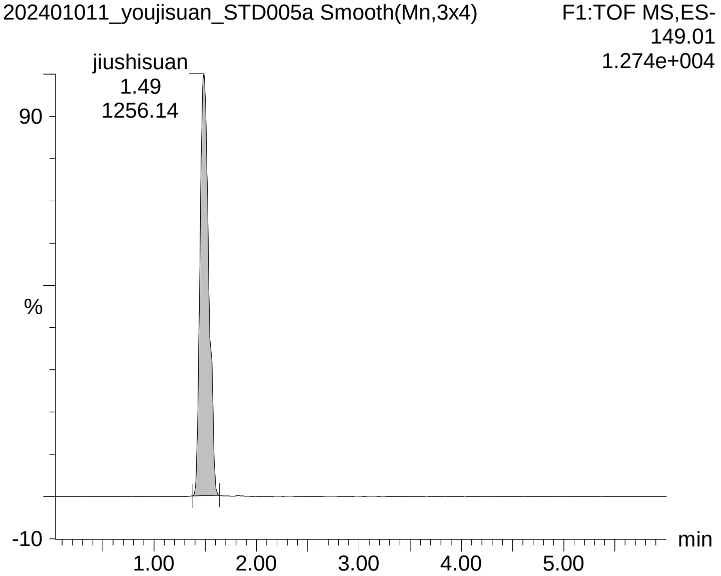

ningmensuan

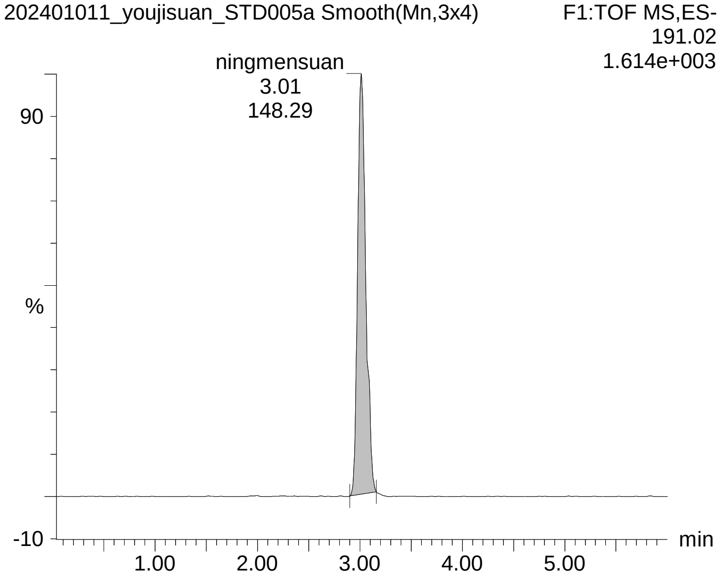

pinguosuan

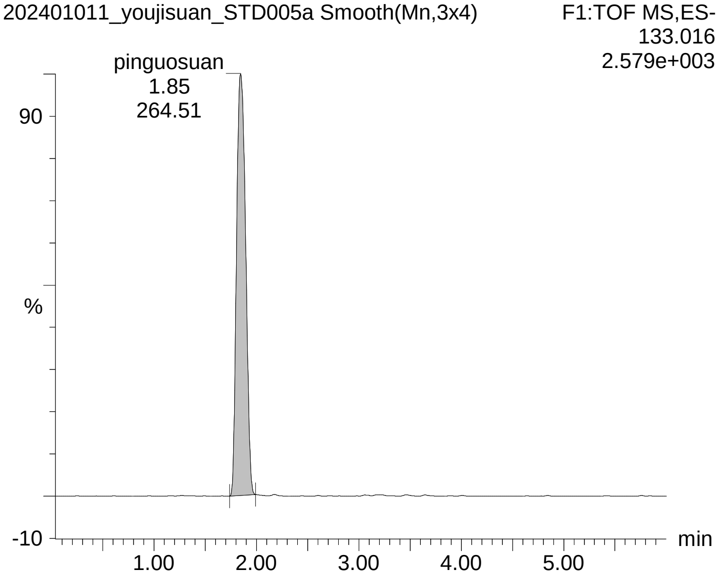

huposaun

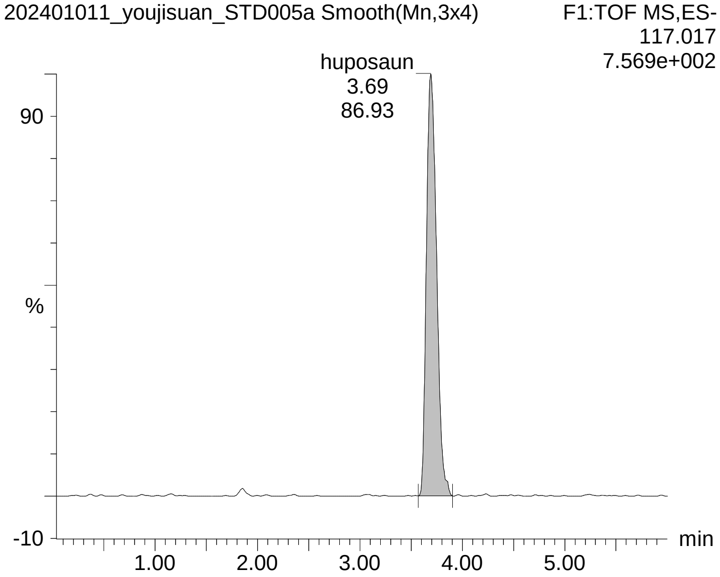

	#	Name	Sample Text	RT	Area	Std. Conc	Conc.
1	-1						
2	1	jiushisuan		1.49	1256.137	200.600	298.445537
3	2	ningmensuan		3.01	148.291	21.200	21.067826
4	3	pinguosuan		1.85	264.508	102.300	177.549147
5	4	huposaun		3.69	86.932	40.000	52.110937

Name: 202401011_youjisuan_STD006a, Date: 11-Oct-2024, Time: 11:58:43, ID: , Description:

Name: 202401011_youjisuan_STD006a, Date: 11-Oct-2024, Time: 11:58:43, ID: , Description:

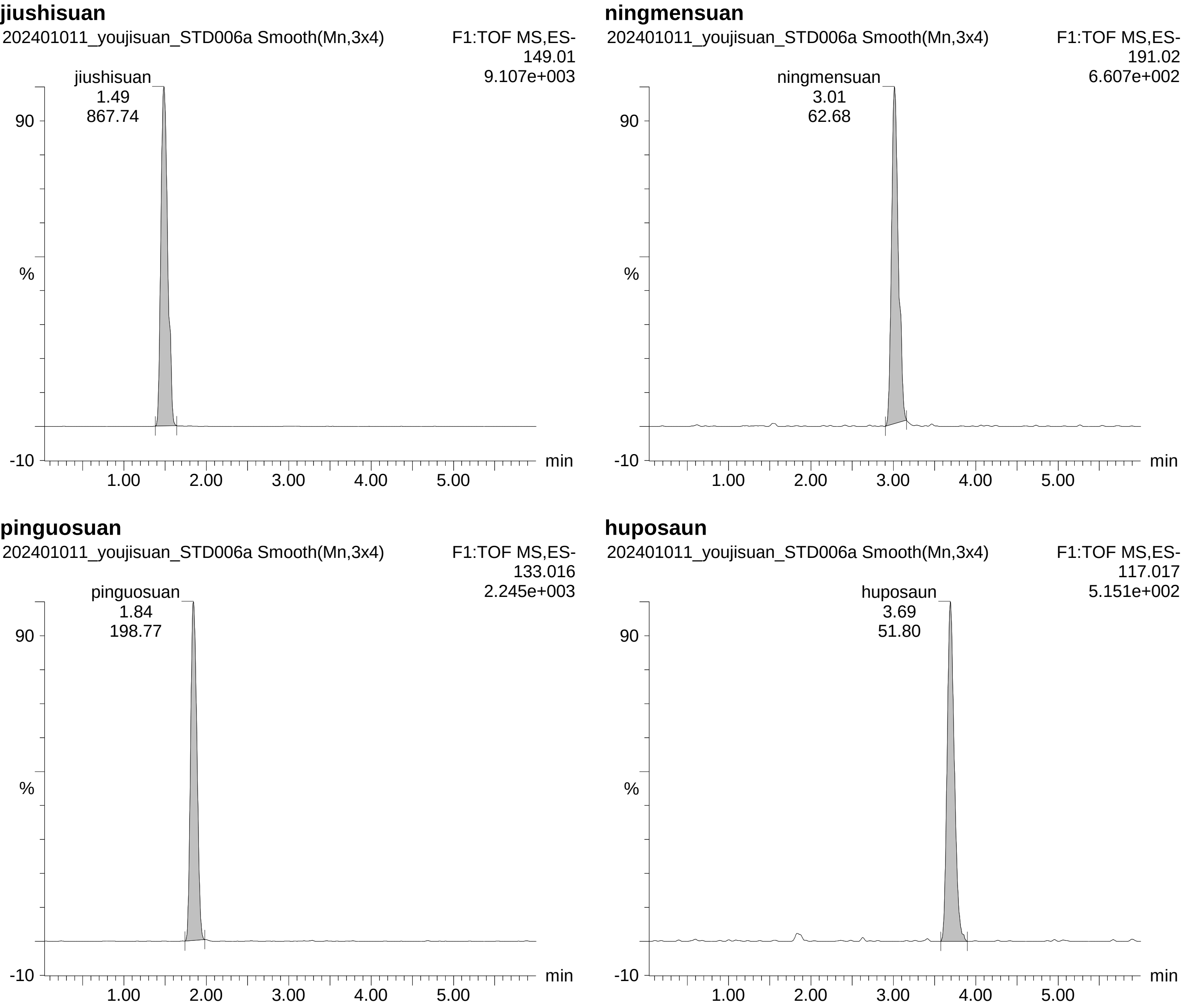

	#	Name	Sample Text	RT	Area	Std. Conc	Conc.
1	-1						
2	1	jiushisuan		1.49	867.736	100.300	130.989269
3	2	ningmensuan		3.01	62.682	10.600	8.032289
4	3	pinguosuan		1.84	198.767	51.200	88.945192
5	4	huposaun		3.69	51.804	20.000	17.417600

project_wangzhonghua_BeiMu
Dataset: F:\data\Wu_yueyan.PRO\20240918-STD.qld
Last Altered: Wednesday, September 18, 2024 14:19:16 China Standard Time
Printed: Monday, January 13, 2025 08:56:42 China Standard Time

Method: F:\data\Wu_yueyan.PRO\MethDB\20240918_ tang lei_ELSD .mdb 18 Sep 2024 14:15:05
Calibration: F:\data\zhanghuien.PRO\CurveDB\20240918_tanglei.cdb 18 Sep 2024 14:19:16

Compound name: guotang
Correlation coefficient: r = 0.990650, r^2 = 0.981387
Calibration curve: 1757.26 * x + -25179.6
Response type: External Std, Area
Curve type: Linear, Origin: Exclude, Weighting: Null, Axis trans: None

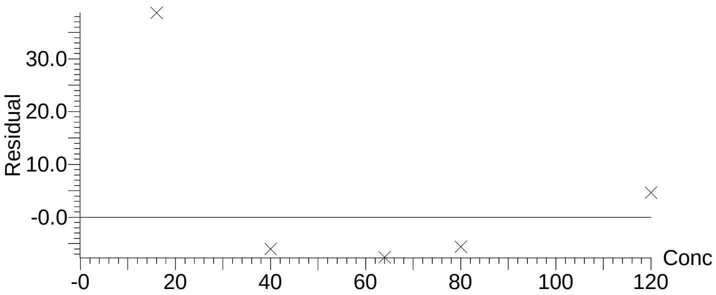

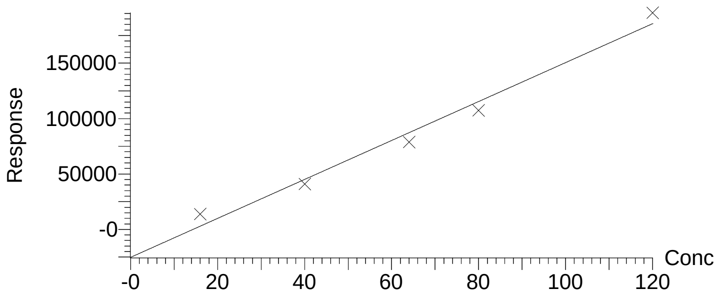

Compound name: putaotang
Correlation coefficient: r = 0.995122, r^2 = 0.990268
Calibration curve: 1486.74 * x + -23705.7
Response type: External Std, Area
Curve type: Linear, Origin: Exclude, Weighting: Null, Axis trans: None

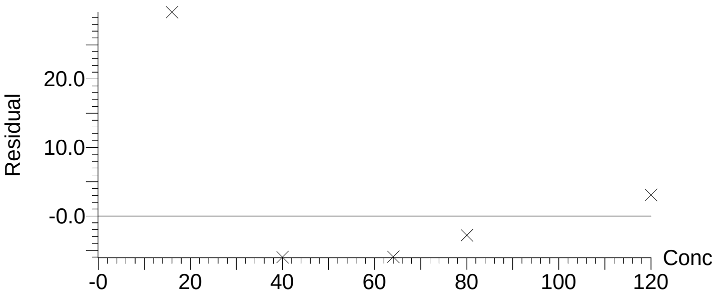

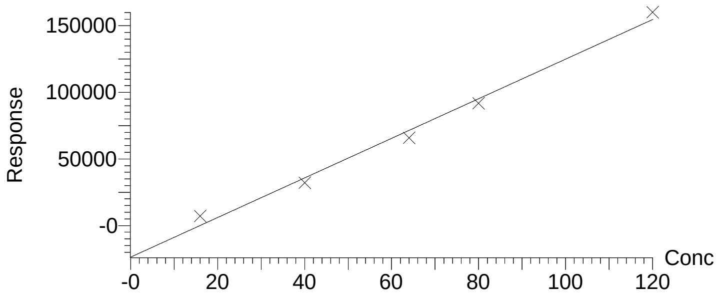

Compound name: zhetang
Correlation coefficient: r = 0.994858, r^2 = 0.989743
Calibration curve: 1866.58 * x + -37450
Response type: External Std, Area
Curve type: Linear, Origin: Exclude, Weighting: Null, Axis trans: None

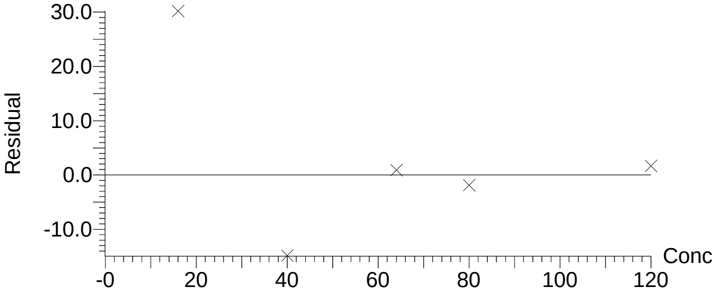

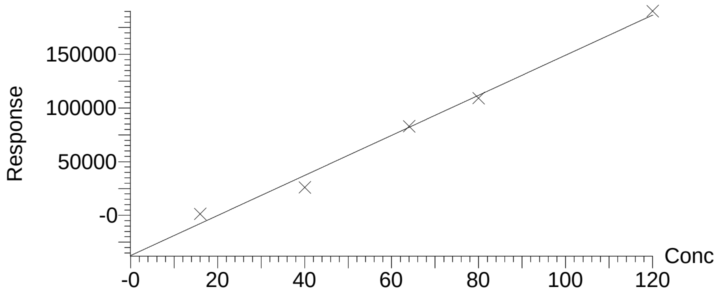

Compound name: mutang
Correlation coefficient: r = 0.991167, r^2 = 0.982411
Calibration curve: 1844.46 * x + -27630.8
Response type: External Std, Area
Curve type: Linear, Origin: Exclude, Weighting: Null, Axis trans: None

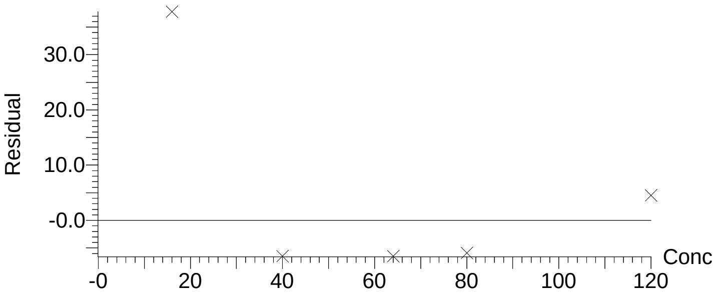

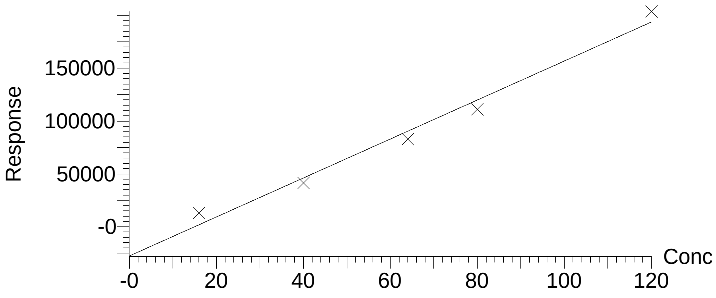

project_wangzhonghua_BeiMu
Dataset: F:\data\Wu_yueyan.PRO\20240918-STD.qld
Last Altered: Wednesday, September 18, 2024 14:19:16 China Standard Time
Printed: Monday, January 13, 2025 08:56:42 China Standard Time

Method: F:\data\Wu_yueyan.PRO\MethDB\20240918_ tang lei_ELSD .mdb 18 Sep 2024 14:15:05
Calibration: F:\data\zhanghuien.PRO\CurveDB\20240918_tanglei.cdb 18 Sep 2024 14:19:16

Name: 20240914_Wu_STD001, Date: 15-Sep-2024, Time: 08:28:23, ID: , Description:

guotang

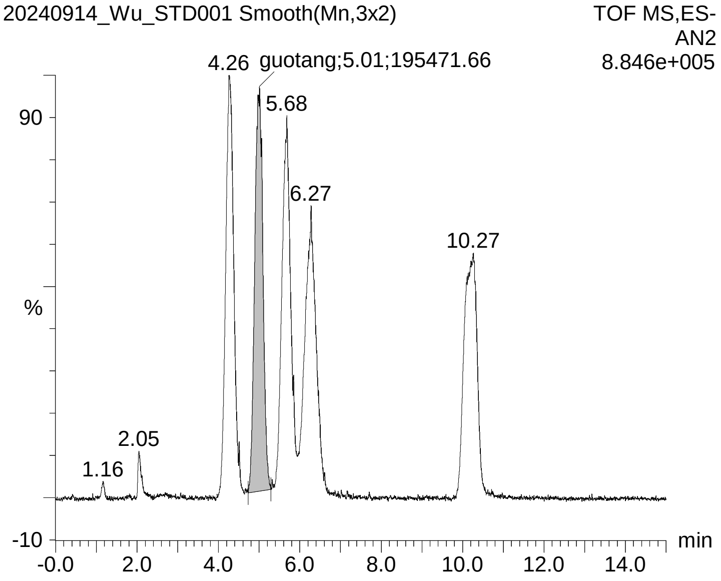

putaotang

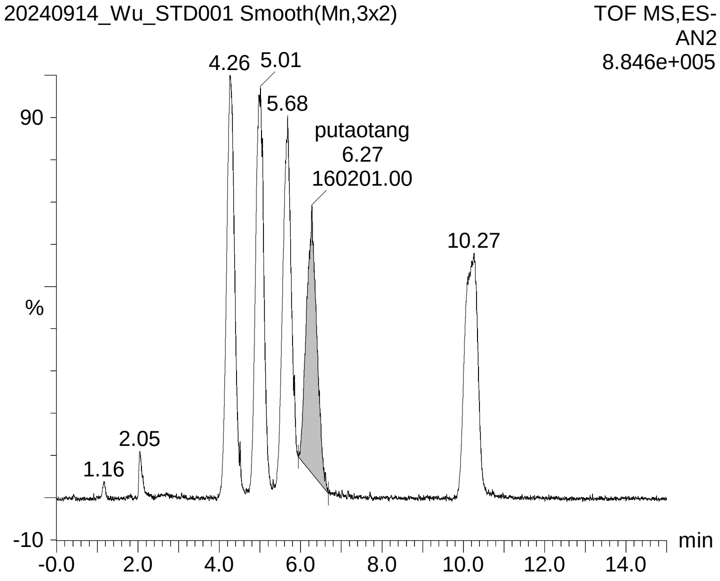

zhetang

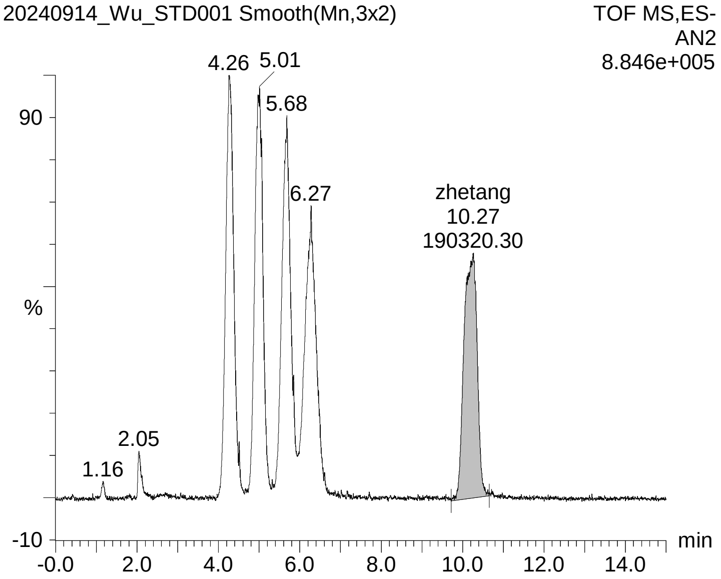

	#	Name	Sample Text	RT	Area	Std. Conc	Conc.
1	1	guotang		5.01	195471.656	120.000	
2	2	putaotang		6.27	160201.000	120.000	
3	3	zhetang		10.27	190320.297	120.000	

Name: 20240914_Wu_STD002, Date: 15-Sep-2024, Time: 08:44:21, ID: , Description:

guotang

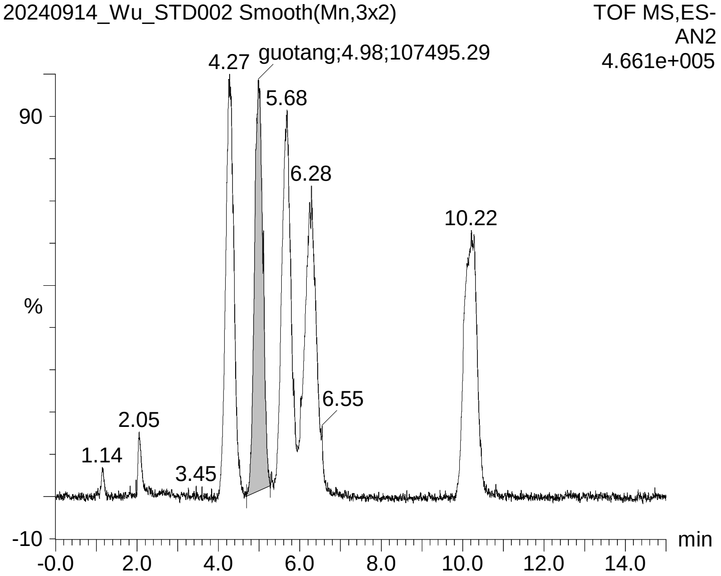

putaotang

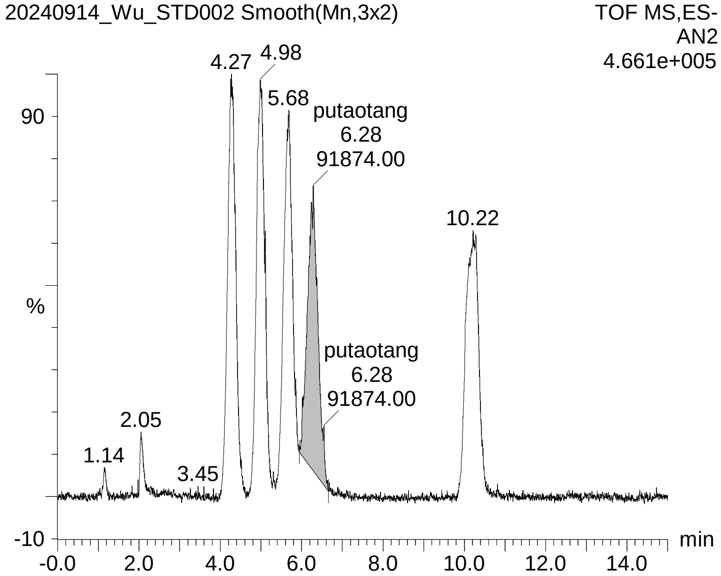

zhetang

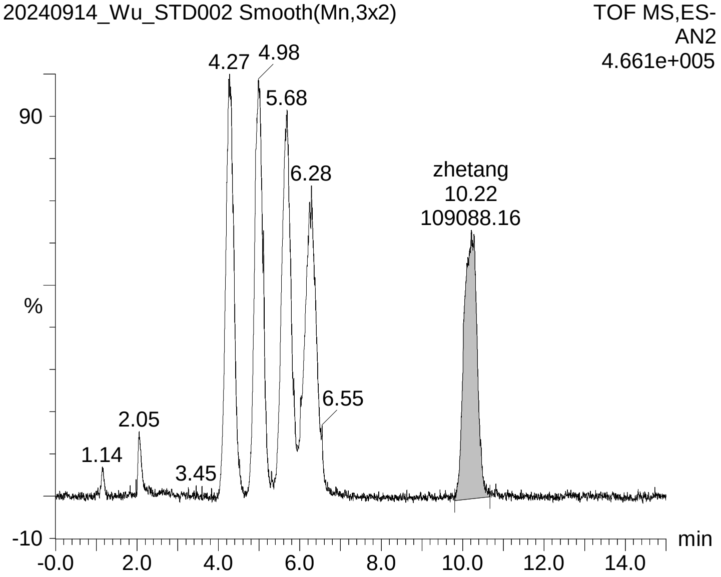

	#	Name	Sample Text	RT	Area	Std. Conc	Conc.
1	1	guotang		4.98	107495.289	80.000	
2	2	putaotang		6.28	91874.000	80.000	
3	3	zhetang		10.22	109088.156	80.000	

project_wangzhonghua_BeiMu
Dataset: F:\data\Wu_yueyan.PRO\20240918-STD.qld
Last Altered: Wednesday, September 18, 2024 14:19:16 China Standard Time
Printed: Monday, January 13, 2025 08:56:42 China Standard Time

Name: 20240914_Wu_STD003, Date: 15-Sep-2024, Time: 09:00:23, ID: , Description:

guotang

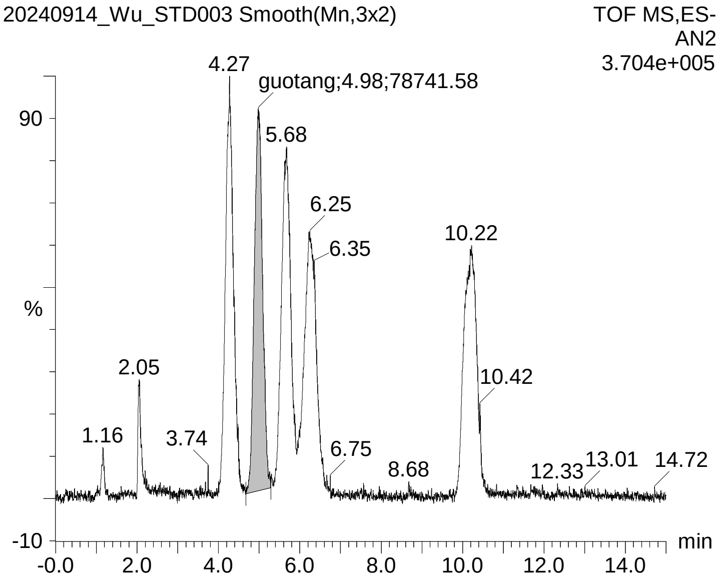

putaotang

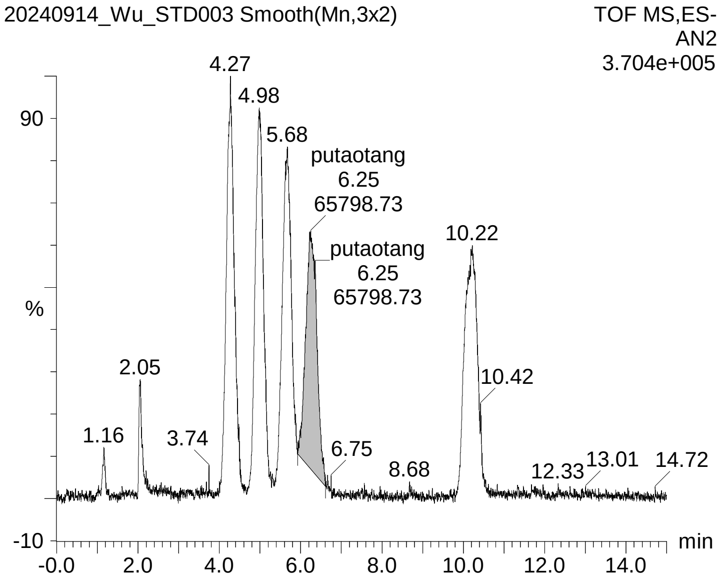

zhetang

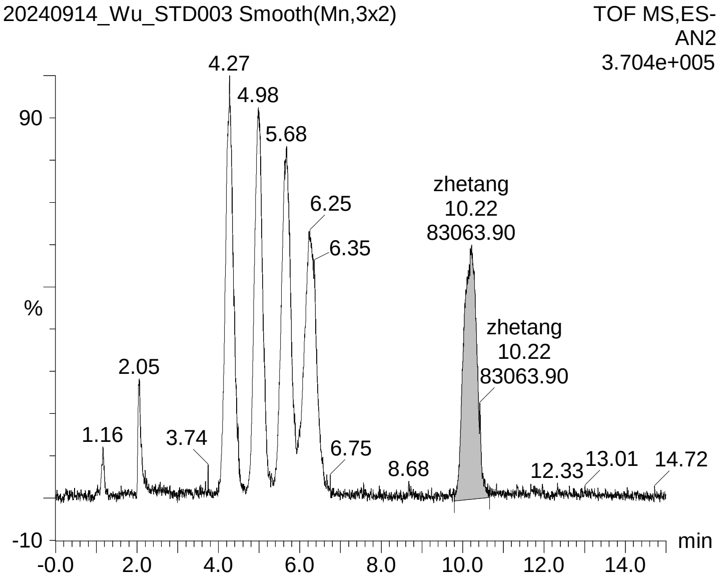

	#	Name	Sample Text	RT	Area	Std. Conc	Conc.
1	1	guotang		4.98	78741.578	64.000	
2	2	putaotang		6.25	65798.727	64.000	
3	3	zhetang		10.22	83063.898	64.000	

project_wangzhonghua_BeiMu
Dataset: F:\data\Wu_yueyan.PRO\20240918-STD.qld
Last Altered: Wednesday, September 18, 2024 14:19:16 China Standard Time
Printed: Monday, January 13, 2025 08:56:42 China Standard Time

Name: 20240914_Wu_STD004, Date: 15-Sep-2024, Time: 09:16:25, ID: , Description:

guotang

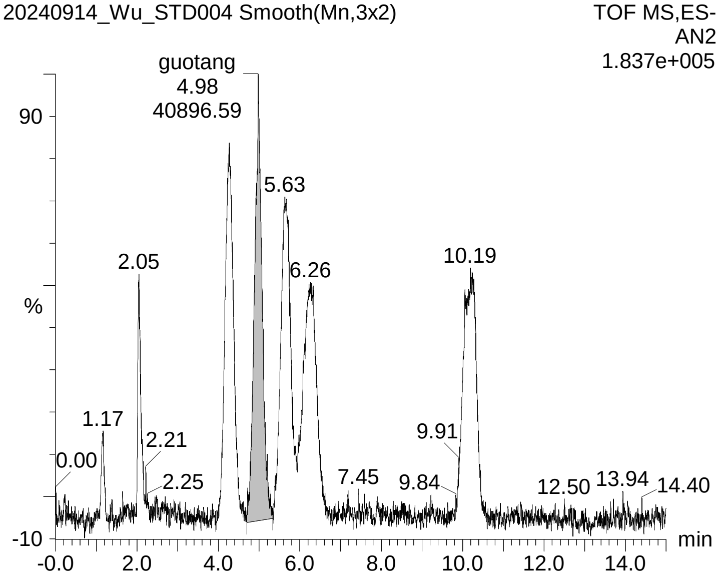

putaotang

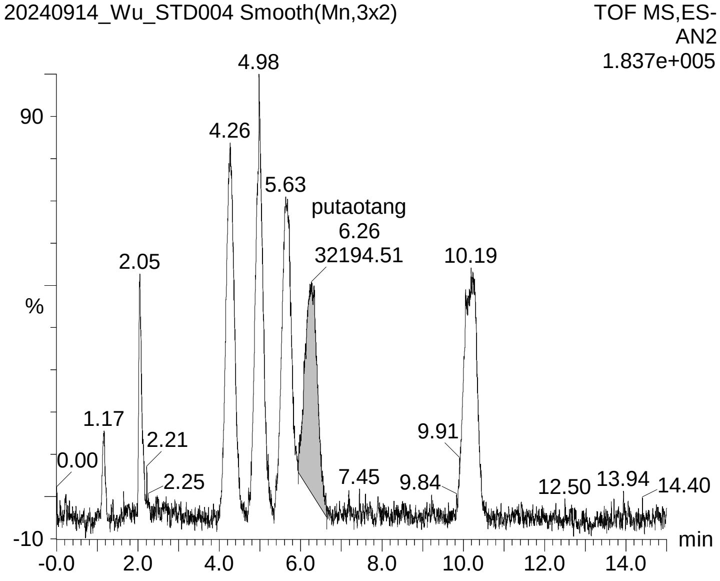

zhetang

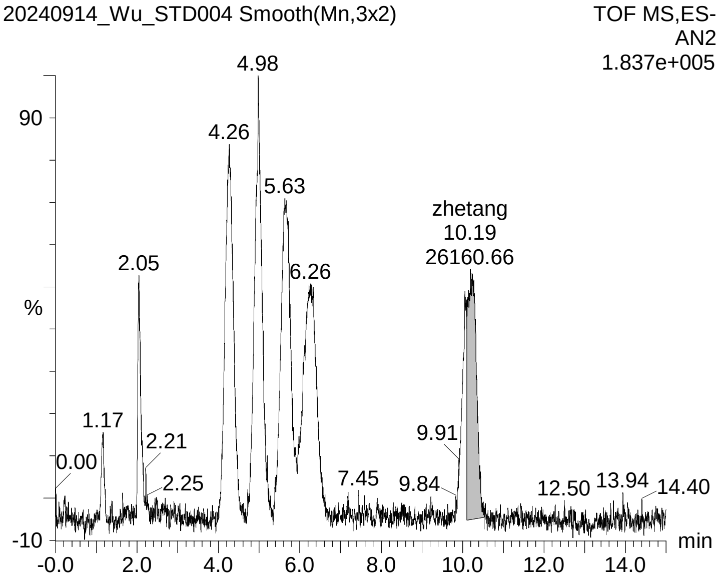

	#	Name	Sample Text	RT	Area	Std. Conc	Conc.
1	1	guotang		4.98	40896.590	40.000	
2	2	putaotang		6.26	32194.514	40.000	
3	3	zhetang		10.19	26160.664	40.000	

project_wangzhonghua_BeiMu

Dataset: F:\data\Wu_yueyan.PRO\20240918-STD.qld

Last Altered: Wednesday, September 18, 2024 14:19:16 China Standard Time

Printed: Monday, January 13, 2025 08:56:42 China Standard Time

Name: 20240914_Wu_STD005, Date: 15-Sep-2024, Time: 09:32:26, ID: , Description:

guotang

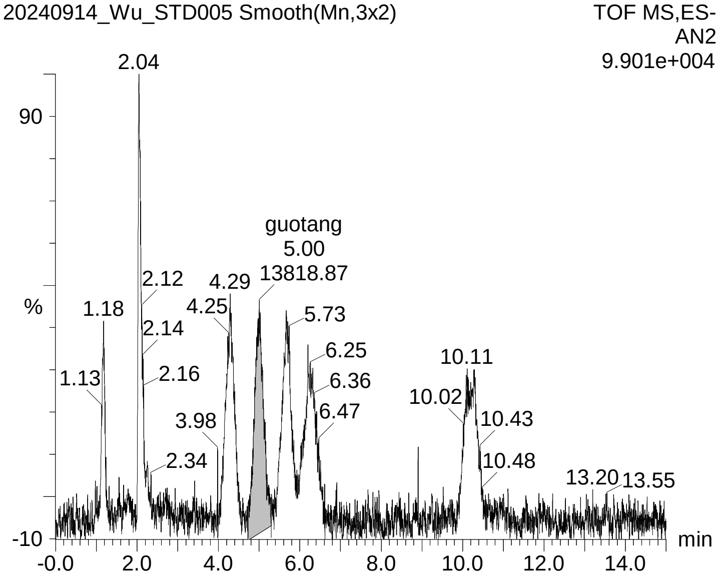

putaotang

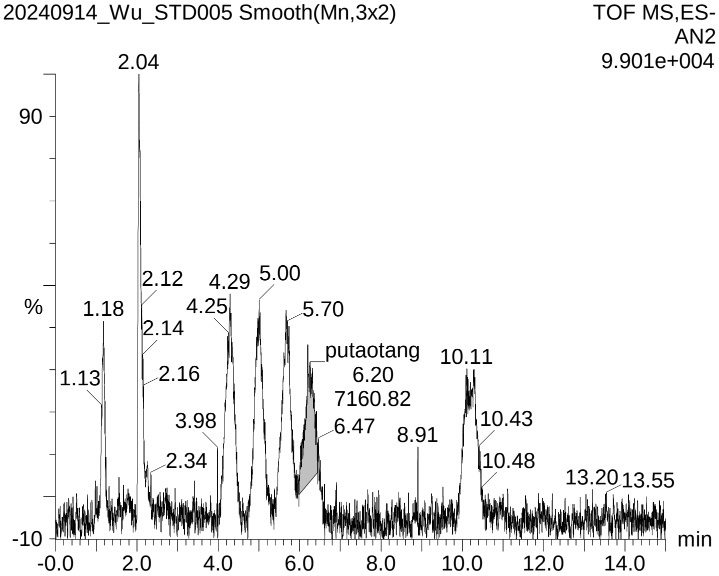

zhetang

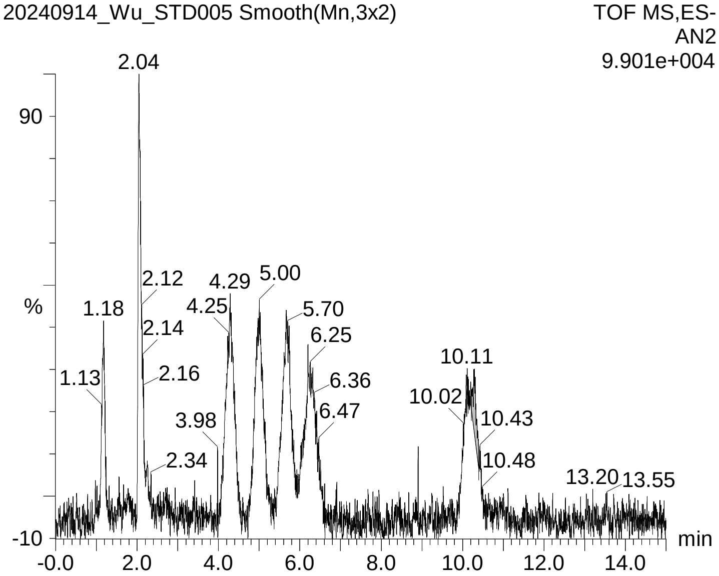

	#	Name	Sample Text	RT	Area	Std. Conc	Conc.
1	1	guotang		5.00	13818.868	16.000	
2	2	putaotang		6.20	7160.820	16.000	
3	3	zhetang		10.29	1422.774	16.000	
